# Flow‐Enabled, Modular Access to α,α‐Difluoromethylene Amines

**DOI:** 10.1002/anie.202517282

**Published:** 2025-10-30

**Authors:** Dmitrii Nagornîi, Pietro Ronco, Khadijah Anwar, Nikolaos Kaplaneris, James J. Douglas, Timothy Noël

**Affiliations:** ^1^ Flow Chemistry Group Van't Hoff Institute for Molecular Sciences (HIMS) University of Amsterdam Science Park 904 XH Amsterdam 1098 The Netherlands; ^2^ Department of Chemistry University of Pavia Viale Taramelli Pavia 27100 Italy; ^3^ Early Chemical Development Pharmaceutical Sciences R&D AstraZeneca, Charter Way Macclesfield SK10 2NA UK

**Keywords:** Difluorination, Flow chemistry, Isosteres, Late‐stage functionalization, Library synthesis

## Abstract

The α,α‐difluoromethylene amine (NCF_2_R) motif represents a useful functionality in medicinal chemistry, yet practical and modular methods to access this class of compounds are lacking. Here, we report a safe and scalable flow‐based strategy for the on‐demand generation of NCF_2_R anions using a packed‐bed microreactor containing caesium fluoride. This protocol enables the late‐stage installation of the CF_2_ group under mild conditions, avoiding the use of hazardous fluorinating agents and minimizing fluorinated waste. This fully modular strategy features three points of diversification (carboxylic acid, sulfonamide, and electrophile), allowing efficient access to a broad range of α,α‐difluoromethylene amines. The method tolerates a variety of functional groups, supports late‐stage functionalization of pharmaceutically relevant scaffolds, and is compatible with downstream cross‐coupling reactions, demonstrating the robustness of the reaction protocol. This work provides a versatile platform for the streamlined incorporation of NCF_2_ motifs, expanding the range of synthetic strategies available in medicinal and fluorine chemistry.

## Graphical abstract

We report a safe and scalable flow‐based strategy for the on‐demand generation of NCF_2_R anions using a packed‐bed microreactor containing CsF. This protocol enables the late‐stage installation of the CF_2_ group under mild conditions leveraging three points of diversification, allowing efficient access to a broad range of NCF_2_R scaffolds.

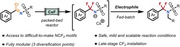

Fluorinated motifs play a pivotal role in pharmaceutical and agrochemical development due to their ability to significantly modulate the physicochemical and biological properties of small molecules.^[^ ^1^, ^2^ ^]^ The incorporation of fluorine atoms into a molecule can improve metabolic resistance, adjust lipophilicity, and enhance bioavailability, making fluorination a widely employed strategy in modern drug discovery (Figure 1a).^[^ ^3^, ^4^ ^]^

**Figure 1:**
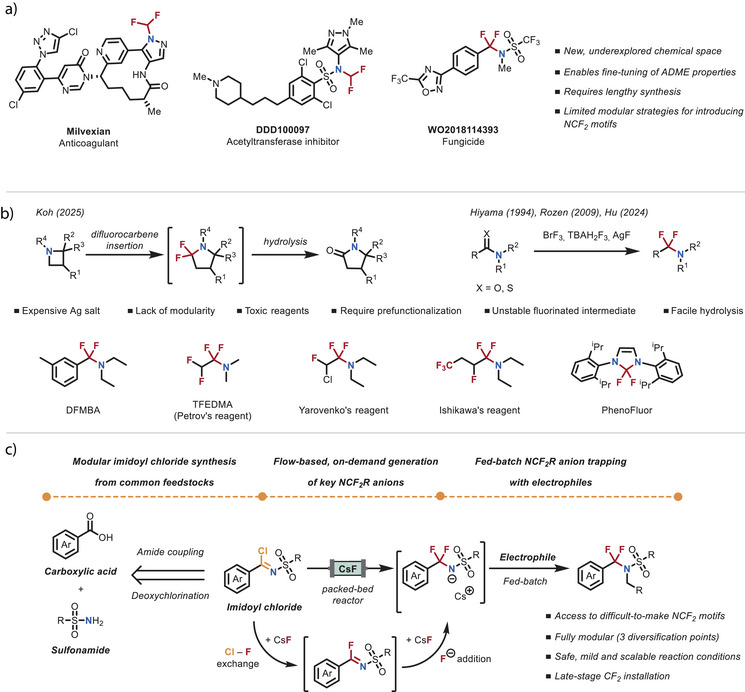
Background and concept. a) Examples of active pharmaceutical ingredients containing NCF_2_ motifs. b) Prior art in the synthesis of NCF_2_R motifs and common deoxyfluorinating reagents. c) Reaction design: modular and scalable access to NCF_2_ motifs using flow‐enabled anion generation and fed‐batch trapping.

Among the various fluorinated groups, the difluoromethylene unit (CF_2_) is particularly attractive as a bioisosteric replacement for methylene (CH_2_) and carbonyl (C = O) groups.^[^ ^5^ ^]^ Traditional approaches for installing difluoromethylene units typically rely on bespoke deoxyfluorinating reagents like DFMBA,^[^ ^6^ ^]^ TFEDMA,^[^ ^7^ ^]^ Yarovenko's reagent,^[^ ^8^ ^]^ Ishikawa's reagent,^[^ ^9^ ^]^ and PhenoFluor,^[^ ^10^ ^]^ or hydrogen fluoride derivatives such as Olah's reagent,^[^ ^11^ ^]^ triethylamine tris(hydrogen fluoride) (Figure 1b).^[^ ^12^ ^]^ Recent advances in difluoromethylation and carbene insertion strategies have provided valuable tools for incorporating such moieties into organic molecules, enabling direct linkages to carbon, oxygen, and sulfur atoms.^[^ ^13^, ^14^, ^15^, ^16^, ^17^, ^18^, ^19^, ^20^ ^]^ In contrast, analogous methods to prepare α,α‐difluoromethylene amines (NCF_2_R, where R ≠ H) remain scarce, primarily due to the inherent difficulty of direct fluoroalkylation of amines and the limited stability of the resulting products (Figure 1b).^[^ ^21^, ^22^, ^23^ ^]^ Notably, practical and scalable methods to access α,α‐difluoromethylene amines with substituents other than hydrogen or fluorine (R ≠ H or F) are highly limited.^[^ ^24^, ^25^, ^26^ ^]^ A survey of AstraZeneca's internal electronic lab notebook highlights the prevalence of NCF_2_R motifs with R═H or F, compared to those bearing other substituents (R ≠ H or F). To the best of our knowledge, the only reliable reported strategy to access this motif involves prefunctionalized thioamides and silver fluoride, as reported by Hu et al., highlighting the need for more flexible, scalable, and widely applicable methods.^[^ ^27^ ^]^ Developing a general, functional group‐tolerant approach to NCF_2_R motifs would significantly expand the chemical space available to medicinal chemists.

To address this need, we developed a safer and more practical strategy employing caesium fluoride (CsF) in a packed‐bed microreactor^[^ ^28^, ^29^, ^30^ ^]^ to efficiently generate NCF_2_R anions under mild conditions (Figure 1c). These anions are generated by passing readily accessible imidoyl chlorides through a packed‐bed reactor filled with CsF. Within this system, a rapid chloride–fluoride (Cl─F) exchange followed by fluoride addition takes place, producing the corresponding anion building blocks on demand and in high yield.^[^ ^31^, ^32^ ^]^ The resulting NCF_2_R anions can then be combined with a variety of electrophiles to afford stable α,α‐difluoromethylene amine compounds. Consequently, this reaction blueprint offers a reliable, scalable, and modular platform to access a wide range of α,α‐difluoromethylene amines. Structural diversification can be rapidly and systematically achieved by varying the benzoic acid or sulfonamide precursors of the imidoyl chloride, or by selecting different electrophilic coupling partners. Importantly, our strategy avoids the use of pre‐fluorinated building blocks and instead installs the CF_2_ moiety at a late stage, thereby significantly reducing the unnecessary generation of fluorinated waste.

Building upon our previous work,^[^ ^33^ ^]^ we developed a novel difluorination strategy starting from substituted imidoyl chlorides. These reagents were readily synthesized through a straightforward, modular two‐step sequence comprising an amide coupling followed by a deoxychlorination step, starting from widely available benzoic acid derivatives and sulfonamides.^[^ ^34^ ^]^ Most of the aryl‐substituted imidoyl chloride reagents prepared in this study could be conveniently isolated as crystalline solids by simple precipitation, while a few required additional purification by column chromatography (see Supporting Information). Furthermore, these compounds proved to be stable under ambient conditions (i.e., at room temperature in air on the benchtop) for several weeks without any noticeable loss of reactivity.

Initial batch experiments revealed that aryl‐substituted imidoyl chloride **1** could be converted into the targeted anion, which subsequently underwent nucleophilic substitution with benzyl bromide (BnBr) to afford the desired product **2** in a 64% isolated yield (see Supporting Information). However, the comparable nucleophilicity of the generated anion and the fluoride ion led to competing side reactions, notably the formation of benzyl fluoride from benzyl bromide. This side reaction consumed a significant portion of the electrophile, making the batch process impractical.

To overcome this limitation, we developed a flow‐based protocol using a packed‐bed reactor loaded with CsF and glass beads as filling material. The flow setup enhanced the efficiency of the Cl─F exchange and subsequent fluoride addition by providing a large excess of CsF, improved mixing, and an expanded surface area, while also offering improved safety by containing the reactive intermediates. Additionally, this flow‐based approach effectively shifted the reaction equilibrium toward the formation of the NCF_2_R anion via a Cl─F exchange followed by fluoride addition. A residence time screening indicated that complete conversion of the imidoyl chloride to the anion was achieved within 15 min (see Table S1). Upon exiting the reactor, the anion was combined with a suitable electrophile in a fed‐batch process. This combination of a packed‐bed reactor and batch reactor also effectively minimized fluoride ion carryover, thereby reducing the competitive formation of benzyl fluoride.

Further finetuning of the nucleophilic substitution step revealed that using an excess of the anion relative to the electrophile significantly improved the yield (Table 1, Entries 1–3, 9). Moderate yields of 41% and 67% were obtained at room temperature when using 1.5 and 3.0 equiv. of the anion, respectively, although these conditions required extended reaction times (Table 1, Entries 4 and 5). Screening various additives (Table 1, Entries 6 and 7; Tables S3 and S4) showed that the addition of 1.1 equivalents of tetrabutylammonium iodide (TBAI) increased the yield to 75% with only mild heating at 40 °C. Further increasing the amount of TBAI, however, did not lead to a significant improvement in yield (Table 1, Entry 8).

**Table 1:** Reaction optimization: Influence of reagent stoichiometry, temperature, and time optimization on the formation of compound **2**.

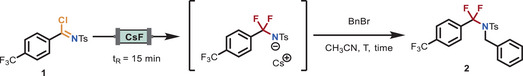						
Entry^a)^	Anion (equiv.)	BnBr (equiv.)	Temperature (°C)	Time (hours)	Additive	Product yield^a)^
1	1.5	1	80	3	–	39%
2	2.0	1	80	3	–	53%
3	3.0	1	80	3	–	46%
4	1.5	1	20	60	–	41%
5	3.0	1	20	60	–	67%
6	3.0	1	40	18	KI (1.5 equiv.)	25%
**7** **^b)^**	**3.0**	**1**	**40**	**18**	**TBAI (1.1 equiv.)**	**75%**
8	3.0	1	40	18	TBAI (3.0 equiv.)	79%
9	1.0	3	40	18	–	25%

^a)^ Yields were determined by ^19^F NMR using 1,2‐difluorobenzene as external standard (0.1 mmol scale).

^b)^ These conditions were selected as optimal and were used to explore the reaction scope.

With the optimized reaction conditions in hand, we next explored the substrate scope. Our reaction design is highly modular, offering three distinct diversification points throughout the synthesis. We began by investigating the first diversification point, i.e., various electrophilic coupling partners were combined with our benchmark imidoyl chloride **1** (Figure 2). Benzyl and allyl bromides bearing diverse functional groups were well‐tolerated. Substituted benzyl bromides containing halogens (F, Cl, Br, and I, **3**–**6**), methoxy (**7**), aldehyde (**8**), nitrile (**9**), ketone (**10**), and ester (**11**) groups successfully reacted with the NCF_2_R anion, delivering the corresponding difluoromethylene amine products in moderate to good yields (49%–77%). The method was readily scalable without reoptimization, affording gram quantities of NCF_2_R compounds, exemplified by the 1‐gram synthesis of compound **5** in 70% yield. Notably, heteroaryl electrophiles such as benzothiophene (**12**), quinoline (**13**), pyridine (**14**), and furan (**15**) also underwent the transformation efficiently, with isolated yields ranging from 44% to 84%. In addition, allylic (**16**–**17**, 67–85%) and propargylic bromides (**18**, 44%) proved to be suitable coupling partners, further showcasing the versatility of the method. Given the importance of N‐methylation in medicinal chemistry, the NCF_2_R anion could also be efficiently trapped with methyl triflate, affording the N‐methylated product and its ^13^C‐labeled analogue (**19**–**20**, 82%–83%).^[^ ^35^ ^]^ Finally, the synthetic utility of the protocol was demonstrated in late‐stage functionalization. Several complex, medicinally relevant molecules, including Rosuvastatin (**21**), Probenecid (**22**), Ataluren (**23**), Repaglinide (**24**), and Menthol (**25**) derivatives, were successfully functionalized, affording the corresponding products in synthetically useful yields (21%–64% isolated yields). These examples underscore the potential of our modular, flow‐enabled approach for the rapid generation of diverse, pharmacologically relevant NCF_2_R compounds.

**Figure 2:**
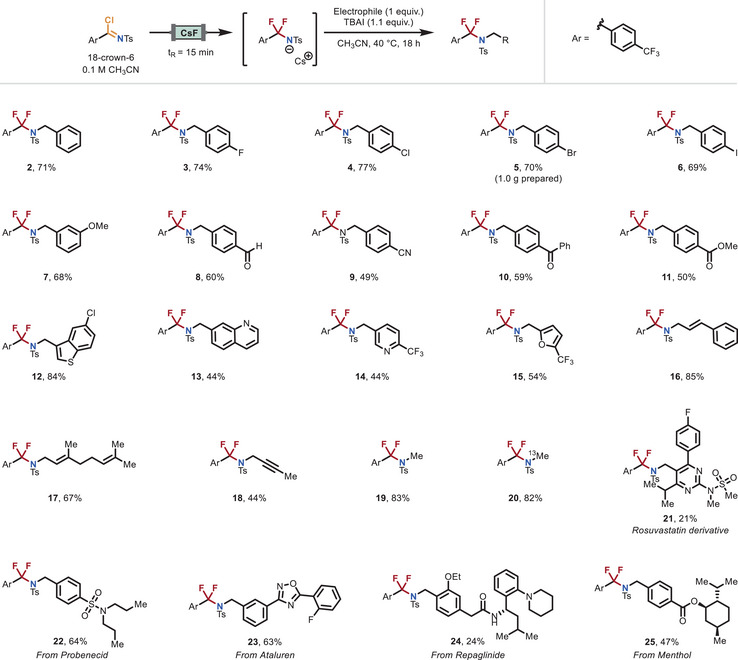
Electrophile scope of the difluorinative functionalization reaction. Reaction conditions: A 0.1 M solution of imidoyl chloride and 18‐crown‐6 in dry CH_3_CN was passed through the CsF packed‐bed reactor at a flow rate of 0.22 mL min^−1^ (residence time of 15 min). The outflow (6 mL, 3 equiv.) was collected in an oven‐dried vial containing TBAI (1.1 equiv.) and the corresponding electrophile (if solid) (1 equiv., 0.2 mmol). Upon collection of the desired amount of outflow, the electrophile (if liquid) (0.2 mmol, 1 equiv.) was added, and the reaction was stirred at 40 °C for 18 h. For further experimental details, see the Supporting Information.

Medicinal chemistry benefits from synthetic strategies that offer robust functional group tolerance, flexible vector modification, and straightforward compound preparation to accelerate drug discovery. To demonstrate this potential, and as the second diversification point in our modular platform, we explored the variation of the imidoyl chloride fragment, focusing specifically on the carboxylic acid building block (Figure 3). Following the established protocol, various imidoyl chlorides, readily prepared from substituted benzoic acids or acyl chlorides, were passed through the CsF packed‐bed reactor and subsequently reacted with cinnamyl bromide in a fed‐batch process. Substrates bearing electron‐withdrawing groups generally outperformed those with no substituents (**29**, 53%) or electron‐donating groups (see Supporting Information). This trend is likely due to the increased electrophilicity of the imidoyl chloride, which facilitates the nucleophilic attack by the fluoride anion and promotes a more efficient generation of the desired NCF_2_R anion. A broad range of electron‐withdrawing substituents was well–tolerated, including halides (F, Cl, Br; **26**–**28**), trifluoromethyl (**7**), trifluoromethoxy (**30**), nitrile (**31**), methyl ester (**32**), and methyl sulfone (**33**) groups, providing the corresponding products in 57%–85% yields. Additionally, when thiophene derivative **34** was subjected to the developed protocol, the desired product was obtained in good yield (60%). To further demonstrate the synthetic utility of the method, we applied the protocol to the imidoyl chloride derivative of the marketed drug Probenecid (**35**), which was successfully functionalized under the optimized conditions, delivering the product in 71% yield.

**Figure 3:**
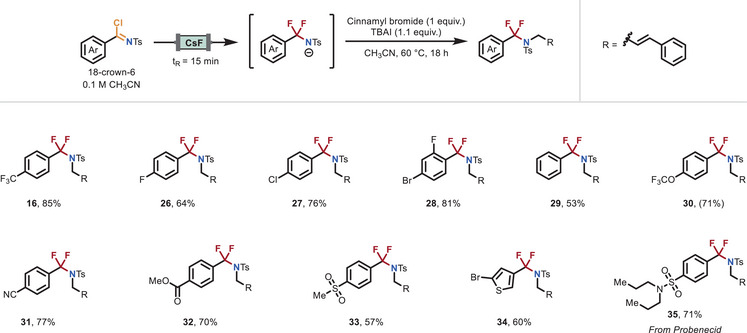
Scope of the difluorinative functionalization reaction starting from various benzoic acid or acyl chloride derivatives. Reaction conditions: A 0.1 M solution of imidoyl chloride and 18‐crown‐6 in dry CH_3_CN was passed through the CsF packed‐bed reactor at a flow rate of 0.22 mL min^−1^ (residence time of 15 min). The outflow (6 or 8 mL, 3 or 4 equiv.) was collected in an oven‐dried vial containing TBAI (1.1 equiv.). Upon collection of the desired amount of outflow, cinnamyl bromide (0.2 mmol, 1 equiv.) was added, and the reaction was stirred at 60 °C for 18 h. For further experimental details see the Supporting Information.

As the third diversification point in our modular platform, we explored the variation of the last fragment using a range of commercially available sulfonamides (Figure 4). A variety of functional groups commonly used in organic synthesis, including tosyl (**7**), nosyl (**39**), mesyl (**43**), and triflyl (**44**), were successfully incorporated under the optimized conditions. Substitution on the aromatic ring of the sulfonamide was well tolerated, with substrates bearing chloride (**36**), fluoride (**37**), and methoxy (**38**) groups efficiently participating in the transformation. In addition, several heterocyclic sulfonamides, such as pyrazole (**40**) and thiophene derivatives (**41**–**42**), proved to be compatible coupling partners. The utility of the method was further demonstrated through the successful functionalization of sulfonamide derivatives of the nonsteroidal anti‐inflammatory drugs Valdecoxib (**45**) and Deracoxib (**46**), highlighting the potential of this strategy for late‐stage modification of complex, pharmacologically relevant molecules.

**Figure 4:**
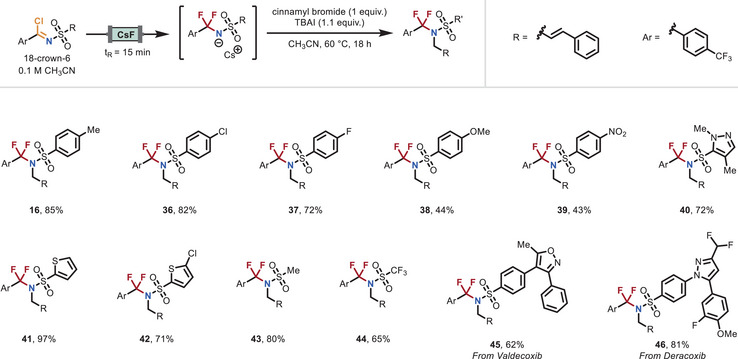
Scope of the difluorinative functionalization reaction starting from various sulfonamides. Reaction Conditions: A 0.1 M solution of imidoyl chloride and 18‐crown‐6 in dry CH_3_CN was passed through the CsF packed‐bed reactor at a flow rate of 0.22 mL min^−1^ (residence time of 15 min). The outflow (6 or 8 mL, 3 or 4 equiv.) was collected in an oven‐dried vial containing TBAI (1.1 equiv.). Upon collection of the desired amount of outflow, cinnamyl bromide (0.2 mmol, 1 equiv.) was added, and the reaction was stirred at 60 °C for 18 h. For further experimental details see the Supporting Information.

Gem‐difluoro derivatives are well‐established bioisosteres for carbonyl and methylene groups.^[^ ^5^, ^36^, ^37^, ^38^, ^39^, ^40^, ^41^ ^]^ To showcase the synthetic utility of our methodology, we applied our difluorinative protocol to the selective editing of various biologically active compounds (Figure 5a,b).

**Figure 5:**
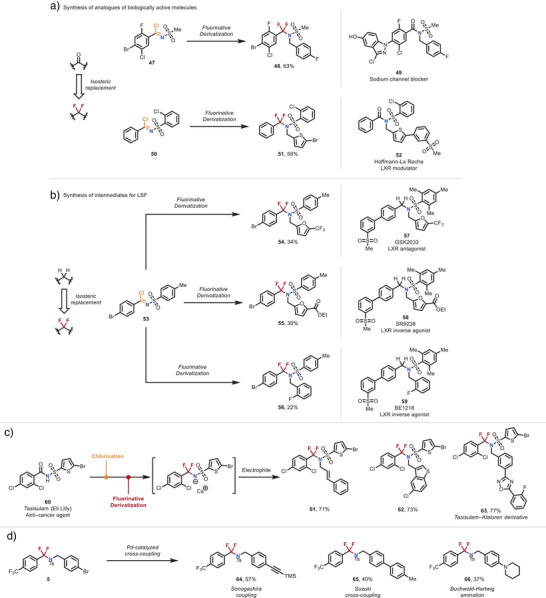
Late‐stage diversification strategies. a) Strategic replacement of carbonyl motifs with the difluoromethylene unit and application to the formal synthesis of fluorinated analogues of drug molecules. b) Replacement of methylene motifs with the difluoromethylene unit. c) Rapid fluorinative functionalization of Tasisulam using the developed methodology. d) Product post‐functionalization with traditional cross‐coupling methods.

For example, imidoyl chloride **47** was converted to α,α‐difluoromethylene amine **48**, which could be subsequently transformed into a difluoro analogue of compound **49**, a sodium channel blocker used for the treatment and prevention of pain.^[^ ^42^ ^]^ Similarly, imidoyl chloride **50** was reacted with a bromo‐thiophene derivative to afford product **51**, which could then be further elaborated to deliver a difluoro analogue of compound **52**, a ligand known to modulate liver X receptors alpha (LXR‐α) and beta (LXR‐β).^[^ ^43^ ^]^

Furthermore, using imidoyl chloride **53** as a common precursor, we synthesized difluoro analogues (**54**–**56**) of several LXR modulators, including GSK2033, SR9238, and BE1218 (**57**–**59**).^[^ ^44^, ^45^, ^46^ ^]^ This transformation was enabled by processing a single starting material through the packed‐bed reactor and sequentially combining the resulting NCF2R anion with three different electrophiles. This streamlined sequence highlights the modular nature of our method, which is particularly well‐suited for library synthesis.

To further demonstrate the versatility of our strategy, we developed a direct transformation of biologically active amide compounds into α,α‐difluoromethylene amines through sequential deoxygenative chlorination followed by our difluorination protocol (Figure 5c). As an illustrative example, Tasisulam (**60**), an antitumor agent known to inhibit mitotic progression and induce vascular normalization, was subjected to this sequence, affording a series of difluoro derivatives (**61**–**63**), including a Tasisulam–Ataluren hybrid (**63**).^[^ ^47^ ^]^

We also evaluated the stability of the α,α‐difluoromethylene amine motif under typical cross‐coupling conditions (Figure 5d). Derivatizations such as alkynylation, arylation, and amination were successfully performed using standard, unoptimized Sonogashira, Suzuki‐Miyaura, and Buchwald–Hartwig protocols (**64**–**66**). These results underscore the robustness of the α,α‐difluoromethylene amine structure under strongly basic, nucleophilic, high‐temperature, and palladium‐catalyzed conditions.

In summary, we have developed a modular, scalable, and flow‐enabled strategy for the synthesis of α,α‐difluoromethylene amines (NCF_2_R) under mild and practical conditions. This methodology addresses key limitations in the field by avoiding pre‐fluorinated building blocks, hazardous reagents, and multi‐step syntheses, while enabling late‐stage installation of the CF_2_ unit. The platform features three distinct points of diversification, including carboxylic acid, sulfonamide, and electrophilic coupling partner, providing broad functional group tolerance, and facilitating the rapid generation of structurally diverse NCF_2_R compounds. The versatility of the approach was demonstrated through the selective difluorination of complex, bioactive molecules, library synthesis from a common precursor, and successful downstream functionalization via cross‐coupling reactions. Collectively, these advances offer a powerful tool for the synthesis and exploration of difluorinated amine chemical space, with significant potential to accelerate the discovery and development of next‐generation fluorinated therapeutics.

## Supporting Information

The authors have cited additional references within the Supporting Information.^[^ ^33^, ^48^, ^49^, ^50^, ^51^ ^]^

## Conflict of Interests

The authors declare no conflict of interest.

## Supporting information



Supporting Information

## Data Availability

The data that support the findings of this study are available in the Supporting Information of this article.
