# Supplementary material for: Flow‐Enabled, Modular Access to α,α‐Difluoromethylene Amines
Source: Angew Chem Int Ed Engl. 2025 Oct 30;64(52):e17282. doi: 10.1002/anie.202517282 (PMC12723450; doi:10.1002/anie.202517282)
Supplement: Supplementary file 1 — Supporting Information [file ANIE-64-e17282-s001.pdf]

Supplementary Materials for

Flow-Enabled, Modular Access to α,α-Difluoromethylene Amines

Dmitrii Nagornîi[1], Pietro Ronco[1,2], Khadijah Anwar[1], Nikolaos Kaplaneris[1], James J. Douglas[3], Timothy Noël[1,*].

[1] Flow Chemistry Group, Van 't Hoff Institute for Molecular Sciences (HIMS), University of Amsterdam, Amsterdam, The Netherlands.

[2] Department of Chemistry, University of Pavia, Viale Taramelli, 27100 Pavia, Italy

[3] Early Chemical Development, Pharmaceutical Sciences R&D, AstraZeneca, Macclesfield, UK.

*Correspondence to: T.Noel@uva.nl.

Table of Contents

1. General Information ... 3
1.1 Materials ... 3
1.2 Safety Considerations ... 3
1.3 Packed-Bed Reactor Preparation ... 4
1.4 Preparation of 18-crown-6 Solution ... 4
2. Optimizations ... 5
2.1 Preliminary Batch Experiments ... 5
2.2 Optimization of the $TsNCF_2R$ Anion Generation in Flow ... 5
2.3 Optimization of Fed Batch Step ... 6
2.3.1 Optimization of Reaction Stoichiometry, Temperature, and Time ... 6
2.3.2 Optimization of Additives ... 7
2.3.3 Optimization with TBAI ... 7
2.4 Life-Span Assessment for the CsF Packed-bed Reactor ... 8
2.5 Limitations the developed method ... 9
3. Experimental Procedures ... 10
3.1 General Procedure (A): Synthesis of Imidoyl Chlorides from Acyl Chlorides ... 10
3.2 General Procedure (B): Synthesis of Imidoyl Chlorides from Carboxylic Acids ... 10
3.3 General procedure (C): Synthesis of NCF_2R Compounds from Different Electrophiles ... 10
3.4 General procedure (D): Synthesis of NCF_2 Compounds from Different Acyl Chlorides/Sulfonamides ... 11
3.5 Synthesis of Benzyl Bromide Derivatives ... 11
4. Characterization data ... 14
4.1 Starting materials ... 14
4.2 Scope of Electrophiles ... 23
4.3 Scope of Aryl Groups ... 33
4.4 Scope of Sulfonamides ... 37
4.5 Applications ... 42
5. NMR Spectra ... 47
5.1 Benzyl Bromide Derivatives ... 47
5.2 Imidoyl Chlorides ... 51
5.2 Scope of Electrophiles ... 93
5.3 Scope of Aryl Groups ... 141
5.5 Scope of Sulfonamides ... 161
5.6 Applications ... 183
6. References ... 205

1. General Information

^{1}H (300 MHz), ^{13}C (75 MHz) and ^{19}F (282 MHz) spectra were recorded at ambient temperature using Bruker AV 300-I. ^{1}H NMR spectra are reported in parts per million (ppm) downfield relative to $CDCl_3$ (7.26 ppm) and all ^{13}C NMR spectra are reported in ppm relative to $CDCl_3$ (77.16 ppm). The multiplicities of signals are designated by the following abbreviations: s (singlet), d (doublet), t (triplet), q (quartet), p (pentet), m (multiplet), dd (doublet of doublets), dt (doublet of triplets), td (triplet of doublets), ddd (doublet of doublet of doublets), br s (broad singlet). Coupling constants (J) are reported in hertz (Hz). NMR data was processed using the MestReNova 14 software package. High resolution mass spectra (HRMS) were collected on an AccuTOF LC, JMS-T100LP Mass spectrometer (JEOL, Japan) or on an AccuTOF GC v 4g, JMS-T100GCV Mass spectrometer (JEOL, Japan), or on a 7200 GC−qTOF (Agilent Technologies). Disposable syringes were purchased from Laboratory Glass Specialist. Syringe pumps were purchased from Chemix Inc. model Fusion 200 Touch. Product isolation was performed automatically, by a Biotage® Isolation Four, with Biotage® Ultra C18 10 g flash chromatography cartridges, or manually, using silica (P60, SILICYCLE). TLC analysis was performed using Silica on aluminum foils TLC plates (F254, SILICYCLE) with visualization under ultraviolet light (254 nm and 365 nm). Organic solutions were concentrated under reduced pressure on a Büchi rotary evaporator (in vacuo at 40 °C, ~5 mbar).

1.1 Materials

All the solvents were used as received without further purification. Reagents and solvents were purchased from Sigma Aldrich, TCI, abcr, BLD Pharma and Fluorochem. Technical solvents were purchased from VWR International and used as received. Empty cartridges used for the packed-bed reactor were purchased at Screening Devices (catalogue number SD-0000-004). Dry CH_3CN was purchased from Fisher Scientific (Landsmeer, The Netherlands). Flow module was assembled using PFA tubing (ID = 0.8 mm, OD = 1.6 mm) and PEEK connectors.

(1R,2S,5R)-2-isopropyl-5-methylcyclohexyl 4-(bromomethyl)benzoate (SI-1)[1], 4-(bromomethyl)-N,N-dipropylbenzenesulfonamide (SI-2)[2], 3-(3-bromophenyl)-5-(2-fluorophenyl)-1,2,4-oxadiazole (SI-3)[3] and 2-bromo-5-(bromomethyl)thiophene[4] were prepared according to reported literature procedures.

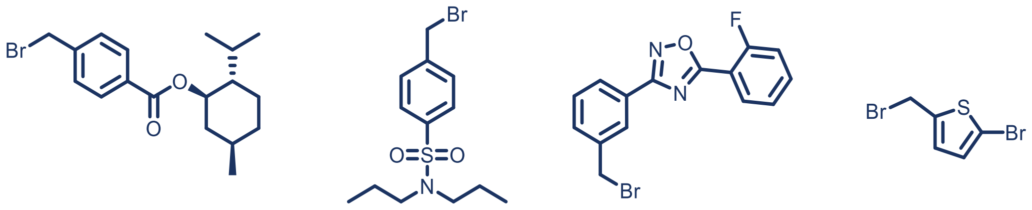

SI-1 **SI-2** **SI-3** **SI-4**

Figure S1. Starting materials synthesized for the scope of the methodology.

1.2 Safety Considerations

The reported methodology utilizes fluoride salts, which can produce hydrofluoric acid (HF) upon contact with acidic mediums. HF is an extremely corrosive and lethal substance through inhalation, ingestion, or skin and eye contact. In the case of skin exposure, there may be a latency period of up to 24 hours for diluted solutions. It is strongly recommended to keep a 2.5% calcium gluconate gel on hand as a first aid measure for potential burns. Prior to conducting any reaction involving HF precursors, chemists should consult Material Safety Data Sheets (MSDS) for HF and review first aid procedures. Wearing personal protective equipment and working under the fume hood is mandatory.

1.3 Packed-Bed Reactor Preparation

Similar to our previous work[5], an empty polypropylene (PP) cartridge for automated flash chromatography was used as a packed-bed reactor module.* The cartridge was loaded with a dried mixture of caesium fluoride (CsF) and glass beads (425–600 µm) in a weight ratio of 7:3. This mixture was prepared by mixing previously ground CsF and glass beads in a Schlenk flask, followed by overnight drying at 300 ºC under vacuum (0.02 to 1 mbar). During the drying process, CsF is prone to forming aggregates that are challenging to break apart upon cooling. This can be mitigated by manually breaking the aggregates by using a spatula or a glass rod under a stream of nitrogen. The cartridge was then completely filled with this mixture (approximately 15.8 g) with gentle tapping against a flat surface to ensure optimal packing. A frit, included with the cartridge, was then used to seal the top and the Luer cap was attached. The CsF-packed cartridges were used immediately after preparation. The dead volume of the reactor was determined by weighting the filled cartridge before and after flushing it with dry CH_3CN and dividing the mass difference by the density of CH_3CN (0.786 g mL-1). For the sake of clarity, averaged dead volume (3.3 mL) will be used throughout the rest of the document. The CsF packed cartridges, filled with dry CH_3CN, were kept under nitrogen until they were used for the reaction (Figure S2). As the dead volume of the reactor would slightly vary each time, the flow rates used were calculated for each cartridge in order to satisfy targeted residence time.

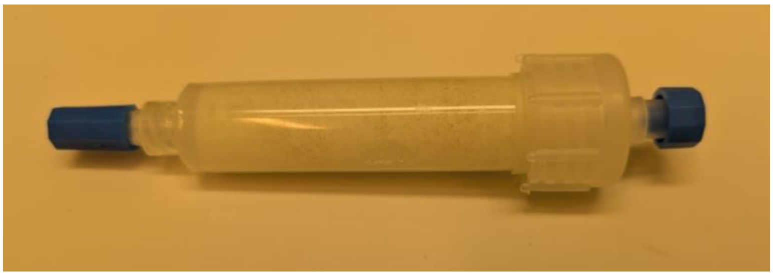

Figure S2. Packed-bed reactor used in this study.

1.4 Preparation of 18-crown-6 Solution

In a 250 mL round-bottom flask, 18-crown-6 (5.30 g, 20.0 mmol) and propan-2-ol were charged (ca. 10.0 mL). The solution was then concentrated under reduced pressure at 40 ˚C in a rotary evaporator system. Next, the resulting 18-crown-6 was dried under high vacuum for 15 minutes. This procedure was repeated 3 more times for azeotropic drying, and after the last cycle 18-crown-6 was left under the high vacuum overnight. Next, the flask was backfilled with nitrogen, and 18-crown-6 was dissolved in dry CH_3CN (200 mL). Then, the 0.1 M 18-crown-6 solution was transferred to another flask containing activated 3 Å molecular sieves. 18-Crown-6 solutions can be stored for extended periods of time, but freshly prepared solutions usually provided the best results and are recommended to ensure the highest reaction yields and reproducible results.

* The PP cartridge was purchased form Screening Devices company: 4g, Double Luer Lock Top and Bottom Empty Solid Load Cartridge with Screw Cap, frits, O-ring, and end tips, 20p (catalogue number SD-0000- 004).

2. Optimizations

2.1 Preliminary Batch Experiments

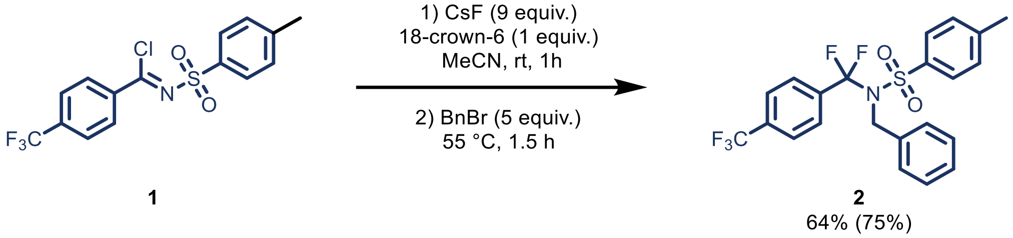

Figure S3. Synthesis of NCF_2R compound **1** in batch.

A 7 mL vial containing a magnetic stirring bar was charged with CsF (273 mg, 1.8 mmol, 9 equiv.). The inorganic salt was then dried in the vial by heating it with a heat gun under vacuum (300 ºC, 5 minutes). Then, the flask was backfilled with nitrogen and left cooling down at room temperature. A solution of the corresponding imidoyl chloride (72 mg, 0.2 mmol, 1 equiv.) and 18-crown-6 ether (52.8 mg, 0.2 mmol, 1 equiv.) in anhydrous CH_3CN (2 mL, 0.1 M) was added to the vial and stirred for 1 hour at room temperature. Next, benzyl bromide (118 µL, 1 mmol, 5 equiv.) was added to the vial and the solution was stirred at 55 °C for 1.5 hours. Then, 1,2-difluorobenzene (19.7 µL, 0.2 mmol) was added and an aliquot was taken to measure the amount of product formed by quantitative ^{19}F NMR (75%). The crude was purified using column chromatography to confirm the formation of the desired product (64%).

2.2 Optimization of the $TsNCF_2R$ Anion Generation in Flow

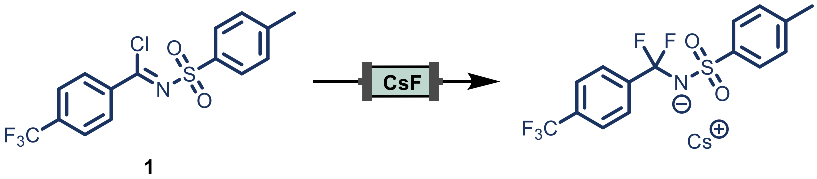

Figure S4: Formation of the intermediate NCF_2R anion.

The optimization of the generation of the $TsNCF_2R$ anion was performed by feeding a 0.1 M solution of imidoyl chloride and 18-crown-6 ether in dry CH_3CN to the packed-bed reactor at different flow rates and determining the yield of the reaction by quantitative ^{19}F NMR (Table S1). Solutions were prepared by dissolving the appropriate amount of the imidoyl chloride in the 18-crown-6 solution, the preparation of which is described above, under nitrogen. Three empty volumes of the reactor (ca. 10 mL) were discarded prior to the collection to ensure equilibration of the system. Samples were prepared by feeding the reaction stream (0.500 mL) into a N_2 filled oven-dried NMR tube, containing 1,2-difluorobenzene as the external standard. Conversion was analysed by ^{19}F NMR. A residence time of 15 minutes was chosen as optimal and used for all further reactions.

Table S1. Flow rate optimization for the generation of the $TsNCF_2R$ anion.

Entry	Residence time (min)	Flow rate (mL/min)	Anion Yield (%)
1	5 min	0.63	68%
2	10 min	0.32	92%
3	15 min	0.21	99%

2.3 Optimization of Fed Batch Step

2.3.1 Optimization of Reaction Stoichiometry, Temperature, and Time

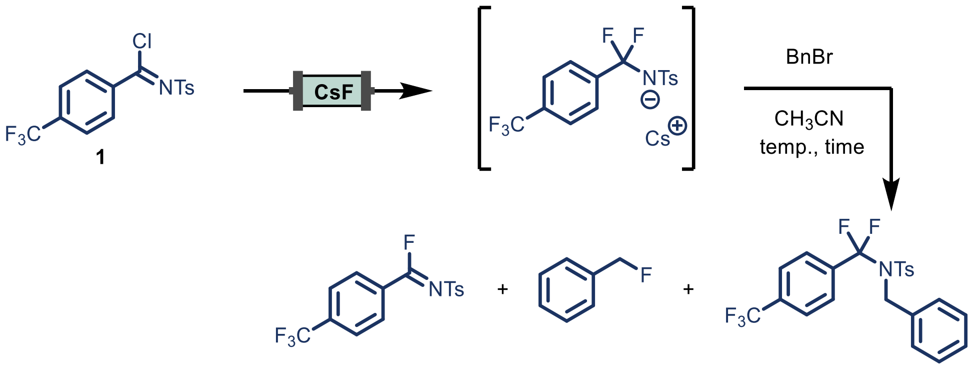

Figure S5: Formation of compound **2**, benzyl fluoride and F exchange product in a fed batch reaction.

An extensive optimization of the reaction parameters of the fed batch step was performed (Table S2). Increasing the equivalents of anion added to the reaction resulted in an increased yield (Entry 3, 6 and 12). Increasing the temperature resulted in higher yields of the desired product, but also decomposition of the anion and the formation of benzyl fluoride byproduct. Lower temperatures din not yield satisfying results, unless extremely long reaction times were used (Entry 8). Using an excess amount of benzyl bromide (Entry 13 and 14) did not result in the desired product in a sufficient yield.

Table S2: Stoichiometry, temperature, and time optimization for the formation of compound **1**.

Entry[a]	Anion (equiv.)	BnBr (equiv.)	Temperature (°C)	Time (hours)	Product	BnF	F exchange	Anion left
1	1.5	1	20	3	**14%**	3%	11%	112%
2	1.5	1	20	60	**41%**	11%	72%	–
3	1.5	1	80	3	**39%**	39%	77%	–
4	2	1	40	3	**28%**	15%	44%	60%
5	2	1	60	3	**38%**	31%	73%	–
6	2	1	80	3	**53%**	52%	57%	–
7	3	1	20	3	**13%**	4%	–	250%
8	3	1	20	60	**67%**	12%	84%	60%
9	3	1	40	3	**24%**	10%	12%	106%

10	3	1	40	18	**48%**	16%	38%	–
11	3	1	60	3	**52%**	44%	109%	42%
12	3	1	80	3	**46%**	46%	43%	5%
13	1	3	40	18	**25%**	12%	20%	–
14	1	5	40	18	**28%**	14%	22%	–

[a]Reaction conditions: A 0.1 M solution of imidoyl chloride and 18-crown-6 in dry CH_3CN was passed through the packed-bed reactor at a flow rate of 0.22 mL min^{-1} (residence time of 15 minutes). The appropriate amount of outflow (1-3 mL, 1-3 equiv.) was collected in an oven-dried vial. The corresponding amount of BnBr (1-5 equiv.) was added, and the reaction was stirred at the stated temperature for the corresponding time. Then, 1,2-difluorobenzene was added and an aliquot was taken to measure the amount of product formed by quantitative ^{19}F NMR.

2.3.2 Optimization of Additives

Various additives were screened to determine their effects on the reaction yield (Table S3). Only the addition of tetra-*n*-butylammonium iodide (TBAI) was observed to have a beneficial effect on the reaction yield, and further optimizations focused on optimizing the amount of TBAI added. The addition of AgOTf, NaI and KI were found to be detrimental to the reaction yield.

Table S3: Additive optimization for the formation of compound **1**

**Entry[a]**	**Additive**	**Product**	**BnF**	**F exchange**	**Anion left**
**1[b]**	AgOTf (1.1 equiv.)	**2%**	22%	183%	7%
**2**	NaI (1.5 equiv.)	–	–	160%	–
**3**	KI (1.5 equiv.)	**25%**	4%	132%	–
**4**	TBAI (1.5 equiv.)	**71%**	16%	92%	–

[a]Reaction conditions: A 0.1 M solution of imidoyl chloride and 18-crown-6 in dry CH_3CN was passed through the packed-bed reactor at a flow rate of 0.22 mL min^{-1} (residence time of 15 minutes). The appropriate amount of outflow (3 mL, 3 equiv.) was collected in an oven-dried vial containing the corresponding additive (1.1 or 1.5 equiv.). BnBr (1 equiv.) was added, and the reaction was stirred at 40 °C for 18h. Then, 1,2-difluorobenzene was added and an aliquot was taken to measure the amount of product formed by quantitative ^{19}F NMR. [b]Reaction time: 3 hours.

2.3.3 Optimization with TBAI

The reaction temperature, the amount of anion and TBAI in the reaction mixture were screened to obtain the highest reaction yield (Table S4). Temperature screening revealed that increased temperature (40°C, Table S3, Entry 4 and 60°C, Table S4, Entry 2) resulted in similar yields (71% and 68% respectively). Increasing the equivalents of TBAI added (Entry 3,4 and 5) resulted in a slightly increased reaction yield, which was deemed insufficient to justify the large excess of TBAI salt. Hence, 1.1 equivalents of TBAI were chosen as the best reaction condition and used for all the following reactions. Lowering or increasing the amount of NCF_2R anion added did not yield improved results.

Table S4: Optimization of reaction temperature, the amount of anion and TBAI added

**Entry[a]**	**Anion (equiv.)**	**TBAI (equiv.)**	**Temperature (°C)**	**Product**	**BnF**	**F exchange**	**Anion left**

**1**	3	1.5	20	**56%**	4%	56%	54%
**2**	3	1.5	60	**68%**	26%	98%	–
**3**	3	1.1	40	**75%**	16%	86%	–
**4**	3	2	40	**77%**	10%	70%	17%
**5**	3	3	40	**79%**	14%	68%	24%
**6**	1.1	1.5	40	**44%**	10%	16%	–
**7**	2	1.5	40	**62%**	12%	44%	–
**8**	4	1.5	40	**72%**	18%	88%	113%

[a]Reaction conditions: A 0.1 M solution of imidoyl chloride and 18-crown-6 in dry CH_3CN was passed through the packed-bed reactor at a flow rate of 0.22 mL min^{-1} (residence time of 15 minutes). The appropriate amount of outflow (1.1–4 mL, 1.1–4 equiv.) was collected in an oven-dried vial containing TBAI (1.1–3 equiv.). BnBr (1 equiv.) was added, and the reaction was stirred at the corresponding temperature for 18h. Then, 1,2-difluorobenzene was added and an aliquot was taken to measure the amount of product formed by quantitative ^{19}F NMR.

2.4 Life-Span Assessment for the CsF Packed-bed Reactor

The lifespan of the cartridge was assessed using the optimized residence time of 15 min (flow rate of 0.22 mL min^{-1}). A 0.1 M solution of imidoyl chloride and 18-crown-6 in dry CH_3CN was passed through the CsF/glass beads cartridge. The outflow was collected in a N_2 filled, over-dried NMR tube, containing 1,2-difluorobenzene as an external standard. The generation of the anion was measured by collecting 1 mL of reaction mixture at appropriate time intervals and analysing it using quantitative ^{19}F NMR. A decrease in the yield of the generated anion was observed after passing 5 mmol of starting material, suggesting that the cartridge is starting to be exhausted (Figure S6).

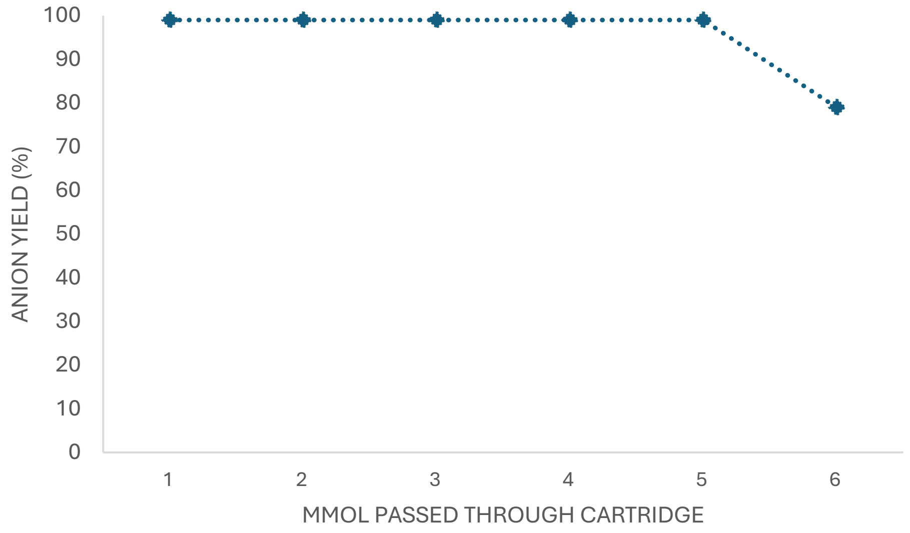

Figure S6. Life-span cartridge assessment for the CsF cartridge.

2.5 Limitations the developed method

During the development of the difluorination reaction, we encountered several limitations in the reaction scope. Electrophiles containing basic nitrogen atoms were poorly tolerated. Screening of the carboxylic acid component revealed that only aromatic groups with neutral or electron-withdrawing substituents were compatible. In contrast, electron-donating groups, such as methoxy, failed to yield the desired product. This was attributed to a decrease in the electrophilicity of the intermediate imidoyl fluoride, which hindered fluoride addition. Additionally, some imidoyl chlorides exhibited poor solubility in the reaction mixture, impairing their use in flow reactions. Alkyl-substituted electrophiles did not provide significant yields of the target product; instead, decomposition products predominated. Regarding the sulfonamide component, sulfamides and groups with strong electron-donating properties, as well as those that reduced the solubility of the imidoyl chloride, were not tolerated.

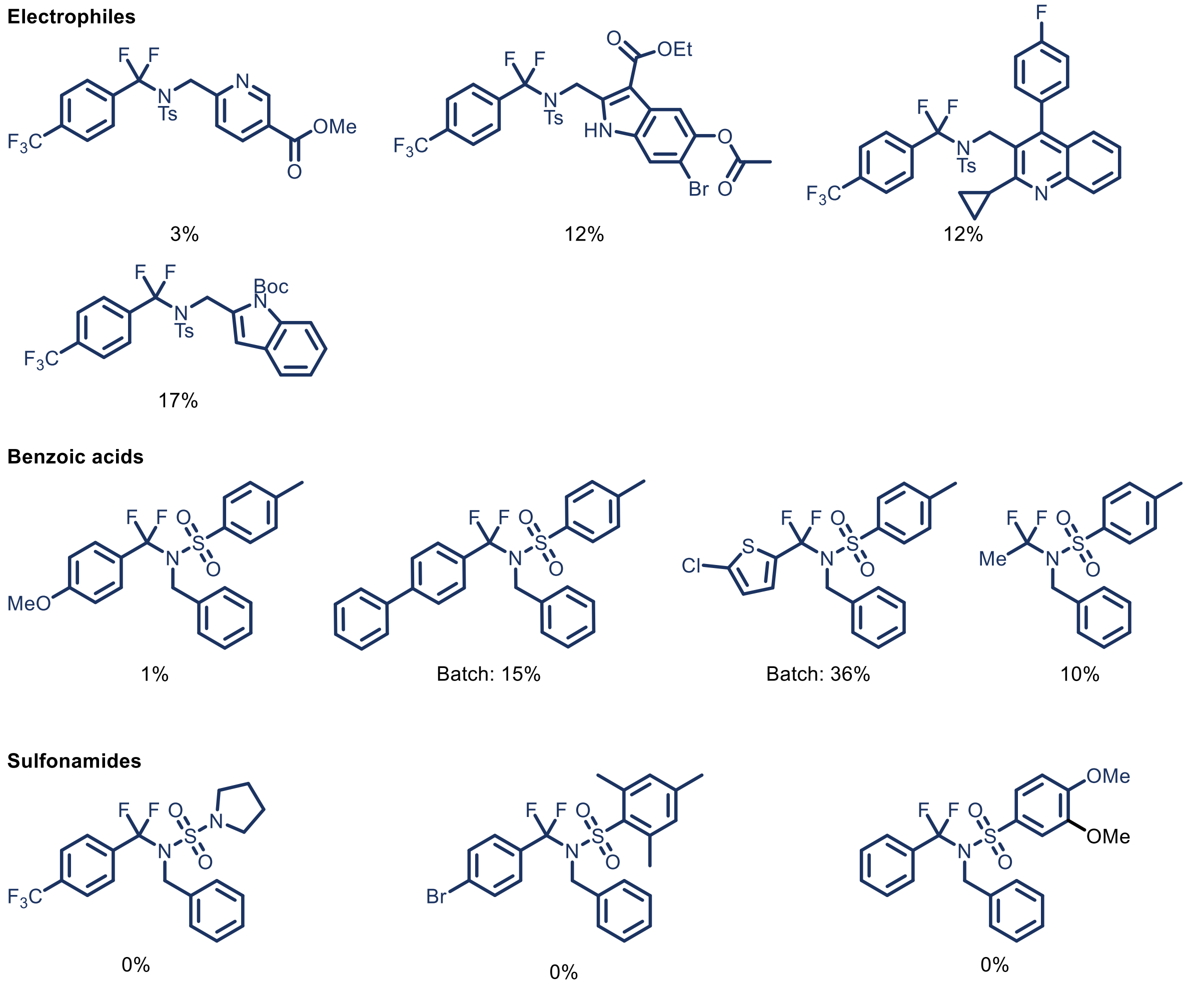

Figure S7: Limitations of the reaction scope.

3. Experimental Procedures

3.1 **General Procedure (A)**: Synthesis of Imidoyl Chlorides from Acyl Chlorides

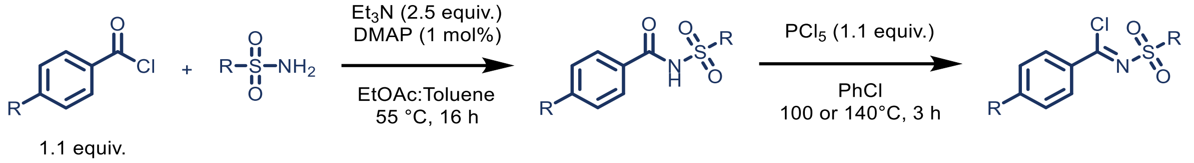

Figure S8. Reaction scheme to synthesize imidoyl chloride starting materials.

To a solution of sulfonamide (1.0 equiv.), 4-dimethylaminopyridine (1.0 mol%) and triethylamine (2.5 equiv.) in AcOEt (2.0 M) was dropwise added a solution of the respective benzoyl chloride (1.1 equiv.) in toluene (0.8 M). The mixture was stirred at 55 °C overnight, cooled to room temperature and quenched with a 2.0 M aqueous HCl solution. The resulting mixture was extracted with AcOEt, dried over $MgSO_4$, and concentrated in vacuo. The crude was washed with a minimal amount of diethyl ether and used in the next step without further purification.

The corresponding sulfonimide (1 equiv.) and PCl_5 (1.1 equiv.) were dissolved in chlorobenzene (0.6 M). The reaction mixture was heated to reflux for 3 h, after which the solvent was evaporated under reduced pressure at 60 °C. The crude was purified by the below-mentioned method.

3.2 **General Procedure (B)**: Synthesis of Imidoyl Chlorides from Carboxylic Acids

To a stirring solution of carboxylic acid (20 mmol, 1.0 equiv.) in dry CH_2Cl_2 (0.2 M), oxalyl chloride (1.2 equiv.) and few drops of dry DMF were added dropwise at 0 °C under N_2 atmosphere. Then, the reaction mixture was stirred overnight at room temperature. Upon completion, the solvent was removed under reduced pressure to afford the crude carbonyl chloride, which was immediately used in the next step. The synthesis sequence then follows **General Procedure A**.

3.3 **General procedure (C)**: Synthesis of NCF_2R Compounds from Different Electrophiles

The respective *N*-tosyl imidoyl chloride (3.5 mmol) was dissolved in an 18-crown-6 solution in dry CH_3CN (35 mL, 0.1 M) in an oven-dried, N_2 filled 100 mL round bottom flask with a rubber septum. The packed-bed reactor was flushed with dry CH_3CN (ca. 15 mL). The 0.1 M solution of *N*-tosyl imidoyl chloride was taken up with a 50 mL syringe and mounted on a syringe pump. Then, N-tosyl imidoyl chloride solution (10.0 mL) was passed through the reactor at a flowrate of 0.22 mL min-1 to equilibrate it. Once this procedure was done, the cartridge could be used continuously until its exhaustion. The solution of N-tosyl imidoyl chloride was constantly pushed through the equilibrated CsF/glass beads packed-bed at 0.22 mL min-1 (t_R = ca. 15 min). The resulting caesium $TsNCF_2R$ anion solution (6 mL, 0.6 mmol, 3 equiv.) was collected into an oven-dried, N_2 filled 20 mL reaction vial equipped with a Teflon stirring bar, containing tetrabutylammonium iodide (81.3 mg, 0.22 mmol, 1.1 equiv.) and the respective electrophile (if solid) (0.2 mmol, 1 equiv.). Upon collection of the desired amount of caesium $TsNCF_2R$ anion solution, electrophile (if liquid) (0.2 mmol, 1 equiv.) was added, and the reaction mixture was heated for 18 hours at 40 °C. After reaction completion, the solvent was evaporated *in vacuo* and the crude was purified using flash column chromatography (pentane/AcOEt).

3.4 **General procedure (D)**: Synthesis of NCF_2 Compounds from Different Acyl Chlorides/Sulfonamides

The respective imidoyl chloride (2.0 mmol) was dissolved in an 18-crown-6 solution in dry CH_3CN (20 mL, 0.1 M) in an oven-dried, N_2 filled 50 mL round bottom flask with a rubber septum. The packed-bed reactor was flushed with dry CH_3CN (ca. 15 mL). The 0.1 M solution of imidoyl chloride was taken up with a 20 mL syringe and mounted on a syringe pump. Then, imidoyl chloride solution (10.0 mL) was passed through the reactor at flowrate of 0.22 mL min-1 to equilibrate it. The solution of imidoyl chloride was constantly pushed through the equilibrated CsF/glass beads packed-bed at 0.22 mL min-1 (t_R = ca. 15 min). The resulting anion solution (8 mL, 0.8 mmol, 4 equiv.) was collected into an oven-dried, N_2 filled 20 mL reaction vial equipped with a Teflon stirring bar, containing tetrabutylammonium iodide (81.3 mg, 0.22 mmol, 1.1 equiv.). Upon collection of the desired amount of anion solution, cinnamyl bromide (39.4 mg, 29.6 µL, 0.2 mmol, 1 equiv.) was added, and the reaction mixture was heated for 18 hours at 60 °C. After reaction completion, the solvent was evaporated *in vacuo* and the crude was purified using flash column chromatography (pentane/AcOEt).

3.5 Synthesis of Benzyl Bromide Derivatives

Synthesis of Compound **SI-3**

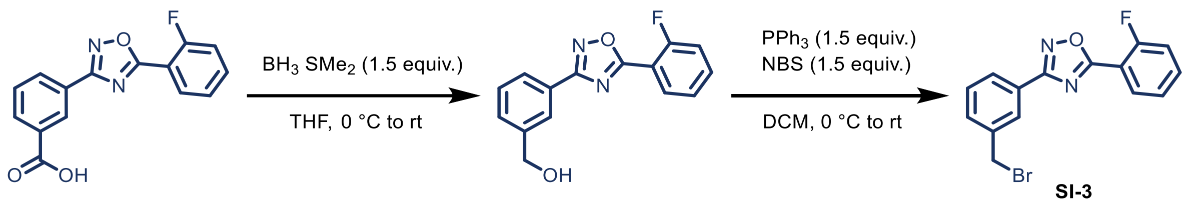

Figure S9. Reaction scheme for the synthesis of compound **SI-3**.

According to a previously reported literature procedure[3], ataluren (568 mg, 2.0 mmol) was added to an oven-dried, nitrogen-purged flask with a stir bar, followed by THF (0.1 M), and the mixture was cooled in an ice bath. $BH_3 \cdot SMe_2$ (1.5 equiv., 0.284 mL, 3.0 mmol) was added dropwise, and the reaction progress was monitored using TLC. Once complete, the reaction was quenched by slowly adding water while maintaining cooling. THF was removed using a rotary evaporator, and the crude product was extracted with ethyl acetate, dried over Na_2SO_4, and concentrated. The crude product was used in the next step without further purification.

The crude alcohol and PPh_3 (1.5 equiv., 787 mg, 3.0 mmol) were added to an oven-dried two-necked flask containing a stir bar under nitrogen. Dry DCM (0.1 M) was added, the flask was placed in an ice bath, and NBS (1.5 equiv., 534 mg, 3.0 mmol) was slowly added portion-wise. The ice bath was then removed, and the reaction completion was monitored using TLC. The reaction was concentrated under vacuum and the crude was purified using flash column chromatography (100% *n*-pentane to 5% AcOEt in *n*-pentane), affording compound **SI-3** (543 mg, 82%) as a white solid.

^1H NMR (300 MHz, $CDCl_3$) δ 8.24 – 8.18 (m, 2H), 8.13 (dt, *J* = 7.5, 1.6 Hz, 1H), 7.69 – 7.45 (m, 3H), 7.43 – 7.25 (m, 2H), 4.56 (s, 2H).

^{13}C NMR (75 MHz, $CDCl_3$) δ 172.9 (d, *J* = 4.6 Hz), 168.2, 160.8 (d, *J* = 260.8 Hz), 138.7, 134.7 (d, *J* = 8.4 Hz), 131.9, 130.9, 129.5, 128.1, 127.5, 127.3, 124.7 (d, *J* = 3.8 Hz), 117.2 (d, *J* = 20.7 Hz), 112.7 (d, *J* = 11.2 Hz), 32.7.

^{19}F NMR (282 MHz, $CDCl_3$) δ -108.8 (s, 1F).

HRMS (FD+) (m/z): $[M]^+$ calculated for $C_{15}H_{10}BrFN_2O$, 331.9961; found: 331.9968.

Synthesis of Compound **SI-5**

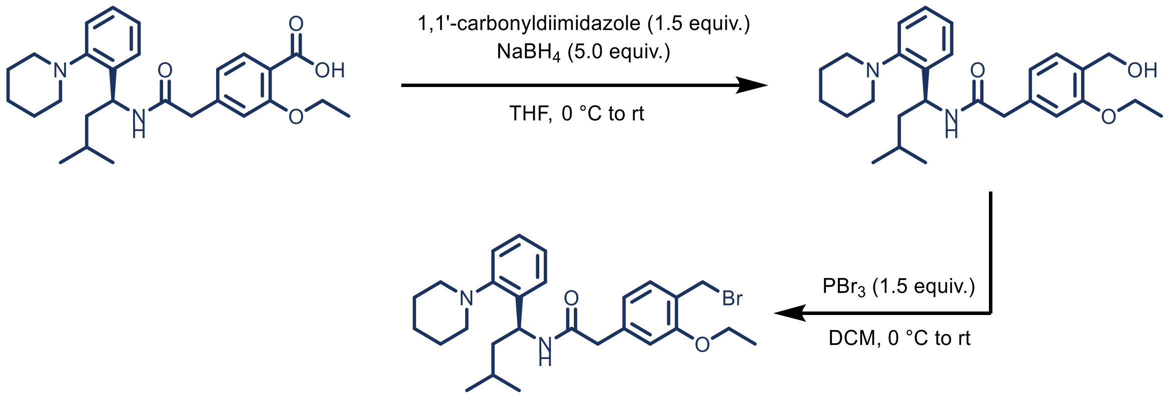

Figure S10. Reaction scheme for the synthesis of compound **SI-5**.

Repaglinide (453 mg, 1.0 mmol) was dissolved in THF (0.1 M), and 1,1'-carbonyldiimidazole (1.5 equiv., 243 mg, 1.5 mmol) was added in one portion. The solution was stirred at room temperature for 15 minutes, then cooled to 0°C for 10 minutes. $NaBH_4$ (5.0 equiv., 189 mg, 5.0 mmol) was added portion-wise and the mixture was stirred at 0°C for 25 minutes. The reaction was quenched with water, then extracted with ethyl acetate, washed with saturated $NaHCO_3$ and brine, dried over Na_2SO_4, and concentrated. The crude product is used in the next step without further purification.

The crude alcohol was added to an oven-dried two-necked flask containing a stir bar under nitrogen. DCM (1 M) was added, the flask was placed in an ice bath, and PBr_3 (1.5 equiv., 0.140 mL, 1.5 mmol) was slowly added dropwise. The ice bath was removed, and the reaction progress was monitored using TLC. The reaction was quenched by the slow addition of water while maintaining the mixture in an ice bath, then the crude product was extracted with ethyl acetate, dried over Na_2SO_4, and concentrated. The crude was purified using flash column chromatography (100% DCM to 1% MeOH in DCM), affording compound **SI-5** (358 mg, 72%) as a white solid.

^{1}H NMR (300 MHz, $CDCl_3$) δ 7.26 (d, J = 7.6 Hz, 1H), 7.23 – 7.15 (m, 2H), 7.10 – 7.01 (m, 2H), 6.81 – 6.68 (m, 3H), 5.47 – 5.24 (m, 1H), 4.55 (s, 2H), 4.16 – 3.84 (m, 2H), 3.51 (s, 2H), 2.92 (s, 2H), 2.59 (t, J = 8.4 Hz, 2H), 1.87 – 1.48 (m, 9H), 1.41 (t, J = 7.0 Hz, 4H), 0.91 (d, J = 6.5 Hz, 6H).

^{13}C NMR (75 MHz, $CDCl_3$) δ 169.4, 157.1, 152.6, 138.9, 137.7, 131.1, 127.9, 127.7, 125.1, 125.0, 122.7, 121.3, 112.6, 63.9, 55.0, 49.8, 46.6, 44.2, 28.9, 26.8, 25.4, 24.2, 22.9, 22.6, 4.8.

HRMS (FD+) (m/z): $[M]^+$ calculated for $C_{27}H_{37}BrN_2O_2$, 502.2022; found: 502.2047.

Synthesis of Compound **SI-6**

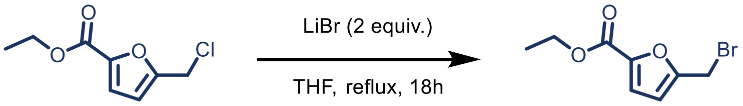

Figure S11. Reaction scheme for the synthesis of compound **SI-6**.

5-Chloromethyl-furan-2-carboxylic acid ethyl ester (943 mg, 5 mmol, 1 equiv.) was dissolved in THF (50 mL). LiBr (868 mg, 10 mmol, 2 equiv.) was added and the mixture was refluxed for 18 hours. The solvent was removed in vacuo and the crude was extracted with AcOEt and water, affording the product SI-6 (1.16 g, quantitative) as a slightly orange oil.

^{1}H NMR (300 MHz, $CDCl_3$) δ 7.09 (d, J = 3.5 Hz, 1H), 6.47 (d, J = 3.5 Hz, 1H), 4.47 (s, 2H), 4.34 (q, J = 7.1 Hz, 2H), 1.35 (t, J = 7.1 Hz, 3H).

^{13}C NMR (75 MHz, $CDCl_3$) δ 158.4, 154.2, 145.1, 118.8, 111.6, 61.2, 22.1, 14.4.

HRMS (FD+) (m/z): $[M]^+$ calculated for $C_8H_9BrO_3$, 231.9735; found: 231.9743.

4. Characterization data

4.1 Starting materials

N-Tosyl-4-(trifluoromethyl)benzimidoyl chloride (1)

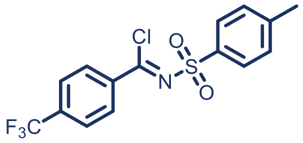

Prepared according to **General Procedure A**, from p-toluenesulfonamide (12.0 g, 70 mmol) and 4-(trifluoromethyl) benzoyl chloride (11.5 mL, 77 mmol). The crude mixture was purified either using column chromatography (100% *n*-pentane to *n*-pentane 90:10 AcOEt) (or via precipitation in *n*-pentane, giving a similar purity and yield), to afford compound **SI-5** (18.6 g, 73% over 2 steps) as a white solid.

^{1}H NMR (300 MHz, $CDCl_3$) δ 8.23 – 8.13 (m, 2H), 7.99 – 7.89 (m, 2H), 7.75 – 7.65 (m, 2H), 7.44 – 7.33 (m, 2H), 2.47 (s, 3H).

^{13}C NMR (75 MHz, $CDCl_3$) δ 154.9, 145.0, 137.3, 136.8, 135.8 (q, J = 33.1 Hz), 130.5, 129.9, 127.9, 125.8 (d, J = 3.7 Hz), 123.3 (d, J = 273.0 Hz), 21.8.

^{19}F NMR (282 MHz, $CDCl_3$) δ -63.32 (s, 3F).

HRMS (FD+) (m/z): $[M]^+$ calculated for $C_{15}H_{11}ClF_3NO_2S$, 361.0151; found: 361.0142.

4-Fluoro-*N*-tosylbenzimidoyl chloride (SI-7)

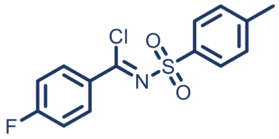

Prepared according to **General Procedure A**, from p-toluenesulfonamide (3.42 g, 20 mmol) and 4-fluorobenzoyl chloride (2.60 mL, 22 mmol). The crude mixture was purified using column chromatography (100% *n*-pentane to *n*-pentane 90:10 AcOEt), to afford compound **SI-7** (3.90 g, 69% over 2 steps) as a white solid.

^{1}H NMR (300 MHz, $CDCl_3$) δ 8.16 – 8.07 (m, 2H), 7.94 (d, J = 8.4 Hz, 2H), 7.38 (d, J = 7.9 Hz, 2H), 7.18 – 7.06 (m, 2H), 2.46 (s, 3H).

^{13}C NMR (75 MHz, $CDCl_3$) δ 166.8 (d, J = 258.6 Hz), 155.1, 144.7, 137.0, 132.9 (d, J = 9.8 Hz), 130.2 (d, J = 9.8 Hz), 129.7, 127.7, 116.1 (d, J = 22.3 Hz), 21.7.

^{19}F NMR (282 MHz, $CDCl_3$) δ -101.81 (s, 1F).

HRMS (FD+) (m/z): $[M]^+$ calculated for $C_{14}H_{11}C_{11}FNO_2S$, 311.0183; found: 311.0173.

4-Chloro-*N*-tosylbenzimidoyl chloride (SI-8)

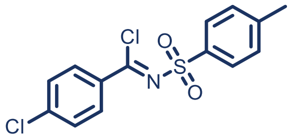

Prepared according to **General Procedure A**, from p-toluenesulfonamide (3.42 g, 20 mmol) and 4-chlorobenzoyl chloride (2.80 mL, 22 mmol). The crude mixture was dissolved in a minimal amount of DCM and added to a large excess of pentane, after which the mixture was briefly cooled down in liquid nitrogen, precipitating compound **SI-8** (4.08 g, 65% over 2 steps) as a white solid.

^{1}H NMR (300 MHz, $CDCl_3$) δ 8.04 – 7.98 (m, 2H), 7.93 (d, J = 8.3 Hz, 2H), 7.47 – 7.33 (m, 4H), 2.47 (s, 3H).

^{13}C NMR (75 MHz, $CDCl_3$) δ 155.4, 144.8, 141.7, 137.1, 132.6, 131.6, 129.8, 129.3, 127.9, 21.8.

HRMS (FD+) (m/z): $[M]^+$ calculated for $C_{14}H_{11}Cl_2NO_2S$, 326.9888, found: 326.9888.

4-bromo-2-fluoro-*N*-tosylbenzimidoyl chloride (SI-9)

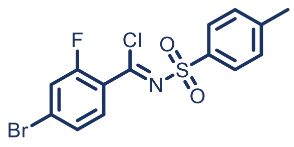

Prepared according to **General Procedure A**, from p-toluenesulfonamide (3.42 g, 20 mmol) and 4-bromo-2-fluorobenzoyl chloride (3.00 mL, 22 mmol). The crude mixture was purified using column chromatography (100% *n*-pentane to *n*-pentane 90:10 AcOEt), to afford compound **SI-9** (4.20 g, 59% over 2 steps) as a white solid.

^{1}H NMR (300 MHz, $CDCl_3$) δ 7.92 (d, J = 8.4 Hz, 2H), 7.80 – 7.69 (m, 1H), 7.44 – 7.29 (m, 4H), 2.45 (s, 3H).

^{13}C NMR (75 MHz, $CDCl_3$) δ 160.2 (d, J = 268.1 Hz), 150.6, 144.8, 136.9, 133.1, 129.8, 129.3 (d, J = 9.8 Hz), 128.0 (d, J = 3.8 Hz), 127.6, 122.6 (d, J = 7.9 Hz), 120.9 (d, J = 25.1 Hz), 21.7.

^{19}F NMR (282 MHz, $CDCl_3$) δ -105.16 (s, 1F).

HRMS (FD+) (m/z): $[M]^+$ calculated for $C_{14}H_{10}BrClFNO_2S$, 390.9266; found: 390.9293.

***N*-tosylbenzimidoyl chloride (SI-10)**

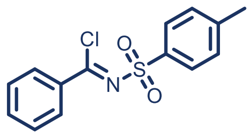

Prepared according to **General Procedure A**, from p-toluenesulfonamide (3.42 g, 20 mmol) and benzoyl chloride (2.55 mL, 22 mmol). The crude mixture was dissolved in a minimal amount of DCM and added to a large excess of pentane, after which the mixture was briefly cooled down in liquid nitrogen, precipitating compound **SI-10** (3.24 g, 58% over 2 steps) as a white solid.

^{1}H NMR (300 MHz, , $CDCl_3$) δ 8.13 – 7.98 (m, 2H), 7.94 (d, J = 8.4 Hz, 2H), 7.64 – 7.55 (m, 1H), 7.46 – 7.32 (m, 4H), 2.44 (s, 3H).

^{13}C NMR (75 MHz, $CDCl_3$) δ 156.6, 144.6, 137.1, 134.8, 134.0, 130.2, 129.7, 128.8, 127.7, 21.7.

HRMS (FD+) (m/z): $[M]^+$ calculated for $C_{14}H_{12}ClNO_2S$, 293.0277; found: 293.0274.

***N*-tosyl-4-(trifluoromethoxy)benzimidoyl chloride (SI-11)**

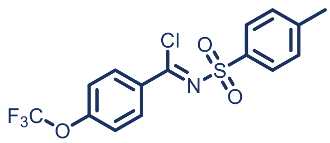

Prepared according to **General Procedure A**, from p-toluenesulfonamide (3.43 g, 20 mmol) and 4-(trifluoromethoxy)benzoyl chloride (3.47 mL, 22 mmol). The crude mixture was dissolved in a minimal amount of DCM and added to a large excess of pentane, after which the mixture was briefly cooled down in liquid nitrogen, precipitating compound **SI-11** (3.96 g, 52% over 2 steps) as a white solid.

^{1}H NMR (300 MHz, $CDCl_3$) δ 8.18 – 8.07 (m, 2H), 7.98 – 7.88 (m, 2H), 7.43 – 7.33 (m, 2H), 7.32 – 7.20 (m, 2H), 2.46 (s, 3H).

^{13}C NMR (75 MHz, $CDCl_3$) δ 154.8, 153.9 (q, J = 1.9 Hz), 144.8, 137.0, 132.3, 132.2, 129.8, 127.8, 120.3 (d, J = 1.1 Hz), 120.3 (q, J = 259.9 Hz), 21.8.

^{19}F NMR (282 MHz, $CDCl_3$) δ -57.61 (s, 3F).

HRMS (FD+) (m/z): $[M]^+$ calculated for $C_{15}H_{11}ClF_3NO_3S$, 377.0100; found: 377.0089.

4-cyano-*N*-tosylbenzimidoyl chloride (SI-12)

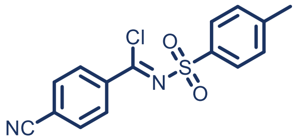

Prepared according to **General Procedure A**, from p-toluenesulfonamide (3.43 g, 20 mmol) and 4-cyanobenzoyl chloride (3.63 g, 22 mmol). The crude mixture was dissolved in a minimal amount of DCM and added to a large excess of pentane, after which the mixture was briefly cooled down in liquid nitrogen, precipitating compound **SI-12** (2.94 g, 46% over 2 steps) as a white solid.

^{1}H NMR (300 MHz, $CDCl_3$) δ 8.17 (d, *J* = 8.8 Hz, 2H), 7.93 (d, *J* = 8.4 Hz, 2H), 7.75 (d, *J* = 8.8 Hz, 1H), 7.40 (d, *J* = 8.4 Hz, 1H), 2.47 (s, 3H).

^{13}C NMR (75 MHz, $CDCl_3$) δ 154.2, 145.1, 137.7, 136.5, 132.5, 130.4, 129.9, 127.8, 117.6, 117.5, 21.7.

HRMS (FD+) (m/z): $[M]^+$ calculated for $C_{15}H_{11}ClN_2O_2S$, 318.0230; found: 318.0229.

Methyl -4-(chloro(tosylimino)methyl)benzoate (SI-13)

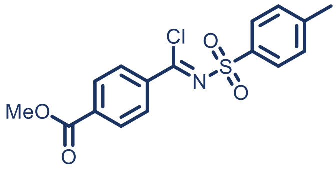

Prepared according to **General Procedure A**, from p-toluenesulfonamide (3.43 g, 20 mmol) and methyl 4-(chlorocarbonyl)benzoate (4.37 g, 22 mmol). The crude mixture was washed with Et_2O, obtaining compound **SI-13** (4.5 g, 64% over 2 steps) as a white solid.

^{1}H NMR (300 MHz, $CDCl_3$) δ 8.20 – 8.03 (m, 4H), 7.95 (d, J = 8.3 Hz, 2H), 7.39 (d, J = 8.1 Hz, 2H), 3.95 (s, 3H), 2.47 (s, 3H).

^{13}C NMR (75 MHz, $CDCl_3$) δ 165.7, 155.3, 144.8, 137.6, 136.8, 135.2, 130.0, 129.8, 129.7, 127.8, 52.6, 21.7.

HRMS (FD+) (m/z): $[M]^+$ calculated for $C_{16}H_{14}ClNO_4S$, 351.0332; found: 351.0323.

4-(methylsulfonyl)-*N*-tosylbenzimidoyl chloride (SI-14)

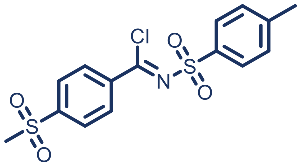

Prepared according to **General Procedure B**, from 4-methylsulfonylbenzoic acid (4.0 g, 20 mmol) and p-toluenesulfonamide (3.08 g, 18 mmol). The crude mixture was purified using column chromatography (100% *n*-pentane to *n*-pentane 80:20 AcOEt), to afford compound **SI-14** (4.68 g, 70% over 3 steps) as a white solid.

^{1}H NMR (300 MHz, $CDCl_3$) δ 8.25 (d, *J* = 8.6 Hz, 2H), 8.02 (d, *J* = 8.6 Hz, 2H), 7.94 (d, *J* = 8.3 Hz, 2H), 7.40 (d, *J* = 8.3 Hz, 2H), 3.07 (s, 3H), 2.48 (s, 3H).

^{13}C NMR (75 MHz, $CDCl_3$) δ 154.3, 145.4, 145.2, 138.7, 136.5, 130.9, 129.9, 127.9, 127.8, 44.3, 21.8.

HRMS (FD+) (m/z): $[M]^+$ calculated for $C_{15}H_{14}ClNO_4S_2$, 371.0052; found: 371.0057.

5-bromo-*N*-tosylthiophene-3-carbimidoyl chloride (SI-15)

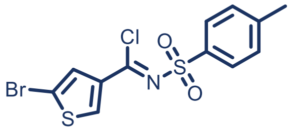

Prepared according to **General Procedure B**, from 5-bromothiophene-3-carboxylic acid (4.14 g, 20 mmol) and p-toluenesulfonamide (3.08 g, 18 mmol). The crude mixture was dissolved in a minimal amount of DCM and added to a large excess of pentane, after which the mixture was briefly cooled down in liquid nitrogen, precipitating compound **SI-15** (4.71 g, 69% over 3 steps) as a white solid.

^{1}H NMR (300 MHz, $CDCl_3$) δ 8.17 (d, *J* = 1.6 Hz, 1H), 7.95 – 7.85 (m, 2H), 7.46 (d, *J* = 1.6 Hz, 1H), 7.36 (d, *J* = 8.0 Hz, 2H), 2.46 (s, 3H).

^{13}C NMR (75 MHz, $CDCl_3$) δ 148.4, 144.8, 138.1, 137.4, 137.0, 129.8, 129.6, 127.8, 114.5, 21.8.

HRMS (FD+) (m/z): $[M]^+$ calculated for $C_{12}H_9BrClNO_2S_2$, 376.8946; found: 376.8958.

4-(*N*,*N*-dipropylsulfamoyl)-*N*-tosylbenzimidoyl chloride (SI-16)

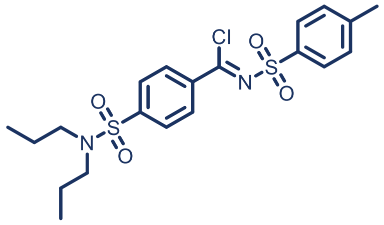

Prepared according to **General Procedure B**, from 4-(*N*,*N*-dipropylsulfamoyl)benzoic acid (6.28 g, 22 mmol) and p-toluenesulfonamide (3.22 g, 20 mmol). The crude mixture was purified using column chromatography (100% *n*-pentane to *n*-pentane 80:20 AcOEt), to afford compound **SI-16** (4.22 g, 46% over 3 steps) as a white solid.

^{1}H NMR (300 MHz, $CDCl_3$) δ 8.18 (d, *J* = 8.8 Hz, 2H), 7.92 (d, *J* = 8.2 Hz, 2H), 7.86 (d, *J* = 8.8 Hz, 2H), 7.38 (d, *J* = 8.2 Hz, 2H), 3.14 – 3.04 (m, 4H), 2.45 (s, 3H), 1.62 – 1.44 (m, 4H), 0.93 – 0.80 (m, 6H).

^{13}C NMR (75 MHz, $CDCl_3$) δ 154.6, 145.6, 145.0, 137.1, 136.6, 130.7, 129.8, 127.8, 127.2, 49.9, 21.9, 21.6, 11.1.

HRMS (FD+) (m/z): $[M]^+$ calculated for $C_{20}H_{25}ClN_2O_4S_2$, 456.0944; found: 456.0956.

***N*-((4-chlorophenyl)sulfonyl)-4-(trifluoromethyl)benzimidoyl chloride (SI-17)**

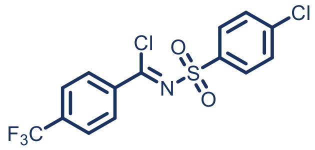

Prepared according to **General Procedure A**, from 4-chlorobenzenesulfonamide (3.83 g, 20 mmol) and 4-(trifluoromethyl)benzoyl chloride (3.27 mL, 22 mmol). The crude mixture was dissolved in a minimal amount of DCM and added to a large excess of pentane, after which the mixture was briefly cooled down in liquid nitrogen, precipitating compound **SI-17** (4.74 g, 62% over 2 steps) as a white solid.

^{1}H NMR (300 MHz, $CDCl_3$) δ 8.17 (d, *J* = 8.3 Hz, 2H), 8.03 – 7.95 (m, 2H), 7.71 (d, *J* = 8.3 Hz, 2H), 7.60 – 7.53 (m, 2H).

^{13}C NMR (75 MHz, $CDCl_3$) δ 155.9, 140.6, 138.2, 137.0 (q, *J* = 1.3 Hz), 136.0 (q, *J* = 33.1 Hz), 130.6, 129.6, 129.3, 125.9 (q, *J* = 3.7 Hz), 123.3 (q, *J* = 273.0 Hz).

^{19}F NMR (282 MHz, $CDCl_3$) δ -63.33 (s, 3F).

HRMS (FD+) (m/z): $[M]^+$ calculated for $C_{14}H_8Cl_2F_3NO_2S$, 380.9605; found: 380.9610.

***N*-((4-fluorophenyl)sulfonyl)-4-(trifluoromethyl)benzimidoyl chloride (SI-18)**

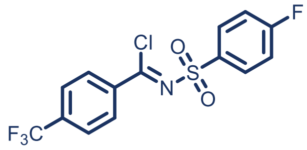

Prepared according to **General Procedure A**, from 4-fluorobenzenesulfonamide (3.50 g, 20 mmol) and 4-(trifluoromethyl)benzoyl chloride (3.27 mL, 22 mmol). The crude mixture was dissolved in a minimal amount of DCM and added to a large excess of pentane, after which the mixture was briefly cooled down in liquid nitrogen, precipitating compound **SI-18** (3.64 g, 58% over 2 steps) as a white solid.

^{1}H NMR (300 MHz, $CDCl_3$) δ 8.17 (d, *J* = 8.3 Hz, 2H), 8.12 – 8.02 (m, 2H), 7.70 (d, *J* = 8.3 Hz, 2H), 7.30 – 7.21 (m, 2H).

^{13}C NMR (75 MHz, $CDCl_3$) δ 165.8 (d, J = 256.6 Hz), 155.6, 137.1 (q, J = 1.3 Hz), 135.9 (q, J = 33.1 Hz), 135.8 (d, J = 3.3 Hz), 130.7 (d, J = 9.6 Hz), 130.5, 125.9 (q, J = 3.7 Hz), 123.3 (q, J = 273.0 Hz), 116.6 (d, J = 22.8 Hz).

^{19}F NMR (282 MHz, $CDCl_3$) δ -63.35 (s, 3F), -103.04 (s, 1F).

HRMS (FD+) (m/z): $[M]^+$ calculated for $C_{14}H_8ClF_4NO_2S$, 364.9900; found: 364.9886.

***N*-((4-methoxyphenyl)sulfonyl)-4-(trifluoromethyl)benzimidoyl chloride (SI-19)**

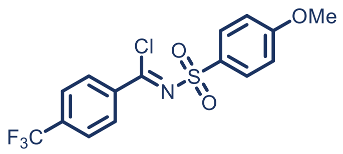

Prepared according to **General Procedure A**, from 4-methoxybenzenesulfonamide (3.74 g, 20 mmol) and 4-(trifluoromethyl)benzoyl chloride (3.27 mL, 22 mmol). The crude mixture was purified using column chromatography (100% *n*-pentane to *n*-pentane 90:10 AcOEt), to afford compound **SI-19** (3.80 g, 50% over 2 steps) as a white solid.

^{1}H NMR (300 MHz, $CDCl_3$) δ 8.16 (d, J = 8.3 Hz, 2H), 8.01 – 7.93 (m, 2H), 7.67 (d, J = 8.3 Hz, 2H), 7.06 – 7.00 (m, 2H), 3.88 (s, 3H).

^{13}C NMR (75 MHz, $CDCl_3$) δ 164.0, 154.4, 137.3 (d, J = 1.3 Hz), 135.6 (q, J = 33.0 Hz), 131.0, 130.5, 130.2, 125.8 (q, J = 3.7 Hz), 123.3 (q, J = 273.0 Hz), 114.5, 55.8.

^{19}F NMR (282 MHz, $CDCl_3$) δ -63.30 (s, 3F).

HRMS (FD+) (m/z): $[M]^+$ calculated for $C_{15}H_{11}ClF_3NO_3S$, 377.0100; found 377.0089.

***N*-((4-nitrophenyl)sulfonyl)-4-(trifluoromethyl)benzimidoyl chloride (SI-20)**

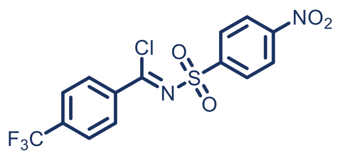

Prepared according to **General Procedure A**, from 4-(hydroxyamino)benzenesulfonamide (4.04 g, 20 mmol) and 4-(trifluoromethyl)benzoyl chloride (3.27 mL, 22 mmol). The crude mixture was purified using column chromatography (100% *n*-pentane to *n*-pentane 90:10 AcOEt), to afford compound **SI-20** (6.4 g, 81% over 2 steps) as a white solid.

^{1}H NMR (300 MHz, $CDCl_3$) δ 8.49 – 8.41 (m, 2H), 8.30 – 8.22 (m, 2H), 8.18 (d, J = 8.4 Hz, 2H), 7.74 (d, J = 8.4 Hz, 2H).

^{13}C NMR (75 MHz, $CDCl_3$) δ 157.4, 150.8, 145.3, 137.0, 136.7 (d, J = 1.37 Hz), 136.4 (q, J = 33.2 Hz), 130.7, 129.1, 126.1 (q, J = 3.7 Hz), 123.2 (q, J = 273.2 Hz).

^{19}F NMR (282 MHz, $CDCl_3$) δ -63.39 (s, 3F).

HRMS (FD+) (m/z): $[M]^+$ calculated for $C_{14}H_8ClF_3N_2O_4S$, 391.9845; found 391.9846.

***N*-((1,4-dimethyl-1H-pyrazol-5-yl)sulfonyl)-4-(trifluoromethyl)benzimidoyl chloride (SI-21)**

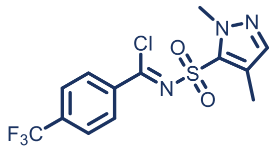

Prepared according to **General Procedure A**, from 1,4-dimethyl-1H-pyrazole-5-sulfonamide (3.50 g, 20 mmol) and 4-(trifluoromethyl)benzoyl chloride (3.27 mL, 22 mmol). The crude mixture was purified using column chromatography (100% *n*-pentane to *n*-pentane 90:10 AcOEt), to afford compound **SI-21** (3,01 g, 41% over 2 steps) as a white solid.

^{1}H NMR (300 MHz, $CDCl_3$) δ 8.20 (d, J = 8.3 Hz, 2H), 7.93 (s, 1H), 7.72 (d, J = 8.3 Hz, 2H), 3.90 (s, 3H), 2.46 (s, 3H).

^{13}C NMR (75 MHz, $CDCl_3$) δ 154.1, 148.7, 137.2 (d, J = 1.4 Hz), 135.6 (q, J = 33.0 Hz), 134.4, 130.3, 125.8 (q, J = 3.8 Hz), 123.3 (q, J = 273.0 Hz), 119.3, 39.3, 12.5.

^{19}F NMR (282 MHz, $CDCl_3$) δ -63.33 (s, 3F).

HRMS (FD+) (m/z): $[M]^+$ calculated for $C_{13}H_{11}ClF_3N_3O_2S$, 365.0213; found 365.0209.

N-(thiophen-2-ylsulfonyl)-4-(trifluoromethyl)benzimidoyl chloride (SI-22)

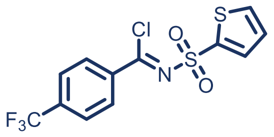

Prepared according to **General Procedure A**, from 4-(trifluoromethyl)benzoyl chloride (3.27 mL, 22 mmol) and 2-thiophenesulfonamide (3.26 g, 20 mmol). The crude mixture was purified using column chromatography (100% *n*-pentane to *n*-pentane 80:20 AcOEt), to afford compound **SI-22** (3.46 g, 49% over 2 steps) as a slightly orange solid.

^1H NMR (300 MHz, $CDCl_3$) δ 8.21 (d, J = 8.4 Hz, 2H), 7.86 (dd, J = 3.8, 1.4 Hz, 1H), 7.76 (dd, J = 5.0, 1.4 Hz, 1H), 7.71 (d, J = 8.4 Hz, 2H), 7.16 (dd, J = 5.0, 3.8 Hz, 1H).

^{13}C NMR (75 MHz, $CDCl_3$) δ 155.4, 140.0, 137.0, 135.9 (q, J = 33.1 Hz), 134.2, 134.2, 130.6, 127.6, 125.9 (q, J = 3.7 Hz), 123.3 (q, J = 273.0 Hz).

^{19}F NMR (282 MHz, $CDCl_3$) δ -63.34 (s, 3F).

HRMS (FD+) (m/z): $[M]^+$ calculated for $C_{12}H_7ClF_3NO_2S_2$, 352.9558; found: 352.9573.

N-((5-chlorothiophen-2-yl)sulfonyl)-4-(trifluoromethyl)benzimidoyl chloride (SI-23)

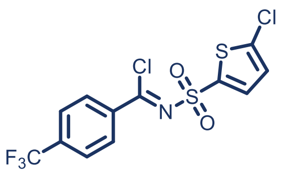

Prepared according to **General Procedure A**, from 4-(trifluoromethyl)benzoyl chloride (3.27 mL, 22 mmol) and 5-chloro-2-thiophenesulfonamide (3.95 g, 20 mmol). The crude mixture was purified using column chromatography (100% *n*-pentane to *n*-pentane 80:20 AcOEt), to afford compound **SI-23** (4.02 g, 55% over 2 steps) as a white solid.

^1H NMR (300 MHz, $CDCl_3$) δ 8.22 (d, J = 8.1 Hz, 2H), 7.74 (d, J = 8.1 Hz, 2H), 7.65 (d, J = 4.0 Hz, 1H), 7.00 (d, J = 4.0 Hz, 1H).

^{13}C NMR (75 MHz, $CDCl_3$) δ 156.1, 139.9, 137.8, 136.9, 136.2 (q, J = 33.3 Hz), 133.7, 130.7, 127.0, 126.0 (q, J = 3.7 Hz), 123.3 (q, J = 273.0 Hz).

^{19}F NMR (282 MHz, $CDCl_3$) δ -63.34.

HRMS (FD+) (m/z): $[M]^+$ calculated for $C_{12}H_6Cl_2F_3NO_2S_2$, 386.9169; found 386.9176.

N-(methylsulfonyl)-4-(trifluoromethyl)benzimidoyl chloride (SI-24)

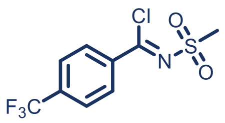

Prepared according to **General Procedure A**, from 4-(trifluoromethyl)benzoyl chloride (3.27 mL, 22 mmol) and methanesulfonamide (1,90 g, 20 mmol). The crude mixture was dissolved in a minimal amount of DCM and added to a large excess of pentane, after which the mixture was briefly cooled down in liquid nitrogen, precipitating compound **SI-24** (4,46 g, 78% over 2 steps) as a white solid.

^1H NMR (300 MHz, $CDCl_3$) δ 8.23 (d, J = 8.2 Hz, 2H), 7.76 (d, J = 8.2 Hz, 2H), 3.32 (s, 3H).

^{13}C NMR (75 MHz, $CDCl_3$) δ 155.4, 136.9, 135.8 (q, J = 33.2 Hz), 130.3, 125.9 (q, J = 3.8 Hz), 123.3 (q, J = 273.0 Hz), 43.1.

^{19}F NMR (282 MHz, $CDCl_3$) δ -63.32.

HRMS (FD+) (m/z): $[M]^+$ calculated for $C_9H_7ClF_3NO_2S$, 284.9838; found 284.9830.

4-(trifluoromethyl)-*N*-((trifluoromethyl)sulfonyl)benzimidoyl chloride (SI-25)

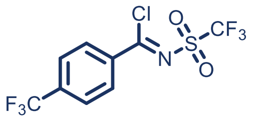

Prepared according to **General Procedure A**, from 4-(trifluoromethyl)benzoyl chloride (3.27 mL, 22 mmol) and trifluoromethanesulfonamide (2,98 g, 20 mmol). The crude mixture was dissolved in a minimal amount of DCM and added to a large excess of pentane, after which the mixture was briefly cooled down in liquid nitrogen, precipitating compound **SI-25** (5,13 g, 75.5% over 2 steps) as a white solid.

^{1}H NMR (300 MHz, $CDCl_3$) δ 8.31 (d, *J* = 8.1 Hz, 1H), 7.84 (d, *J* = 8.7 Hz, 1H).

^{13}C NMR (75 MHz, $CDCl_3$) δ 162.5, 137.3 (q, *J* = 33.5 Hz), 135.5 (d, *J* = 1.4 Hz), 131.0, 126.2 (q, *J* = 3.7 Hz), 123.0 (q, *J* = 273.2 Hz), 118.5 (q, *J* = 319.3 Hz).

^{19}F NMR (282 MHz, $CDCl_3$) δ -63.66 (s, 3F), -78.52 (s, 3F).

HRMS (FD+) (m/z): $[M]^+$ calculated for $C_9H_4ClF_6NO_2S$, 338.9555; found 338.9544.

***N*-((4-(5-methyl-3-phenylisoxazol-4-yl)phenyl)sulfonyl)-4-(trifluoromethyl)benzimidoyl chloride (SI-26)**

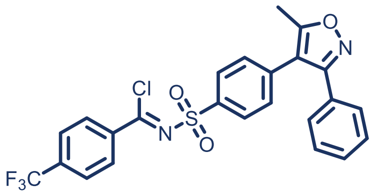

Prepared according to **General Procedure A**, from 4-(trifluoromethyl)benzoyl chloride (1.65 mL, 11 mmol) and 4-(5-methyl-3-phenylisoxazol-4-yl)benzenesulfonamide (3.14 g, 10 mmol). The crude mixture was dissolved in a minimal amount of DCM and added to a large excess of pentane, precipitating compound **SI-26** (2.01 g, 40% over 2 steps) as a white solid.

^{1}H NMR (300 MHz, $CDCl_3$) δ 8.20 (d, *J* = 8.2 Hz, 2H), 8.04 (d, *J* = 8.4 Hz, 2H), 7.72 (d, *J* = 8.4 Hz, 2H), 7.44 – 7.34 (m, 7H), 2.51 (s, 3H).

^{13}C NMR (75 MHz, $CDCl_3$) δ 167.6, 161.1, 155.8, 138.7, 137.1 (q, *J* = 1.4 Hz), 136.5, 135.9 (q, *J* = 32.9 Hz), 130.6, 130.4, 129.9, 128.8, 128.6, 128.4, 128.1, 125.9 (q, *J* = 3.7 Hz), 124.7 (q, *J* = 273.0 Hz), 114.4, 11.9.

^{19}F NMR (282 MHz, $CDCl_3$) δ -63.28 (s, 3F).

HRMS (FD+) (m/z): $[M]^+$ calculated for $C_{24}H_{16}ClF_3N_2O_3S$, 504.0522; found 504.0524.

***N*-((4-(3-(difluoromethyl)-5-(3-fluoro-4-methoxyphenyl)-1H-pyrazol-1-yl)phenyl)sulfonyl)-4-(trifluoromethyl)benzimidoyl chloride (SI-27)**

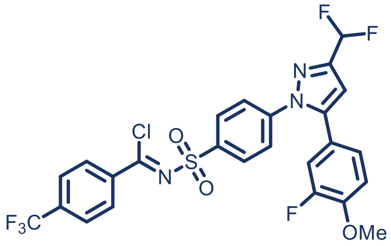

Prepared according to **General Procedure A**, from 4-(trifluoromethyl)benzoyl chloride (1.65 mL, 11 mmol) and 4-[5-(4-methylphenyl)-3-trifluoromethyl)-1H-pyrazolyl]benzenesulfonamide (3.97 g, 10 mmol). The crude was purified using flash column chromatography (100% *n*-pentane to 10% AcOEt in *n*-pentane), affording compound **SI-27** (2.75 g, 43% over 2 steps) as an off-white solid.

^{1}H NMR (300 MHz, $CDCl_3$) δ 8.17 (d, *J* = 8.4 Hz, 2H), 8.09 – 8.01 (m, 2H), 7.72 (d, *J* = 8.4 Hz, 2H), 7.59 – 7.49 (m, 2H), 7.04 – 6.95 (m, 3H), 6.75 (t, *J* = 54.7 Hz, 1H), 6.71 (s, 1H), 3.91 (s, 3H).

^{13}C NMR (300 MHz, $CDCl_3$) δ 156.0, 152.2 (d, J = 248.3 Hz), 148.8 (d, J = 10.5 Hz), 148.6 (t, J = 30.0 Hz), 143.9, 143.5, 138.9, 137.0 (q, J = 1.3 Hz), 136.1 (q, J = 33.1 Hz), 130.6, 128.9, 126.0 (q, J = 3.7 Hz), 125.4, 125.3, 123.3 (q, J = 273.0 Hz), 121.7 (d, J = 7.1 Hz), 116.7 (d, J = 19.8 Hz), 113.7 (d, J = 2.4 Hz), 111.0 (t, J = 234.7 Hz), 106.1, 56.3.

^{19}F NMR (282 MHz, $CDCl_3$) δ -63.34 (s, 3F), -112.41 (s, 2F), -133.23 (s, 1F).

HRMS (FD+) (m/z): $[M]^+$ calculated for $C_{25}H_{16}ClF_6N_3O_3S$, 587.0505; found 587.0509.

_N_-((5-bromothiophen-2-yl)sulfonyl)-2,4-dichlorobenzimidoyl chloride (SI-28)

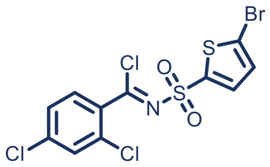

Prepared according to **General Procedure B**, from 2,4-dichlorobenzoic acid (4.04 g, 21.2 mmol) and 5-bromo-2-thiophenesulfonamide (4.5 g, 18 mmol). The crude was purified using flash column chromatography (100% *n*-pentane to 10% AcOEt in *n*-pentane), affording compound **SI-28** (4.65 g, 60% over 2 steps) as white solid.

^{1}H NMR (300 MHz, $CDCl_3$) δ 7.70 – 7.58 (m, 1H), 7.54 (d, J = 4.0 Hz, 1H), 7.38 (d, J = 2.0 Hz, 1H), 7.30 (dd, J = 8.5, 2.0 Hz, 1H), 7.07 (d, J = 4.0 Hz, 1H).

^{13}C NMR (75 MHz, $CDCl_3$) δ 153.1, 140.0, 139.0, 134.2, 133.2, 132.4, 132.1, 130.7, 130.4, 127.3, 122.4.

HRMS (FD+) (m/z): $[M]^+$ calculated for $C_{11}H_5BrC3NO_2S_2$, 430.8011; found 430.8001.

4-bromo-5-chloro-2-fluoro-_N_-(methylsulfonyl)benzimidoyl chloride (47)

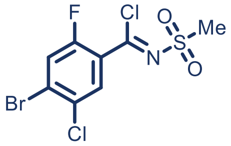

Prepared according to **General Procedure A**, from methanesulfonamide (1,90 g, 20 mmol) and 4-bromo-2-chloro-5-fluorobenzoyl chloride (5.71 g, 21 mmol). The crude mixture was dissolved in a minimal amount of DCM and added to a large excess of pentane, after which the mixture was briefly cooled down in liquid nitrogen, precipitating compound **47** (3.70 g, 53% over 2 steps) as a white solid.

^{1}H NMR (300 MHz, $CDCl_3$) δ 7.97 (d, J = 6.9 Hz, 1H), 7.52 (d, J = 9.8 Hz, 1H), 3.27 (s, 3H).

^{13}C NMR (75 MHz, $CDCl_3$) δ 158.2 (d, J = 267.6 Hz), 150.0, 132.5, 131.0 (d, J = 4.0 Hz), 129.6 (d, J = 9.9 Hz), 123.4 (d, J = 9.1 Hz), 123.0 (d, J = 26.2 Hz), 43.3.

^{19}F NMR (282 MHz, $CDCl_3$) δ -108.67 (s, 1F).

_N_-((2-chlorophenyl)sulfonyl)benzimidoyl chloride (50)

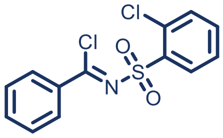

Prepared according to **General Procedure A**, from 2-chlorobenzenesulfonamide (5.0 g, 26 mmol) and benzoyl chloride (4.0 g, 29 mmol). The crude mixture was dissolved in a minimal amount of DCM and added to a large excess of pentane, after which the mixture was briefly cooled down in liquid nitrogen, precipitating compound **50** (5.37 g, 66% over 2 steps) as a white solid.

^{1}H NMR (300 MHz, $CDCl_3$) δ 8.22 (dt, J = 7.8, 1.0 Hz, 1H), 8.18 – 8.06 (m, 2H), 7.67 – 7.54 (m, 3H), 7.51 – 7.40 (m, 3H).

^{13}C NMR (75 MHz, $CDCl_3$) δ 157.5, 137.9, 135.1, 134.5, 133.9, 133.0, 132.0, 130.4, 130.4, 129.0, 127.1.

HRMS (FD+) (m/z): $[M]^+$ calculated for $C_{13}H_9Cl_2ClNO_2S$, 312.9731; found: 312.9730.

4-bromo-N-tosylbenzimidoyl chloride (53)

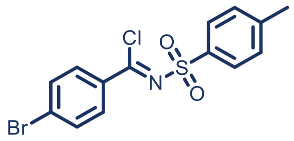

Prepared according to **General Procedure A**, from p-toluenesulfonamide (3.42 g, 20 mmol) and 4-bromobenzoyl chloride (4.83 g, 22 mmol). The crude mixture was dissolved in a minimal amount of DCM and added to a large excess of pentane, after which the mixture was briefly cooled down in liquid nitrogen, precipitating compound **53** (5.44 g, 73% over 2 steps) as a white solid.

^{1}H NMR (300 MHz, $CDCl_3$) δ 7.98 – 7.85 (m, 4H), 7.62 – 7.52 (m, 2H), 7.42 – 7.32 (m, 2H), 2.46 (s, 3H).

^{13}C NMR (75 MHz, $CDCl_3$) δ 155.4, 144.8, 137.0, 133.0, 132.2, 131.5, 130.4, 129.8, 127.8, 21.8.

HRMS (FD+) (m/z): $[M]^+$ calculated for $C_{14}H_{11}BrClNO_2S$, 370.9382; found: 370.9369.

4.2Scope of Electrophiles

***N*-benzyl-*N*-(difluoro(4-(trifluoromethyl)phenyl)methyl)-4-methylbenzenesulfonamide (2)**

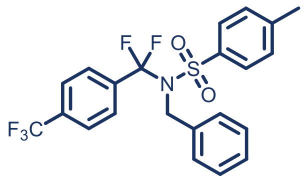

Prepared according to **General Procedure C**, using benzyl bromide (34.2 mg, 23.8 µL, 0.2 mmol, 1 equiv.), tetrabutylammonium iodide (81.3 mg, 0.22 mmol, 1.1 equiv.) and *N*-tosyl-4-(trifluoromethyl)benzimidoyl chloride solution (6 mL, 0.1 M, 0.6 mmol, 3 equiv.). The crude was purified using flash column chromatography (100% *n*-pentane to 10% AcOEt in *n*-pentane), affording compound **2** (65 mg, 71%) as a clear oil.

^{1}H NMR (300 MHz, $CDCl_3$) δ 7.49 (s, 4H), 7.42 – 7.28 (m, 7H), 7.16 (d, J = 8.0 Hz, 2H), 4.74 (s, 2H), 2.40 (s, 3H).

^{13}C NMR (75 MHz, $CDCl_3$) δ 144.3, 137.4 (t, J = 30.0 Hz), 137.0, 136.7, 132.7 (q, J = 32.8f Hz), 129.5, 128.5, 128.4, 127.9, 127.6 (t, J = 4.7 Hz), 124.9 (q, J = 3.8 Hz), 123.0 (t, J = 272.4 Hz), 122.7 (q, J = 256.9 Hz), 49.6 (t, J = 2.0 Hz), 21.5.

^{19}F NMR (282 MHz, $CDCl_3$) δ -63.08 (s, 3F), -69.80 (s, 2F).

HRMS (FD+) (m/z): $[M]^+$ calculated for $C_{22}H_{18}F_5NO_2S$, 455.0978; found: 455.0969.

***N*-(difluoro(4-(trifluoromethyl)phenyl)methyl)-*N*-(4-fluorobenzyl)-4-methylbenzenesulfonamide (3)**

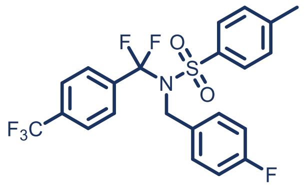

Prepared according to **General Procedure C**, using 4-fluorobenzyl bromide (37.8 mg, 24.9 µL, 0.2 mmol, 1 equiv.), tetrabutylammonium iodide (81.3 mg, 0.22 mmol, 1.1 equiv.) and *N*-tosyl-4-(trifluoromethyl)benzimidoyl chloride solution (6 mL, 0.1 M, 0.6 mmol, 3 equiv.). The crude was purified using flash column chromatography (100% *n*-pentane to 10% AcOEt in *n*-pentane), affording compound **3** (70 mg, 74%) as an off-white solid.

^{1}H NMR (300 MHz, $CDCl_3$) δ 7.57 – 7.43 (m, 4H), 7.42 – 7.29 (m, 4H), 7.21 – 7.11 (m, 2H), 7.08 – 6.95 (m, 2H), 4.71 (s, 2H), 2.40 (s, 3H).

^{13}C NMR (75 MHz, $CDCl_3$) δ 162.5 (d, J = 246.7 Hz), 144.5, 137.3 (t, J = 31.3 Hz), 137.0, 132.8 (q, J = 32.7 Hz), 132.6 (d, J = 3.3 Hz), 130.3 (d, J = 8.2 Hz), 129.6, 127.7 (t, J = 4.8 Hz), 127.6, 125.0 (q, J = 3.7 Hz), 123.6 (q, J = 272.8 Hz), 119.9 (t, J = 255.7 Hz), 115.5 (d, J = 21.5 Hz), 48.9, 21.6.

^{19}F NMR (282 MHz, $CDCl_3$) δ -63.09 (s, 3F), -69.79 (s, 2F), -114.24 (s, 1F).

HRMS (FD+) (m/z): $[M]^+$ calculated for $C_{22}H_{17}F_6NO_2S$, 473.0884; found: 473.0884.

***N*-(4-chlorobenzyl)-*N*-(difluoro(4-(trifluoromethyl)phenyl)methyl)-4-methylbenzenesulfonamide (4)**

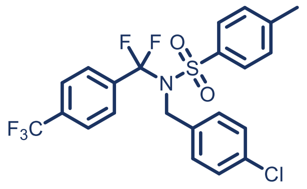

Prepared according to **General Procedure C**, using 4-chlorobenzyl bromide (41.1 mg, 0.2 mmol, 1 equiv.), tetrabutylammonium iodide (81.3 mg, 0.22 mmol, 1.1 equiv.) and *N*-tosyl-4-(trifluoromethyl)benzimidoyl chloride solution (6 mL, 0.1 M, 0.6 mmol, 3 equiv.). The crude was purified using flash column chromatography (100% *n*-pentane to 10% AcOEt in *n*-pentane), affording compound **4** (75.4 mg, 77%) as a white solid.

^{1}H NMR (300 MHz, $CDCl_3$) δ 7.56 – 7.43 (m, 4H), 7.39 – 7.27 (m, 6H), 7.21 – 7.13 (m, 2H), 4.70 (t, *J* = 1.8 Hz, 2H), 2.41 (s, 3H).

^{13}C NMR (75 MHz, $CDCl_3$) δ 144.5, 137.2 (t, *J* = 31.0 Hz) 136.9, 135.4, 133.9, 132.9 (q, *J* = 32.8 Hz), 129.9, 129.6, 128.8, 127.6 (t, *J* = 4.8 Hz), 127.6, 125.1 (q, *J* = 3.7 Hz), 123.6 (q, *J* = 272.6 Hz), 119.9 (t, *J* = 255.6 Hz), 48.9, 21.6.

^{19}F NMR (282 MHz, $CDCl_3$) δ -63.08 (s, 3F), -69.83 (s, 2F).

HRMS (FD+) (m/z): $[M]^+$ calculated for $C_{22}H_{17}ClF_5NO_2S$, 489.0589; found: 489.0589.

_N_-(4-bromobenzyl)-_N_-(difluoro(4-(trifluoromethyl)phenyl)methyl)-4-methylbenzenesulfonamide (5)

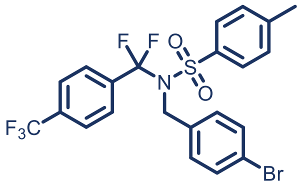

Prepared according to a modified version of **General Procedure C**, using 4-bromobenzyl bromide (666.4 mg, 2.66 mmol, 1 equiv.), tetrabutylammonium iodide (1.08 g, 2.93 mmol, 1.1 equiv.) and *N*-tosyl-4-(trifluoromethyl)benzimidoyl chloride solution (80 mL, 0.1 M, 8 mmol, 3 equiv.). The solution was charged into 2 syringes that were mounted on the syringe pump and each solution was passed through a separate CsF cartridge, and the outflow was collected in the receiving flask containing TBAI. The electrophile was added to the mixture after all the outflow was collected. The reaction mixture was heated at 40°C for 18 hours. The crude was purified using flash column chromatography (100% *n*-pentane to 10% AcOEt in *n*-pentane), affording compound **5** (1.00 g, 70%) as a white solid.

^{1}H NMR (300 MHz, $CDCl_3$) δ 7.56 – 7.41 (m, 6H), 7.34 (d, *J* = 8.3 Hz, 2H), 7.30 – 7.22 (m, 2H), 7.21 – 7.12 (m, 2H), 4.68 (s, 2H), 2.41 (s, 3H).

^{13}C NMR (75 MHz, $CDCl_3$) δ 144.5, 137.2 (t, *J* = 31.0 Hz), 136.9, 135.9, 132.9 (q, *J* = 32.8 Hz), 131.7, 130.2, 129.6, 127.6 (t, *J* = 4.8 Hz), 127.6, 125.1 (q, *J* = 3.7 Hz), 123.6 (q, *J* = 273.5 Hz), 122.0, 119.9 (t, *J* = 255.8 Hz), 49.0, 21.6.

^{19}F NMR (282 MHz, $CDCl_3$) δ -63.09 (s, 3F), -69.83 (s, 2F).

HRMS (FD+) (m/z): $[M]^+$ calculated for $C_{22}H_{17}BrF_5NO_2S$, 535.0065; found: 535.0180.

_N_-(difluoro(4-(trifluoromethyl)phenyl)methyl)-_N_-(4-iodobenzyl)-4-methylbenzenesulfonamide (6)

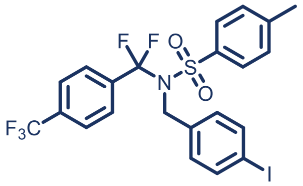

Prepared according to **General Procedure C**, using 4-iodobenzyl bromide (59.4 mg, 0.2 mmol, 1 equiv.), tetrabutylammonium iodide (81.3 mg, 0.22 mmol, 1.1 equiv.) and *N*-tosyl-4-(trifluoromethyl)benzimidoyl chloride solution (6 mL, 0.1 M, 0.6 mmol, 3 equiv.). The crude was purified using flash column chromatography (100% *n*-pentane to 10% AcOEt in *n*-pentane), affording compound **6** (79.6 mg, 69%) as a white solid.

^{1}H NMR (300 MHz, $CDCl_3$) δ 7.66 (d, *J* = 8.4 Hz, 2H), 7.56 – 7.43 (m, 4H), 7.35 (d, *J* = 8.4 Hz, 2H), 7.24 – 7.10 (m, 4H), 4.67 (s, 2H), 2.41 (s, 3H).

^{13}C NMR (75 MHz, $CDCl_3$) δ 144.5, 137.7, 137.1 (t, *J* = 30.3 Hz), 136.8, 136.5, 132.8 (q, *J* = 33.1 Hz), 130.3, 129.6, 127.5, 127.5 (t, *J* = 5.3 Hz), 125.0 (q, *J* = 3.8 Hz), 123.5 (q, *J* = 272.5 Hz), 119.8 (t, *J* = 255.6 Hz), 93.5, 49.0, 21.6.

^{19}F NMR (282 MHz, $CDCl_3$) δ -63.05 (s, 3F), -69.83 (s, 2F).

HRMS (FD+) (m/z): $[M]^+$ calculated for $C_{22}H_{17}F_5INO_2S$, 580.9945; found: 580.9941.

***N*-(difluoro(4-(trifluoromethyl)phenyl)methyl)-*N*-(3-methoxybenzyl)-4-methylbenzenesulfonamide (7)**

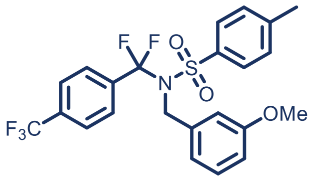

Prepared according to **General Procedure C**, using 3-(bromomethyl)anisole (40.2 mg, 28.0 µL, 0.2 mmol, 1 equiv.), tetrabutylammonium iodide (81.3 mg, 0.22 mmol, 1.1 equiv.) and *N*-tosyl-4-(trifluoromethyl) benzimidoyl chloride solution (6 mL, 0.1 M, 0.6 mmol, 3 equiv.). The crude was purified using flash column chromatography (100% *n*-pentane to 10% AcOEt in *n*-pentane), affording compound **7** (66 mg, 68%) as a yellow oil.

^{1}H NMR (300 MHz, $CDCl_3$) δ 7.55 (s, 4H), 7.50 – 7.42 (m, 2H), 7.30 (d, J = 7.4 Hz, 1H), 7.25 – 7.18 (m, 2H), 7.04 – 6.83 (m, 3H), 4.75 (t, J = 2.1 Hz, 2H), 3.83 (s, 3H), 2.45 (s, 3H).

^{13}C NMR (75 MHz, $CDCl_3$) δ 159.7, 144.3, 138.2, 137.4 (t, J = 30.3 Hz), 137.0, 132.6 (q, J = 32.7 Hz), 129.5, 129.4, 127.6, 127.5 (t, J = 4.8 Hz), 124.9 (q, J = 3.7 Hz), 123.5 (q, J = 272.5 Hz), 119.8 (t, J = 255.6 Hz), 120.5, 113.7, 113.3, 55.2, 49.6, 21.5.

^{19}F NMR (282 MHz, $CDCl_3$) δ -63.07 (s, 3F), -69.90 (s, 2F).

HRMS (FD+) (m/z): $[M]^+$ calculated for $C_{23}H_{20}F_5NO_3S$, 485.1084; found: 485.1101.

***N*-(difluoro(4-(trifluoromethyl)phenyl)methyl)-*N*-(4-formylbenzyl)-4-methylbenzenesulfonamide (8)**

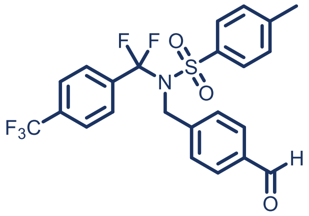

Prepared according to **General Procedure C**, using 4-(bromomethyl)benzaldehyde (39.8 mg, 0.2 mmol, 1 equiv.), tetrabutylammonium iodide (81.3 mg, 0.22 mmol, 1.1 equiv.) and *N*-tosyl-4-(trifluoromethyl) benzimidoyl chloride solution (6 mL, 0.1 M, 0.6 mmol, 3 equiv.). The crude was purified using flash column chromatography (100% *n*-pentane to 10% AcOEt in *n*-pentane), affording compound **8** (58 mg, 60%) as a clear oil.

^{1}H NMR (300 MHz, $CDCl_3$) δ 10.02 (s, 1H), 7.90 – 7.82 (m, 2H), 7.60 – 7.44 (m, 6H), 7.41 – 7.32 (m, 2H), 7.21 – 7.13 (m, 2H), 4.80 (s, 2H), 2.40 (s, 3H).

^{13}C NMR (75 MHz, $CDCl_3$) δ 191.8, 144.7, 143.7, 137.0 (t, J = 30.3 Hz), 136.6, 135.9, 132.9 (q, J = 32.8 Hz), 130.0, 129.7, 128.7, 127.7, 127.6 (t, J = 4.9 Hz), 125.14 (q, J = 3.8 Hz), 123.5 (q, J = 272.8 Hz), 119.9 (t, J = 256.0 Hz), 49.3, 21.6.

^{19}F NMR (282 MHz, $CDCl_3$) δ -63.10 (s, 3F), -69.99 (s, 2F).

HRMS (FD+) (m/z): $[M]^+$ calculated for $C_{23}H_{18}F_5NO_3S$, 483.0928; found: 483.1001.

***N*-(4-cyanobenzyl)-*N*-(difluoro(4-(trifluoromethyl)phenyl)methyl)-4-methylbenzenesulfonamide (9)**

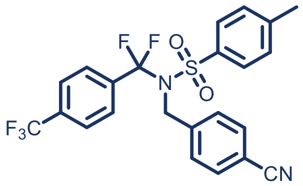

Prepared according to **General Procedure C**, using 4-(bromomethyl) benzonitrile (39.2 mg, 0.2 mmol, 1 equiv.), tetrabutylammonium iodide (81.3 mg, 0.22 mmol, 1.1 equiv.) and *N*-tosyl-4-(trifluoromethyl) benzimidoyl chloride solution (6 mL, 0.1 M, 0.6 mmol, 3 equiv.). The crude was purified using flash column chromatography (100% *n*-pentane to 10% AcOEt in *n*-pentane), affording compound **9** (47 mg, 49%) as a clear oil.

^{1}H NMR (300 MHz, $CDCl_3$) δ 7.72 – 7.61 (m, 2H), 7.60 – 7.46 (m, 6H), 7.42 – 7.32 (m, 2H), 7.25 – 7.16 (m, 2H), 4.80 (s, 2H), 2.44 (s, 3H).

^{13}C NMR (75 MHz, $CDCl_3$) δ 144.9, 142.3, 136.8 (t, J = 30.7 Hz), 136.5, 133.0 (q, J = 32.9 Hz), 132.4, 129.7, 128.8, 127.6 (t, J = 4.8 Hz), 127.6, 125.2 (q, J = 3.8 Hz), 123.4 (q, J = 272.8 Hz), 119.8 (t, J = 255.5 Hz), 118.6, 111.9, 49.1 (t, J = 2.1 Hz), 21.6.

^{19}F NMR (282 MHz, $CDCl_3$) δ -63.10 (s, 3F), -69.94 (s, 2F).

HRMS (FD+) (m/z): $[M]^+$ calculated for $C_{23}H_{17}F_5N_2O_2S$, 480.0931; found: 480.0938.

_N_-(4-benzoylbenzyl)-_N_-(difluoro(4-(trifluoromethyl)phenyl)methyl)-4-methylbenzene-sulfonamide (10)

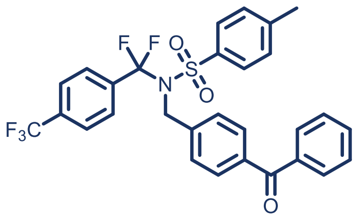

Prepared according to **General Procedure C**, using 4-(bromomethyl) benzophenone (55 mg, 0.2 mmol, 1 equiv.), tetrabutylammonium iodide (81.3 mg, 0.22 mmol, 1.1 equiv.) and *N*-tosyl-4-(trifluoromethyl) benzimidoyl chloride solution (6 mL, 0.1 M, 0.6 mmol, 3 equiv.). The crude was purified using flash column chromatography (100% *n*-pentane to 10% AcOEt in *n*-pentane), affording compound **10** (66 mg, 59%) as a clear oil.

^{1}H NMR (300 MHz, $CDCl_3$) δ 7.85 – 7.74 (m, 4H), 7.67 – 7.56 (m, 1H), 7.56 – 7.45 (m, 8H), 7.39 (d, J = 8.3 Hz, 2H), 7.18 (d, J = 8.1 Hz, 2H), 4.82 (s, 2H), 2.41 (s, 3H).

^{13}C NMR (101 MHz, $CDCl_3$) δ 196.2, 144.6, 141.5, 137.5, 137.2 (t, J = 30.6 Hz), 137.1, 136.8, 132.9 (q, J = 32.9 Hz), 132.6, 130.4, 130.1, 129.6, 128.4, 128.0, 127.7, 127.6 (t, J = 5.0 Hz), 125.1 (q, J = 3.7 Hz), 123.5 (q, J = 272.5 Hz), 119.9 (t, J = 255.8 Hz), 49.3, 21.6.

^{19}F NMR (282 MHz, $CDCl_3$) δ -63.05 (s, 3F), -69.88 (s, 2F).

HRMS (FD+) (m/z): $[M]^+$ calculated for $C_{29}H_{22}F_5NO_3S$, 559.1241; found: 559.1227.

Methyl-4-(((_N_-(difluoro(4-(trifluoromethyl)phenyl)methyl)-4-methylphenyl)sulfonamido) methyl) benzoate (11)

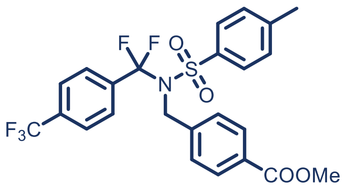

Prepared according to **General Procedure C**, using 4-(bromomethyl) benzonitrile (45.8 mg, 0.2 mmol, 1 equiv.), tetrabutylammonium iodide (81.3 mg, 0.22 mmol, 1.1 equiv.) and *N*-tosyl-4-(trifluoromethyl) benzimidoyl chloride solution (6 mL, 0.1 M, 0.6 mmol, 3 equiv.). The crude was purified using flash column chromatography (100% *n*-pentane to 10% AcOEt in *n*-pentane), affording compound **11** (51 mg, 50%) as a clear oil.

^{1}H NMR (300 MHz, $CDCl_3$) δ 8.01 (d, J = 8.2 Hz, 2H), 7.59 – 7.41 (m, 6H), 7.36 (d, J = 8.1 Hz, 2H), 7.16 (d, J = 8.1 Hz, 2H), 4.78 (s, 2H), 3.93 (s, 3H), 2.40 (s, 3H).

^{13}C NMR (75 MHz, $CDCl_3$) δ 166.8, 144.5, 141.9, 137.1 (t, J = 30.2 Hz), 136.7, 132.8 (q, J = 32.9 Hz), 129.8, 129.7, 129.6, 128.1, 127.7, 127.5 (t, J = 3.8 Hz), 125.0 (q, J = 3.8 Hz), 123.4 (q, J = 272.6 Hz), 119.8 (t, J = 255.6 Hz), 52.2, 49.2, 21.5.

^{19}F NMR (282 MHz, $CDCl_3$) δ -63.10 (s, 3F), -69.93 (s, 2F).

HRMS (FD+) (m/z): $[M]^+$ calculated for $C_{24}H_{20}F_5NO_4S$, 513.1033; found: 513.1252.

_N_-((5-chlorobenzo[b]thiophen-3-yl)methyl)-_N_-(difluoro(4-(trifluoromethyl)phenyl)methyl)-4-methylbenzenesulfonamide (12)

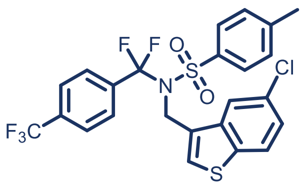

Prepared according to **General Procedure C**, using 3-(bromomethyl)-5-chlorobenzo[b]thiophene (52.3 mg, 0.2 mmol, 1 equiv.), tetrabutylammonium iodide (81.3 mg, 0.22 mmol, 1.1 equiv.) and *N*-tosyl-4-(trifluoromethyl) benzimidoyl chloride solution (6 mL, 0.1 M, 0.6 mmol, 3 equiv.). The crude was purified using flash column chromatography (100% *n*-pentane to 10% AcOEt in *n*-pentane), affording compound **12** (92 mg, 84%) as a white solid.

^{1}H NMR (300 MHz, $CDCl_3$) δ 7.83 – 7.70 (m, 2H), 7.62 (s, 1H), 7.59 – 7.44 (m, 6H), 7.32 (dd, *J* = 8.5, 2.1 Hz, 1H), 7.20 (d, *J* = 8.3 Hz, 2H), 4.96 (s, 2H), 2.43 (s, 3H).

^{13}C NMR (75 MHz, $CDCl_3$) δ 144.5, 138.6, 138.3, 137.1 (t, *J* = 31.0 Hz), 136.9, 132.7 (q, *J* = 32.8 Hz), 130.7, 129.6, 128.3, 127.5, 127.4 (t, *J* = 4.8 Hz), 125.0, 124.9 (q, *J* = 4.5 Hz), 123.8, 123.4 (q, *J* = 272.3 Hz), 121.1, 119.9 (t, *J* = 256.4 Hz), 43.5, 21.5.

^{19}F NMR (282 MHz, $CDCl_3$) δ -63.10 (s, 3F), -70.12 (s, 2F).

HRMS (FD+) (m/z): $[M]^+$ calculated for $C_{24}H_{17}C_{l1}F_5NO_2S_2$, 545.0309; found: 545.0304.

_N_-(difluoro(4-(trifluoromethyl)phenyl)methyl)-4-methyl-_N_-(quinolin-8-ylmethyl)benzenesulfonamide (13)

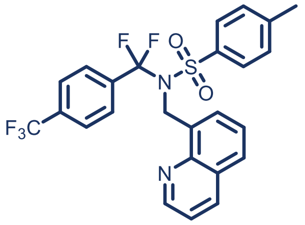

Prepared according to **General Procedure C**, using 8-(bromomethyl)quinoline (44.2 mg, 0.2 mmol, 1 equiv.), tetrabutylammonium iodide (81.3 mg, 0.22 mmol, 1.1 equiv.) and *N*-tosyl-4-(trifluoromethyl) benzimidoyl chloride solution (6 mL, 0.1 M, 0.6 mmol, 3 equiv.). The crude was purified using flash column chromatography (100% *n*-pentane to 10% AcOEt in *n*-pentane), affording compound **13** (45 mg, 44%) as a clear oil.

^{1}H NMR (300 MHz, $CDCl_3$) δ 8.75 (dd, *J* = 4.2, 1.8 Hz, 1H), 8.15 – 8.03 (m, 2H), 7.77 – 7.66 (m, 3H), 7.62 – 7.54 (m, 1H), 7.48 (d, *J* = 8.3 Hz, 2H), 7.41 – 7.32 (m, 3H), 7.28 (d, *J* = 8.1 Hz, 2H), 5.46 (s, 2H), 2.45 (s, 3H).

^{13}C NMR (75 MHz, $CDCl_3$) δ 149.2, 145.3, 144.5, 137.3 (t, *J* = 30.5 Hz), 137.0, 136.3, 134.9, 132.4 (q, *J* = 32.8 Hz), 129.6, 128.0, 127.9, 127.8, 127.2, 127.2 (t, *J* = 5.0 Hz), 126.4, 124.7 (q, *J* = 3.7 Hz), 123.4 (q, *J* = 274.2 Hz), 121.5 (t, *J* = 260.9 Hz), 121.1, 46.3, 21.6.

^{19}F NMR (282 MHz, $CDCl_3$) δ -63.10 (s, 3F), -70.38 (s, 2F).

HRMS (FD+) (m/z): $[M]^+$ calculated for $C_{25}H_{19}F_5N_2O_2S$, 507.1166; found: 507.1156.

_N_-(difluoro(4-(trifluoromethyl)phenyl)methyl)-4-methyl-_N_-((6-(trifluoromethyl)pyridin-3-yl)methyl)benzenesulfonamide (14)

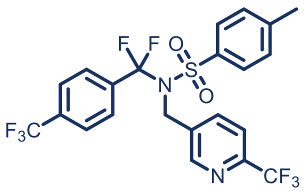

Prepared according to **General Procedure C**, using 5-(bromomethyl)-2-(trifluoromethyl)pyridine (48.0 mg, x µL, 0.2 mmol, 1 equiv.), tetrabutylammonium iodide (81.3 mg, 0.22 mmol, 1.1 equiv.) and *N*-tosyl-4-(trifluoromethyl) benzimidoyl chloride solution (6 mL, 0.1 M, 0.6 mmol, 3 equiv.). The crude was purified using flash column chromatography (100% *n*-pentane to 10% AcOEt in *n*-pentane), affording compound **14** (66 mg, 68%) as a yellow oil.

^1H NMR (300 MHz, $CDCl_3$) δ 8.72 (d, J = 2.4 Hz, 1H), 8.03 (dd, J = 8.1, 2.4 Hz, 1H), 7.71 (d, J = 8.2 Hz, 1H), 7.60 – 7.47 (m, 4H), 7.34 (d, J = 8.4 Hz, 2H), 7.20 (d, J = 8.2 Hz, 2H), 4.88 (d, J = 2.1 Hz, 2H), 2.43 (s, 3H).

^{13}C NMR (75 MHz, $CDCl_3$) δ 149.7, 147.7 (q, J = 34.9 Hz), 145.0, 137.4, 136.4 (t, J = 30.1 Hz), 136.2, 135.0, 133.1 (q, J = 32.4 Hz), 129.7, 127.6 (t, J = 4.9 Hz), 127.5, 125.2 (q, J = 3.7 Hz), 123.3 (q, J = 272.9 Hz) 121.4 (q, J = 274.0 Hz), 120.3 (q, J = 2.7 Hz), 119.8 (t, J = 255.7 Hz), 46.5 (t, J = 2.3 Hz), 21.5.

^{19}F NMR (282 MHz, $CDCl_3$) δ -63.17 (s, 3F), -67.90 (s, 3F), -70.07 (s, 2F).

HRMS (FD+) (m/z): $[M+H]^+$ calculated for $C_{22}H_{16}F_8N_2O_2S$, 525.0883; found: 525.0880.

***N*-(difluoro(4-(trifluoromethyl)phenyl)methyl)-4-methyl-*N*-((5-(trifluoromethyl)furan-2-yl)methyl)benzenesulfonamide (15)**

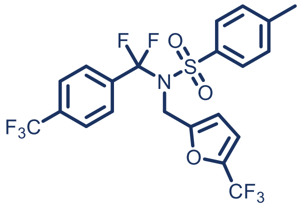

Prepared according to **General Procedure C**, using 2-(bromomethyl)-5-(trifluoromethyl)furan (45.8 mg, 0.2 mmol, 1 equiv.), tetrabutylammonium iodide (81.3 mg, 0.22 mmol, 1.1 equiv.) and *N*-tosyl-4-(trifluoromethyl) benzimidoyl chloride solution (6 mL, 0.1 M, 0.6 mmol, 3 equiv.). The crude was purified using flash column chromatography (100% *n*-pentane to 10% AcOEt in *n*-pentane), affording compound **15** (55.2 mg, 54%) as an oil.

^1H NMR (300 MHz, $CDCl_{3z}$) δ 7.57 – 7.44 (m, 4H), 7.37 – 7.28 (m, 2H), 7.14 (d, J = 8.4 Hz, 2H), 6.73 (dd, J = 3.5, 1.3 Hz, 1H), 6.39 (d, J = 3.5 Hz, 1H), 4.83 (s, 2H), 2.39 (s, 3H).

^{13}C NMR (75 MHz, $CDCl_3$) δ 153.0, 144.5, 141.5 (q, J = 42.8 Hz), 136.9, 136.6 (t, J = 30.1 Hz), 133.2 (q, J = 34.4 Hz), 129.4, 127.6 (t, J = 4.8 Hz), 127.4, 125.1 (q, J = 3.8 Hz), 123.4 (q, J = 272.4 Hz), 119.4 (t, J = 256.2 Hz), 118.9 (q, J = 266.9 Hz), 112.5 (q, J = 2.9 Hz), 110.1, 41.5, 21.5.

^{19}F NMR (282 MHz, $CDCl_3$) δ -63.20 (s, 3F), -64.18 (s, 3F), -71.00 (s, 2F).

HRMS (FD+) (m/z): $[M]^+$ calculated for $C_{21}H_{15}F_8NO_3S$, 513.0645; found: 513.0636.

***N*-cinnamyl-*N*-(difluoro(4-(trifluoromethyl)phenyl)methyl)-4-methylbenzenesulfonamide (16)**

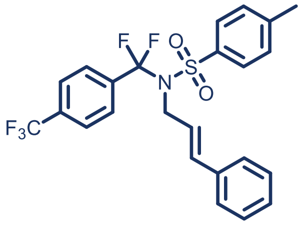

Prepared according to **General Procedure C**, using cinnamyl bromide (39.4 mg, 0.2 mmol, 1 equiv.), tetrabutylammonium iodide (81.3 mg, 0.22 mmol, 1.1 equiv.) and *N*-tosyl-4-(trifluoromethyl) benzimidoyl chloride solution (6 mL, 0.1 M, 0.6 mmol, 3 equiv.). The crude was purified using flash column chromatography (100% *n*-pentane to 10% AcOEt in *n*-pentane), affording compound **16** (82 mg, 85%) as a yellow oil.

^1H NMR (300 MHz, $CDCl_3$) δ 7.66 – 7.53 (m, 6H), 7.38 – 7.27 (m, 5H), 7.21 (d, J = 8.0 Hz, 2H), 6.51 – 6.35 (m, 1H), 6.28 – 6.11 (m, 1H), 4.38 – 4.24 (m, 2H), 2.40 (s, 3H).

^{13}C NMR (75 MHz, $CDCl_3$) δ 144.3, 137.6 (t, J = 30.8 Hz), 137.4, 136.2, 134.2, 132.8 (q, J = 32.7 Hz), 129.5, 128.6, 128.1, 127.7, 127.5 (t, J = 4.8 Hz), 126.5, 125.1 (q, J = 3.7 Hz), 124.4, 123.5 (q, J = 272.6 Hz), 119.8 (t, J = 255.4 Hz), 48.5, 21.5.

^{19}F NMR (282 MHz, $CDCl_3$) δ -63.02 (s, 3F), -70.20 (s, 2F).

HRMS (FD+) (m/z): $[M]^+$ calculated for $C_{24}H_{20}F_5NO_2S$, 481.1135; found: 481.1140.

(E)-*N*-(difluoro(4-(trifluoromethyl)phenyl)methyl)-*N*-(3,7-dimethylocta-2,6-dien-1-yl)-4-methylbenzenesulfonamide (17)

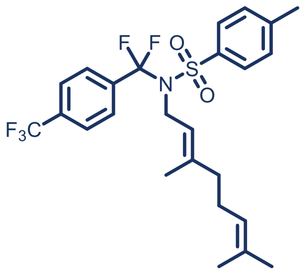

Prepared according to **General Procedure C**, using geranyl bromide (43.4 mg, 39.7 µL, 0.2 mmol, 1 equiv.), tetrabutylammonium iodide (81.3 mg, 0.22 mmol, 1.1 equiv.) and *N*-tosyl-4-(trifluoromethyl) benzimidoyl chloride solution (6 mL, 0.1 M, 0.6 mmol, 3 equiv.). The crude was purified using flash column chromatography (100% *n*-pentane to 10% AcOEt in *n*-pentane), affording compound **17** (67 mg, 67%) as a yellow oil.

^{1}H NMR (300 MHz, $CDCl_3$) δ 7.69 – 7.51 (m, 6H), 7.22 (d, *J* = 8.1 Hz, 2H), 5.25 (t, *J* = 6.9 Hz, 1H), 5.08 (t, *J* = 6.1 Hz, 1H), 4.16 (d, *J* = 6.7 Hz, 2H), 2.42 (s, 3H), 2.12 – 1.92 (m, 4H), 1.69 (s, 3H), 1.62 (s, 3H), 1.53 (s, 3H).

^{13}C NMR (75 MHz, $CDCl_3$) δ 144.1, 139.7, 138.0 (t, *J* = 31.4 Hz), 137.7, 132.7 (q, *J* = 32.7 Hz), 131.9, 129.5, 127.6, 127.5 (t, *J* = 4.7 Hz), 125.2 (q, *J* = 3.7 Hz), 123.8, 123.6 (q, *J* = 272.2 Hz), 120.1, 119.9 (t, *J* = 255.2 Hz), 44.8, 39.6, 26.3, 25.8, 21.6, 17.7, 16.1.

^{19}F NMR (282 MHz, $CDCl_3$) δ -63.04 (s, 3F), -70.12 (s, 2F).

HRMS (FD+) (m/z): $[M]^+$ calculated for $C_{25}H_{28}F_5NO_2S$, 501.1761; found: 501.1753.

***N*-(but-2-yn-1-yl)-*N*-(difluoro(4-(trifluoromethyl)phenyl)methyl)-4-methylbenzenesulfonamide (18)**

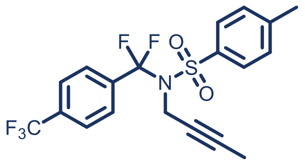

Prepared according to **General Procedure C**, using 1-bromo-2-butyne (26.6 mg, 17.7 µL, 0.2 mmol, 1 equiv.), tetrabutylammonium iodide (83.26 mg, 0.22 mmol, 1.1 equiv.) and *N*-tosyl-4-(trifluoromethyl) benzimidoyl chloride solution (6 mL, 0.1 M, 0.6 mmol, 3 equiv.). The crude was purified using flash column chromatography (100% *n*-pentane to 10% AcOEt in *n*-pentane), affording compound **18** (36.7 mg, 44%) as a clear oil.

^{1}H NMR (300 MHz, $CDCl_3$) δ 7.68 – 7.53 (m, 6H), 7.23 (d, *J* = 8.1 Hz, 2H), 4.29 (q, *J* = 1.9 Hz, 2H), 2.43 (s, 3H), 1.75 (t, *J* = 2.3 Hz, 3H).

^{13}C NMR (75 MHz, $CDCl_3$) δ 144.4, 137.3 (t, *J* = 31.0 Hz), 137.2, 132.9 (q, *J* = 32.9 Hz), 129.4, 127.9, 127.6 (t, *J* = 4.8 Hz), 125.2 (q, *J* = 3.7 Hz), 123.6 (q, *J* = 272.4 Hz), 119.5 (t, *J* = 255.5 Hz), 80.8, 73.9, 36.2, 21.6, 3.5.

^{19}F NMR (282 MHz, $CDCl_3$) δ -63.07 (s, 3F), -71.08 (s, 2F).

HRMS (FD+) (m/z): $[M]^+$ calculated for $C_{19}H_{16}F_5NO_2S$, 417.0821; found: 417.0819.

***N*-(difluoro(4-(trifluoromethyl)phenyl)methyl)-*N*,4-dimethylbenzenesulfonamide (19)**

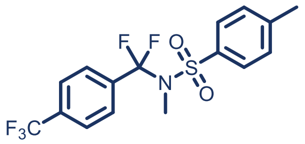

Prepared according to a modified version of **General Procedure C**, using methyl trifluoromethanesulfonate (32.8 mg, 22.6 µL, 0.2 mmol, 1.0 equiv.) and *N*-tosyl-4-(trifluoromethyl) benzimidoyl chloride solution (6 mL, 0.1 M, 0.6 mmol, 3 equiv.). The crude was purified using flash column chromatography (100% *n*-pentane to 10% AcOEt in *n*-pentane), affording compound **19** (63.2 mg, 83%) as a clear oil.

^{1}H NMR (300 MHz, $CDCl_3$) δ 7.70 – 7.56 (m, 6H), 7.33 – 7.23 (m, 2H), 3.06 (t, *J* = 1.9 Hz, 3H), 2.43 (s, 3H).

^{13}C NMR (75 MHz, $CDCl_3$) δ 144.6, 137.8 (t, J = 31.0 Hz), 136.2, 132.9 (qt, J = 32.8, 1.6 Hz), 129.7, 127.8, 127.2 (t, J = 4.8 Hz), 125.4 (q, J = 3.7 Hz), 123.6 (q, J = 272.2 Hz), 119.7 (t, J = 255.0 Hz), 32.4 (t, J = 2.7 Hz), 21.6.

^{19}F NMR (282 MHz, $CDCl_3$) δ -63.02 (s, 3F), -74.12 (s, 2F).

HRMS (FD+) (m/z): $[M]^+$ calculated for $C_{16}H_{14}F_5NO_2S$, 379.0665; found: 379.0656.

***N*-(difluoro(4-(trifluoromethyl)phenyl)methyl)-*N*,4-dimethylbenzenesulfonamide-^{13}C (20)**

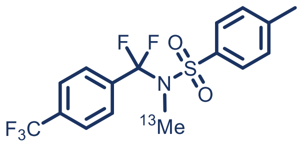

Prepared according to a modified version of **General Procedure C**, using methyl-^{13}C trifluoromethanesulfonate (32.8 mg, 22.6 µL, 0.2 mmol, 1 equiv.) and *N*-tosyl-4-(trifluoromethyl) benzimidoyl chloride solution (6 mL, 0.1 M, 0.6 mmol, 3 equiv.). The crude was purified using flash column chromatography (100% *n*-pentane to 10% AcOEt in *n*-pentane), affording compound **20** (62.5 mg, 82%) as a clear oil.

^1H NMR (300 MHz, $CDCl_3$) δ 7.70 – 7.56 (m, 6H), 7.33 – 7.23 (m, 2H), 3.05 (dt, J = 142.2, 1.9 Hz), 2.44 (s, 3H).

^{13}C NMR (75 MHz, $CDCl_3$) δ 144.6, 137.8 (t, J = 30.6 Hz), 136.2, 132.9 (qt, J = 32.8, 1.6 Hz), 129.7, 127.8, 127.2 (t, J = 4.8 Hz), 125.4 (q, J = 3.7 Hz), 123.6 (q, J = 272.2 Hz), 119.7 (t, J = 255.0 Hz), 32.5 (t, J = 2.7 Hz), 21.6.

^{19}F NMR (282 MHz, $CDCl_3$) δ -63.02 (s, 3F), -74.13 (s, 2F).

HRMS (FD+) (m/z): $[M]^+$ calculated for $C_{15}\,^{13}CH_{14}F_5NO_2S$, 380.0698; found: 380.0697.

***N*-(difluoro(4-(trifluoromethyl)phenyl)methyl)-*N*-((4-(4-fluorophenyl)-6-isopropyl-2-(N-methylmethylsulfonamido)pyrimidin-5-yl)methyl)-4-methylbenzenesulfonamide (21)**

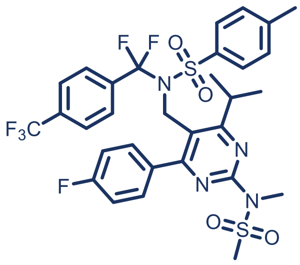

Prepared according to a modified **General Procedure C**, using bromide *N*-(5-(bromomethyl)-4-(4-fluorophenyl)-6-isopropylpyrimidin-2-yl)-*N*-methylmethanesulfonamide (185 mg, 0.44 mmol, 1 equiv.), tetrabutylammonium iodide (246 mg, 0.67 mmol, 1.5 equiv.) and *N*-tosyl-4-(trifluoromethyl) benzimidoyl chloride solution (13.2 mL, 0.1 M, 1.32 mmol, 3 equiv.). The crude was purified using flash column chromatography (100% *n*-pentane to 20% AcOEt in *n*-pentane), affording compound **21** (64.4 mg, 21%) as a clear oil.

^1H NMR (300 MHz, $CDCl_3$) δ 7.50 (d, J = 8.4 Hz, 2H), 7.46 – 7.37 (m, 2H), 7.26 – 7.10 (m, 6H), 7.01 (d, J = 8.4 Hz, 2H), 5.12 (s, 2H), 3.68 (h, J = 6.6 Hz, 1H), 3.52 (s, 3H), 3.50 (s, 3H), 2.39 (s, 3H), 1.28 (d, J = 6.6 Hz, 6H).

^{13}C NMR (75 MHz, $CDCl_3$) δ 177.5, 166.9, 163.6 (d, J = 250.2 Hz), 157.5, 144.8, 137.3 (t, J = 30.6 Hz), 136.5, 134.3 (d, J = 3.4 Hz), 132.8 (q, J = 32.9 Hz), 131.7 (d, J = 8.5 Hz), 129.6, 127.4, 127.2 (t, J = 5.0 Hz), 125.1 (q, J = 3.7 Hz), 123.5 (q, J = 272.4 Hz), 120.0 (t, J = 258.4 Hz), 116.9, 115.5 (d, J = 21.7 Hz), 42.6 (t, J = 2.6 Hz), 42.5, 33.0, 31.5, 22.0, 21.6.

^{19}F NMR (282 MHz, $CDCl_3$) δ -63.01 (s, 3F), -70.60 (s, 2F), -111.02 (s, 1F).

HRMS (FD+) (m/z): $[M]^+$ calculated for $C_{31}H_{30}F_6N_4O_4S_2$, 700.1613; found: 700.1599.

N-(difluoro(4-(trifluoromethyl)phenyl)methyl)-N-(4-(N,N-dipropylsulfamoyl)benzyl)-4-methylbenzenesulfonamide (22)

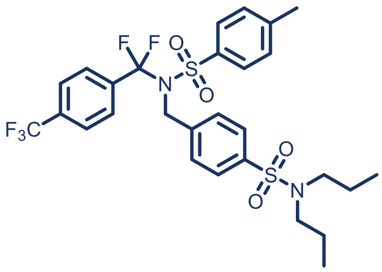

Prepared according to **General Procedure C**, using 4-(bromomethyl)-N,N-dipropylbenzenesulfonamide **SI-2** (66.8 mg, 0.2 mmol, 1 equiv.), tetrabutylammonium iodide (81.3 mg, 0.22 mmol, 1.1 equiv.) and *N*-tosyl-4-(trifluoromethyl) benzimidoyl chloride solution (6 mL, 0.1 M, 0.6 mmol, 3 equiv.). The crude was purified using flash column chromatography (100% *n*-pentane to 10% AcOEt in *n*-pentane), affording compound **22** (67 mg, 64%) as a clear oil.

^{1}H NMR (300 MHz, $CDCl_3$) δ 7.85 – 7.77 (m, 2H), 7.58 – 7.46 (m, 6H), 7.41 – 7.33 (m, 2H), 7.23 – 7.15 (m, 2H), 4.81 (t, J = 2.1 Hz, 2H), 3.19 – 3.03 (m, 4H), 2.43 (s, 3H), 1.70 – 1.49 (m, 4H), 0.90 (t, J = 7.4 Hz, 6H).

^{13}C NMR (75 MHz, $CDCl_3$) δ 144.7, 141.4, 139.7, 136.9 (t, J = 29.0 Hz), 136.5, 132.9 (q, J = 33.2), 129.6, 128.7, 127.5, 127.5 (t, J = 4.7 Hz), 127.3, 125.1 (q, J = 3.7 Hz), 122.5 (q, J = 273.9 Hz), 121.5 (t, J = 256.4 Hz), 50.0, 48.9, 22.0, 21.5, 11.2.

^{19}F NMR (282 MHz, $CDCl_3$) δ -63.07 (s, 3F), -69.79 (s, 2F).

HRMS (FD+) (m/z): $[M]^+$ calculated for $C_{28}H_{31}F_5N_2O_4S_2$, 618.1645; found: 618.1634.

N-(difluoro(4-(trifluoromethyl)phenyl)methyl)-N-(3-(5-(2-fluorophenyl)-1,2,4-oxadiazol-3-yl)benzyl)-4-methylbenzenesulfonamide (23)

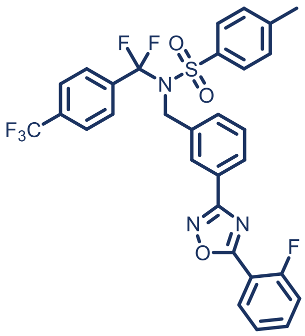

Prepared according to a modified **General Procedure C**, using 3-(3-(bromomethyl)phenyl)-5-(2-fluorophenyl)-1,2,4-oxadiazole **SI-3** (112.7 mg, 0.34 mmol, 1 equiv.), tetrabutylammonium iodide (81.3 mg, 0.22 mmol, 0.65 equiv.) and *N*-tosyl-4-(trifluoromethyl) benzimidoyl chloride solution (6 mL, 0.1 M, 0.6 mmol, 1.76 equiv.). The crude was purified using flash column chromatography (100% *n*-pentane to 10% AcOEt in *n*-pentane), affording compound **23** (132 mg, 63%) as a clear oil.

^{1}H NMR (300 MHz, $CDCl_3$) δ 8.27 – 8.16 (m, 1H), 8.15 – 8.08 (m, 1H), 8.05 (s, 1H), 7.68 – 7.27 (m, 11H), 7.20 – 7.11 (m, 2H), 4.85 (s, 2H), 2.37 (s, 3H).

^{13}C NMR (75 MHz, $CDCl_3$) δ 172.8 (d, J = 4.5 Hz), 168.4, 160.8 (d, J = 260.6 Hz), 144.5, 137.8, 137.2 (t, J = 30.7 Hz), 136.8, 134.7 (d, J = 8.7 Hz), 132.8 (q, J = 32.7 Hz), 131.0 (2C), 129.6, 129.3, 127.7, 127.6 (t, J = 4.8 Hz), 127.2, 127.0, 126.9, 125.1 (q, J = 3.7 Hz), 124.8 (d, J = 3.8 Hz), 123.6 (q, J = 273.2 Hz), 120.0 (t, J = 256.0 Hz), 117.2 (d, J = 20.9 Hz), 112.8 (d, J = 11.4 Hz), 49.2, 21.5.

^{19}F NMR (282 MHz, $CDCl_3$) δ -63.12 (s, 3F), -70.19 (s, 2F), -108.32 (s, 1F).

HRMS (FD+) (m/z): $[M]^+$ calculated for $C_{30}H_{21}F_6N_3O_3S$, 617.1208; found: 617.1182.

(S)-2-(4-(((*N*-(difluoro(4-(trifluoromethyl)phenyl)methyl)-4-methylphenyl)sulfonamido)methyl)-3-ethoxyphenyl)-*N*-(3-methyl-1-(2-(piperidin-1-yl)phenyl)butyl)acetamide (24)

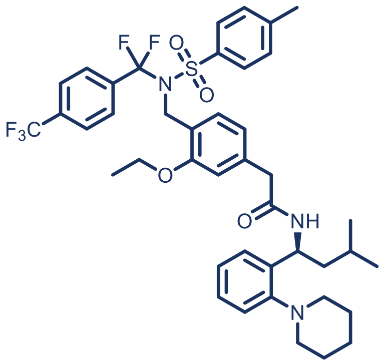

Prepared according to **General Procedure C**, using bromide **SI-5** (100.3 mg, 0.2 mmol, 1 equiv.), tetrabutylammonium iodide (81.3 mg, 0.22 mmol, 1.1 equiv.) and *N*-tosyl-4-(trifluoromethyl) benzimidoyl chloride solution (6 mL, 0.1 M, 0.6 mmol, 3 equiv.). The crude was purified using flash column chromatography (100% *n*-pentane to 10% AcOEt in *n*-pentane), affording compound **24** (37 mg, 24%) as a clear oil.

^{1}H NMR (300 MHz, $CDCl_3$) δ 7.63 – 7.41 (m, 7H), 7.26 – 7.13 (m, 4H), 7.11 – 6.99 (m, 2H), 6.88 – 6.79 (m, 1H), 6.69 – 6.52 (m, 2H), 5.46 – 5.32 (m, 1H), 4.72 (s, 2H), 3.90 – 3.70 (m, 2H), 3.50 (s, 2H), 3.08 – 2.83 (m, 2H), 2.71 – 2.53 (m, 2H), 2.43 (s, 3H), 1.77 – 1.35 (m, 9H), 1.28 (t, J = 6.9 Hz, 3H), 1.01 – 0.80 (m, 6H).

^{13}C NMR (75 MHz, $CDCl_3$) δ 169.5, 156.0, 152.5, 144.3, 138.9, 137.5 (t, J = 30.1 Hz), 137.2, 135.9, 132.5 (q, J = 33.4 Hz), 129.5, 129.0, 127.8, 127.7, 127.5, 127.3 (t, J = 4.8 Hz), 125.0, 124.8 (q, J = 3.7 Hz), 123.9, 123.4 (q, J = 273.0 Hz), 122.7, 121.1, 119.9 (t, J = 255.1 Hz), 111.6, 63.5, 49.6, 46.7, 44.4, 44.1, 26.8, 25.4, 24.2, 22.8, 22.5, 21.6, 14.7.

^{19}F NMR (282 MHz, $CDCl_3$) δ -63.00 (s, 3F), -69.96 (s, 2F).

HRMS (FD+) (m/z): $[M+H]^+$ calculated for $C_{42}H_{48}F_5N_3O_4S$, 786.3364; found: 786.3365.

(1R,2S,5R)-2-isopropyl-5-methylcyclohexyl 4-(((*N*-(difluoro(4-(trifluoromethyl)phenyl)methyl)-4-methylphenyl)sulfonamido)methyl)benzoate (25)

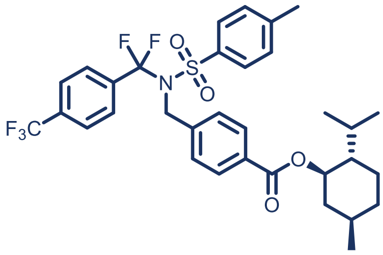

Prepared according to **General Procedure C**, using bromide **SI-1** (70.6 mg, 0.2 mmol, 1 equiv.), tetrabutylammonium iodide (81.3 mg, 0.22 mmol, 1.1 equiv.) and *N*-tosyl-4-(trifluoromethyl) benzimidoyl chloride solution (6 mL, 0.1 M, 0.6 mmol, 3 equiv.). The crude was purified using flash column chromatography (100% *n*-pentane to 10% AcOEt in *n*-pentane), affording compound **25** (60.5 mg, 47%) as a clear oil.

^{1}H NMR (300 MHz, $CDCl_3$) δ 8.01 (d, J = 8.4 Hz, 2H), 7.55 – 7.47 (m, 4H), 7.44 (d, J = 8.4 Hz, 2H), 7.37 (d, J = 8.4 Hz, 2H), 7.17 (d, J = 8.0 Hz, 2H), 4.95 (td, J = 10.9, 4.4 Hz, 1H), 4.78 (s, 2H), 2.41 (s, 3H), 2.19 – 2.09 (m, 1H), 1.97 (pd, J = 7.0, 2.8 Hz, 1H), 1.79 – 1.71 (m, 2H), 1.66 – 1.51 (m, 2H), 1.19 – 1.08 (m, 2H), 0.97 – 0.90 (m, 7H), 0.81 (d, J = 7.0 Hz, 3H).

^{13}C NMR (75 MHz, $CDCl_3$) δ 165.8, 144.5, 141.7, 137.3 (t, J = 29.7 Hz), 136.7, 132.8 (q, J = 33.0 Hz), 130.4, 129.8, 129.6, 128.0, 127.6, 127.5 (t, J = 5.0 Hz), 125.0 (q, J = 3.8 Hz), 123.1 (q, J = 272.1 Hz), 119.9 (t, J = 255.6 Hz), 75.0, 49.2, 41.0, 34.3, 31.5, 26.6, 23.7, 22.0, 21.5, 20.8, 16.6.

^{19}F NMR (282 MHz, $CDCl_3$) δ -63.10 (s, 3F), -69.99 (s, 2F).

HRMS (FD+) (m/z): $[M]^+$ calculated for $C_{33}H_{36}F_5NO_4S$, 637.2285; found: 637.2277.

4.3 Scope of Aryl Groups

***N*-cinnamyl-*N*-(difluoro(4-fluorophenyl)methyl)-4-methylbenzenesulfonamide (26)**

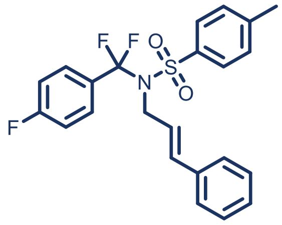

Prepared according to **General Procedure D**, cinnamyl bromide (39.4 mg, 0.2 mmol, 1 equiv.), tetrabutylammonium iodide (83.26 mg, 0.22 mmol, 1.1 equiv.) and 4-fluoro-*N*-tosylbenzimidoyl chloride solution (**S-7**) (8 mL, 0.1 M, 0.8 mmol, 4 equiv.). The crude was purified using flash column chromatography (100% *n*-pentane to 10% AcOEt in *n*-pentane), affording compound **26** (55 mg, 64%) as a clear oil.

^{1}H NMR (300 MHz, $CDCl_3$) δ 7.66 (d, *J* = 8.3 Hz, 2H), 7.55 – 7.47 (m, 2H), 7.41 – 7.22 (m, 7H), 7.05 (t, *J* = 8.7 Hz, 2H), 6.53 – 6.37 (m, 1H), 6.30 – 6.13 (m, 1H), 4.34 – 4.24 (m, 2H), 2.43 (s, 3H).

^{13}C NMR (75 MHz, $CDCl_3$) δ 164.1 (d, *J* = 251.4 Hz), 144.2, 137.8, 136.3, 133.9, 130.1 (td, *J* = 31.2, 3.3 Hz), 129.5, 129.2(dt, *J* = 9.3, 4.8 Hz), 128.6, 128.3, 127.7, 126.6, 124.7, 120.3 (t, *J* = 254.6 Hz), 115.3 (d, *J* = 22.3 Hz), 48.8, 21.6.

^{19}F NMR (282 MHz, $CDCl_3$) δ -68.72 (s, 2F), -108.86 – -109.12 (m, 1F).

HRMS (FD+) (m/z): $[M]^+$ calculated for $C_{23}H_{20}F_3NO_2S$, 431.1167; found: 431.1160.

***N*-((4-chlorophenyl)difluoromethyl)-*N*-cinnamyl-4-methylbenzenesulfonamide (27)**

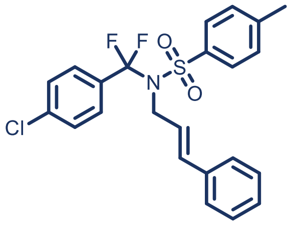

Prepared according to **General Procedure D**, cinnamyl bromide (39.4 mg, 0.2 mmol, 1 equiv.), tetrabutylammonium iodide (83.26 mg, 0.22 mmol, 1.1 equiv.) and 4-chloro-*N*-tosylbenzimidoyl chloride solution (**S-8**) (8 mL, 0.1 M, 0.8 mmol, 4 equiv.). The crude was purified using flash column chromatography (100% *n*-pentane to 10% AcOEt in *n*-pentane), affording compound **27** (68 mg, 76%) as a clear oil.

^{1}H NMR (300 MHz, $CDCl_3$) δ 7.64 (d, *J* = 8.4 Hz, 2H), 7.45 (d, *J* = 8.8 Hz, 2H), 7.40 – 7.28 (m, 7H), 7.25 (d, *J* = 8.0 Hz, 2H), 6.53 – 6.35 (m, 1H), 6.27 – 6.08 (m, 1H), 4.28 (d, *J* = 6.6 Hz, 2H), 2.44 (s, 3H).

^{13}C NMR (75 MHz, $CDCl_3$) δ 144.2, 137.6, 137.2 (t, *J* = 2.2 Hz), 136.2, 134.0, 132.5 (t, *J* = 31.3 Hz), 129.5, 128.6, 128.4, 128.3 (t, *J* = 4.6 Hz), 128.0, 127.7, 126.6, 124.6, 120.2 (t, *J* = 254.9 Hz), 48.7, 21.6.

^{19}F NMR (282 MHz, $CDCl_3$) δ -69.59 (s, 2F).

HRMS (FD+) (m/z): $[M]^+$ calculated for $C_{23}H_{20}ClF_2NO_2S$, 447.0871; found: 447.0866.

***N*-((4-bromo-2-fluorophenyl)difluoromethyl)-*N*-cinnamyl-4-methylbenzenesulfonamide (28)**

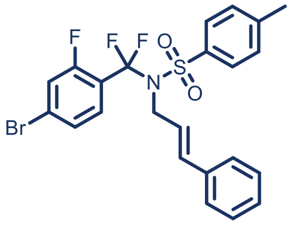

Prepared according to **General Procedure D**, cinnamyl bromide (39.4 mg, 0.2 mmol, 1 equiv.), tetrabutylammonium iodide (83.26 mg, 0.22 mmol, 1.1 equiv.) and 4-bromo-2-fluoro-*N*-tosylbenzimidoyl chloride solution (**S-9**) (8 mL, 0.1 M, 0.8 mmol, 4 equiv.). The crude was purified using flash column chromatography (100% *n*-pentane to 10% AcOEt in *n*-pentane), affording compound **28** (83 mg, 81%) as a clear oil.

^{1}H NMR (300 MHz, $CDCl_3$) δ 7.57 – 7.46 (m, 3H), 7.40 – 7.24 (m, 6H), 7.23 – 7.16 (m, 2H), 7.07 – 6.97 (m, 1H), 6.53 – 6.41 (m, 1H), 6.32 – 6.16 (m, 1H), 4.44 – 4.34 (m, 2H), 2.40 (s, 3H).

^{13}C NMR (75 MHz, $CDCl_3$) δ 159.5 (dt, J = 259.6, 3.7 Hz), 144.3, 137.0, 136.4, 133.7, 130.4 (td, J = 5.4, 1.8 Hz), 129.4, 128.6, 128.0, 127.6, 127.1 (d, J = 3.7 Hz), 126.6, 126.3 (dt, J = 9.3, 1.6 Hz), 124.8, 121.0 (td, J = 31.8, 10.7 Hz), 119.9 (d, J = 24.1 Hz), 118.1 (td, J = 254.8, 1.8 Hz), 48.4, 21.6.

^{19}F NMR (282 MHz, $CDCl_3$) δ -68.57 (d, J = 11.8 Hz, 2F), -109.50 (t, J = 11.8 Hz, 1F).

HRMS (FD+) (m/z): $[M]^+$ calculated for $C_{23}H_{19}BrF_3NO_2S$, 509.0272; found: 509.0280.

***N*-cinnamyl-*N*-(difluoro(phenyl)methyl)-4-methylbenzenesulfonamide (29)**

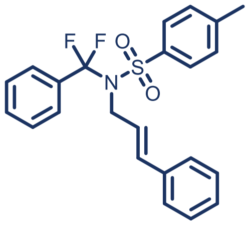

Prepared according to **General Procedure D**, cinnamyl bromide (39.4 mg, 0.2 mmol, 1 equiv.), tetrabutylammonium iodide (83.26 mg, 0.22 mmol, 1.1 equiv.) and *N*-tosylbenzimidoyl chloride solution (**S-10**) (8 mL, 0.1 M, 0.8 mmol, 4 equiv.). The crude was purified using flash column chromatography (100% *n*-pentane to 10% AcOEt in *n*-pentane), affording compound **29** (44 mg, 53%) as a clear oil.

^{1}H NMR (300 MHz, $CDCl_3$) δ 7.69 (d, J = 7.8 Hz, 2H), 7.56 – 7.21 (m, 12H), 6.43 – 6.26 (m, 1H), 6.26 – 6.11 (m, 1H), 4.24 (d J = 6.6 Hz, 2H), 2.44 (s, 3H).

^{13}C NMR (75 MHz, $CDCl_3$) δ 144.0, 138.0, 136.4, 134.0 (t, J = 30.6 Hz), 133.8, 130.9 (t, J = 1.9 Hz), 129.5, 128.6, 128.7, 127.9, 127.7, 126.8 (t, J = 4.8 Hz), 126.6, 124.8, 120.7 (t, J = 254.8 Hz), 48.9, 21.6.

^{19}F NMR (282 MHz, $CDCl_3$) δ -69.81 (s, 2F).

HRMS (FD+) (m/z): $[M]^+$ calculated for $C_{23}H_{21}F_2NO_2S$, 413.1261; found: 413.1255.

***N*-cinnamyl-*N*-(difluoro(4-(trifluoromethoxy)phenyl)methyl)-4-methylbenzenesulfonamide (30)**

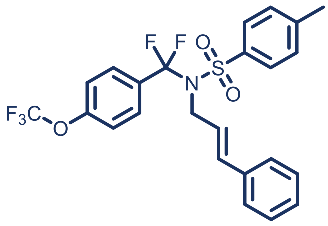

Prepared according to **General Procedure D**, cinnamyl bromide (39.4 mg, 0.2 mmol, 1 equiv.), tetrabutylammonium iodide (83.26 mg, 0.22 mmol, 1.1 equiv.) and *N*-tosyl-4-(trifluoromethoxy)benzimidoyl chloride (**SI-11**) solution (8 mL, 0.1 M, 0.8 mmol, 4 equiv.). The crude was purified using flash column chromatography (100% *n*-pentane to 10% AcOEt in *n*-pentane), affording compound **30** (71 mg, 71%) as a clear oil.

^{1}H NMR (300 MHz, $CDCl_3$) δ 7.65 – 7.53 (m, 4H), 7.42 – 7.27 (m, 5H), 7.27 – 7.15 (m, 4H), 6.48 (m, 1H), 6.23 (m, 1H), 4.44 – 4.28 (d, J = 6.7 Hz, 2H), 2.42 (s, 3H).

^{13}C NMR (75 MHz, $CDCl_3$) δ 150.9 (q, J = 1.9 Hz), 144.2, 137.6, 136.2, 134.11, 132.4 (t, J = 31.3 Hz), 129.5, 129.0 (t, J = 4.8 Hz), 128.6, 128.1, 127.60, 126.6, 124.6, 120.3, 120.3 (q, J = 258.4 Hz) 120.0 (t, J = 254.8 Hz), 48.6, 21.5.

^{19}F NMR (282 MHz, $CDCl_3$) δ -57.73(s, 3F), -69.18 (s, 2F).

HRMS (FD+) (m/z): $[M]^+$ calculated for $C_{24}H_{20}F_5NO_3S$, 497.1084; found: 497.1068.

N-cinnamyl-N-((4-cyanophenyl)difluoromethyl)-4-methylbenzenesulfonamide (31)

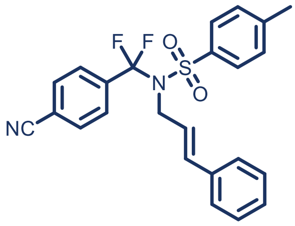

Prepared according to **General Procedure D**, cinnamyl bromide (39.4 mg, 0.2 mmol, 1 equiv.), tetrabutylammonium iodide (83.26 mg, 0.22 mmol, 1.1 equiv.) and 4-cyano-*N*-tosylbenzimidoyl chloride (**SI-12**) solution (8 mL, 0.1 M, 0.8 mmol, 4 equiv.). The crude was purified using flash column chromatography (100% *n*-pentane to 10% AcOEt in *n*-pentane), affording compound **31** (70 mg, 80%) as a clear oil.

^{1}H NMR (300 MHz, $CDCl_3$) δ 7.75 – 7.54 (m, 6H), 7.42 – 7.21 (m, 7H), 6.53 – 6.40 (m, 1H), 6.26 – 6.10 (m, 1H), 4.34 – 4.21 (m, 2H), 2.44 (s, 3H).

^{13}C NMR (75 MHz, $CDCl_3$) δ 144.6, 138.8 (t, J = 31.7 Hz), 137.1, 136.1, 134.3, 132.0, 129.7, 128.7, 128.2, 127.8, 127.7 (t, J = 4.9 Hz), 126.6, 124.2, 119.6 (t, J = 255.8 Hz), 117.8, 114.8 (t, J = 2.0 Hz), 48.6, 21.6.

^{19}F NMR (282 MHz, $CDCl_3$) δ -70.25 (s, 2F).

HRMS (FD+) (m/z): $[M]^+$ calculated for $C_{24}H_{20}F_2N_2O_2S$, 438.1214; found: 438.1229.

Methyl 4-(N-cinnamyl-N-tosylfluorocarbonyl)benzoate (32)

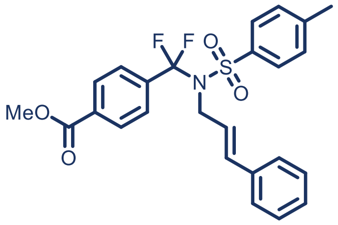

Prepared according to **General Procedure D**, cinnamyl bromide (39.4 mg, 0.2 mmol, 1 equiv.), tetrabutylammonium iodide (83.26 mg, 0.22 mmol, 1.1 equiv.) and methyl-4-(chloro(tosylimino)methyl)benzoate (**SI-13**) solution (8 mL, 0.1 M, 0.8 mmol, 4 equiv.). The crude was purified using flash column chromatography (100% *n*-pentane to 10% AcOEt in *n*-pentane), affording compound **32** (66 mg, 70%) as a clear oil.

^{1}H NMR (300 MHz, $CDCl_3$) δ 8.02 (d, J = 8.6 Hz, 2H), 7.68 – 7.53 (m, 4H), 7.39 – 7.17 (m, 7H), 6.45 – 6.33 (m, 1H), 6.23 – 6.07 (m, 1H), 4.30 – 4.20 (m, 2H), 3.94 (s, 3H), 2.40 (s, 3H).

^{13}C NMR (75 MHz, $CDCl_3$) δ 166.1, 144.3, 138.3 (t, J = 30.9 Hz), 137.6, 136.2, 134.0, 132.4 (t, J = 1.5 Hz), 129.6, 129.5, 128.6, 128.0, 127.8, 127.0 (t, J = 4.7 Hz), 126.6, 124.5, 120.2 (t, J = 255.5 Hz), 52.5, 48.8, 21.6.

^{19}F NMR (282 MHz, $CDCl_3$) δ -70.04 (s, 2F).

HRMS (FD+) (m/z): $[M]^+$ calculated for $C_{25}H_{23}F_2NO_4S$, 471.1316; found: 471.1321.

N-cinnamyl-N-(difluoro(4-(methylsulfonyl)phenyl)methyl)-4-methylbenzenesulfonamide (33)

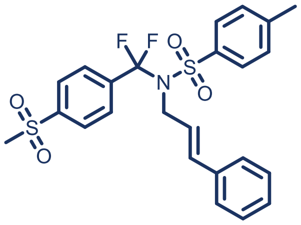

Prepared according to **General Procedure D**, cinnamyl bromide (39.4 mg, 0.2 mmol, 1 equiv.), tetrabutylammonium iodide (83.26 mg, 0.22 mmol, 1.1 equiv.) and 4-(methylsulfonyl)-*N*-tosylbenzimidoyl chloride (**SI-14**) solution (8 mL, 0.1 M, 0.8 mmol, 4 equiv.). The crude was purified using flash column chromatography (100% *n*-pentane to 10% AcOEt in *n*-pentane), affording compound **33** (56 mg, 57%) as a clear oil.

^{1}H NMR (300 MHz, $CDCl_3$) δ 7.91 (d, J = 8.5 Hz, 2H), 7.72 (d, J = 8.6 Hz, 2H), 7.60 (d, J = 8.3 Hz, 2H), 7.38 – 7.18 (m, 7H), 6.47 – 6.35 (m, 1H), 6.23 – 6.07 (m, 1H), 4.34 – 4.24 (m, 2H), 2.99 (s, 3H), 2.41 (s, 3H).

^{13}C NMR (75 MHz, $CDCl_3$) δ 144.6, 142.8, 139.7 (t, J = 31.6 Hz), 137.1, 136.1, 134.2, 129.7, 128.7, 128.2, 128.0 (t, J = 4.6 Hz), 127.7, 127.4, 126.6, 124.3, 119.6 (t, J = 256.0 Hz), 48.6, 44.3, 21.6.

^{19}F NMR (282 MHz, CDCl$_3$) δ -70.37 (s, 2F).

HRMS (FD+) (m/z): $[M]^+$ calculated for $C_{24}H_{23}F_2NO_4S_2$, 491.1037; found: 491.1049.

_N_-((5-bromothiophen-3-yl)difluoromethyl)-_N_-cinnamyl-4-methylbenzenesulfonamide (34)

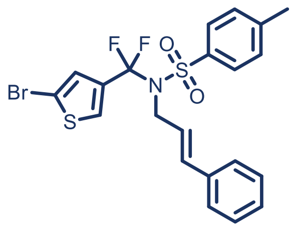

Prepared according to **General Procedure D**, cinnamyl bromide (39.4 mg, 0.2 mmol, 1 equiv.), tetrabutylammonium iodide (83.26 mg, 0.22 mmol, 1.1 equiv.) and 5-bromo-*N*-tosylthiophene-3-carbimidoyl chloride (**SI-15**) solution (8 mL, 0.1 M, 0.8 mmol, 4 equiv.). The crude was purified using flash column chromatography (100% *n*-pentane to 10% AcOEt in *n*-pentane), affording compound **34** (59.3 mg, 60%) as a white solid.

^{1}H NMR (300 MHz, CDCl$_3$) δ 7.64 – 7.55 (m, 2H), 7.50 – 7.44 (m, 1H), 7.40 – 7.18 (m, 7H), 6.85 (d, J = 1.5 Hz, 1H), 6.56 – 6.44 (m, 1H), 6.29 – 6.13 (m, 1H), 4.41 – 4.22 (m, 2H), 2.41 (s, 3H).

^{13}C NMR (75 MHz, CDCl$_3$) δ 144.2, 137.7, 136.3, 135.3 (t, J = 34.7 Hz), 134.1, 129.6, 128.9 (t, J = 5.2 Hz), 128.7, 128.6 (t, J = 3.1 Hz), 128.1, 127.6, 126.7, 124.8, 117.5 (t, J = 252.9 Hz), 113.3, 48.5, 21.6.

^{19}F NMR (282 MHz, CDCl$_3$) δ -66.61 (s, 2F).

HRMS (FD+) (m/z): $[M]^+$ calculated for $C_{21}H_{18}BrF_2NO_2S_2$, 496.9930; found: 496.9933.

_N_-cinnamyl-_N_-((4-(N,N-dipropylsulfamoyl)phenyl)difluoromethyl)-4-methylbenzenesulfonamide (35)

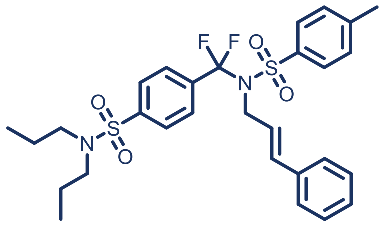

Prepared according to **General Procedure D**, cinnamyl bromide (39.4 mg, 0.2 mmol, 1 equiv.), tetrabutylammonium iodide (83.26 mg, 0.22 mmol, 1.1 equiv.) and 4-(*N*,*N*-dipropylsulfamoyl)-N-tosylbenzimidoyl chloride (**SI-16**) solution (8 mL, 0.1 M, 0.8 mmol, 4 equiv.). The crude was purified using flash column chromatography (100% *n*-pentane to 10% AcOEt in *n*-pentane), affording compound **35** (82 mg, 71%) as a clear oil.

^{1}H NMR (300 MHz, CDCl$_3$) δ 7.81 – 7.72 (m, 2H), 7.69 – 7.54 (m, 4H), 7.37 – 7.18 (m, 7H), 6.48 – 6.36 (m, 1H), 6.24 – 6.08 (m, 1H), 4.32 – 4.22 (m, 2H), 3.10 – 2.97 (m, 4H), 2.41 (s, 3H), 1.54 (h, J = 7.4 Hz, 4H), 0.86 (t, J = 7.4 Hz, 6H).

^{13}C NMR (75 MHz, CDCl$_3$) δ 144.5, 142.6, 137.8 (t, J = 31.3 Hz), 137.3, 136.1, 134.1, 129.6, 128.7, 128.1, 127.7, 127.7 (t, J = 4.6 Hz), 126.8, 126.6, 124.4, 119.8 (t, J = 255.6 Hz), 50.1, 48.6, 22.1, 21.6, 11.2.

^{19}F NMR (282 MHz, CDCl$_3$) δ -69.98 (s, 2F).

HRMS (FD+) (m/z): $[M]^+$ calculated for $C_{24}H_{20}F_5NO_3S$, 576.1928; found: 576.1951.

4.4 Scope of Sulfonamides

4-Chloro-*N*-cinnamyl-*N*-(difluoro(4-(trifluoromethyl)phenyl)methyl)benzenesulfonamide (36)

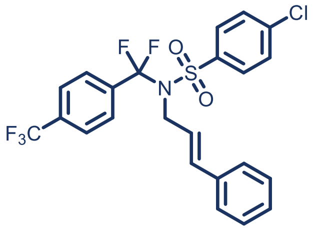

Prepared according to **General Procedure D**, cinnamyl bromide (39.4 mg, 0.2 mmol, 1 equiv.), tetrabutylammonium iodide (83.26 mg, 0.22 mmol, 1.1 equiv.) and *N*-((4-chlorophenyl)sulfonyl)-4-(trifluoromethyl)benzimidoyl chloride (**SI-17**) solution (8 mL, 0.1 M, 0.8 mmol, 4 equiv.). The crude was purified using flash column chromatography (100% *n*-pentane to 10% AcOEt in *n*-pentane), affording compound **36** (82 mg, 82%) as a clear oil.

^{1}H NMR (300 MHz, $CDCl_3$) δ 7.75 – 7.57 (m, 6H), 7.46 – 7.32 (m, 7H), 6.54 – 6.41 (m, 1H), 6.29 – 6.12 (m, 1H), 4.33 (d, J = 6.8, 2H).

^{13}C NMR (75 MHz, $CDCl_3$) δ 139.9, 138.9, 137.4 (t, J = 31.3 Hz), 135.9, 134.7, 133.1 (q, J = 32.8 Hz), 129.3, 129.1, 128.7, 128.3, 127.5 (t, J = 4.8 Hz), 126.6, 125.4 (q, J = 3.7 Hz), 123.9, 123.4 (q, J = 272.8 Hz), 119.7 (t, J = 256.1 Hz), 53.5, 48.8.

^{19}F NMR (282 MHz, $CDCl_3$) δ -63.01 (s, 3F), -70.35 (s, 2F).

HRMS (FD+) (m/z): $[M]^+$ calculated for $C_{23}H_{17}ClF_5NO_2S$, 501.0589; found: 501.0583.

***N*-Cinnamyl-*N*-(difluoro(4-(trifluoromethyl)phenyl)methyl)-4-fluorobenzenesulfonamide (37)**

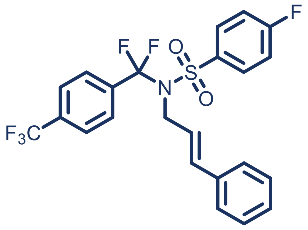

Prepared according to **General Procedure D**, cinnamyl bromide (39.4 mg, 0.2 mmol, 1 equiv.), tetrabutylammonium iodide (83.26 mg, 0.22 mmol, 1.1 equiv.) and *N*-((4-fluorophenyl)sulfonyl)-4-(trifluoromethyl)benzimidoyl chloride (**SI-18**) solution (8 mL, 0.1 M, 0.8 mmol, 4 equiv.). The crude was purified using flash column chromatography (100% *n*-pentane to 10% AcOEt in *n*-pentane), affording compound **37** (69.7 mg, 72%) as a clear oil.

^{1}H NMR (300 MHz, $CDCl_3$) δ 7.78 – 7.69 (m, 2H), 7.68 – 7.59 (m, 4H), 7.40 – 7.26 (m, 5H), 7.16 – 7.03 (m, 2H), 6.45 (d, J = 15.9 Hz, 1H), 6.25 – 6.09 (m, 1H), 4.36 – 4.26 (m, 2H).

^{13}C NMR (75 MHz, $CDCl_3$) δ 165.4 (d, J = 256.7 Hz), 137.5 (t, J = 31.3 Hz), 136.5 (d, J = 3.3 Hz), 136.0, 134.7, 133.2 (q, J = 32.5 Hz), 130.6 (d, J = 9.5 Hz), 128.8, 128.3, 127.6 (t, J = 4.7 Hz), 126.6, 125.4 (q, J = 3.8 Hz), 124.1, 123.5 (q, J = 272.8 Hz), 119.9 (t, J = 255.5 Hz), 116.3 (d, J = 22.8 Hz), 48.9.

^{19}F NMR (282 MHz, $CDCl_3$) δ -63.03 (s, 3F), -70.37 (s, 2F), -103.71 (s, 1F).

HRMS (FD+) (m/z): $[M]^+$ calculated for $C_{23}H_{17}F_6NO_2S$, 485.0884; found: 485.0878.

***N*-Cinnamyl-*N*-(difluoro(4-(trifluoromethyl)phenyl)methyl)-4-methoxybenzenesulfonamide (38)**

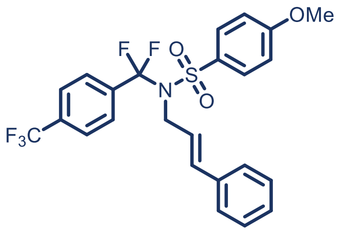

Prepared according to **General Procedure D**, cinnamyl bromide (39.4 mg, 0.2 mmol, 1 equiv.), tetrabutylammonium iodide (83.26 mg, 0.22 mmol, 1.1 equiv.) and *N*-((4-methoxyphenyl)sulfonyl)-4-(trifluoromethyl)benzimidoyl chloride (**SI-19**) solution (8 mL, 0.1 M, 0.8 mmol, 4 equiv.). The crude was purified using flash column chromatography (100% *n*-pentane to 10% AcOEt in *n*-pentane), affording compound **38** (44 mg, 44%) as a clear oil.

^{1}H NMR (300 MHz, $CDCl_3$) δ 7.69 – 7.57 (m, 6H), 7.37 – 7.25 (m, 5H), 6.88 (d, J = 9.0 Hz, 2H), 6.53 – 6.38 (m, 1H), 6.29 – 6.17 (m, 1H), 4.33 (d, J = 6.6 Hz, 2H), 3.86 (s, 3H).

^{13}C NMR (75 MHz, $CDCl_3$) δ 163.3, 137.7 (t, J = 31.5 Hz), 136.2, 134.1, 132.8 (q, J = 32.7 Hz), 131.8, 129.9, 128.7, 128.1, 127.6 (t, J = 4.8 Hz), 126.6, 125.2 (q, J = 3.8 Hz), 124.5, 123.1 (q, J = 273.7 Hz), 119.9 (t, J = 255.2 Hz), 114.1, 55.6, 48.5.

^{19}F NMR (282 MHz, $CDCl_3$) δ -62.99 (s, 3F), -70.22 (s, 2F).

HRMS (FD+) (m/z): $[M]^+$ calculated for $C_{24}H_{20}F_5NO_3S$, 497.1084; found: 497.1078.

N-Cinnamyl-*N*-(difluoro(4-(trifluoromethyl)phenyl)methyl)-4-nitrobenzenesulfonamide (39)

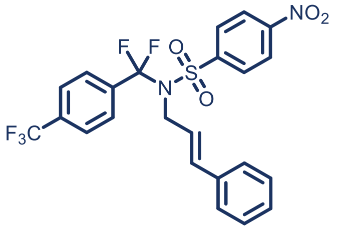

Prepared according to **General Procedure D**, cinnamyl bromide (39.4 mg, 0.2 mmol, 1 equiv.), tetrabutylammonium iodide (83.26 mg, 0.22 mmol, 1.1 equiv.) and *N*-((4-nitrophenyl)sulfonyl)-4-(trifluoromethyl)benzimidoyl chloride (**SI-20**) solution (8 mL, 0.1 M, 0.8 mmol, 4 equiv.). The crude was purified using flash column chromatography (100% *n*-pentane to 10% AcOEt in *n*-pentane), affording compound **39** (39 mg, 38%) as a clear oil.

^{1}H NMR (300 MHz, $CDCl_3$) δ 8.29 (d, J = 9.1 Hz, 2H), 7.97 (d, J = 9.1 Hz, 2H), 7.73 – 7.64 (m, 4H), 7.41 – 7.25 (m, 5H), 6.55 – 6.44 (m, 1H), 6.25 – 6.05 (m, 1H), 4.32 (d, J = 6.8, 2H).

^{13}C NMR (75 MHz, $CDCl_3$) δ 150.2, 146.0, 137.0 (t, J = 32.7 Hz), 135.6, 135.3, 133.4 (q, J = 31.6 Hz), 129.0, 128.8, 128.5, 127.3 (t, J = 4.6 Hz), 126.5, 125.5 (q, J = 4.0 Hz), 124.1, 123.2, 123.3 (q, J = 272.3 Hz), 119.7 (t, J = 256.9 Hz), 49.2.

^{19}F NMR (282 MHz, $CDCl_3$) δ -63.08 (s, 3F), -70.46 (s, 2F).

HRMS (FD+) (m/z): $[M]^+$ calculated for $C_{23}H_{17}F_5N_2O_4S$, 512.0829; found: 512.0839.

N-Cinnamyl-*N*-(difluoro(4-(trifluoromethyl)phenyl)methyl)-1,4-dimethyl-1H-pyrazole-5-sulfonamide (40)

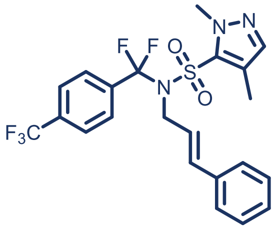

Prepared according to **General Procedure D**, cinnamyl bromide (39.4 mg, 0.2 mmol, 1 equiv.), tetrabutylammonium iodide (83.26 mg, 0.22 mmol, 1.1 equiv.) and *N*-((1,4-dimethyl-1H-pyrazol-5-yl)sulfonyl)-4-(trifluoromethyl)benzimidoyl chloride (**SI-21**) solution (8 mL, 0.1 M, 0.8 mmol, 4 equiv.). The crude was purified using flash column chromatography (100% *n*-pentane to 10% AcOEt in *n*-pentane), affording compound **40** (70 mg, 72%) as a clear oil.

^{1}H NMR (300 MHz, $CDCl_3$) δ 7.72 – 7.61 (m, 4H), 7.38 – 7.25 (m, 6H), 6.52 – 6.40 (m, 1H), 6.33 – 6.20 (m, 1H), 4.35 (d, J = 6.6 Hz, 2H), 3.70 (s, 3H), 2.39 (s, 3H).

^{13}C NMR (75 MHz, $CDCl_3$) δ 147.8, 137.6 (t, J = 31.1 Hz), 136.1, 134.5, 134.1, 132.9 (q, J = 32.8 Hz), 128.7, 128.1, 127.7 (t, J = 4.9 Hz), 126.5, 125.1 (q, J = 3.8 Hz), 124.7, 123.4 (q, J = 272.6 Hz), 120.0 (t, J = 255.0 Hz), 119.9, 48.6, 39.0, 12.5.

^{19}F NMR (282 MHz, $CDCl_3$) δ -62.81 (s, 3F), -70.76 (s, 2F).

HRMS (FD+) (m/z): $[M]^+$ calculated for $C_{22}H_{20}F_5N_3O_2S$, 485.1196; found: 485.1181.

N-Cinnamyl-N-(difluoro(4-(trifluoromethyl)phenyl)methyl)thiophene-2-sulfonamide (41)

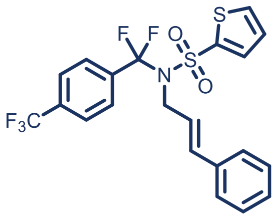

Prepared according to **General Procedure D**, cinnamyl bromide (39.4 mg, 0.2 mmol, 1 equiv.), tetrabutylammonium iodide (83.26 mg, 0.22 mmol, 1.1 equiv.) and *N*-(thiophen-2-ylsulfonyl)-4-(trifluoromethyl)benzimidoyl chloride (**SI-22**) solution (8 mL, 0.1 M, 0.8 mmol, 4 equiv.). The crude was purified using flash column chromatography (100% *n*-pentane to 10% AcOEt in *n*-pentane), affording compound **41** (91 mg, 96%) as a clear oil.

^{1}H NMR (300 MHz, $CDCl_3$) δ 7.74 – 7.62 (m, 4H), 7.61 (dd, *J* = 5.0, 1.3 Hz, 1H), 7.49 – 7.41 (m, 1H), 7.39 – 7.27 (m, 5H), 7.00 (dd, *J* = 5.0, 3.9 Hz, 1H), 6.54 – 6.41 (m, 1H), 6.31 – 6.15 (m, 1H), 4.33 (d, *J* = 6.5 Hz, 2H).

^{13}C NMR (75 MHz, $CDCl_3$) δ 140.8, 137.7 (t, *J* = 31.9 Hz), 136.1, 134.5, 134.0, 133.3, 132.9 (q, *J* = 32.7 Hz), 128. 7, 128.2, 127.4 (t, *J* = 4.8 Hz), 127.2, 126.6, 125.3 (q, *J* = 3.7 Hz), 123.9, 123.6 (d, *J* = 272.6 Hz), 119.9 (t, *J* = 256.3 Hz), 49.3.

^{19}F NMR (282 MHz, $CDCl_3$) δ -62.95 (s, 3F), -70.32 (s, 2F).

HRMS (FD+) (m/z): $[M]^+$ calculated for $C_{21}H_{16}F_5NO_2S_2$, 473.0543; found: 473.0528.

5-Chloro-N-cinnamyl-N-(difluoro(4-(trifluoromethyl)phenyl)methyl)thiophene-2-sulfonamide (42)

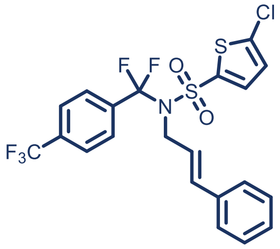

Prepared according to **General Procedure D**, cinnamyl bromide (39.4 mg, 0.2 mmol, 1 equiv.), tetrabutylammonium iodide (83.26 mg, 0.22 mmol, 1.1 equiv.) and *N*-((5-chlorothiophen-2-yl)sulfonyl)-4-(trifluoromethyl)benzimidoyl chloride (**SI-23**) solution (8 mL, 0.1 M, 0.8 mmol, 4 equiv.). The crude was purified using flash column chromatography (100% *n*-pentane to 10% AcOEt in *n*-pentane), affording compound **42** (72 mg, 71%) as a clear oil.

^{1}H NMR (300 MHz, $CDCl_3$) δ 7.84 – 7.68 (m, 4H), 7.43 – 7.25 (m, 6H), 6.86 (d, *J* = 4.1 Hz, 1H), 6.53 – 6.45 (m, 1H), 6.21 (m, 1H), 4.31 (d, *J* = 5.5 Hz, 1H).

^{13}C NMR (75 MHz, $CDCl_3$) δ 139.0, 138.6, 137.5 (t, *J* = 30.8 Hz), 135.9, 134.9, 133.3, 132.7 (q, *J* = 32.7 Hz), 128.7, 128.3, 127.3 (t, *J* = 4.8 Hz), 126.6, 126.5, 125.4 (q, *J* = 3.8 Hz), 123.5, 124.3 (q, *J* = 272.7 Hz), 119.8 (t, *J* = 256.8 Hz), 49.2.

^{19}F NMR (282 MHz, $CDCl_3$) δ -63.00 (s, 3F), -70.40 (s, 2F).

HRMS (FD+) (m/z): $[M]^+$ calculated for $C_{21}H_{15}ClF_5NO_2S_2$, 507.0153; found: 507.0180.

N-Cinnamyl-N-(difluoro(4-(trifluoromethyl)phenyl)methyl)methanesulfonamide (43)

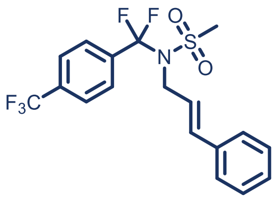

Prepared according to **General Procedure D**, cinnamyl bromide (39.4 mg, 0.2 mmol, 1 equiv.), tetrabutylammonium iodide (83.26 mg, 0.22 mmol, 1.1 equiv.) and *N*-(methylsulfonyl)-4-(trifluoromethyl)benzimidoyl chloride (**SI-24**) solution (8 mL, 0.1 M, 0.8 mmol, 4 equiv.). The crude was purified using flash column chromatography (100% *n*-pentane to 10% AcOEt in *n*-pentane), affording compound **43** (65 mg, 80%) as a clear oil.

^{1}H NMR (300 MHz, $CDCl_3$) δ 7.91 – 7.71 (m, 4H), 7.46 – 7.26 (m, 5H), 6.55 – 6.44 (m, 1H), 6.31 – 6.17 (m, 1H), 4.23 (d, *J* = 6.8, 2H), 3.15 (s, 3H).

^{13}C NMR (75 MHz, $CDCl_3$) δ 137.9 (t, J = 32.2 Hz), 135.9, 135.0, 133.2 (q, J = 32.8 Hz), 128.7, 128.4, 127.1 (t, J = 4.6 Hz), 126.6, 125.7 (q, J = 3.7 Hz), 123.6 (q, J = 274.3 Hz), 123.5, 120.2 (t, J = 255.9 Hz), 48.9, 43.6.

^{19}F NMR (282 MHz, $CDCl_3$) δ -62.98 (s, 3F), -70.36 (s, 2F).

HRMS (FD+) (m/z): $[M]^+$ calculated for $C_{18}H_{16}F_5NO_2S$, 405.0822; found: 405.0618.

_N_-Cinnamyl-_N_-(difluoro(4-(trifluoromethyl)phenyl)methyl)-1,1,1-trifluoromethanesulfonamide (44)

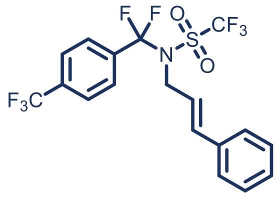

Prepared according to **General Procedure D**, cinnamyl bromide (39.4 mg, 0.2 mmol, 1 equiv.), tetrabutylammonium iodide (83.26 mg, 0.22 mmol, 1.1 equiv.) and 4-(trifluoromethyl)-*N*-((trifluoromethyl)sulfonyl)benzimidoyl chloride (**SI-25**) solution (8 mL, 0.1 M, 0.8 mmol, 4 equiv.). The crude was purified using flash column chromatography (100% *n*-pentane to 10% AcOEt in *n*-pentane), affording compound **44** (60 mg, 65%) as a clear oil.

^{1}H NMR (300 MHz, $CDCl_3$) δ 7.83 – 7.72 (m, 4H), 7.39 – 7.33 (m, 5H), 6.45 – 6.31 (m, 1H), 6.21 – 6.06 (m, 1H), 4.31 (d, J = 6.8, 2H).

^{13}C NMR (75 MHz, $CDCl_3$) δ 136.5 (t, J = 30.4 Hz), 135.8, 135.4, 133.8 (q, J = 34.1 Hz), 128.8, 128.6, 127.1 (t, J = 5.0 Hz), 126.7, 125.8 (q, J = 3.8 Hz), 123.1 (q, J = 273.5 Hz), 122.1, 119.9 (q, J = 323.5 Hz), 119.3 (t, J = 260.2 Hz), 51.3.

^{19}F NMR (282 MHz, $CDCl_3$) δ -63.16 (s, 3F), -69.17 (s, 2F), -75.15 (t, J = 6.9 Hz, 3F).

HRMS (FD+) (m/z): $[M]^+$ calculated for $C_{18}H_{13}F_8NO_2S$, 459.0539; found: 459.0521.

_N_-Cinnamyl-_N_-(difluoro(4-(trifluoromethyl)phenyl)methyl)-4-(5-methyl-3-phenylisoxazol-4-yl)benzenesulfonamide (45)

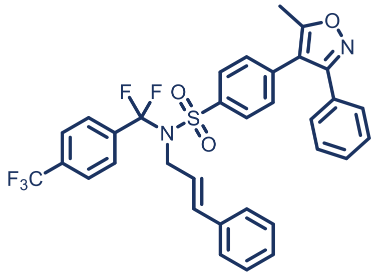

Prepared according to **General Procedure D**, cinnamyl bromide (39.4 mg, 0.2 mmol, 1 equiv.), tetrabutylammonium iodide (83.26 mg, 0.22 mmol, 1.1 equiv.) and *N*-((4-(5-methyl-3-phenylisoxazol-4-yl)phenyl)sulfonyl)-4-(trifluoromethyl)benzimidoyl chloride (**SI-26**) solution (8 mL, 0.1 M, 0.8 mmol, 4 equiv.). The crude was purified using flash column chromatography (100% *n*-pentane to 10% AcOEt in *n*-pentane), affording compound **45** (77 mg, 62%) as a clear oil.

^{1}H NMR (300 MHz, $CDCl_3$) δ 7.75 (d, J = 8.5 Hz, 2H), 7.73 – 7.62 (m, 4H), 7.50 – 7.22 (m, 12H), 6.64 – 6.32 (m, 1H), 6.33 – 6.08 (m, 1H), 4.44 – 4.10 (m, 2H), 2.48 (s, 3H).

^{13}C NMR (75 MHz, $CDCl_3$) δ 167.3, 161.1, 139.5, 137.7 (t, J = 31.3 Hz), 136.0, 135.9, 134.5, 133.1 (q, J = 32.7 Hz), 130.1, 129.8, 128.8, 128.7, 128.5, 128.4, 128.2, 128.1, 127.5 (t, J = 4.8 Hz), 126.5, 125.4 (q, J = 3.8 Hz), 124.0, 123.2 (q, J = 272.5 Hz), 119.8 (t, J = 256.1 Hz), 114.3, 48.9, 11.7.

^{19}F NMR (282 MHz, $CDCl_3$) δ -62.97 (s, 3F), -70.04 (s, 2F).

HRMS (FD+) (m/z): $[M]^+$ calculated for $C_{33}H_{25}F_5N_2O_3S$, 624.1506; found: 624.1507.

N-Cinnamyl-N-(difluoro(4-(trifluoromethyl)phenyl)methyl)-4-(3-(difluoromethyl)-5-(3-fluoro-4-methoxyphenyl)-1H-pyrazol-1-yl)benzenesulfonamide (46)

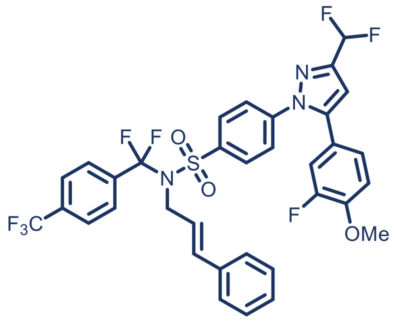

Prepared according to **General Procedure D**, cinnamyl bromide (39.4 mg, 0.2 mmol, 1 equiv.), tetrabutylammonium iodide (83.26 mg, 0.22 mmol, 1.1 equiv.) and *N*-((4-(3-(difluoromethyl)-5-(3-fluoro-4-methoxyphenyl)-1H-pyrazol-1-yl)phenyl)sulfonyl)-4-(trifluoromethyl)benzimidoyl chloride (**SI-27**) solution (8 mL, 0.1 M, 0.8 mmol, 4 equiv.). The crude was purified using flash column chromatography (100% *n*-pentane to 10% AcOEt in *n*-pentane), affording compound **46** (114 mg, 81%) as a clear oil.

^{1}H NMR (300 MHz, $CDCl_3$) δ 7.78 (d, J = 8.7 Hz, 2H), 7.69 – 7.64 (m, 4H), 7.46 – 7.38 (m, 2H), 7.37 – 7.26 (m, 5H), 7.04 – 6.90 (m, 3H), 6.80 (t, J = 54.8 Hz, 1H), 6.74 (s, 1H), 6.43 (d, J = 15.9 Hz, 1H), 6.18 (s, 1H), 4.28 (d, J = 6.0 Hz, 2H), 3.93 (s, 3H).

^{13}C NMR (75 MHz, $CDCl_3$) δ 152.1 (d, J = 248.4 Hz), 148.6 (d, J = 10.5 Hz), 148.4 (t, J = 30.0 Hz), 143.7, 143.0, 139.5, 137.4 (t, J = 31.9 Hz), 135.8, 134.6, 133.1 (q, J = 32.7 Hz), 128.8, 128.7, 128.2, 127.4 (t, J = 4.7 Hz), 126.5, 125.4 (q, J = 3.7 Hz), 125.1 (q, J = 3.6 Hz), 125.0, 123.8, 123.4 (q, J = 272.8 Hz), 121.6 (d, J = 7.0 Hz), 119.7 (t, J = 255.5 Hz), 116.5 (d, J = 19.8 Hz), 113.5 (d, J = 2.3 Hz), 110.9 (t, J = 234.6 Hz), 105.9, 56.2, 48.9.

^{19}F NMR (282 MHz, $CDCl_3$) δ -63.02 (s, 3F), -70.35 (s, 2F), -112.38 (s, 2F), -133.25 (s, 1F).

HRMS (FD+) (m/z): $[M]^+$ calculated for $C_{34}H_{25}F_8N_3O_3S$, 707.1489; found: 707.1488.

4.5 Applications

N-((4-bromo-5-chloro-2-fluorophenyl)difluoromethyl)-N-(4-fluorobenzyl)methanesulfonamide (48)

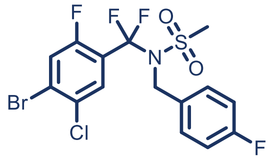

Prepared according to a modified version of **General Procedure D**, using 4-fluorobenzyl bromide (37.8 mg, 0.2 mmol, 1 equiv.), tetrabutylammonium iodide (81.3 mg, 0.22 mmol, 1.1 equiv.) and 4-bromo-5-chloro-2-fluoro-*N*-(methylsulfonyl)benzimidoyl chloride (**47**) (8 mL, 0.1 M, 0.8 mmol, 4 equiv.). The reaction was heated at 60 °C for 18 hours after which the solvent was evaporated and the crude was purified using column chromatography (100% *n*-pentane to 10% AcOEt in *n*-pentane), affording compound **48** (58.1 mg, 63%) as a clear oil.

^{1}H NMR (300 MHz, $CDCl_3$) δ 7.58 (d, *J* = 7.0 Hz, 1H), 7.39 (d, *J* = 9.8 Hz, 1H), 7.34 – 7.27 (m, 2H), 7.09 – 6.96 (m, 2H), 4.67 (s, 2H), 2.93 (s, 3H).

^{13}C NMR (75 MHz, $CDCl_3$) δ 162.6 (d, *J* = 247.2 Hz), 157.3 (dt, *J* = 257.7, 3.3 Hz), 131.6 (d, *J* = 3.2 Hz), 130.5 (d, *J* = 3.9 Hz), 130.2 (d, *J* = 8.2 Hz), 129.5 (td, *J* = 6.0, 2.2 Hz), 126.7 (dt, *J* = 9.7, 1.6 Hz), 122.8 (td, *J* = 32.6, 12.2 Hz), 122.1 (d, *J* = 25.4 Hz), 119.62 (td, *J* = 255.1, 2.2 Hz), 115.6 (d, *J* = 21.6 Hz), 49.1, 43.1.

^{19}F NMR (282 MHz, $CDCl_3$) δ -68.45 (d, *J* = 10.7 Hz, 2F), -113.71 (s, 1F), -114.21 (t, *J* = 10.8 Hz, 1F).

HRMS (FD+) (m/z): $[M]^+$ calculated for $C_{15}H_{11}BrClF_4NO_2S$, 458.9318; found: 458.9298.

N-((4-N-((5-bromothiophen-2-yl)methyl)-2-chloro-N-(difluoro(phenyl)methyl)-benzenesulfonamide (51)

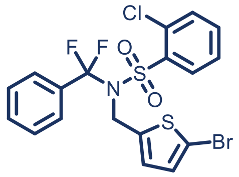

Prepared according to a modified version of **General Procedure D**, using 2-bromo-5-(bromomethyl)thiophene (51.2 mg, 0.2 mmol, 1 equiv.), tetrabutylammonium iodide (81.3 mg, 0.22 mmol, 1.1 equiv.) and *N*-((2-chlorophenyl)sulfonyl)benzimidoyl chloride (**50**) (8 mL, 0.1 M, 0.8 mmol, 4 equiv.). The reaction was heated at 60 °C for 18 hours after which the solvent was evaporated and the crude was purified using column chromatography (100% *n*-pentane to 10% AcOEt in *n*-pentane), affording compound **51** (58.1 mg, 59%) as a white solid.

^{1}H NMR (300 MHz, $CDCl_3$) δ 7.63 (dd, *J* = 8.0, 1.4 Hz, 1H), 7.54 – 7.40 (m, 2H), 7.38 – 7.29 (m, 3H), 7.24 – 7.12 (m, 3H), 6.88 (d, *J* = 3.7 Hz, 1H), 6.76 (d, *J* = 3.7 Hz, 1H), 4.99 (s, 2H).

^{13}C NMR (75 MHz, $CDCl_3$) δ 141.3, 138.4, 134.0, 132.3 (t, *J* = 29.5 Hz), 131.8, 131.6, 131.6, 131.3, 129.5, 128.7, 128.2, 127.1, 127.1 (t, *J* = 5.0 Hz), 120.2 (t, *J* = 255.5 Hz), 113.1, 45.7.

^{19}F NMR (282 MHz, $CDCl_3$) δ -69.89 (s, 2F).

HRMS (FD+) (m/z): $[M]^+$ calculated for $C_{18}H_{13}BrClF_2NO_2S_2$, 490.9227; found: 490.9214.

bromophenyl)difluoromethyl)-4-methyl-N-((5-(trifluoromethyl)furan-2-yl)methyl)-benzenesulfonamide (54)

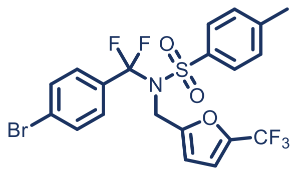

Prepared according to a modified version of **General Procedure D**, using 2-(bromomethyl)-5-(trifluoromethyl)furan (45.8 mg, 0.2 mmol, 1 equiv.), tetrabutylammonium iodide (81.3 mg, 0.22 mmol, 1.1 equiv.) and 4-bromo-*N*-tosylbenzimidoyl chloride solution (**53**) (8 mL, 0.1 M, 0.8 mmol, 4 equiv.). The reaction was heated at 60 °C for 18 hours and then at 80 °C for

8 hours, after which the solvent was evaporated and the crude was purified using column chromatography (100% *n*-pentane to 10% AcOEt in *n*-pentane), affording compound **54** (35.8 mg, 34%) as a clear oil.

^{1}H NMR (300 MHz, $CDCl_3$) δ 7.44 – 7.34 (m, 4H), 7.25 – 7.14 (m, 4H), 6.77 – 6.68 (m, 1H), 6.41 – 6.33 (m, 1H), 4.78 (s, 2H), 2.41 (s, 3H).

^{13}C NMR (75 MHz, $CDCl_3$) δ 153.3, 144.5, 141.5 (q, *J* = 42.8 Hz), 137.2, 132.2 (t, *J* = 30.8 Hz), 131.4, 129.5, 128.7 (t, *J* = 4.8 Hz), 127.5, 125.8, 120.0 (t, *J* = 255.7 Hz), 119.0 (q, *J* = 266.9 Hz), 112.6 (q, *J* = 2.8 Hz), 110.1, 41.8, 21.6.

^{19}F NMR (282 MHz, $CDCl_3$) δ -64.10 (s, 3F), -70.54 (s, 2F).

HRMS (FD+) (m/z): $[M]^+$ calculated for $C_{20}H_{15}BrF_5NO_3S$, 522.9876; found: 522.9890.

Ethyl-5-(((*N*-((4-bromophenyl)difluoromethyl)-4-methylphenyl)sulfonamido)methyl)furan-2-carboxylate (55)

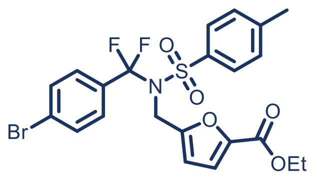

Prepared according to a modified version of **General Procedure D**, using ethyl 5-(bromomethyl)furan-2-carboxylate (46.6 mg, 0.2 mmol, 1 equiv.), tetrabutylammonium iodide (81.3 mg, 0.22 mmol, 1.1 equiv.) and 4-bromo-*N*-tosylbenzimidoyl chloride (**53**) solution (8 mL, 0.1 M, 0.8 mmol, 4 equiv.). The reaction was heated at 60 °C for 18 hours and then at 80 °C for 8 hours, after which the solvent was evaporated and the crude was purified using column chromatography (100% *n*-pentane to 10% AcOEt in *n*-pentane), affording compound **55** (30.3 mg, 30%) as a slightly yellow oil.

^{1}H NMR (400 MHz, $CDCl_3$) δ 7.47 (d, *J* = 8.2 Hz, 2H), 7.40 (d, *J* = 8.4 Hz, 2H), 7.27 (d, *J* = 7.8 Hz, 2H), 7.19 (d, *J* = 8.1 Hz, 2H), 7.09 (d, *J* = 3.4 Hz, 1H), 6.42 (d, *J* = 3.4 Hz, 1H), 4.79 (s, 2H), 4.37 (q, *J* = 7.2 Hz, 2H), 2.42 (s, 3H), 1.39 (t, *J* = 7.2 Hz, 3H).

^{13}C NMR (75 MHz, $CDCl_3$) δ 158.6, 154.6, 144.4, 144.3, 137.2, 132.1 (t, *J* = 30.7 Hz), 131.4, 129.5, 128.8 (t, *J* = 4.8 Hz), 127.7, 125.8, 120.1 (t, *J* = 255.3 Hz), 118.8, 110.9, 61.1, 42.3, 21.7, 14.5.

^{19}F NMR (282 MHz, $CDCl_3$) δ -70.51.

HRMS (FD+) (m/z): $[M]^+$ calculated for $C_{22}H_{20}BrF_2NO_5S$, 527.0213; found: 527.0129.

N-((4-bromophenyl)difluoromethyl)-*N*-(2-fluorobenzyl)-4-methylbenzenesulfonamide (56)

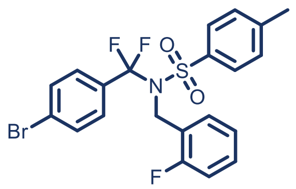

Prepared according to a modified version of **General Procedure D**, using 2-fluorobenzyl bromide (37.8 mg, 0.2 mmol, 1 equiv.), tetrabutylammonium iodide (81.3 mg, 0.22 mmol, 1.1 equiv.) and 4-bromo-*N*-tosylbenzimidoyl chloride (**53**) solution (8 mL, 0.1 M, 0.8 mmol, 4 equiv.). The reaction was heated at 60 °C for 18 hours and then at 80 °C for 8 hours, after which the solvent was evaporated and the crude was purified using column chromatography (100% *n*-pentane to 10% AcOEt in *n*-pentane), affording compound **56** (20.9 mg, 22%) as a clear oil.

^{1}H NMR (400 MHz, $CDCl_3$) δ 7.61 (t, *J* = 7.3 Hz, 1H), 7.50 (d, *J* = 8.3 Hz, 2H), 7.39 (d, *J* = 8.4 Hz, 2H), 7.28 – 7.21 (m, 5H), 7.15 (t, *J* = 7.5 Hz, 1H), 7.00 – 6.91 (m, 1H), 4.77 (s, 2H), 2.44 (s, 3H).

^{13}C NMR (75 MHz, $CDCl_3$) δ 160.2 (d, *J* = 247.0 Hz), 144.5, 137.1, 132.7 (t, *J* = 30.9 Hz), 131.3, 130.1 (d, *J* = 3.4 Hz), 129.6, 129.4 (d, *J* = 8.2 Hz), 128.6 (t, *J* = 4.9 Hz), 127.8, 125.5, 124.3 (d, *J* = 3.6 Hz), 124.2 (d, *J* = 13.5 Hz), 120.3 (t, *J* = 255.4 Hz), 115.3 (d, *J* = 21.6 Hz), 43.1 (d, *J* = 5.2 Hz), 21.7.

^{19}F NMR (282 MHz, $CDCl_3$) δ -70.25 (d, J = 3.2 Hz, 2F), -118.67 (t, J = 3.5 Hz, 1F).

5-Bromo-*N*-cinnamyl-N-((2,4-dichlorophenyl)difluoromethyl)thiophene-2-sulfonamide (61)

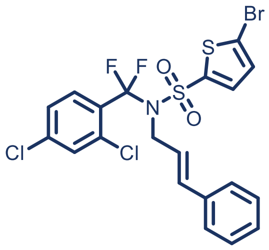

Prepared according to **General Procedure D**, cinnamyl bromide (39.4 mg, 0.2 mmol, 1 equiv.), tetrabutylammonium iodide (83.26 mg, 0.22 mmol, 1.1 equiv.) and *N*-((5-bromothiophen-2-yl)sulfonyl)-2,4-dichlorobenzimidoyl chloride (**SI-28**) solution (8 mL, 0.1 M, 0.8 mmol, 4 equiv.). The crude was purified using flash column chromatography (100% *n*-pentane to 10% AcOEt in *n*-pentane), affording compound **61** (78 mg, 71%) as a clear oil.

^1H NMR ^1H NMR (300 MHz, $CDCl_3$) δ 7.73 (d, J = 9.2 Hz, 1H), 7.43 – 7.29 (m, 7H), 7.14 (d, J = 4.0 Hz, 1H), 7.01 (d, J = 4.0 Hz, 1H), 6.52 – 6.39 (m, 1H), 6.26 (m, 1H), 4.35 (d, J = 6.6 Hz, 2H).

^{13}C NMR (75 MHz, $CDCl_3$) δ 141.0, 138.0, 136.1, 134.2, 133.9 (t, J = 3.4 Hz), 133.7, 130.9, 130.7 (t, J = 6.8 Hz), 130.1, 129.9 (t, J = 30.3 Hz), 128.7, 128.1, 126.9, 126.6, 123.8, 121.5, 118.7 (t, J = 256.6 Hz), 49.4.

^{19}F NMR (282 MHz, $CDCl_3$) δ -68.58 (s, 2F).

HRMS (FD+) (m/z): $[M]^+$ calculated for $C_{20}H_{14}BrCl_2F_2NO_2S_2$, 552.8971; found: 552.9022.

5-Bromo-*N*-((5-chlorobenzo[b]thiophen-3-yl)methyl)-*N*-((2,4-dichlorophenyl)difluoromethyl) thiophene-2-sulfonamide (62)

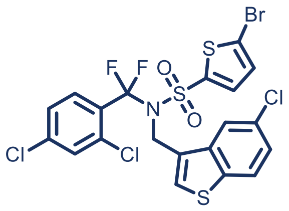

Prepared according to **General Procedure D**, 3-(bromomethyl)-5-chlorobenzo[b]thiophene (52.3 mg, 0.2 mmol, 1 equiv.), tetrabutylammonium iodide (83.26 mg, 0.22 mmol, 1.1 equiv.) and *N*-((5-bromothiophen-2-yl)sulfonyl)-2,4-dichlorobenzimidoyl chloride (**SI-28**) solution (8 mL, 0.1 M, 0.8 mmol, 4 equiv.). The crude was purified using flash column chromatography (100% *n*-pentane to 10% AcOEt in *n*-pentane), affording compound **62** (90 mg, 73%) as a white solid.

^1H NMR (300 MHz, $CDCl_3$) δ 7.77 (d, J = 8.6 Hz, 1H), 7.73 (s, 1H), 7.63 (d, J = 2.0 Hz, 1H), 7.57 (d, J = 8.6 Hz, 1H), 7.36 – 7.30 (m, 1H), 7.25 (dd, J = 8.5, 2.2 Hz, 2H), 7.03 (d, J = 4.0 Hz, 1H), 6.98 (d, J = 4.0 Hz, 1H), 4.95 (s, 2H).

^{13}C NMR (75 MHz, $CDCl_3$) δ 140.2, 138.4, 138.2, 138.2 (t, J = 1.8 Hz), 133.7, 133.7 (t, J = 3.3 Hz), 130.7, 130.7 (t, J = 6.7 Hz), 130.6, 130.0, 130.0, 129.2 (t, J = 30.2 Hz), 128.3, 126.9, 124.9, 123.8, 121.8, 120.9, 118.7 (t, J = 256.8 Hz), 44.2.

^{19}F NMR (282 MHz, $CDCl_3$) δ -68.08 (s, 2F).

HRMS (FD+) (m/z): $[M]^+$ calculated for $C_{20}H_{11}BrCl_3F_2NO_2S_3$, 616.8144; found: 616.8262.

5-Bromo-*N*-((2,4-dichlorophenyl)difluoromethyl)-*N*-(3-(5-(2-fluorophenyl)-1,2,4-oxadiazol-3-yl)benzyl)thiophene-2-sulfonamide (63)

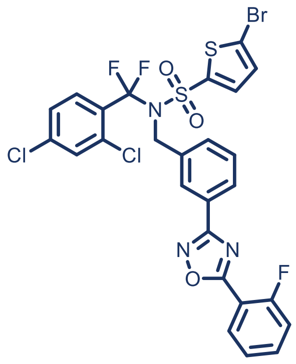

Prepared according to **General Procedure D**, 3-(3-(bromomethyl)phenyl)-5-(2-fluorophenyl)-1,2,4-oxadiazole (66.6 mg, 0.2 mmol, 1 equiv.), tetrabutylammonium iodide (83.26 mg, 0.22 mmol, 1.1 equiv.) and *N*-((5-bromothiophen-2-yl)sulfonyl)-2,4-dichlorobenzimidoyl chloride (**SI-28**) solution (8 mL, 0.1 M, 0.8 mmol, 4 equiv.). The crude was purified using flash column chromatography (100% *n*-pentane to 10% AcOEt in *n*-pentane), affording compound **63** (105 mg, 77%) as a white solid.

^{1}H NMR (300 MHz, $CDCl_3$) δ 8.25 (td, *J* = 7.3, 1.9 Hz, 1H), 8.13 (d, *J* = 7.7 Hz, 1H), 8.07 – 8.01 (m, 1H), 7.70 – 7.60 (m, 3H), 7.51 (t, *J* = 7.7 Hz, 1H), 7.41 – 7.26 (m, 4H), 7.06 (d, *J* = 4.1 Hz, 1H), 6.99 (d, *J* = 4.0 Hz, 1H), 4.86 (s, 2H).

^{13}C NMR (75 MHz, $CDCl_3$) δ 172.8 (d, *J* = 4.4 Hz), 168.3, 160.7 (d, *J* = 260.6 Hz), 140.4, 138.2, 136.6, 134.6 (d, *J* = 8.7 Hz), 134.0, 133.8, 131.2, 130.9, 130.8, 130.8, 130.1, 129.3 (t, *J* = 30.0 Hz), 129.1, 127.3, 127.0, 126.9, 126.8, 124.7 (d, *J* = 3.8 Hz), 121.7, 118.9 (t, *J* = 256.5 Hz), 117.1 (d, *J* = 20.9 Hz), 112.8 (d, *J* = 11.4 Hz), 50.2.

^{19}F NMR (282 MHz, $CDCl_3$) δ -68.71 (s, 2F), -108.18 (s, 1F).

HRMS (FD+) (m/z): $[M]^+$ calculated for $C_{26}H_{15}BrCl_2F_3N_3O_3S_2$, 688.9045; found: 688.9074.

_N_-(Difluoro(4-(trifluoromethyl)phenyl)methyl)-4-methyl-_N_-(4-((trimethylsilyl)ethynyl)benzyl)-benzenesulfonamide (64)

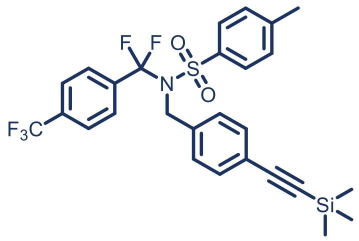

Compound **4** (53.4 mg, 0.1 mmol, 1 equiv.), $Pd(PPh_3)_2Cl_2$ (3.82 mg, 5.4 µmol, 5.4 mol%), PPh_3 (2.6 mg, 10 µmol, 10 mol%) and CuI (2.22 mg, 11.6 µmol, 11.6 mol%) were mixed inside a vial that was evacuated and backfilled with N_2 (×3). To this vial, dry DMF (1 mL) was added, followed by Et_3N (39 µL, 0.28 mmol, 2.8 equiv.) and trimethylsilylacetylene (18 µL, 0.13 mmol, 1.3 equiv.). The reaction mixture was stirred at 80 °C for 16 hours, after which it was evaporated in vacuo and purified using column chromatography (100% *n*-pentane to 10% AcOEt in *n*-pentane), affording compound **64** (31.4 mg, 57%) as a clear oil.

^{1}H NMR (300 MHz, $CDCl_3$) δ 7.55 – 7.39 (m, 6H), 7.38 – 7.27 (m, 4H), 7.16 (d, *J* = 8.1 Hz, 2H), 4.71 (s, 2H), 2.41 (s, 3H), 0.27 (s, 9H).

^{13}C NMR (75 MHz, $CDCl_3$) δ 144.5, 137.3 (t, *J* = 30.5 Hz), 137.2, 136.9, 132.8 (q, *J* = 33.2 Hz), 132.2, 129.6, 128.3, 127.7, 127.6 (t, *J* = 4.7 Hz), 125.1 (q, *J* = 3.8 Hz), 123.6 (t, *J* = 272.4 Hz) 122.8, 119.9 (t, *J* = 256.1 Hz) 104.7, 94.9, 49.3, 21.6, 0.0.

^{19}F NMR (282 MHz, $CDCl_3$) δ -63.08 (s, 3F), -69.78 (s, 2F).

HRMS (FD+) (m/z): $[M]^+$ calculated for $C_{27}H_{26}F_5NO_2SSi$, 551.1373; found: 551.1373.

_N_-(Difluoro(4-(trifluoromethyl)phenyl)methyl)-4-methyl-_N_-((4'-methyl-[1,1'-biphenyl]-4-yl)methyl)benzenesulfonamide (65)

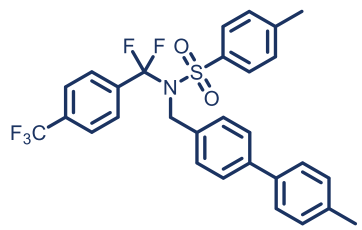

Compound **4** (53.4 mg, 0.1 mmol, 1 equiv.), XPhos Pd G4 (8.6 mg, 0.01 mmol, 10 mol%) and p-tolylboronic acid (27.2 mg, 0.2 mmol, 2 equiv.) were mixed inside a vial that was evacuated and backfilled with N_2 (×3). To this vial, dry dioxane (0.83 mL), water (0.17 mL) and DBU (29.9 µL, 0.2 mmol, 2 equiv.) were added, and N_2 was bubbled through the solution for 10 minutes. The reaction was heated at 100 °C for 18 hours, after which the solvent was evaporated and the crude was purified using column chromatography (100% *n*-pentane to 10% AcOEt in *n*-pentane), affording compound **65** (21.7 mg, 40%) as a white solid.

^{1}H NMR (300 MHz, $CDCl_3$) δ 7.61 – 7.46 (m, 8H), 7.46 – 7.33 (m, 4H), 7.27 (d, *J* = 8.2 Hz, 2H), 7.16 (d, *J* = 8.1 Hz, 2H), 4.77 (s, 2H), 2.42 (s, 3H), 2.40 (s, 3H).

^{13}C NMR (101 MHz, $CDCl_3$) δ 144.3, 140.8, 137.8, 137.5 (t, *J* = 31.4 Hz), 137.4, 137.2, 135.5, 132.8 (q, *J* = 33.1 Hz), 129.7, 129.6, 128.9, 127.7, 127.7 (t, *J* = 4.5 Hz), 127.1, 127.0, 125.0 (q, *J* = 3.7 Hz), 123.6 (q, *J* = 273.5 Hz), 120.0 (t, *J* = 255.8 Hz), 49.4, 21.6, 21.2.

^{19}F NMR (282 MHz, $CDCl_3$) δ -63.05 (s, 3F), -69.75 (s, 2F).

HRMS (FD+) (m/z): $[M]^+$ calculated for $C_{29}H_{24}F_5NO_2S$, 545.1447; found: 545.1441.

***N*-(Difluoro(4-(trifluoromethyl)phenyl)methyl)-4-methyl-*N*-(4-(piperidin-1-yl)benzyl)-benzenesulfonamide (66)**

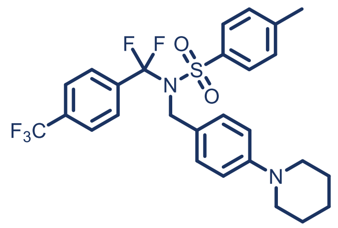

Compound **4** (53.4 mg, 0.1 mmol, 1 equiv.), XPhos Pd G4 (8.6 mg, 0.01 mmol, 10 mol%) and caesium carbonate (65.2 mg, 0.2 mmol, 2 equiv.) were mixed inside a vial that was evacuated and backfilled with N_2 (×3). To this vial, toluene (1 mL) was added and N_2 was bubbled through the solution for 10 minutes. Then, piperidine (19.8 µL, 0.2 mmol, 2 equiv.) was added and the reaction was heated at 100 °C for 18 hours, after which the solvent was evaporated and the crude was purified using column chromatography (100% *n*-pentane to 10% AcOEt in *n*-pentane), affording compound **66** (20.0 mg, 37%) as a clear oil.

^{1}H NMR (300 MHz, $CDCl_3$) δ 7.53 – 7.40 (m, 4H), 7.40 – 7.30 (m, 2H), 7.22 (d, *J* = 8.6 Hz, 2H), 7.14 (d, *J* = 8.1 Hz, 2H), 6.88 (t, *J* = 8.6 Hz, 2H), 4.65 (s, 2H), 3.22 – 3.12 (m, 4H), 2.39 (s, 3H), 1.79 – 1.66 (m, 4H), 1.66 – 1.53 (m, 2H).

^{13}C NMR (75 MHz, $CDCl_3$) δ 151.7, 143.9, 137.5 (t, *J* = 31.1 Hz), 137.3, 132.5 (q, *J* = 33.2 Hz), 129.5, 129.4, 127.6 (t, *J* = 4.6 Hz), 127.5, 126.7, 124.8 (q, *J* = 3.8 Hz), 123.5 (q, *J* = 272.7 Hz), 119.8 (t, *J* = 255.2 Hz), 116.1, 50.5, 49.2, 25.7, 24.2, 21.5.

^{19}F NMR (282 MHz, $CDCl_3$) δ -63.04 (s, 3F), -69.73 (s, 2F).

HRMS (FD+) (m/z): $[M]^+$ calculated for $C_{27}H_{27}F_5N_2O_2S$, 538.1713; found: 538.1724.

5. NMR Spectra

5.1 Benzyl Bromide Derivatives

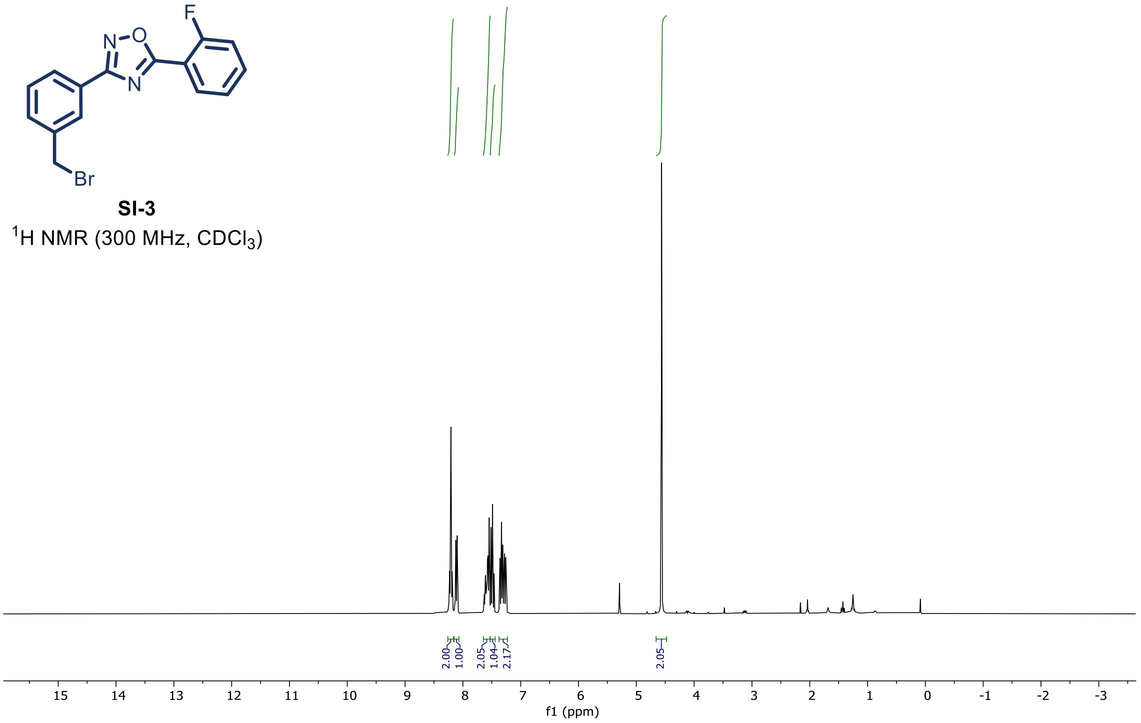

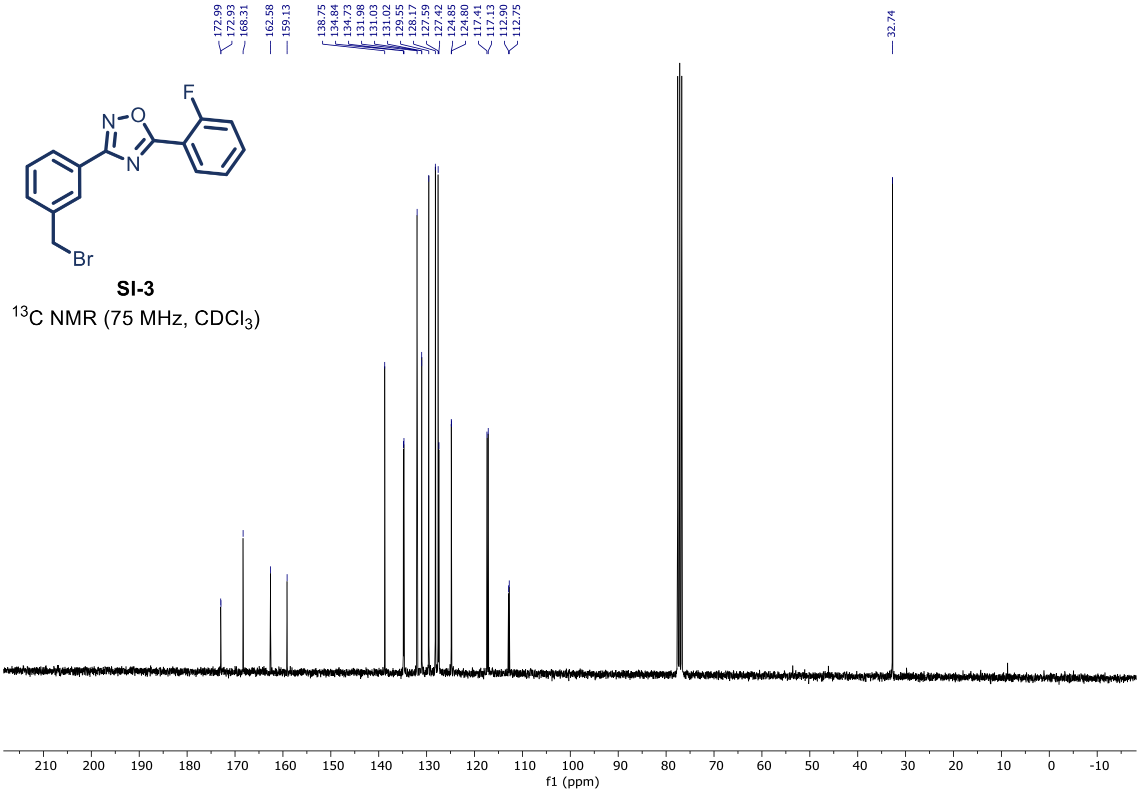

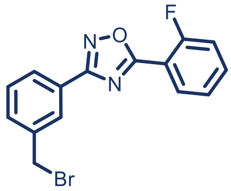

SI-3

^{19}F NMR (282 MHz, $CDCl_3$)

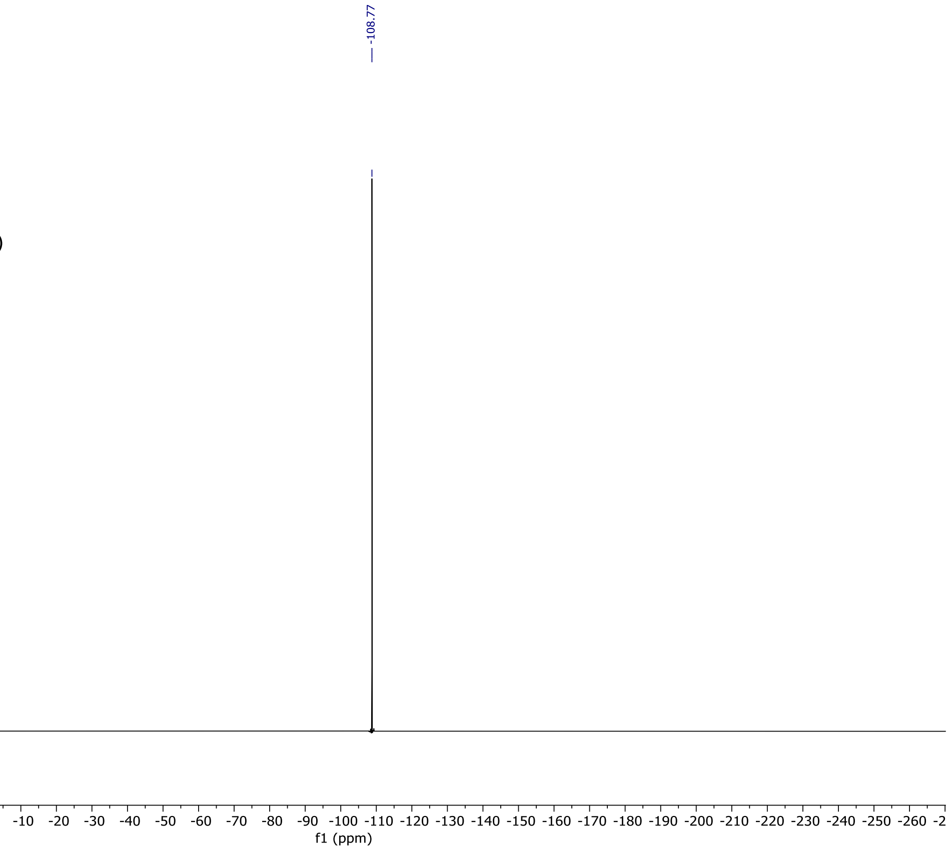

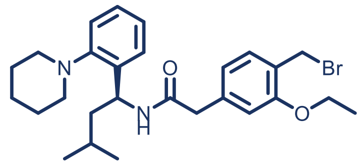

SI-5

^{1}H NMR (300 MHz, $CDCl_3$)

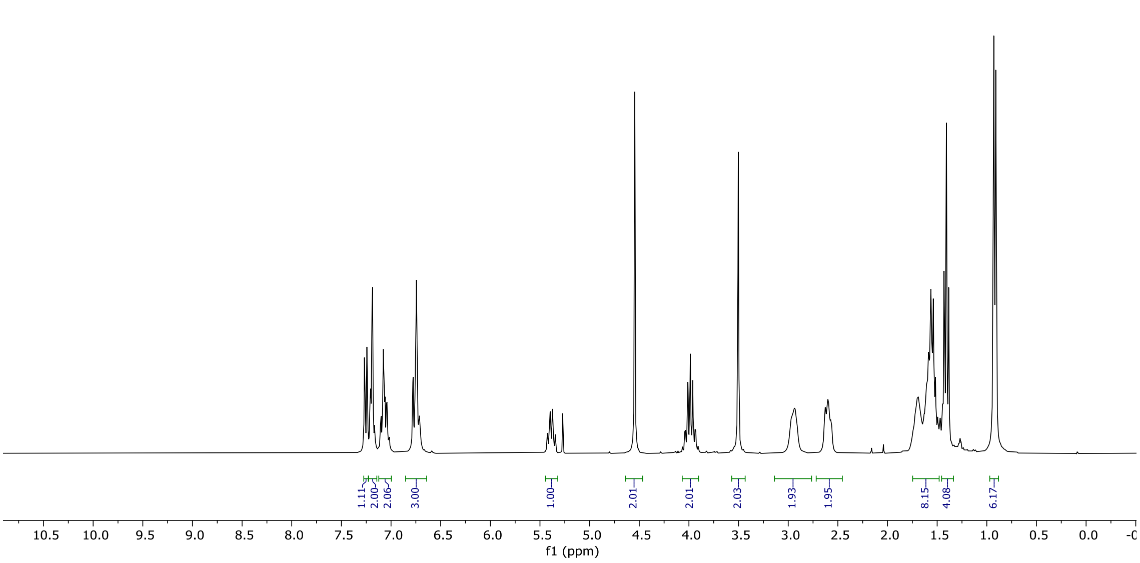

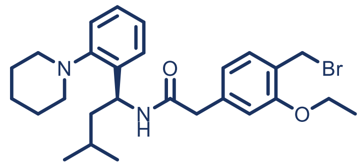

SI-5

^{13}C NMR (75 MHz, $CDCl_3$)

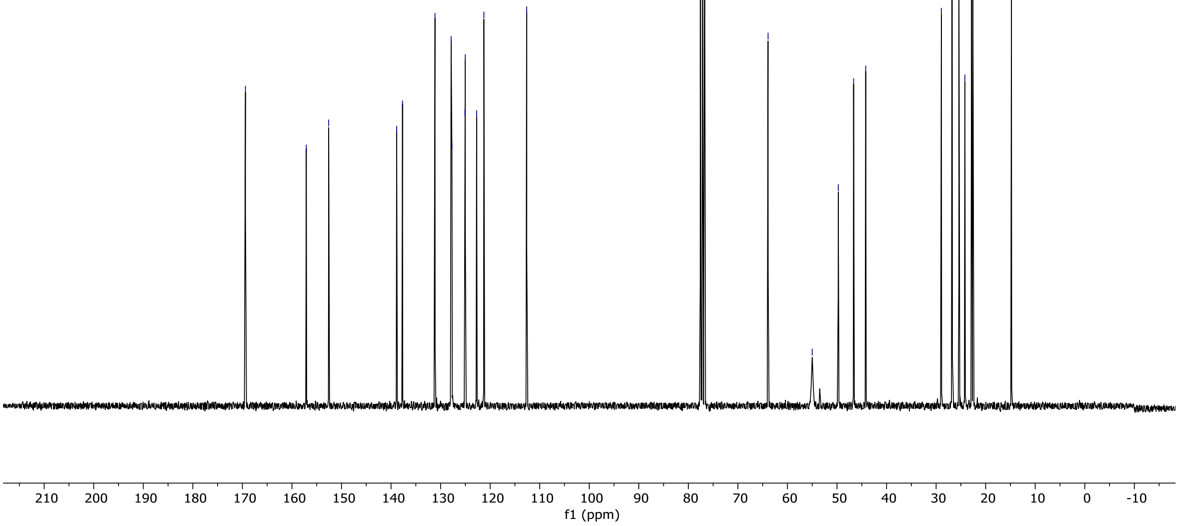

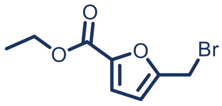

SI-6

^{1}H NMR (300 MHz, $CDCl_3$)

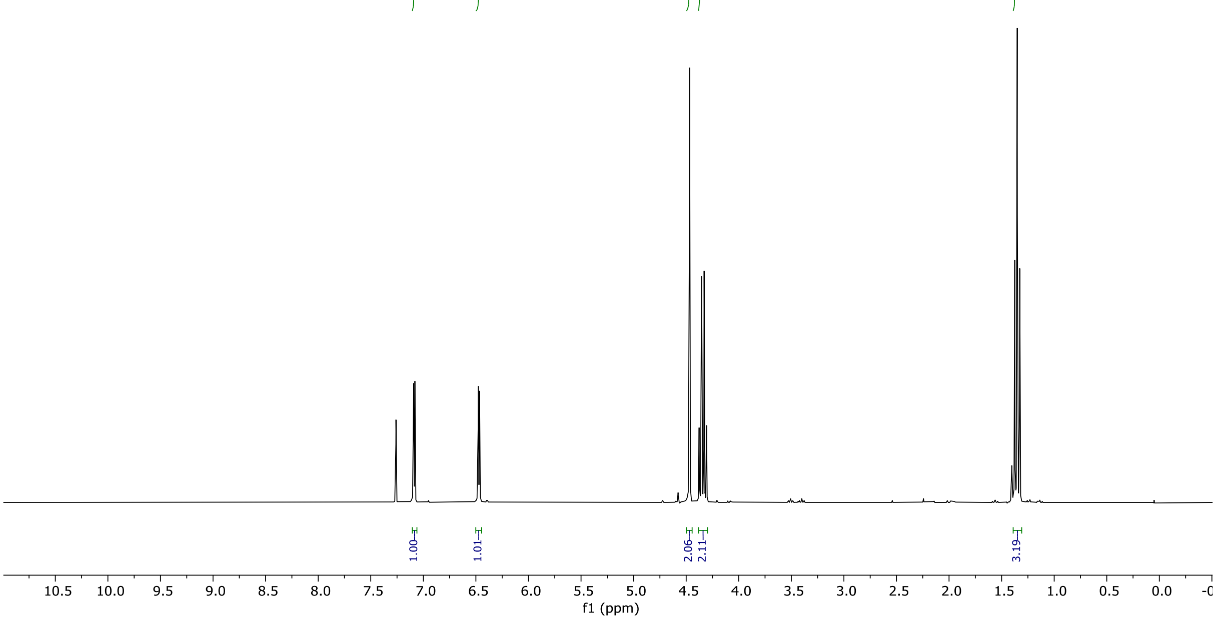

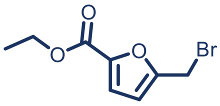

SI-6

^{13}C NMR (75 MHz, $CDCl_3$)

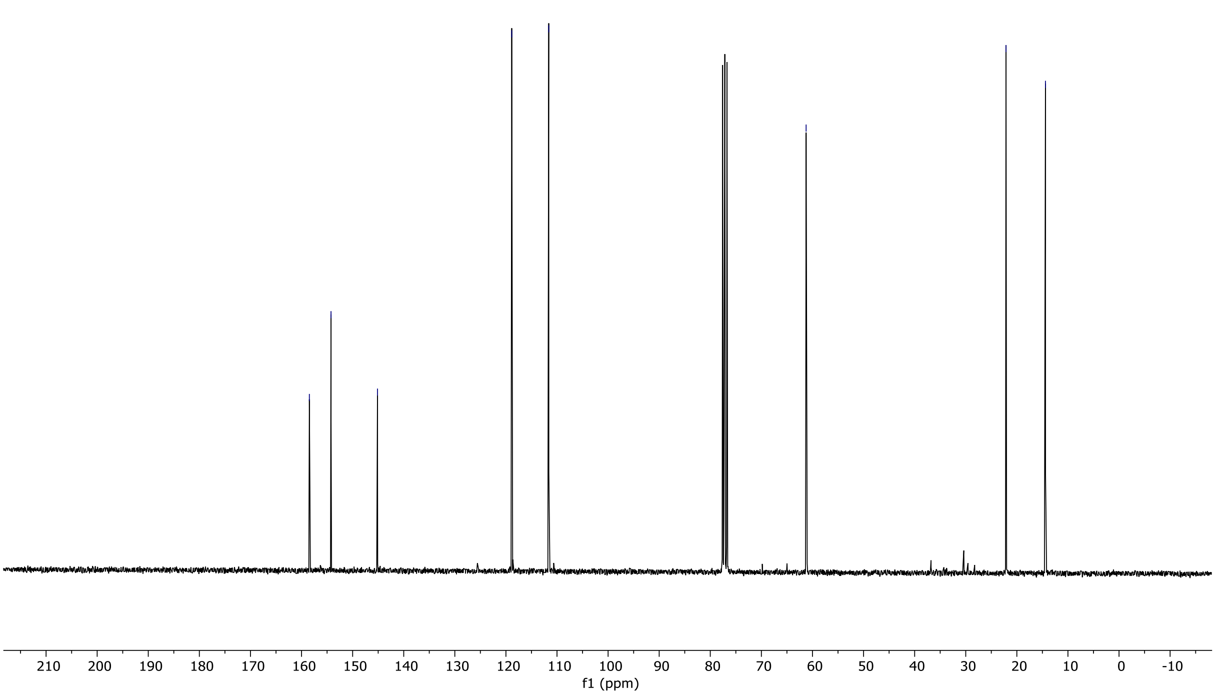

5.2Imidoyl Chlorides

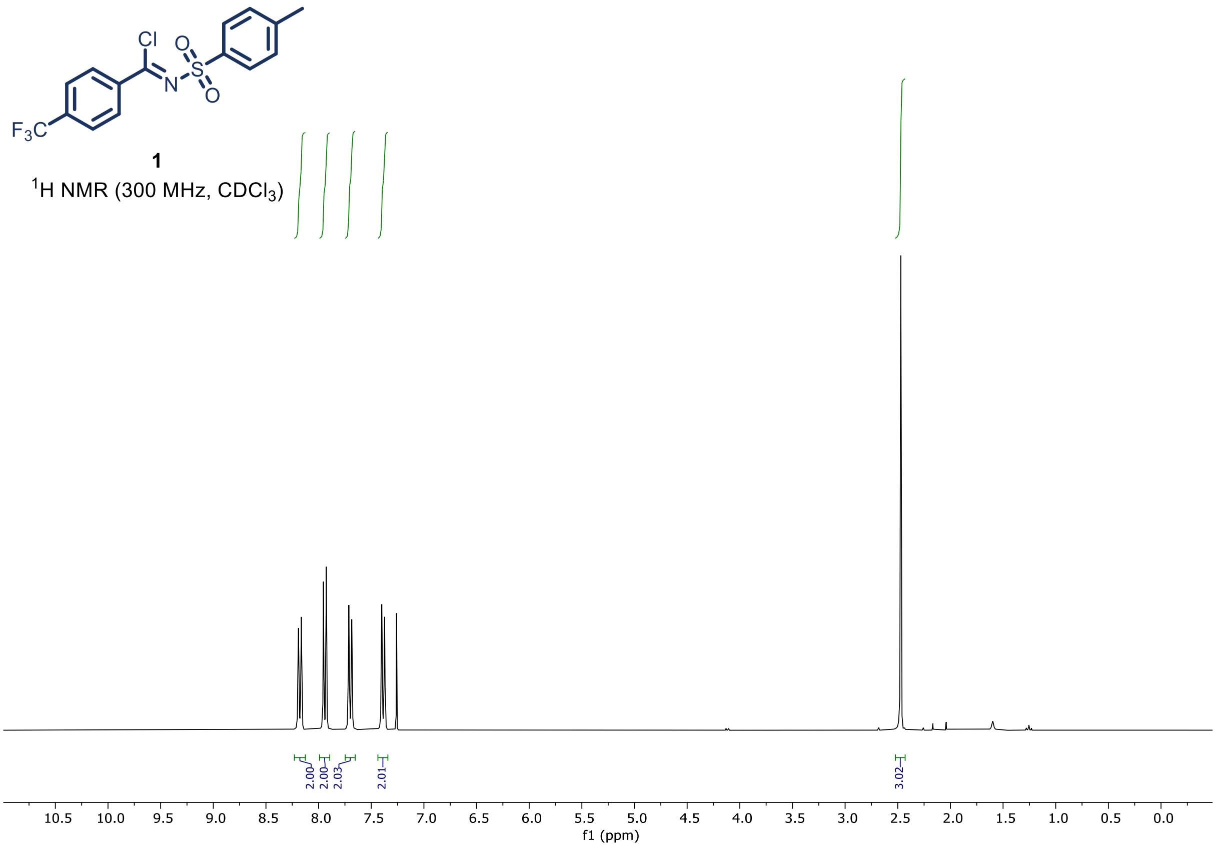

1

^1H NMR (300 MHz, $CDCl_3$)

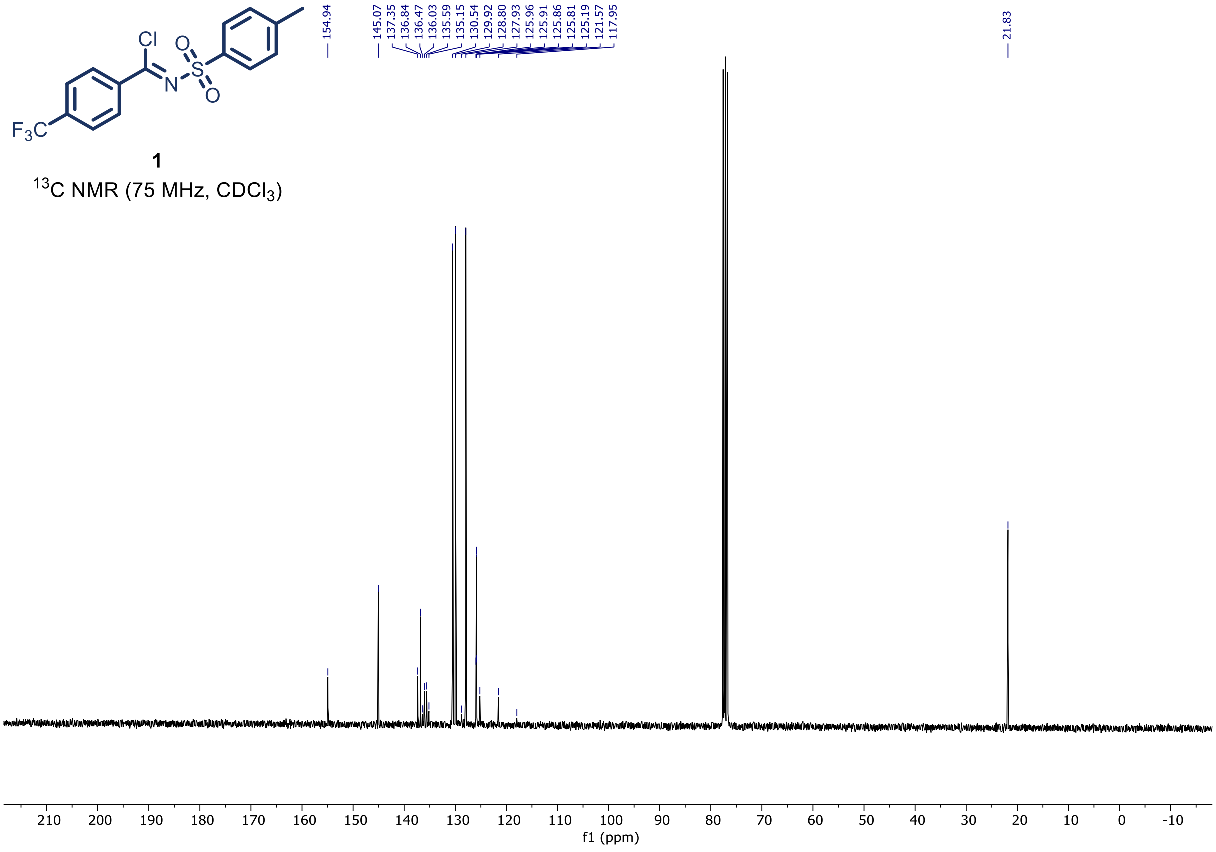

1

^{13}C NMR (75 MHz, $CDCl_3$)

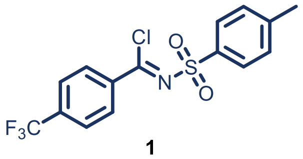

1

^{19}F NMR (282 MHz, $CDCl_3$)

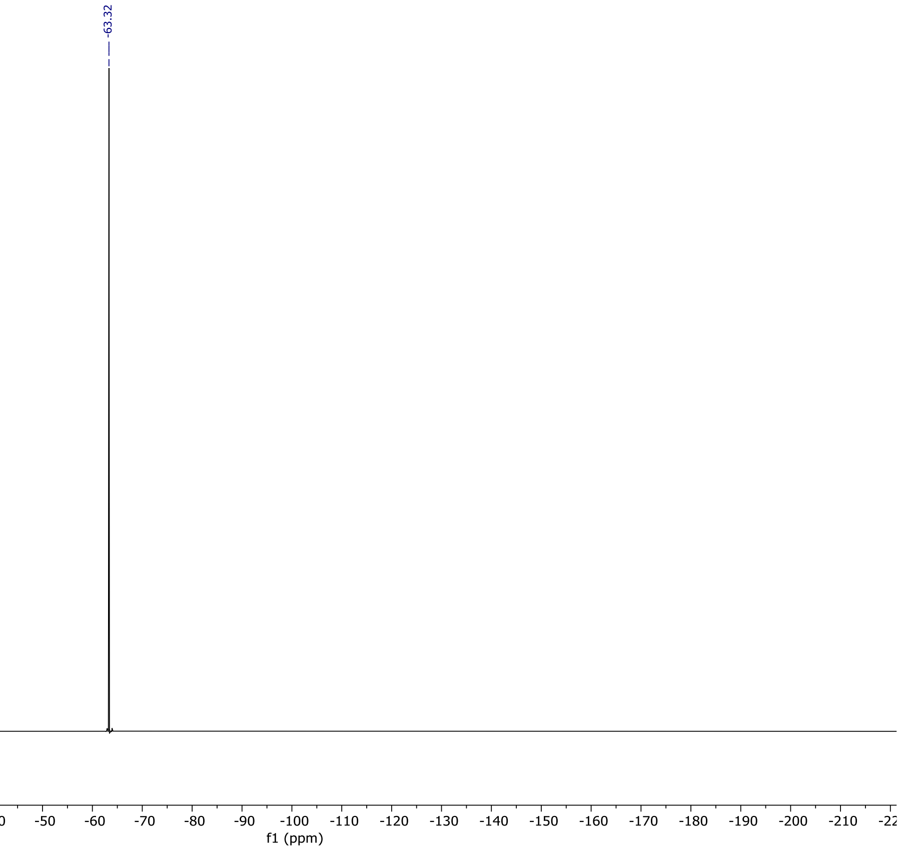

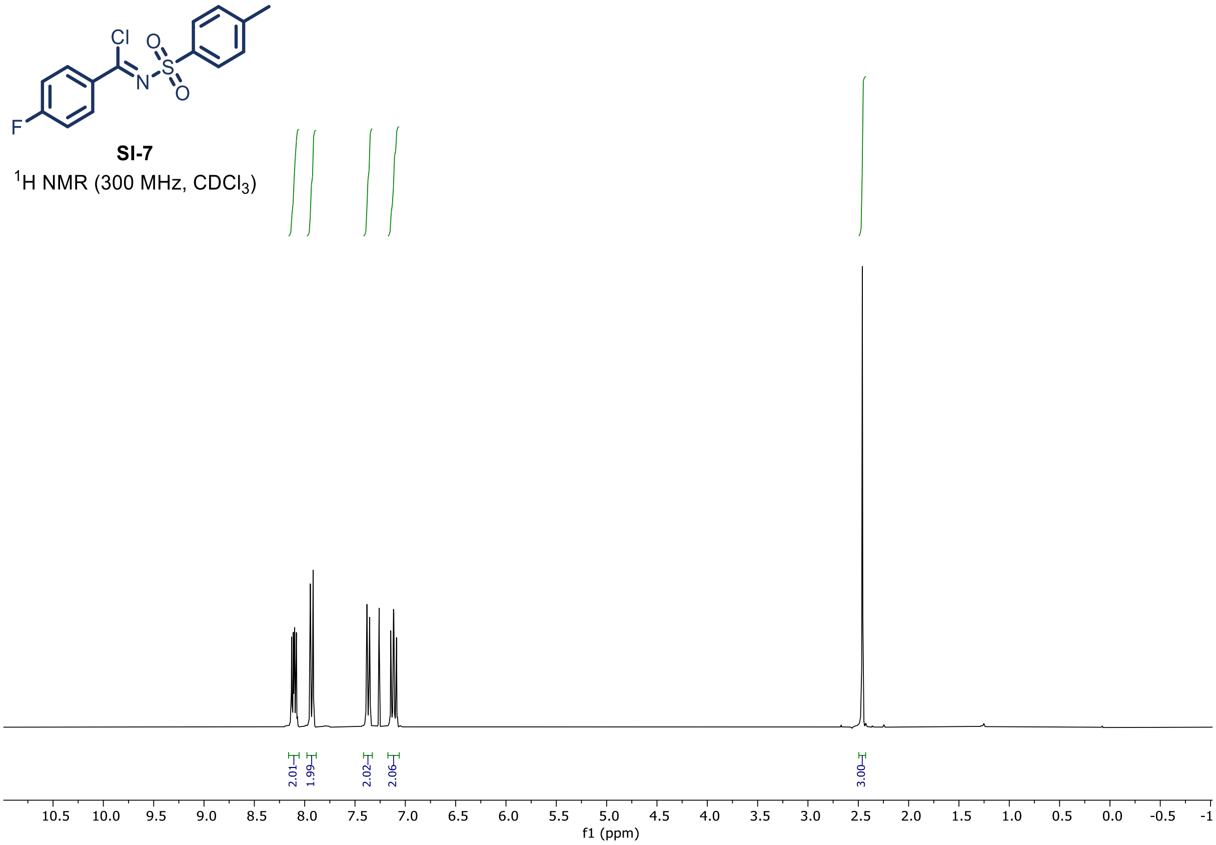
Cl
O
S
N
O
F
SI-7
^{1}H NMR (300 MHz, CDCl$_3$)
2.01
1.99
2.02
2.06
3.00
10.5
10.0
9.5
9.0
8.5
8.0
7.5
7.0
6.5
6.0
5.5
5.0
4.5
4.0
3.5
3.0
2.5
2.0
1.5
1.0
0.5
0.0
-0.5
-1
f1 (ppm)

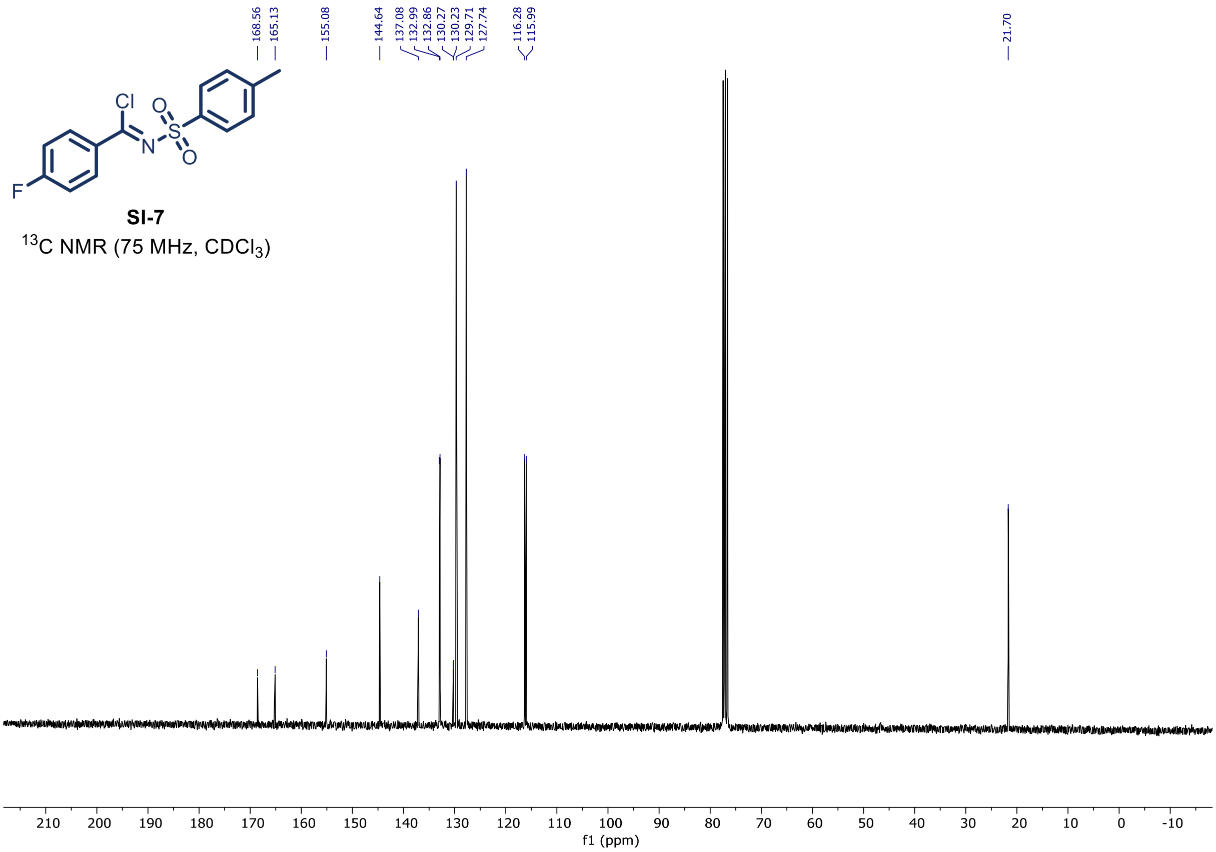
168.56
165.13
155.08
144.64
137.08
132.99
132.86
130.27
130.23
129.71
127.74
116.28
115.99
21.70
Cl
O
S
N
O
F
SI-7
^{13}C NMR (75 MHz, CDCl$_3$)
210
200
190
180
170
160
150
140
130
120
110
100
90
80
70
60
50
40
30
20
10
0
-10
f1 (ppm)

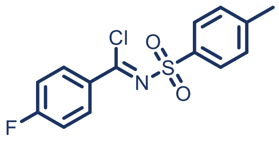

SI-7

^{19}F NMR (282 MHz, $CDCl_3$)

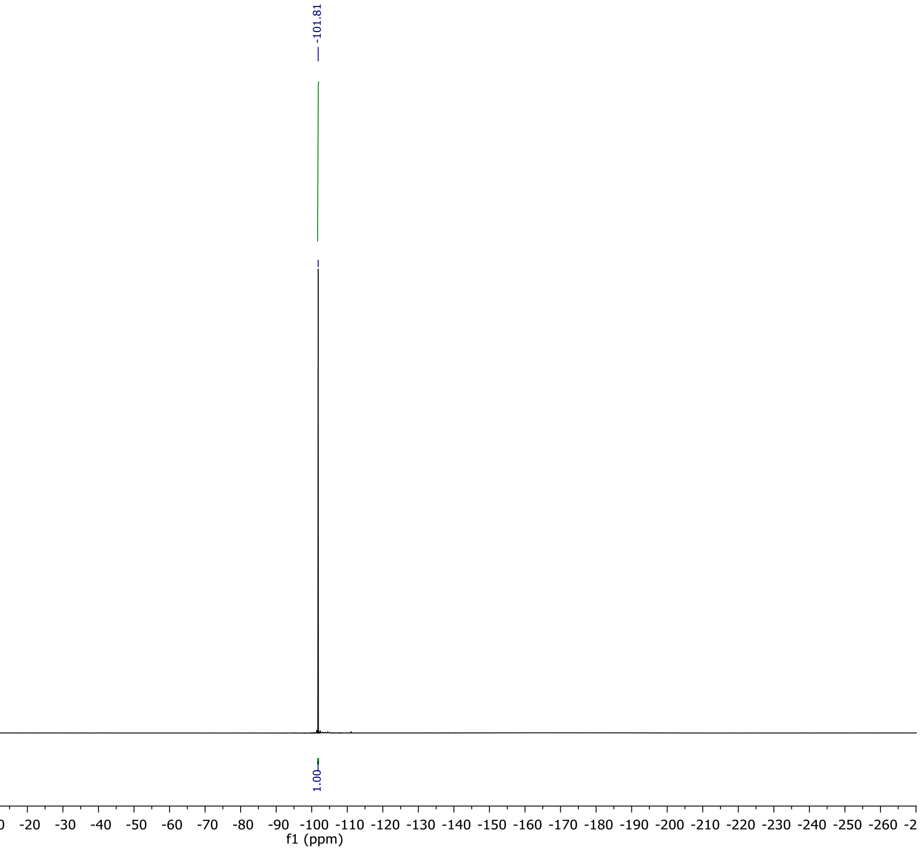

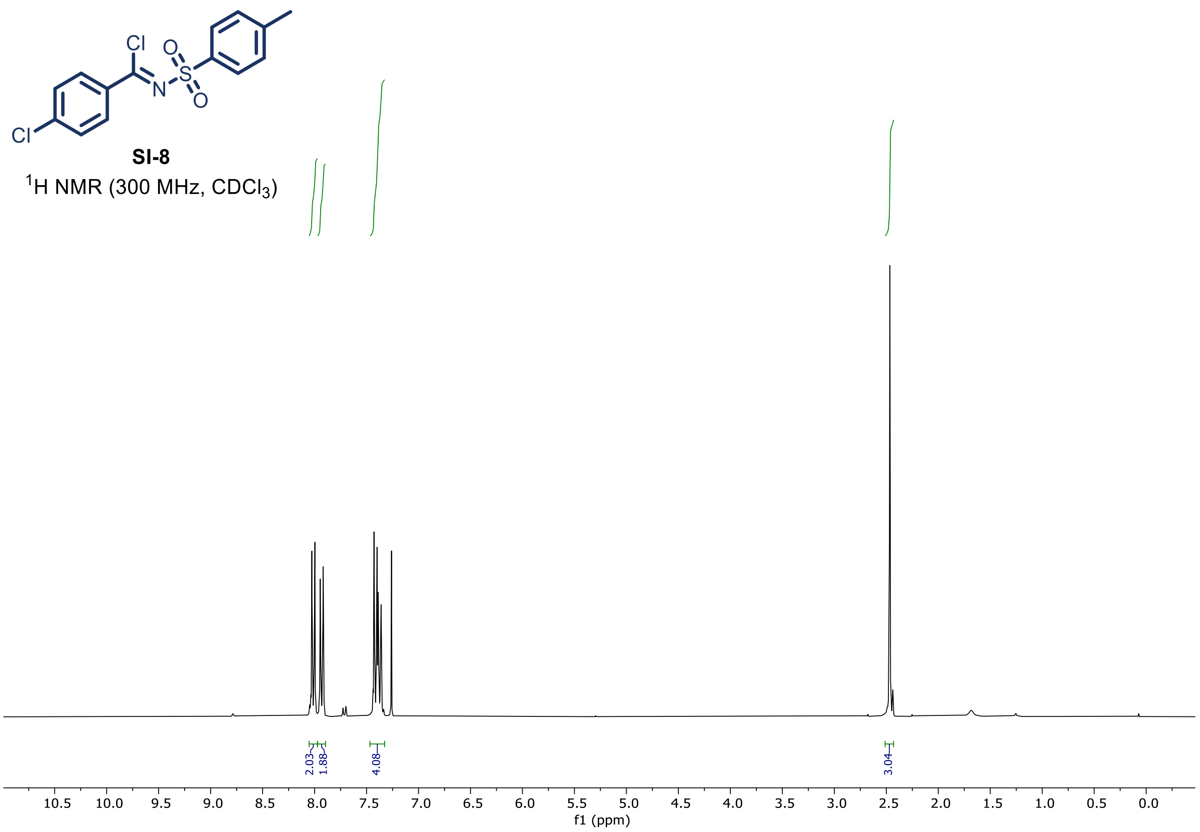
Cl
O
S
N
O
Cl
SI-8
1H NMR (300 MHz, CDCl3)
2.03
1.88
4.08
3.04
10.5 10.0 9.5 9.0 8.5 8.0 7.5 7.0 6.5 6.0 5.5 5.0 4.5 4.0 3.5 3.0 2.5 2.0 1.5 1.0 0.5 0.0
f1 (ppm)

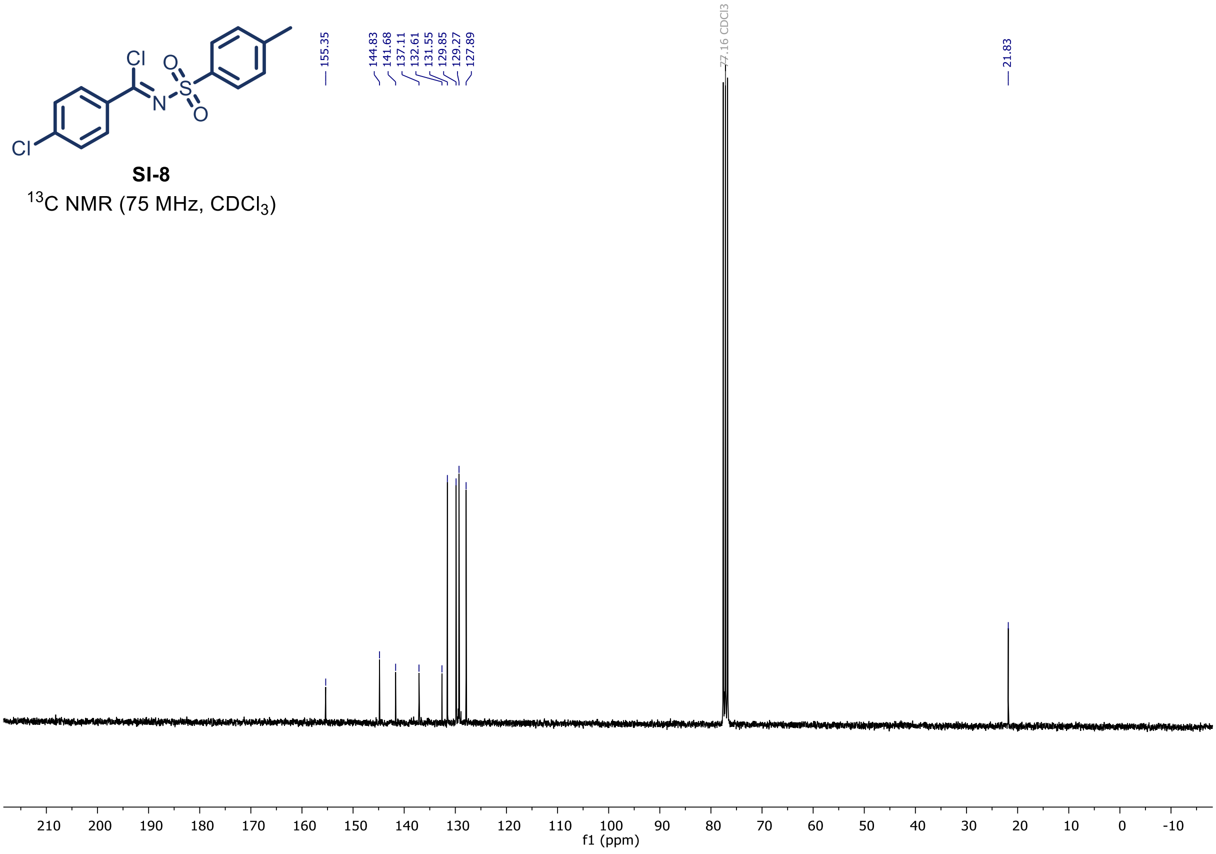
Cl
O
S
N
O
Cl
SI-8
13C NMR (75 MHz, CDCl3)
155.35
144.83
141.68
137.11
132.61
131.55
129.85
129.27
127.89
77.16 CDCl3
21.83
210 200 190 180 170 160 150 140 130 120 110 100 90 80 70 60 50 40 30 20 10 0 -10
f1 (ppm)

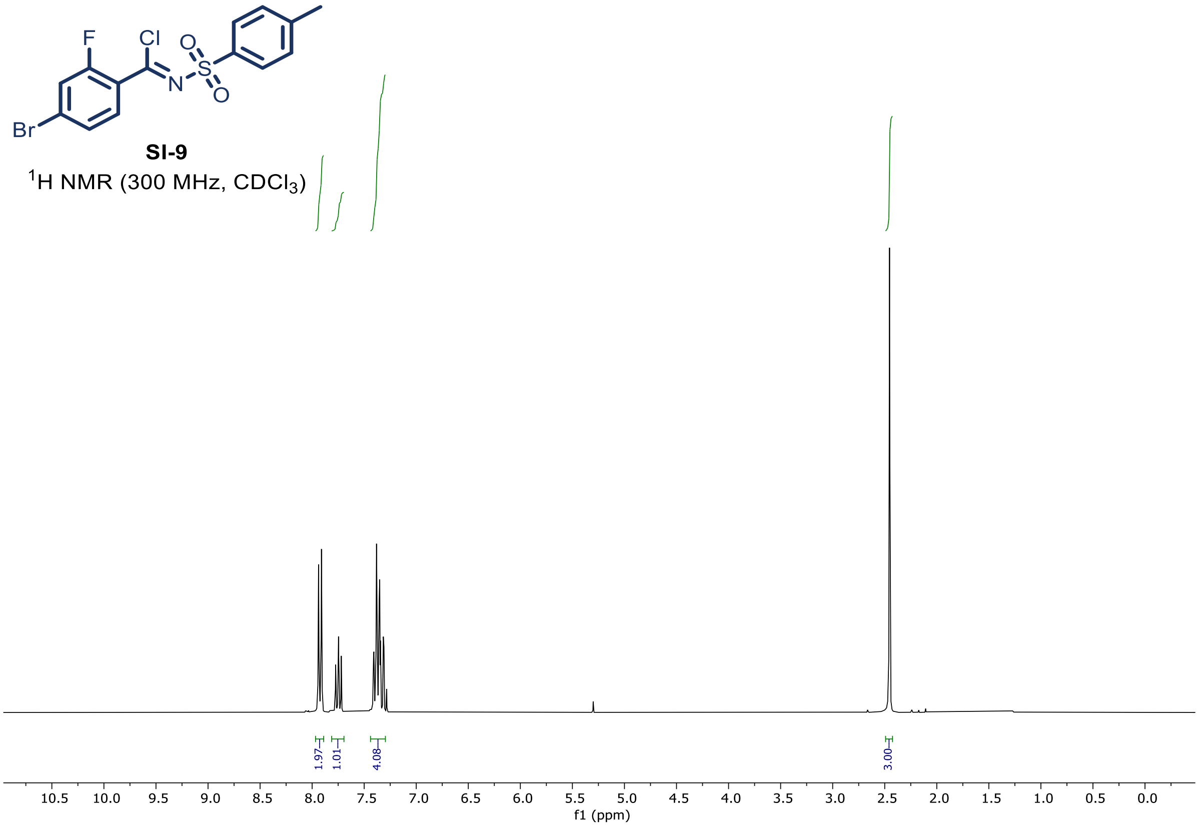
F
Cl
O
S
N
O
Br
SI-9
1H NMR (300 MHz, CDCl3)
1.97
1.01
4.08
3.00
10.5
10.0
9.5
9.0
8.5
8.0
7.5
7.0
6.5
6.0
5.5
5.0
4.5
4.0
3.5
3.0
2.5
2.0
1.5
1.0
0.5
0.0
f1 (ppm)

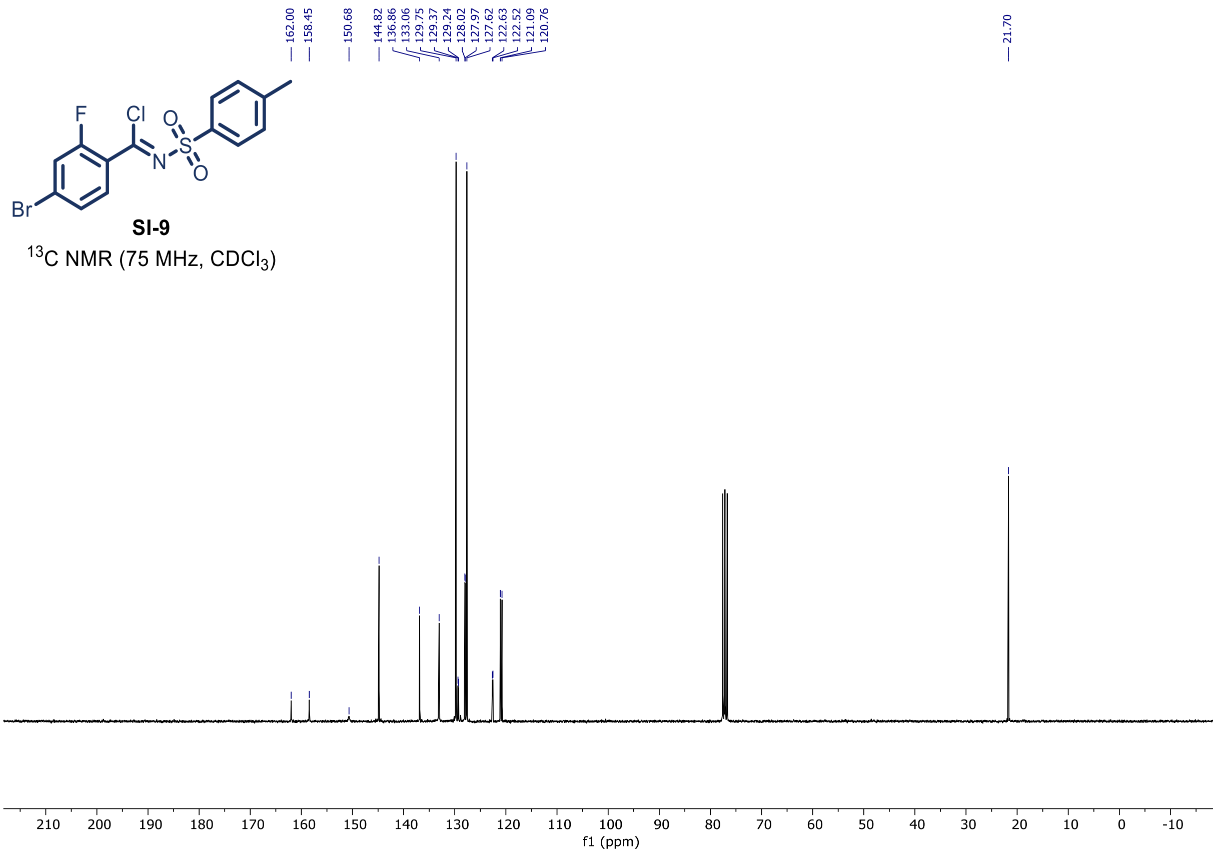
162.00
158.45
150.68
144.82
136.86
133.06
129.75
129.37
129.24
128.02
127.97
127.62
122.63
122.52
121.09
120.76
21.70
F
Cl
O
S
N
O
Br
SI-9
13C NMR (75 MHz, CDCl3)
210
200
190
180
170
160
150
140
130
120
110
100
90
80
70
60
50
40
30
20
10
0
-10
f1 (ppm)

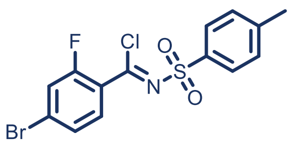

SI-9

^{19}F NMR (282 MHz, $CDCl_3$)

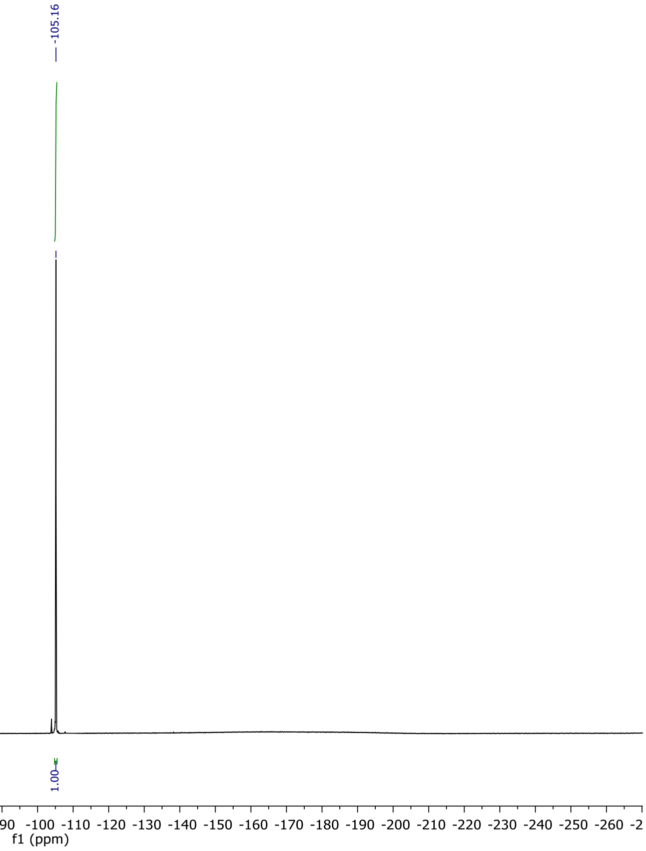

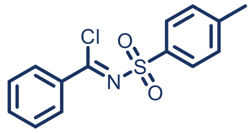

SI-10

^{1}H NMR (300 MHz, $CDCl_3$)

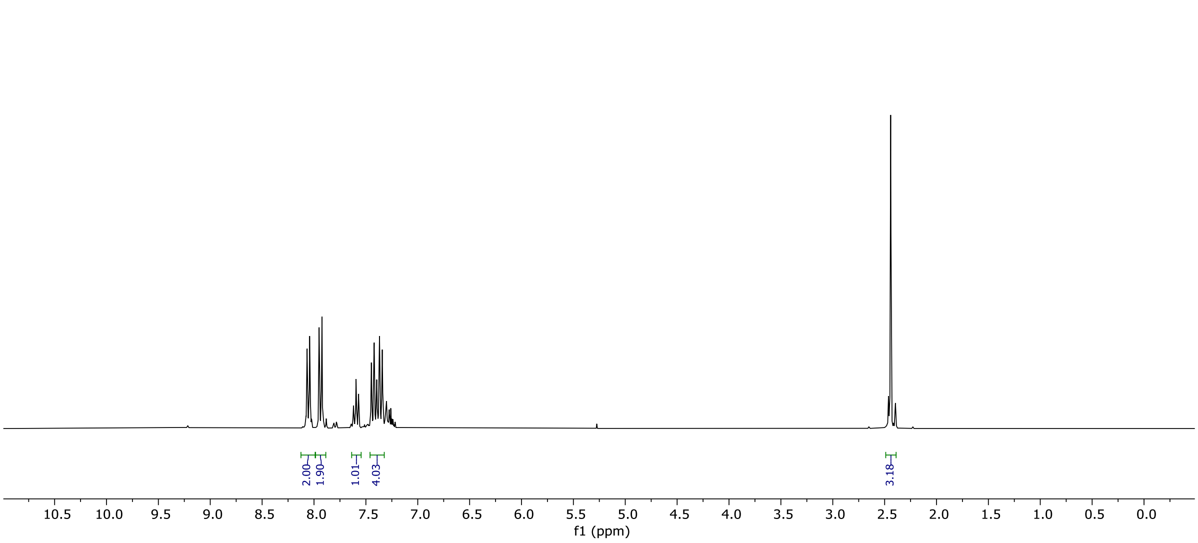

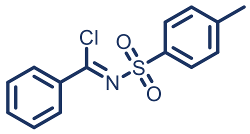

SI-10

^{13}C NMR (75 MHz, $CDCl_3$)

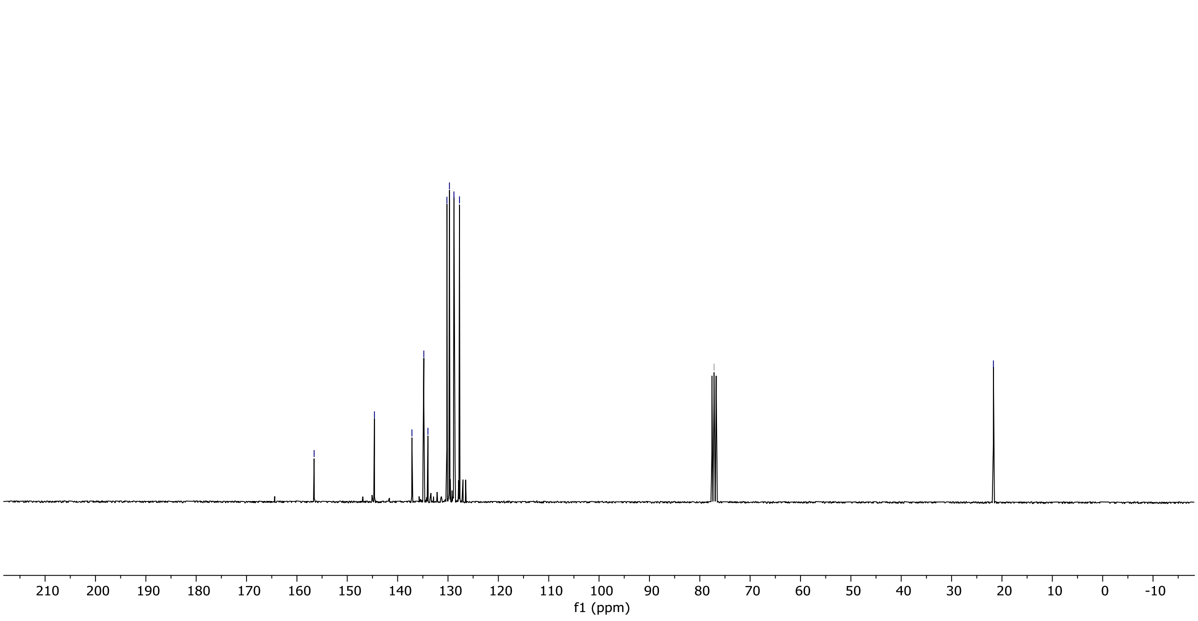

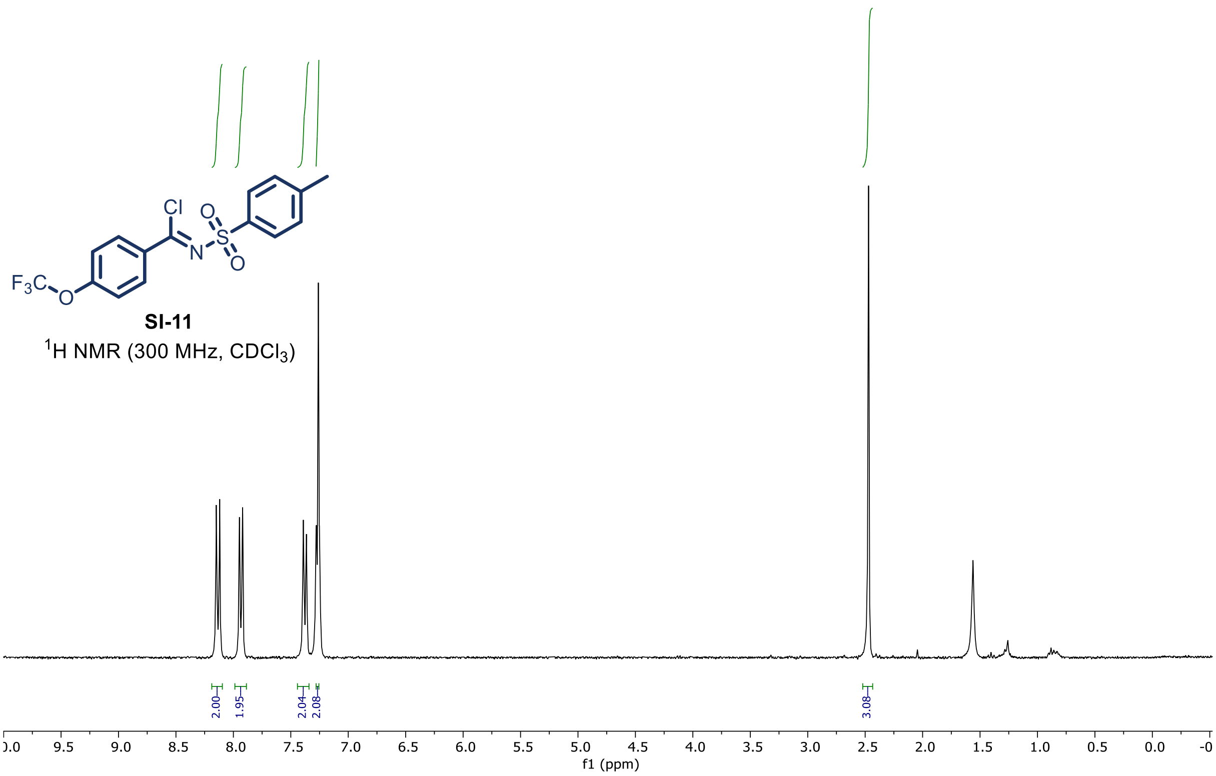
Cl
O
S
N
O
F3C
O
SI-11
1H NMR (300 MHz, CDCl3)
2.00
1.95
2.04
2.08
3.08
f1 (ppm)

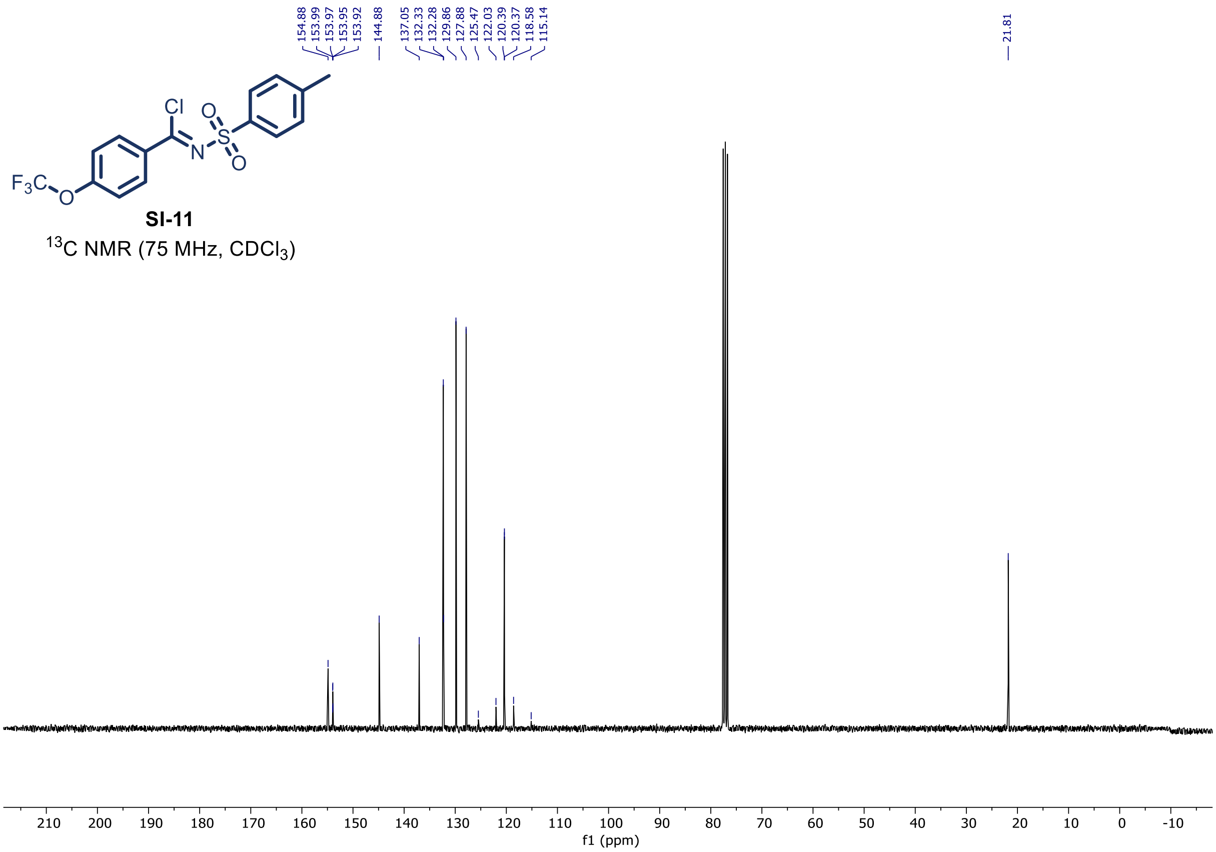
154.88
153.99
153.97
153.95
153.92
144.88
137.05
132.33
132.28
129.86
127.88
125.47
122.03
120.39
120.37
118.58
115.14
21.81
Cl
O
S
N
O
F3C
O
SI-11
13C NMR (75 MHz, CDCl3)
f1 (ppm)

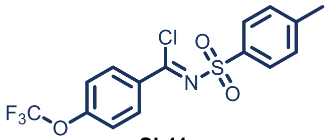

SI-11

^{19}F NMR (282 MHz, $CDCl_3$)

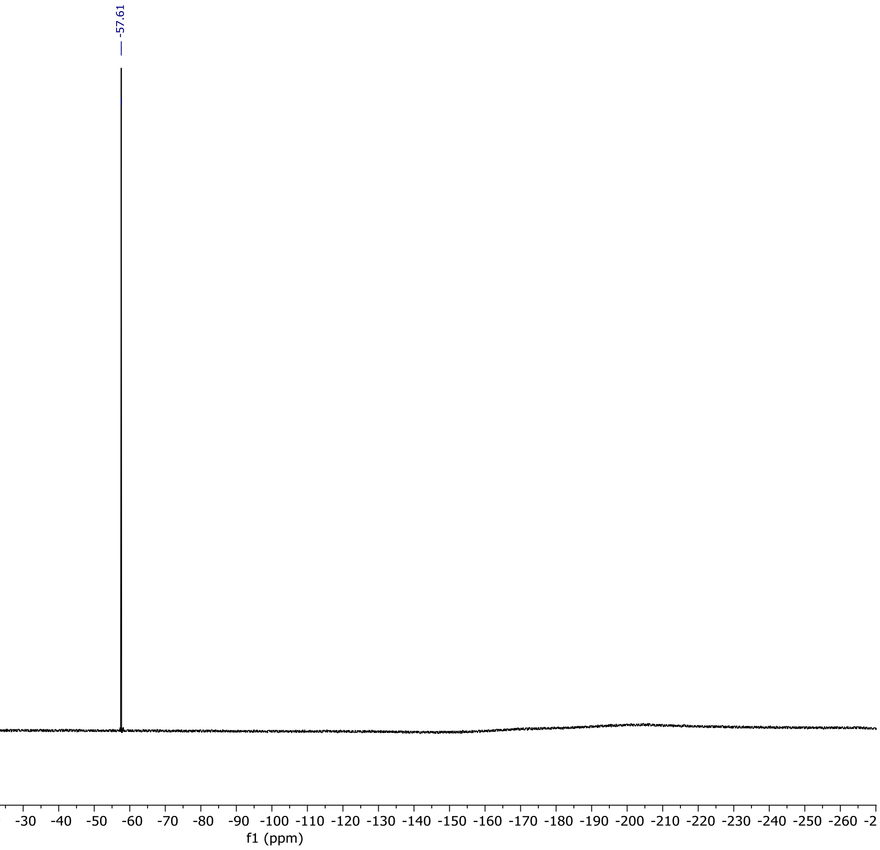

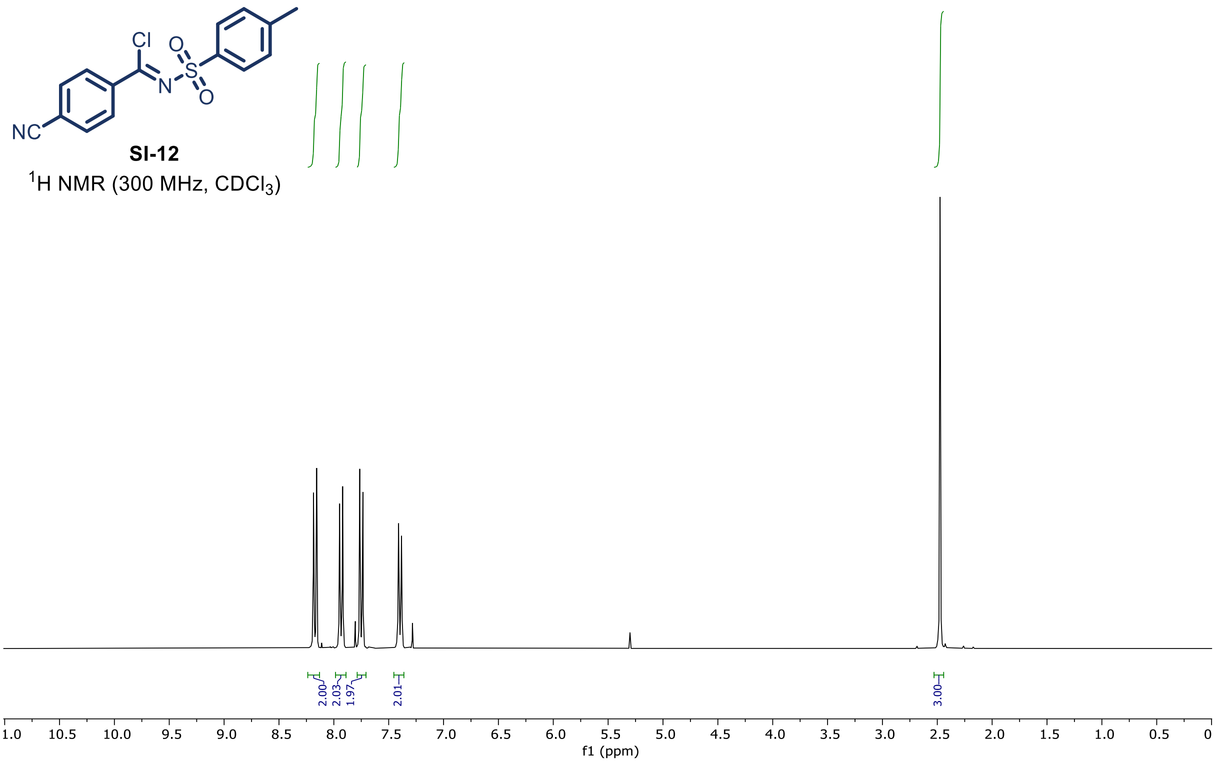
Cl
NC
O
S
O
N
SI-12
^{1}H NMR (300 MHz, CDCl$_3$)
2.00
2.03
1.97
2.01
3.00
f1 (ppm)

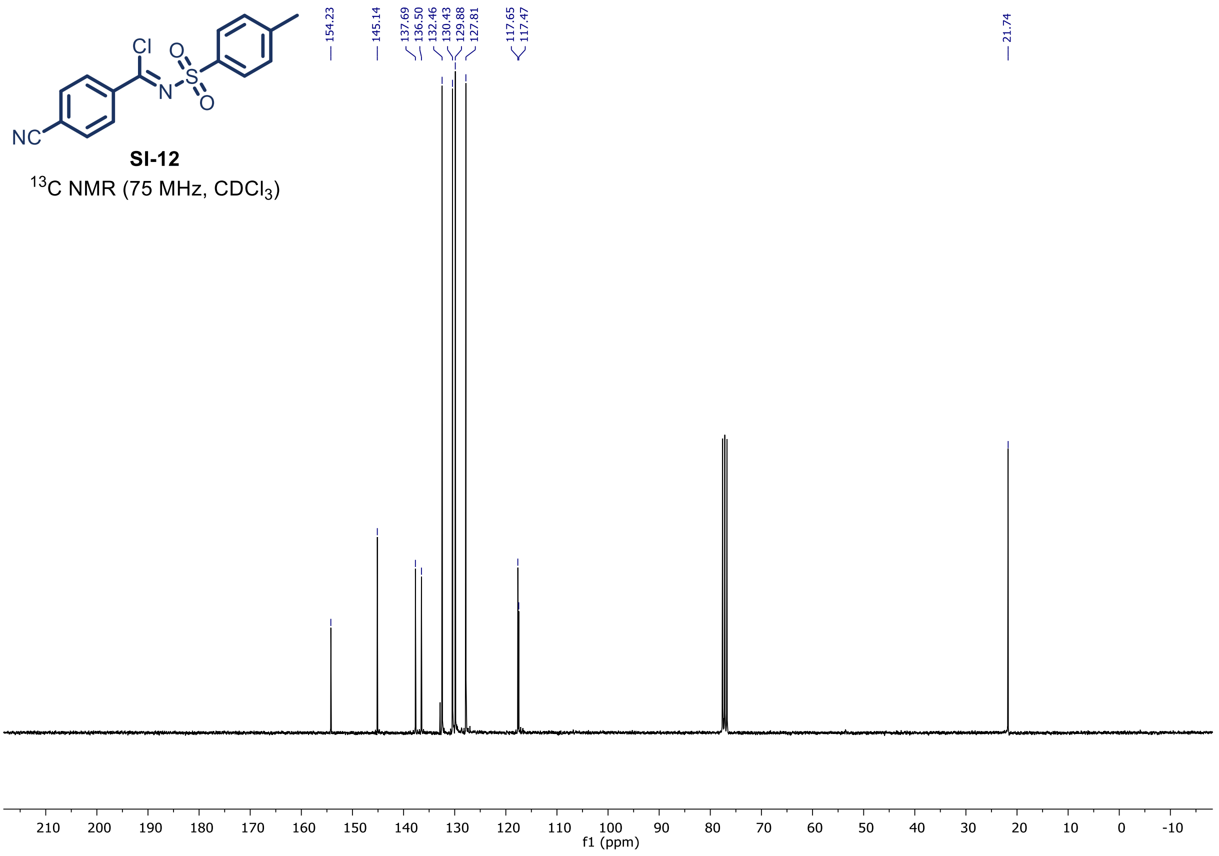
154.23
145.14
137.69
136.50
132.46
130.43
129.88
127.81
117.65
117.47
21.74
Cl
NC
O
S
O
N
SI-12
^{13}C NMR (75 MHz, CDCl$_3$)
f1 (ppm)

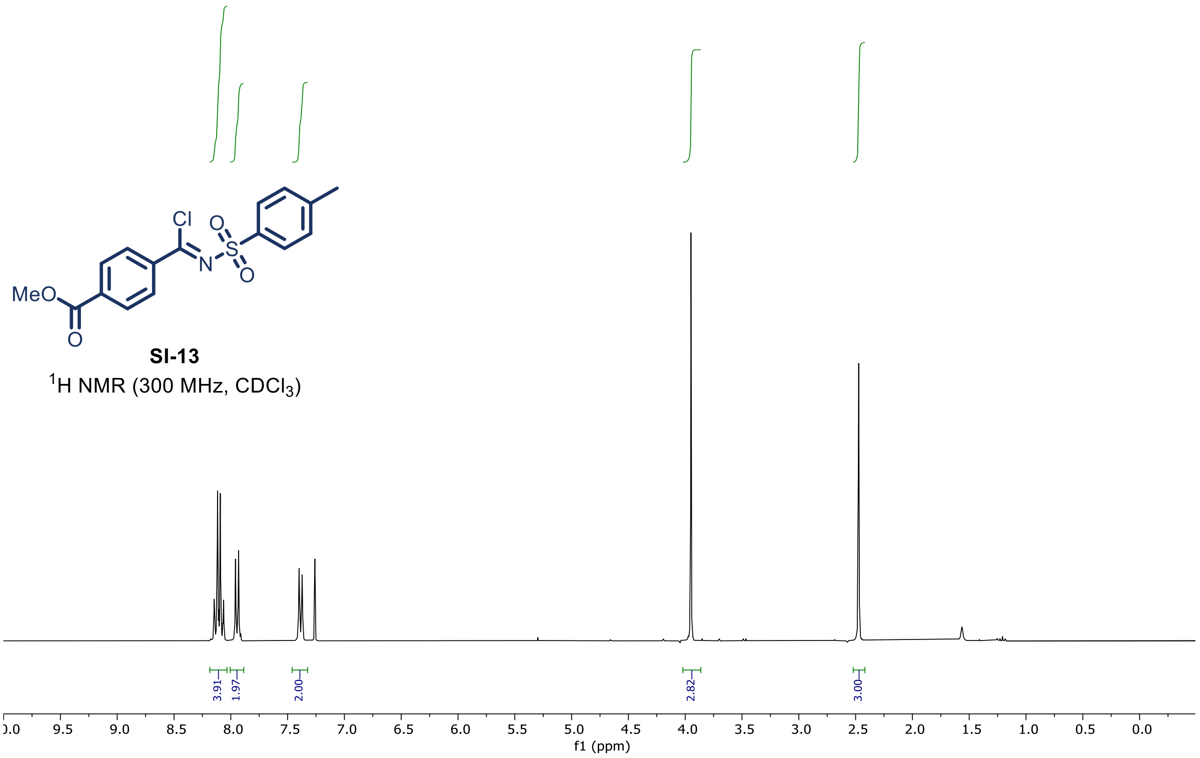

SI-13

^{1}H NMR (300 MHz, $CDCl_3$)

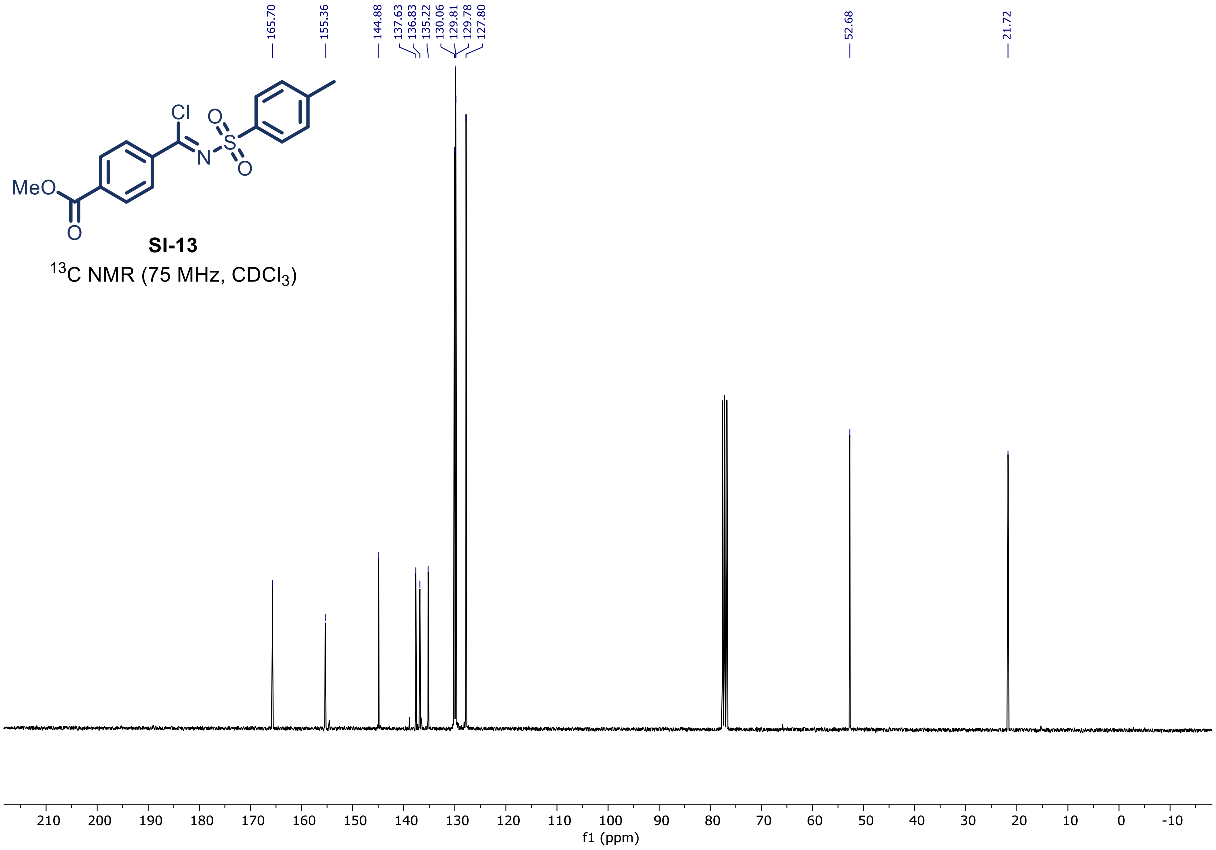

SI-13

^{13}C NMR (75 MHz, $CDCl_3$)

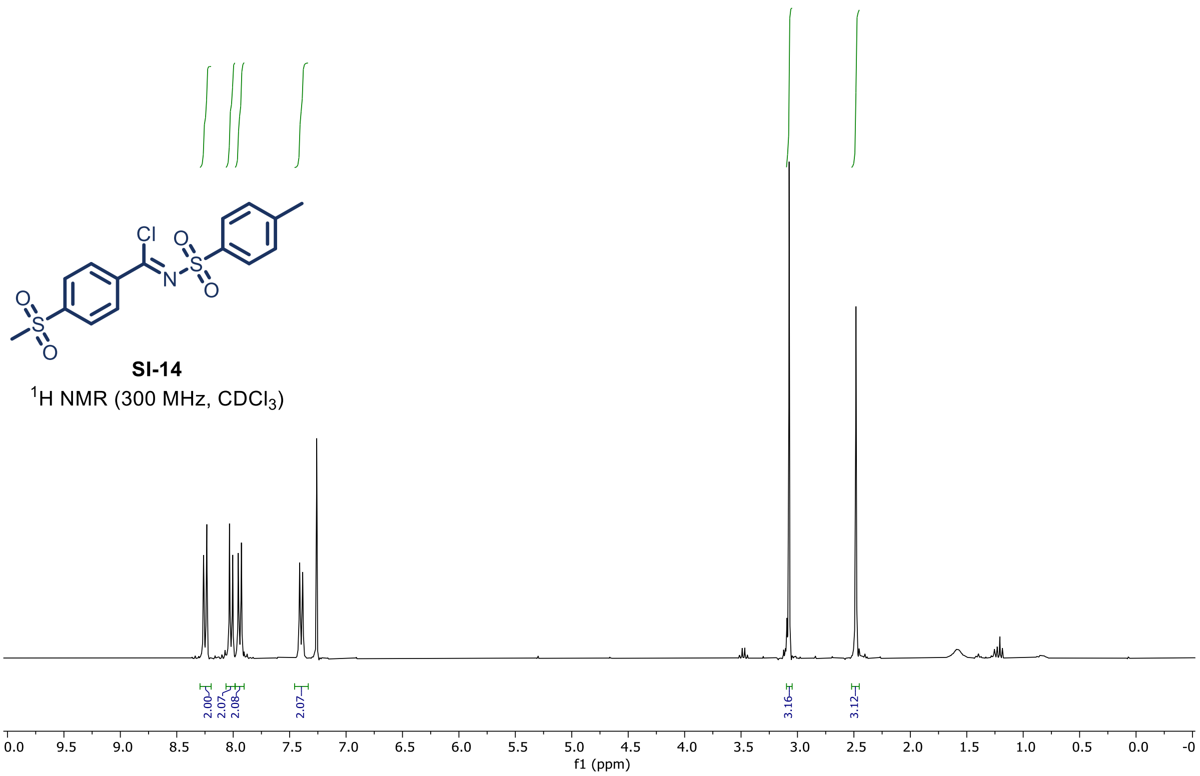
SI-14
1H NMR (300 MHz, CDCl3)
2.00
2.07
2.08
2.07
3.16
3.12
f1 (ppm)

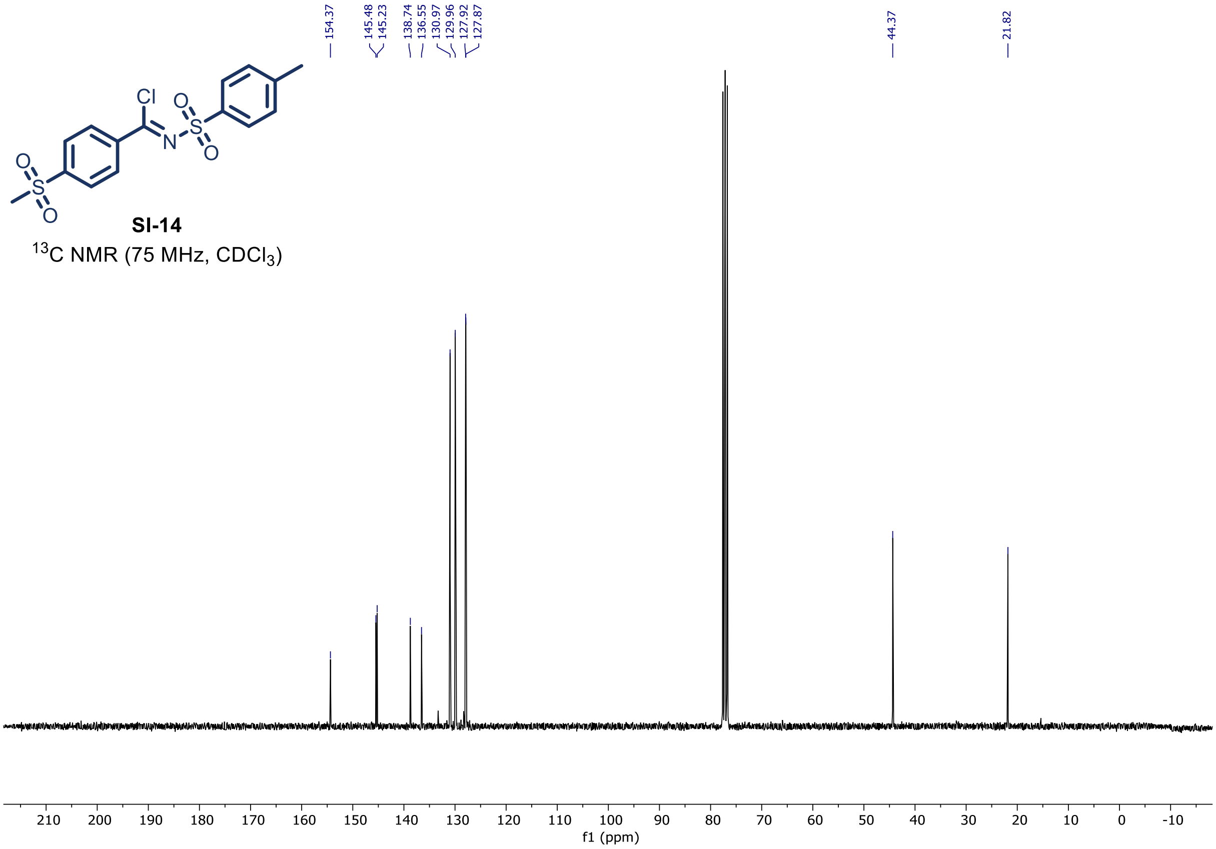
154.37
145.48
145.23
138.74
136.55
130.97
129.96
127.92
127.87
44.37
21.82
SI-14
13C NMR (75 MHz, CDCl3)
f1 (ppm)

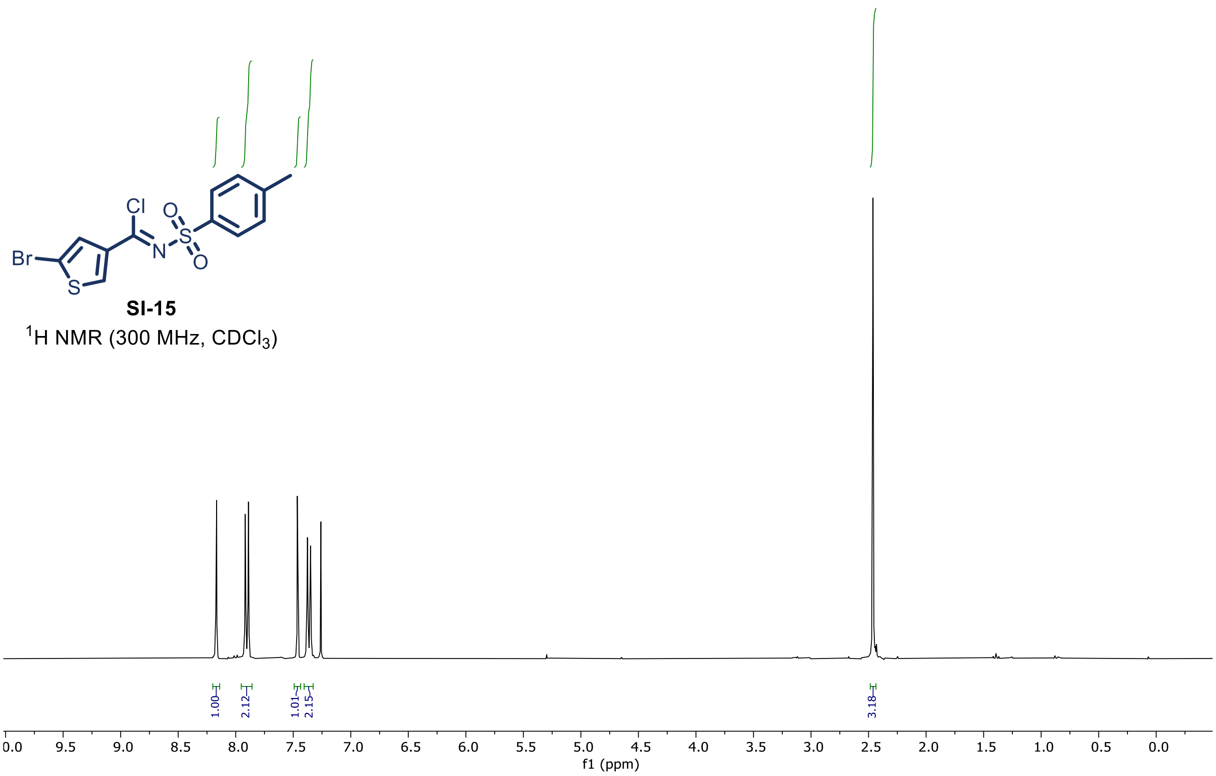

SI-15
^{1}H NMR (300 MHz, $CDCl_3$)

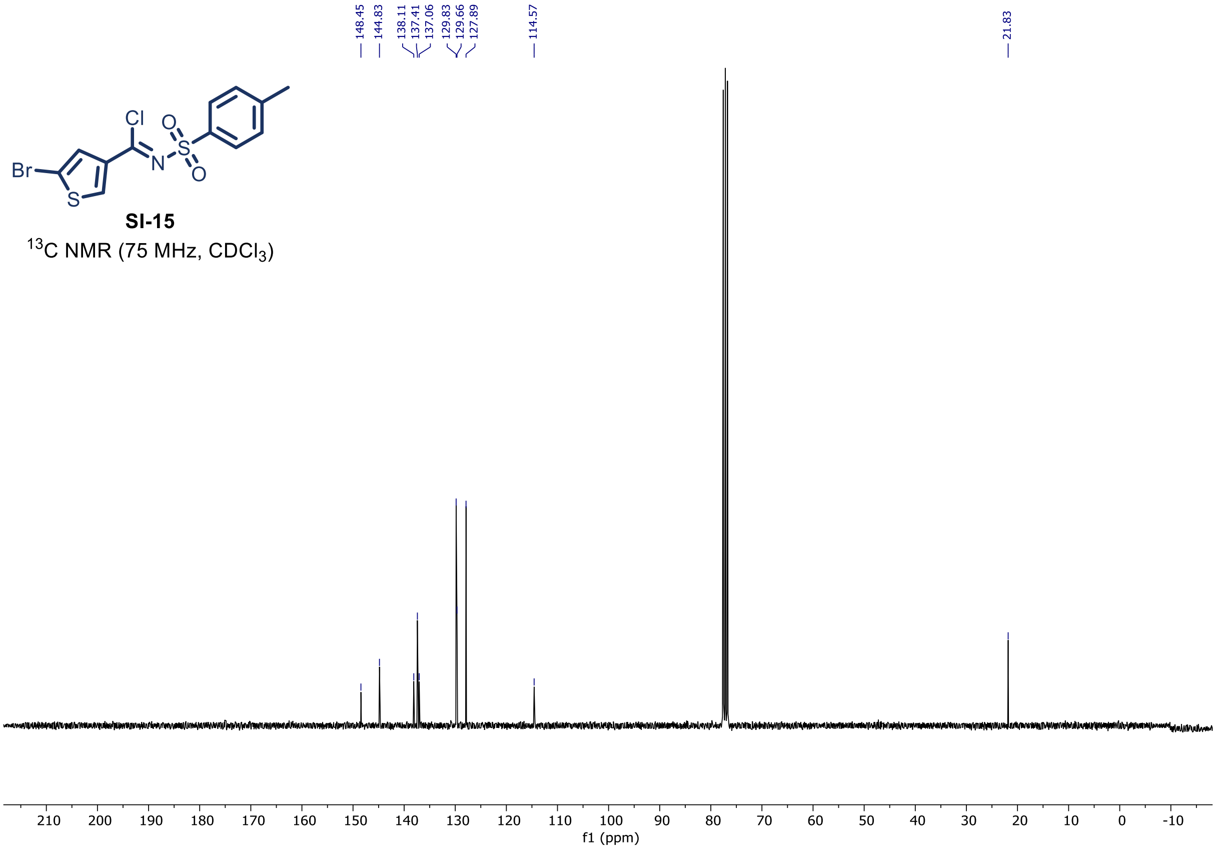

SI-15
^{13}C NMR (75 MHz, $CDCl_3$)

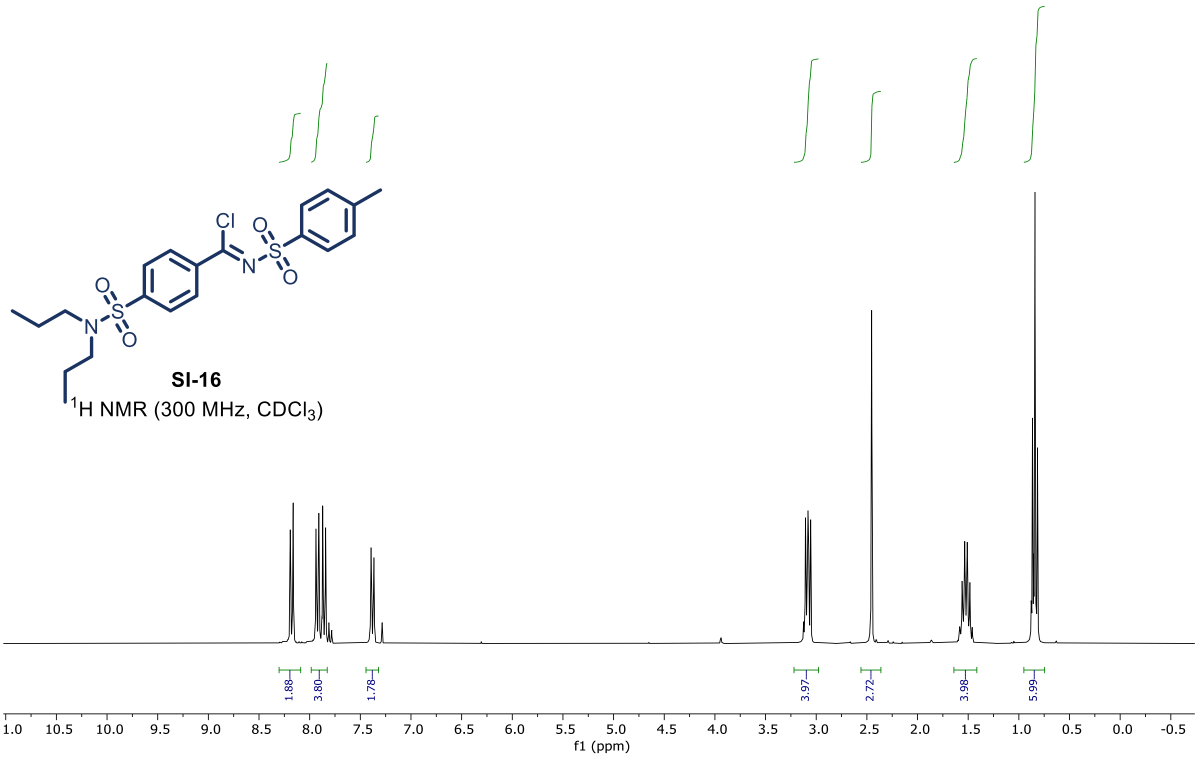

SI-16

^{1}H NMR (300 MHz, $CDCl_3$)

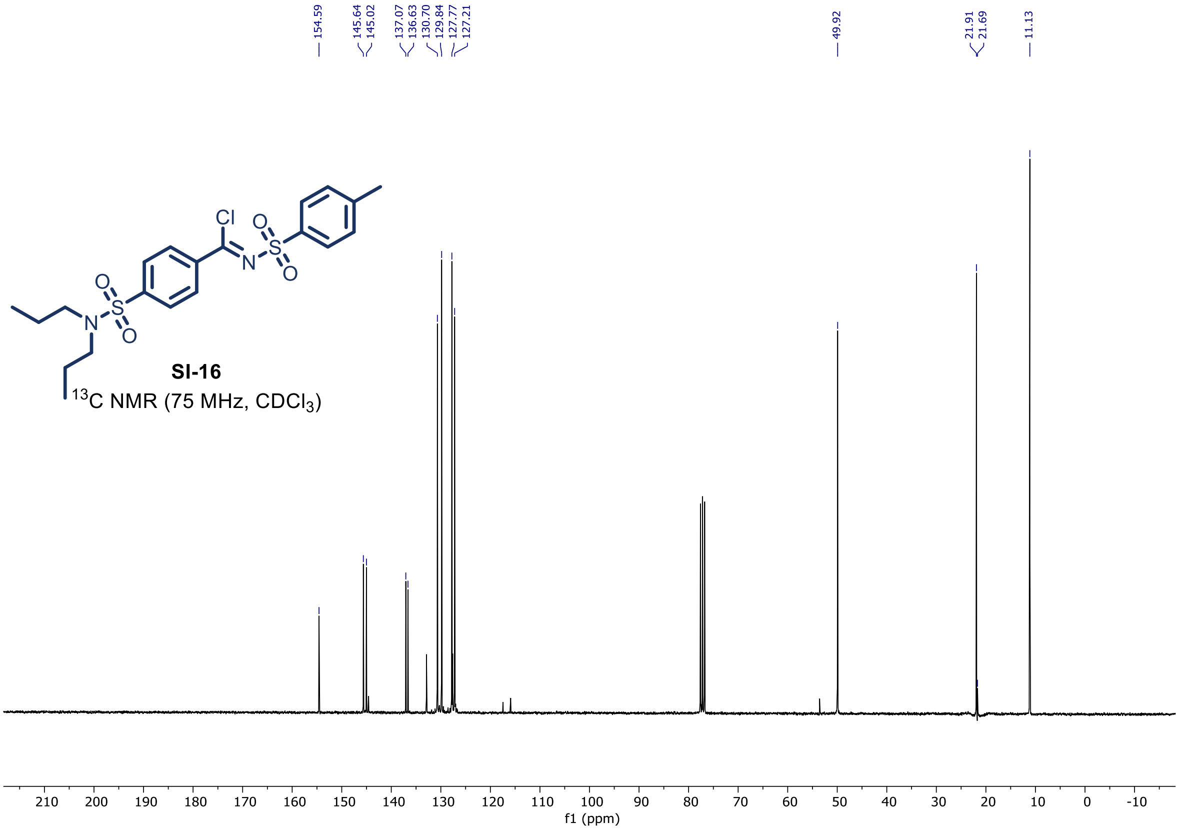

SI-16

^{13}C NMR (75 MHz, $CDCl_3$)

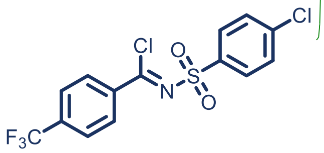

SI-17

^{1}H NMR (300 MHz, $CDCl_3$)

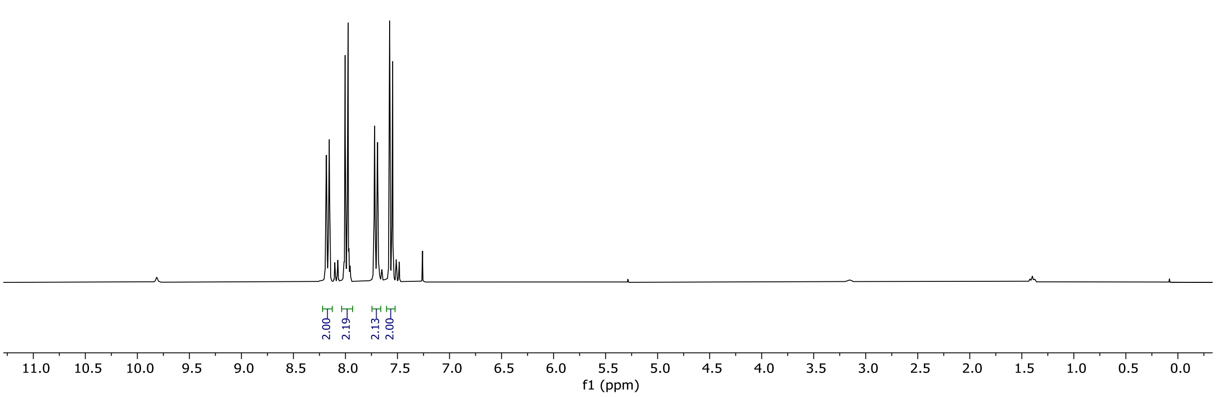

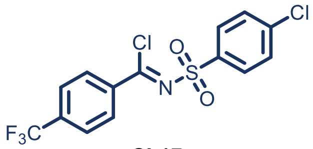

SI-17

^{13}C NMR (75 MHz, $CDCl_3$)

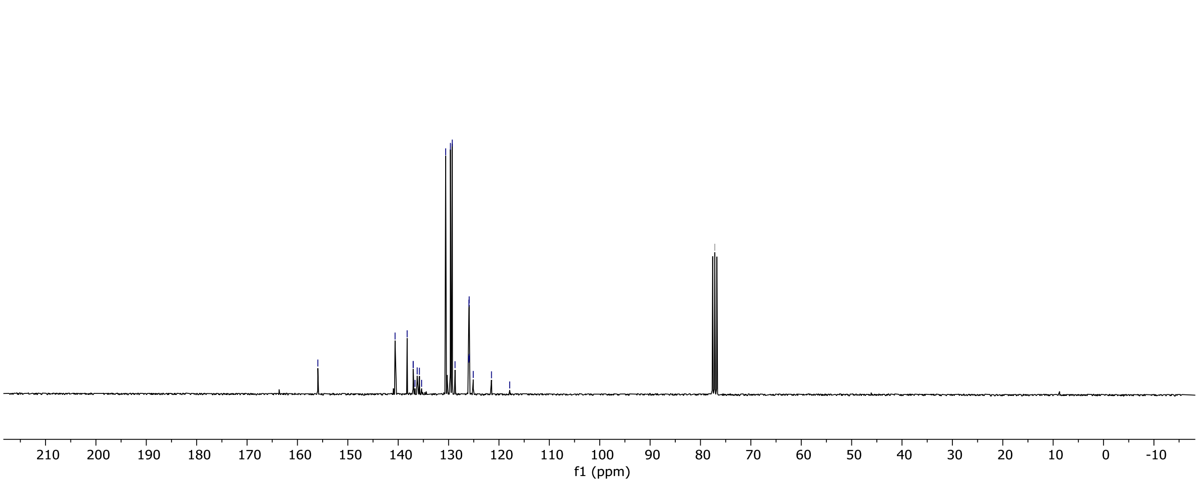

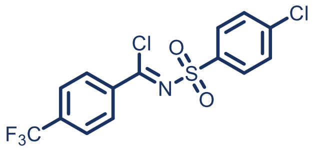

SI-17

^{19}F NMR (282 MHz, $CDCl_3$)

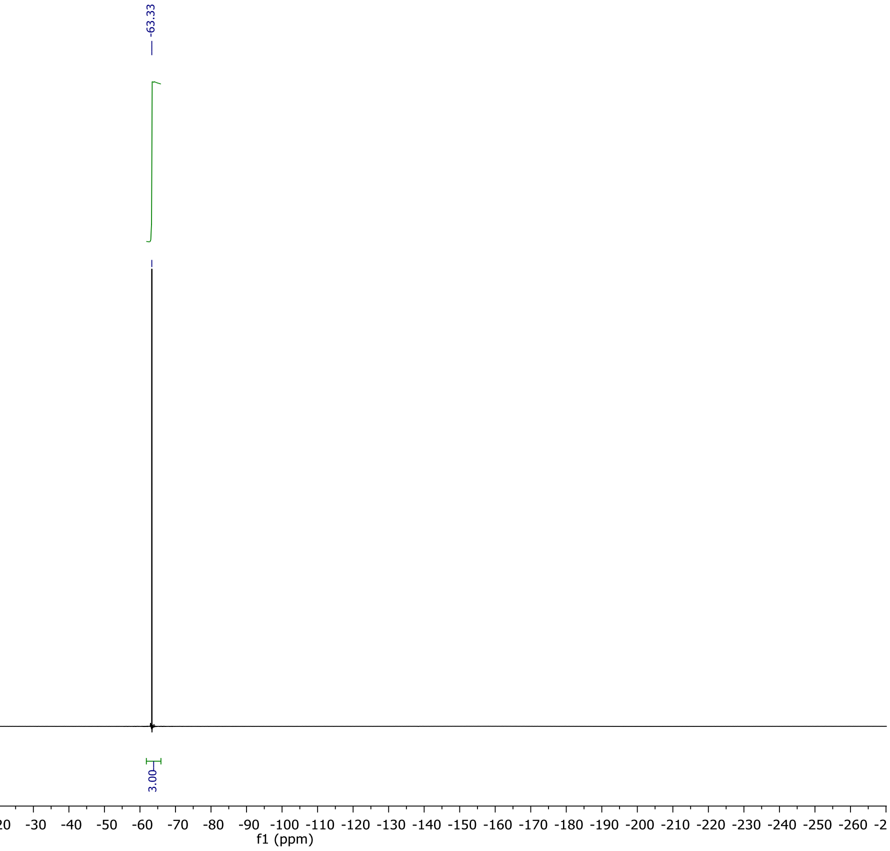

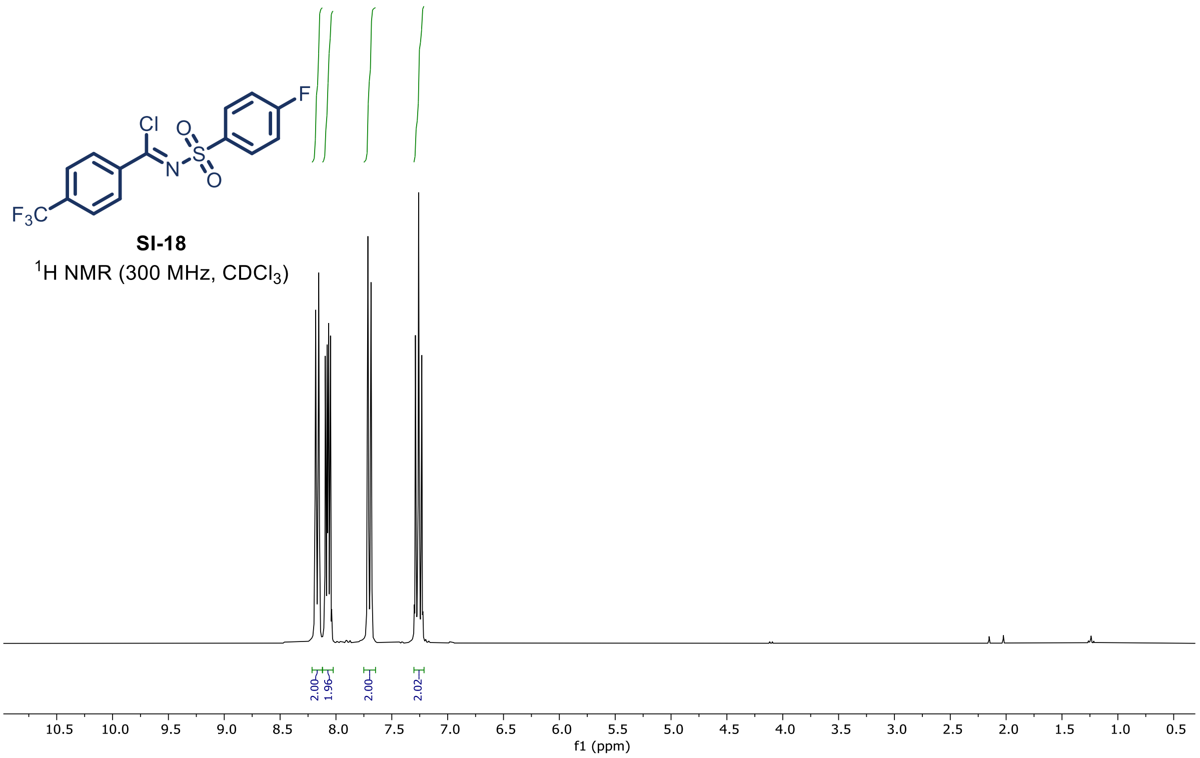
SI-18
^{1}H NMR (300 MHz, $CDCl_3$)
2.00
1.96
2.00
2.02
10.5 10.0 9.5 9.0 8.5 8.0 7.5 7.0 6.5 6.0 5.5 5.0 4.5 4.0 3.5 3.0 2.5 2.0 1.5 1.0 0.5
f1 (ppm)

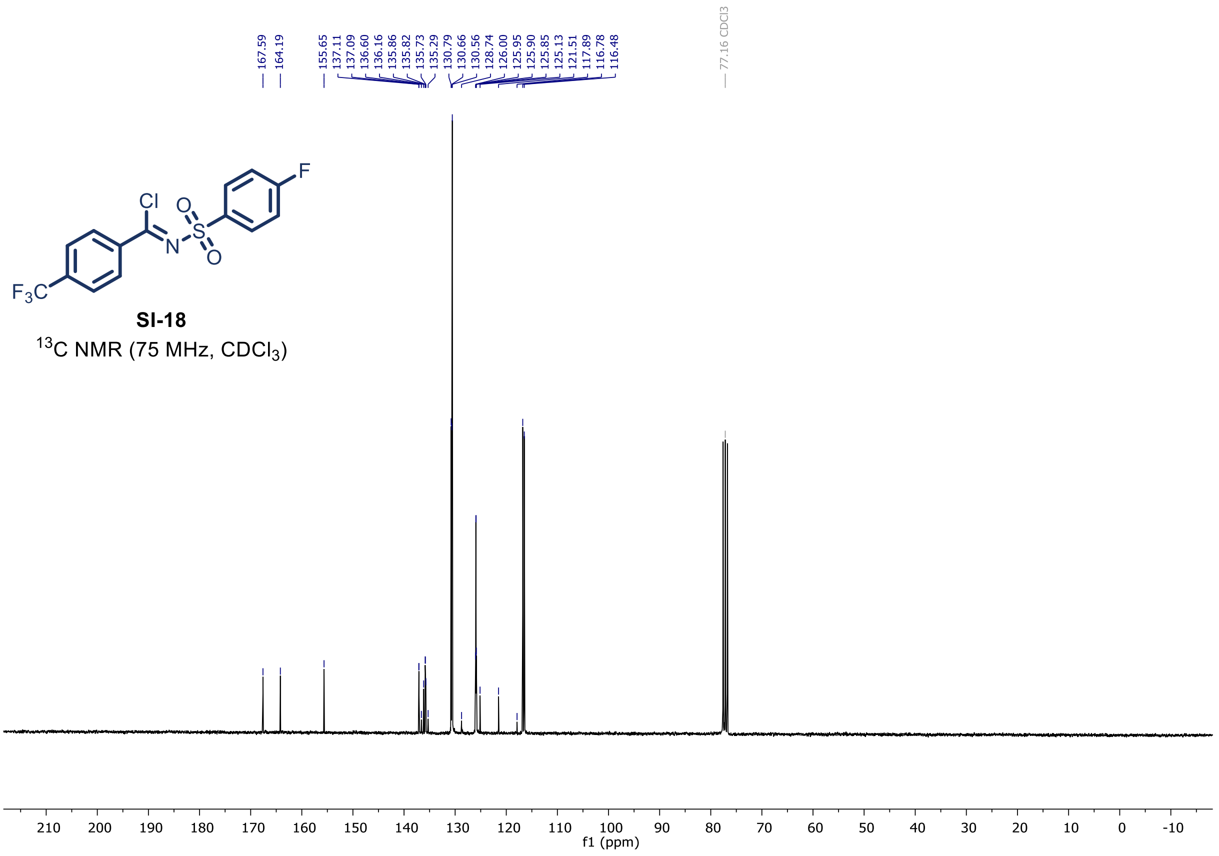
167.59
164.19
155.65
137.11
137.09
136.60
136.16
135.86
135.82
135.73
135.29
130.79
130.66
130.56
128.74
126.00
125.95
125.90
125.85
125.13
121.51
117.89
116.78
116.48
77.16 CDCl3
SI-18
^{13}C NMR (75 MHz, $CDCl_3$)
210 200 190 180 170 160 150 140 130 120 110 100 90 80 70 60 50 40 30 20 10 0 -10
f1 (ppm)

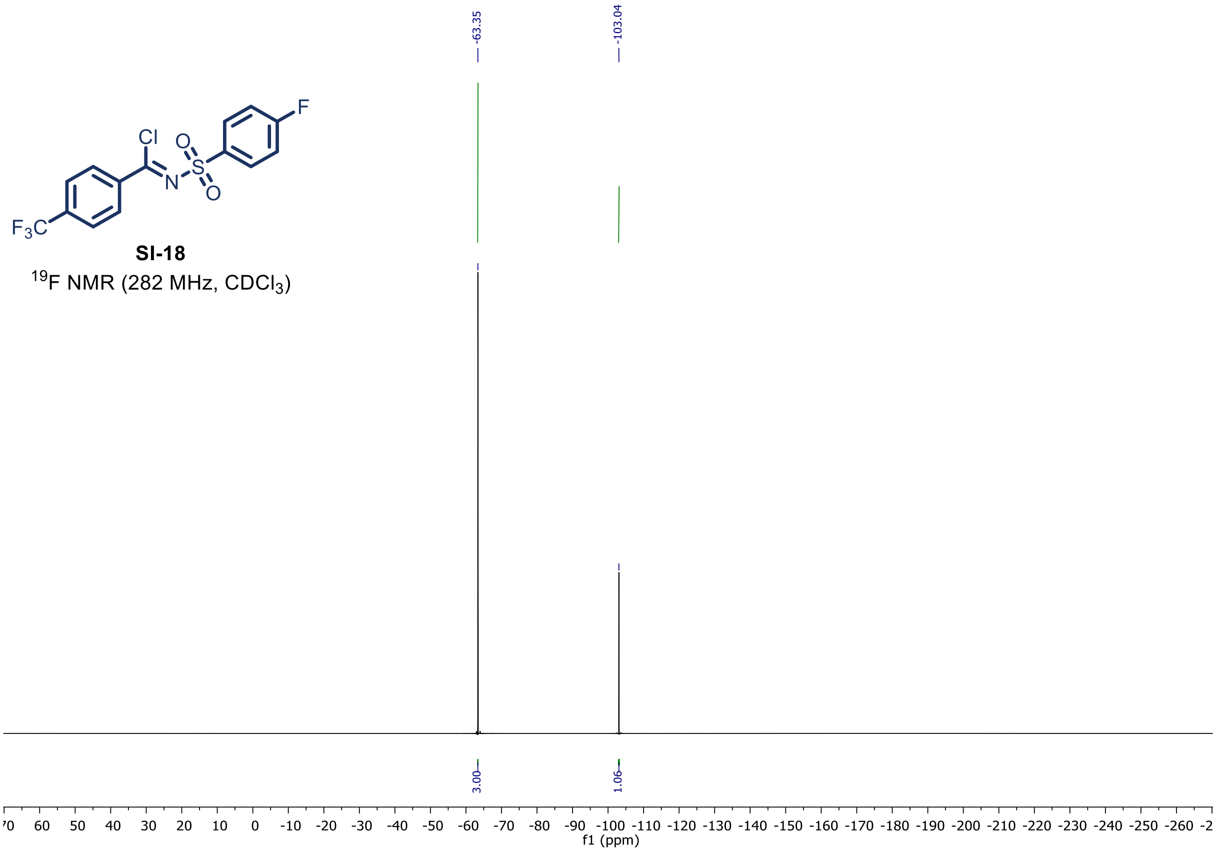

SI-18

^{19}F NMR (282 MHz, $CDCl_3$)

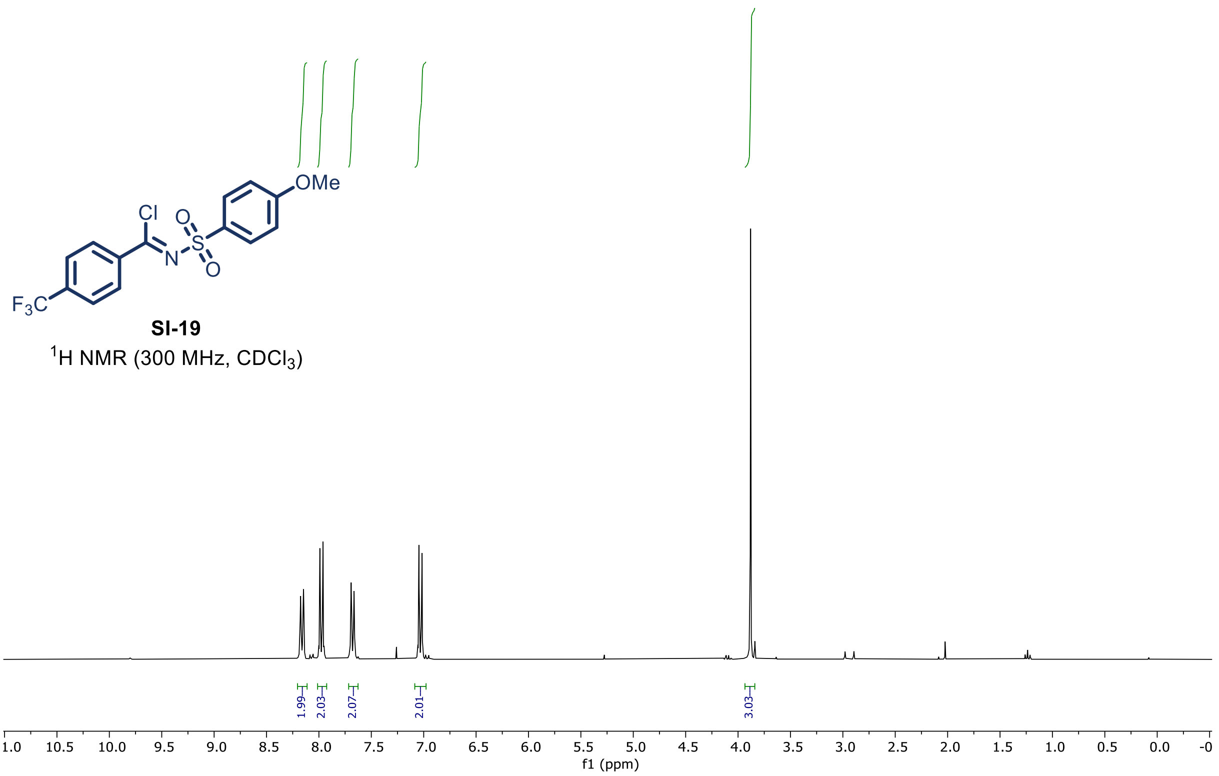
OMe
Cl
O
S
N
O
F3C
SI-19
1H NMR (300 MHz, CDCl3)
1.99
2.03
2.07
2.01
3.03
f1 (ppm)

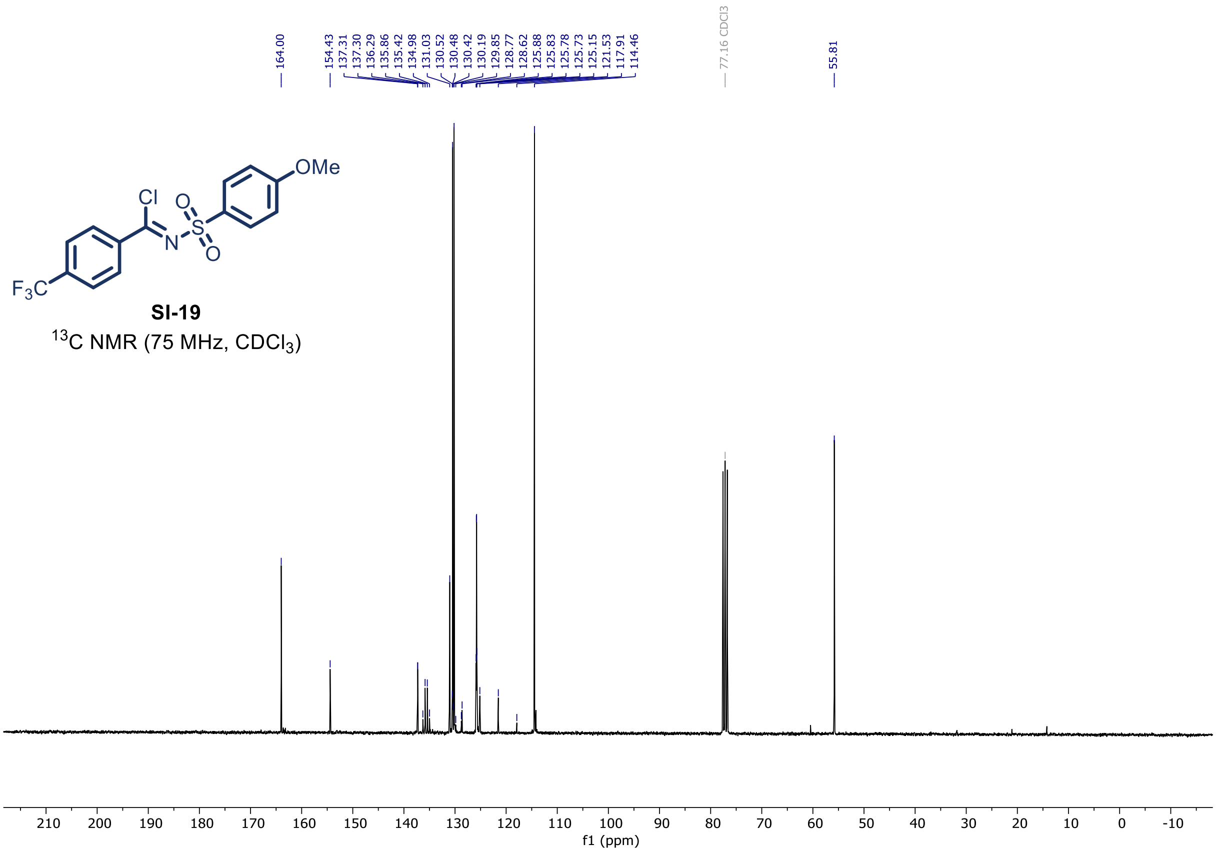
164.00
154.43
137.31
137.30
136.29
135.86
135.42
134.98
131.03
130.52
130.48
130.42
130.19
129.85
128.77
128.62
125.88
125.83
125.78
125.73
125.15
121.53
117.91
114.46
77.16 CDCl3
55.81
OMe
Cl
O
S
N
O
F3C
SI-19
13C NMR (75 MHz, CDCl3)
f1 (ppm)

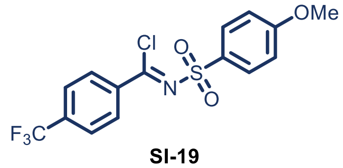

SI-19

^{19}F NMR (282 MHz, $CDCl_3$)

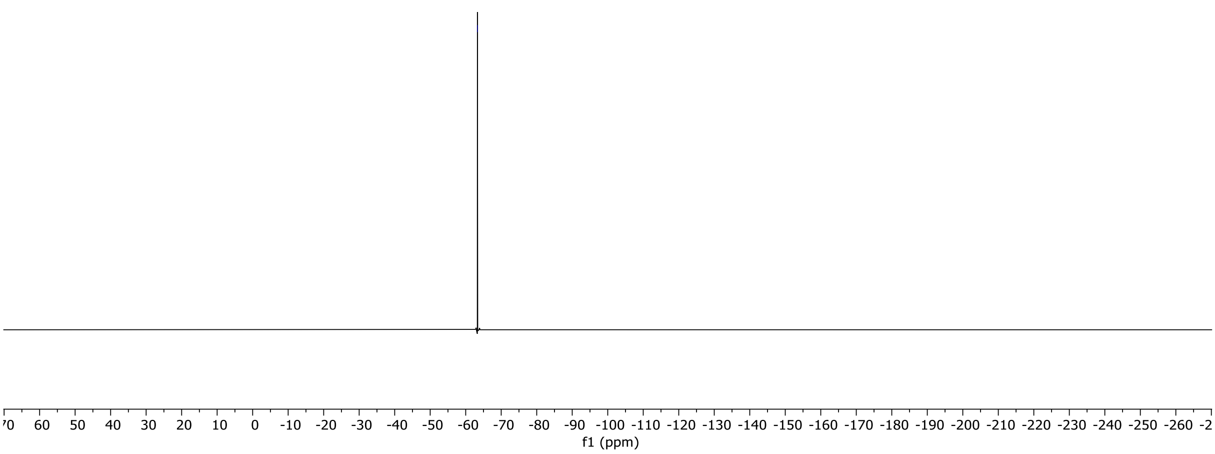

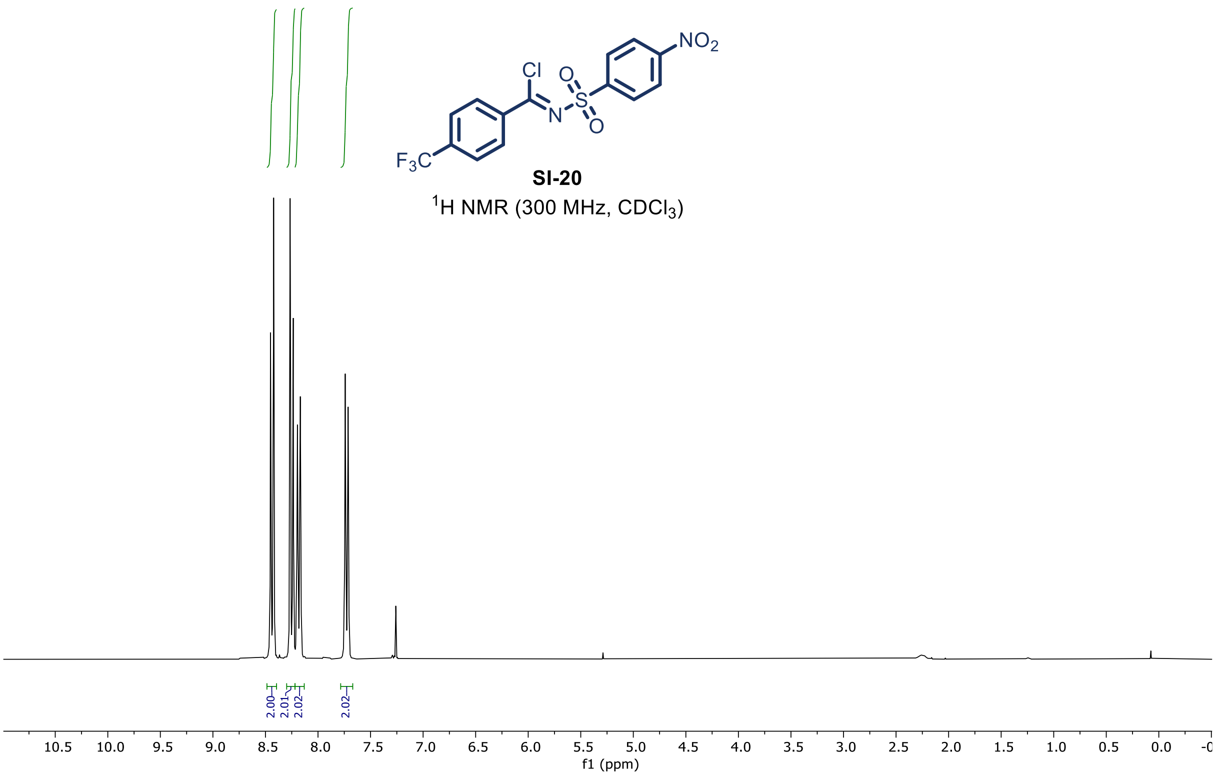
NO2
Cl
O
S
N
O
F3C
SI-20
1H NMR (300 MHz, CDCl3)
2.00
2.01
2.02
2.02
f1 (ppm)

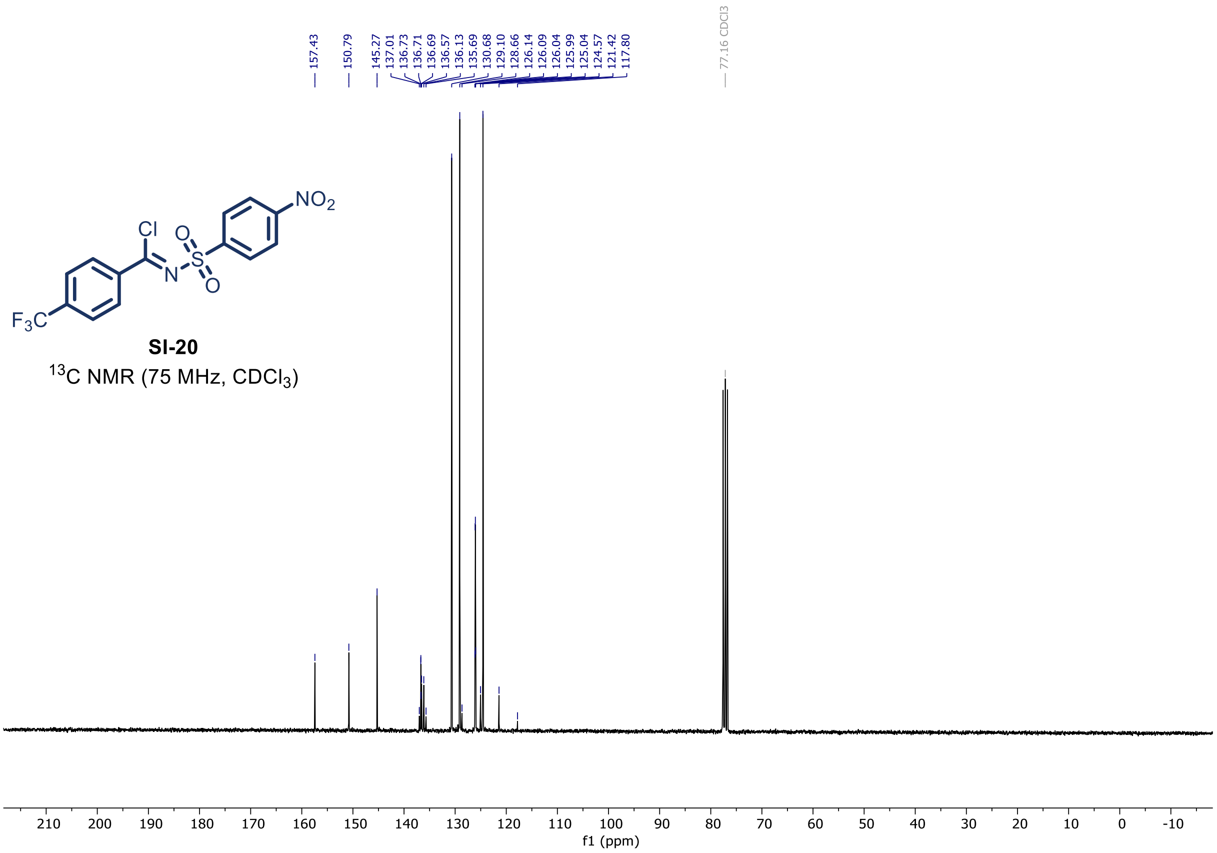
157.43
150.79
145.27
137.01
136.73
136.71
136.69
136.57
136.13
135.69
130.68
129.10
128.66
126.14
126.09
126.04
125.99
125.04
124.57
121.42
117.80
77.16 CDCl3
NO2
Cl
O
S
N
O
F3C
SI-20
13C NMR (75 MHz, CDCl3)
f1 (ppm)

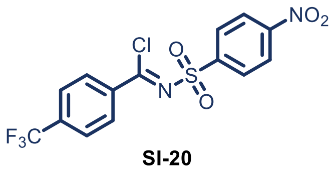

SI-20

^{19}F NMR (282 MHz, $CDCl_3$)

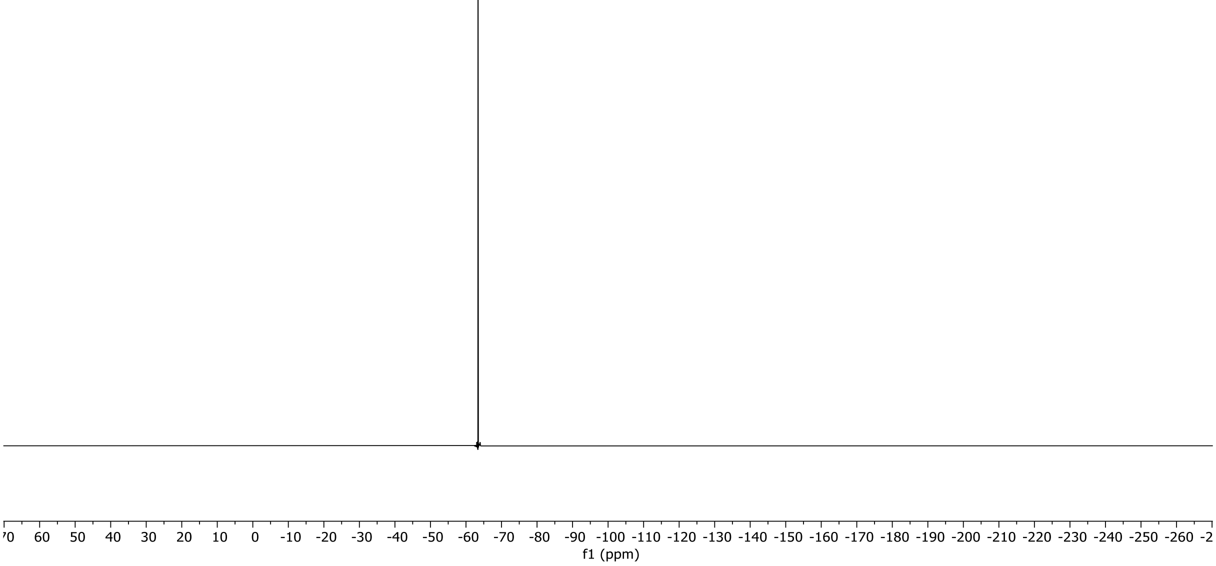

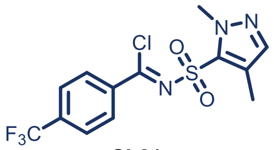

SI-21

^{1}H NMR (300 MHz, $CDCl_3$)

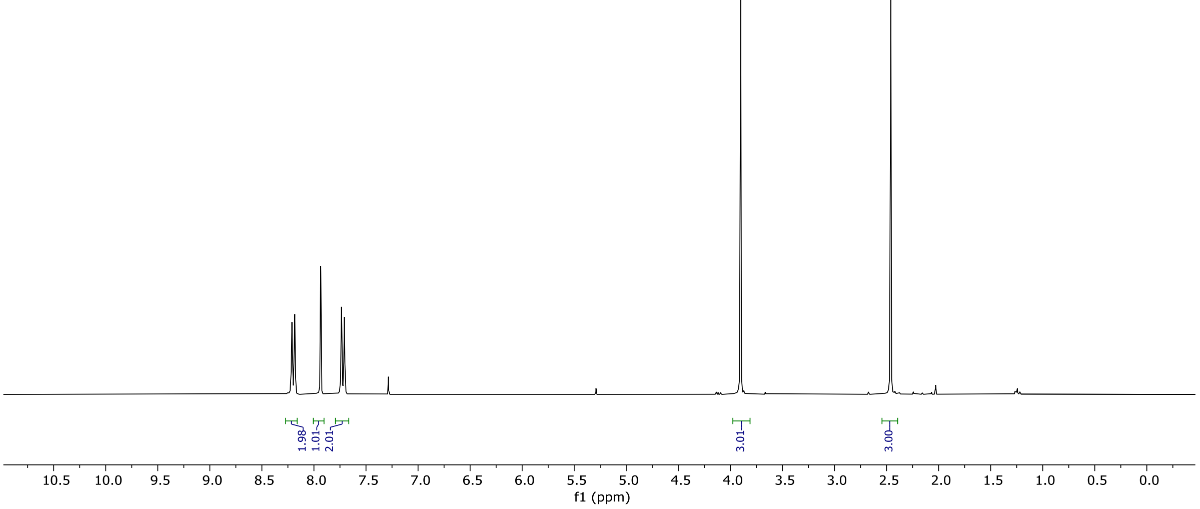

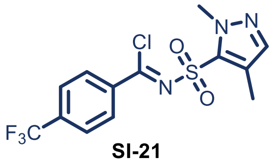

SI-21

^{13}C NMR (75 MHz, $CDCl_3$)

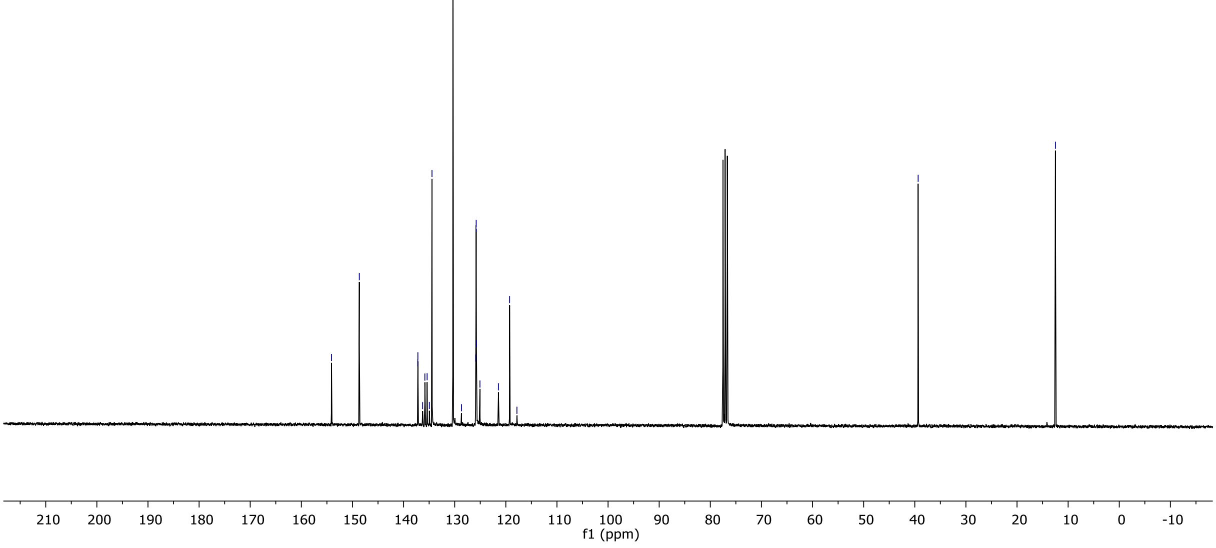

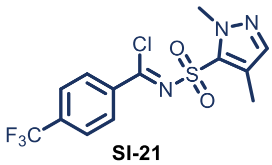

SI-21

^{19}F NMR (282 MHz, $CDCl_3$)

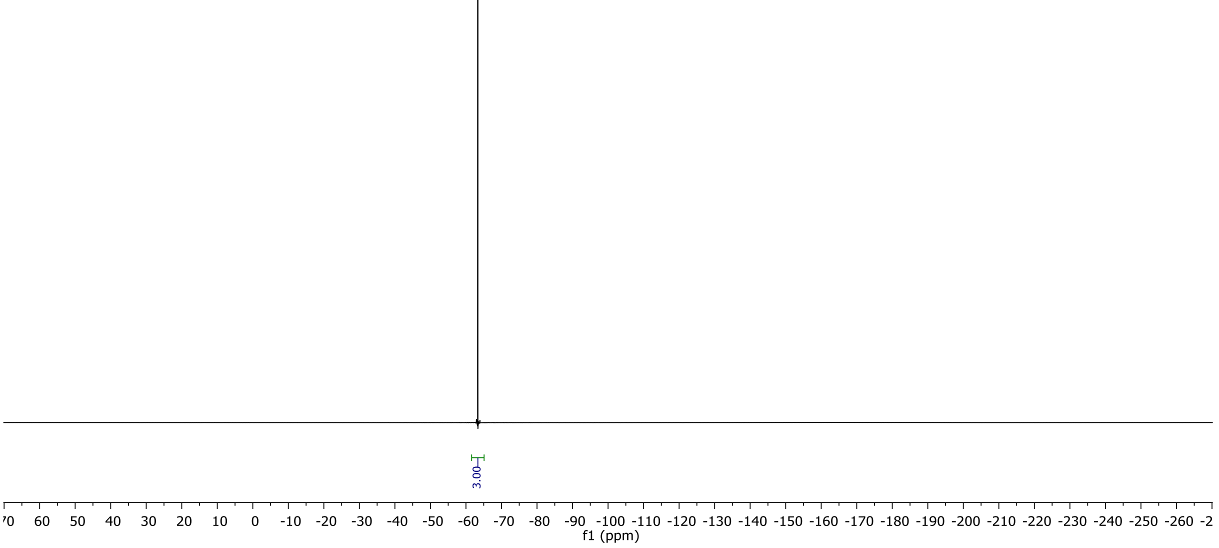

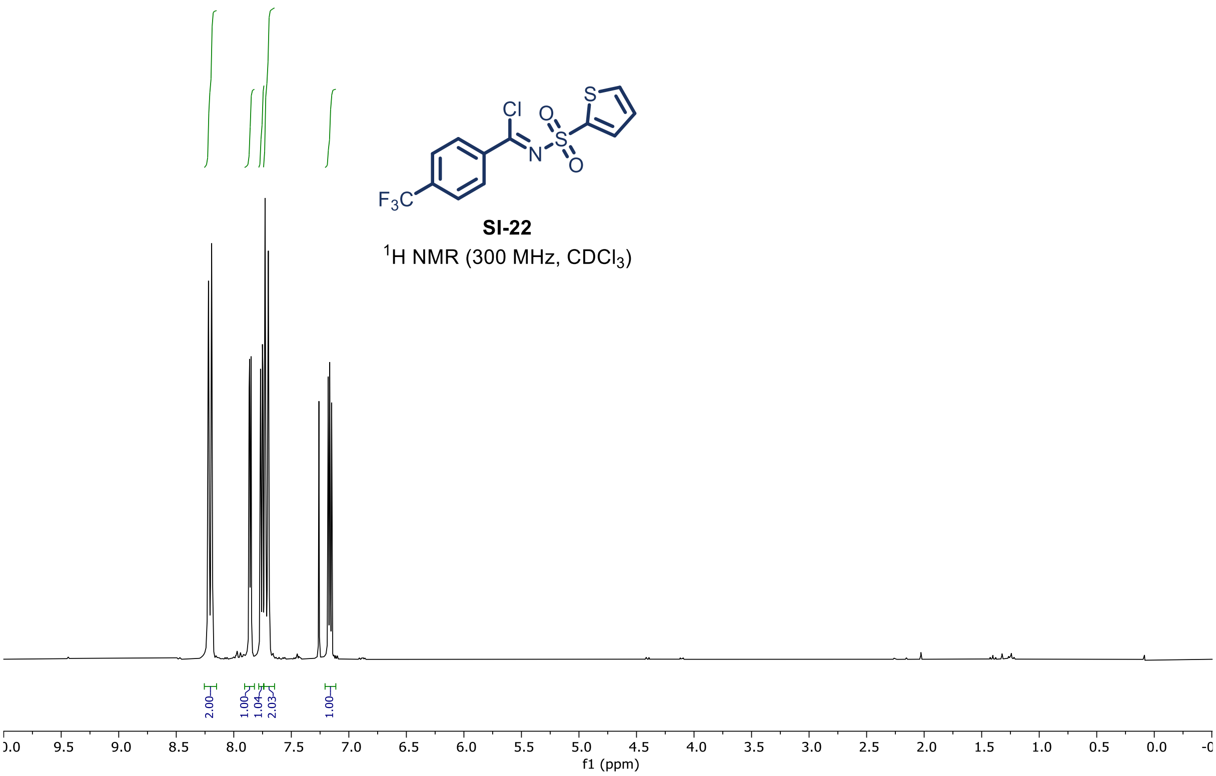

SI-22

^{1}H NMR (300 MHz, $CDCl_3$)

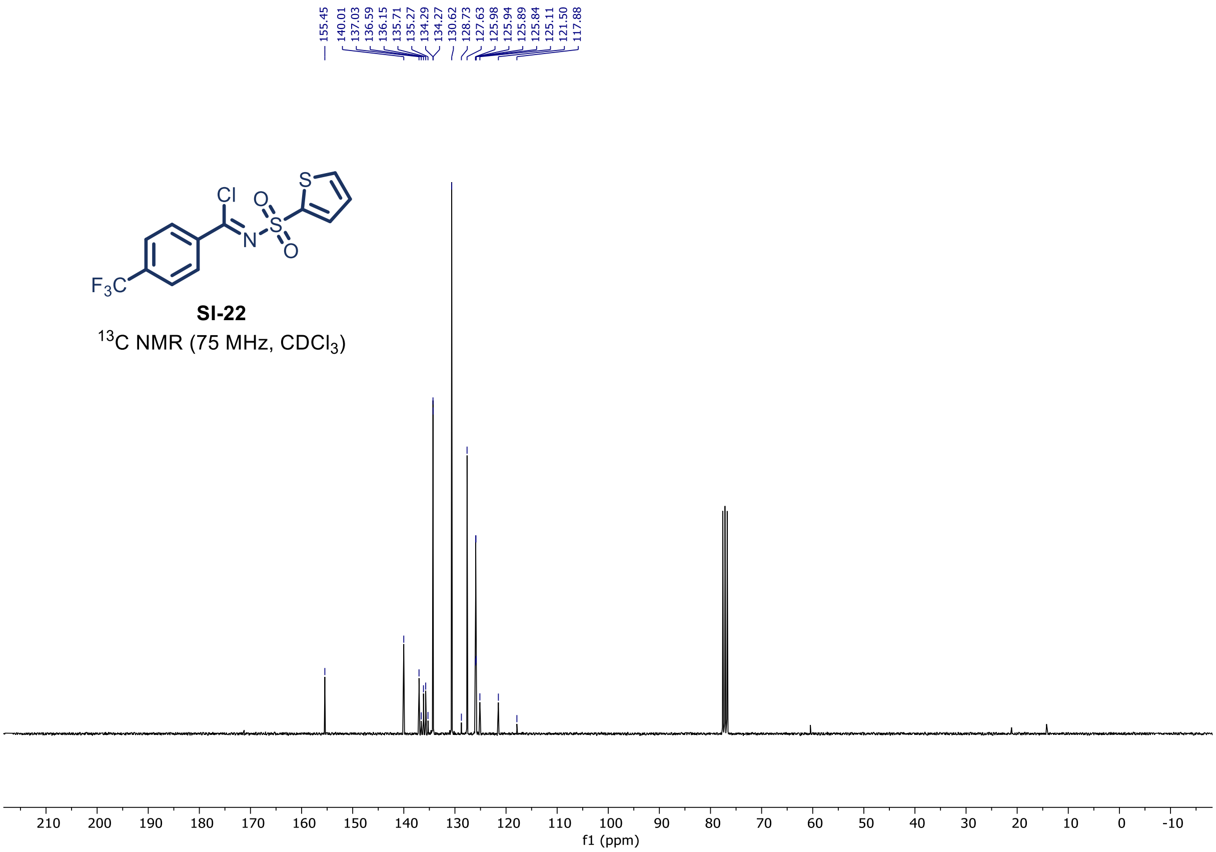

SI-22

^{13}C NMR (75 MHz, $CDCl_3$)

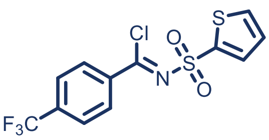

SI-22

^{19}F NMR (282 MHz, $CDCl_3$)

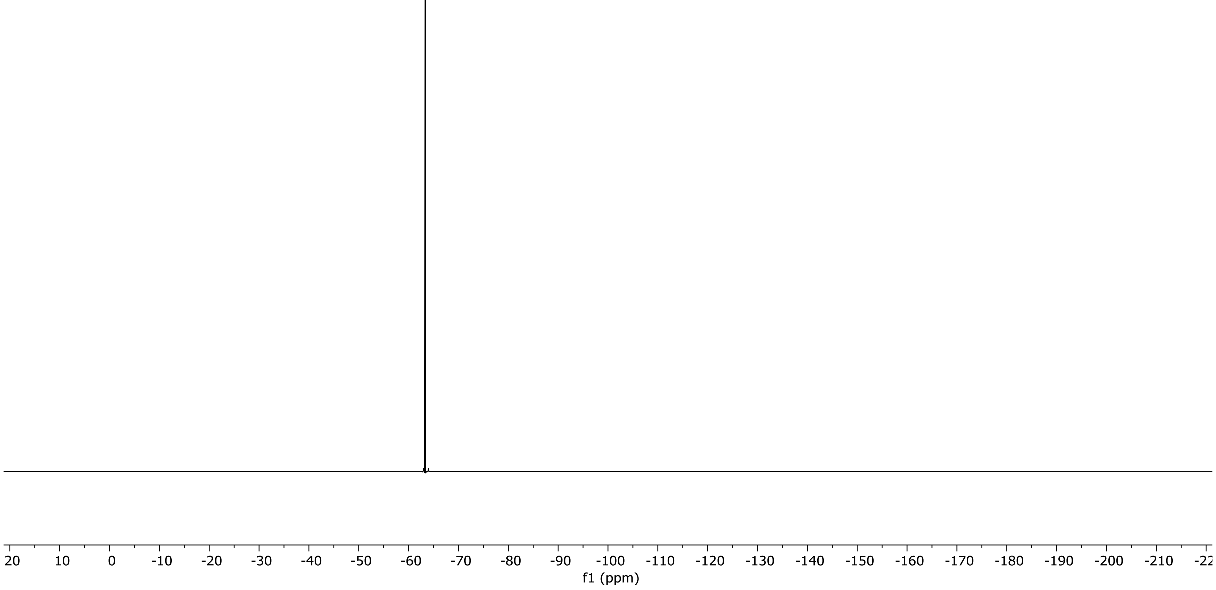

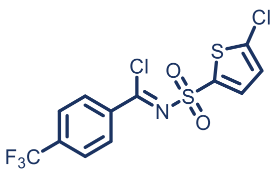

SI-23

^{1}H NMR (300 MHz, $CDCl_3$)

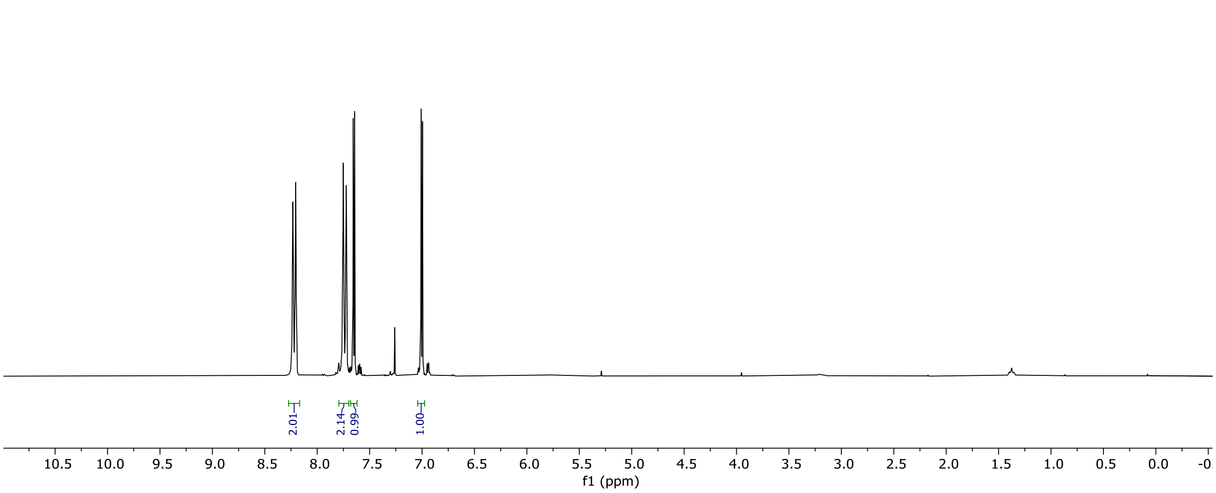

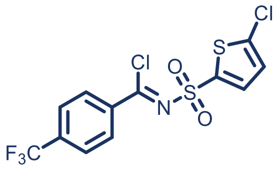

SI-23

^{13}C NMR (75 MHz, $CDCl_3$)

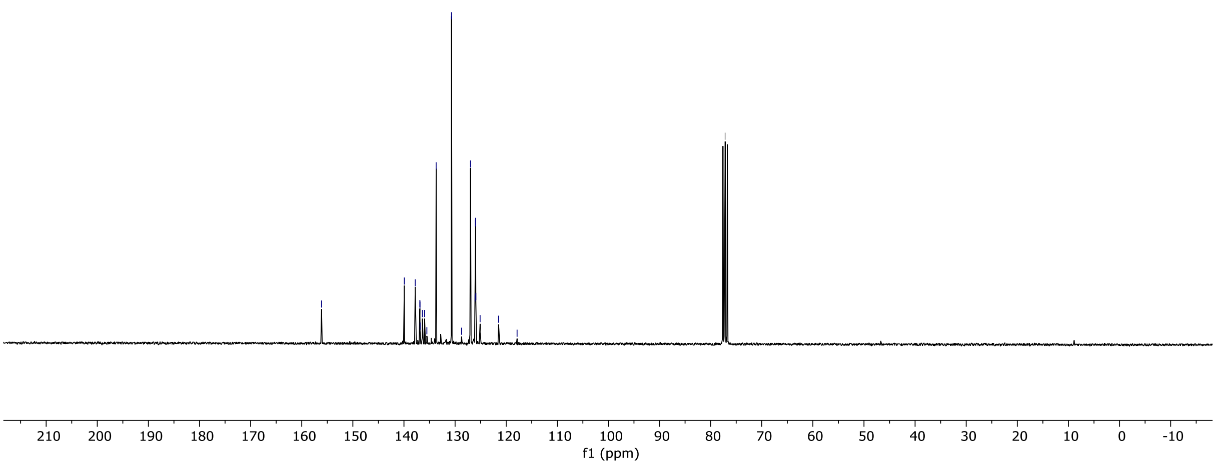

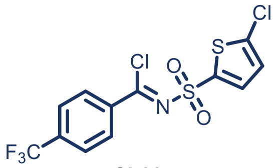

SI-23

^{19}F NMR (282 MHz, $CDCl_3$)

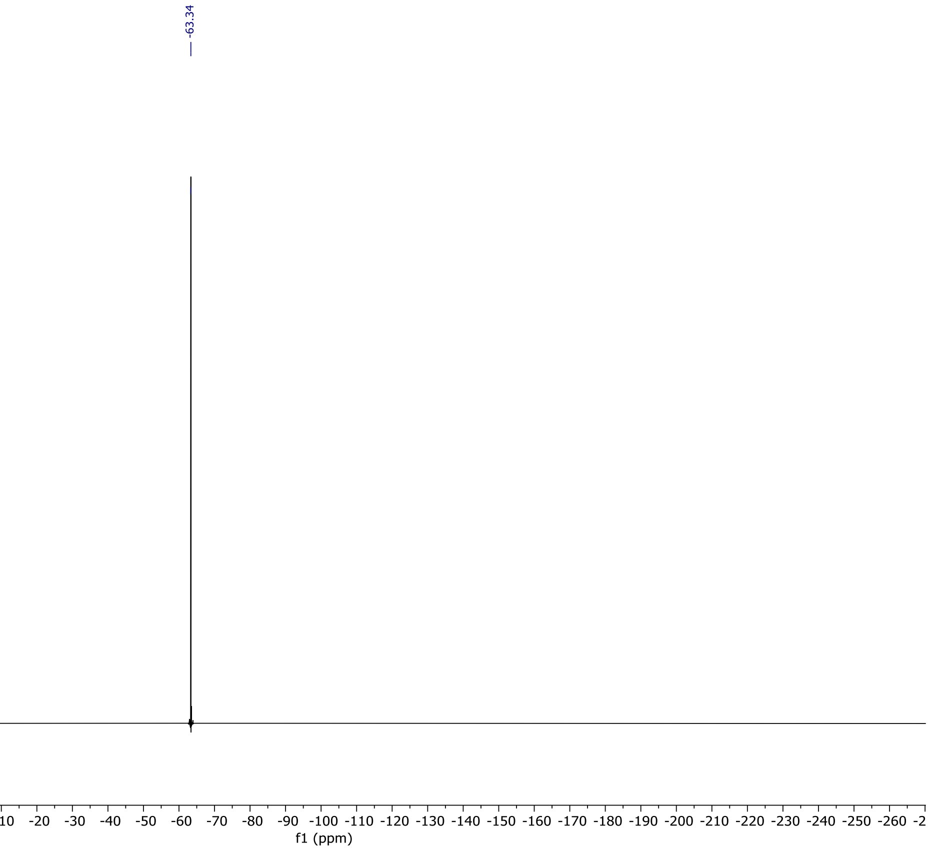

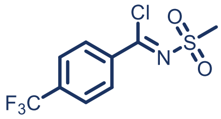

SI-24

^{1}H NMR (300 MHz, $CDCl_3$)

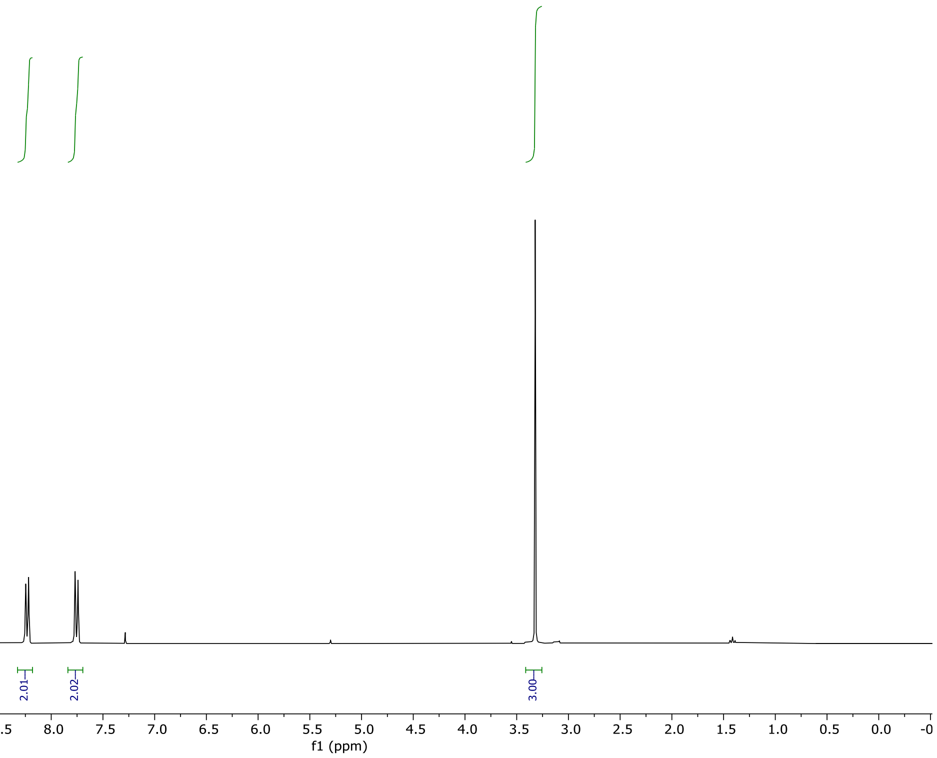

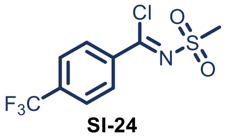

SI-24

^{13}C NMR (75 MHz, $CDCl_3$)

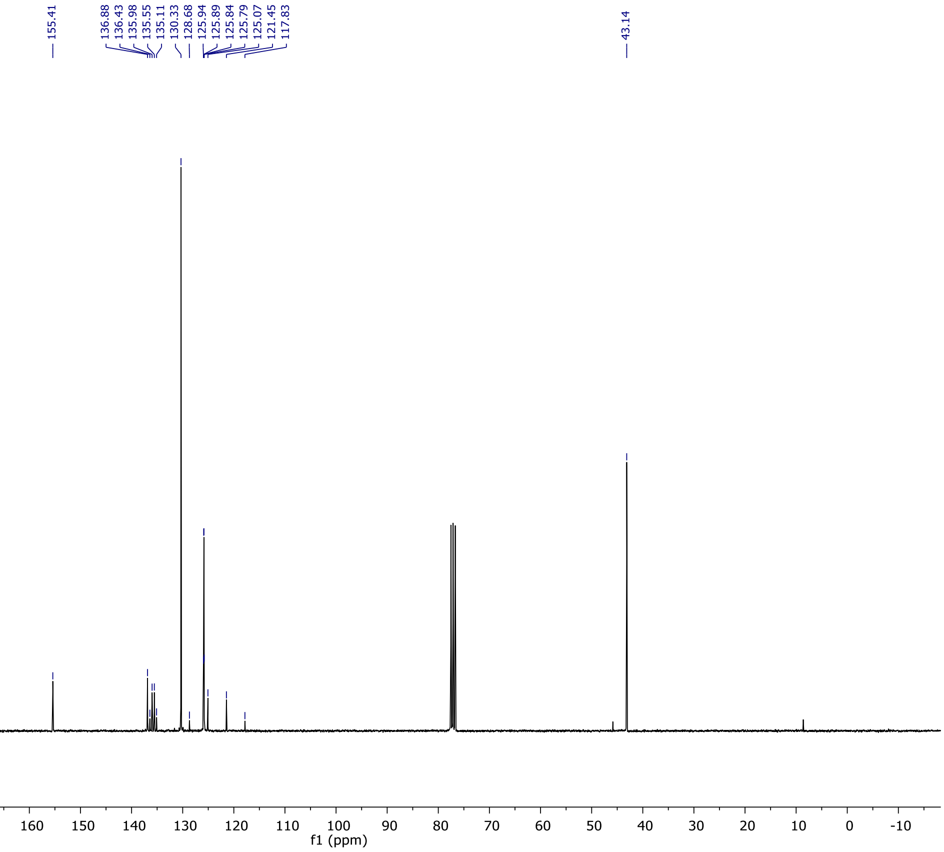

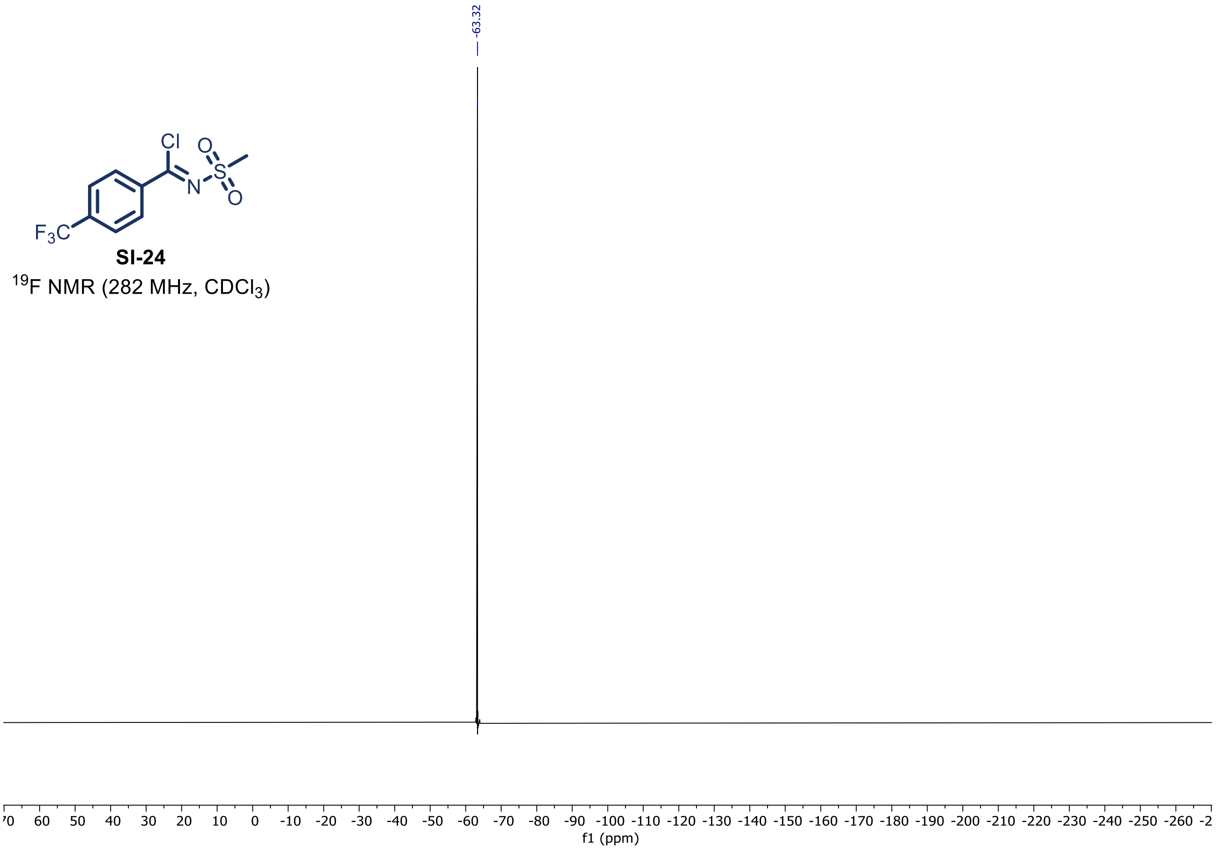

SI-24

^{19}F NMR (282 MHz, $CDCl_3$)

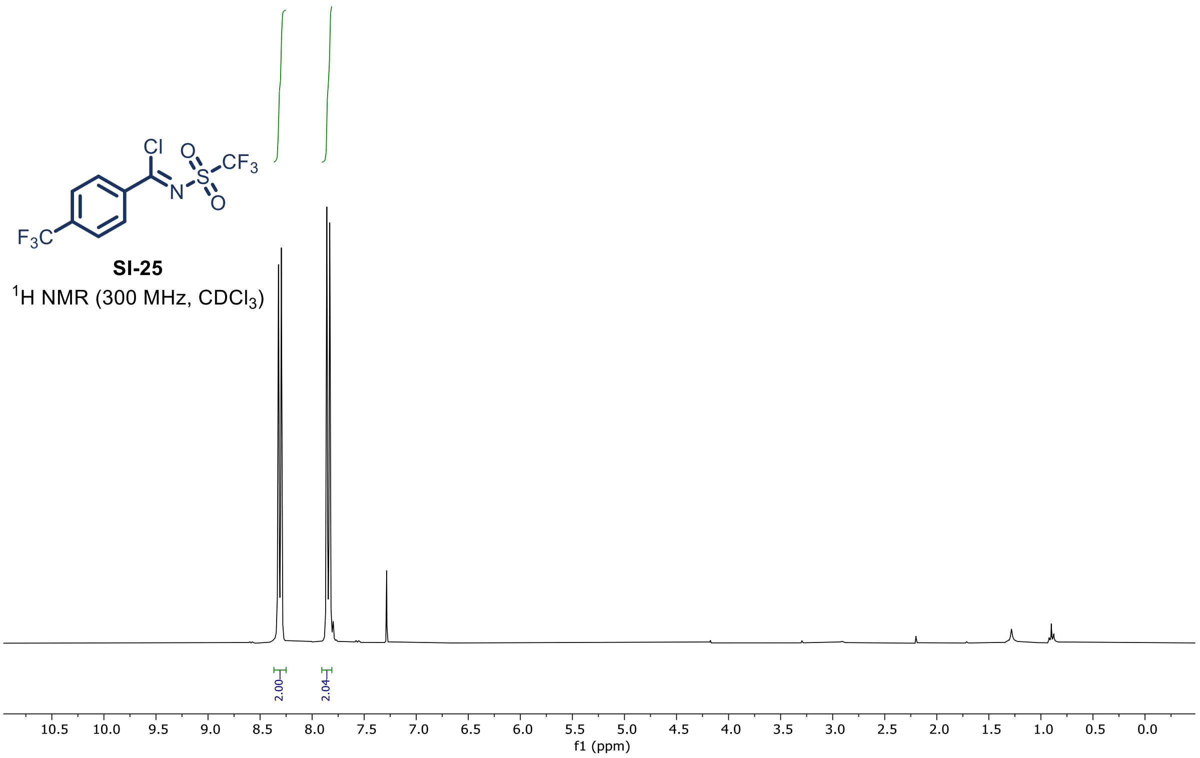

Cl
O
S
CF3
N
O
F3C
SI-25
1H NMR (300 MHz, CDCl3)
2.00
2.04
10.5 10.0 9.5 9.0 8.5 8.0 7.5 7.0 6.5 6.0 5.5 5.0 4.5 4.0 3.5 3.0 2.5 2.0 1.5 1.0 0.5 0.0
f1 (ppm)

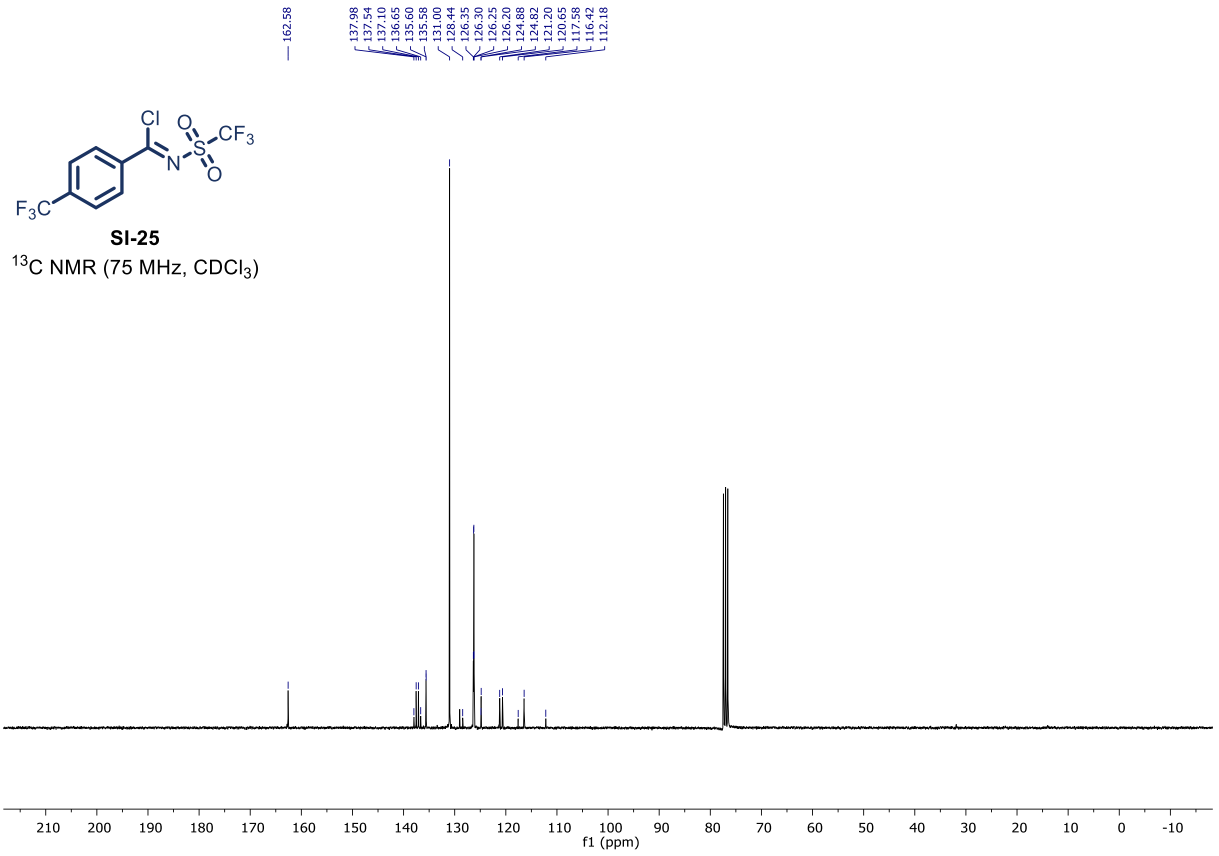

162.58
137.98
137.54
137.10
136.65
135.60
135.58
131.00
128.44
126.35
126.30
126.25
126.20
124.88
124.82
121.20
120.65
117.58
116.42
112.18
Cl
O
S
CF3
N
O
F3C
SI-25
13C NMR (75 MHz, CDCl3)
210 200 190 180 170 160 150 140 130 120 110 100 90 80 70 60 50 40 30 20 10 0 -10
f1 (ppm)

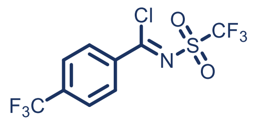

SI-25

^{19}F NMR (282 MHz, $CDCl_3$)

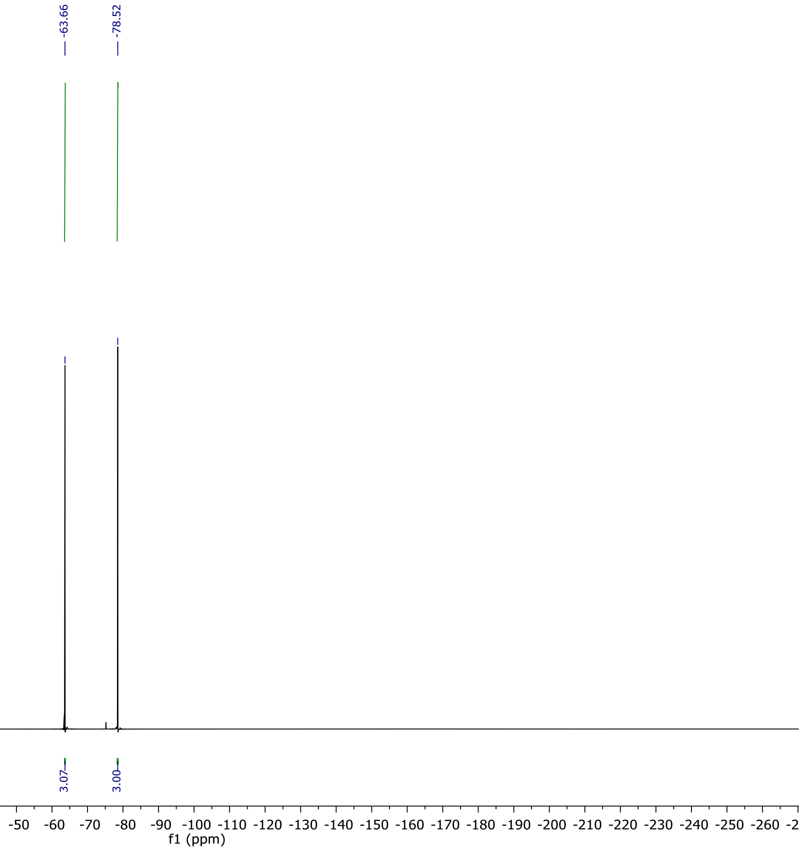

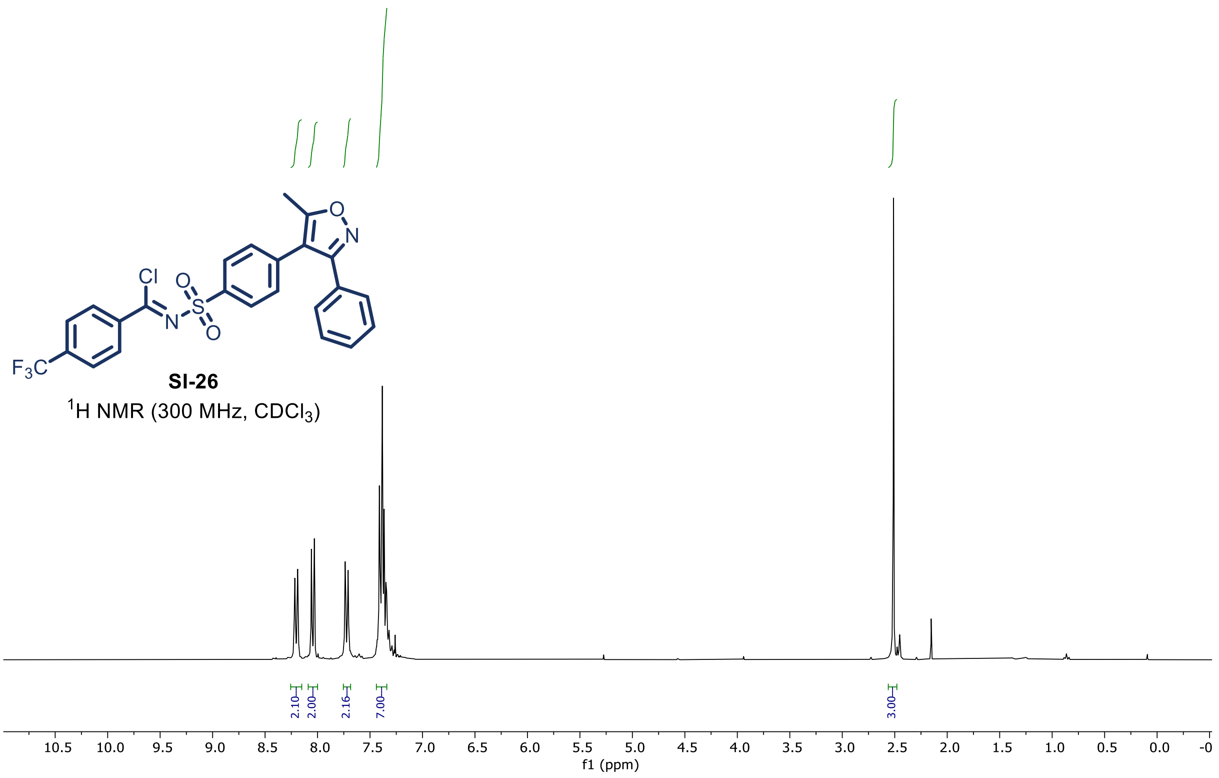
SI-26
^{1}H NMR (300 MHz, $CDCl_3$)
2.10
2.00
2.16
7.00
3.00
10.5 10.0 9.5 9.0 8.5 8.0 7.5 7.0 6.5 6.0 5.5 5.0 4.5 4.0 3.5 3.0 2.5 2.0 1.5 1.0 0.5 0.0
f1 (ppm)

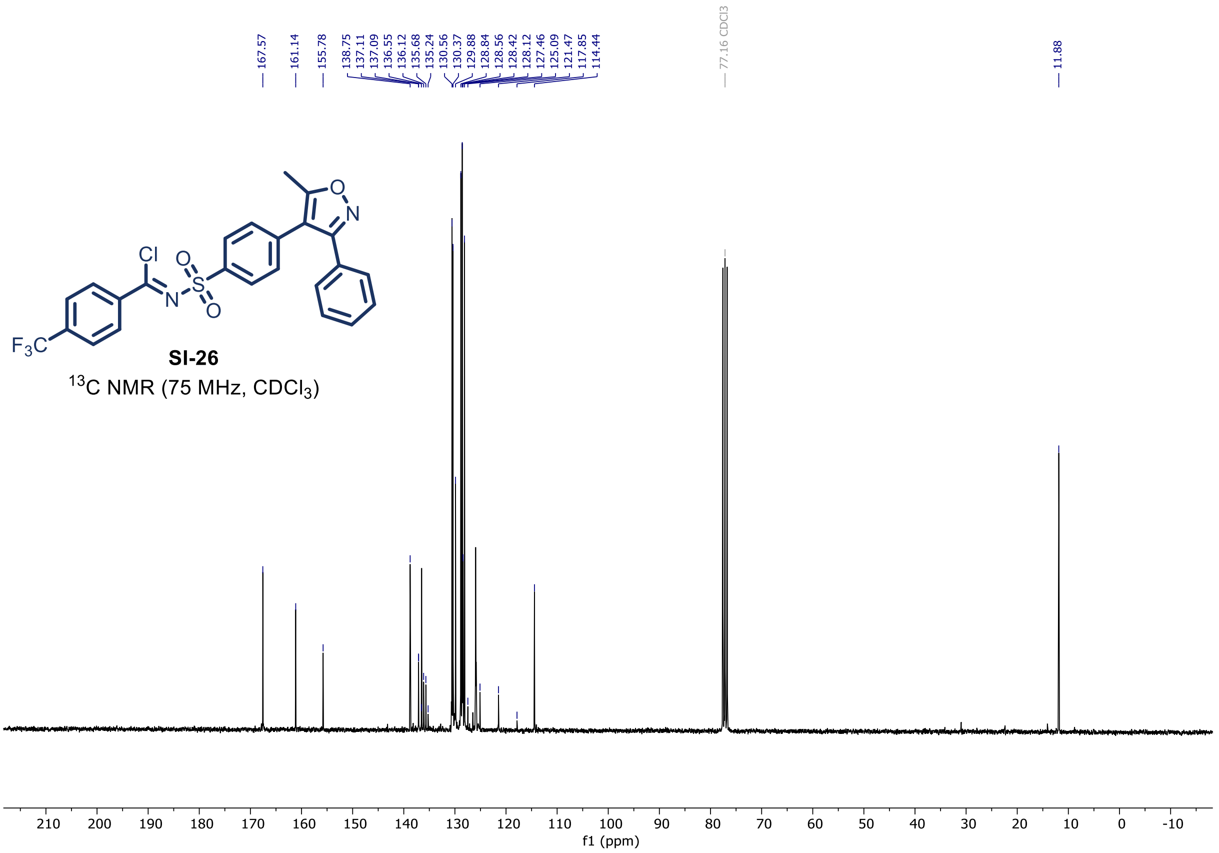
167.57
161.14
155.78
138.75
137.11
137.09
136.55
136.12
135.68
135.24
130.56
130.37
129.88
128.84
128.56
128.42
128.12
127.46
125.09
121.47
117.85
114.44
77.16 CDCl3
11.88
SI-26
^{13}C NMR (75 MHz, $CDCl_3$)
210 200 190 180 170 160 150 140 130 120 110 100 90 80 70 60 50 40 30 20 10 0 -10
f1 (ppm)

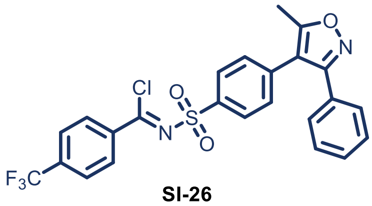

SI-26

^{19}F NMR (282 MHz, $CDCl_3$)

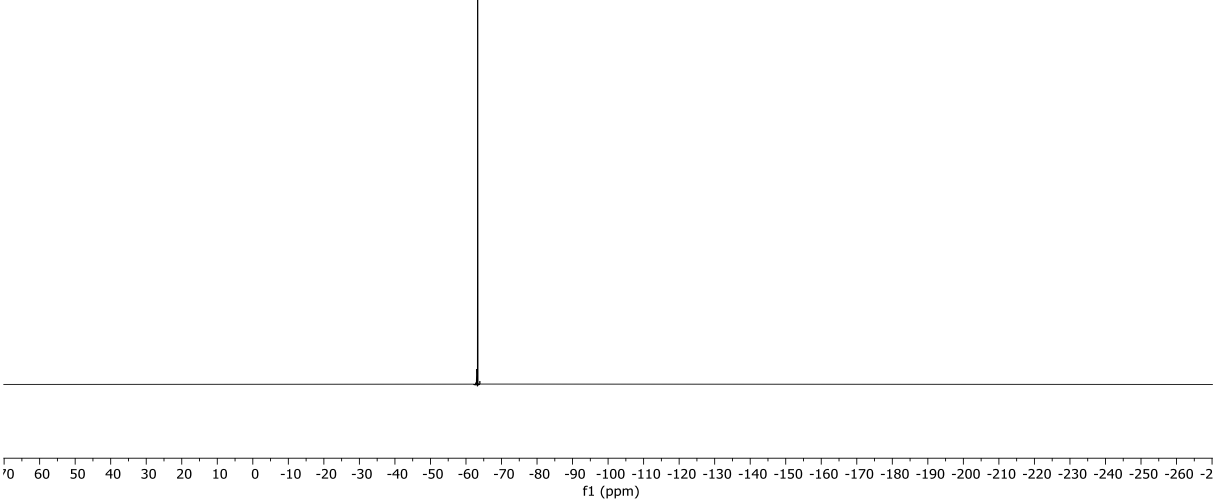

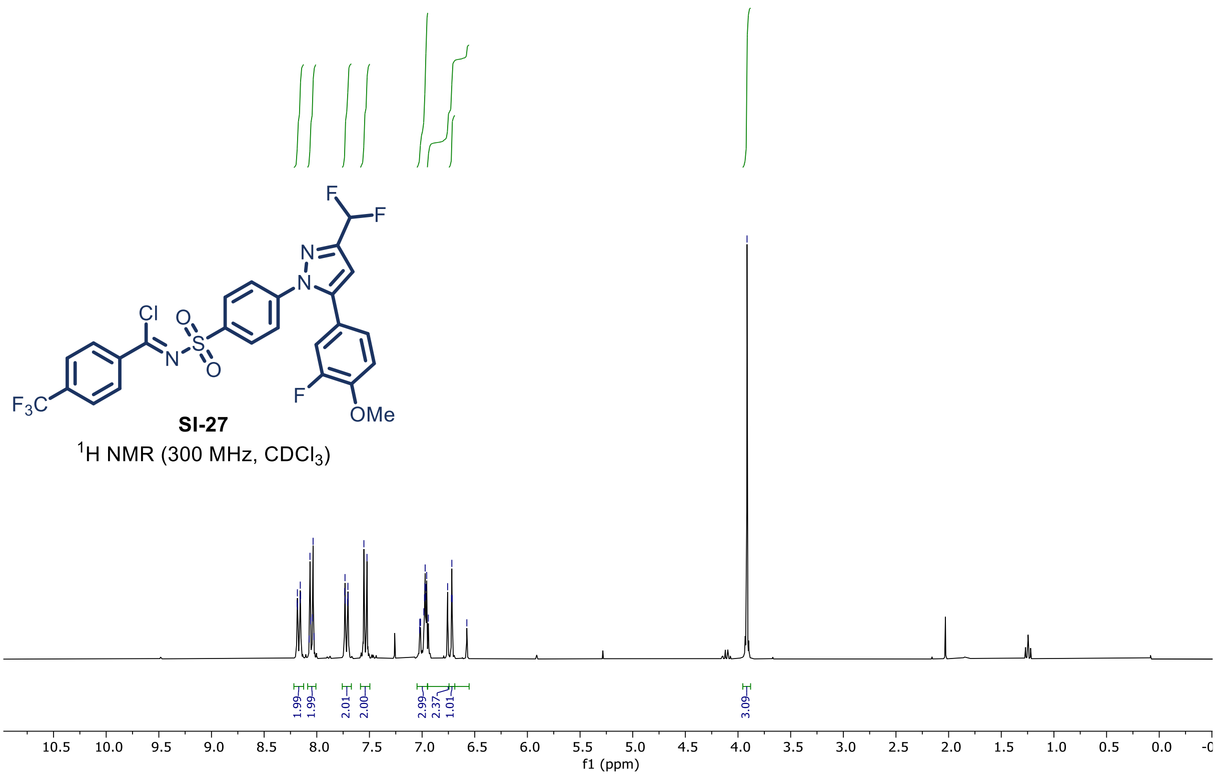
SI-27
^{1}H NMR (300 MHz, CDCl$_3$)
1.99
1.99
2.01
2.00
2.99
2.37
1.01
3.09
f1 (ppm)

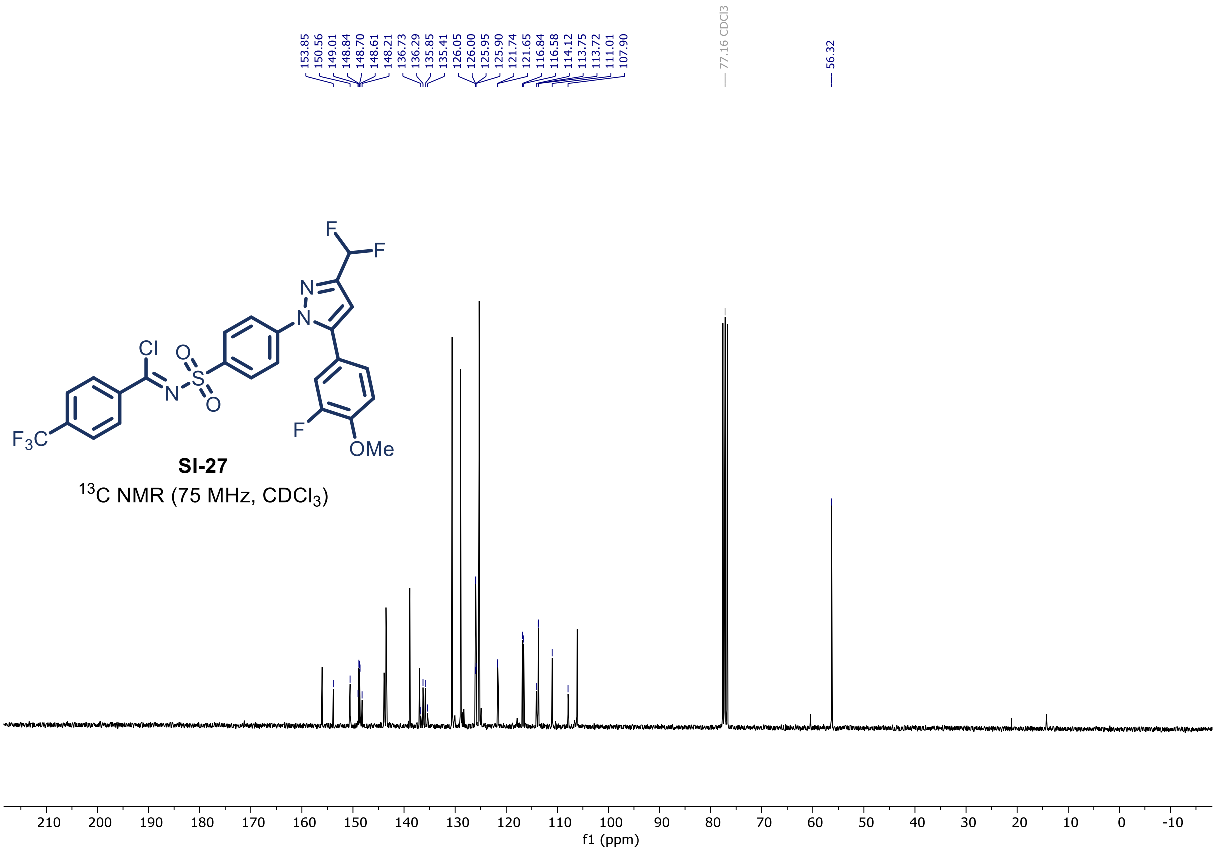
153.85
150.56
149.01
148.84
148.70
148.61
148.21
136.73
136.29
135.85
135.41
126.05
126.00
125.95
125.90
121.74
121.65
116.84
116.58
114.12
113.75
113.72
111.01
107.90
77.16 CDCl3
56.32
SI-27
^{13}C NMR (75 MHz, CDCl$_3$)
f1 (ppm)

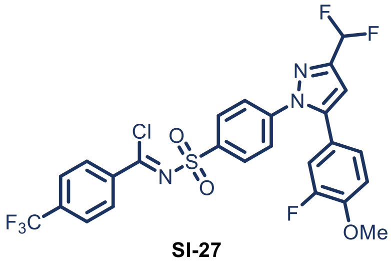

SI-27

^{19}F NMR (282 MHz, $CDCl_3$)

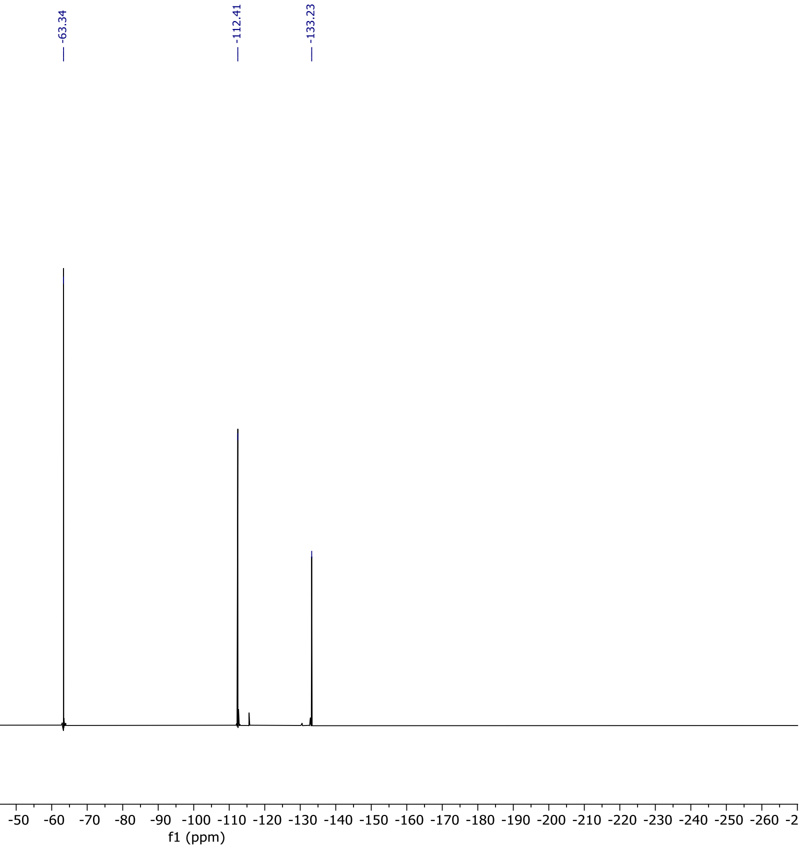

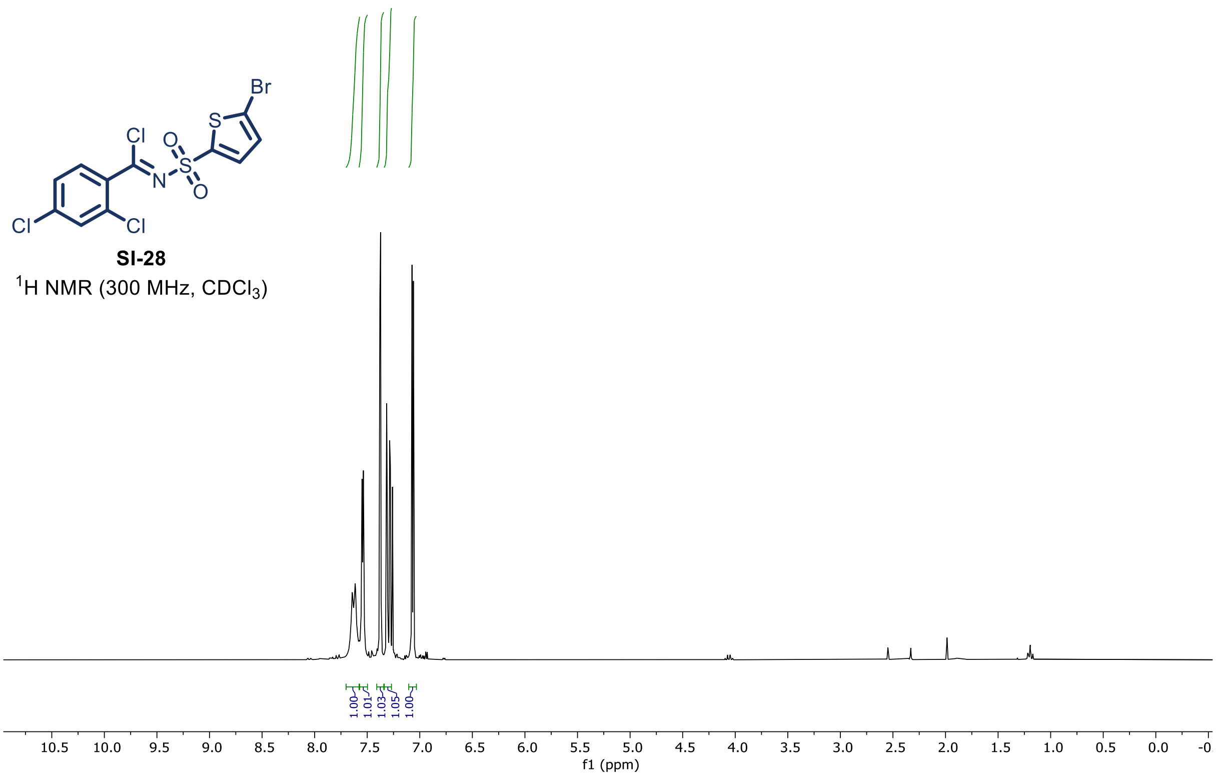
Br
S
Cl
O
N
S
O
Cl
Cl
SI-28
^{1}H NMR (300 MHz, $CDCl_3$)
1.00
1.01
1.03
1.05
1.00
10.5 10.0 9.5 9.0 8.5 8.0 7.5 7.0 6.5 6.0 5.5 5.0 4.5 4.0 3.5 3.0 2.5 2.0 1.5 1.0 0.5 0.0 -0
f1 (ppm)

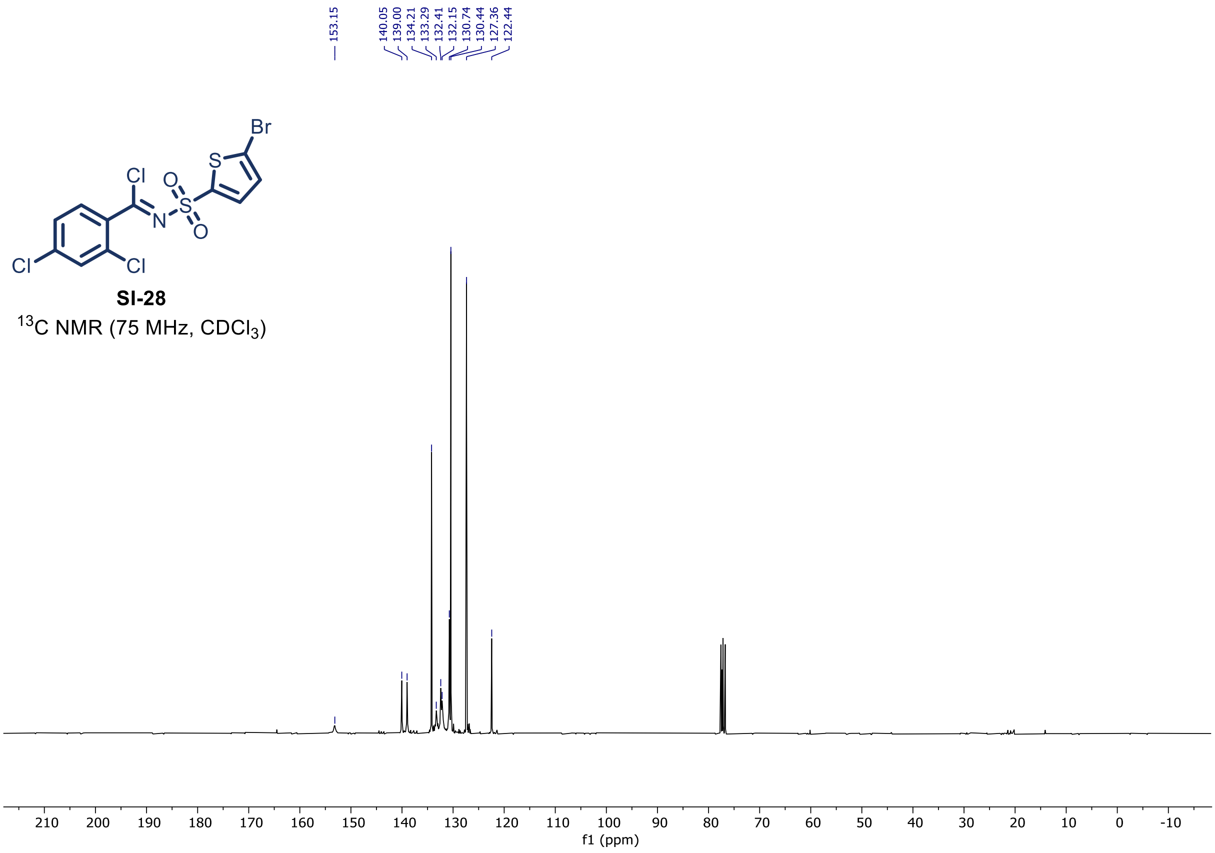
153.15
140.05
139.00
134.21
133.29
132.41
132.15
130.74
130.44
127.36
122.44
Br
S
Cl
O
N
S
O
Cl
Cl
SI-28
^{13}C NMR (75 MHz, $CDCl_3$)
210 200 190 180 170 160 150 140 130 120 110 100 90 80 70 60 50 40 30 20 10 0 -10
f1 (ppm)

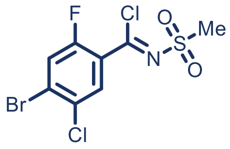

47

^{1}H NMR (300 MHz, $CDCl_3$)

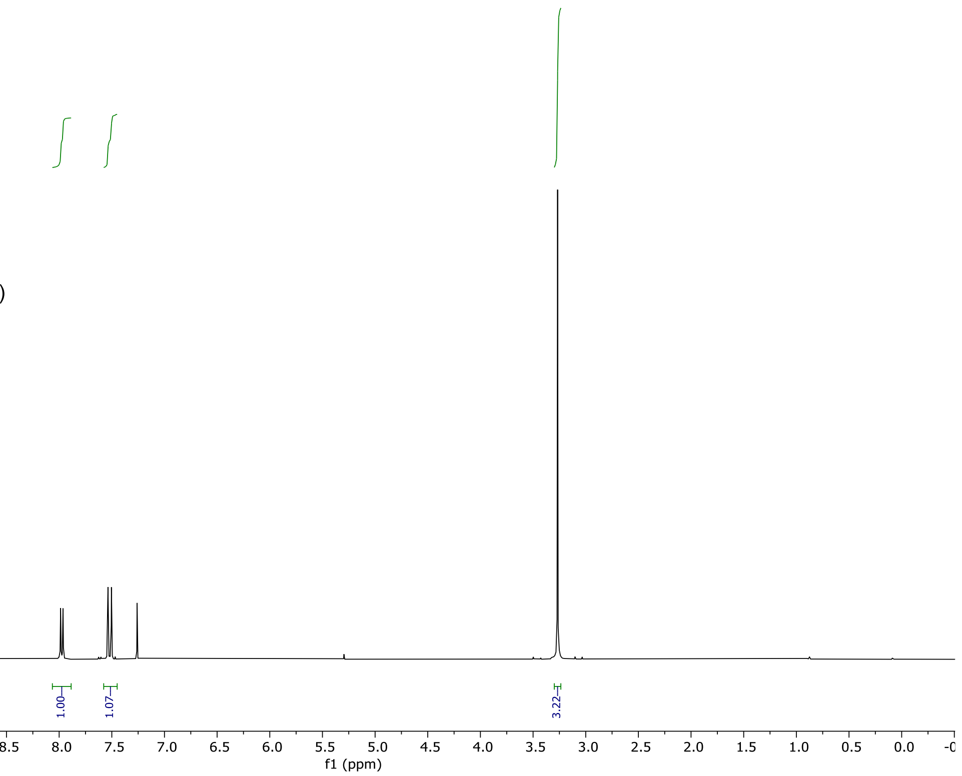

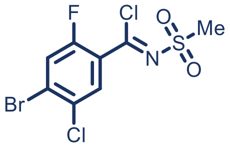

47

^{13}C NMR (75 MHz, $CDCl_3$)

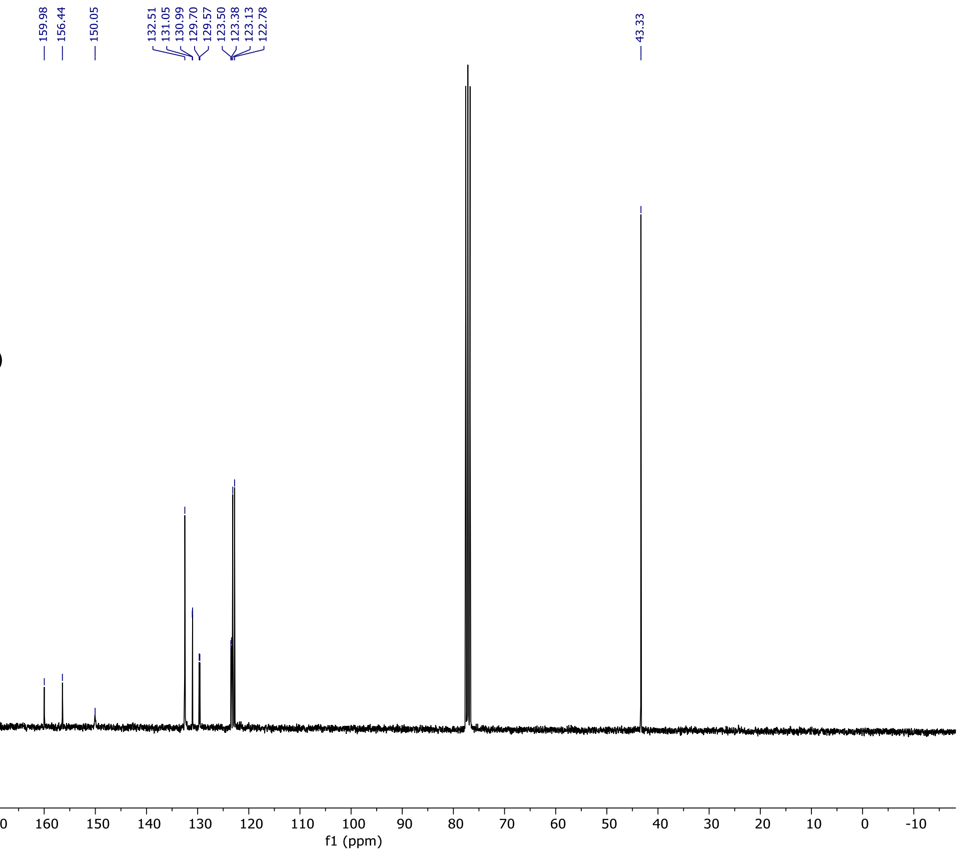

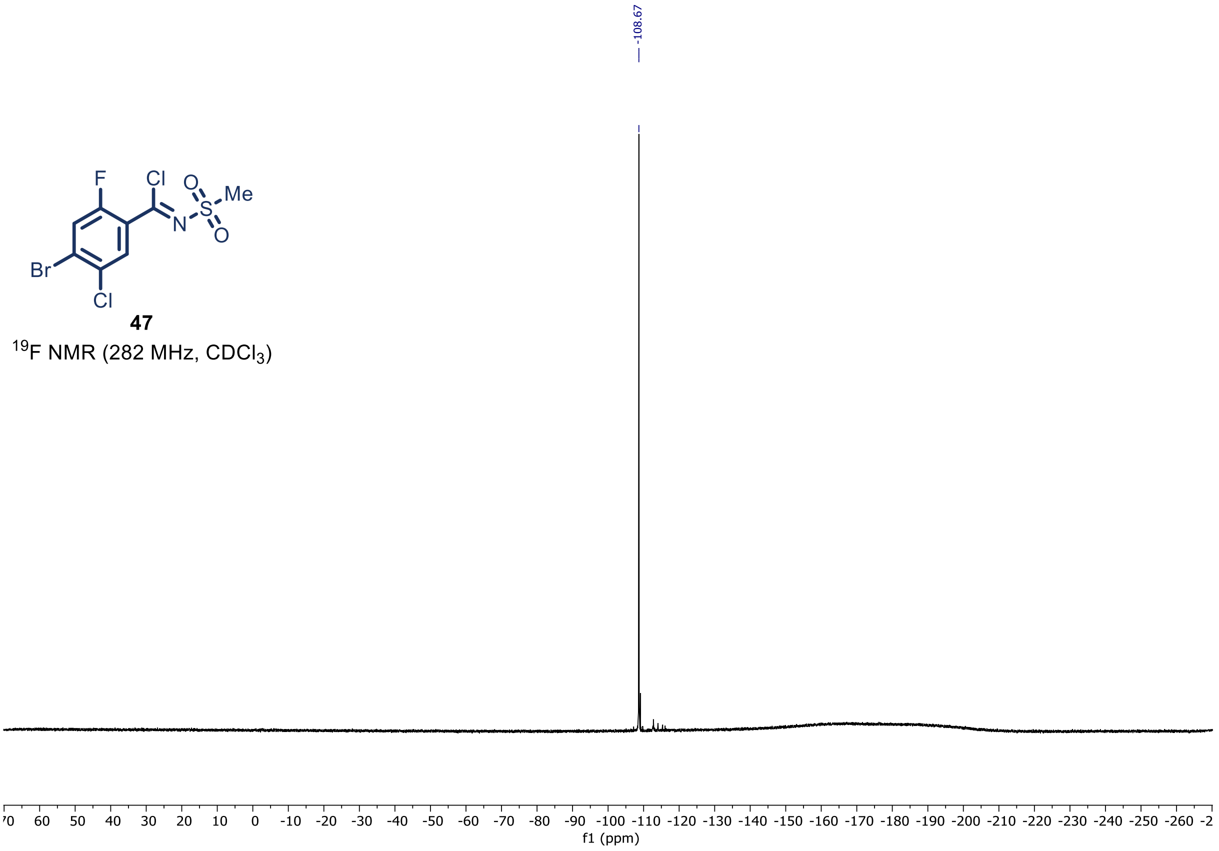

47

^{19}F NMR (282 MHz, $CDCl_3$)

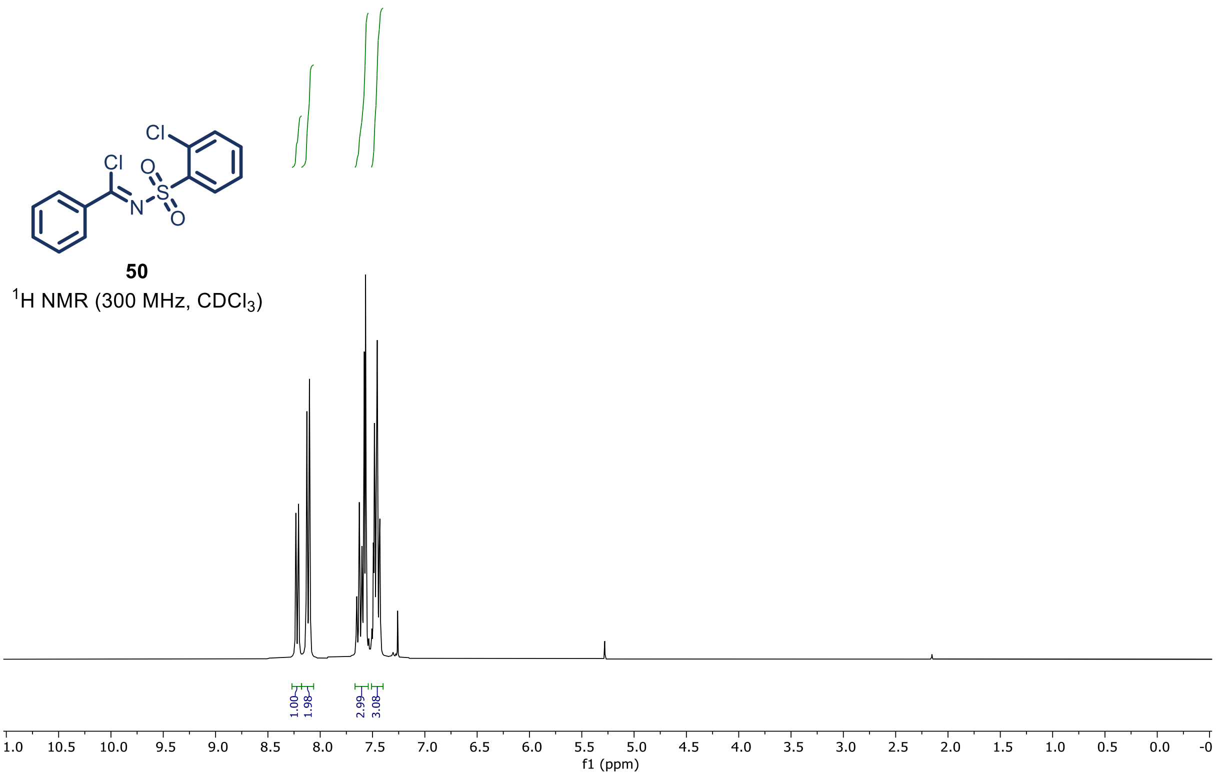
Cl
Cl
O
S
O
N
50
1H NMR (300 MHz, CDCl3)
1.00
1.98
2.99
3.08
f1 (ppm)

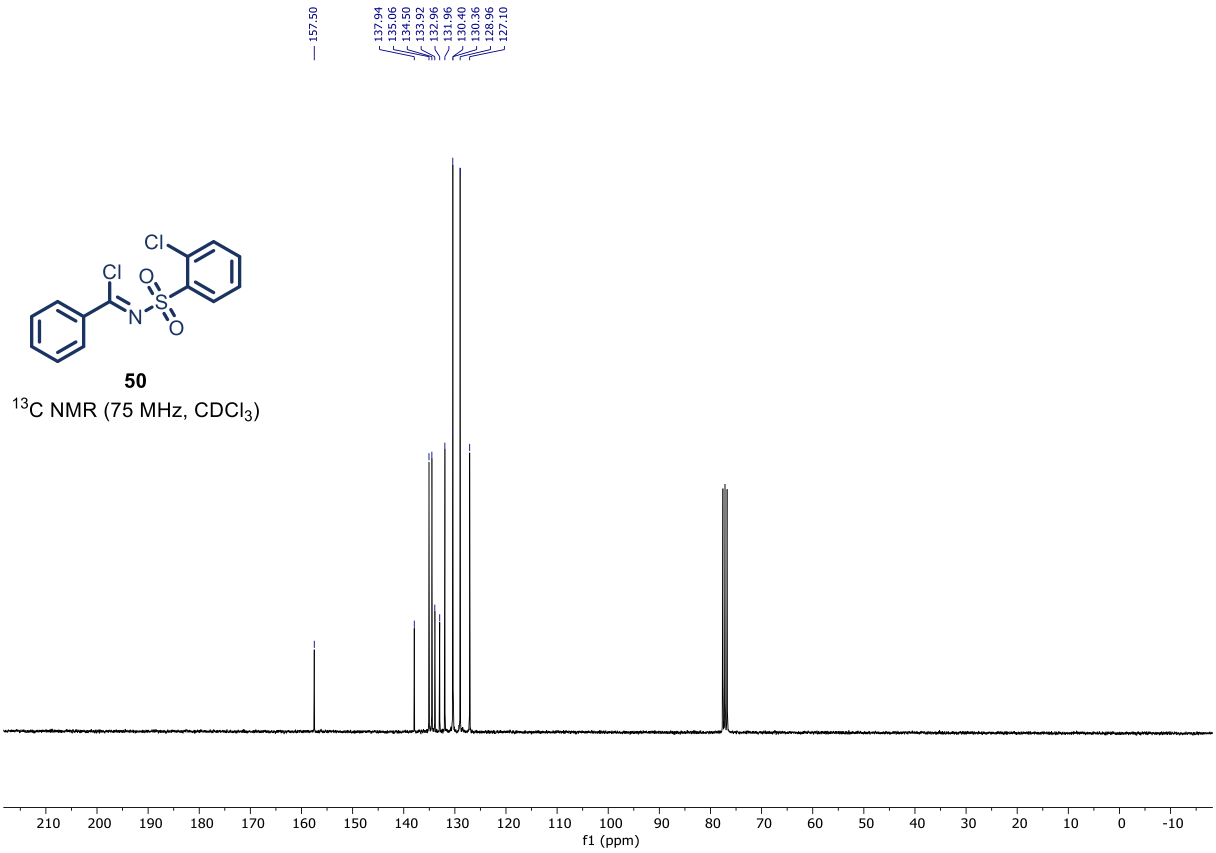
157.50
137.94
135.06
134.50
133.92
132.96
131.96
130.40
130.36
128.96
127.10
Cl
Cl
O
S
O
N
50
13C NMR (75 MHz, CDCl3)
f1 (ppm)

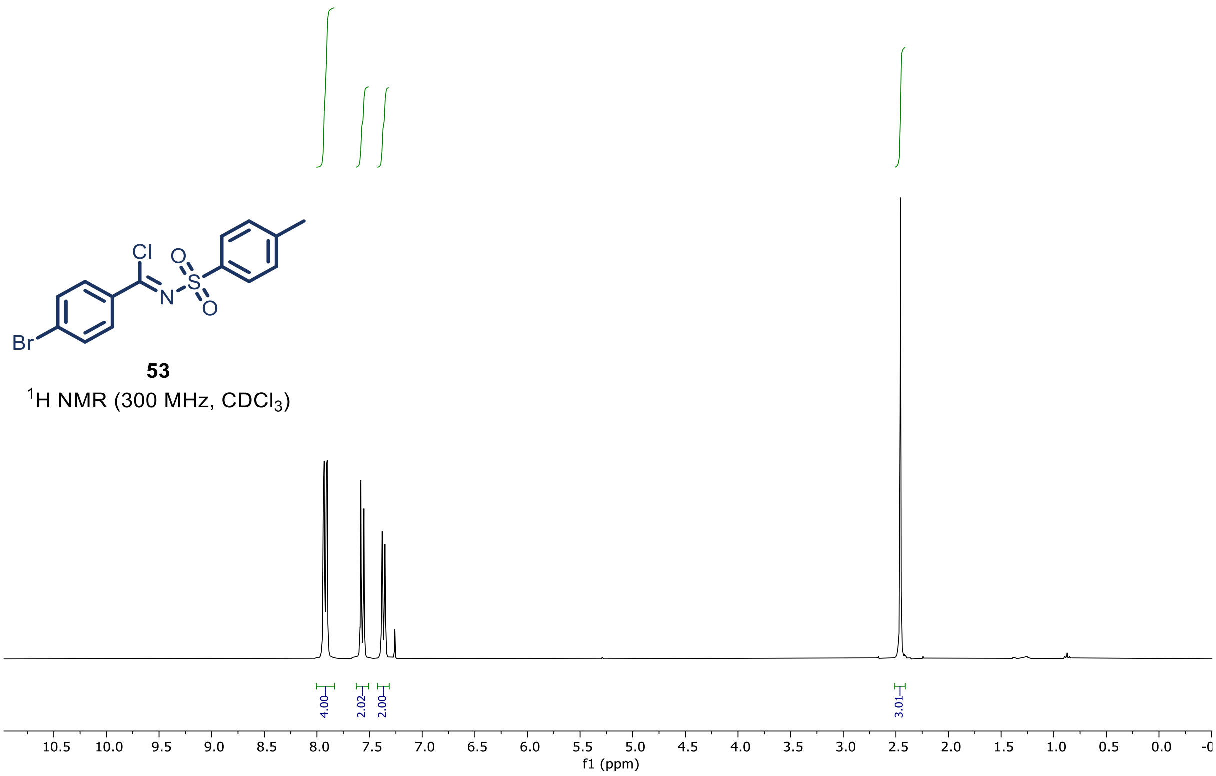

53

^{1}H NMR (300 MHz, $CDCl_3$)

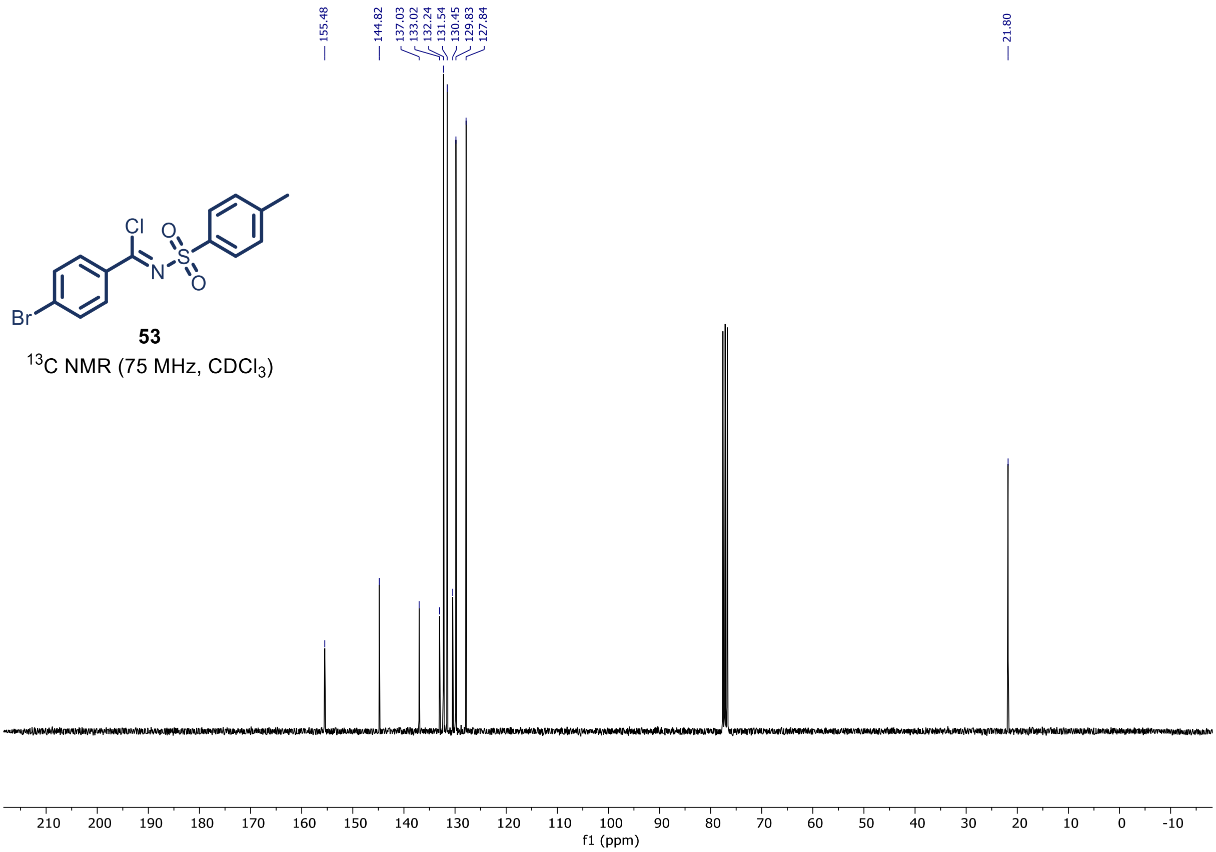

53

^{13}C NMR (75 MHz, $CDCl_3$)

5.2 Scope of Electrophiles

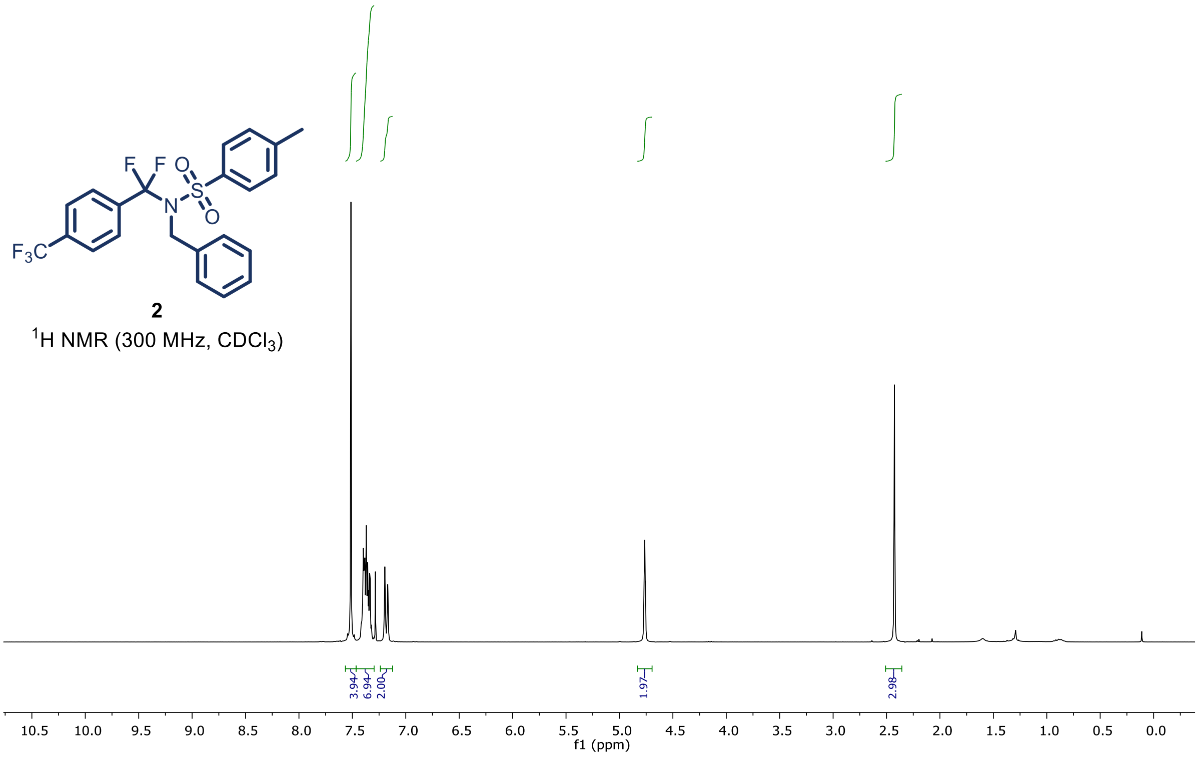

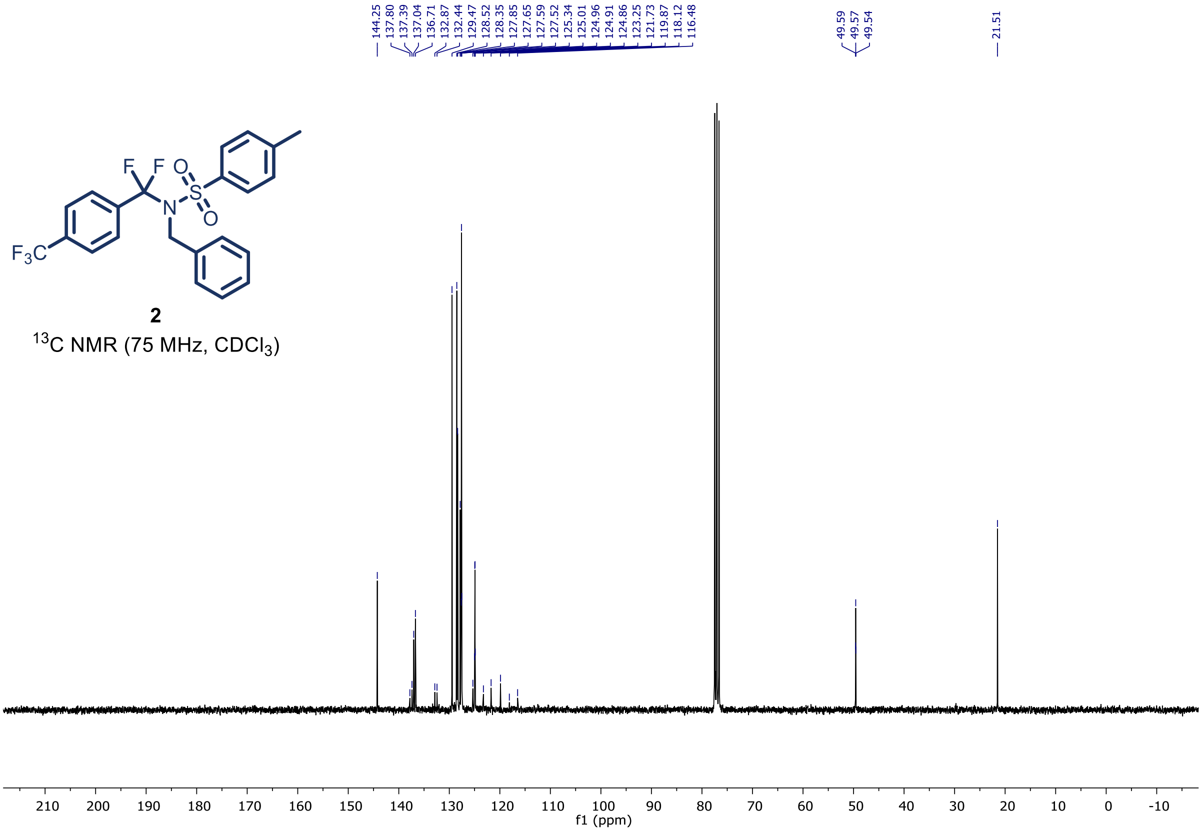

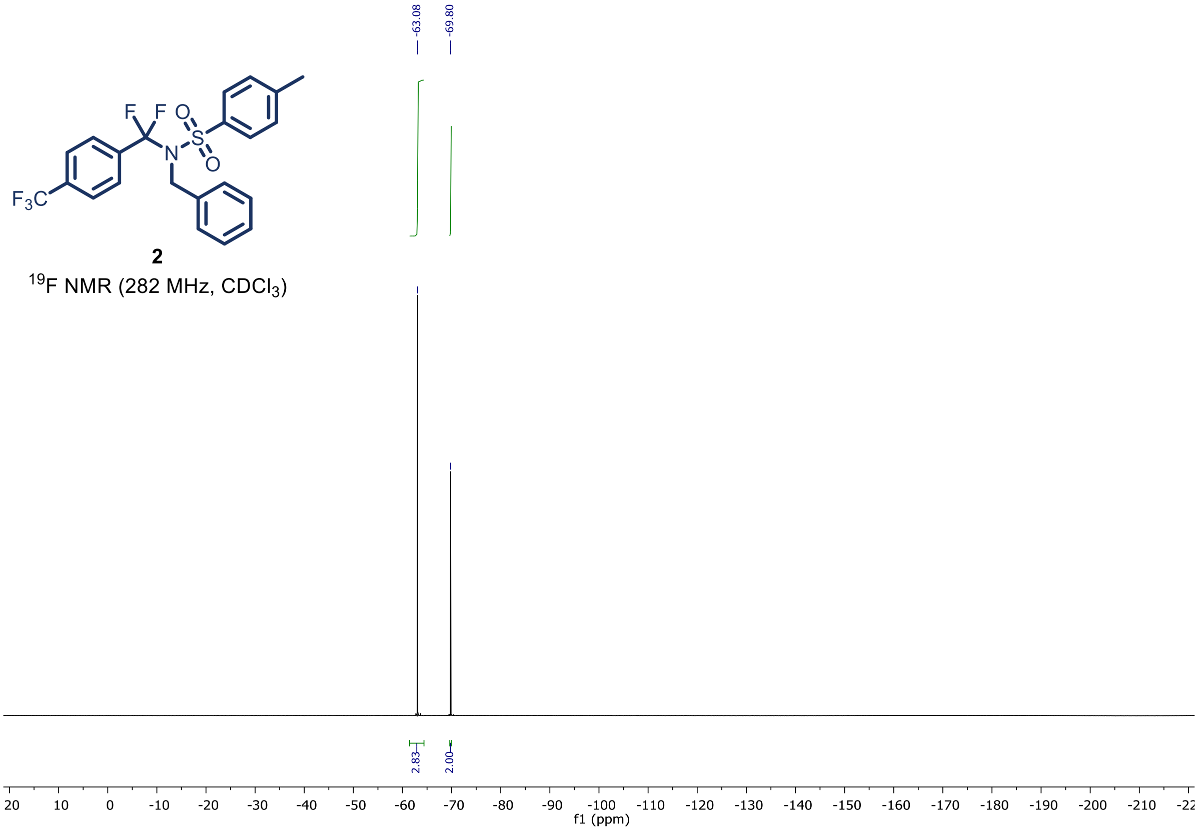

2

^{19}F NMR (282 MHz, $CDCl_3$)

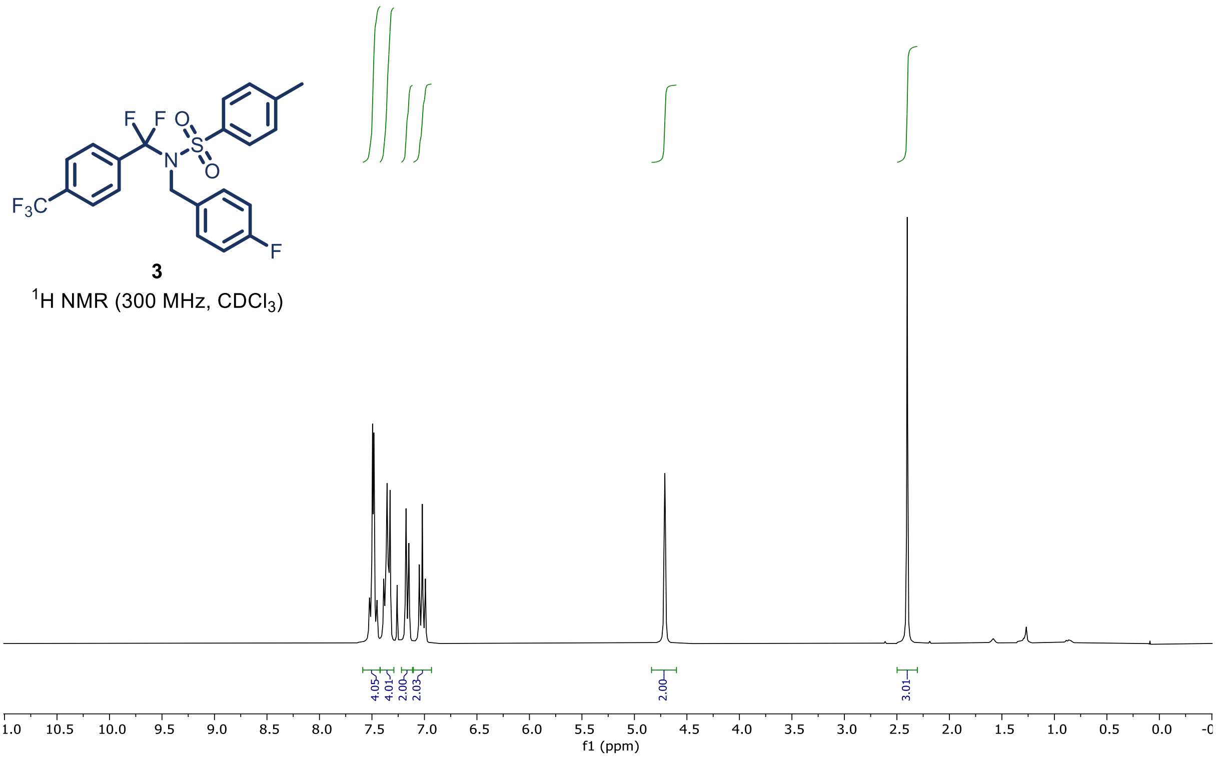

F F O
N S O
F3C
F
3
1H NMR (300 MHz, CDCl3)
4.05
4.01
2.00
2.03
2.00
3.01
1.0 10.5 10.0 9.5 9.0 8.5 8.0 7.5 7.0 6.5 6.0 5.5 5.0 4.5 4.0 3.5 3.0 2.5 2.0 1.5 1.0 0.5 0.0
f1 (ppm)

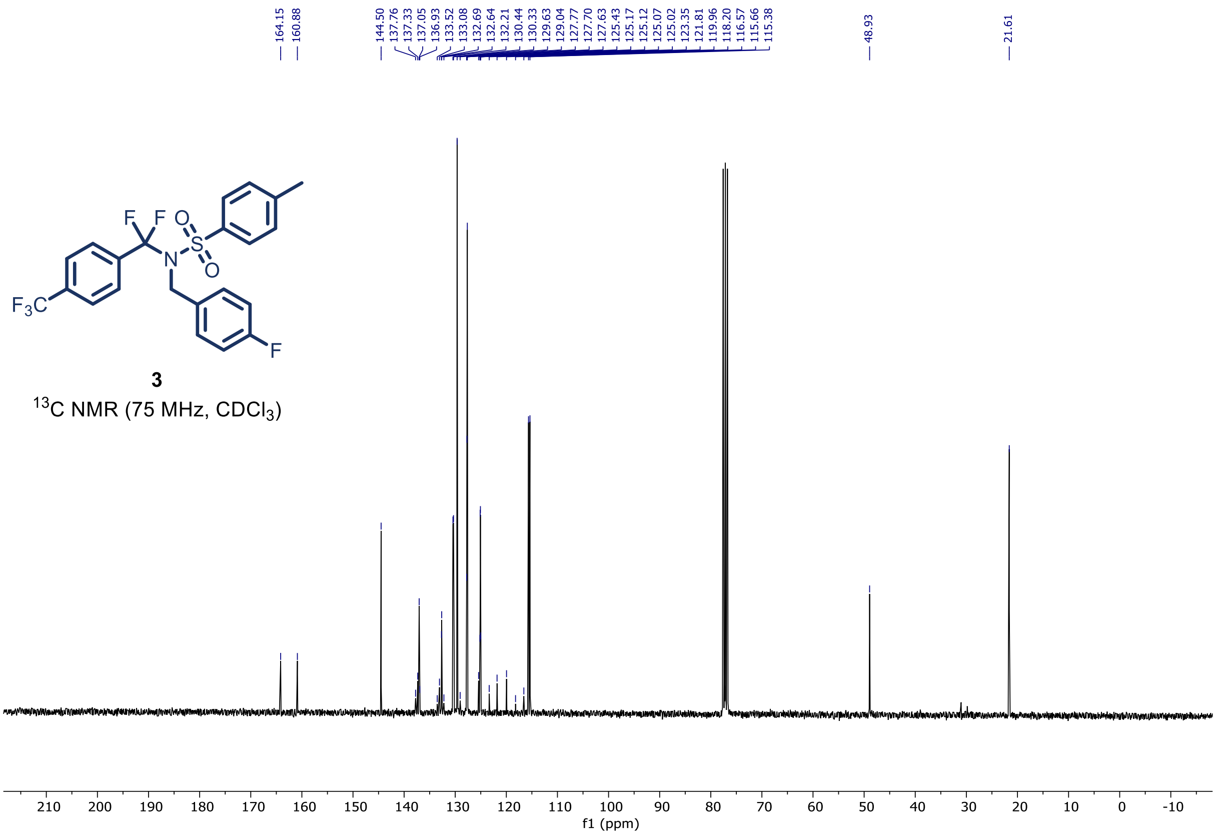

164.15
160.88
144.50
137.76
137.33
137.05
136.93
133.52
133.08
132.69
132.64
132.21
130.44
130.33
129.63
129.04
127.77
127.70
127.63
125.43
125.17
125.12
125.07
125.02
123.35
121.81
119.96
118.20
116.57
115.66
115.38
48.93
21.61
F F O
N S O
F3C
F
3
13C NMR (75 MHz, CDCl3)
210 200 190 180 170 160 150 140 130 120 110 100 90 80 70 60 50 40 30 20 10 0 -10
f1 (ppm)

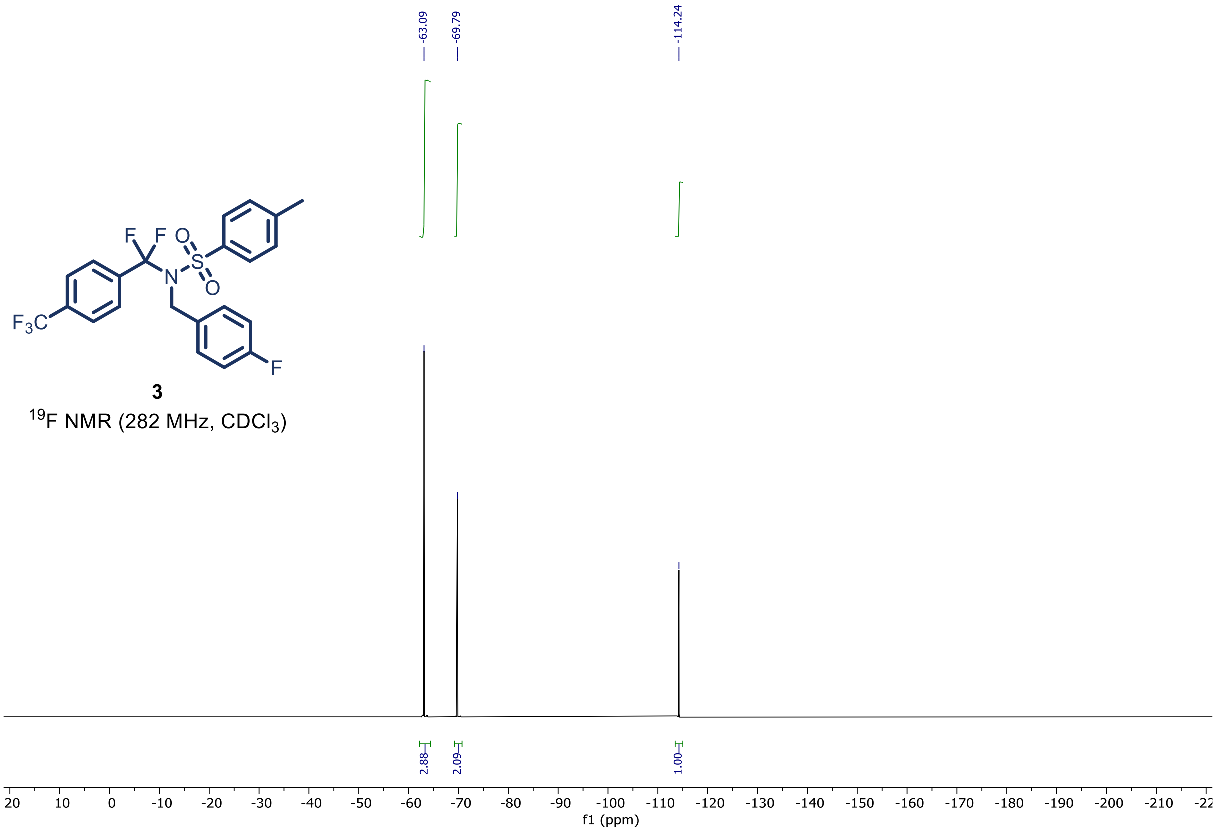

3
^{19}F NMR (282 MHz, $CDCl_3$)
-63.09
-69.79
-114.24
2.88
2.09
1.00
f1 (ppm)

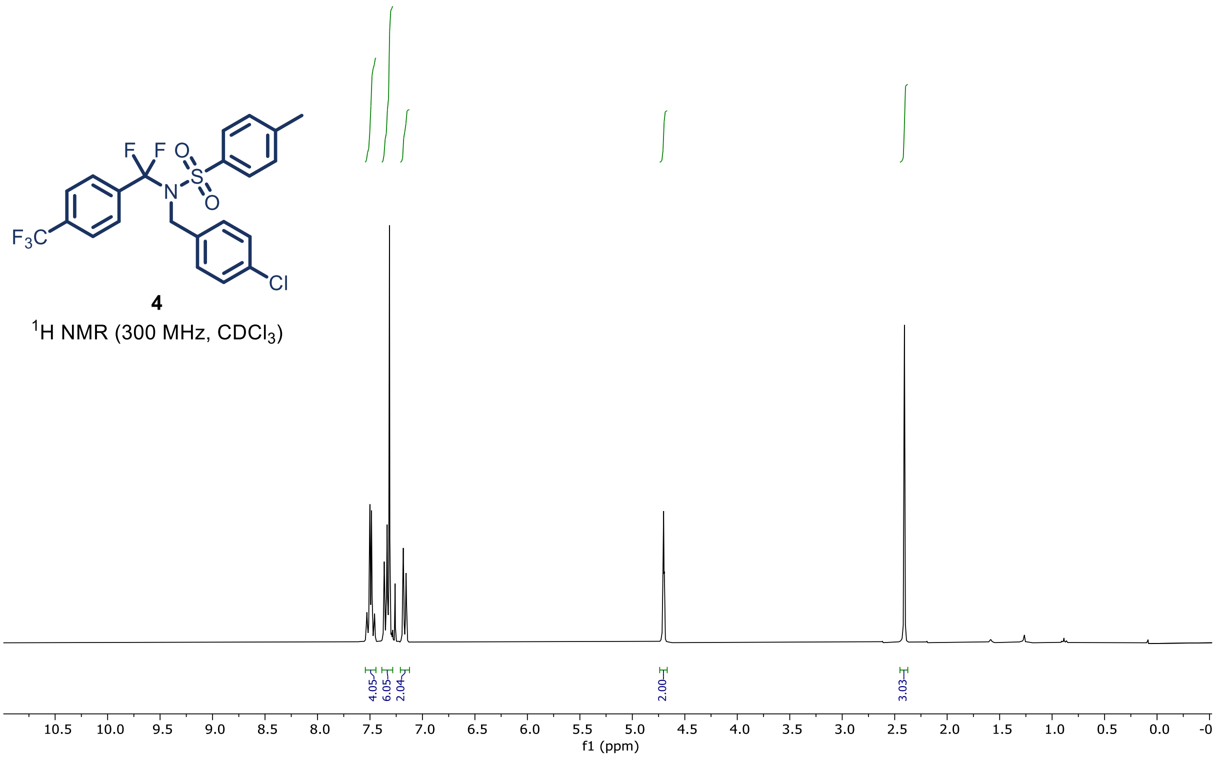

4

^{1}H NMR (300 MHz, $CDCl_3$)

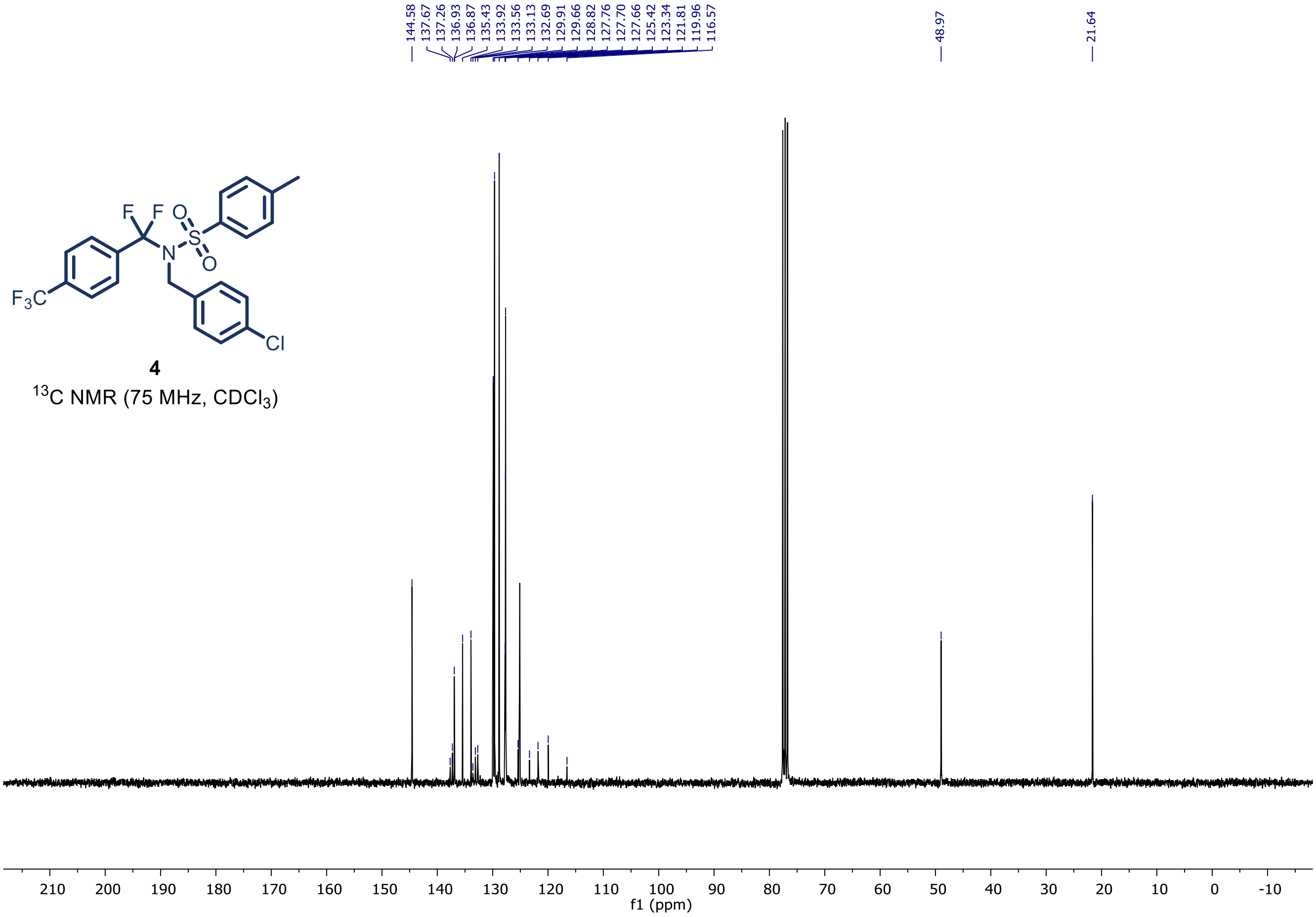

4

^{13}C NMR (75 MHz, $CDCl_3$)

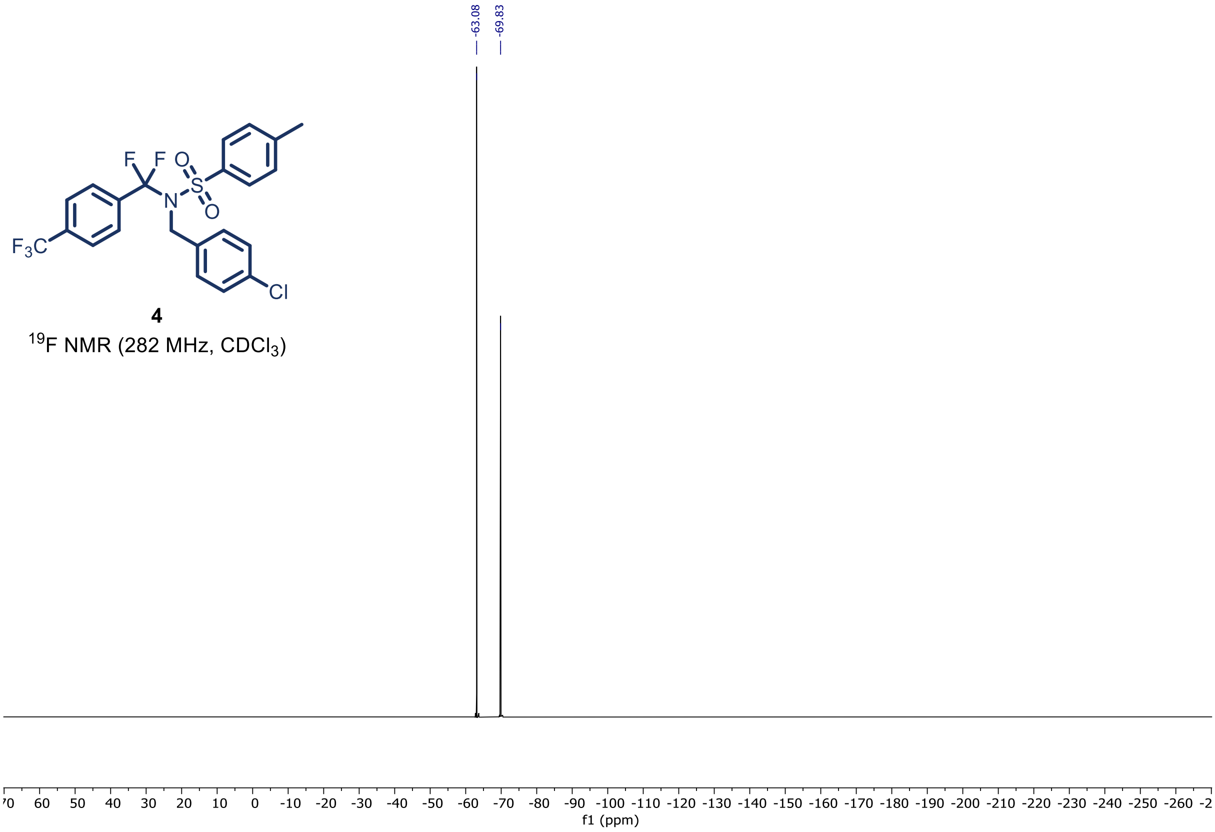

4

^{19}F NMR (282 MHz, $CDCl_3$)

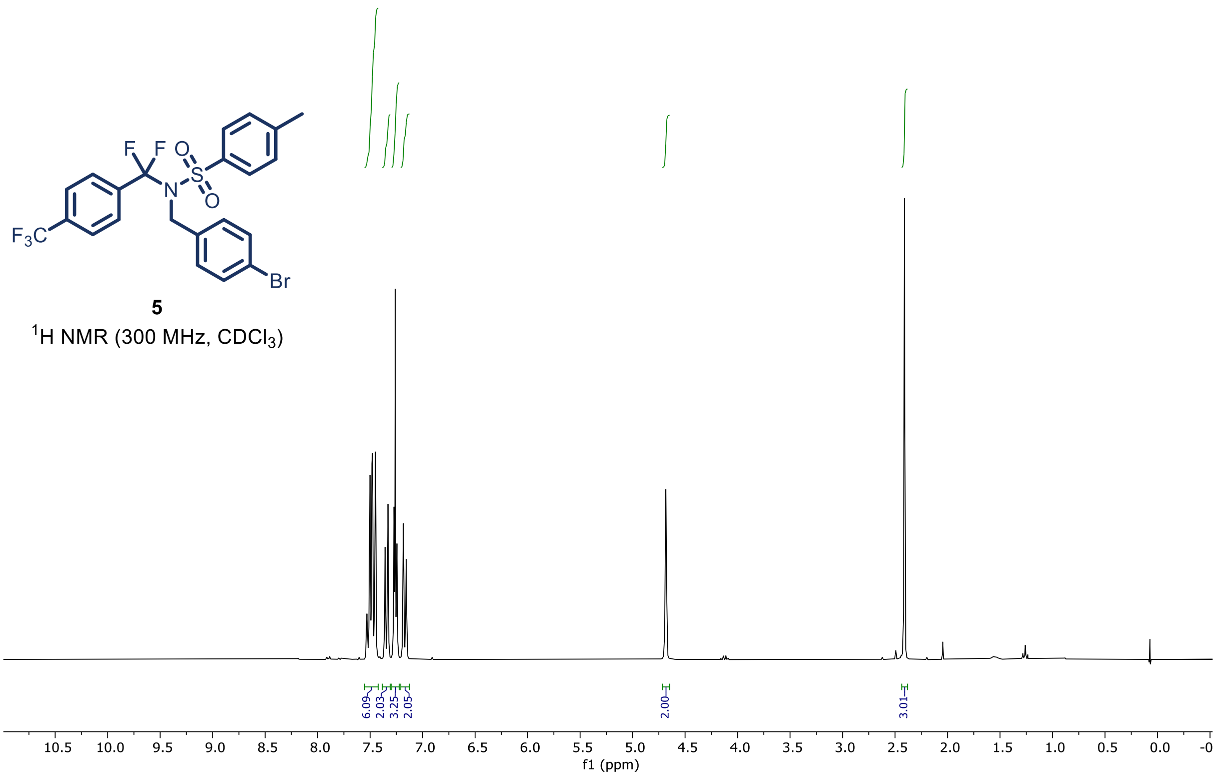
F F
O
N
S
O
F3C
Br
5
1H NMR (300 MHz, CDCl3)
6.09
2.03
3.25
2.05
2.00
3.01
f1 (ppm)

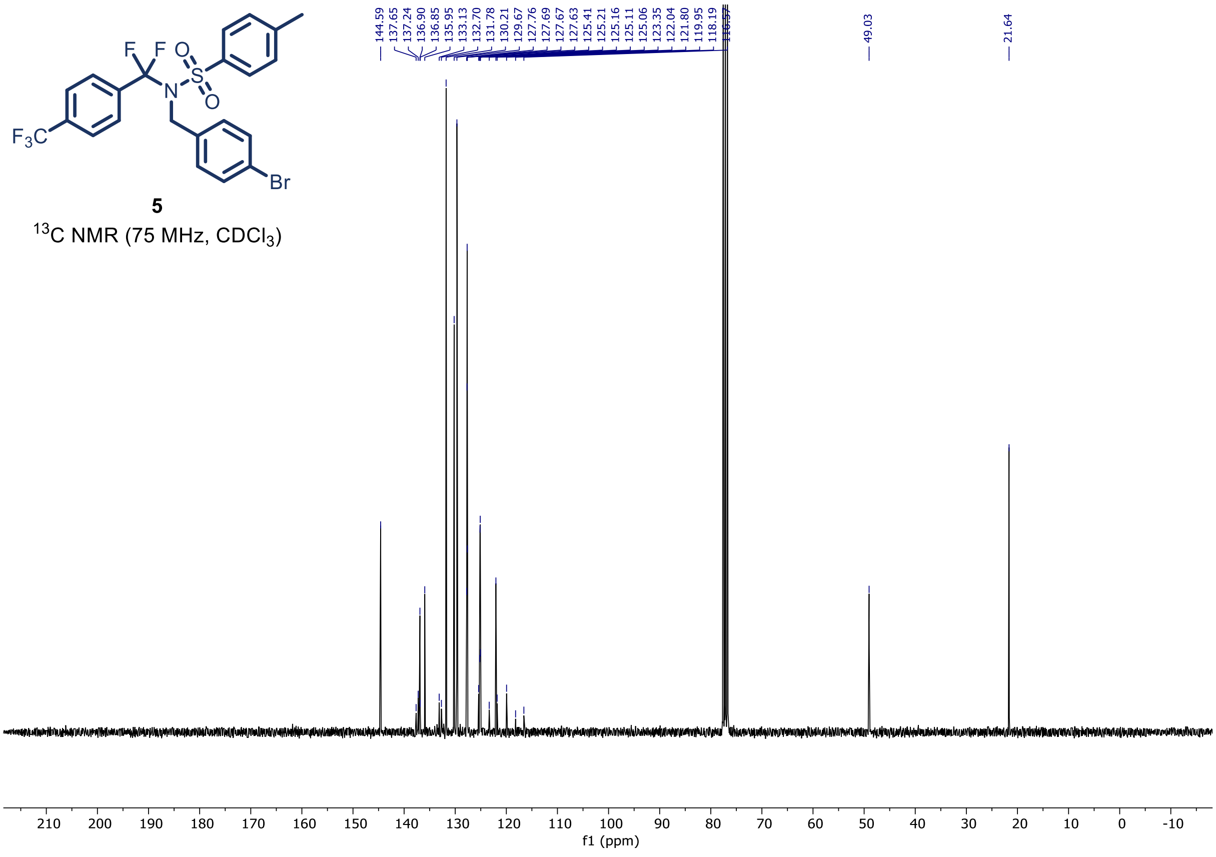
144.59
137.65
137.24
136.90
136.85
135.95
133.13
132.70
131.78
130.21
129.67
127.76
127.69
127.67
127.63
125.41
125.21
125.16
125.11
125.06
123.35
122.04
121.80
119.95
118.19
49.03
21.64
F F
O
N
S
O
F3C
Br
5
13C NMR (75 MHz, CDCl3)
f1 (ppm)

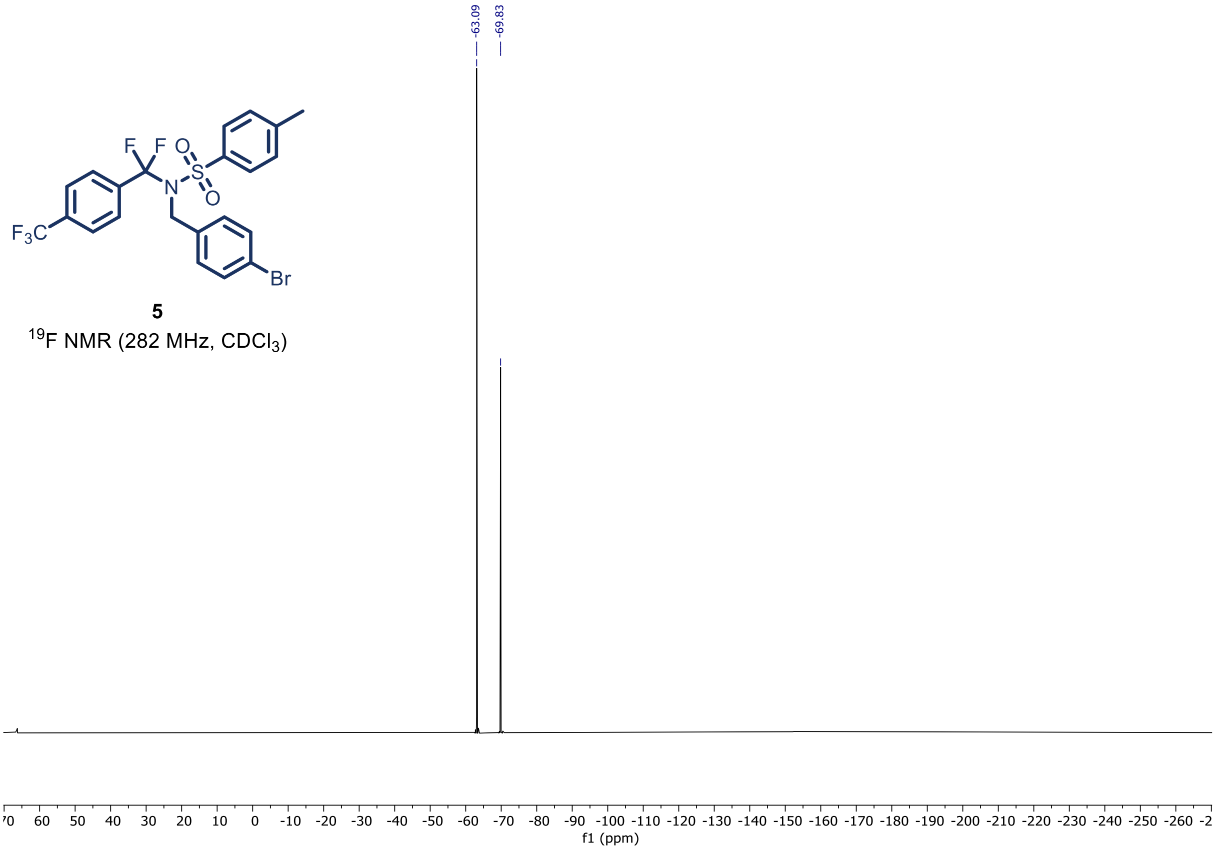
F F
O
S
N
O
F3C
Br
5
19F NMR (282 MHz, CDCl3)
-63.09
-69.83
70 60 50 40 30 20 10 0 -10 -20 -30 -40 -50 -60 -70 -80 -90 -100 -110 -120 -130 -140 -150 -160 -170 -180 -190 -200 -210 -220 -230 -240 -250 -260 -2
f1 (ppm)

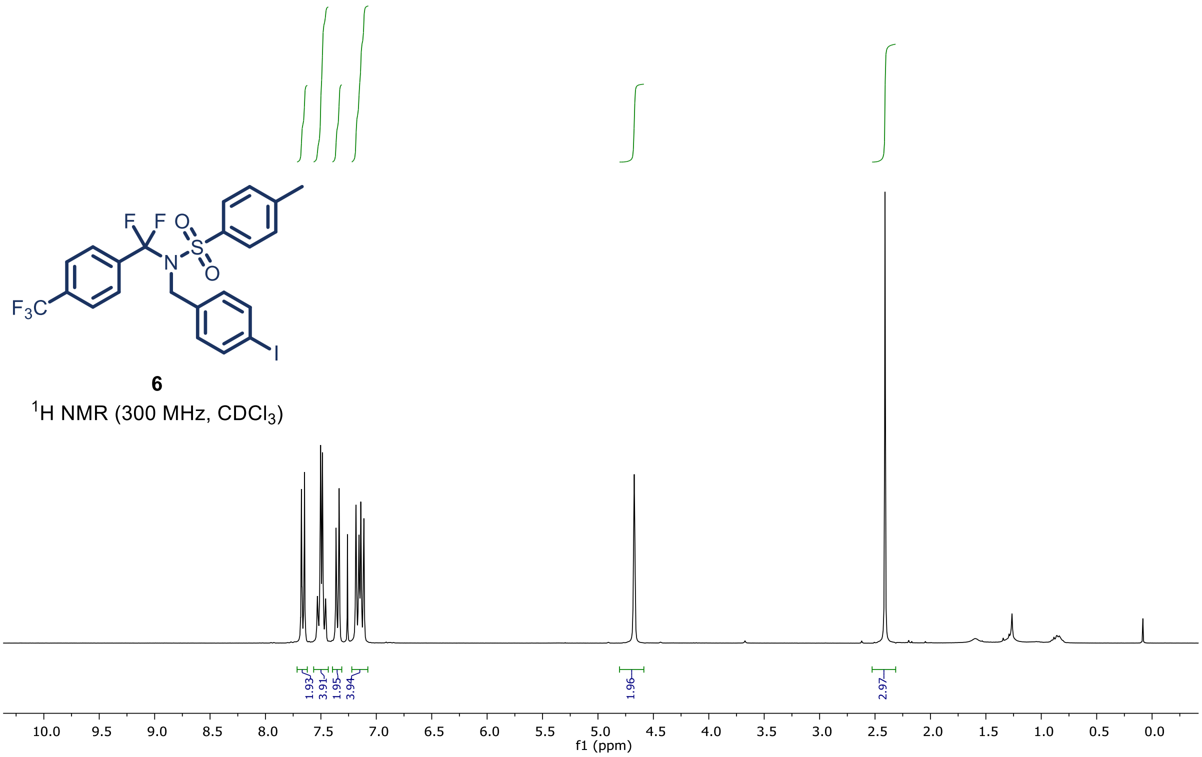
F F O
S
N
O
F3C
I
6
1H NMR (300 MHz, CDCl3)
1.93
3.91
1.95
3.94
1.96
2.97
10.0 9.5 9.0 8.5 8.0 7.5 7.0 6.5 6.0 5.5 5.0 4.5 4.0 3.5 3.0 2.5 2.0 1.5 1.0 0.5 0.0
f1 (ppm)

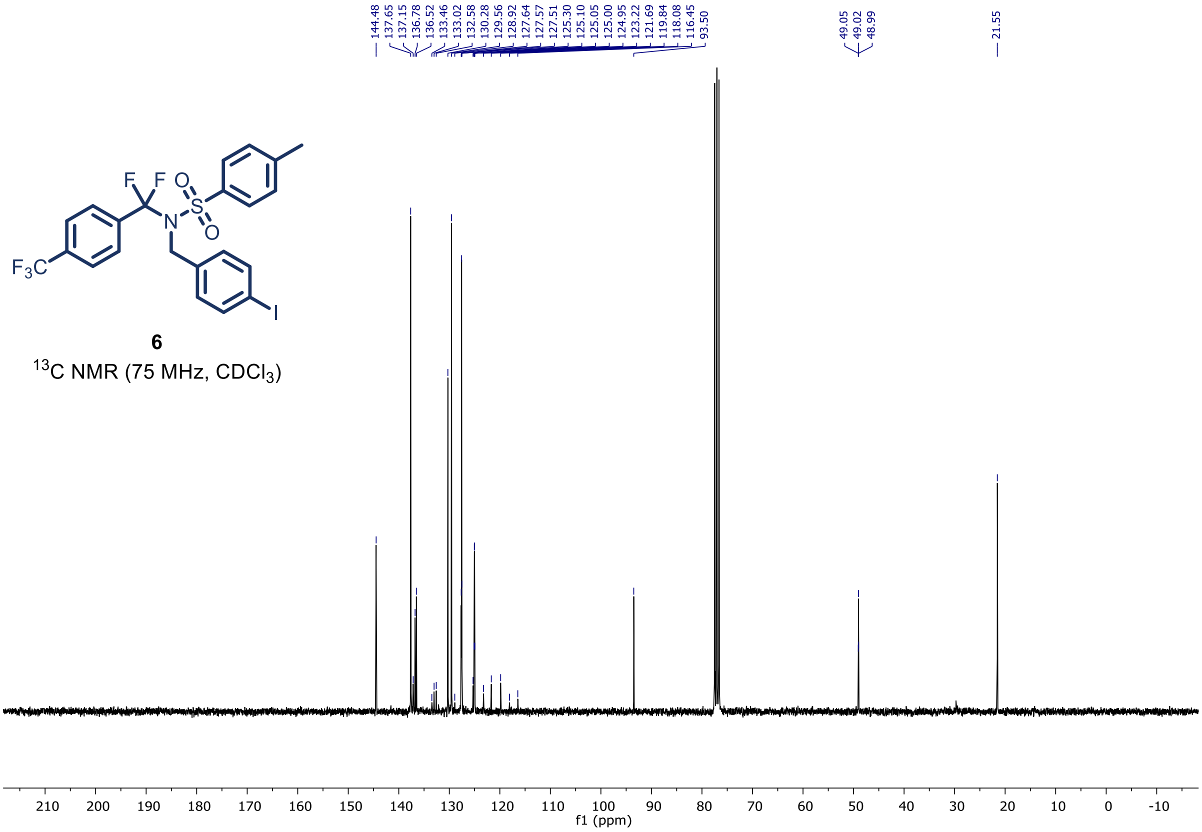
144.48
137.65
137.15
136.78
136.52
133.46
133.02
132.58
130.28
129.56
128.92
127.64
127.57
127.51
125.30
125.10
125.05
125.00
124.95
123.22
121.69
119.84
118.08
116.45
93.50
49.05
49.02
48.99
21.55
F F O
S
N
O
F3C
I
6
13C NMR (75 MHz, CDCl3)
210 200 190 180 170 160 150 140 130 120 110 100 90 80 70 60 50 40 30 20 10 0 -10
f1 (ppm)

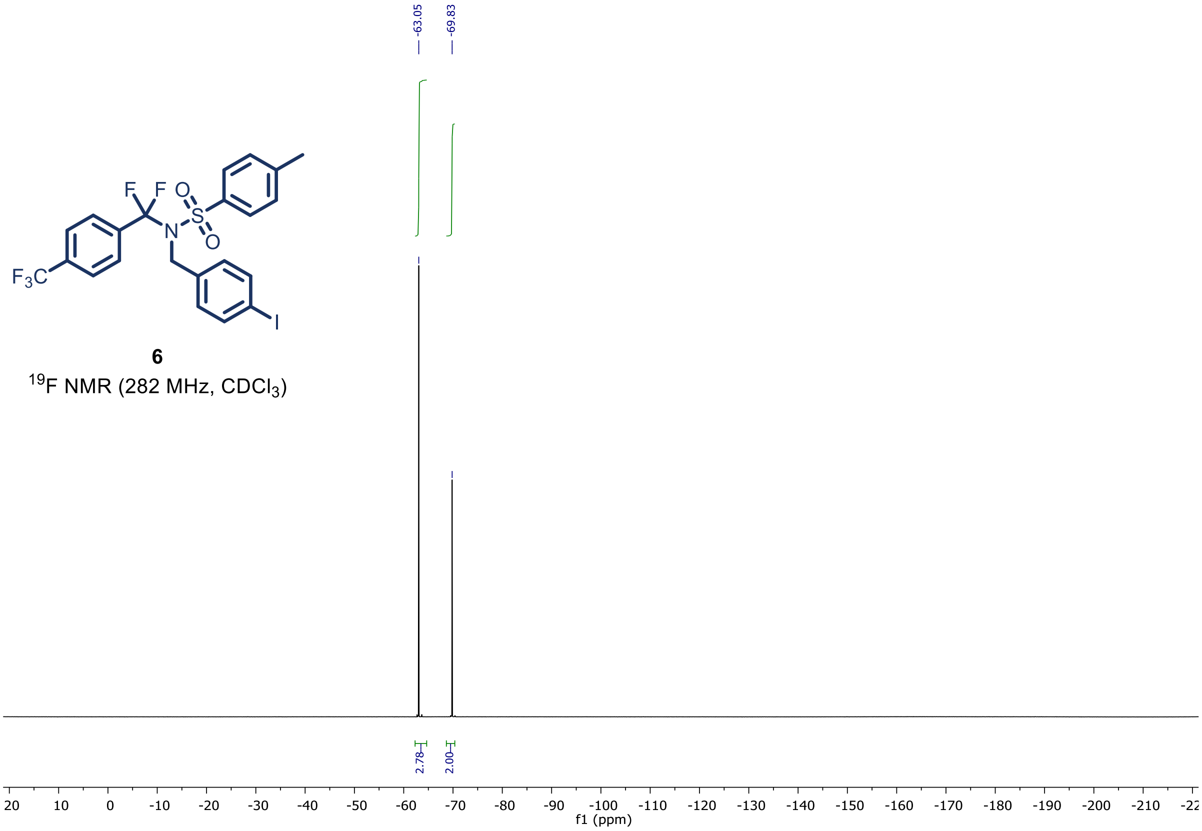

6

^{19}F NMR (282 MHz, $CDCl_3$)

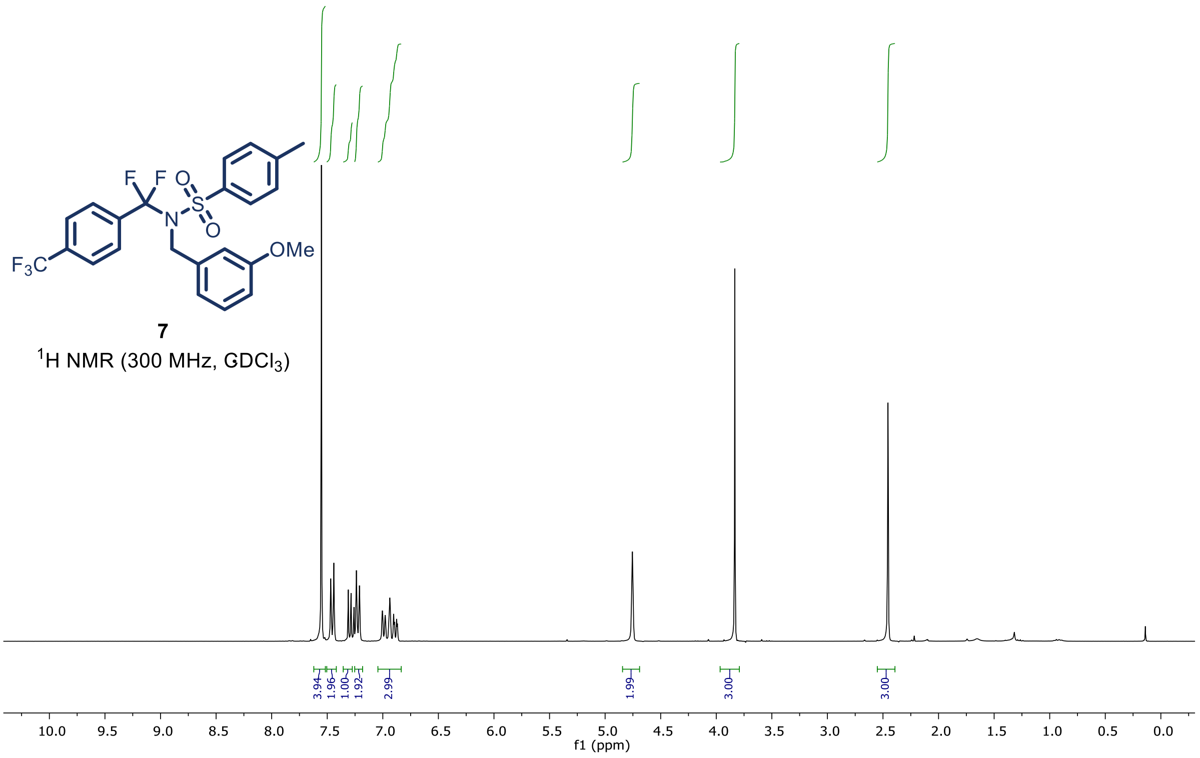

F F O
N S O
F3C OMe
7
1H NMR (300 MHz, GDCl3)
3.94
1.96
1.00
1.92
2.99
1.99
3.00
3.00
f1 (ppm)

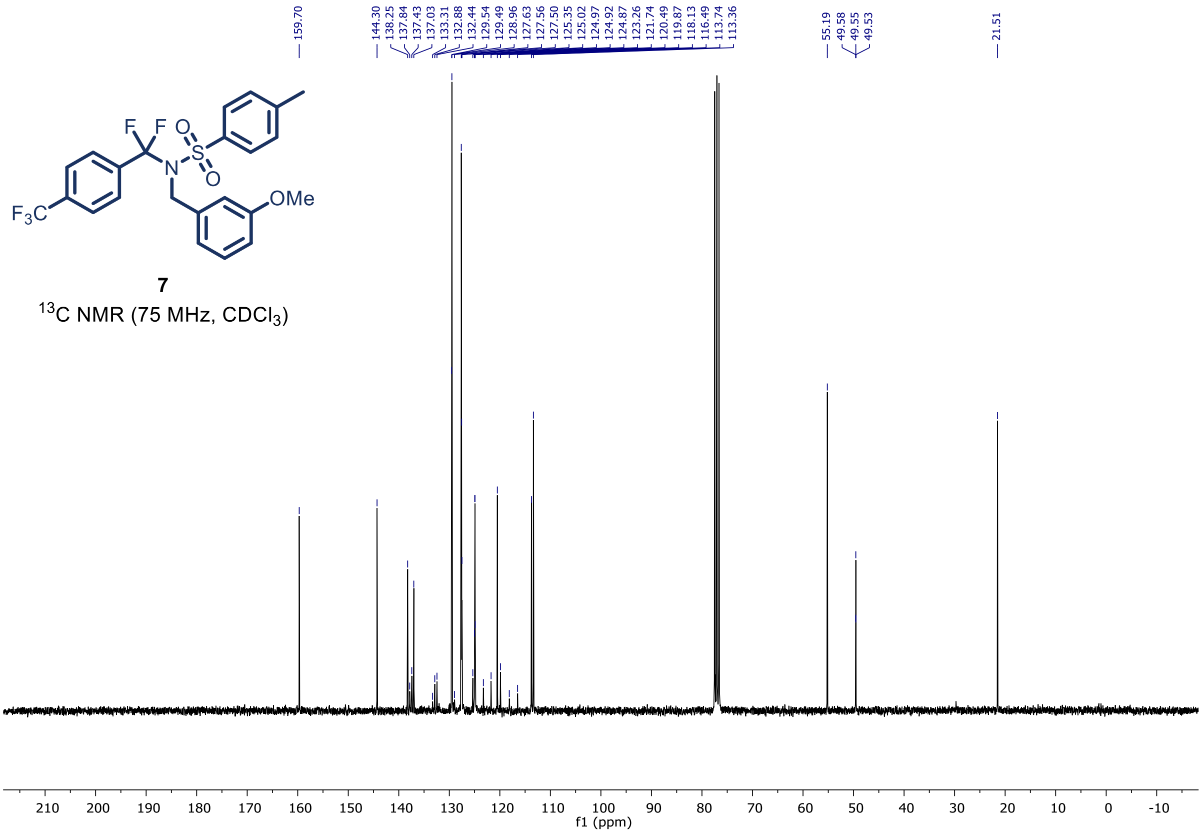

159.70
144.30
138.25
137.84
137.43
137.03
133.31
132.88
132.44
129.54
129.49
128.96
127.63
127.56
127.50
125.35
125.02
124.97
124.92
124.87
123.26
121.74
120.49
119.87
118.13
116.49
113.74
113.36
55.19
49.58
49.55
49.53
21.51
F F O
N S O
F3C OMe
7
13C NMR (75 MHz, CDCl3)
f1 (ppm)

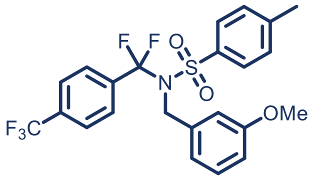

7

^{19}F NMR (282 MHz, $CDCl_3$)

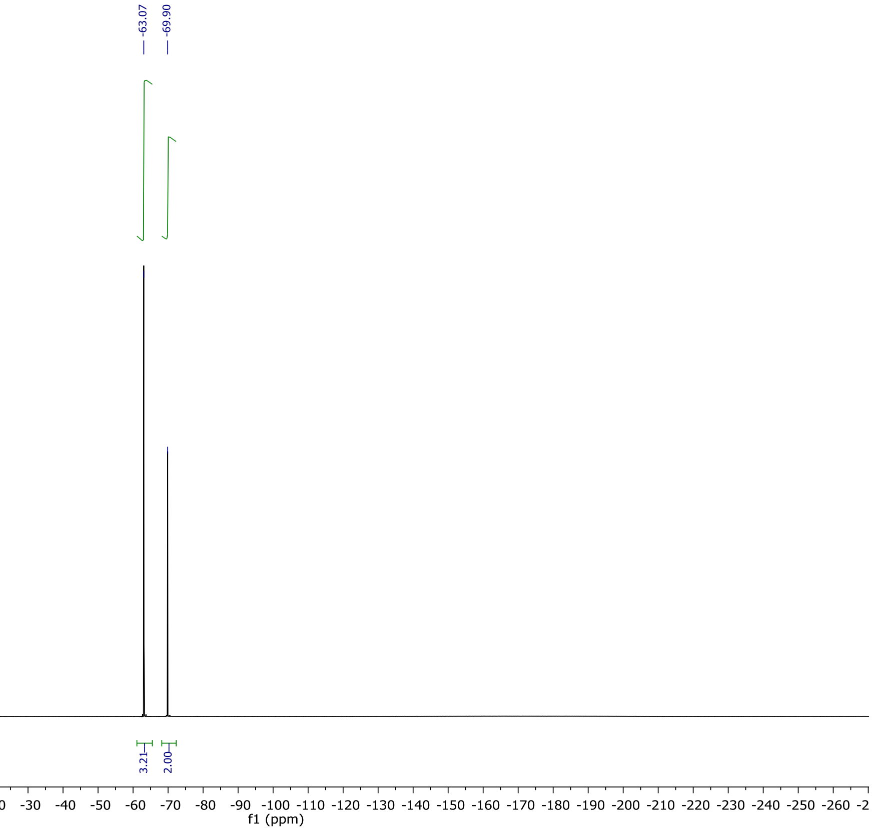

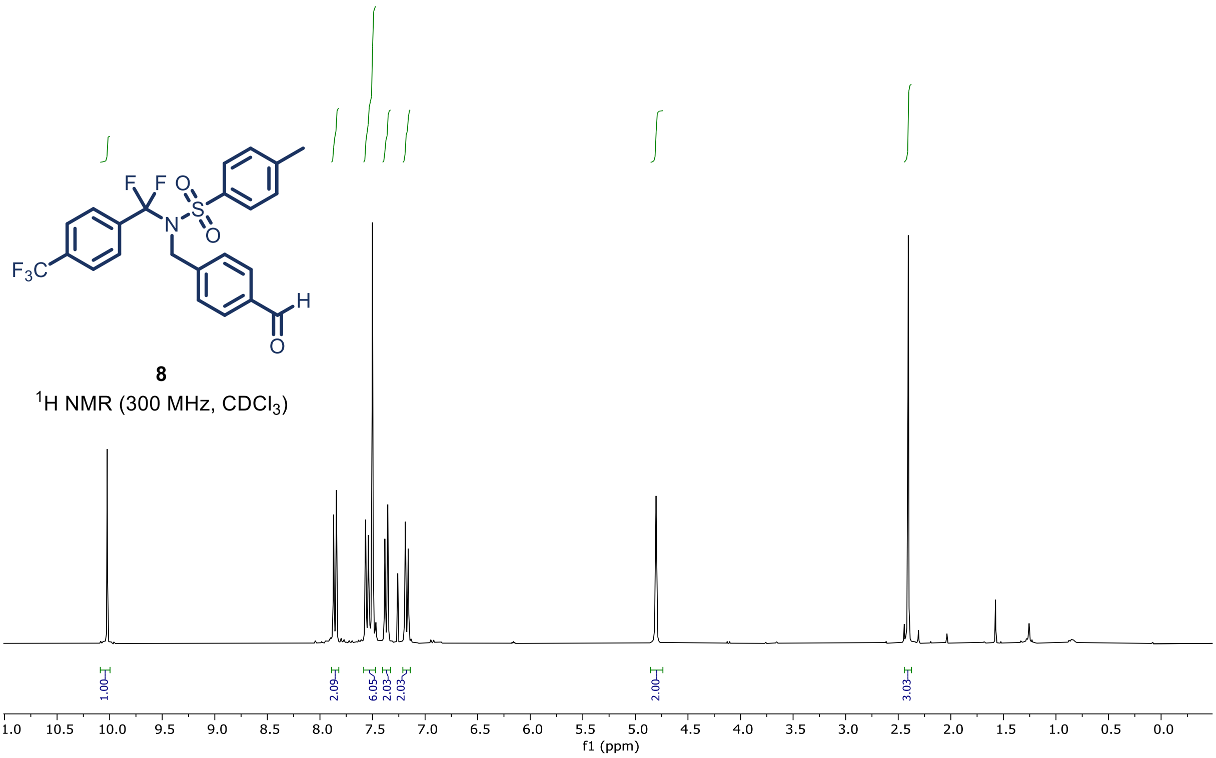
8
^{1}H NMR (300 MHz, CDCl$_3$)
1.00
2.09
6.05
2.03
2.03
2.00
3.03
f1 (ppm)

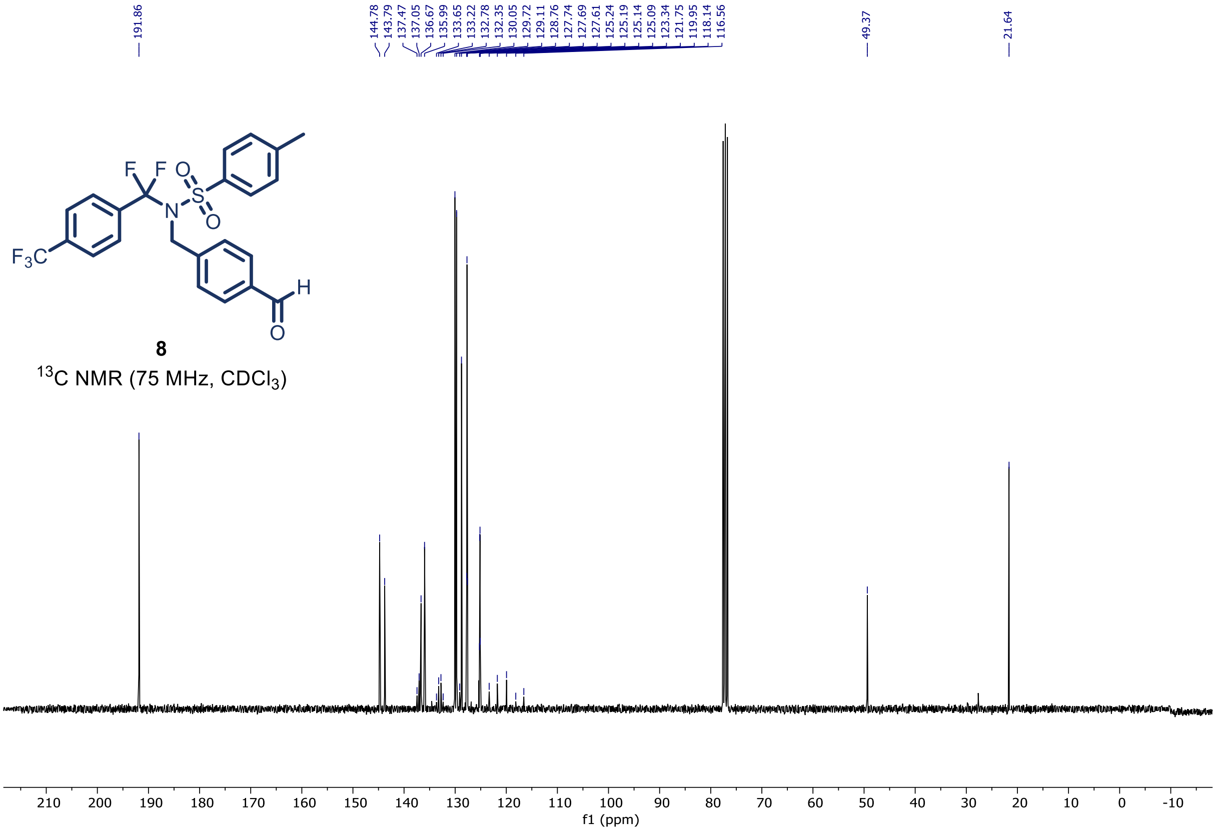
191.86
144.78
143.79
137.47
137.05
136.67
135.99
133.65
133.22
132.78
132.35
130.05
129.72
129.11
128.76
127.74
127.69
127.61
125.24
125.19
125.14
125.09
123.34
121.75
119.95
118.14
116.56
49.37
21.64
8
^{13}C NMR (75 MHz, CDCl$_3$)
f1 (ppm)

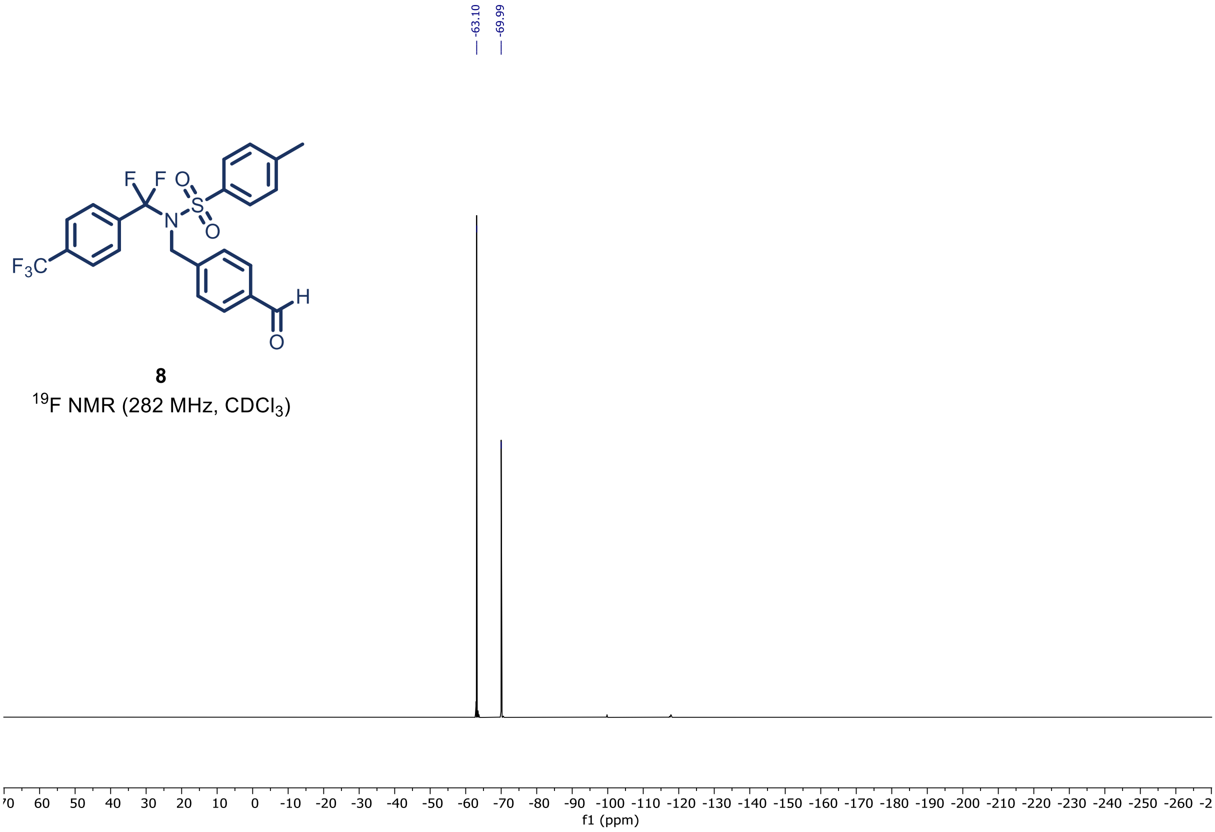

8

^{19}F NMR (282 MHz, $CDCl_3$)

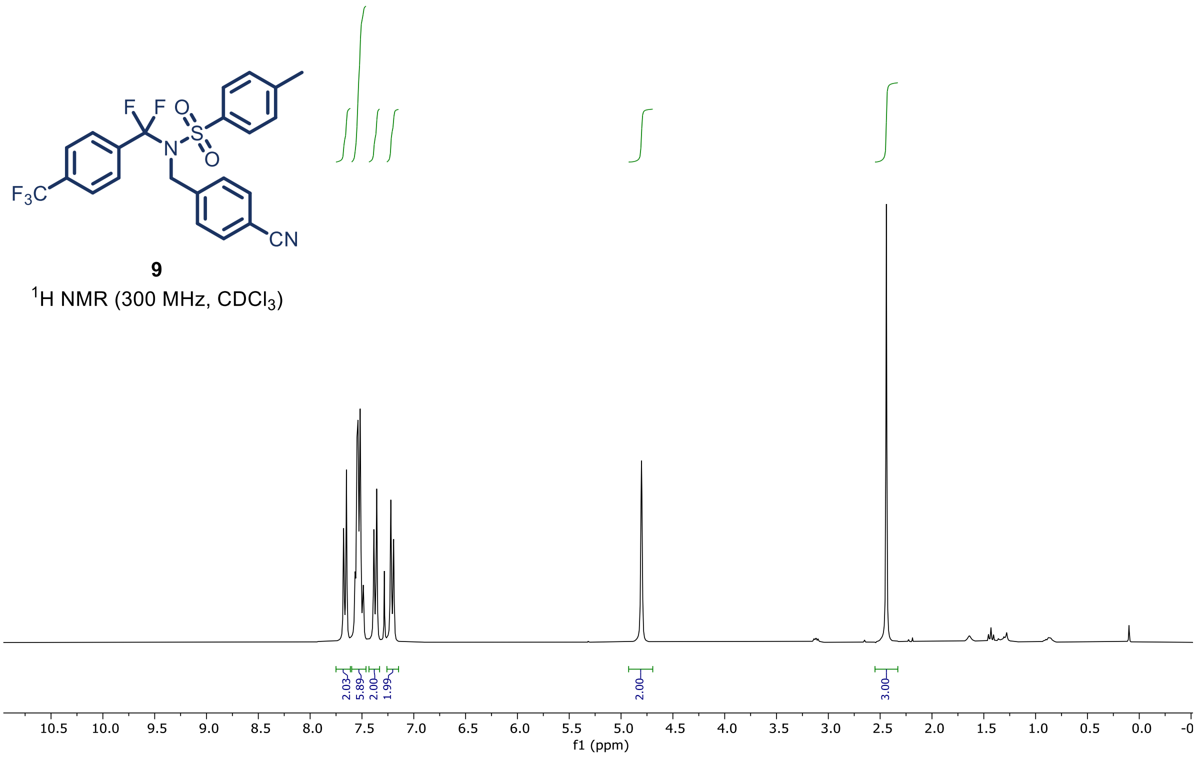

9

^{1}H NMR (300 MHz, $CDCl_3$)

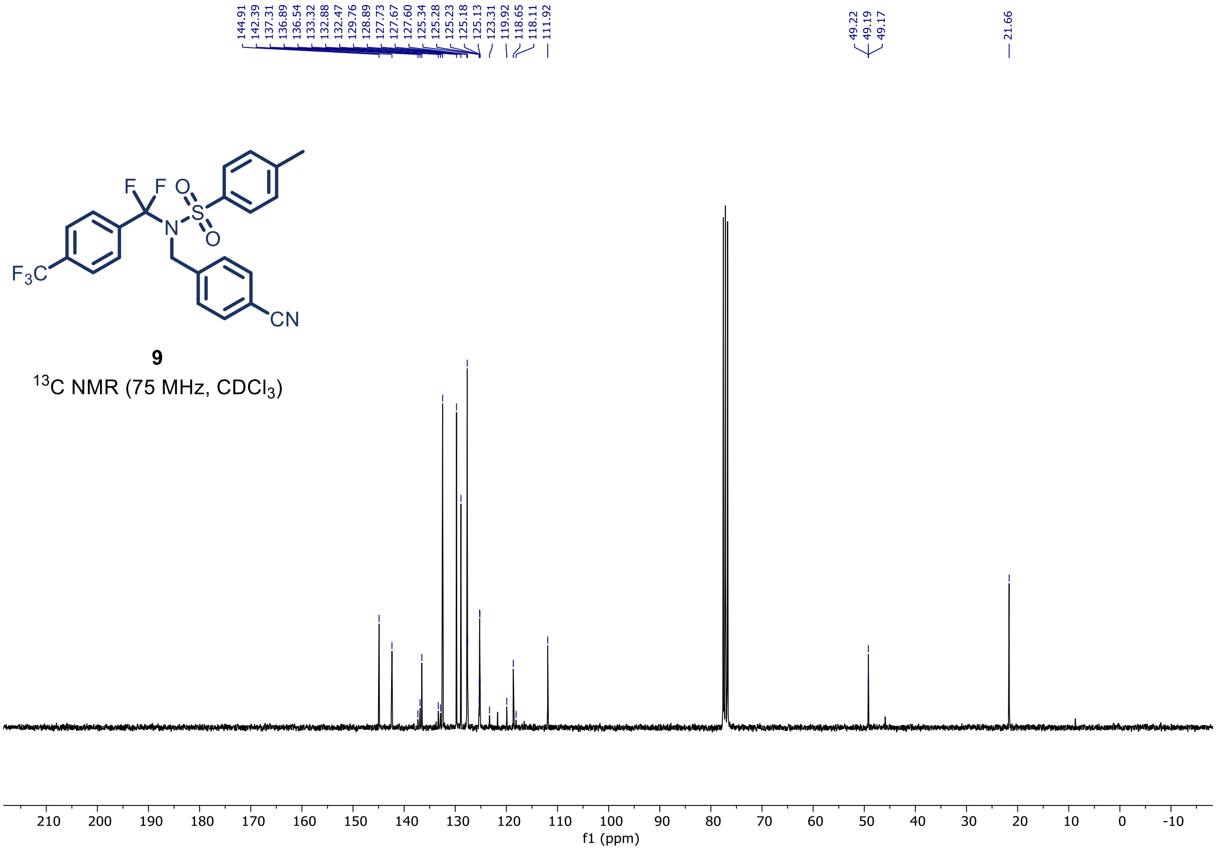

9

^{13}C NMR (75 MHz, $CDCl_3$)

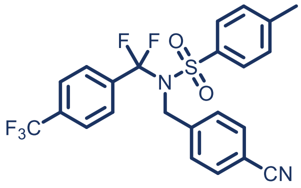

9

^{19}F NMR (282 MHz, $CDCl_3$)

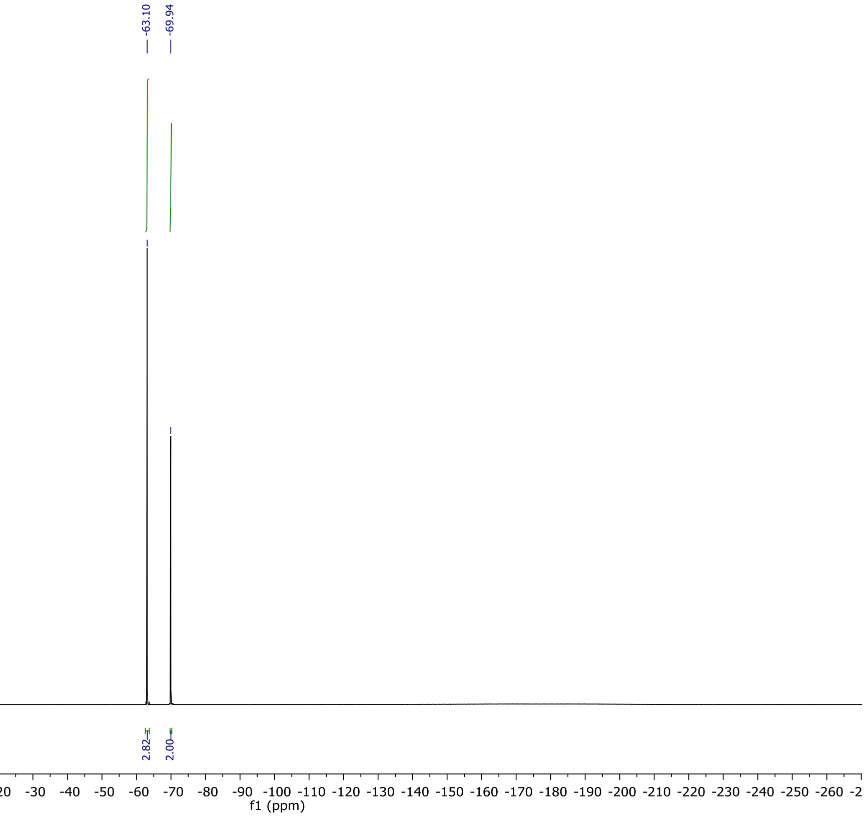

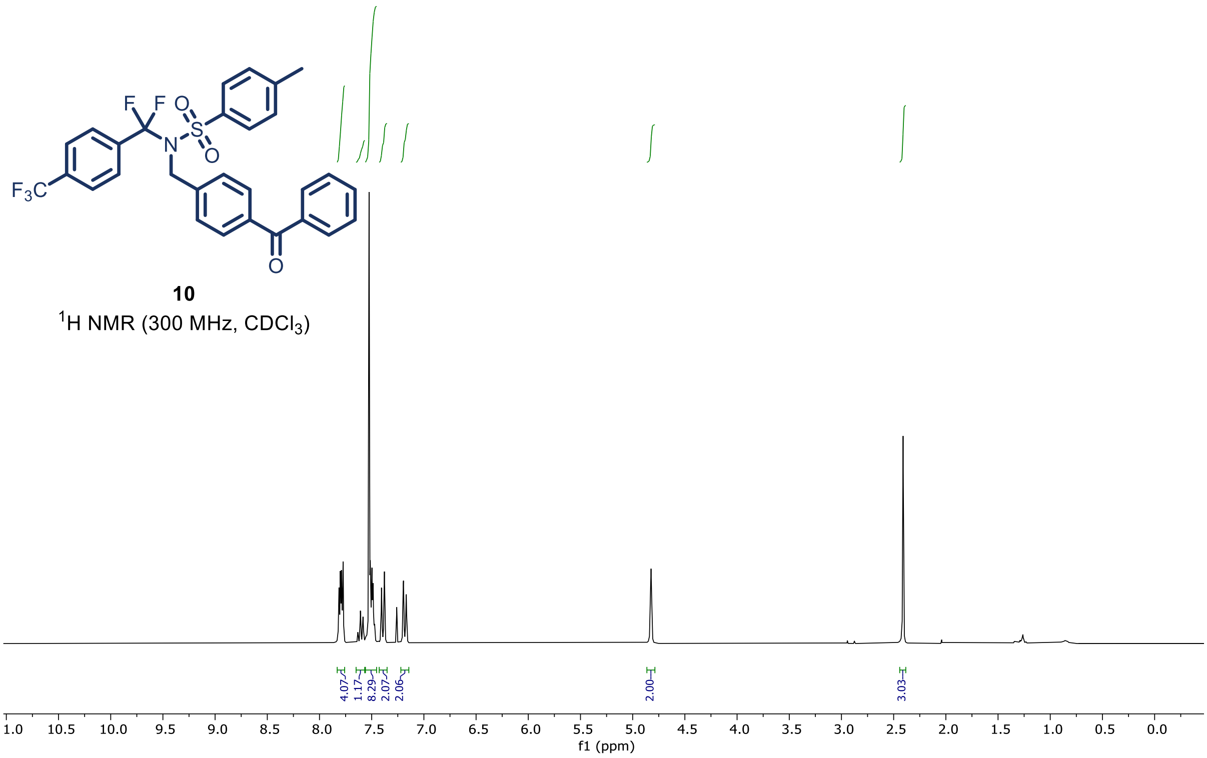
F F O
N S
O
F3C
O
10
1H NMR (300 MHz, CDCl3)
4.07
1.17
8.29
2.07
2.06
2.00
3.03
1.0 10.5 10.0 9.5 9.0 8.5 8.0 7.5 7.0 6.5 6.0 5.5 5.0 4.5 4.0 3.5 3.0 2.5 2.0 1.5 1.0 0.5 0.0
f1 (ppm)

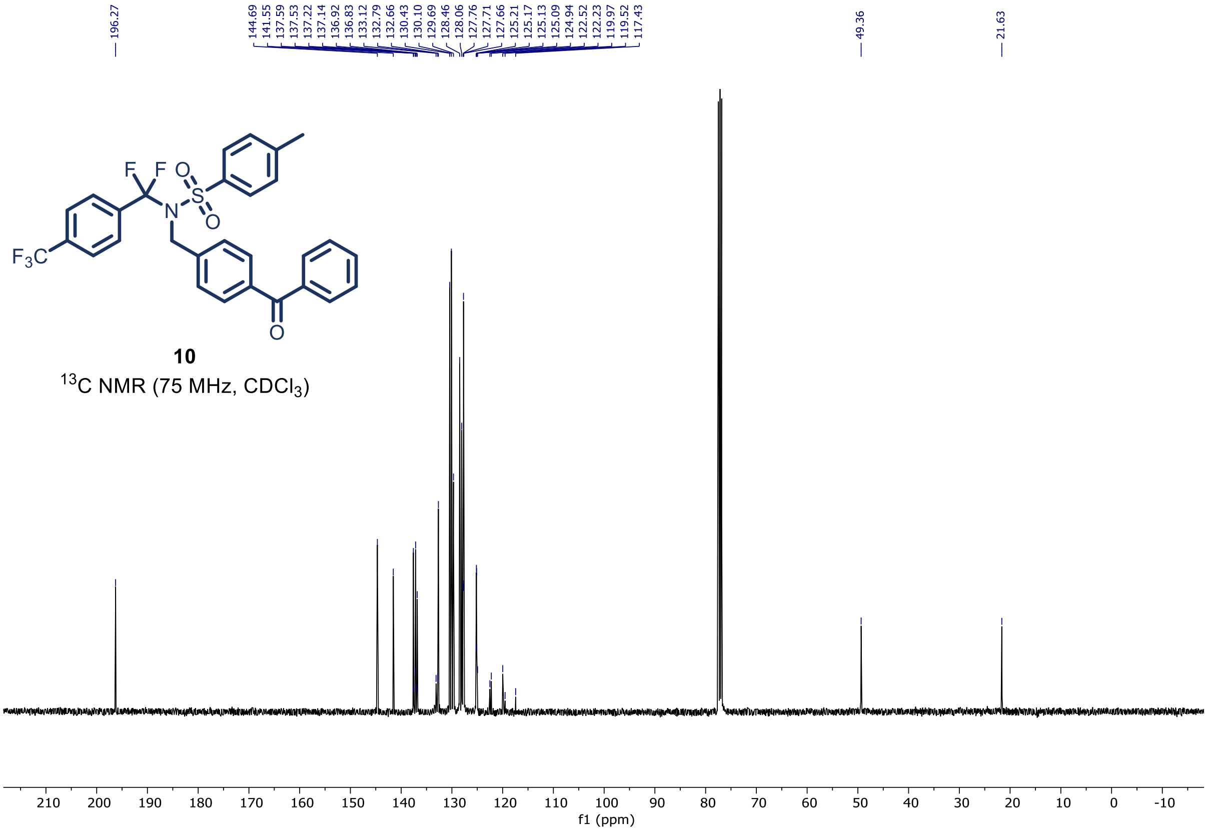
196.27
144.69
141.55
137.59
137.53
137.22
137.14
136.92
136.83
133.12
132.79
132.66
130.43
130.10
129.69
128.46
128.06
127.76
127.71
127.66
125.21
125.17
125.13
125.09
124.94
122.52
122.23
119.97
119.52
117.43
49.36
21.63
F F O
N S
O
F3C
O
10
13C NMR (75 MHz, CDCl3)
210 200 190 180 170 160 150 140 130 120 110 100 90 80 70 60 50 40 30 20 10 0 -10
f1 (ppm)

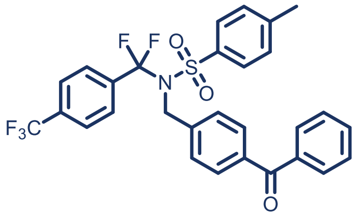

10

^{19}F NMR (282 MHz, $CDCl_3$)

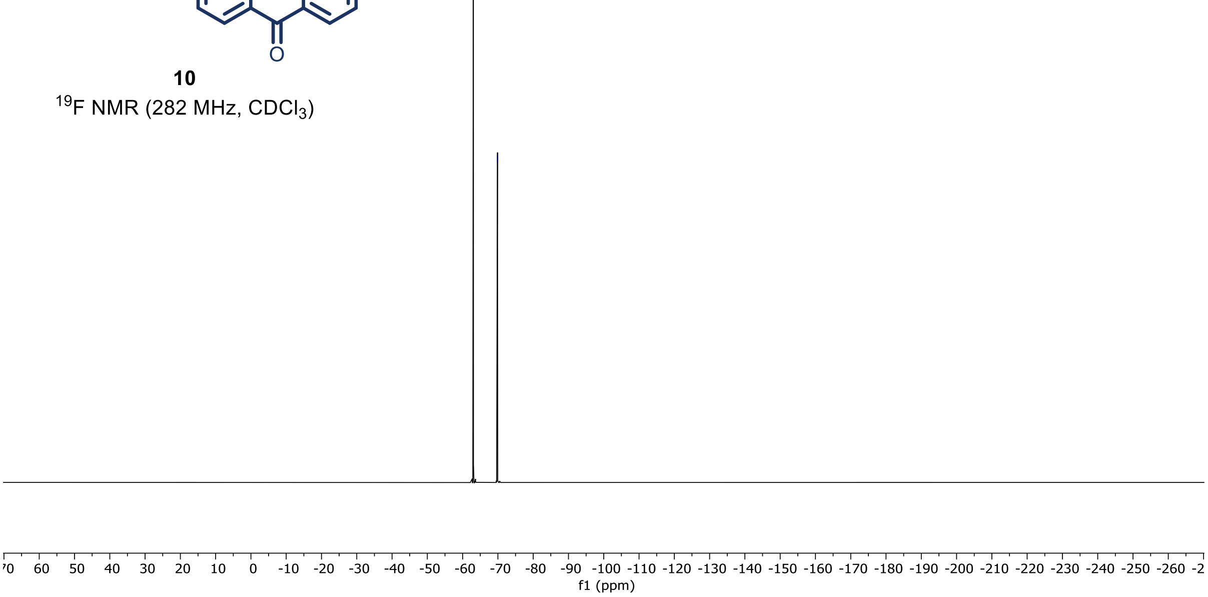

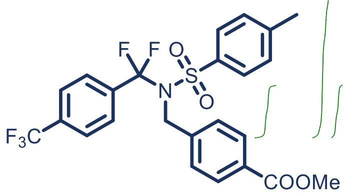

11

^1H NMR (300 MHz, $CDCl_3$)

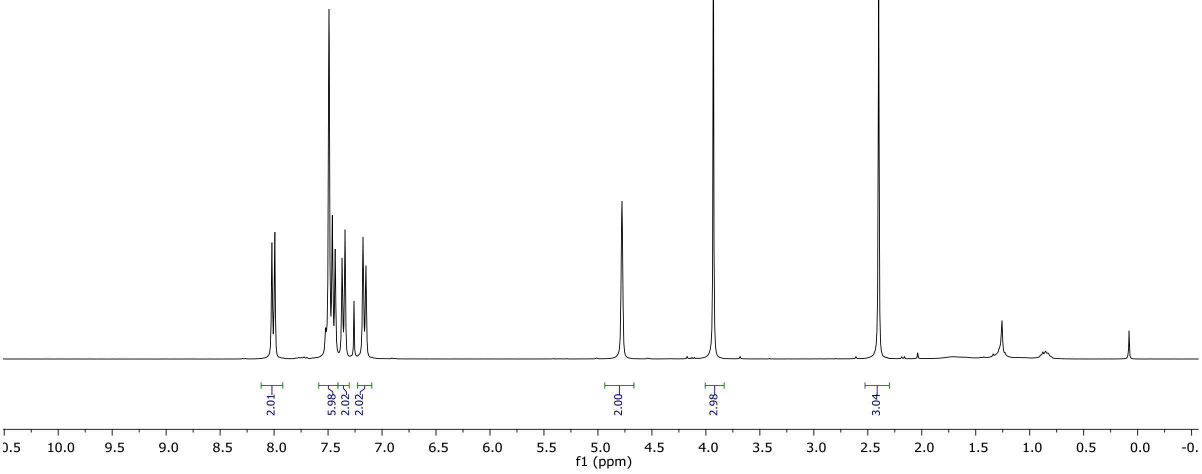

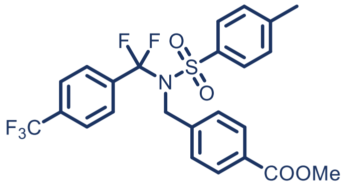

11

^{13}C NMR (75 MHz, $CDCl_3$)

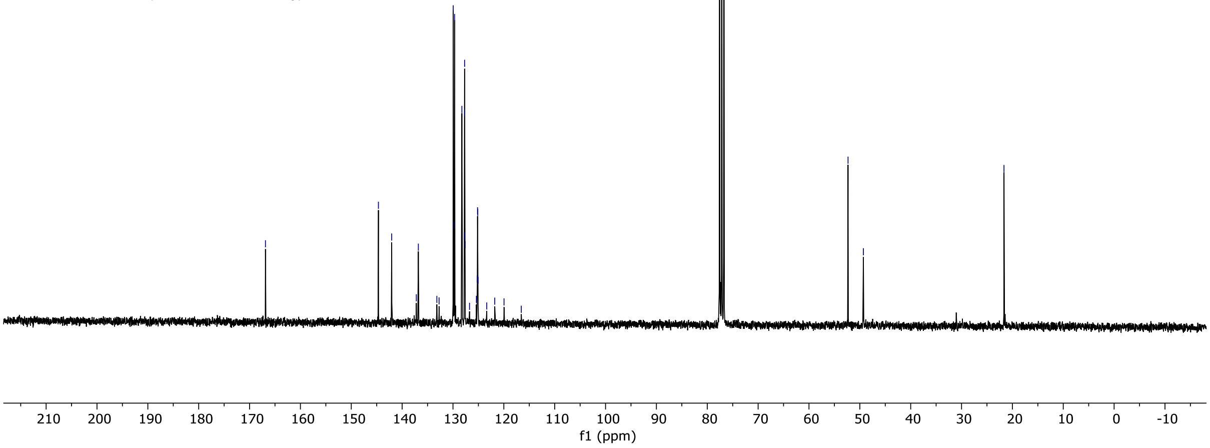

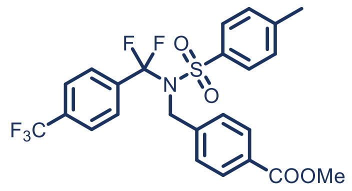

11

^{19}F NMR (282 MHz, $CDCl_3$)

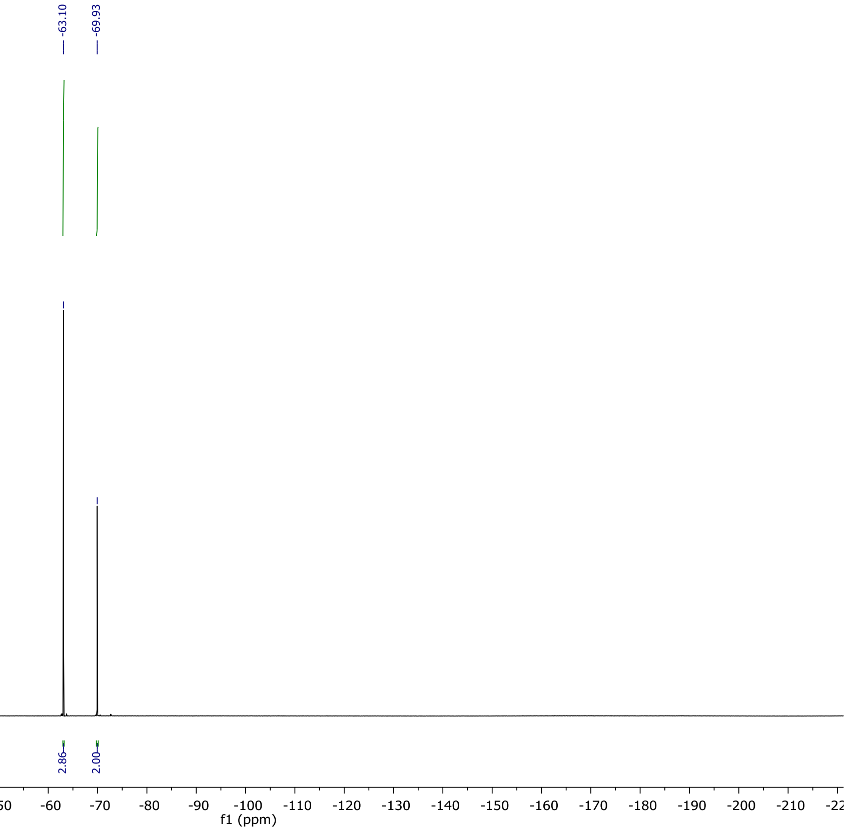

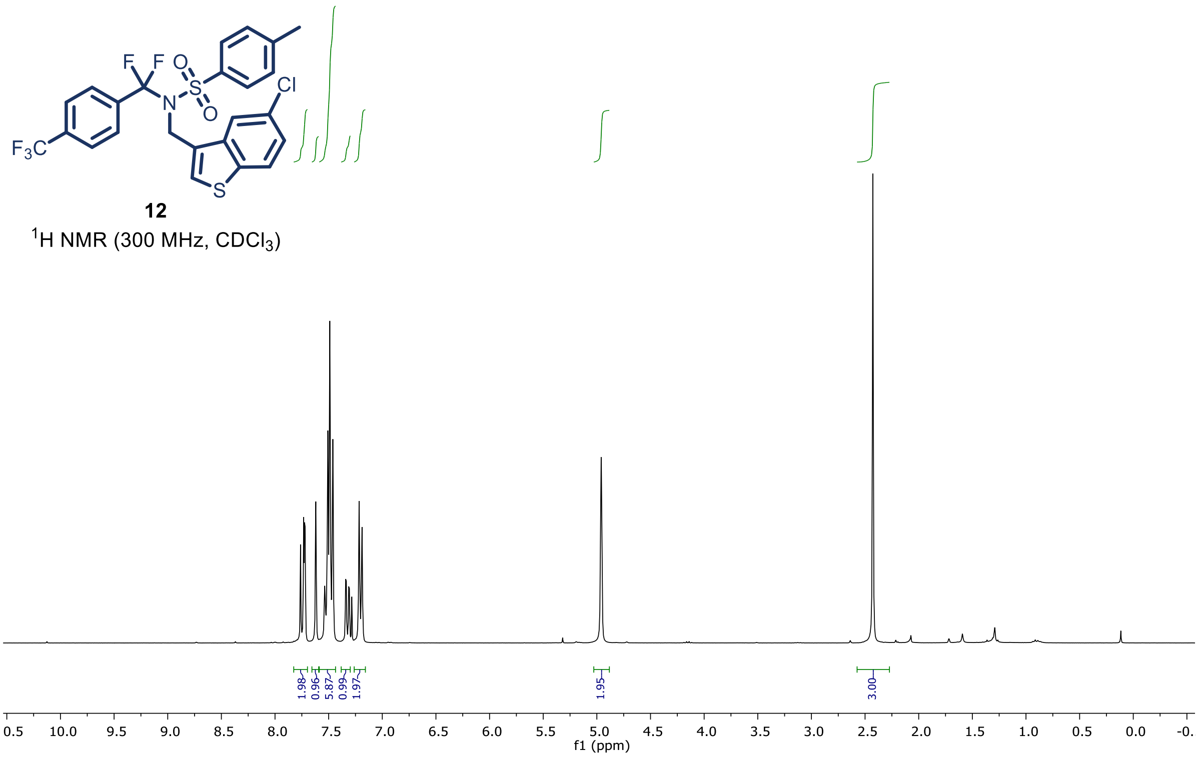
12
1H NMR (300 MHz, CDCl3)
1.98
0.96
5.87
0.99
1.97
1.95
3.00
f1 (ppm)

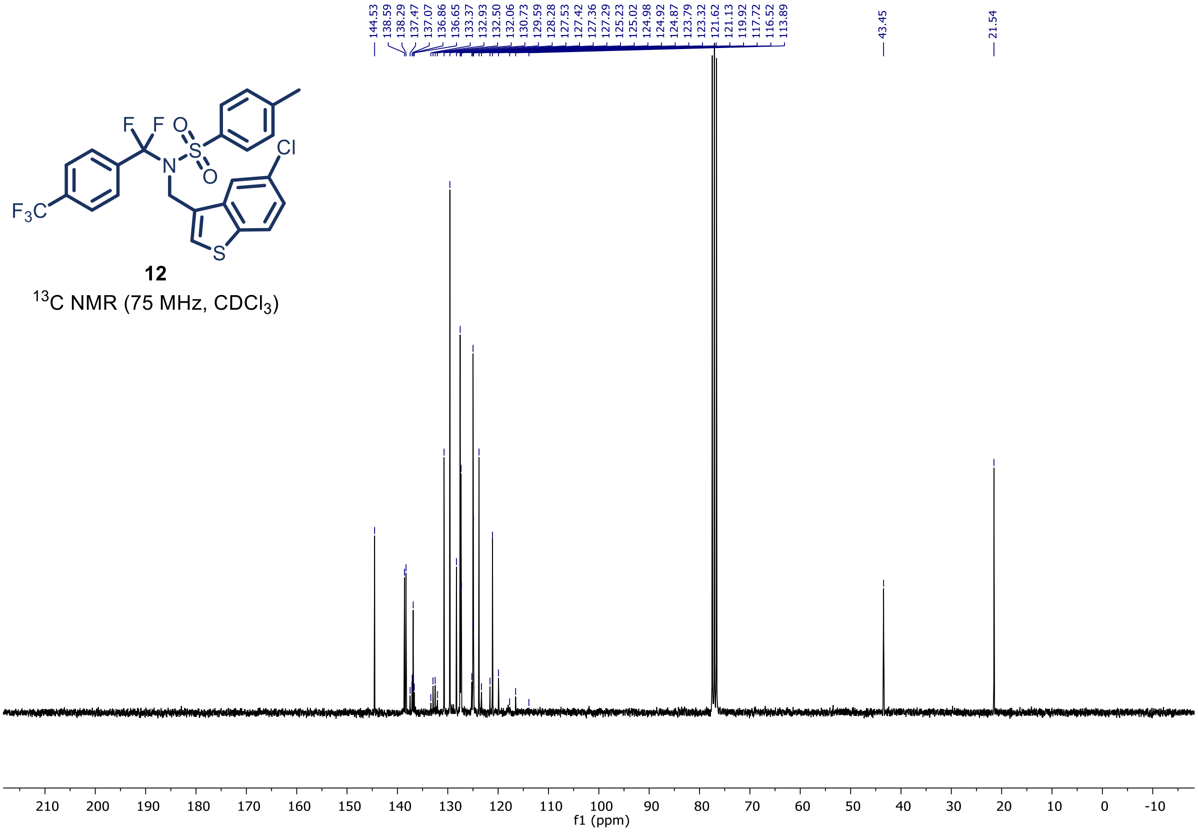
144.53
138.59
138.29
137.47
137.07
136.86
136.65
133.37
132.93
132.50
132.06
130.73
129.59
128.28
127.53
127.42
127.36
127.29
125.23
125.02
124.98
124.92
124.87
123.79
123.32
121.62
121.13
119.92
117.72
116.52
113.89
43.45
21.54
12
13C NMR (75 MHz, CDCl3)
f1 (ppm)

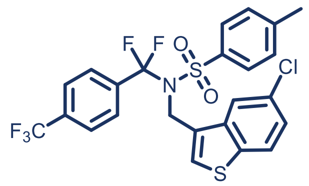

12

^{19}F NMR (282 MHz, $CDCl_3$)

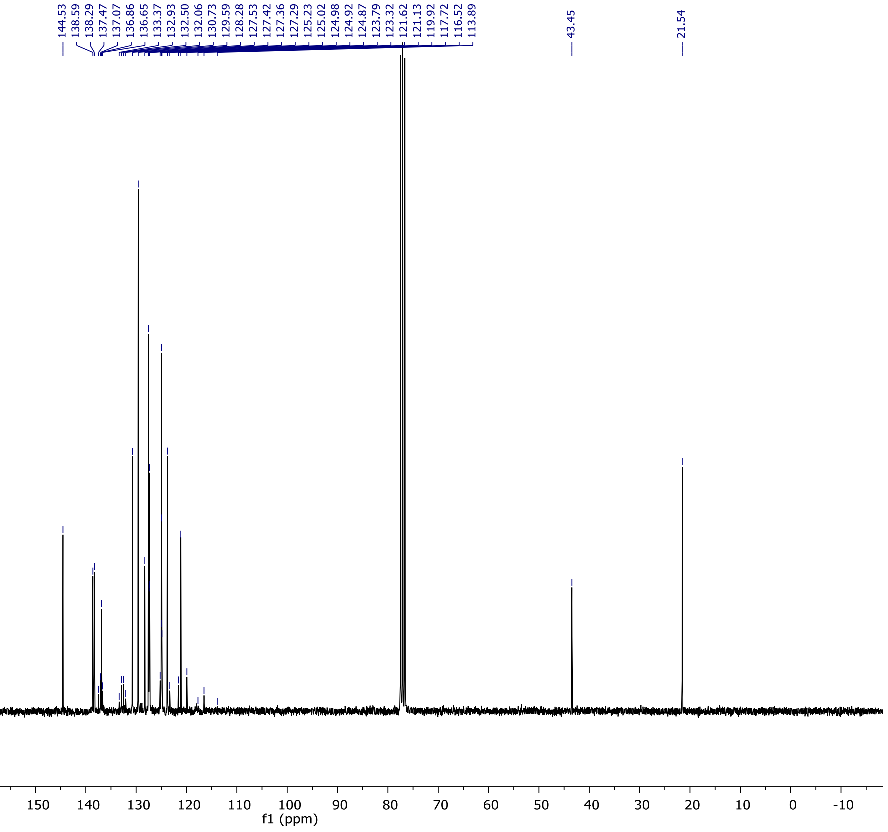

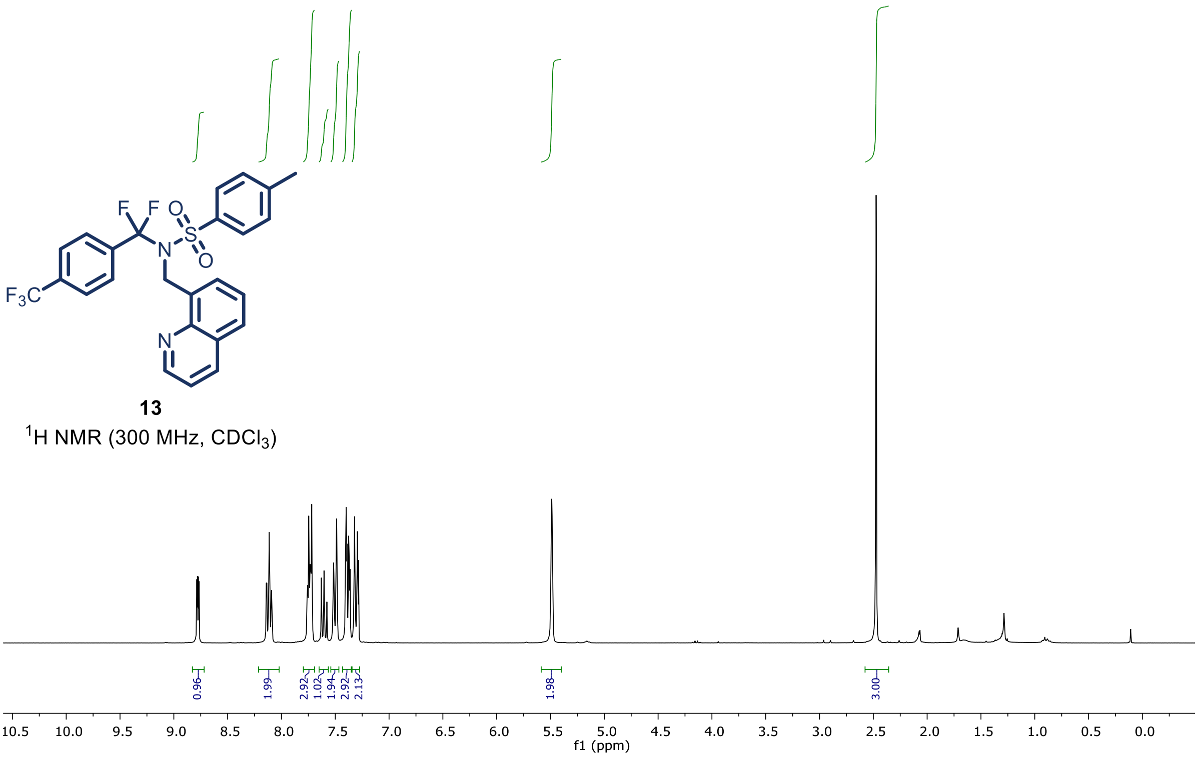

13

^{1}H NMR (300 MHz, $CDCl_3$)

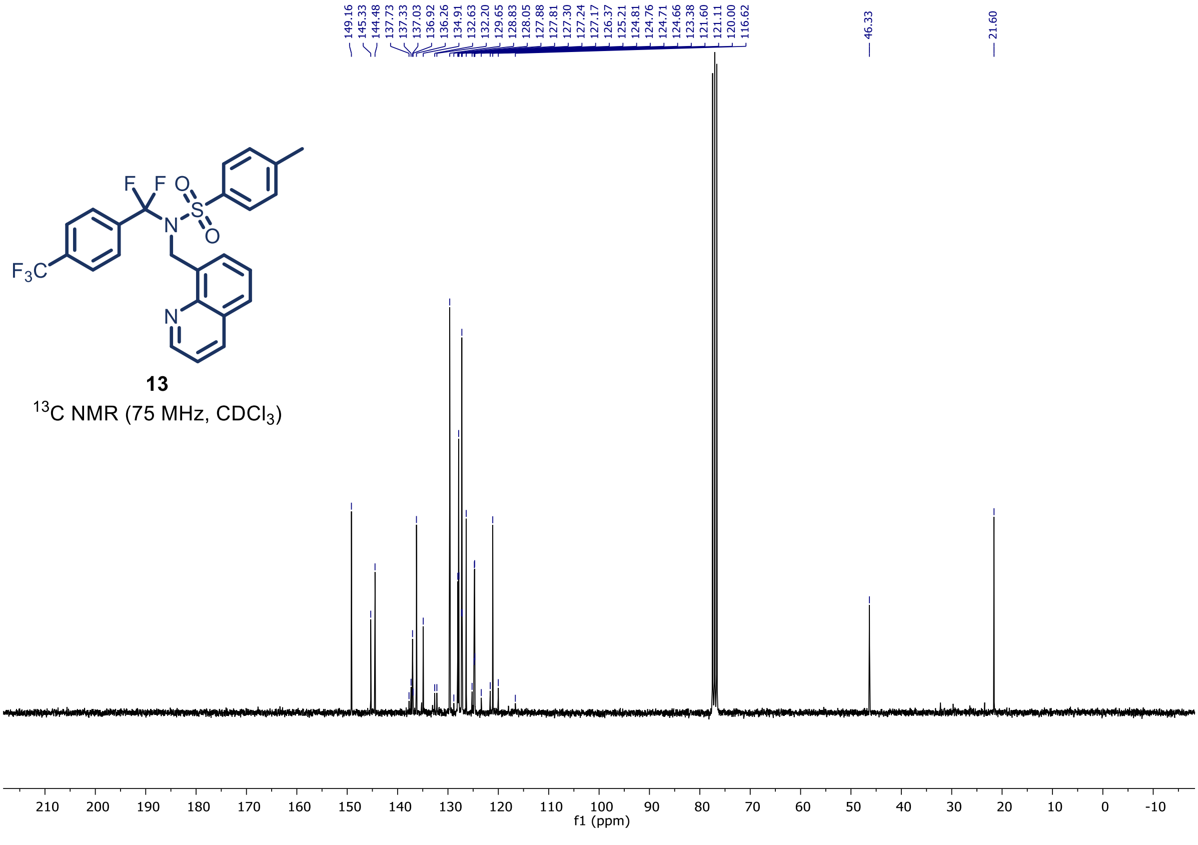

13

^{13}C NMR (75 MHz, $CDCl_3$)

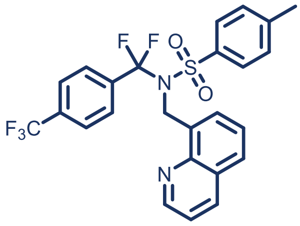

13

^{19}F NMR (282 MHz, $CDCl_3$)

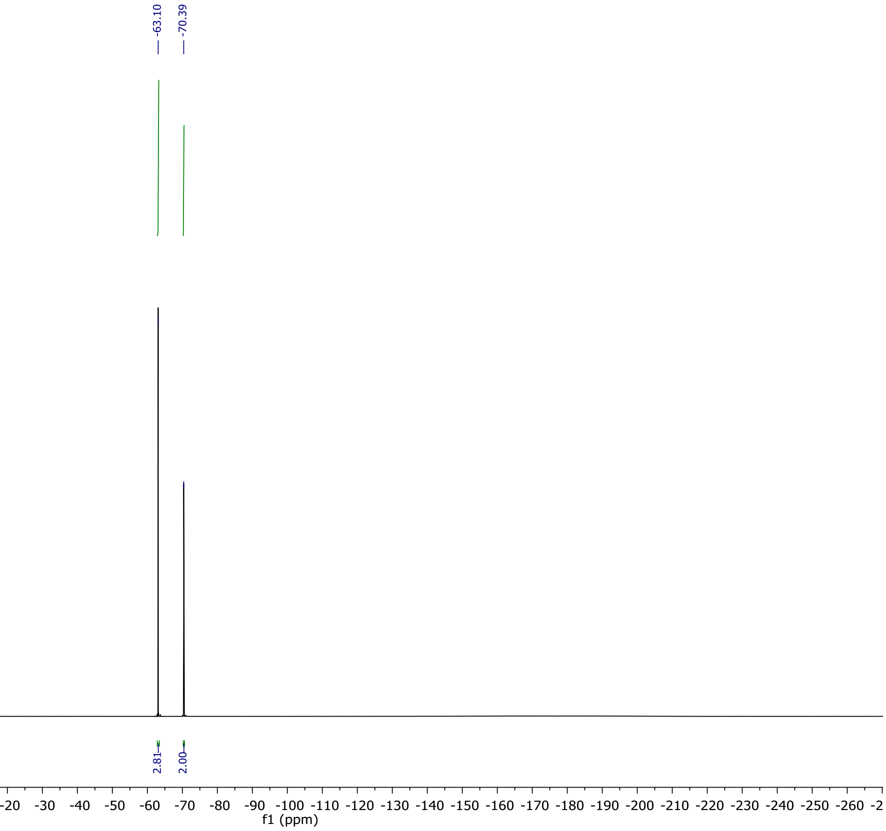

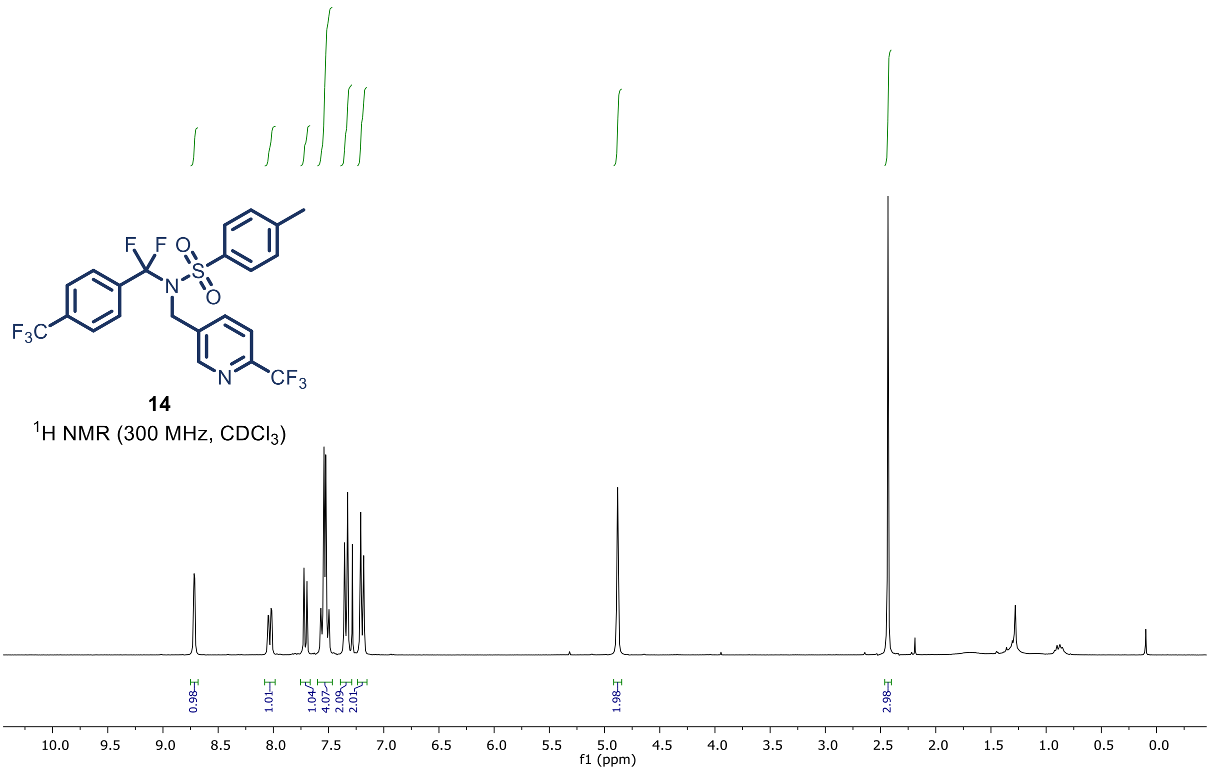

F F O
S
N
O
F3C
N CF3
14
1H NMR (300 MHz, CDCl3)
0.98
1.01
1.04
4.07
2.09
2.01
1.98
2.98
10.0 9.5 9.0 8.5 8.0 7.5 7.0 6.5 6.0 5.5 5.0 4.5 4.0 3.5 3.0 2.5 2.0 1.5 1.0 0.5 0.0
f1 (ppm)

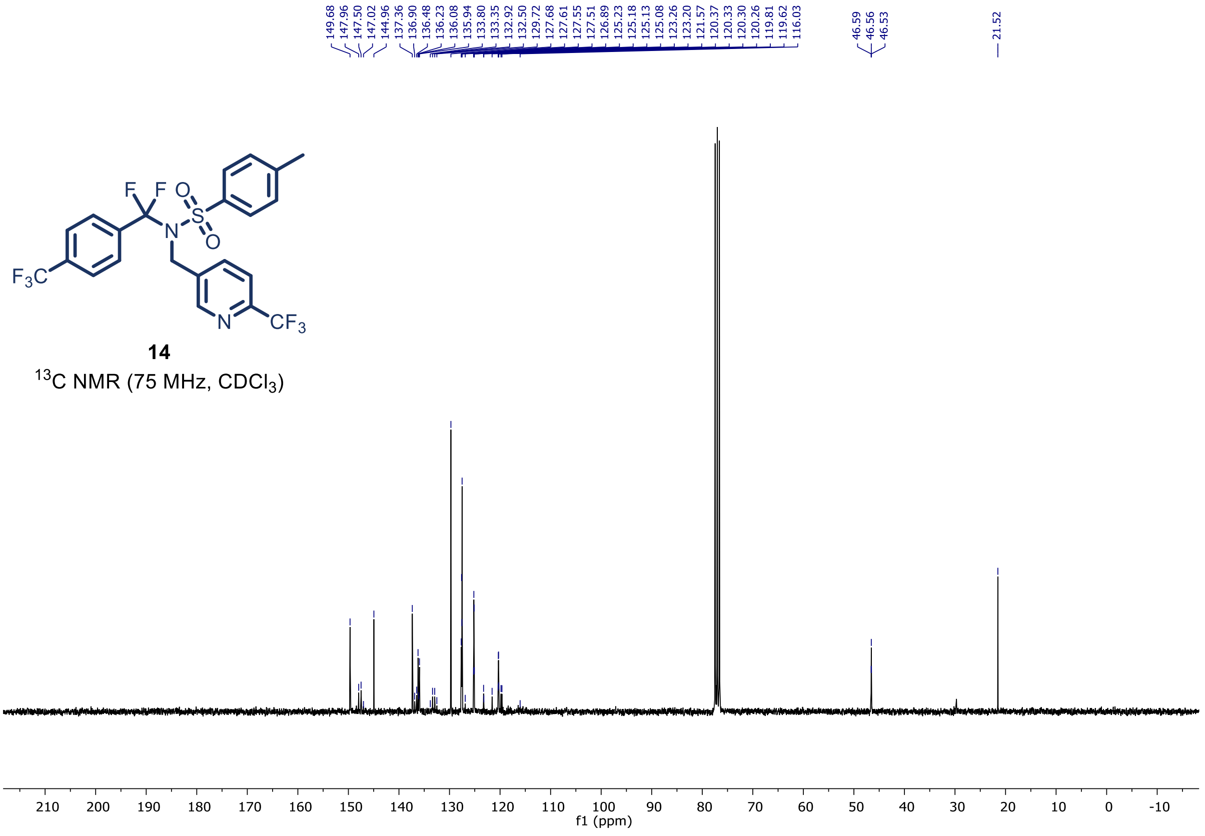

149.68
147.96
147.50
147.02
144.96
137.36
136.90
136.48
136.23
136.08
135.94
133.80
133.35
132.92
132.50
129.72
127.68
127.61
127.55
127.51
126.89
125.23
125.18
125.13
125.08
123.26
123.20
121.57
120.37
120.33
120.30
120.26
119.81
119.62
116.03
46.59
46.56
46.53
21.52
F F O
S
N
O
F3C
N CF3
14
13C NMR (75 MHz, CDCl3)
210 200 190 180 170 160 150 140 130 120 110 100 90 80 70 60 50 40 30 20 10 0 -10
f1 (ppm)

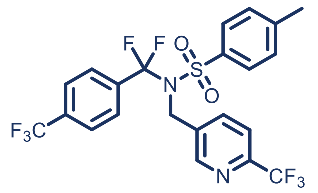

14

^{19}F NMR (282 MHz, $CDCl_3$)

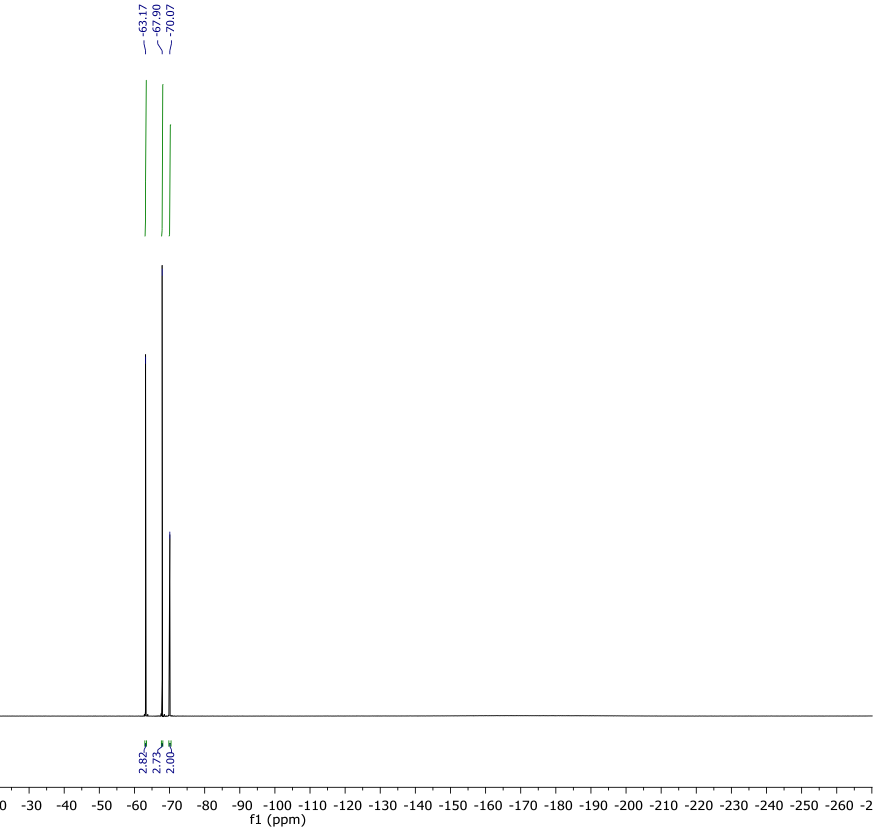

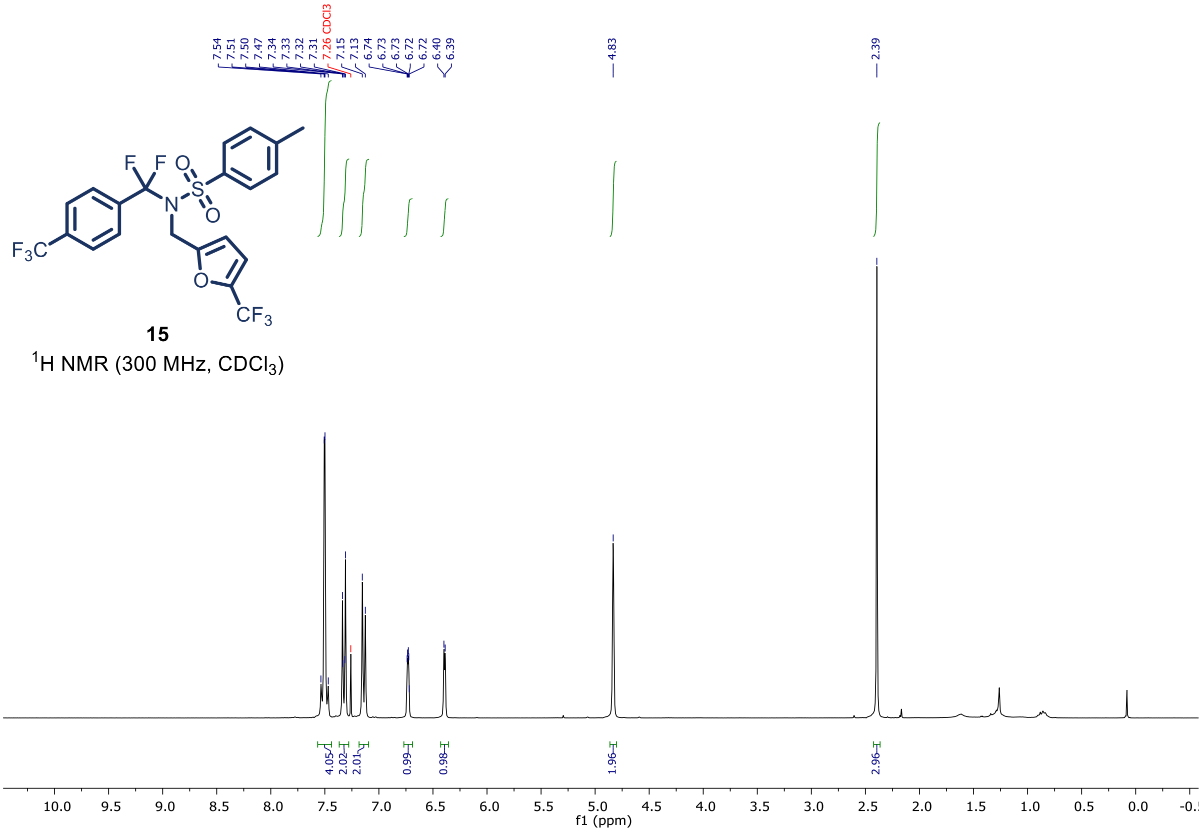
15
^1H NMR (300 MHz, $CDCl_3$)
f1 (ppm)

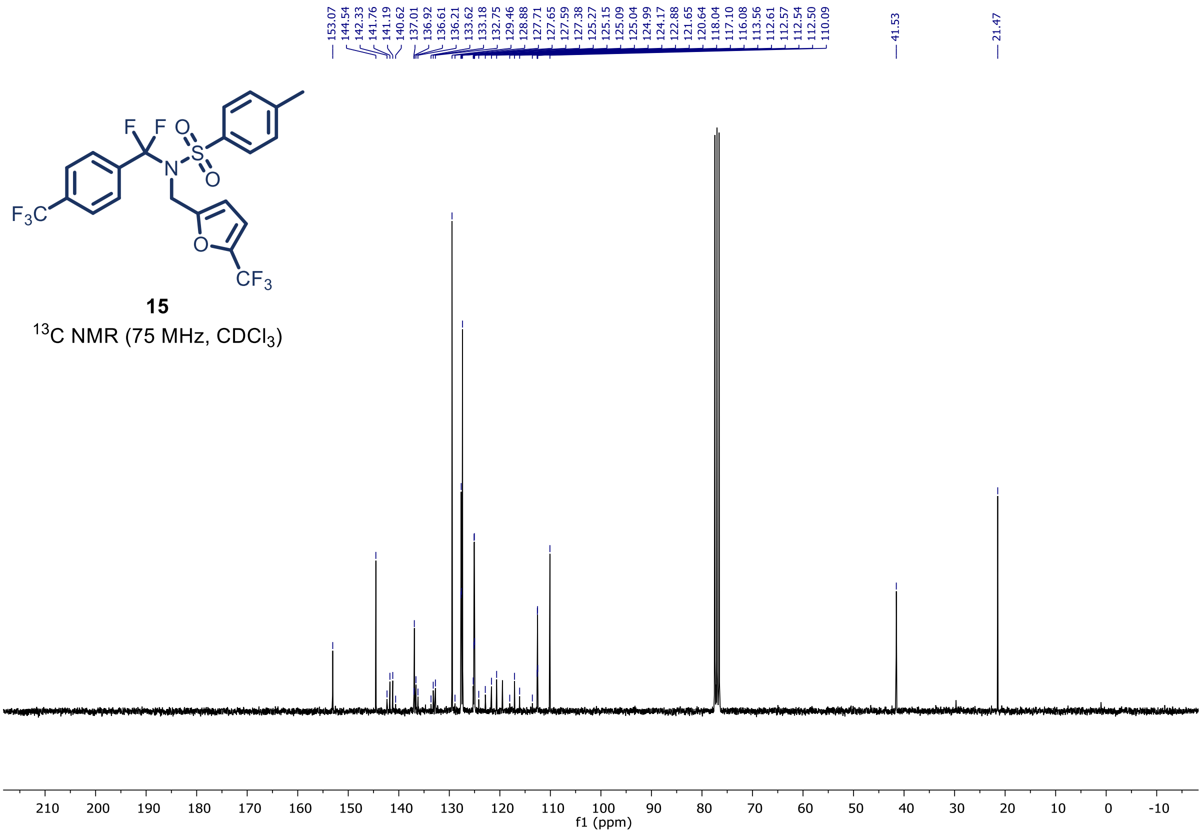
15
^{13}C NMR (75 MHz, $CDCl_3$)
f1 (ppm)

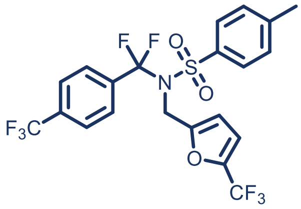

15

^{19}F NMR (282 MHz, $CDCl_3$)

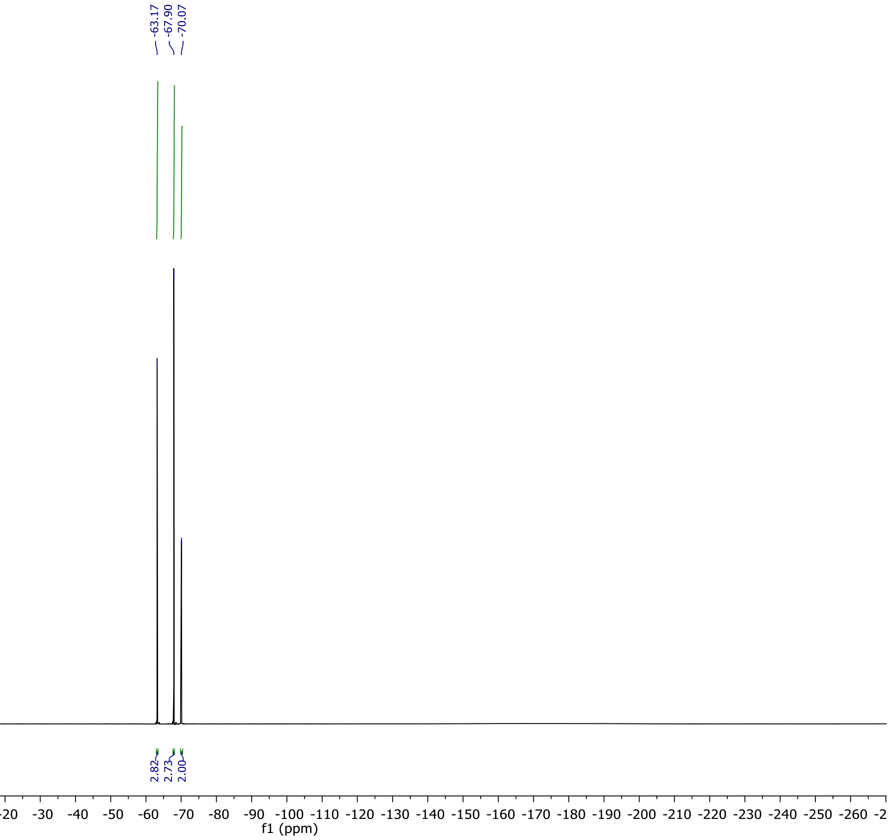

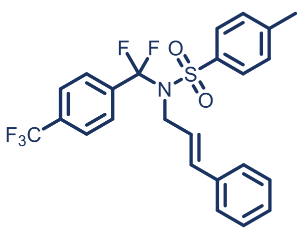

16

^{1}H NMR (300 MHz, $CDCl_3$)

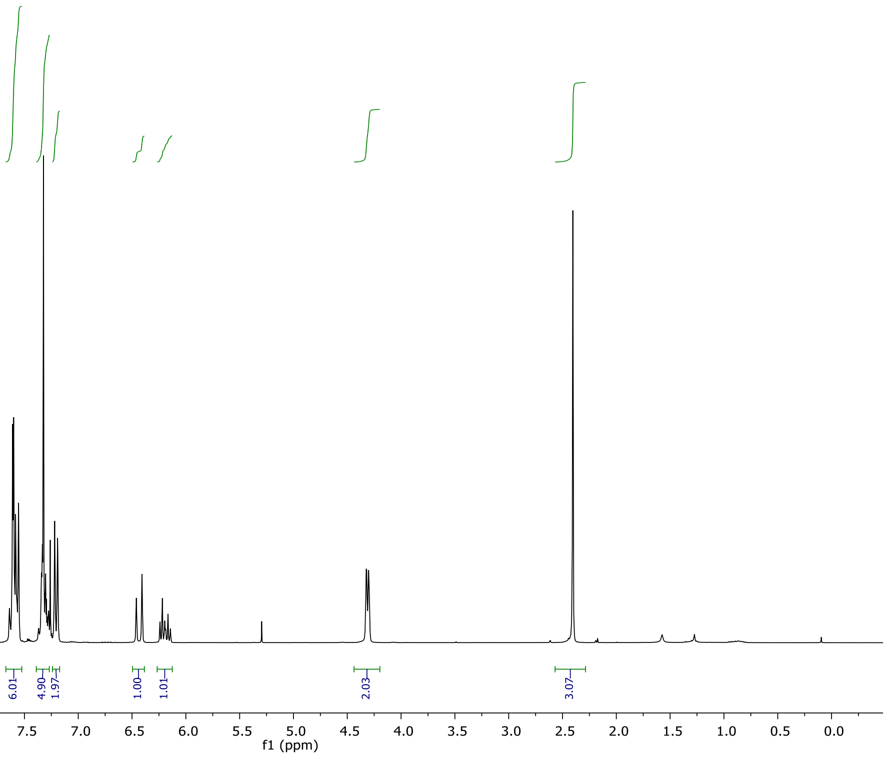

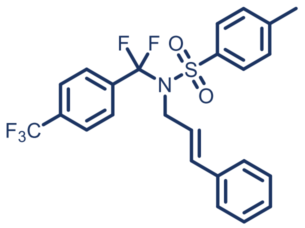

16

^{13}C NMR (75 MHz, $CDCl_3$)

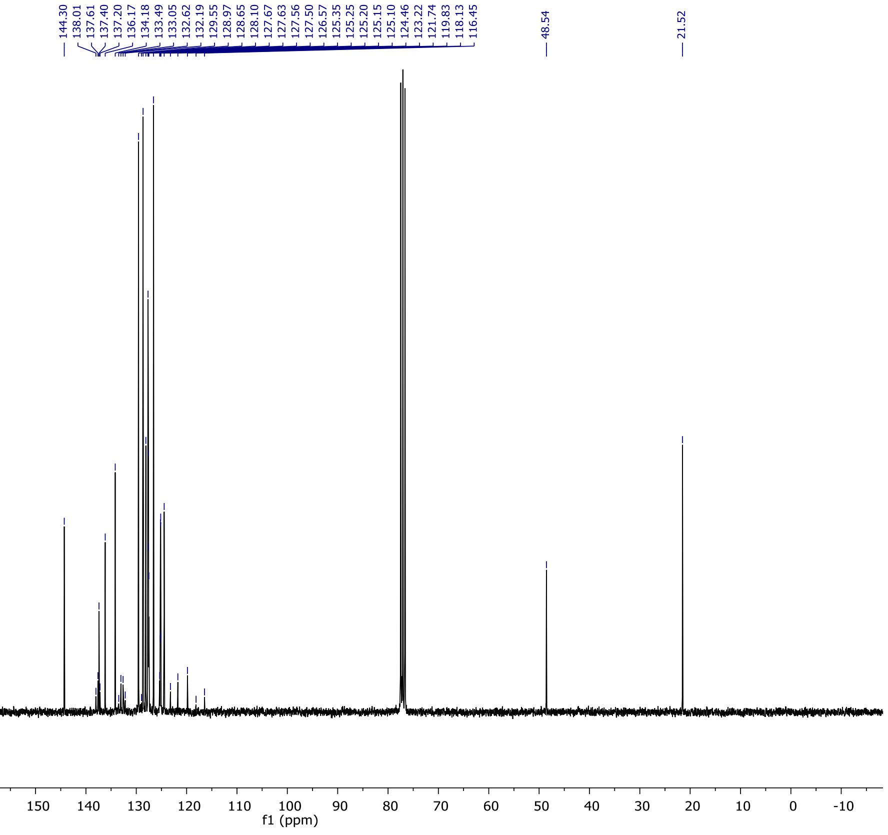

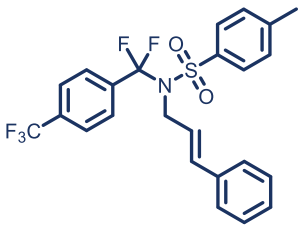

16

^{19}F NMR (282 MHz, $CDCl_3$)

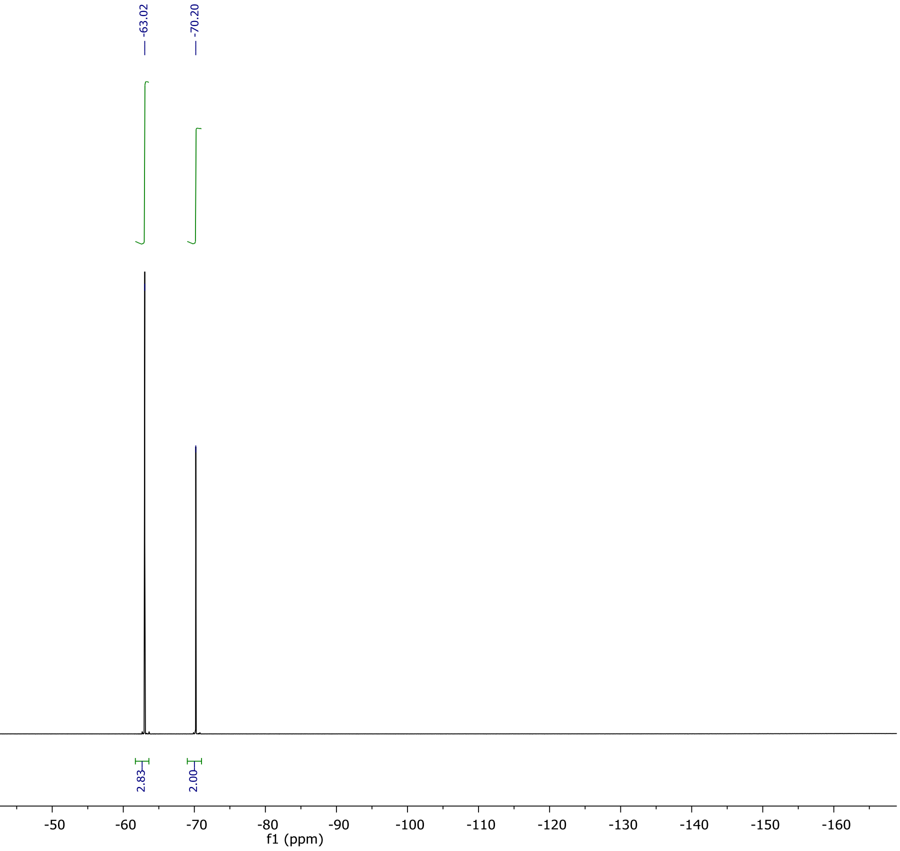

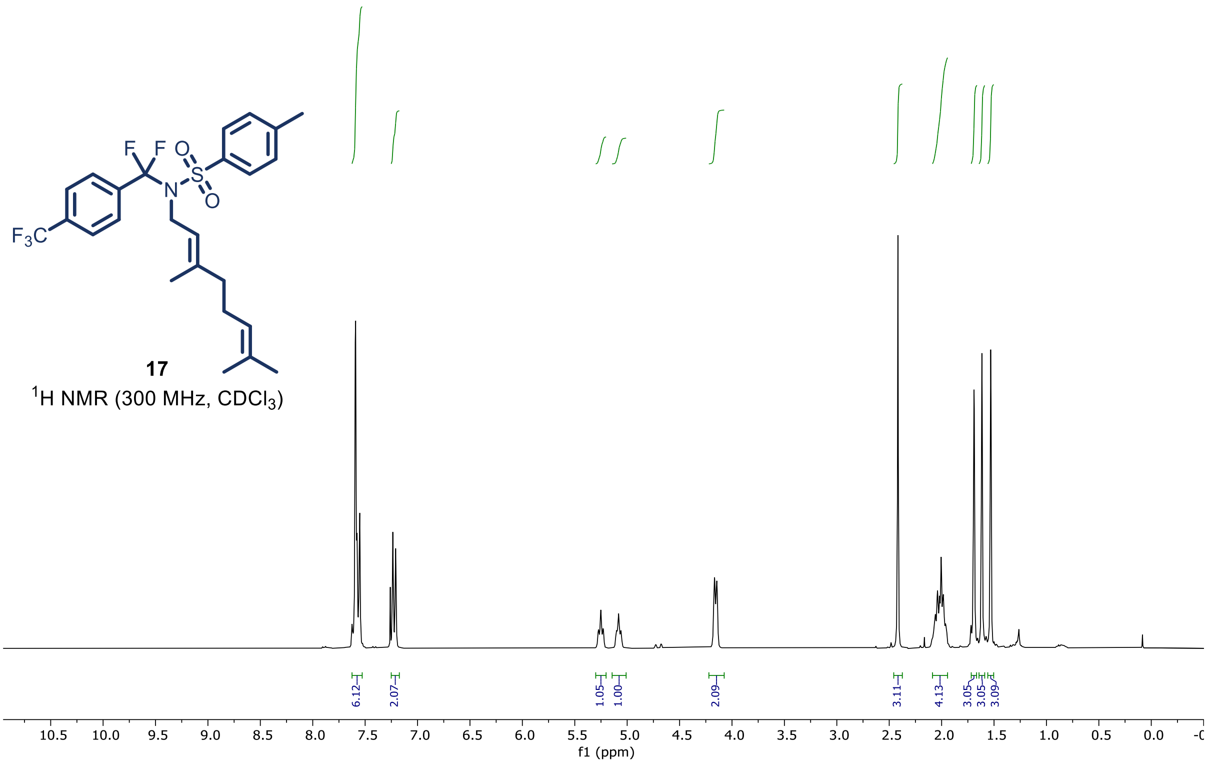

17

^{1}H NMR (300 MHz, $CDCl_3$)

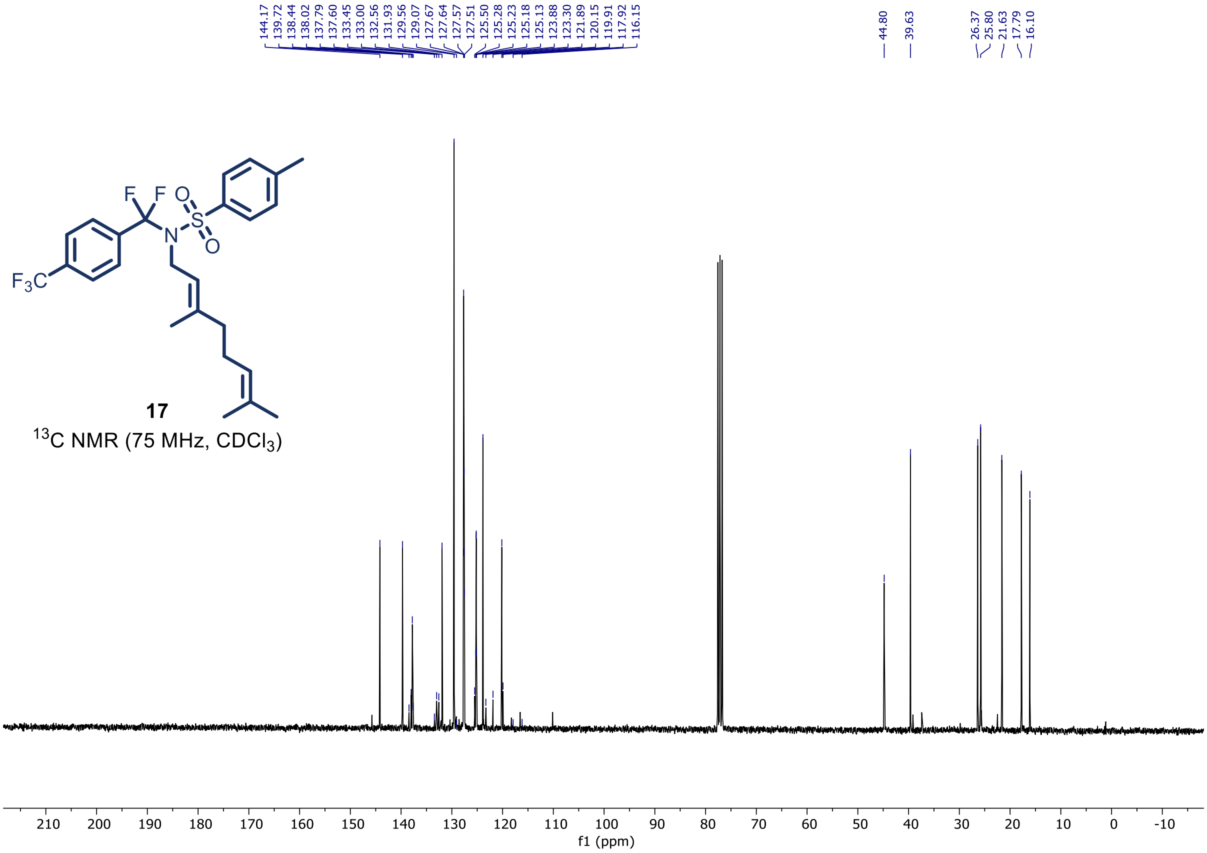

17

^{13}C NMR (75 MHz, $CDCl_3$)

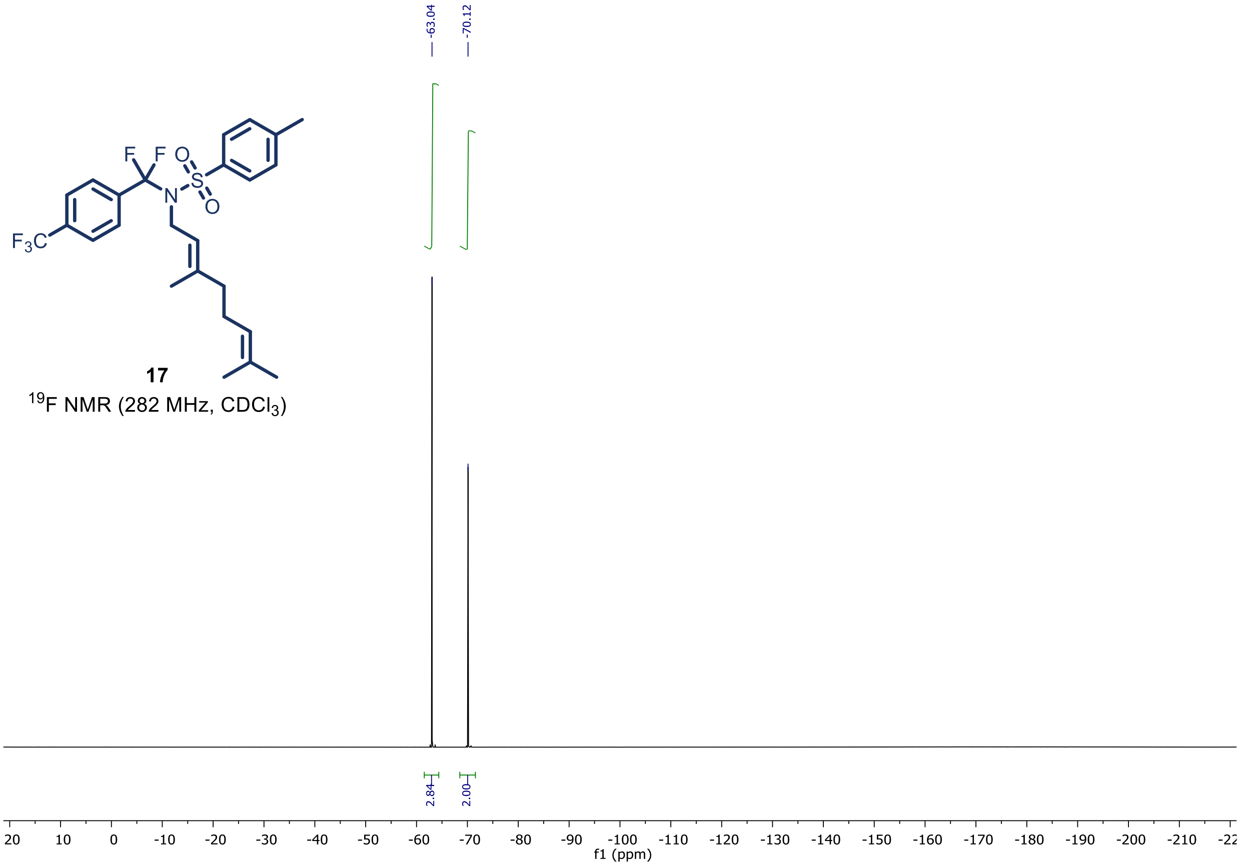
17
19F NMR (282 MHz, CDCl3)
-63.04
-70.12
2.84
2.00
f1 (ppm)

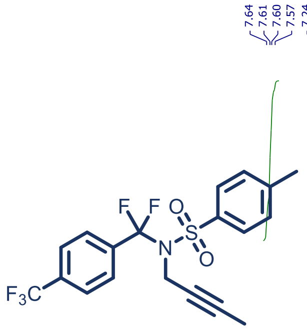

18

^1H NMR (300 MHz, $CDCl_3$)

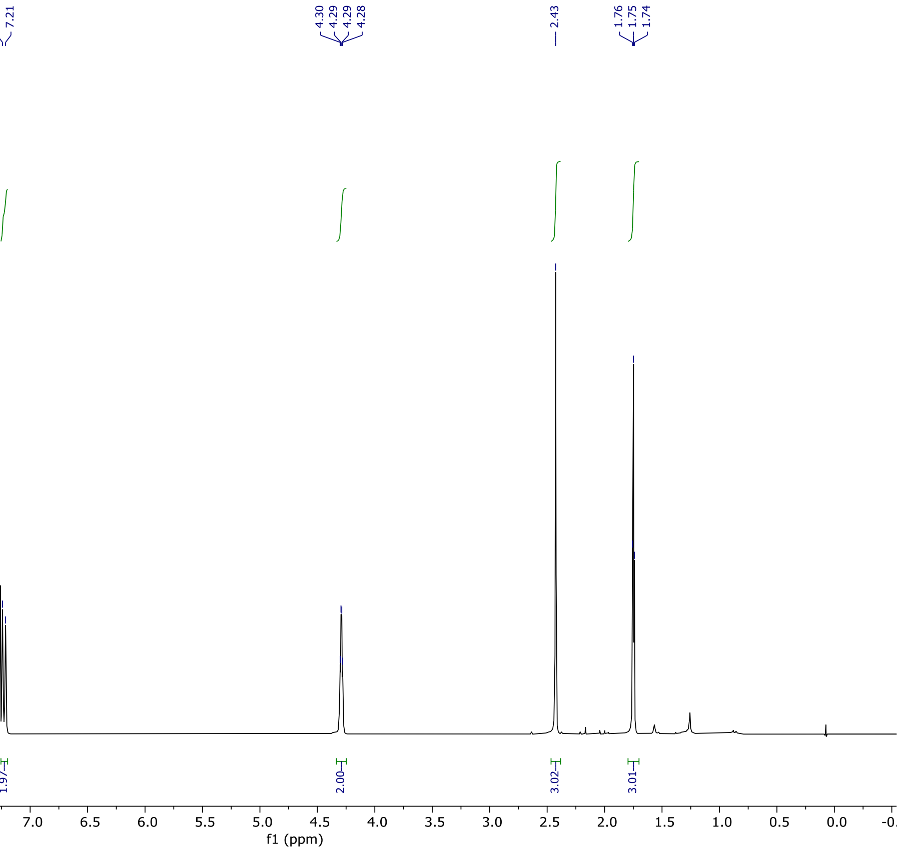

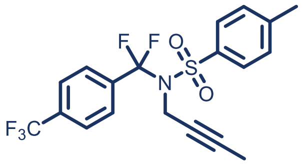

18

^{13}C NMR (75 MHz, $CDCl_3$)

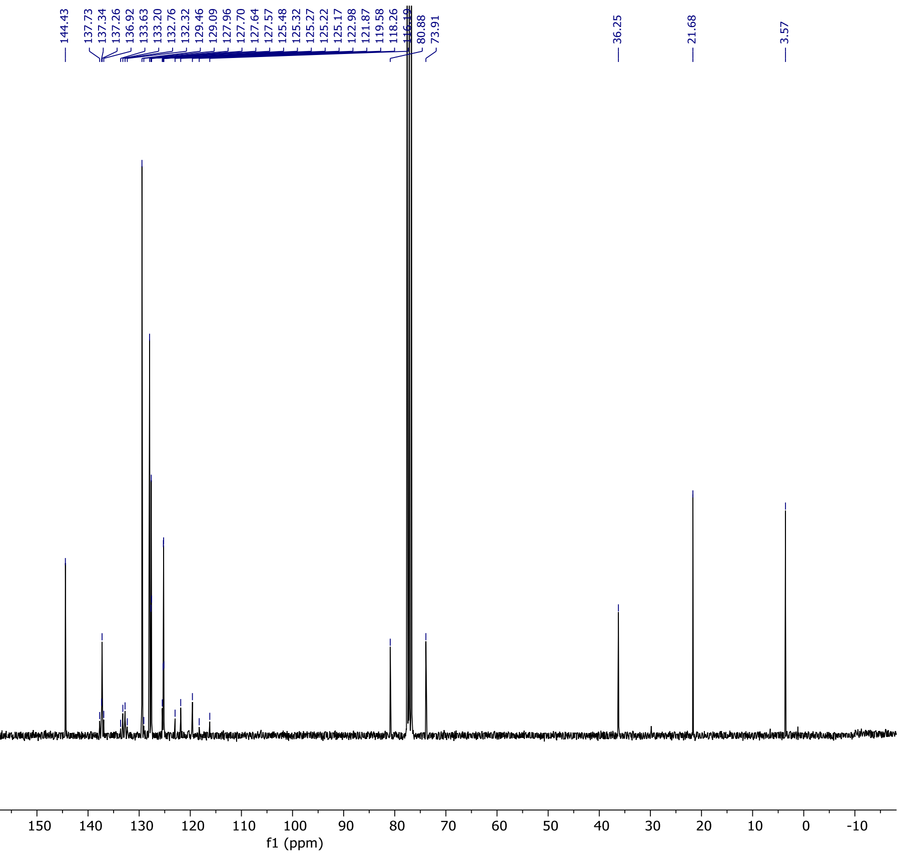

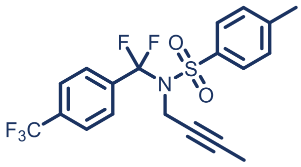

18

^{19}F NMR (282 MHz, $CDCl_3$)

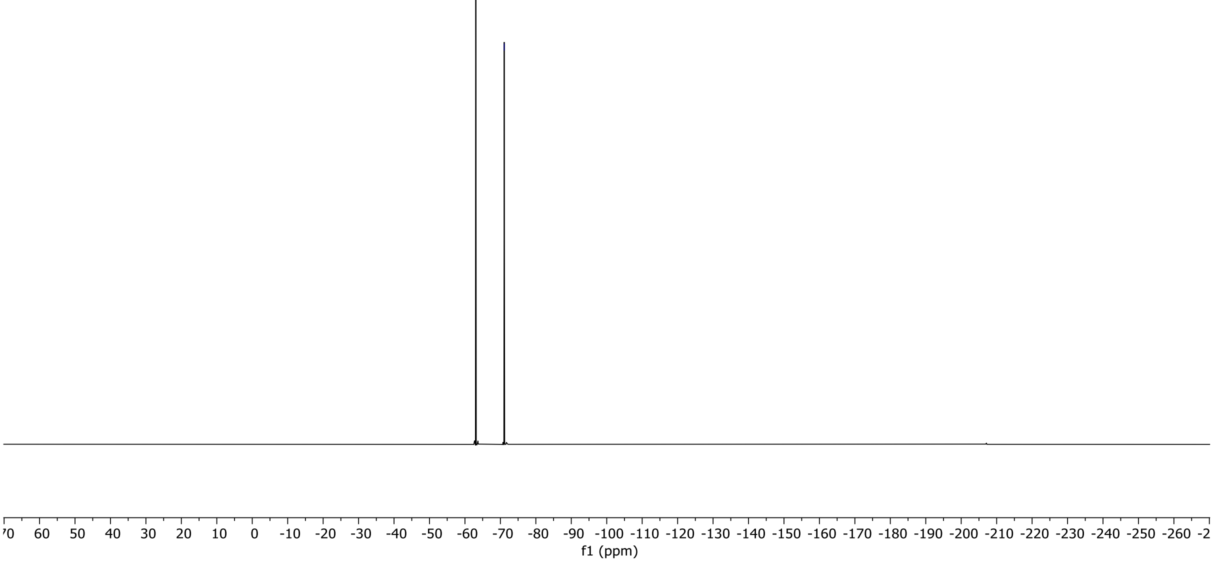

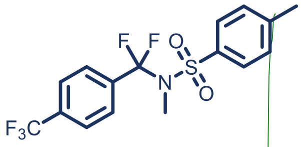

19

^{1}H NMR (300 MHz, $CDCl_3$)

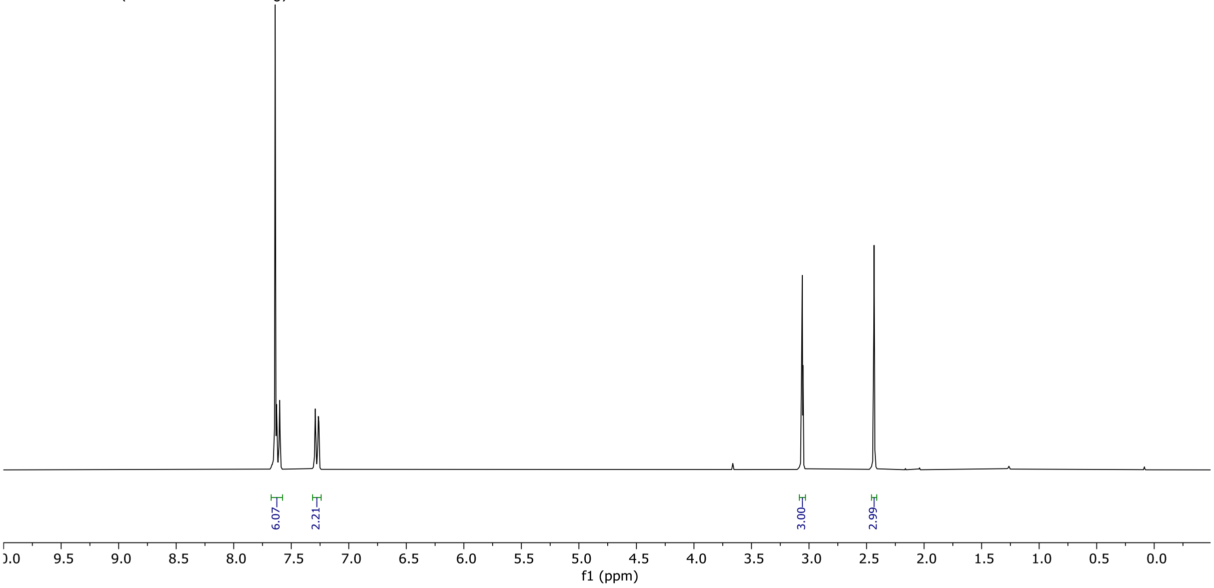

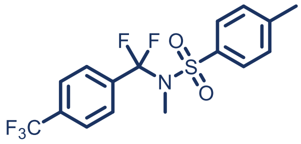

19

^{13}C NMR (75 MHz, $CDCl_3$)

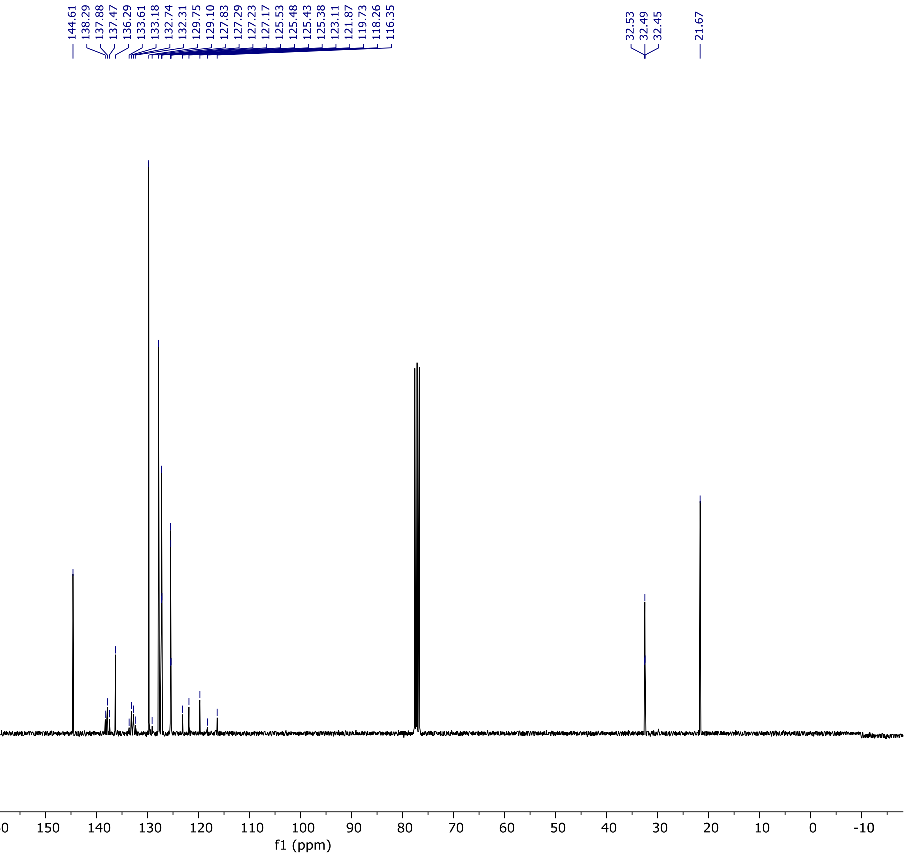

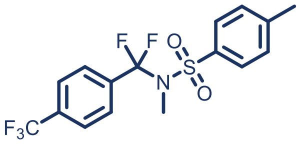

19

^{19}F NMR (282 MHz, $CDCl_3$)

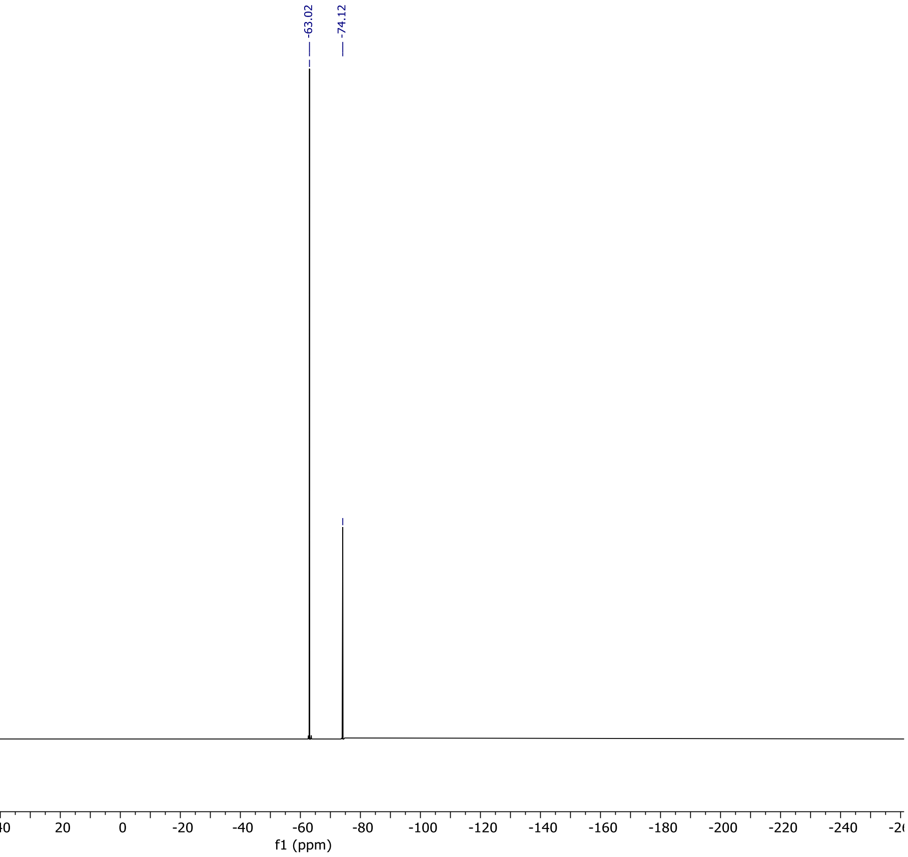

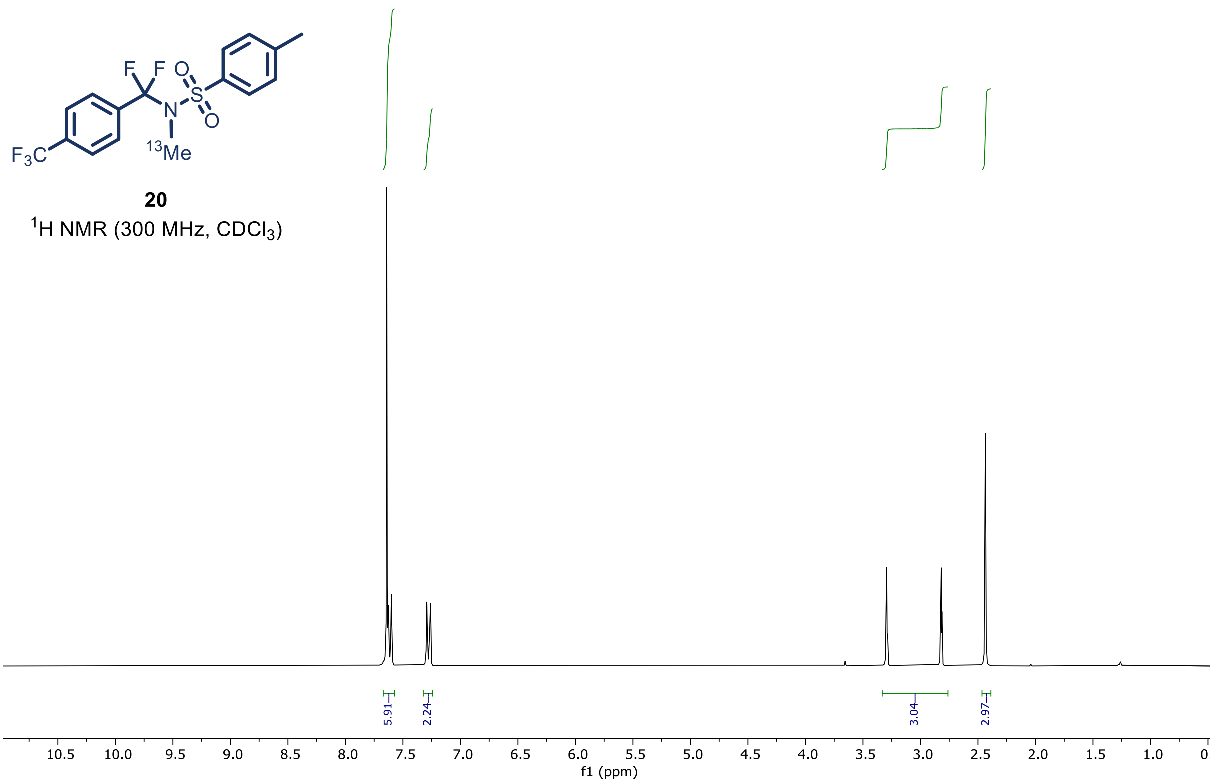

20
^{1}H NMR (300 MHz, $CDCl_3$)

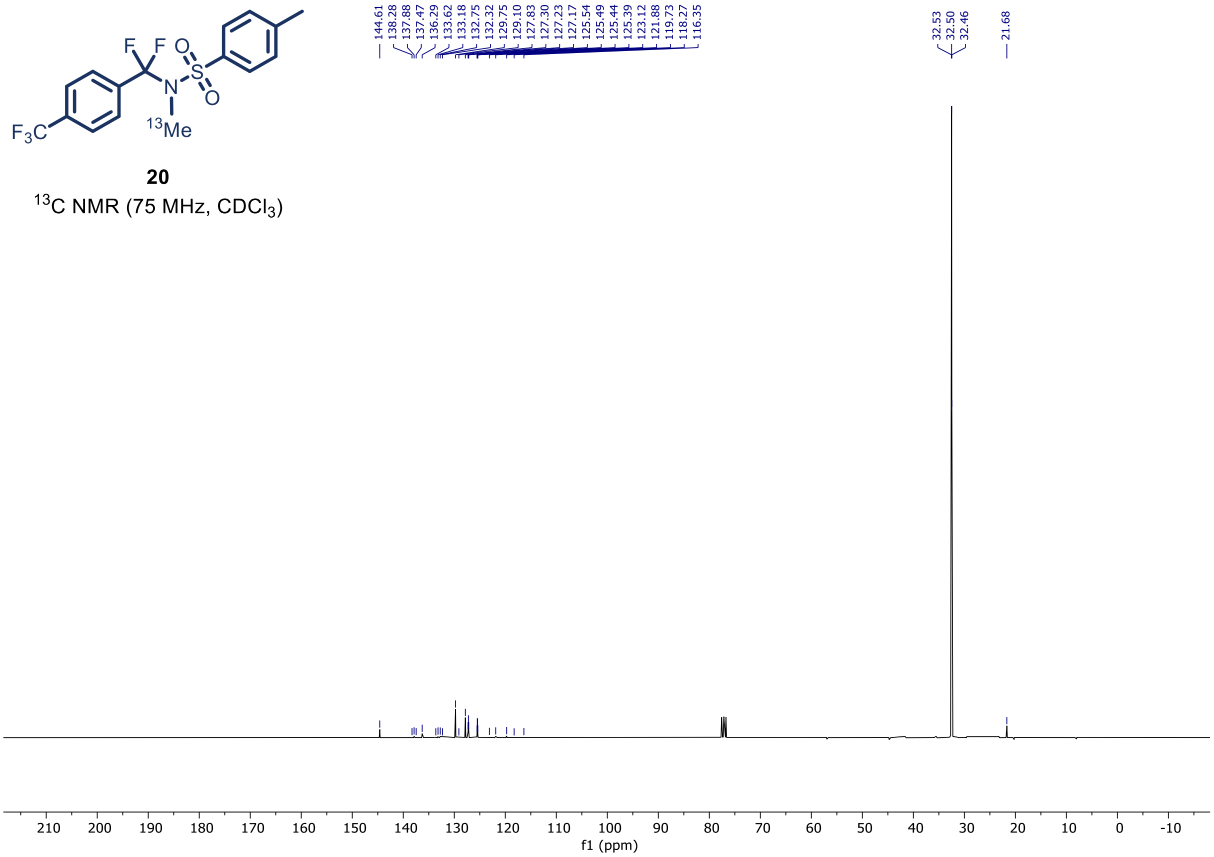

20
^{13}C NMR (75 MHz, $CDCl_3$)

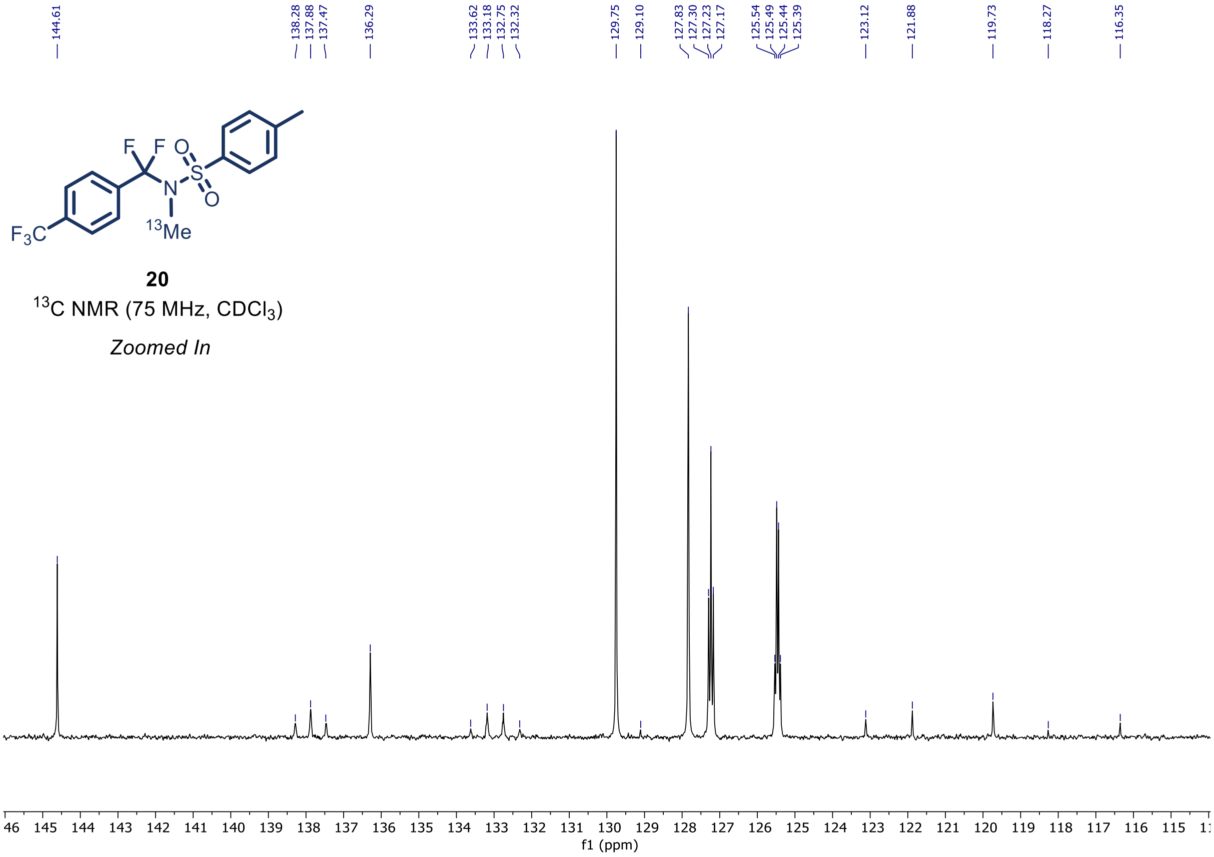

20

^{13}C NMR (75 MHz, $CDCl_3$)

Zoomed In

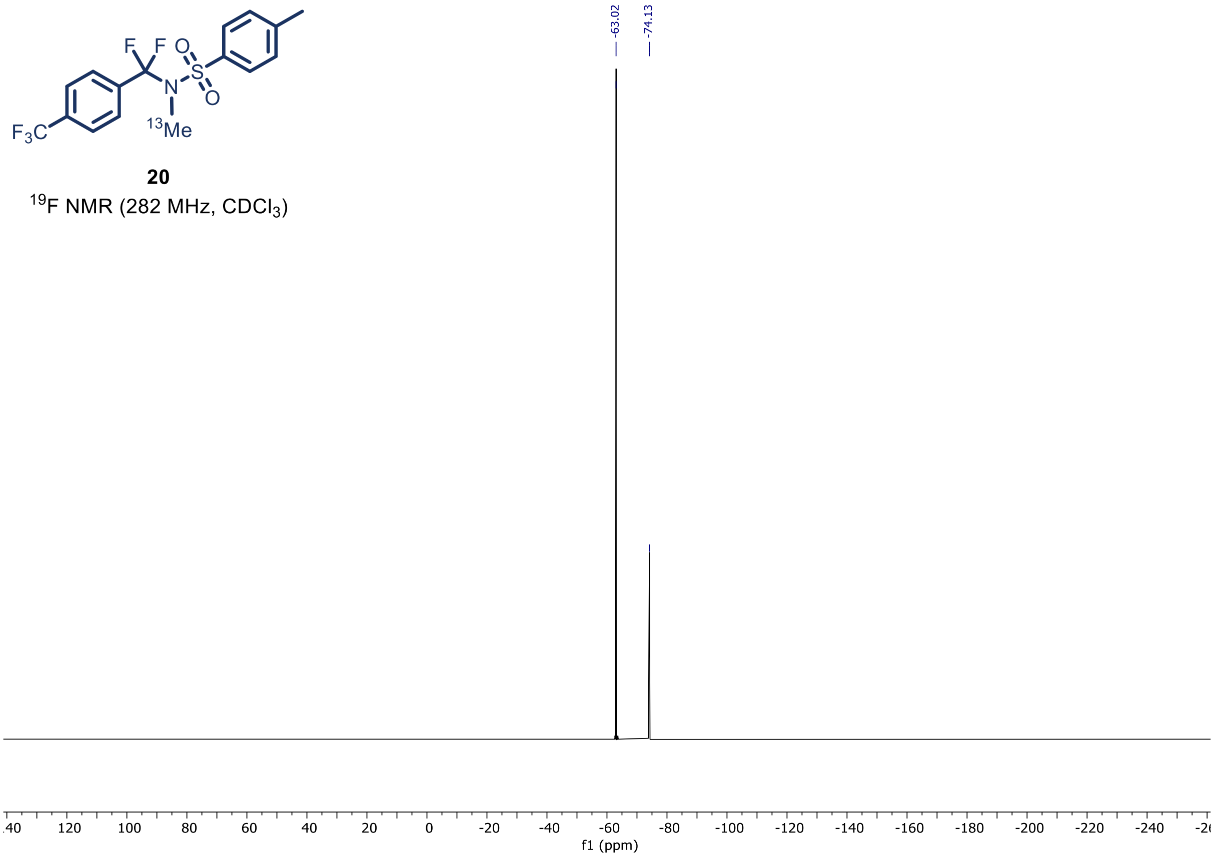

20

^{19}F NMR (282 MHz, $CDCl_3$)

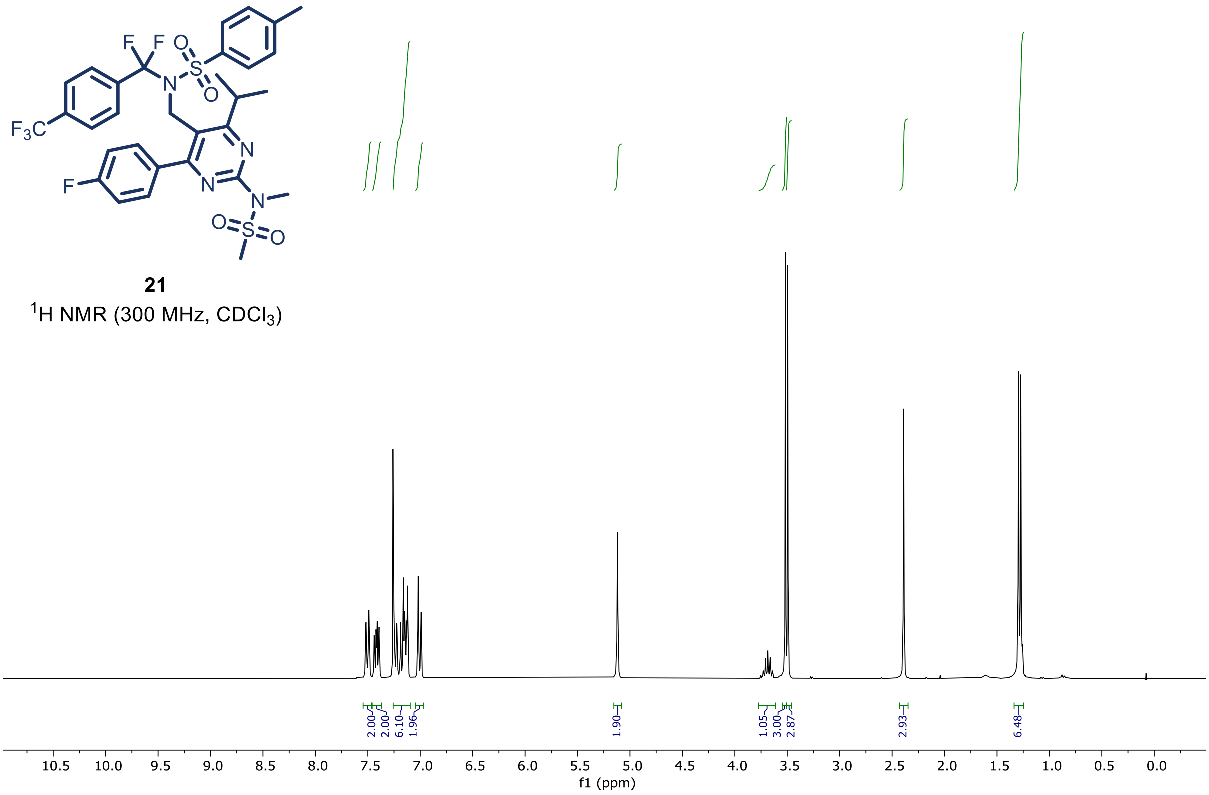

21
^{1}H NMR (300 MHz, $CDCl_3$)

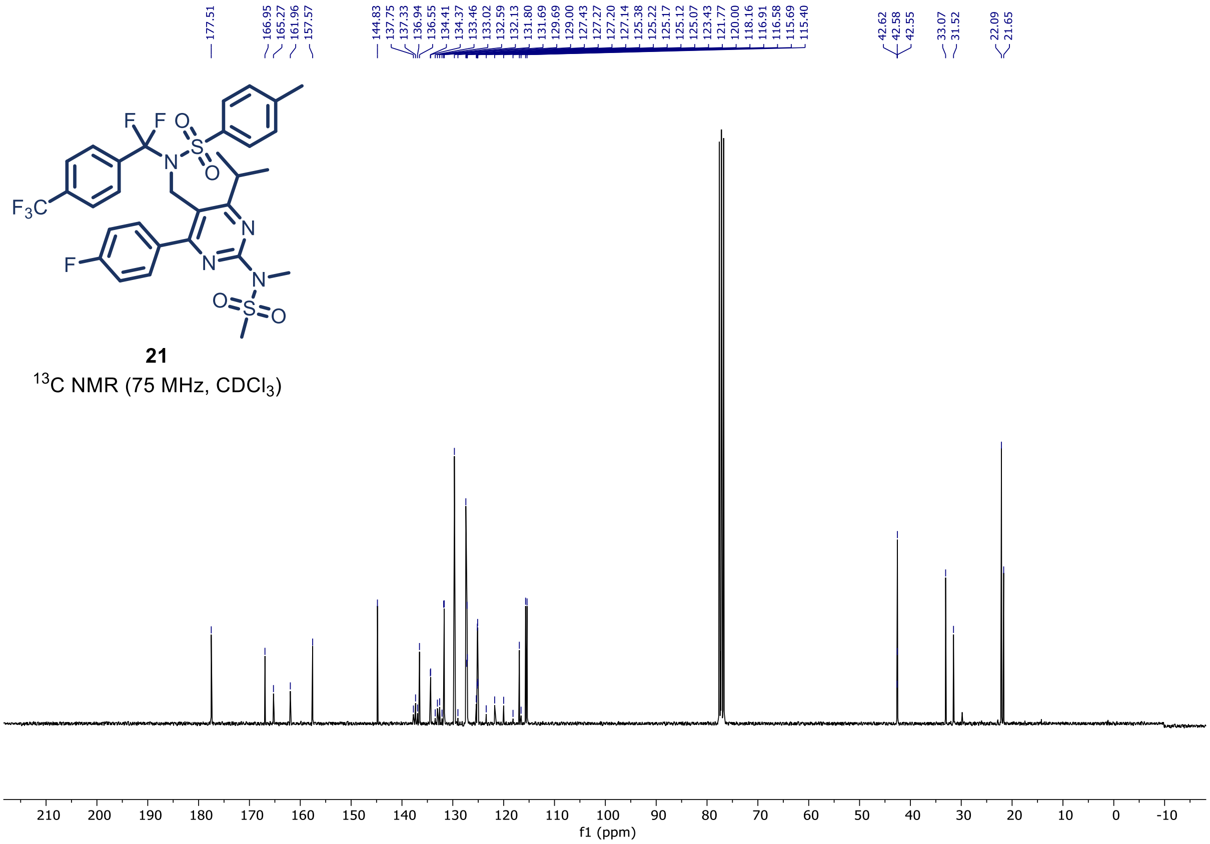

21
^{13}C NMR (75 MHz, $CDCl_3$)

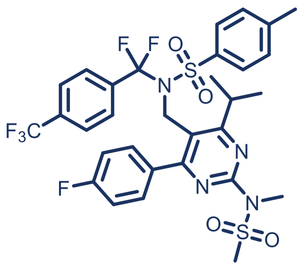

21

^{19}F NMR (282 MHz, $CDCl_3$)

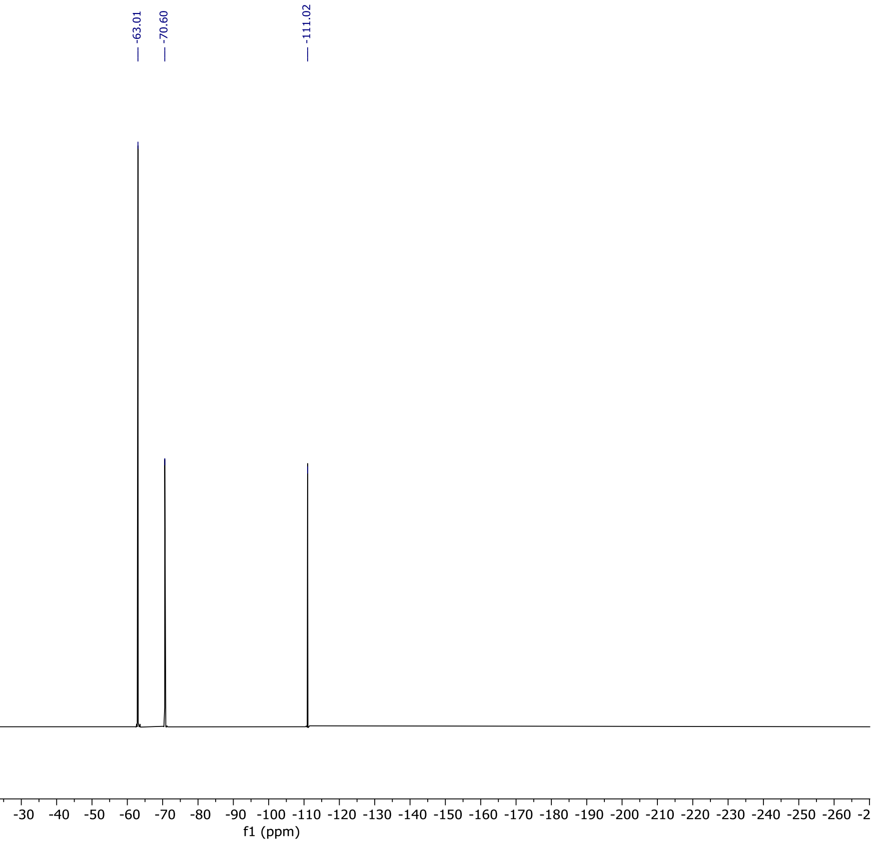

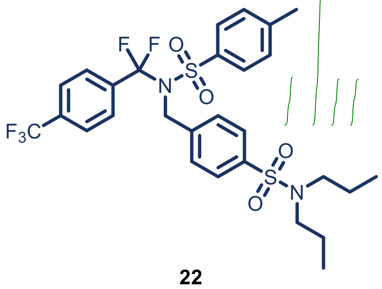

22

^{1}H NMR (300 MHz, $CDCl_3$)

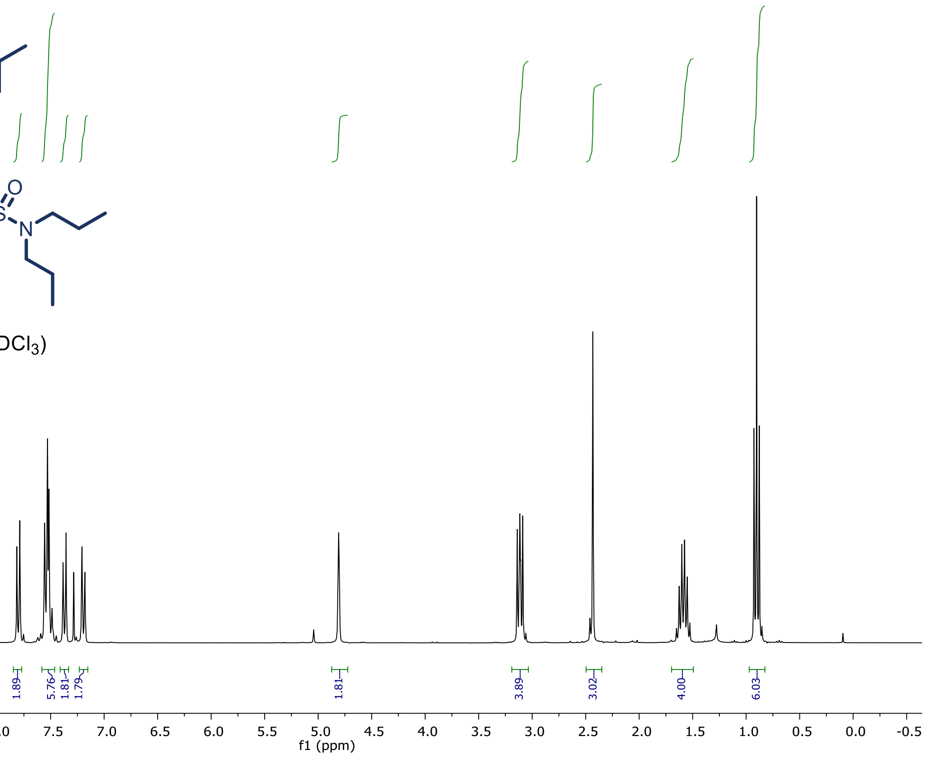

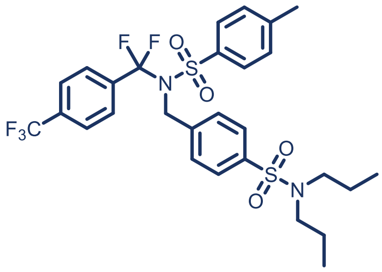

22

^{13}C NMR (75 MHz, $CDCl_3$)

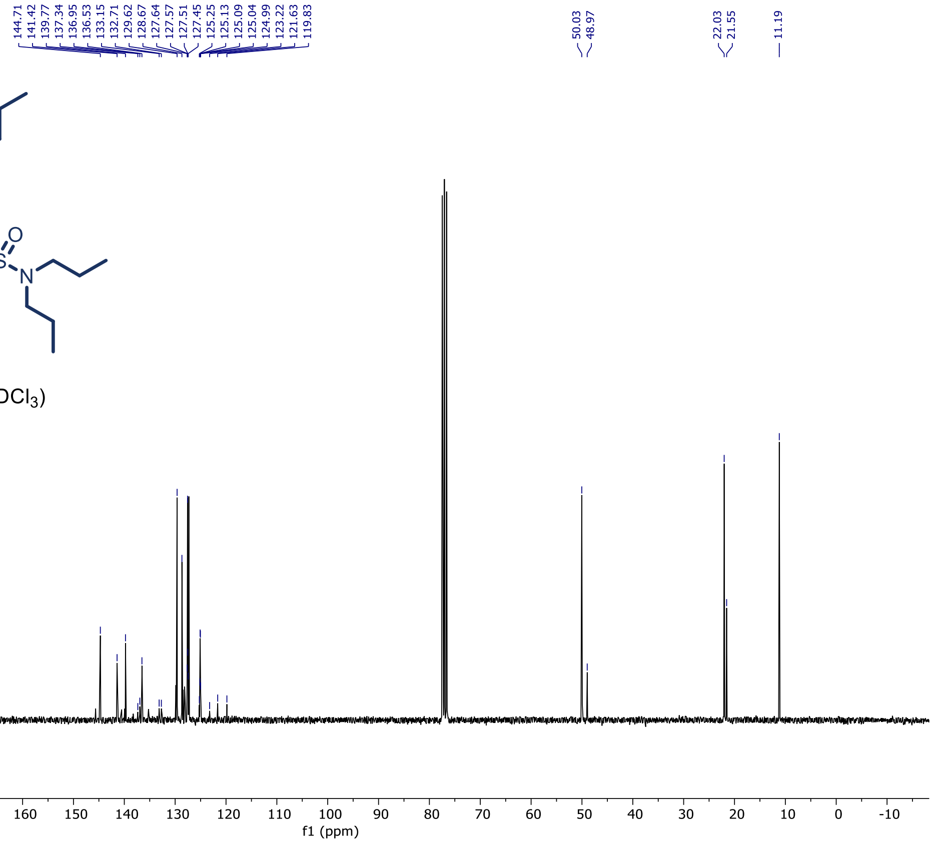

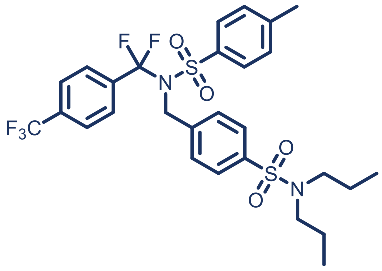

22

^{19}F NMR (282 MHz, $CDCl_3$)

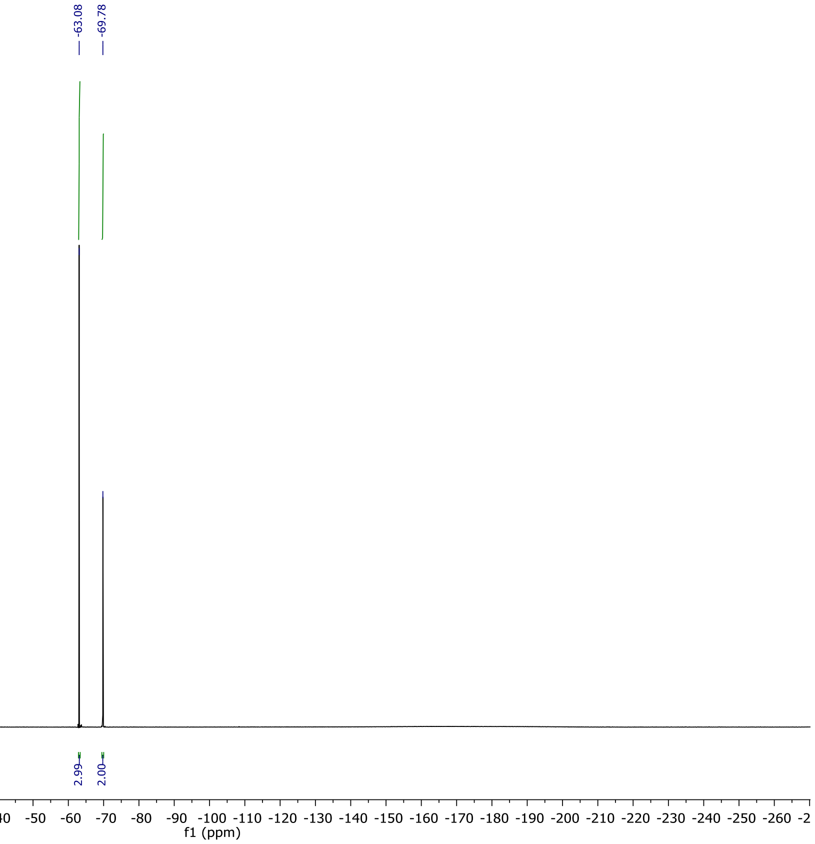

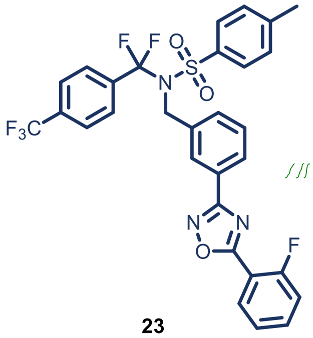

23

^{1}H NMR (300 MHz, $CDCl_3$)

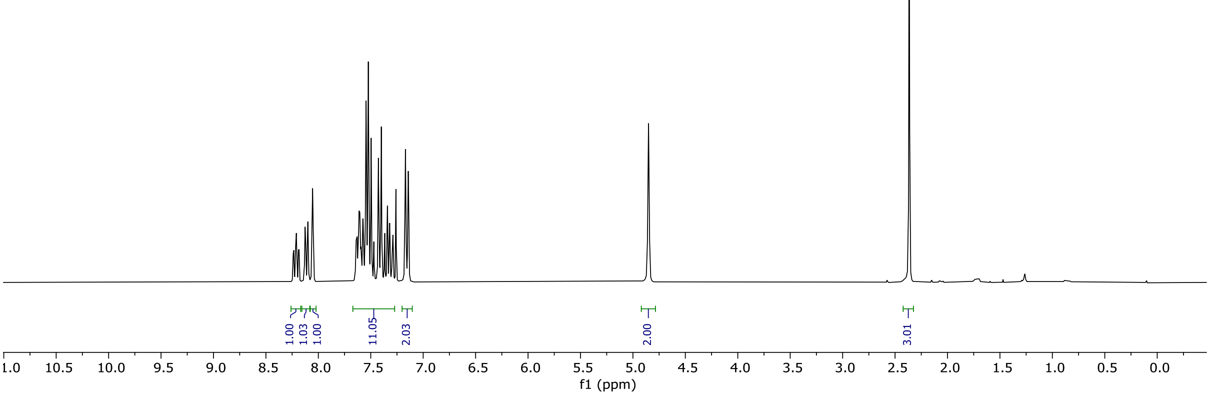

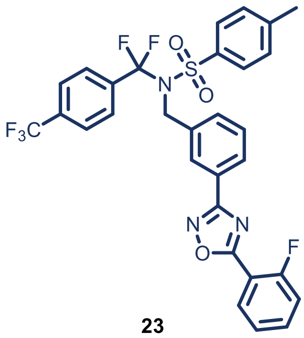

23

^{13}C NMR (75 MHz, $CDCl_3$)

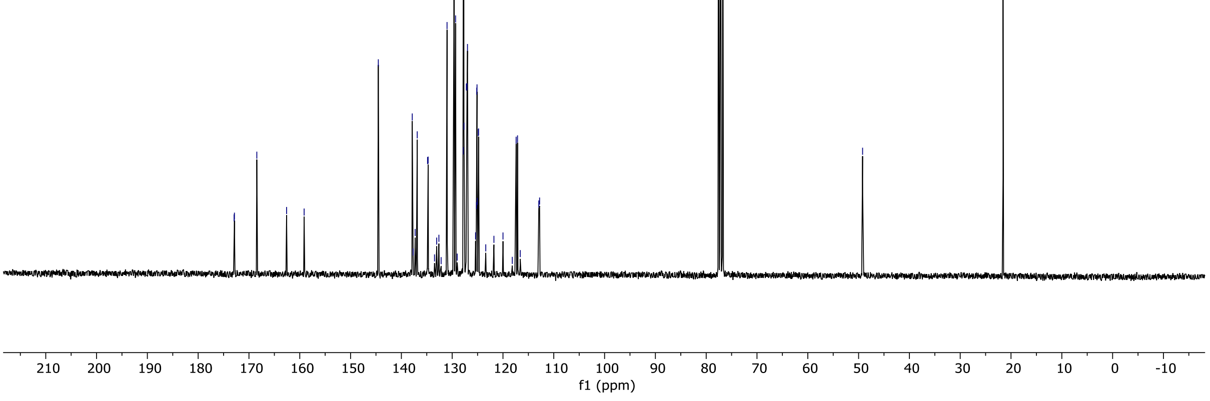

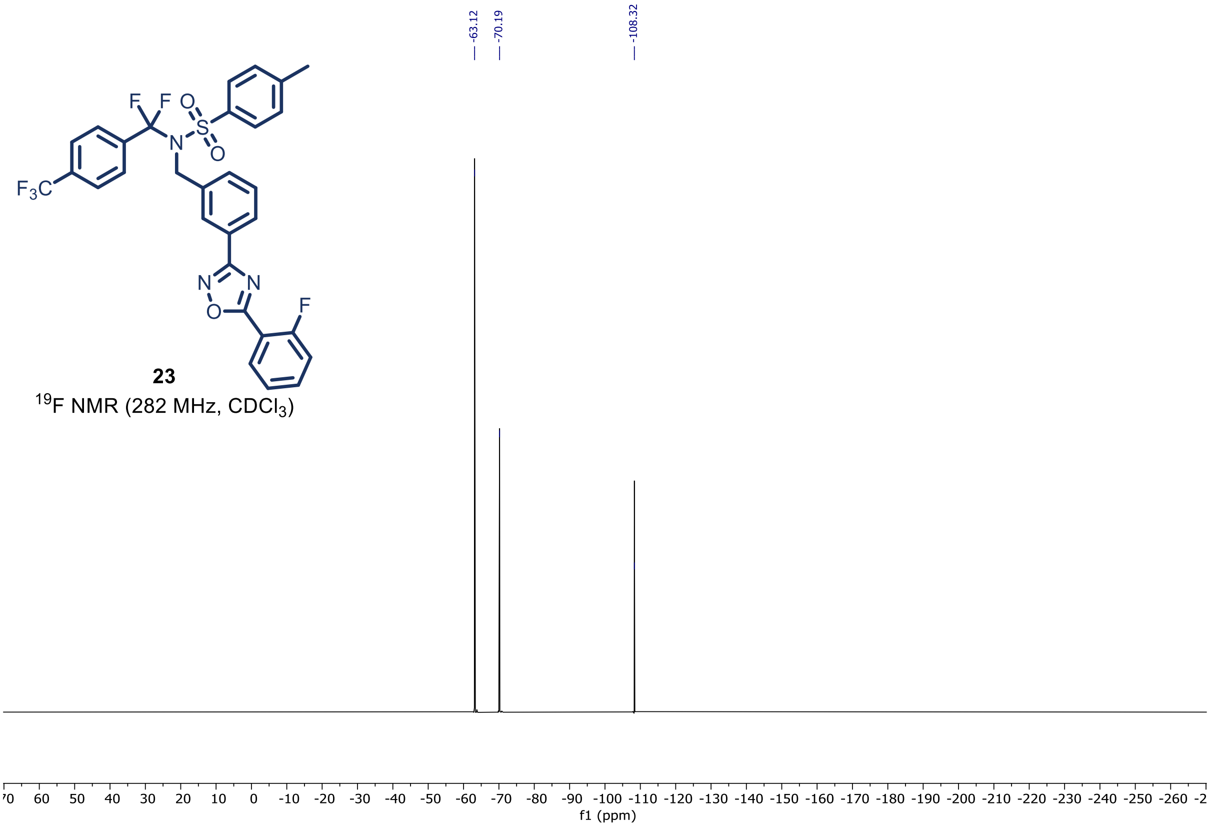

23

^{19}F NMR (282 MHz, $CDCl_3$)

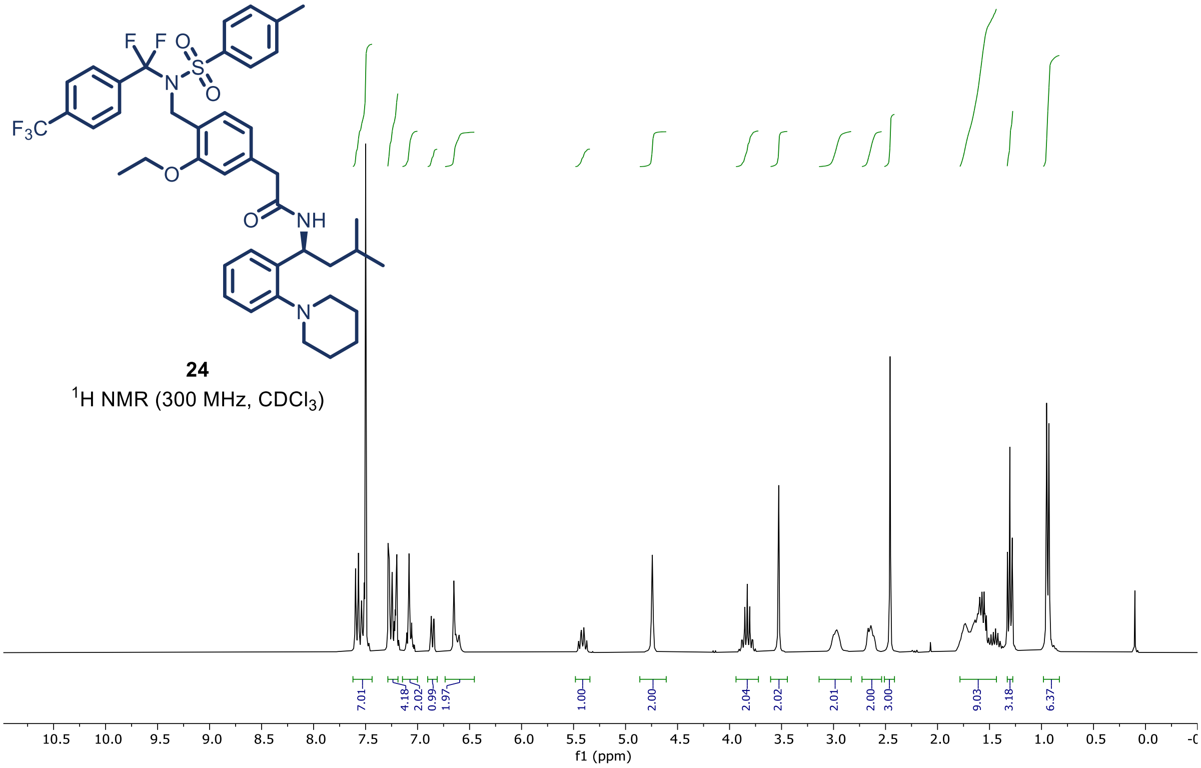

24

^{1}H NMR (300 MHz, $CDCl_3$)

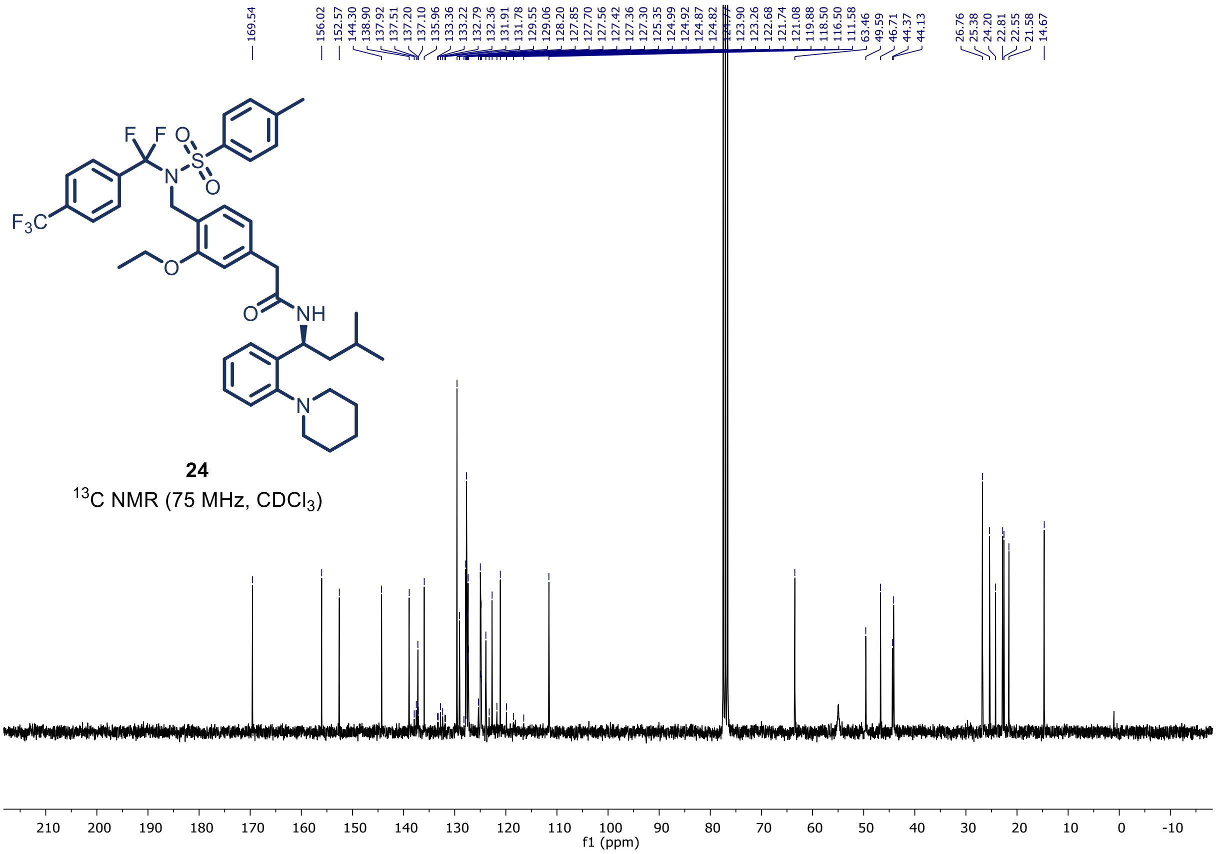

24

^{13}C NMR (75 MHz, $CDCl_3$)

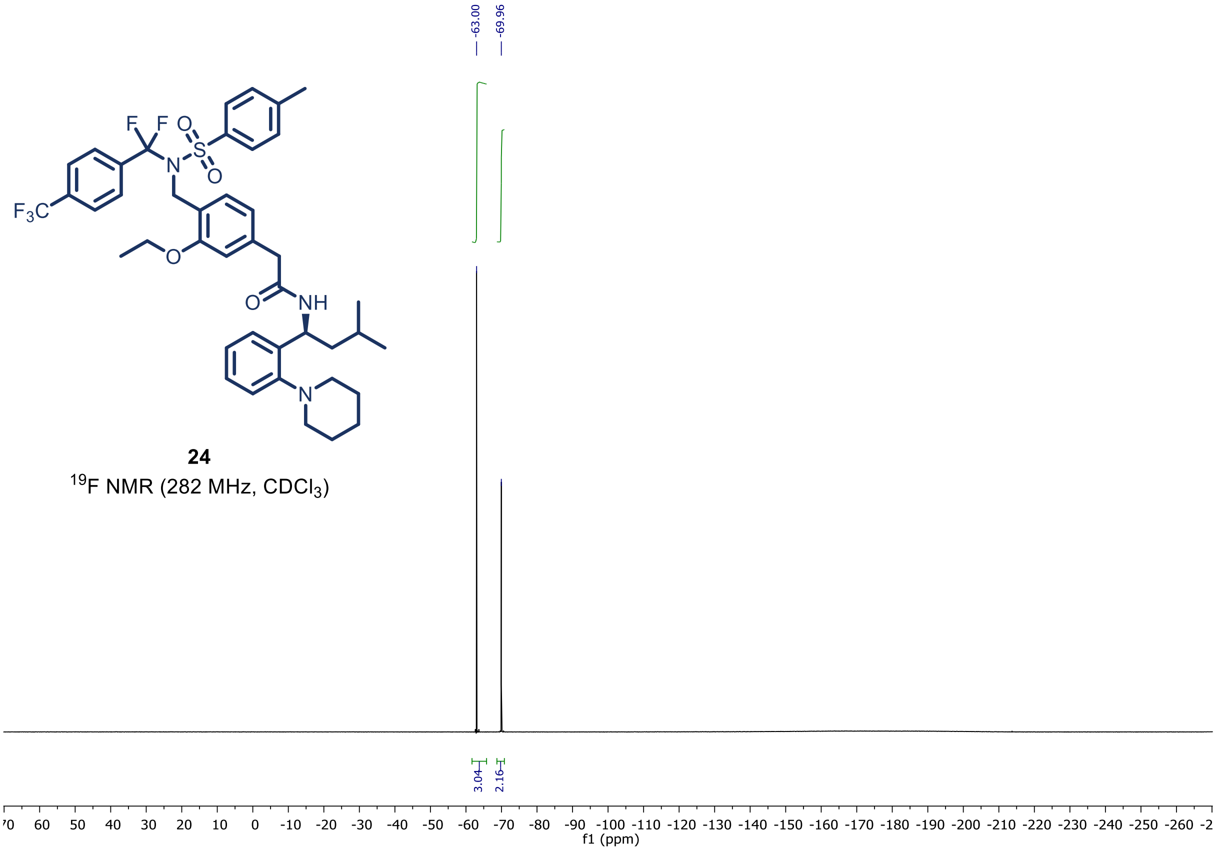

24

^{19}F NMR (282 MHz, $CDCl_3$)

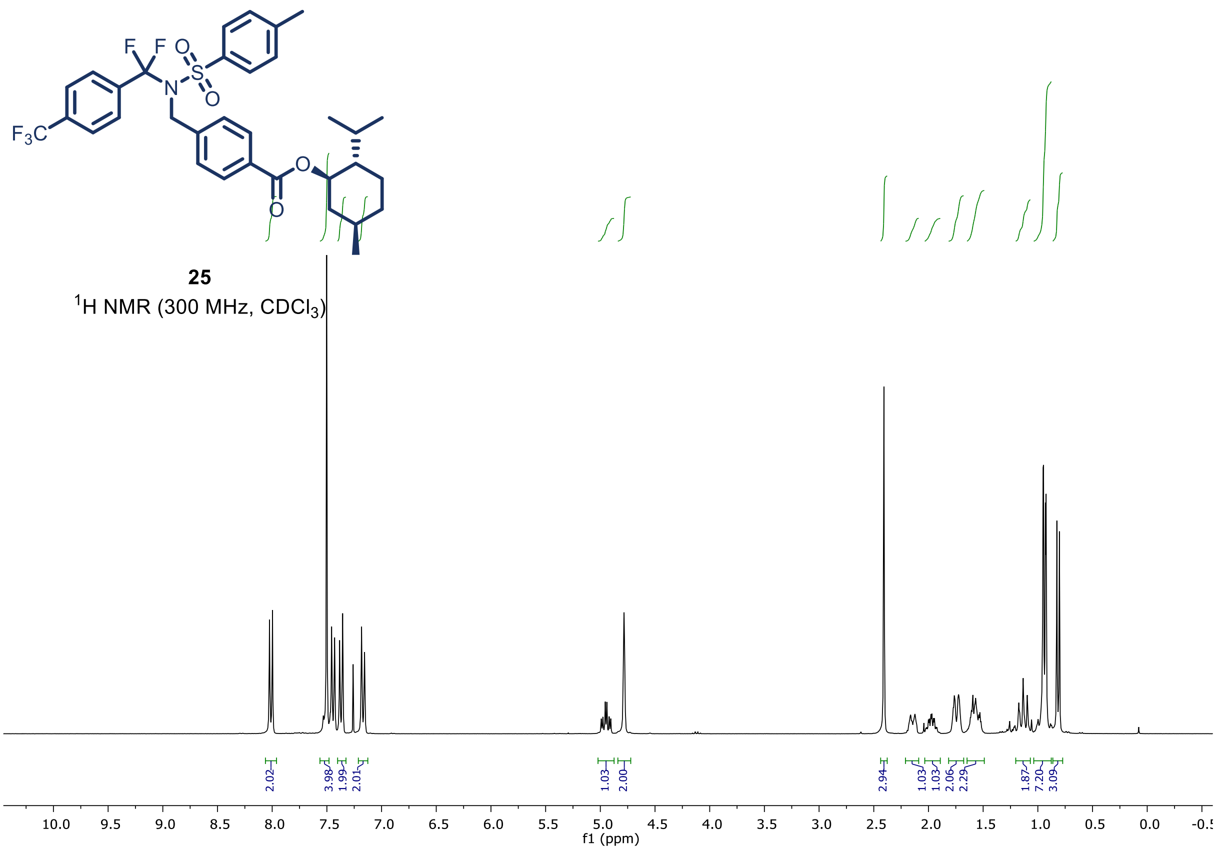
25
1H NMR (300 MHz, CDCl3)
2.02
3.98
1.99
2.01
1.03
2.00
2.94
1.03
1.03
2.06
2.29
1.87
7.20
3.09
f1 (ppm)

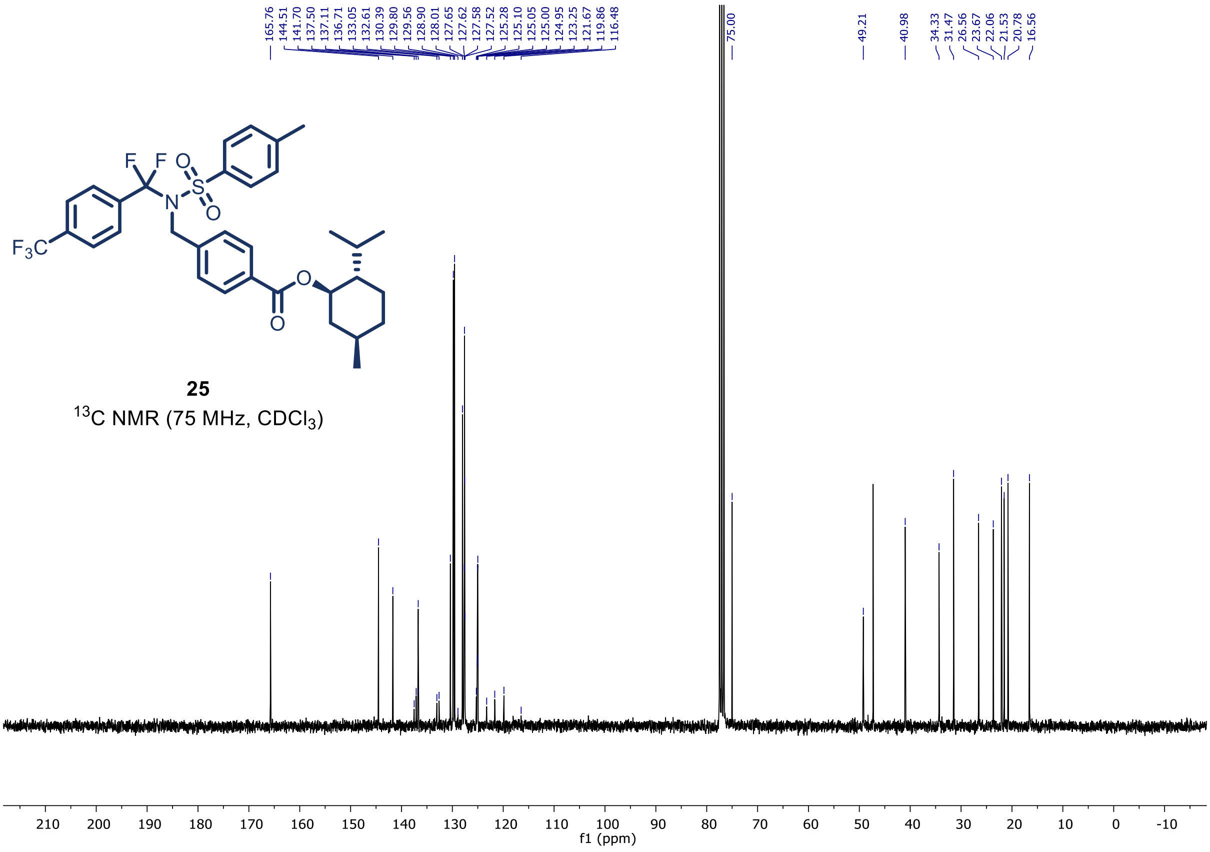
165.76
144.51
141.70
137.50
137.11
136.71
133.05
132.61
130.39
129.80
129.56
128.90
128.01
127.65
127.62
127.58
127.52
125.28
125.10
125.05
125.00
124.95
123.25
121.67
119.86
116.48
75.00
49.21
40.98
34.33
31.47
26.56
23.67
22.06
21.53
20.78
16.56
25
13C NMR (75 MHz, CDCl3)
f1 (ppm)

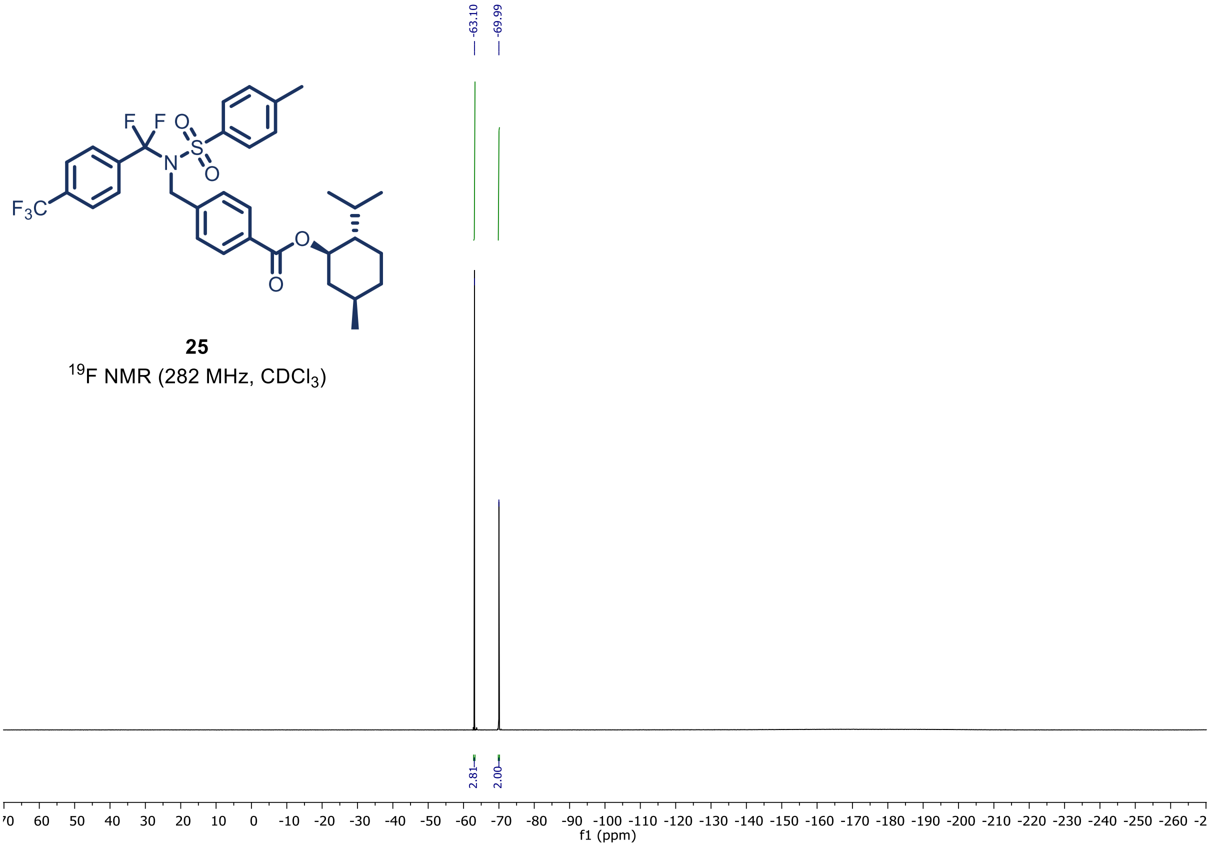
F F O
S
N
O
F3C
O
O
25
19F NMR (282 MHz, CDCl3)
-63.10
-69.99
2.81
2.00
f1 (ppm)

5.3 Scope of Aryl Groups

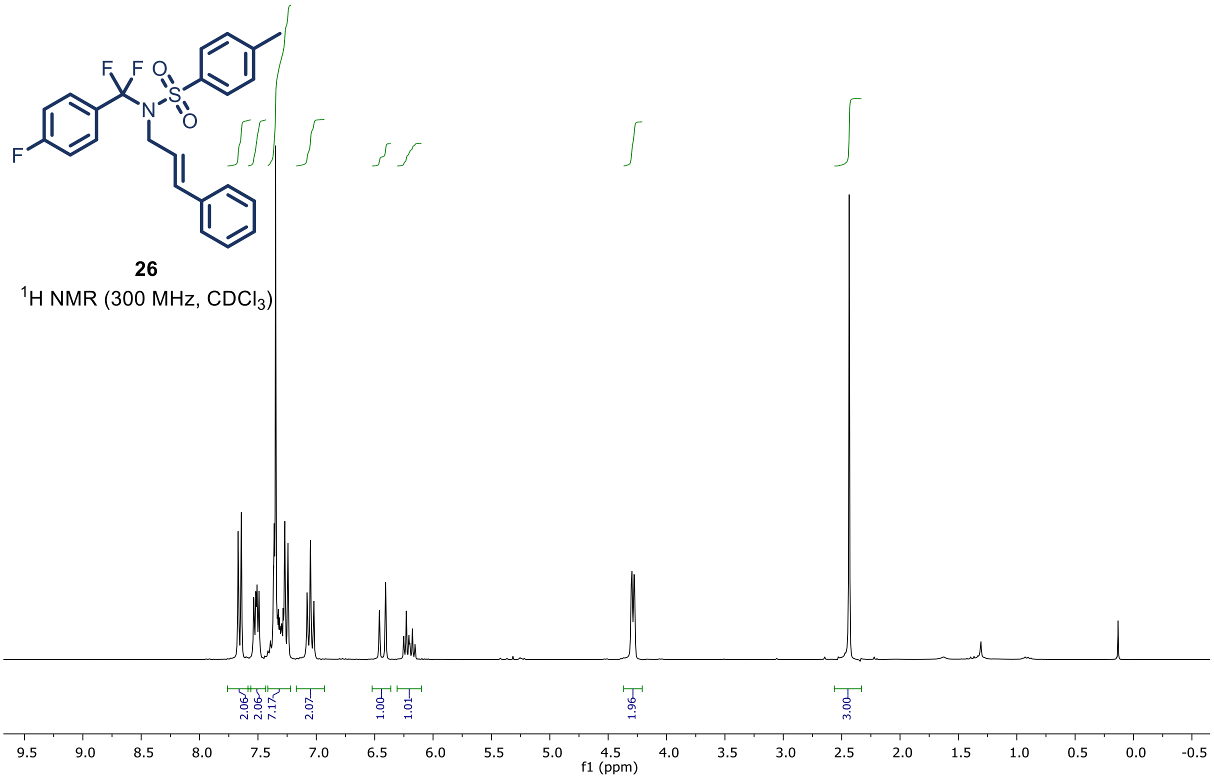

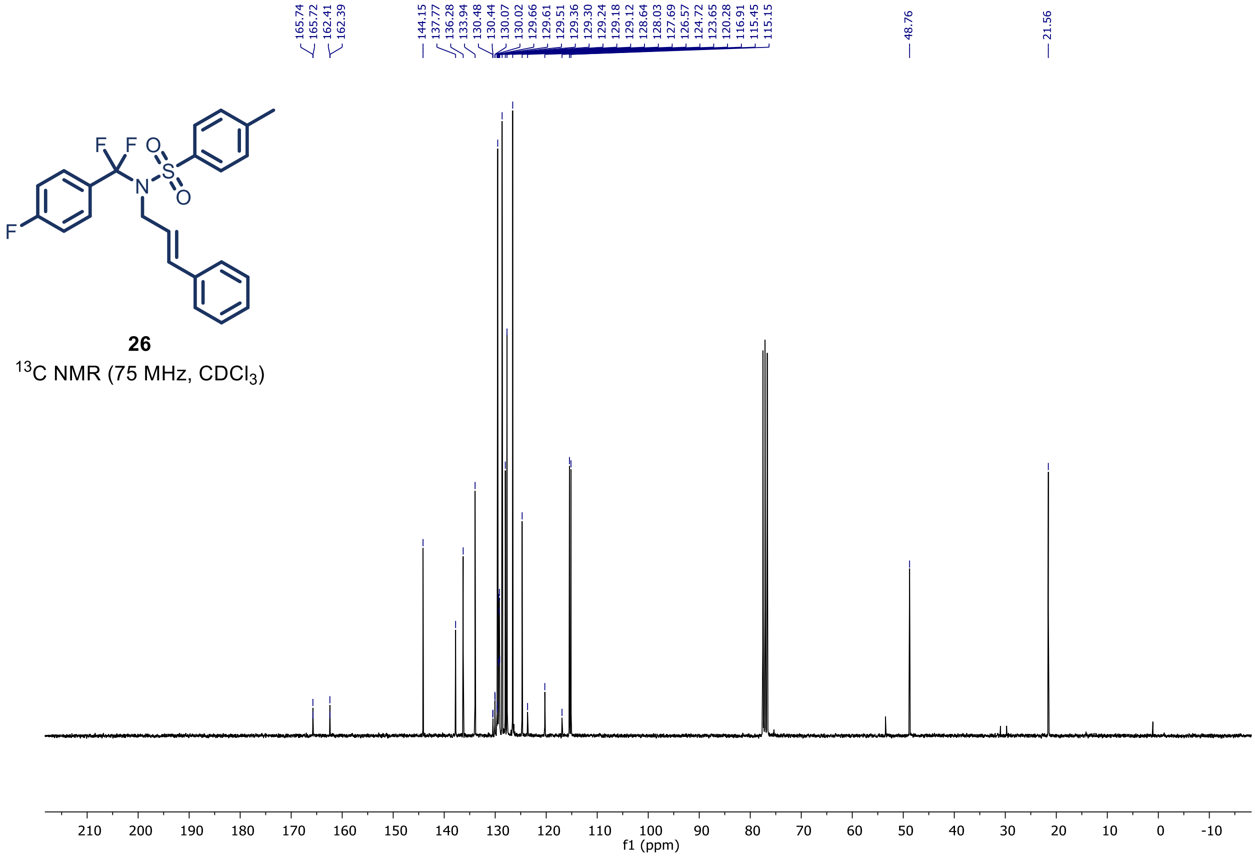

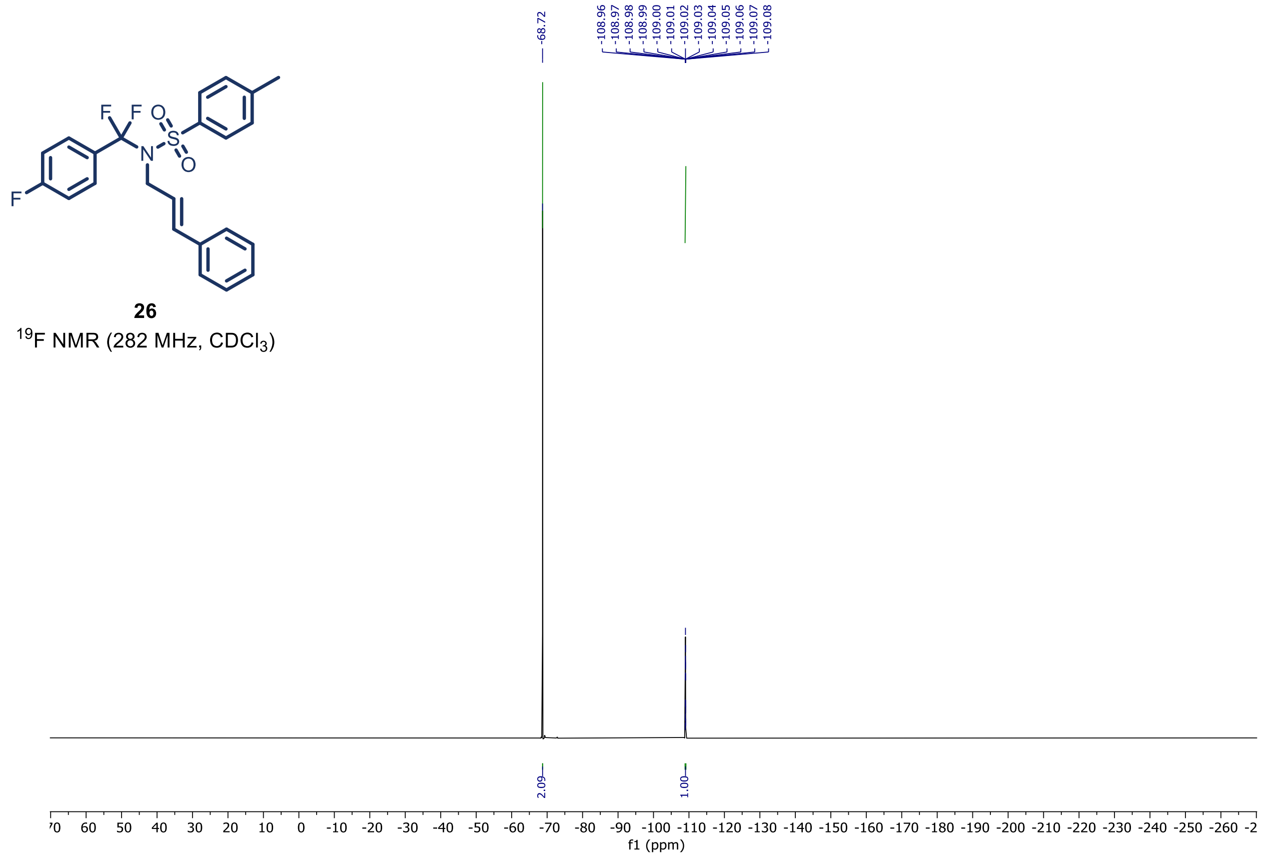

26
^{19}F NMR (282 MHz, $CDCl_3$)

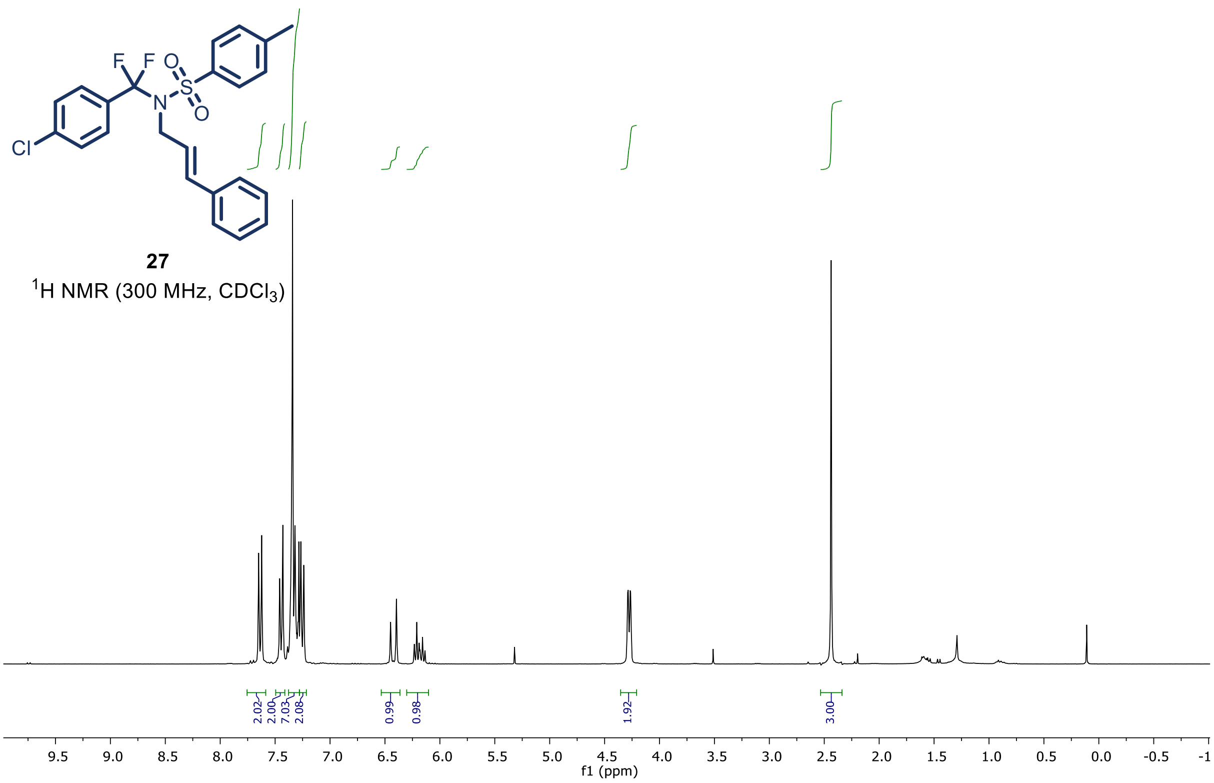

27

^{1}H NMR (300 MHz, $CDCl_3$)

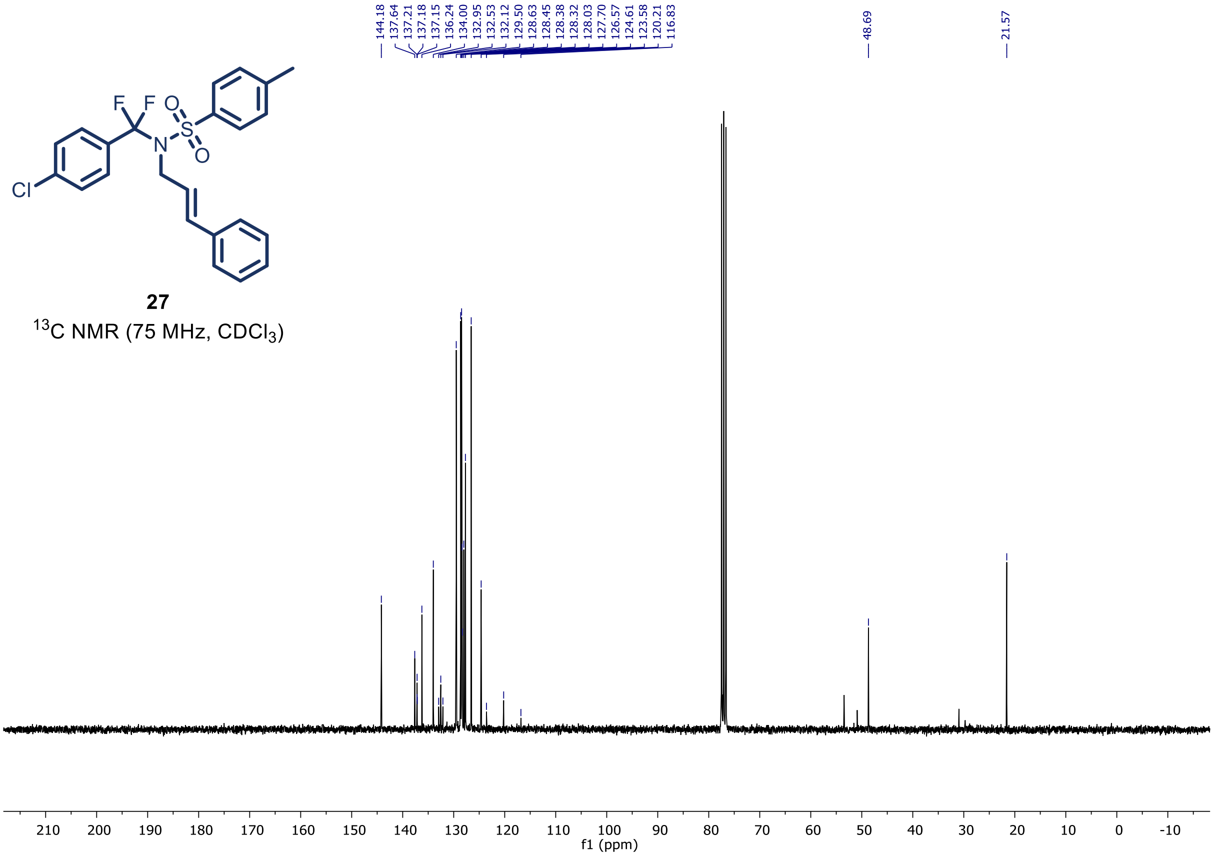

27

^{13}C NMR (75 MHz, $CDCl_3$)

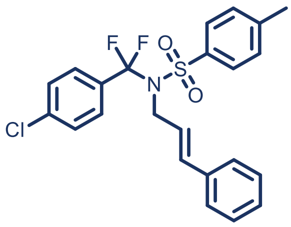

27

^{19}F NMR (282 MHz, $CDCl_3$)

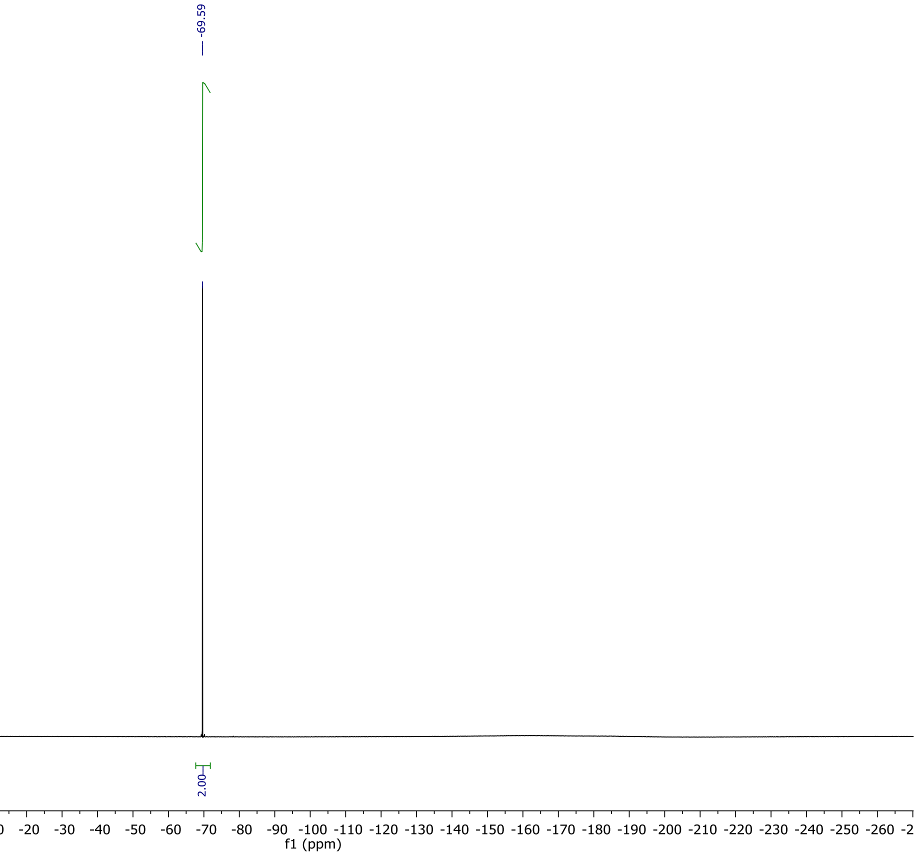

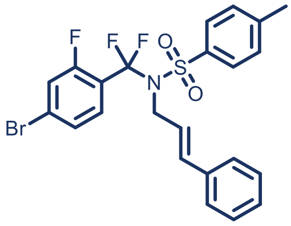

28

^{1}H NMR (300 MHz, $CDCl_3$)

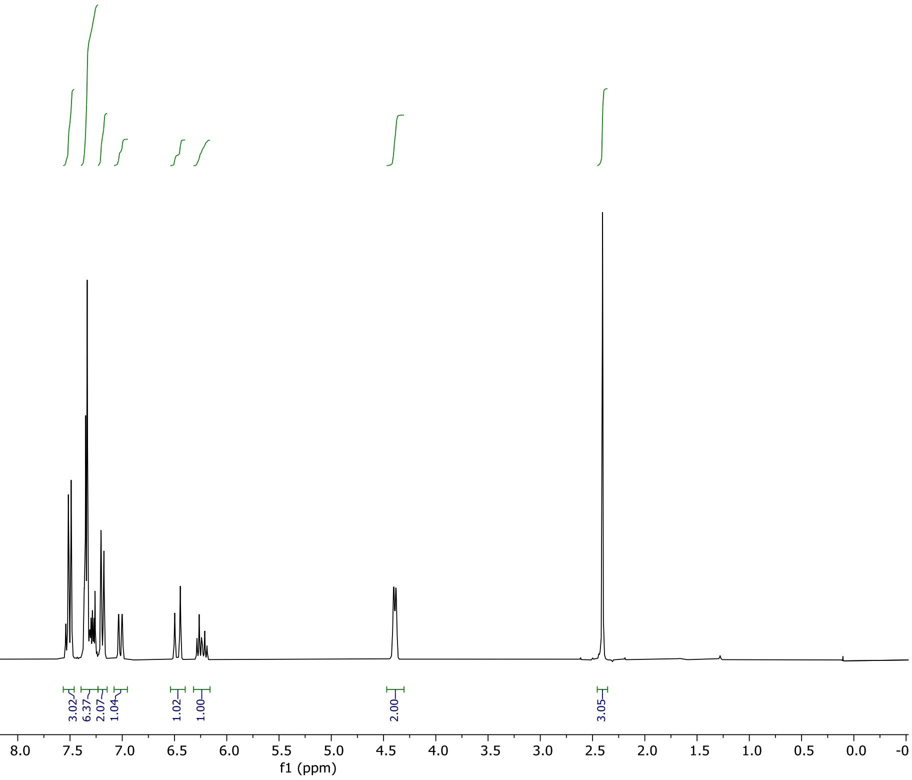

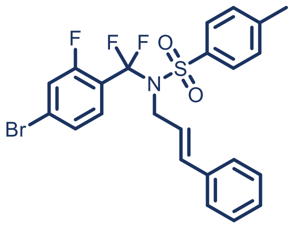

28

^{13}C NMR (75 MHz, $CDCl_3$)

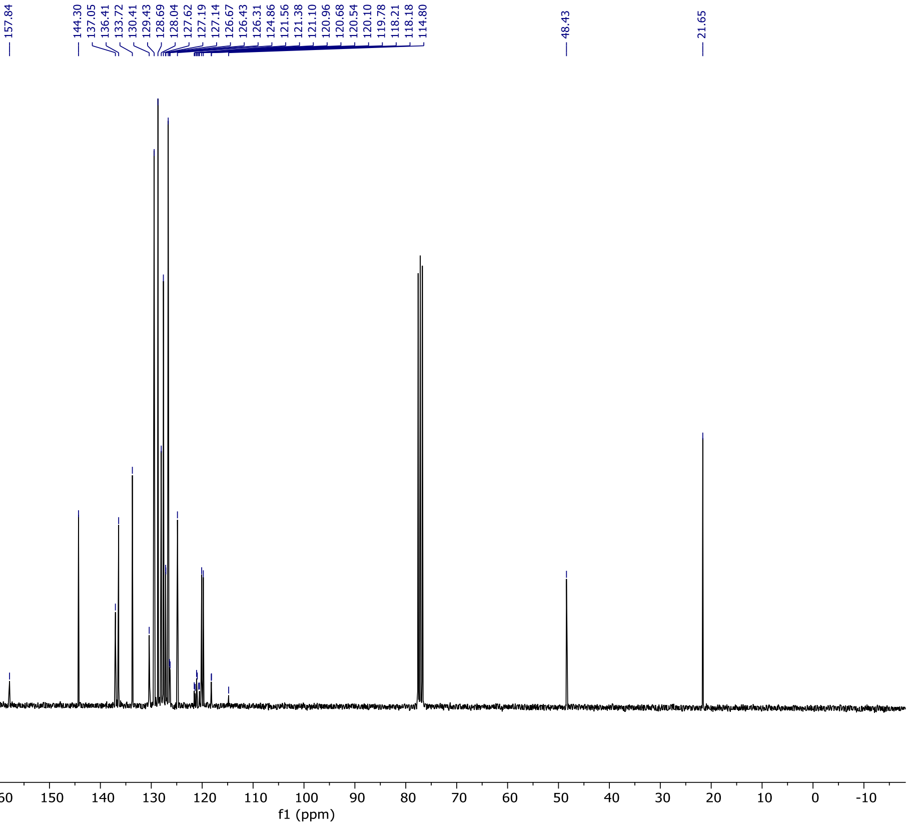

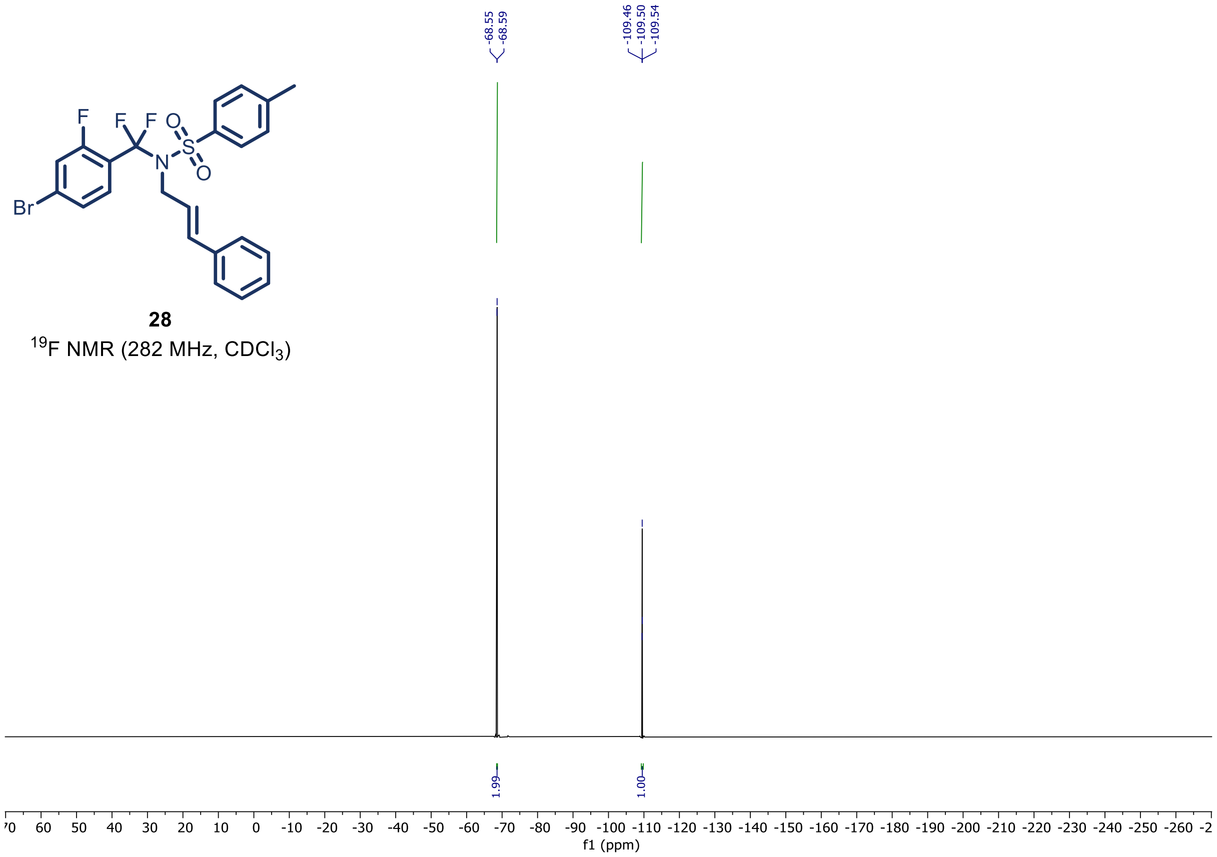

28

^{19}F NMR (282 MHz, $CDCl_3$)

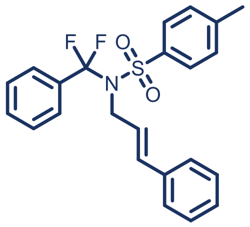

29

^{1}H NMR (300 MHz, $CDCl_3$)

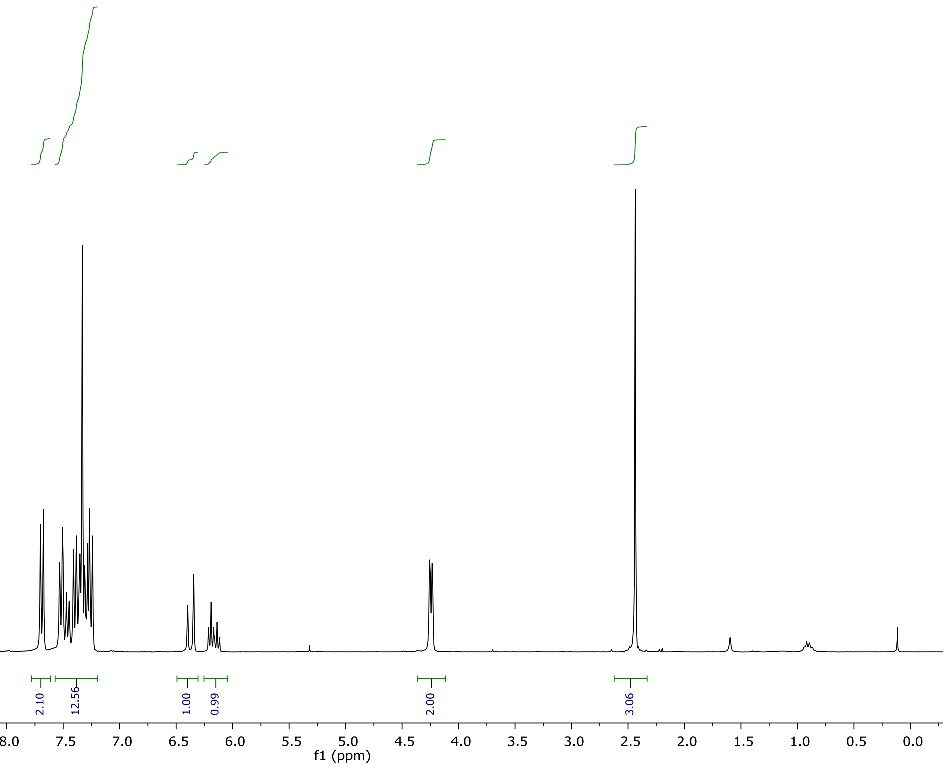

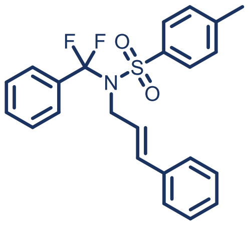

29

^{13}C NMR (75 MHz, $CDCl_3$)

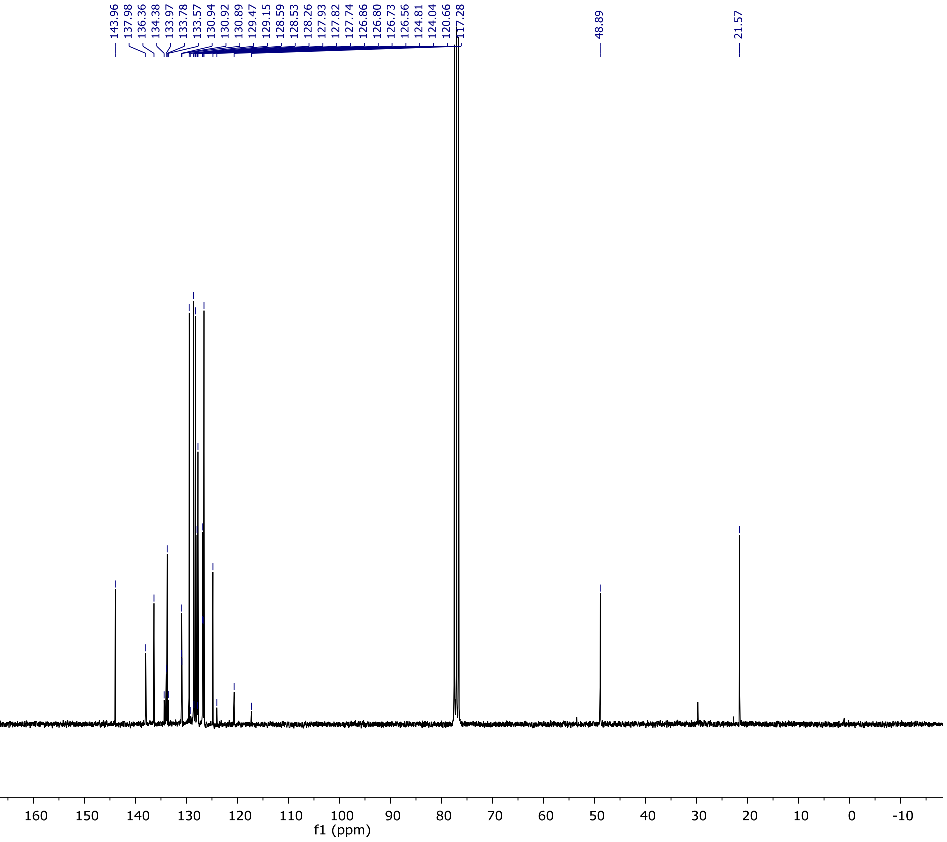

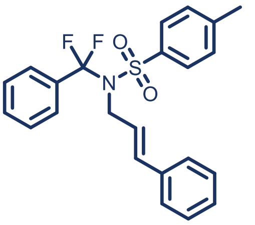

29

^{19}F NMR (282 MHz, $CDCl_3$)

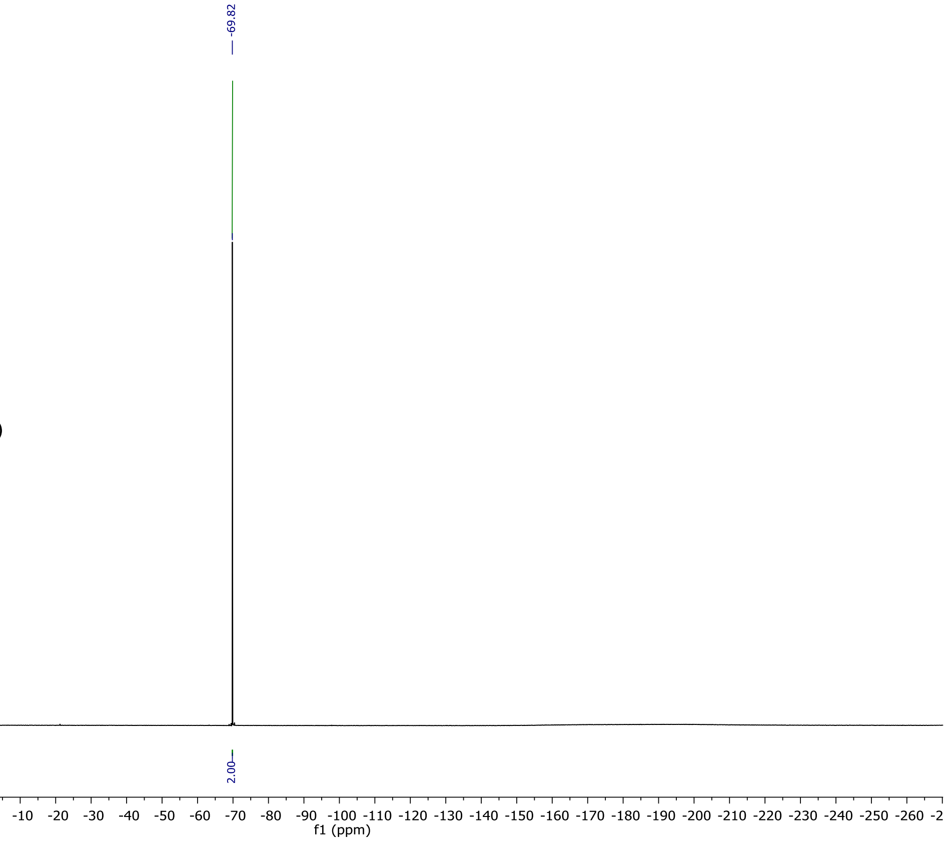

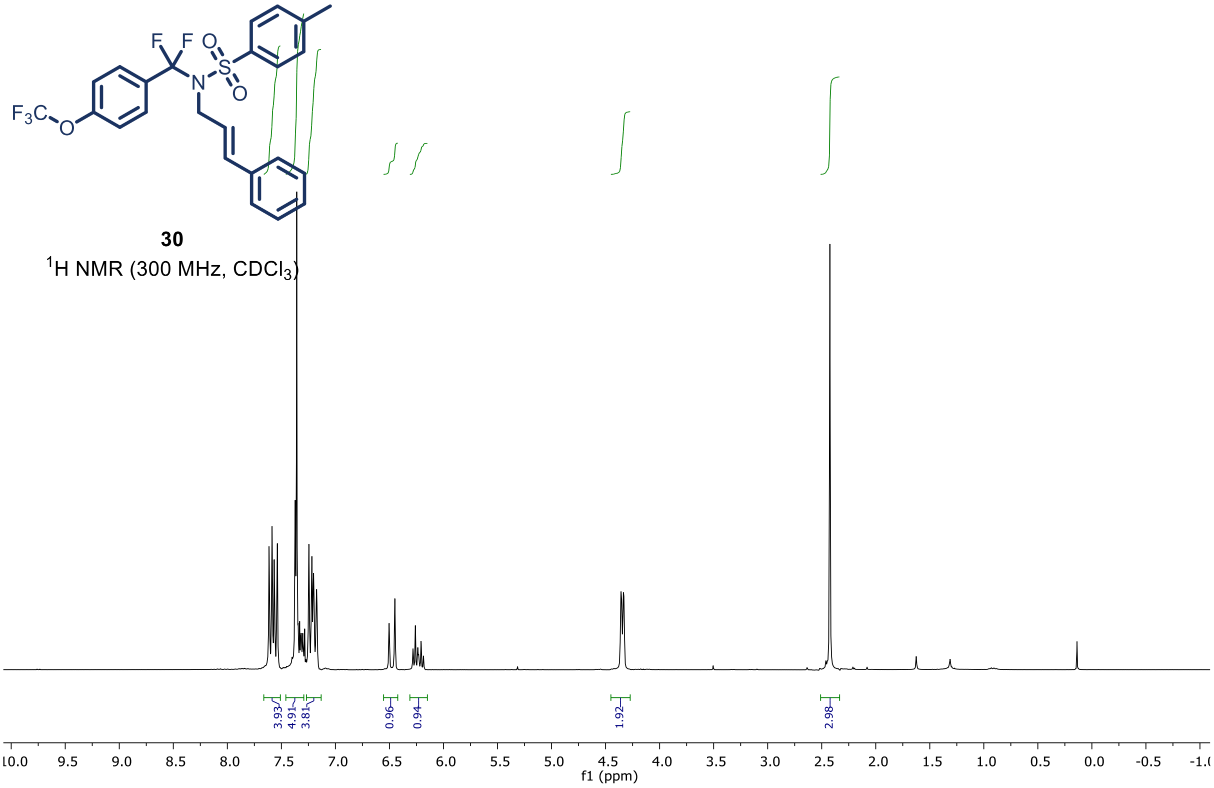

30

^{1}H NMR (300 MHz, $CDCl_3$)

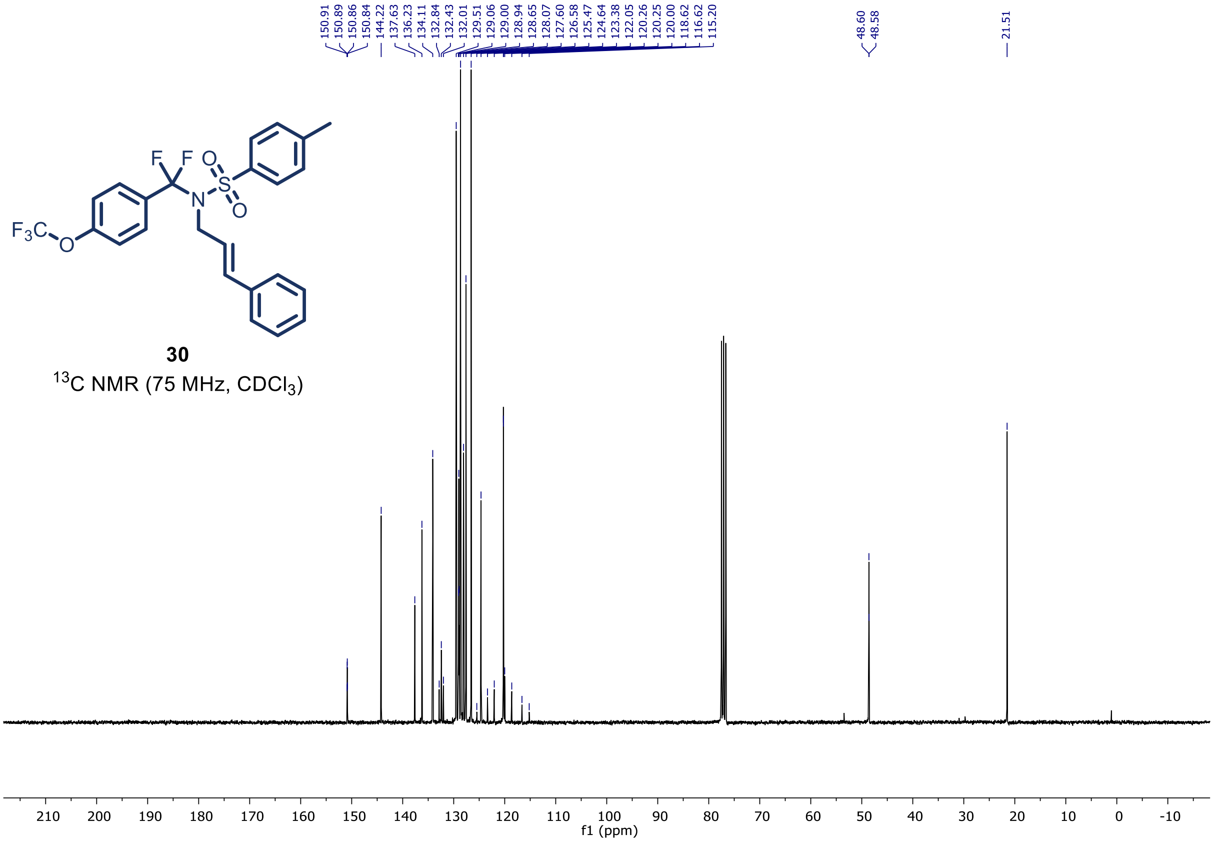

30

^{13}C NMR (75 MHz, $CDCl_3$)

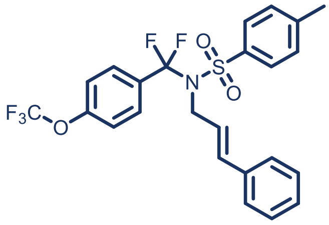

30

^{19}F NMR (282 MHz, $CDCl_3$)

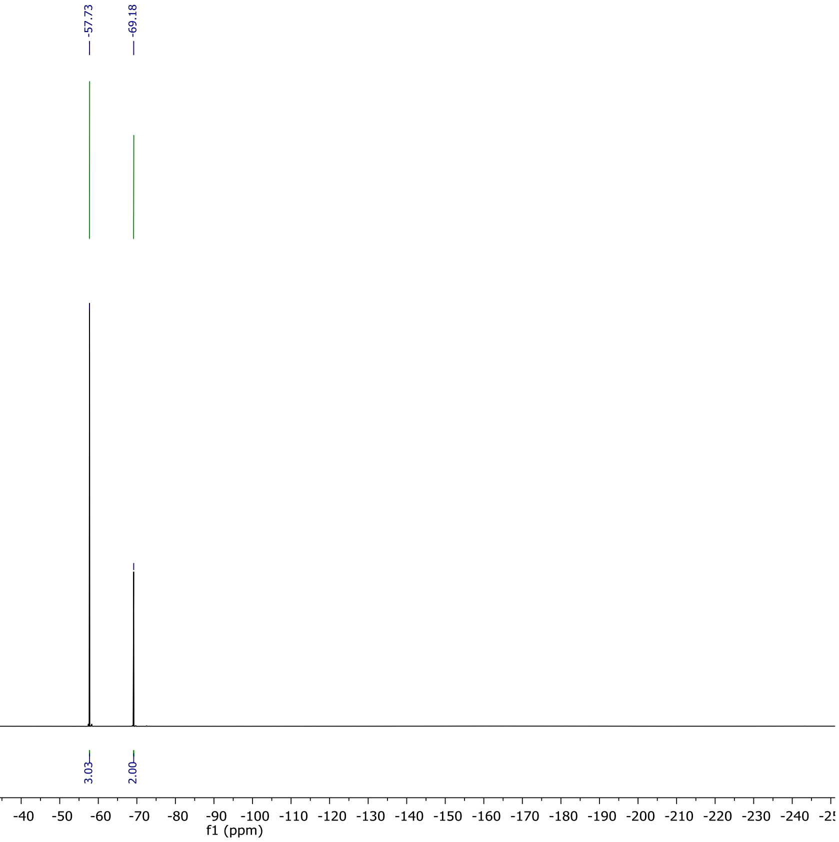

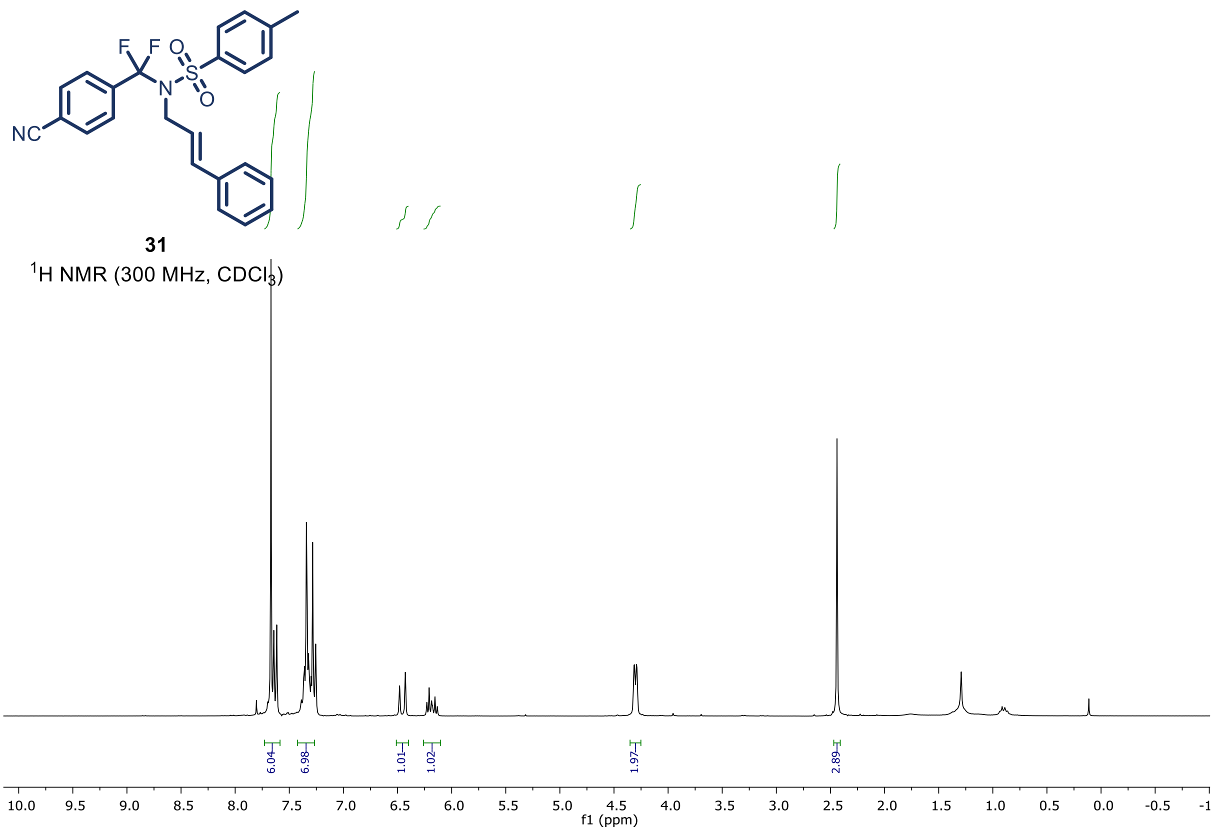
31
^{1}H NMR (300 MHz, CDCl$_3$)
6.04
6.98
1.01
1.02
1.97
2.89
f1 (ppm)

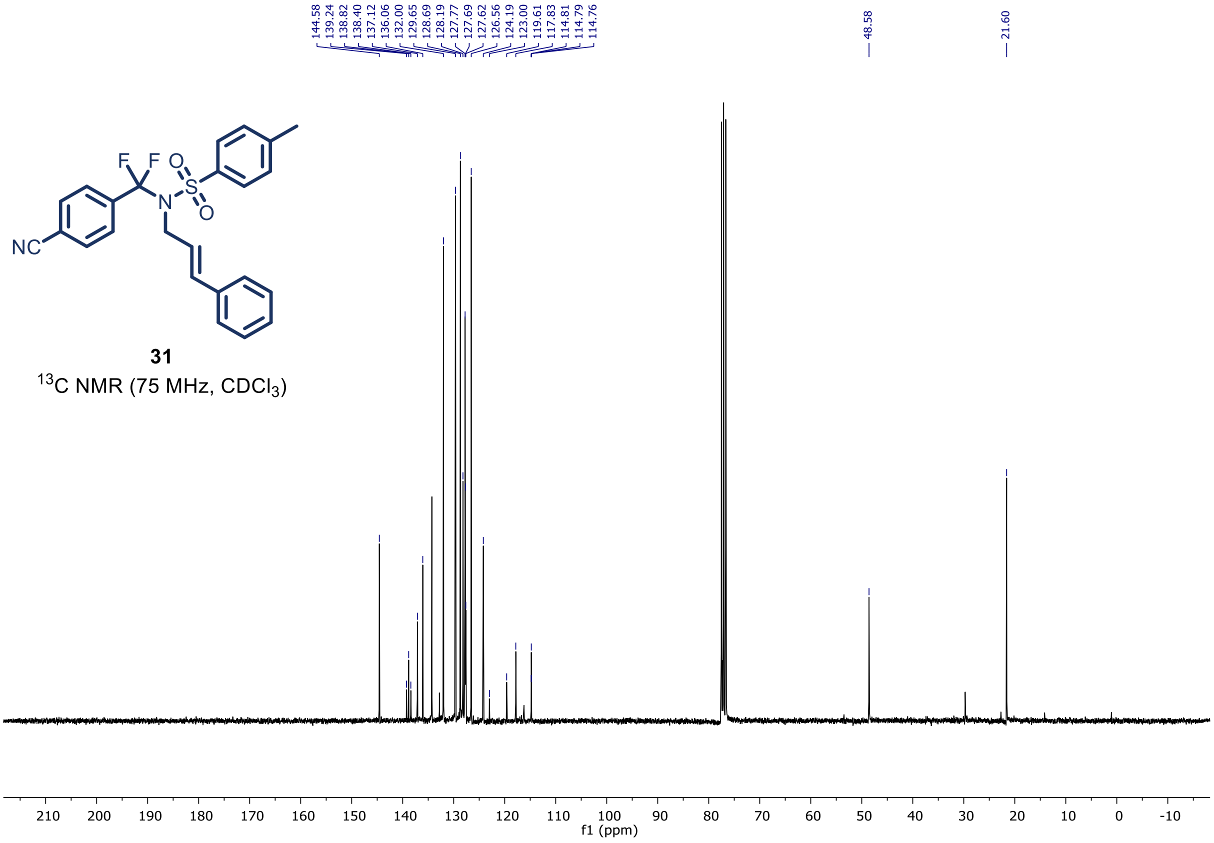
144.58
139.24
138.82
138.40
137.12
136.06
132.00
129.65
128.69
128.19
127.77
127.69
127.62
126.56
124.19
123.00
119.61
117.83
114.81
114.79
114.76
48.58
21.60
31
^{13}C NMR (75 MHz, CDCl$_3$)
f1 (ppm)

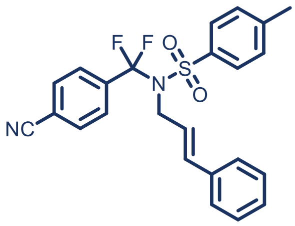

31

^{19}F NMR (282 MHz, $CDCl_3$)

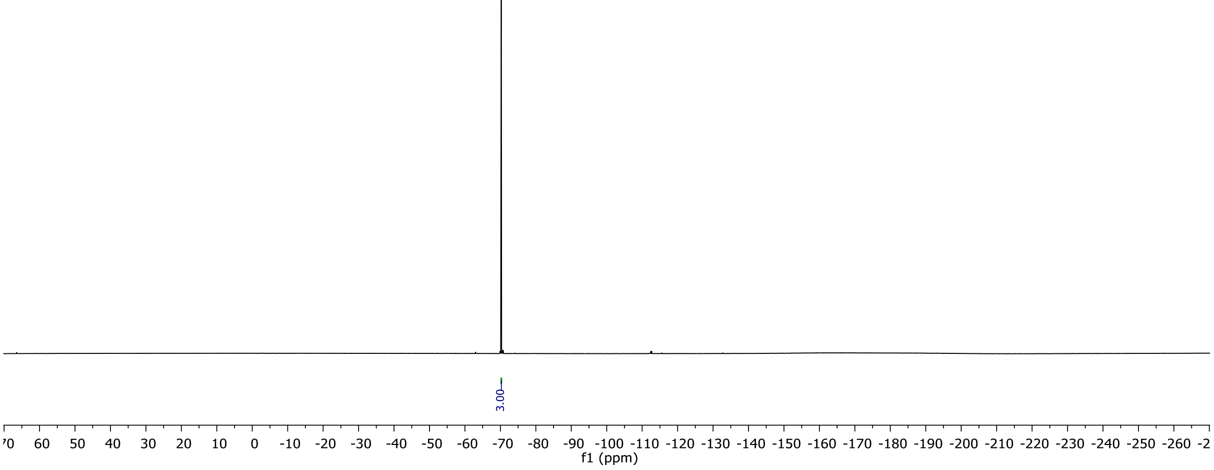

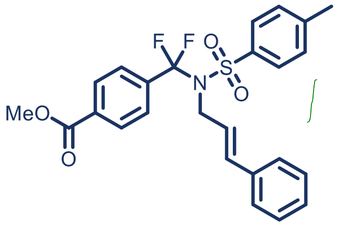

32

^{1}H NMR (300 MHz, $CDCl_3$)

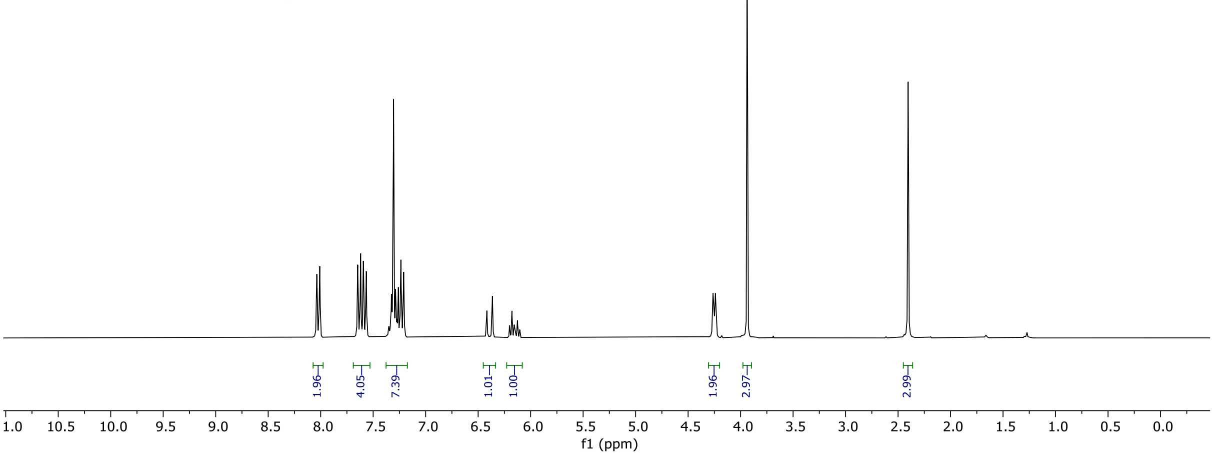

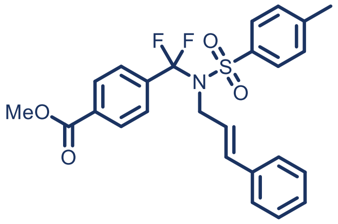

32

^{13}C NMR (75 MHz, $CDCl_3$)

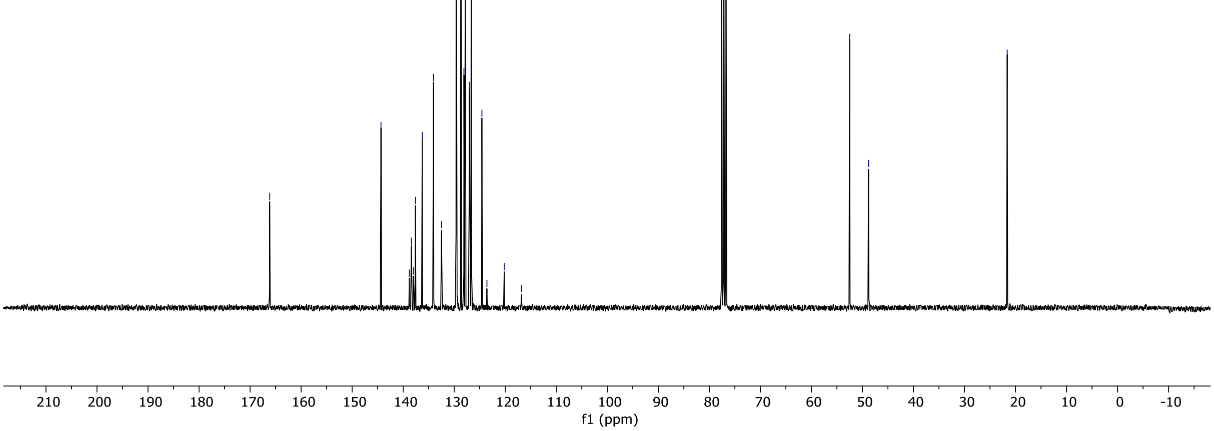

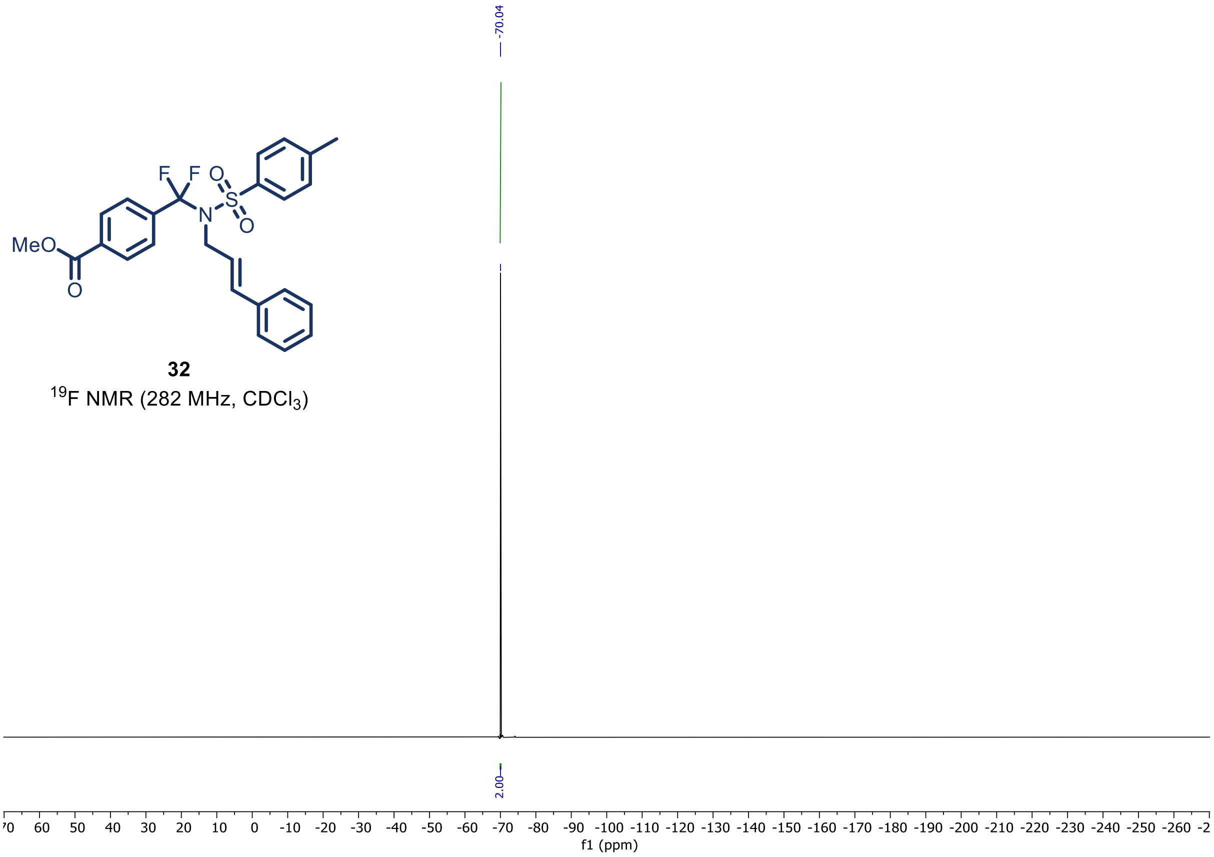

32

^{19}F NMR (282 MHz, $CDCl_3$)

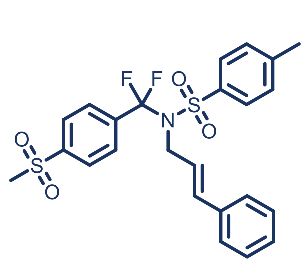

33

^{1}H NMR (300 MHz, $CDCl_3$)

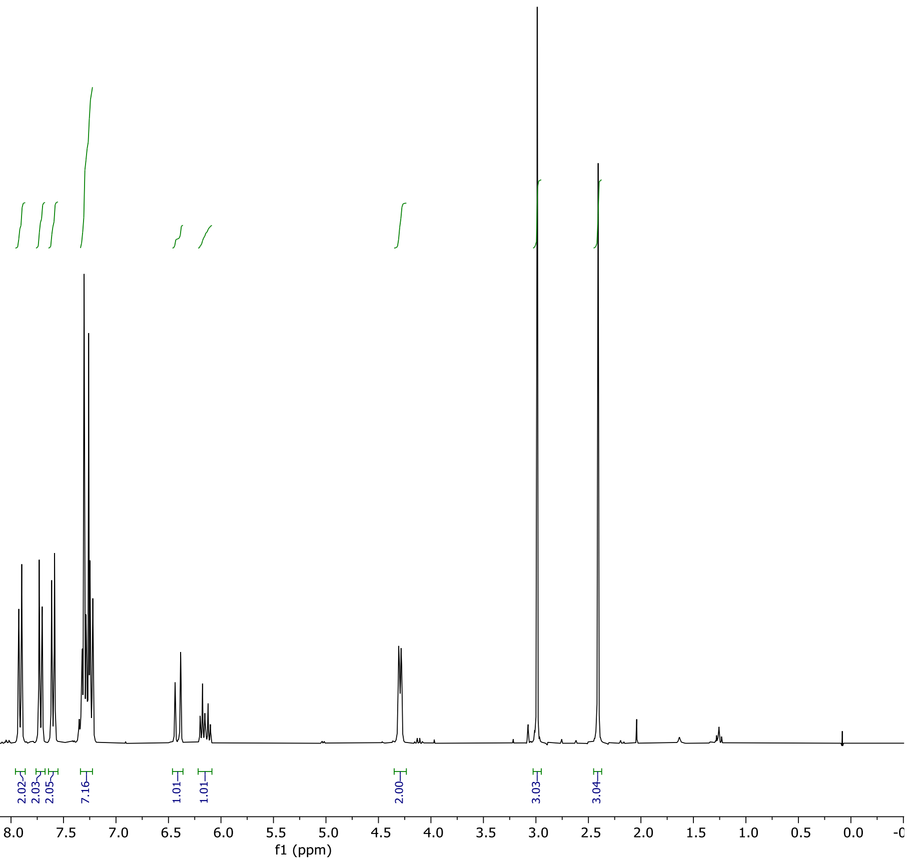

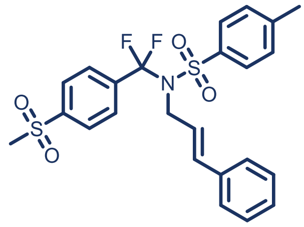

33

^{13}C NMR (75 MHz, $CDCl_3$)

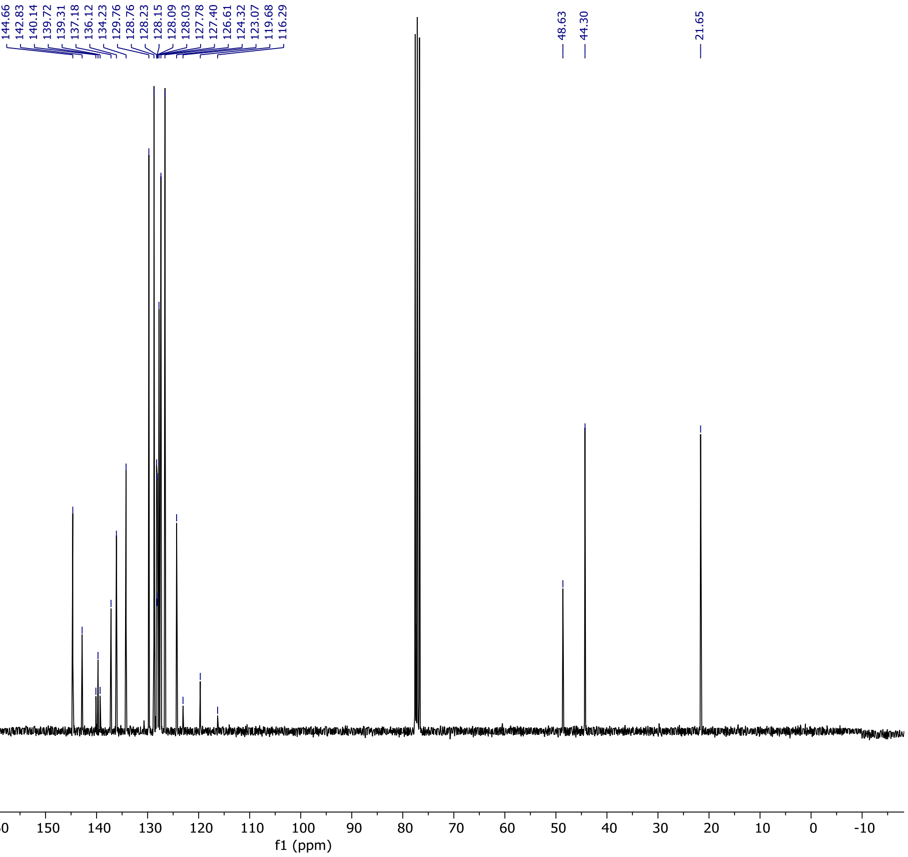

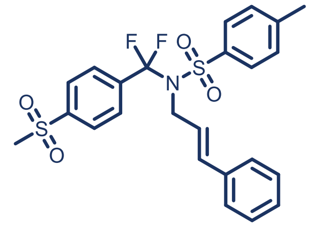

33

^{19}F NMR (282 MHz, $CDCl_3$)

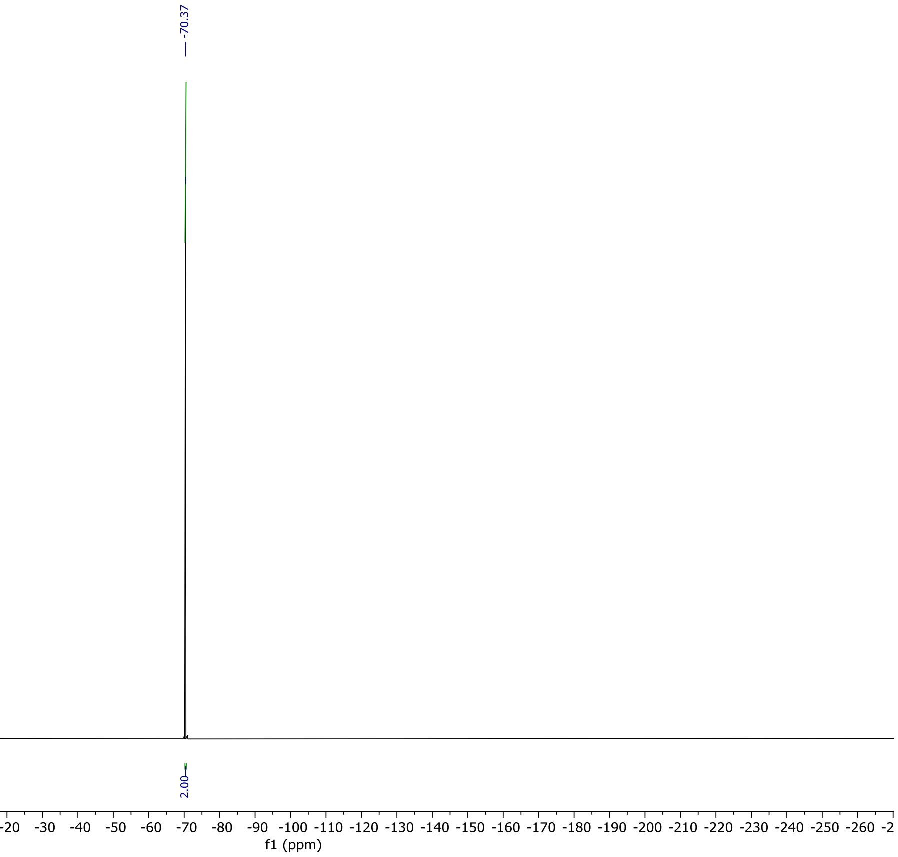

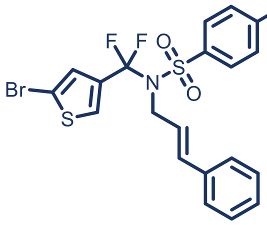

34

^{1}H NMR (300 MHz, $CDCl_3$)

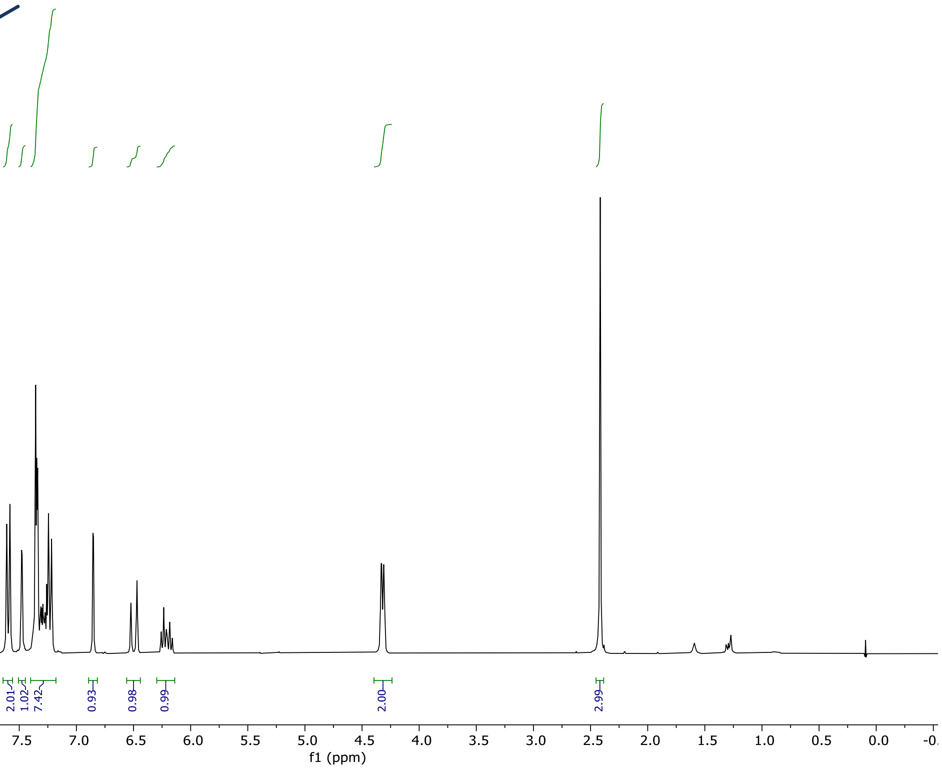

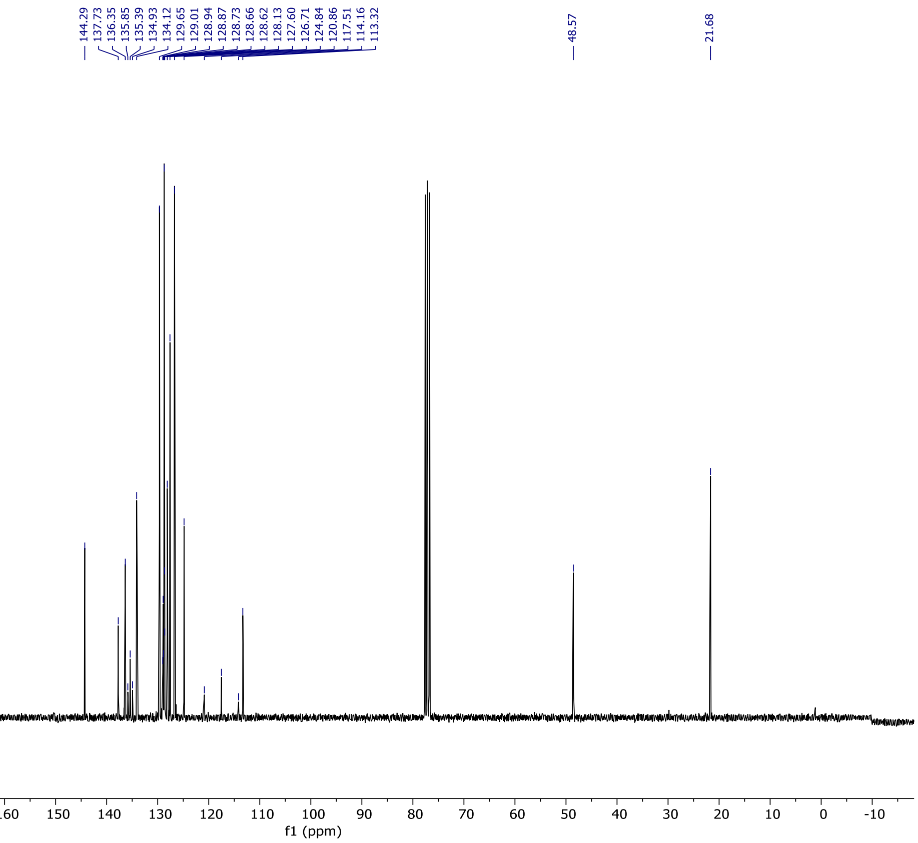

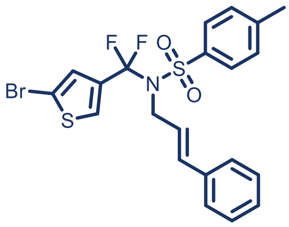

34

^{13}C NMR (75 MHz, $CDCl_3$)

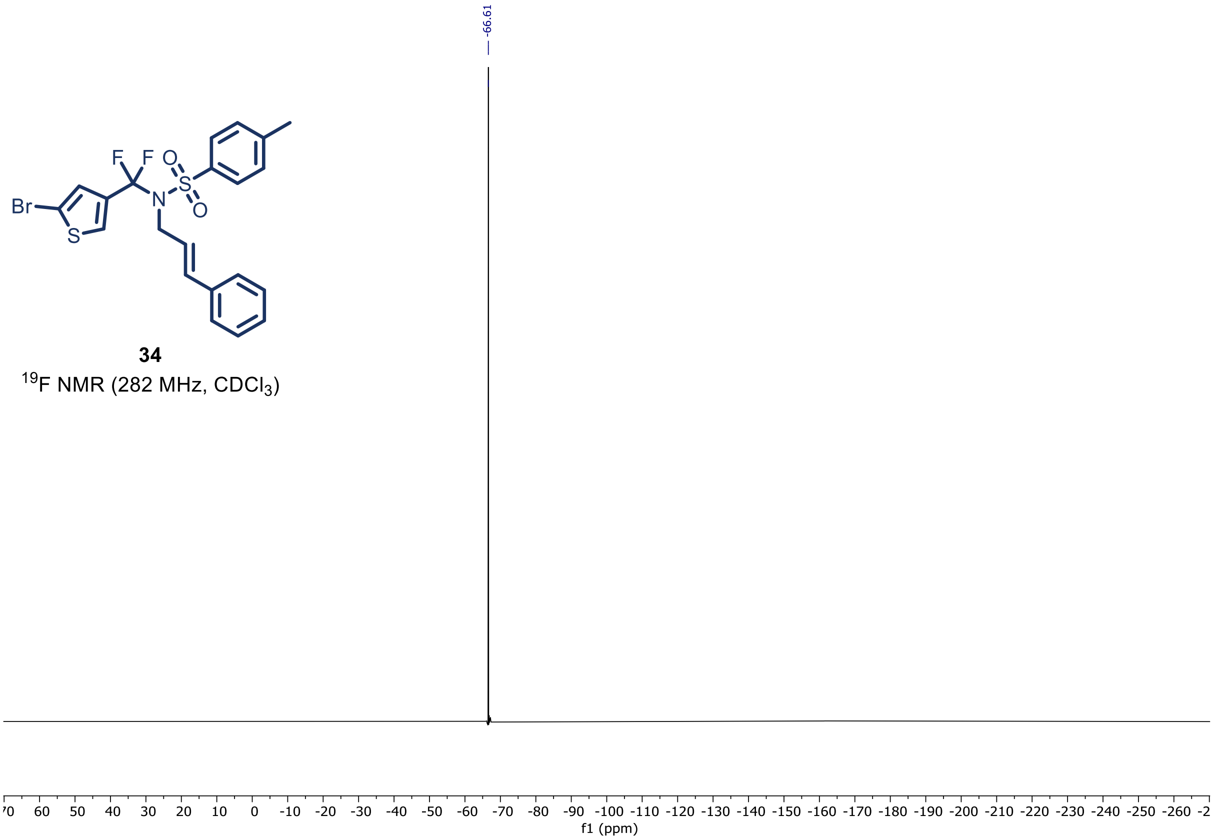

34

^{19}F NMR (282 MHz, $CDCl_3$)

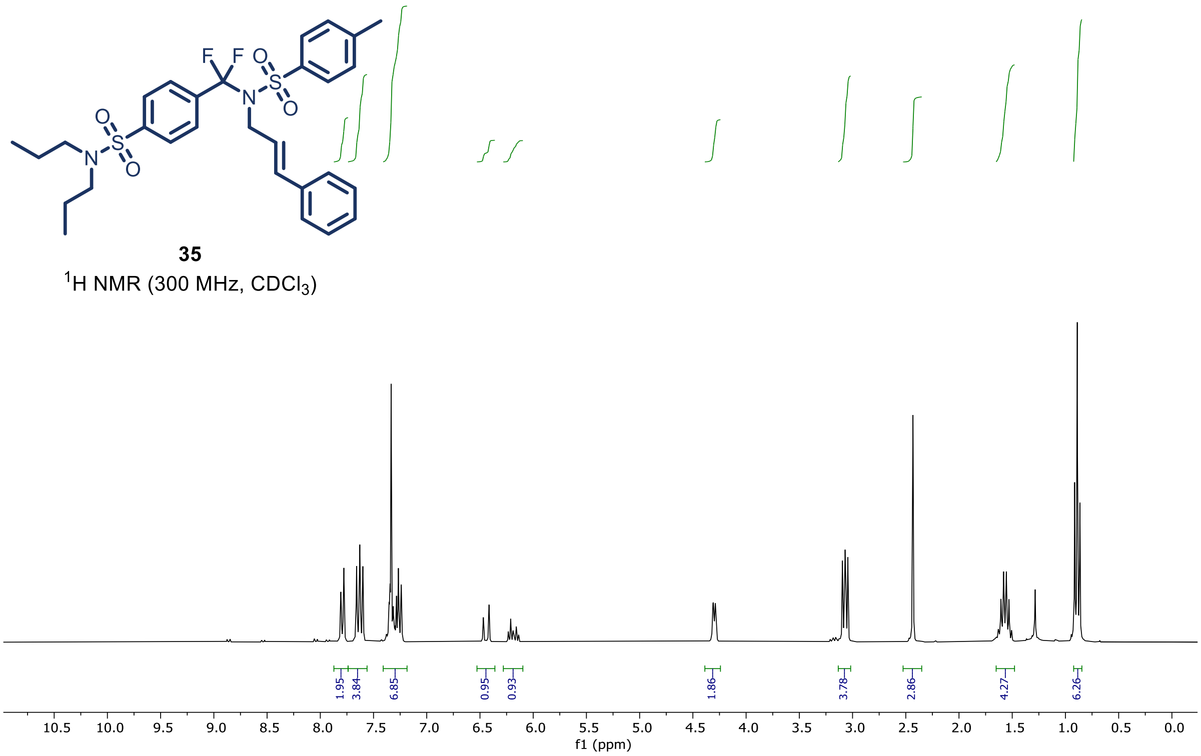

35

^{1}H NMR (300 MHz, $CDCl_3$)

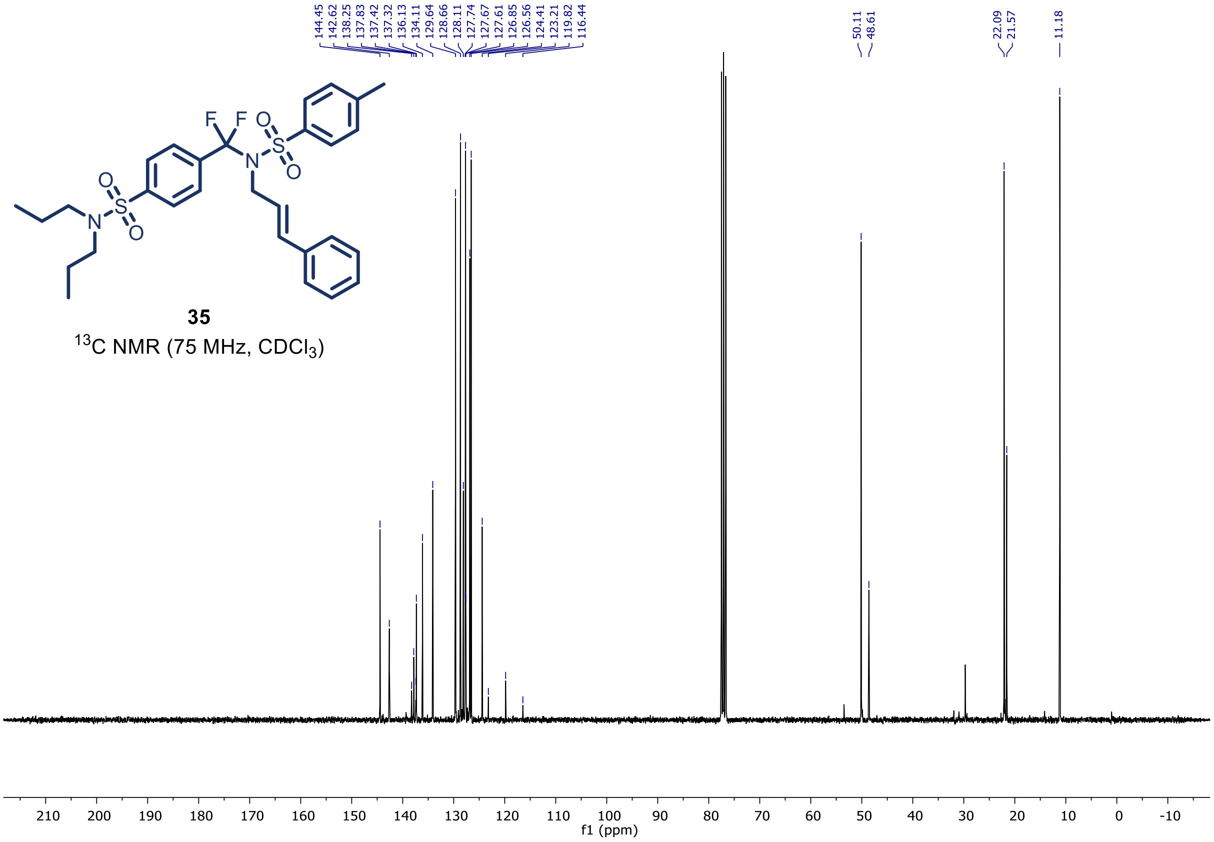

35

^{13}C NMR (75 MHz, $CDCl_3$)

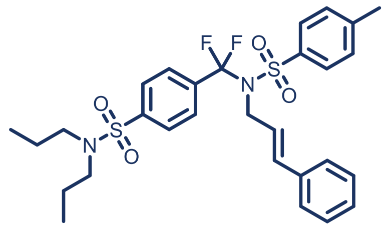

35

^{19}F NMR (282 MHz, $CDCl_3$)

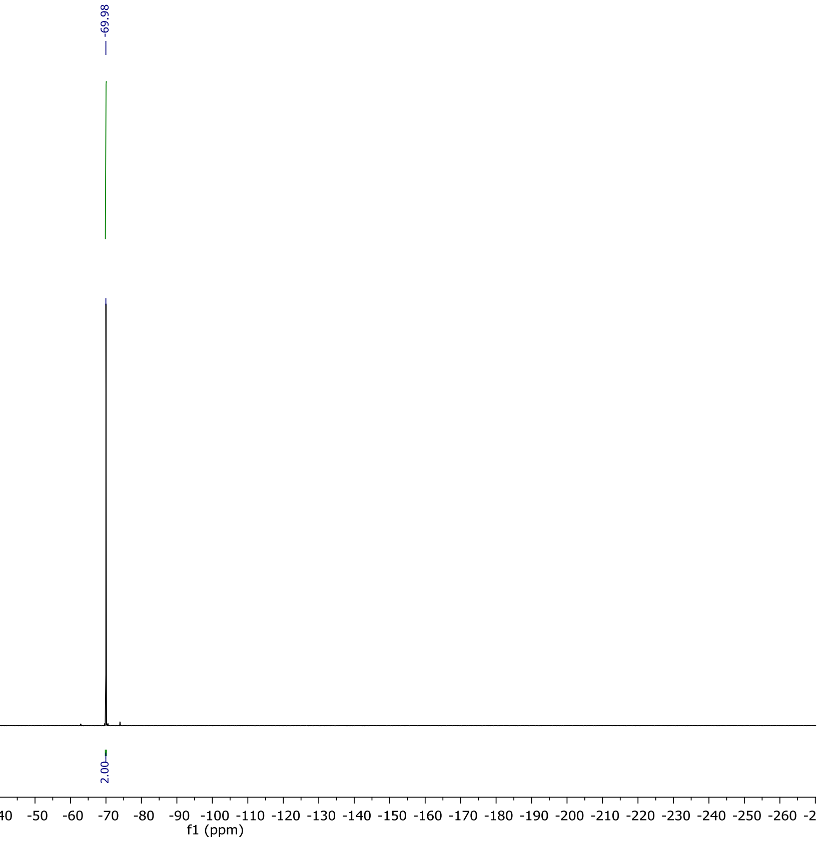

5.5 Scope of Sulfonamides

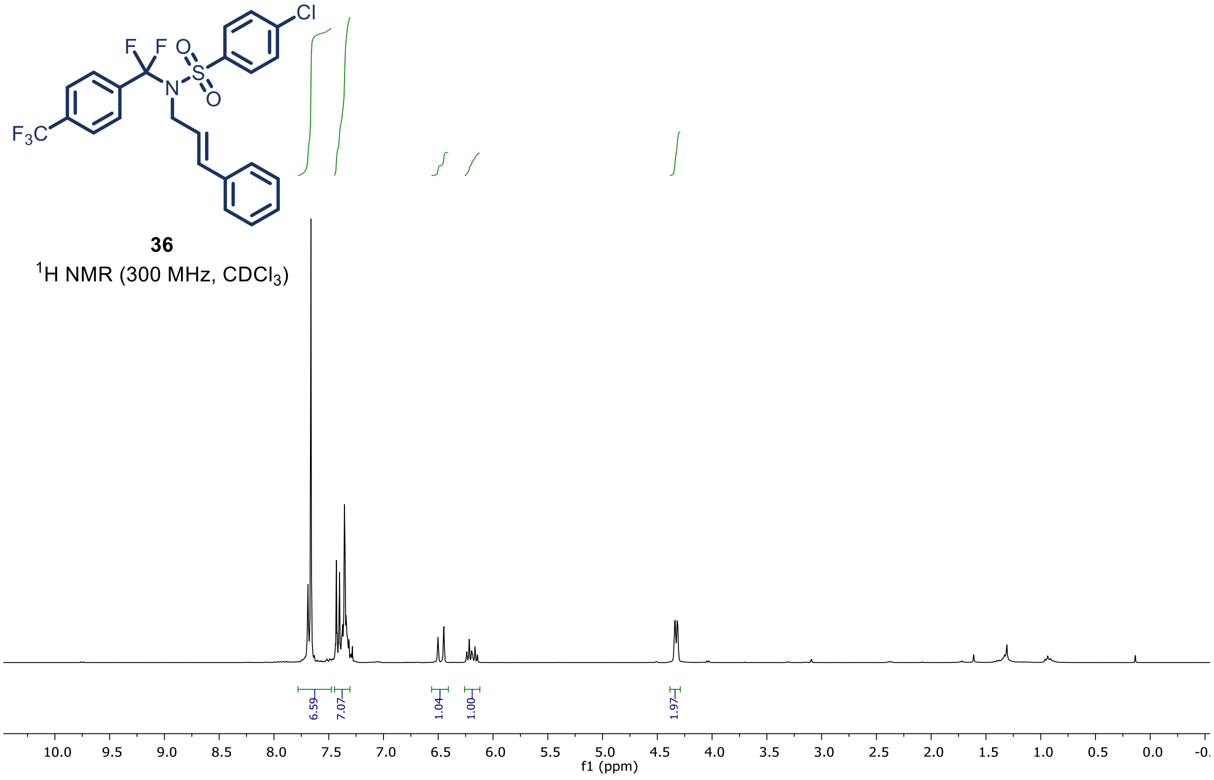

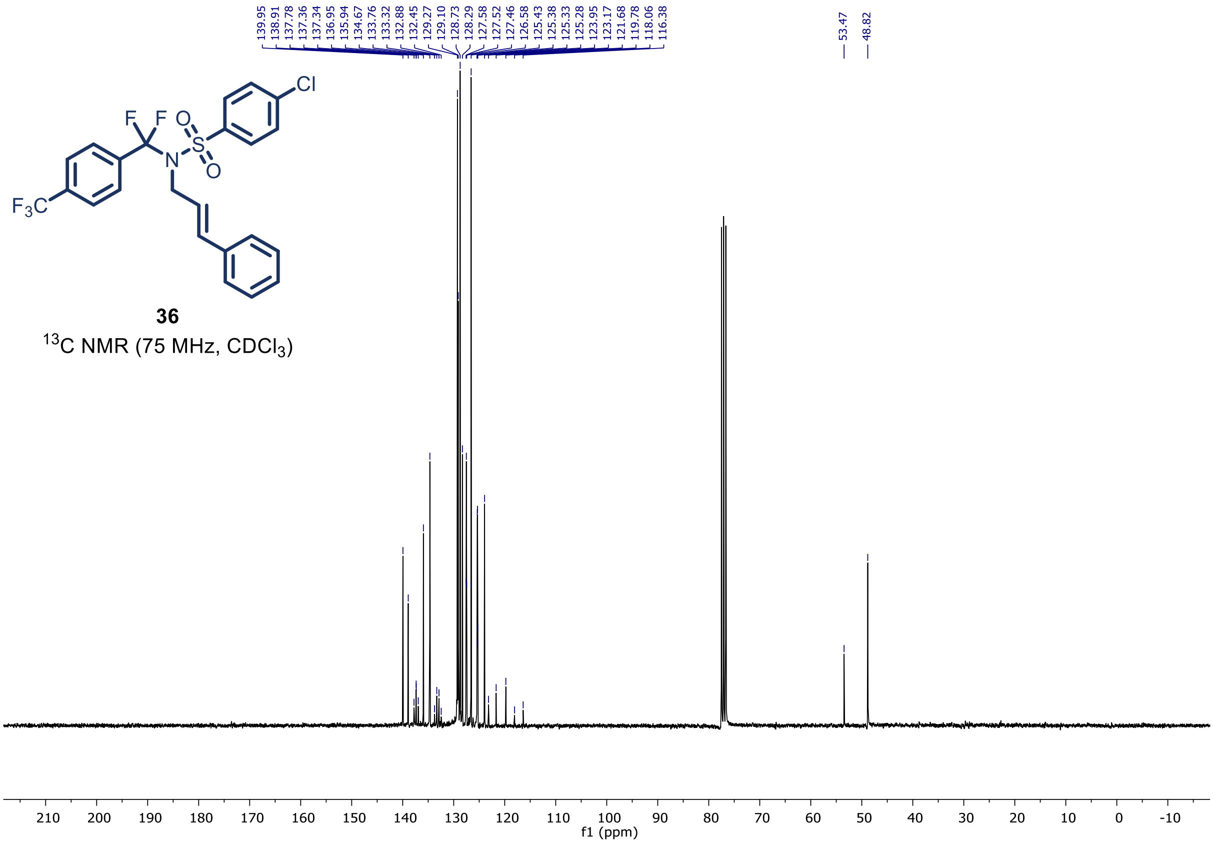

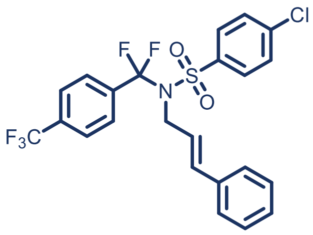

36

^{19}F NMR (282 MHz, $CDCl_3$)

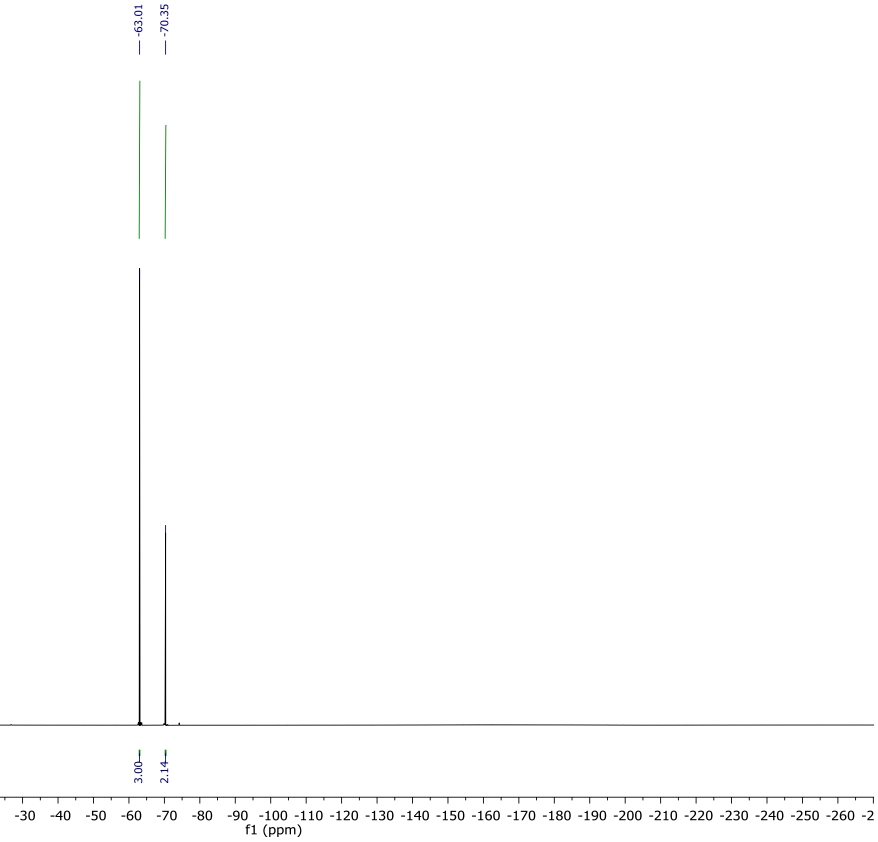

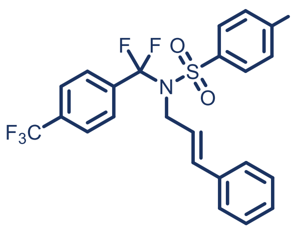

37

^{1}H NMR (300 MHz, $CDCl_3$)

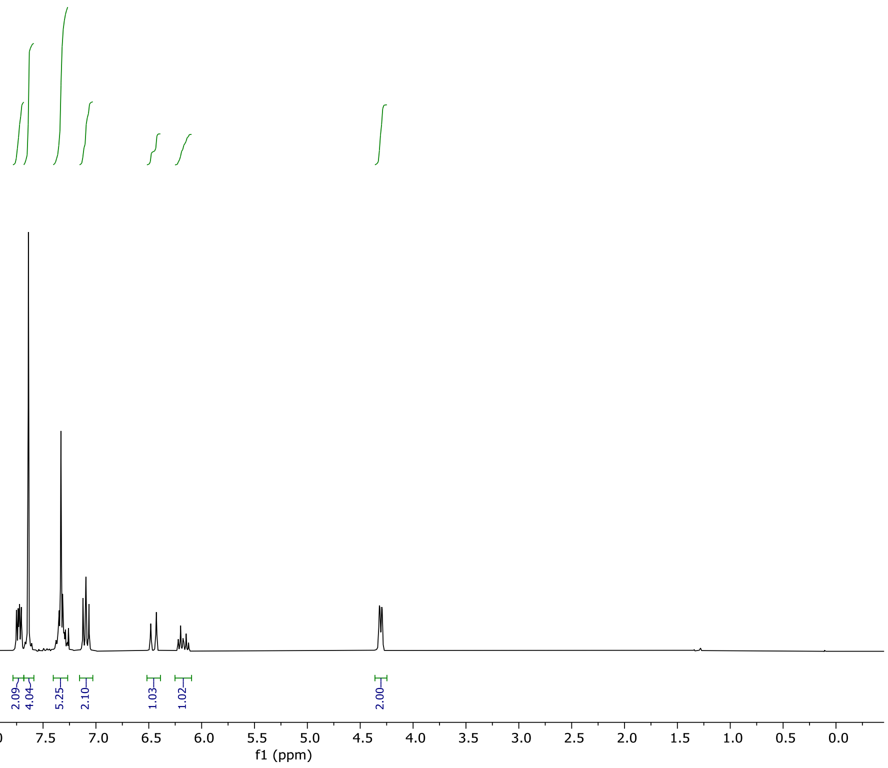

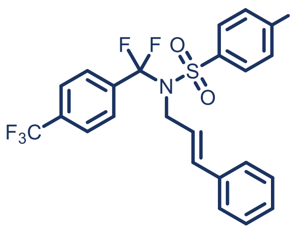

37

^{13}C NMR (75 MHz, $CDCl_3$)

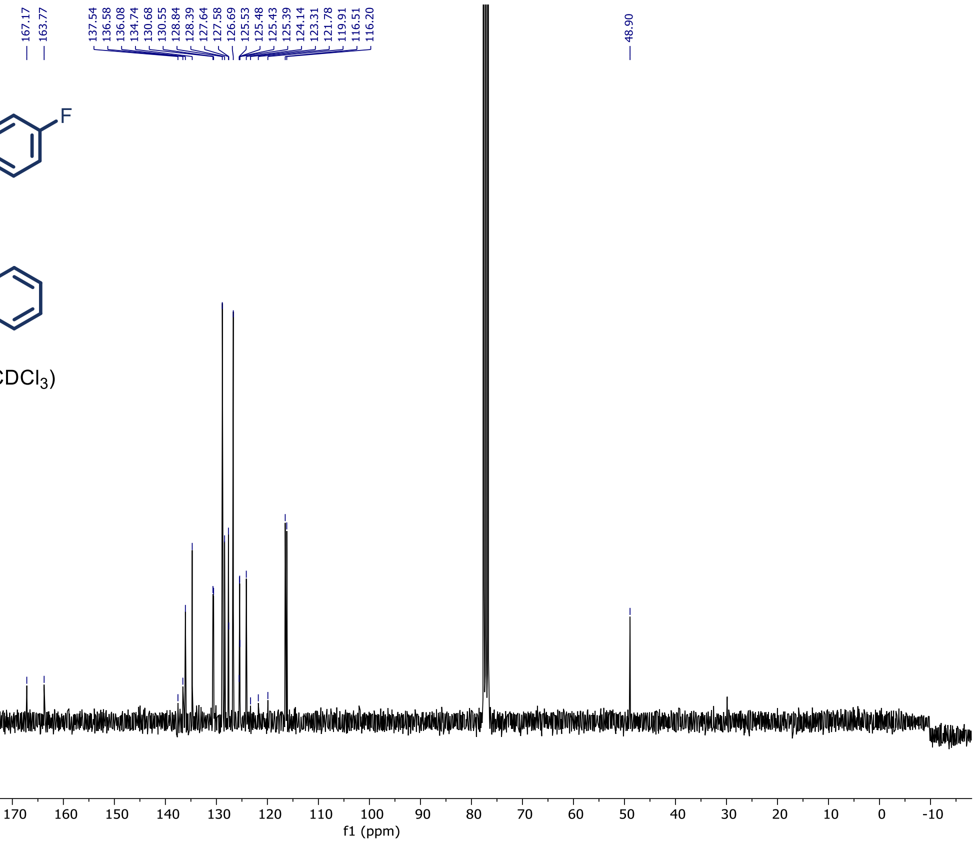

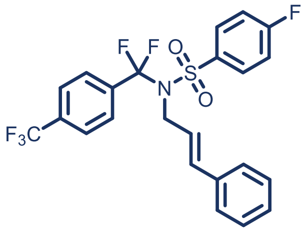

37

^{19}F NMR (282 MHz, $CDCl_3$)

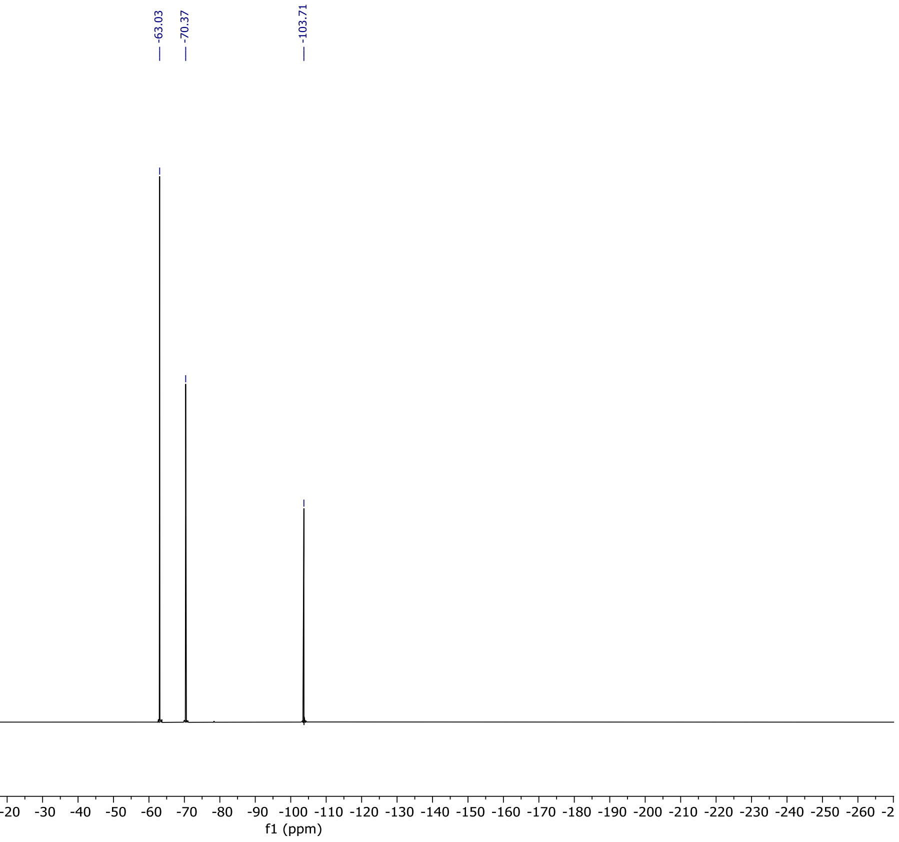

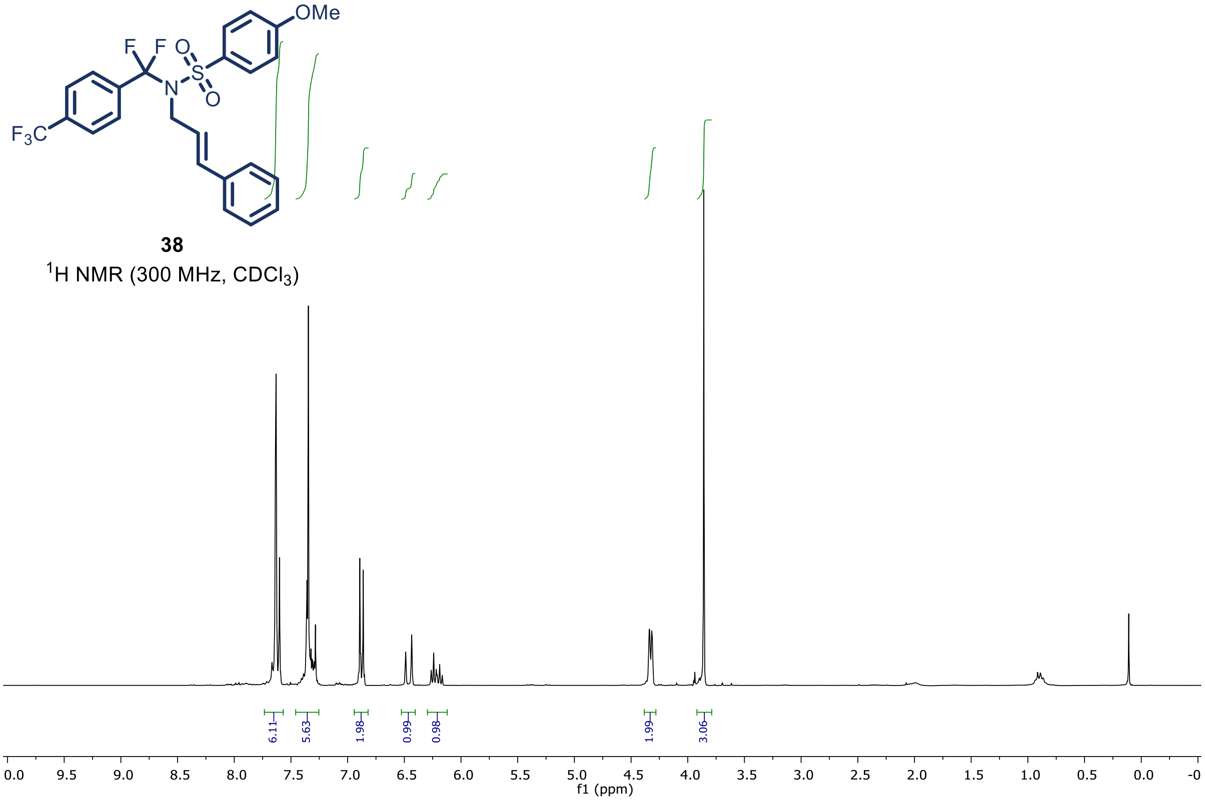

38

^{1}H NMR (300 MHz, $CDCl_3$)

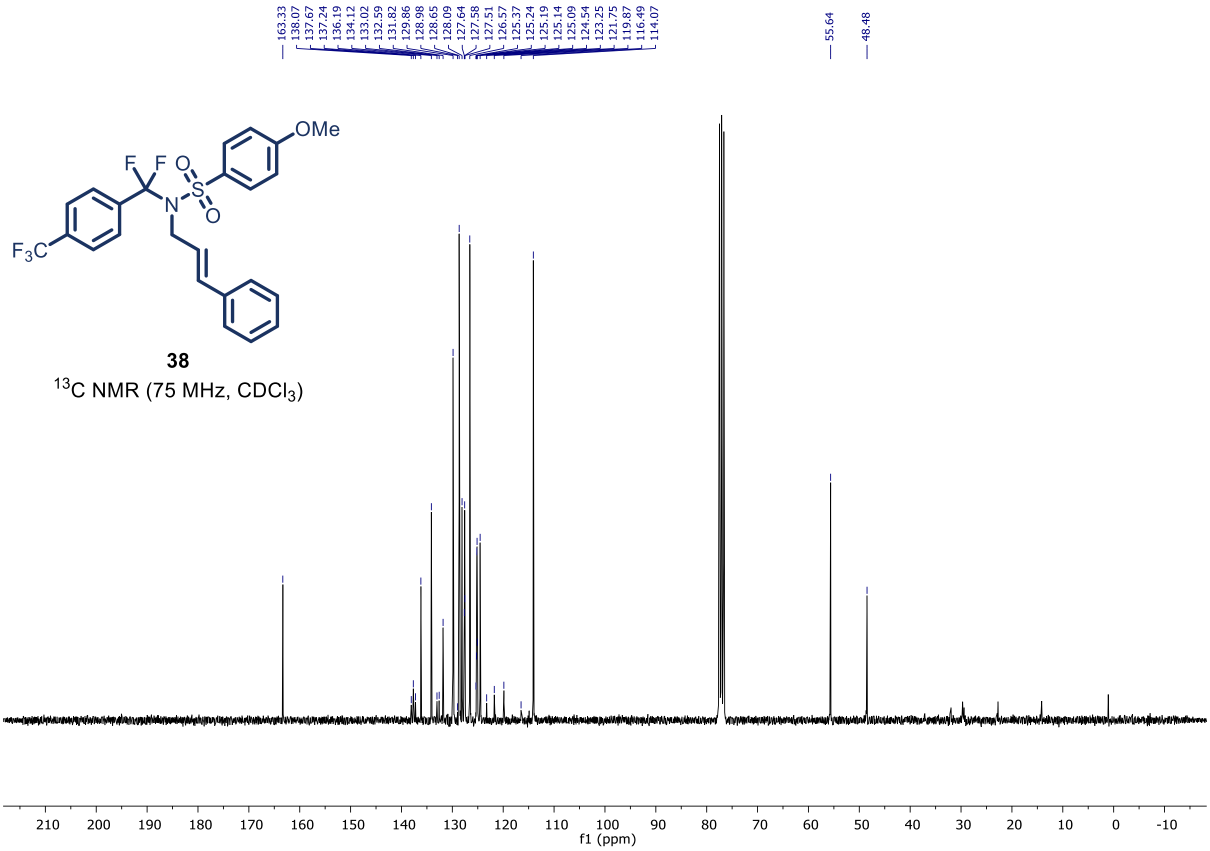

38

^{13}C NMR (75 MHz, $CDCl_3$)

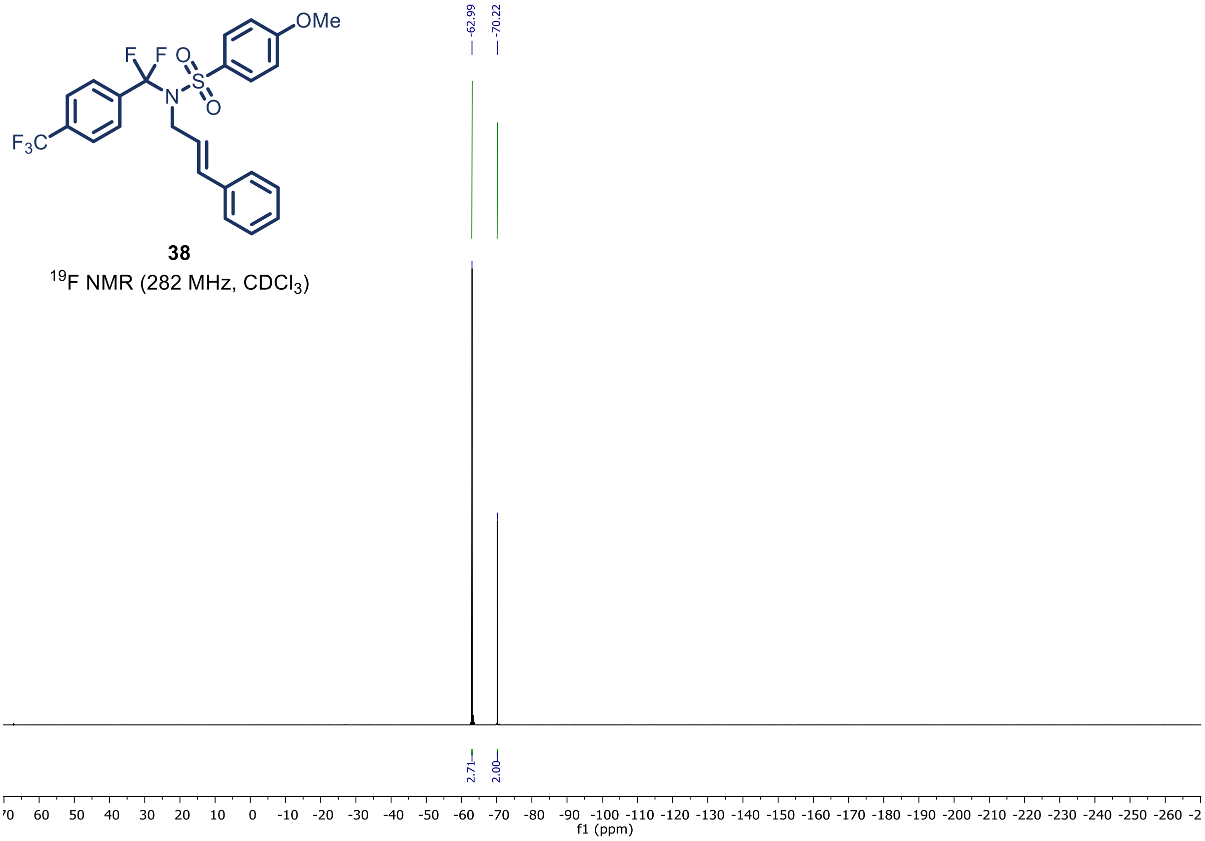

OMe
F F
O
S
N
O
F3C
38
19F NMR (282 MHz, CDCl3)
-62.99
-70.22
2.71
2.00
f1 (ppm)

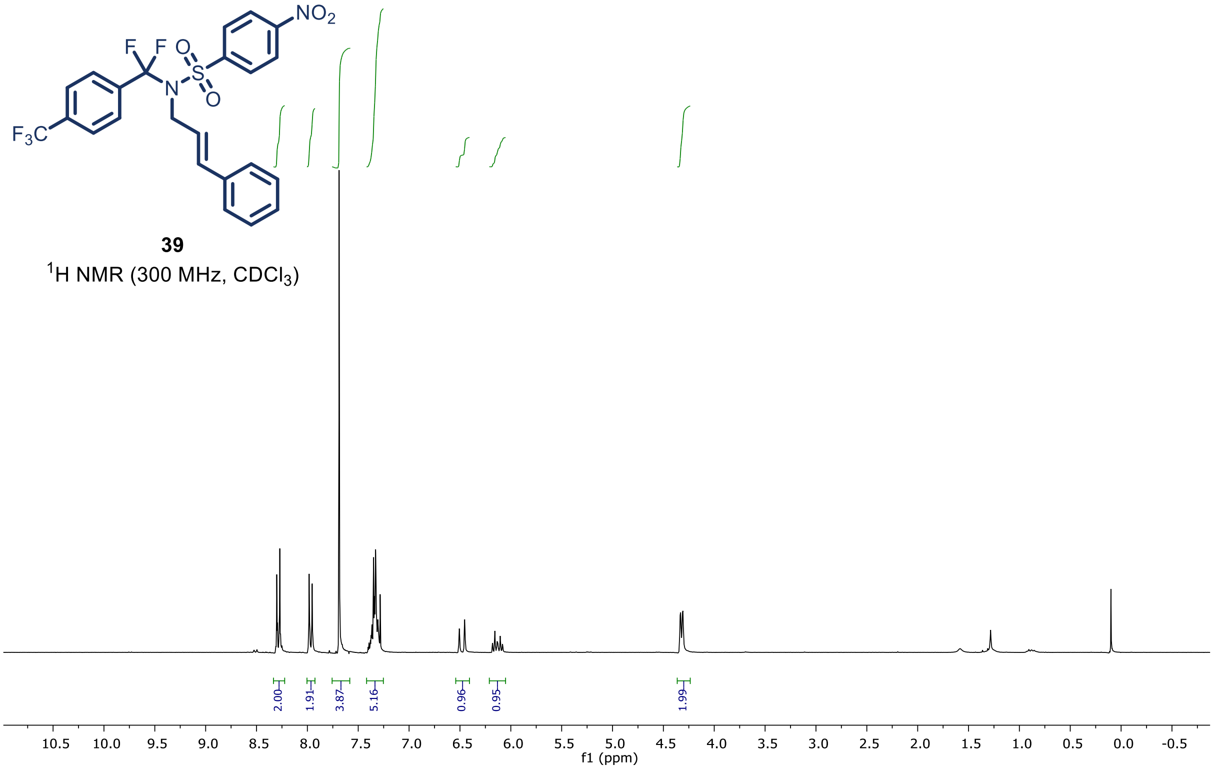

39

^{1}H NMR (300 MHz, $CDCl_3$)

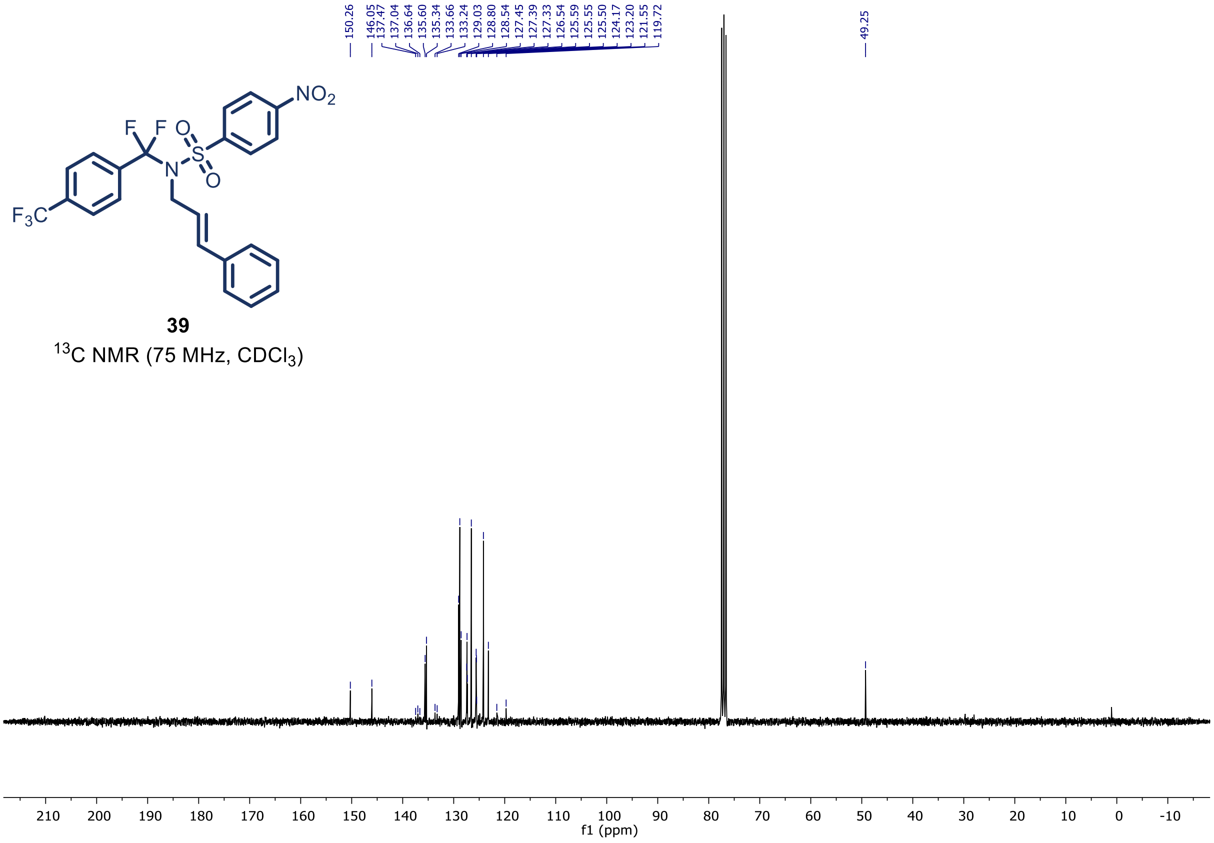

39

^{13}C NMR (75 MHz, $CDCl_3$)

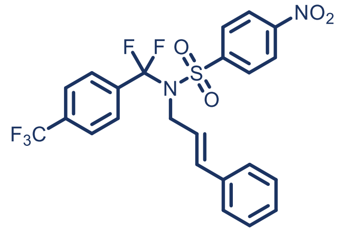

39

^{19}F NMR (282 MHz, $CDCl_3$)

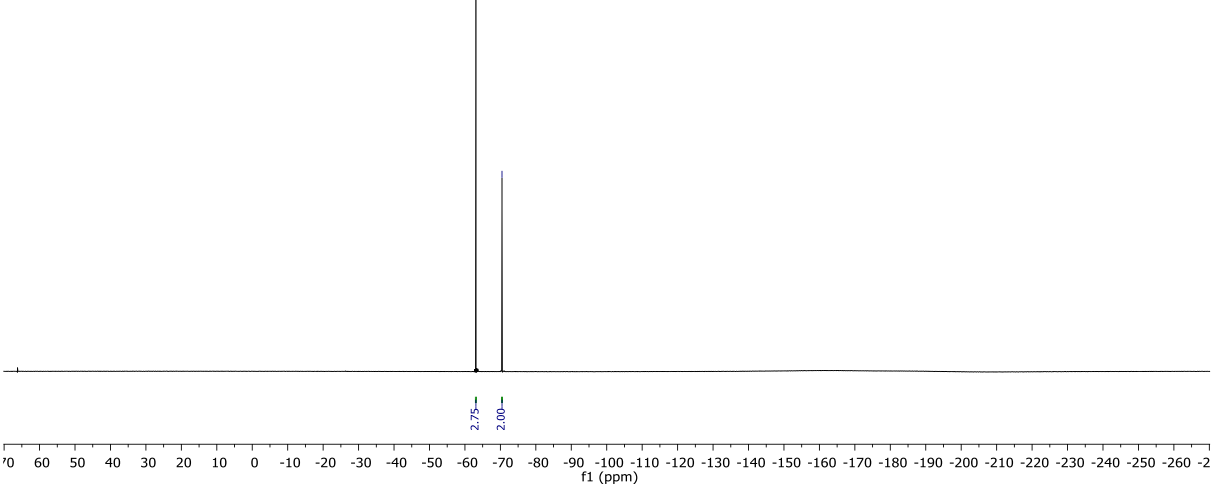

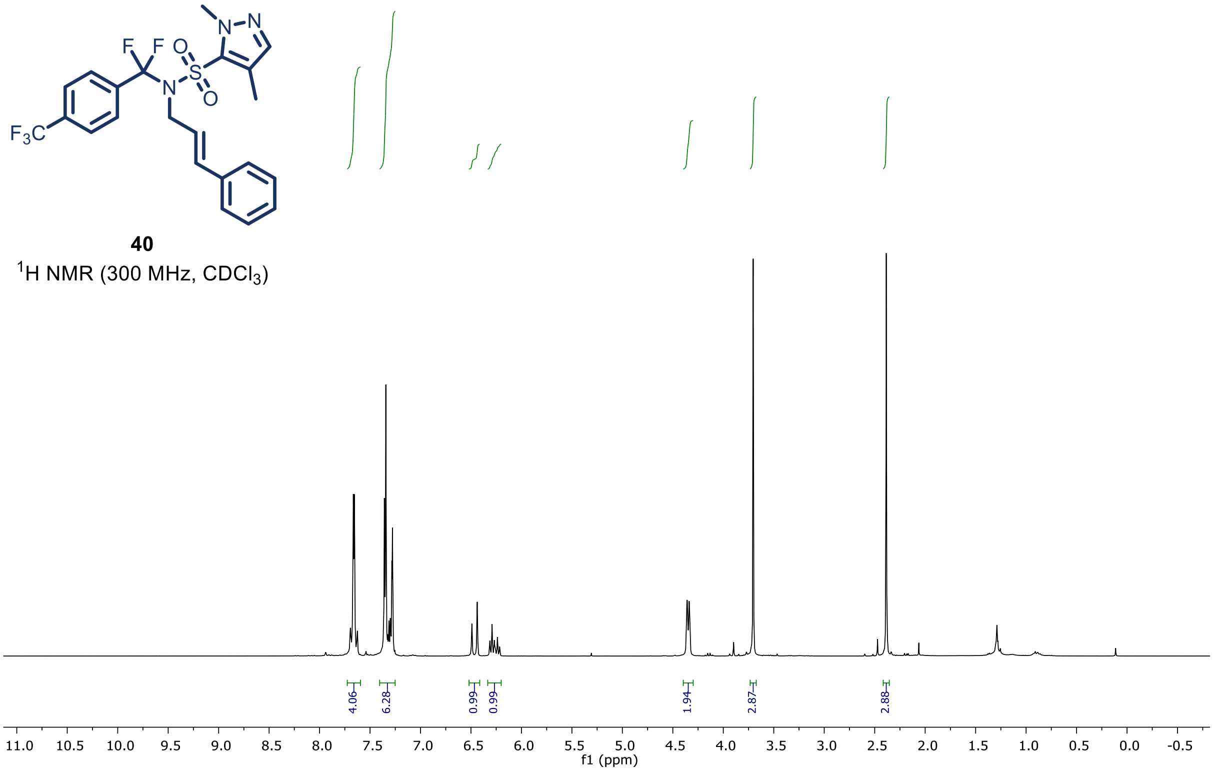
40
^{1}H NMR (300 MHz, CDCl$_3$)
4.06
6.28
0.99
0.99
1.94
2.87
2.88
f1 (ppm)

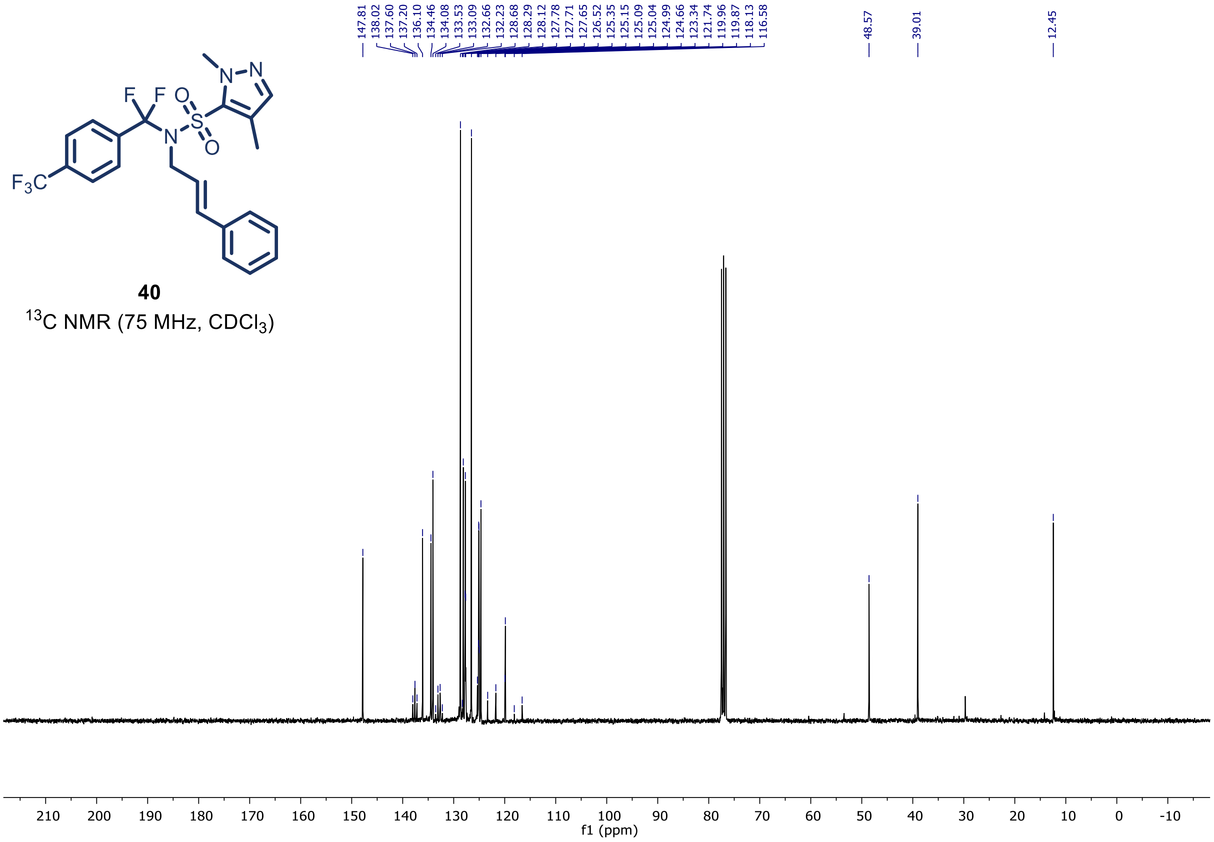
147.81
138.02
137.60
137.20
136.10
134.46
134.08
133.53
133.09
132.66
132.23
128.68
128.29
128.12
127.78
127.71
127.65
126.52
125.35
125.15
125.09
125.04
124.99
124.66
123.34
121.74
119.96
119.87
118.13
116.58
48.57
39.01
12.45
40
^{13}C NMR (75 MHz, CDCl$_3$)
f1 (ppm)

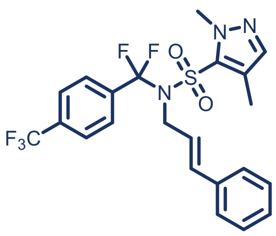

40

^{19}F NMR (282 MHz, $CDCl_3$)

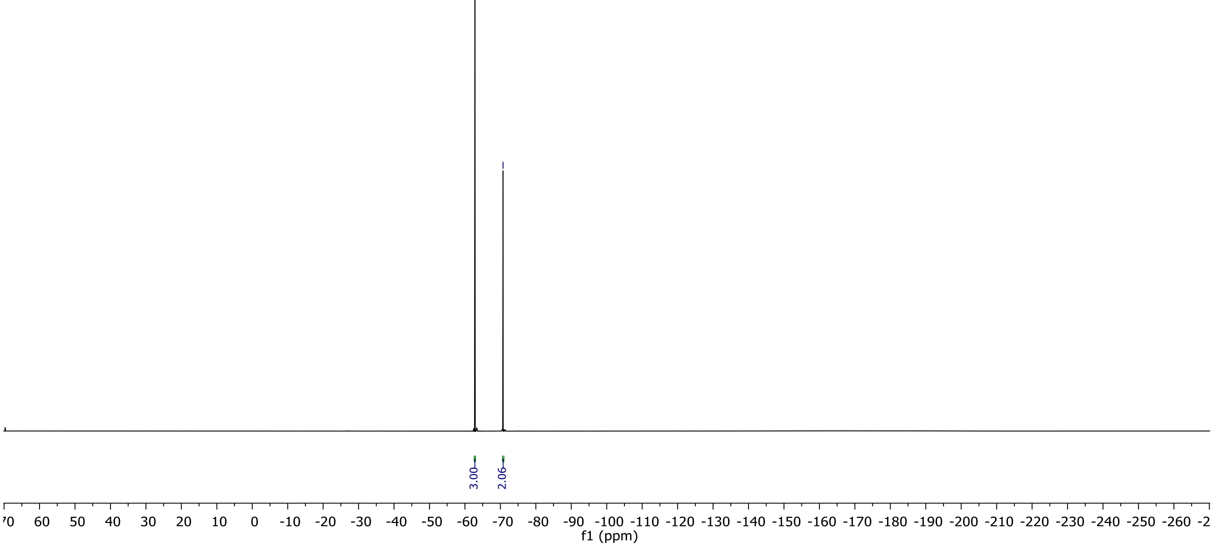

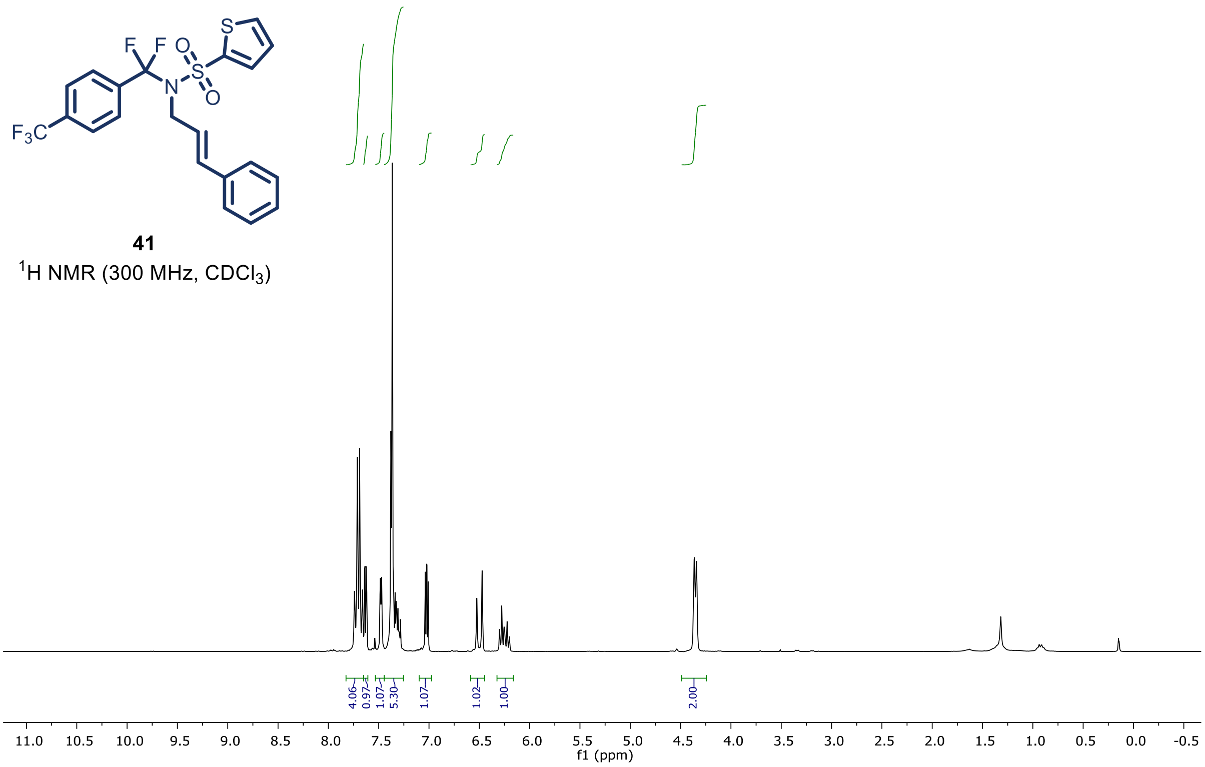
F
F
O
S
N
O
F_3C
41
^1H NMR (300 MHz, $CDCl_3$)
4.06
0.97
1.07
5.30
1.07
1.02
1.00
2.00
11.0 10.5 10.0 9.5 9.0 8.5 8.0 7.5 7.0 6.5 6.0 5.5 5.0 4.5 4.0 3.5 3.0 2.5 2.0 1.5 1.0 0.5 0.0 -0.5
f1 (ppm)

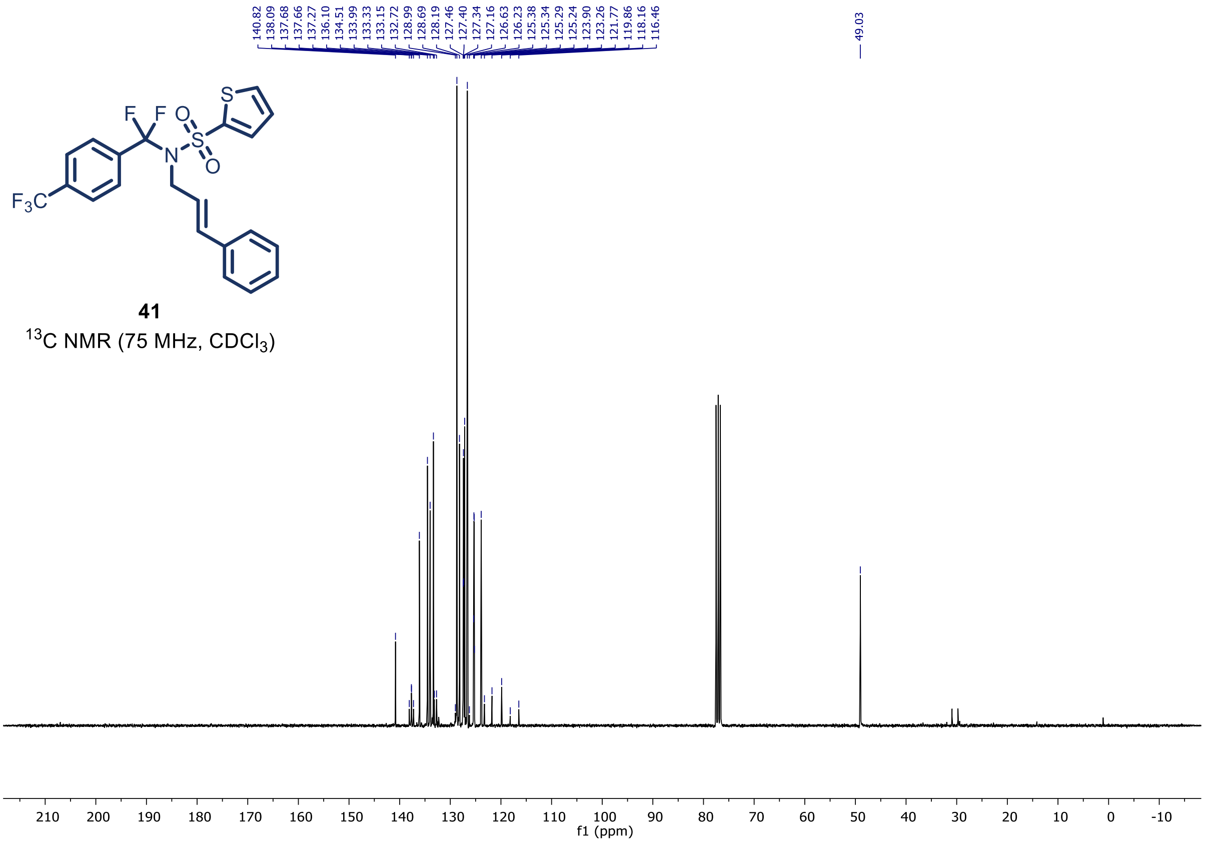
140.82
138.09
137.68
137.66
137.27
136.10
134.51
133.99
133.33
133.15
132.72
128.99
128.69
128.19
127.46
127.40
127.34
127.16
126.63
126.23
125.38
125.34
125.29
125.24
123.90
123.26
121.77
119.86
118.16
116.46
49.03
F
F
O
S
N
O
F_3C
41
^{13}C NMR (75 MHz, $CDCl_3$)
210 200 190 180 170 160 150 140 130 120 110 100 90 80 70 60 50 40 30 20 10 0 -10
f1 (ppm)

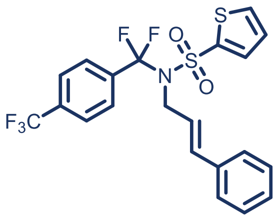

41

^{19}F NMR (282 MHz, $CDCl_3$)

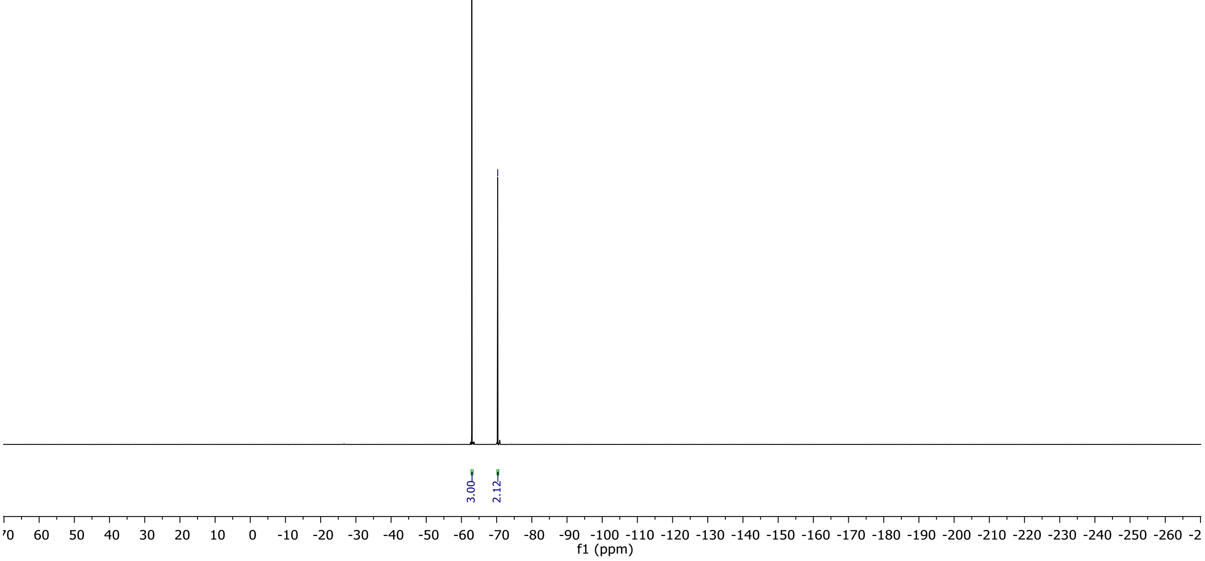

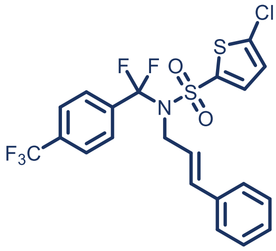

42

^{1}H NMR (300 MHz, $CDCl_3$)

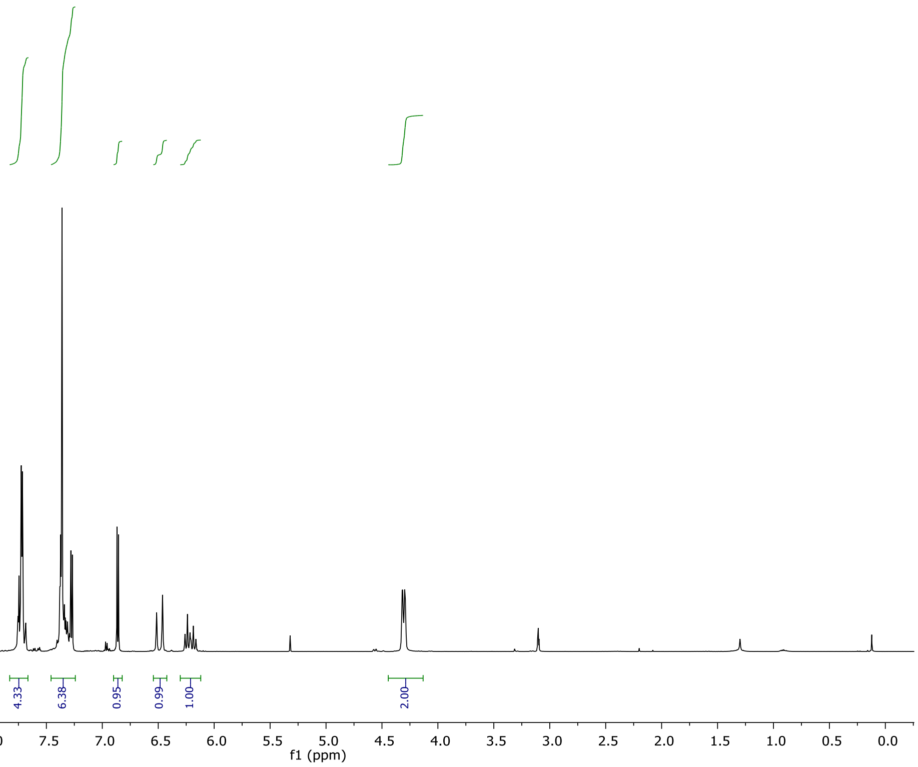

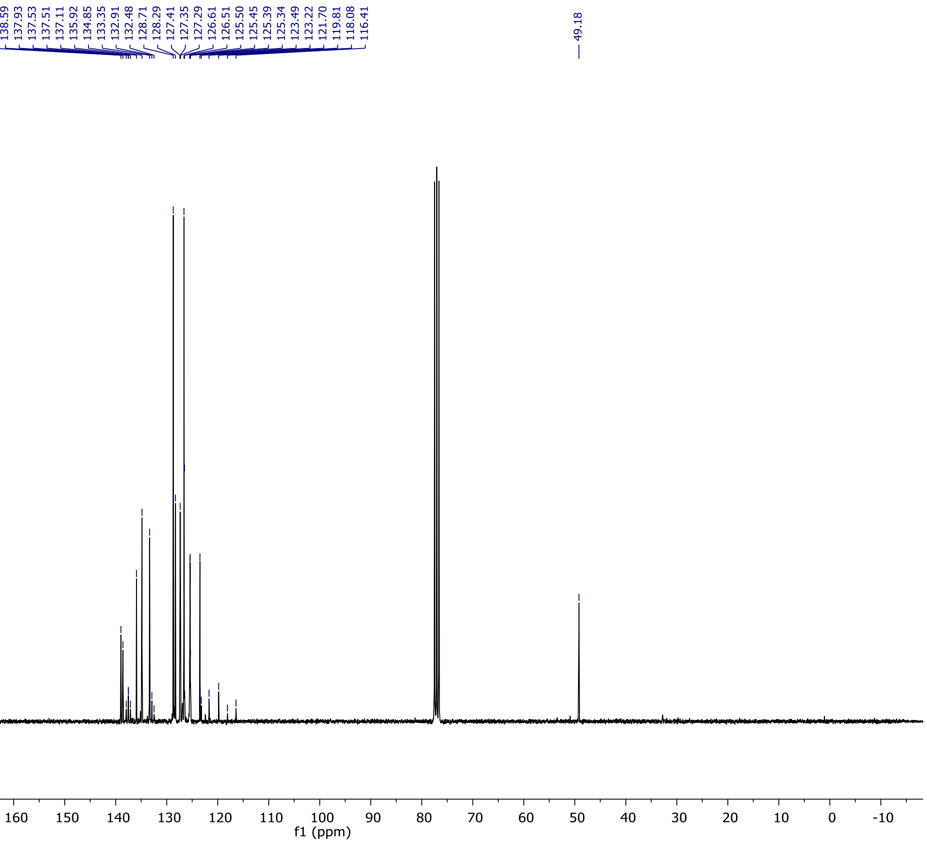

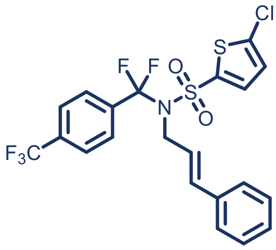

42

^{13}C NMR (75 MHz, $CDCl_3$)

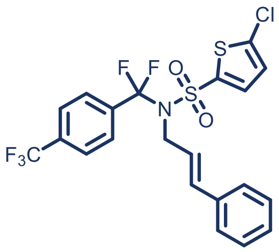

42

^{19}F NMR (282 MHz, $CDCl_3$)

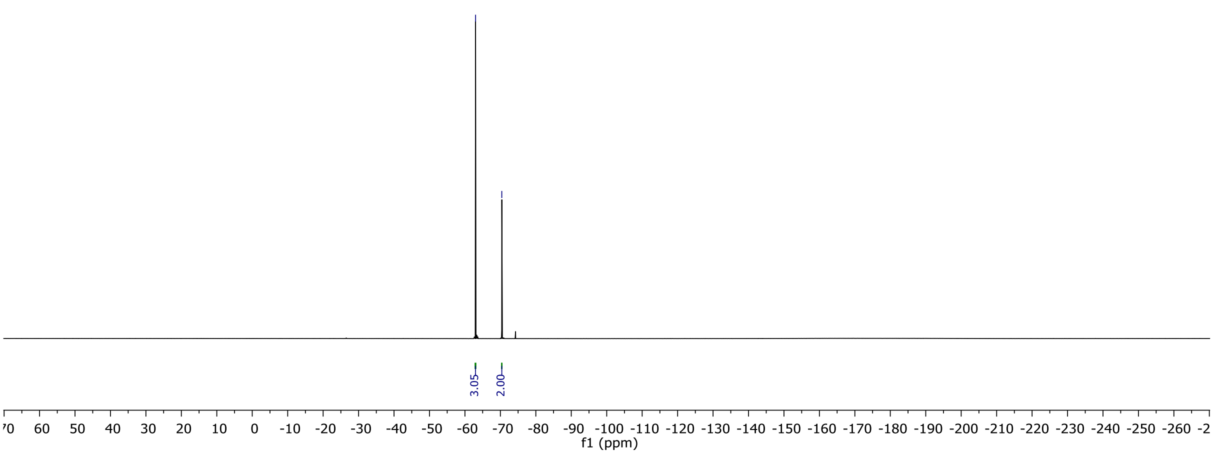

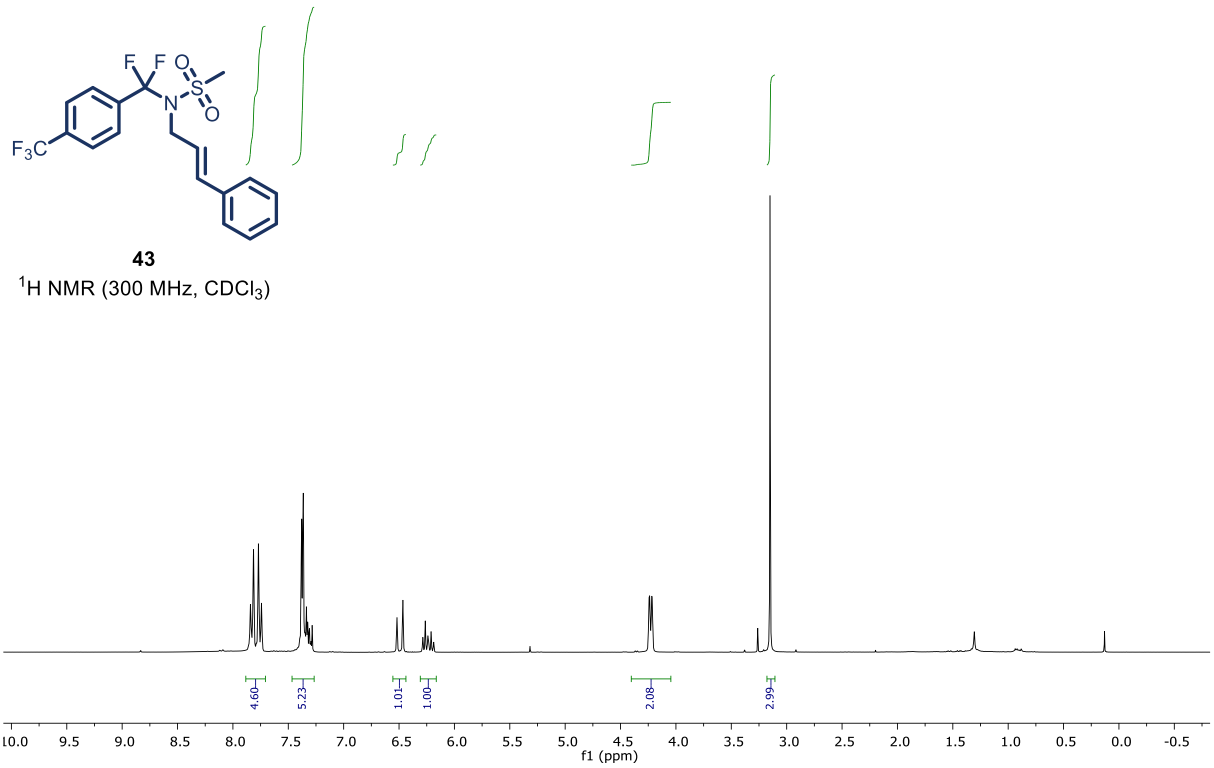

43

^{1}H NMR (300 MHz, $CDCl_3$)

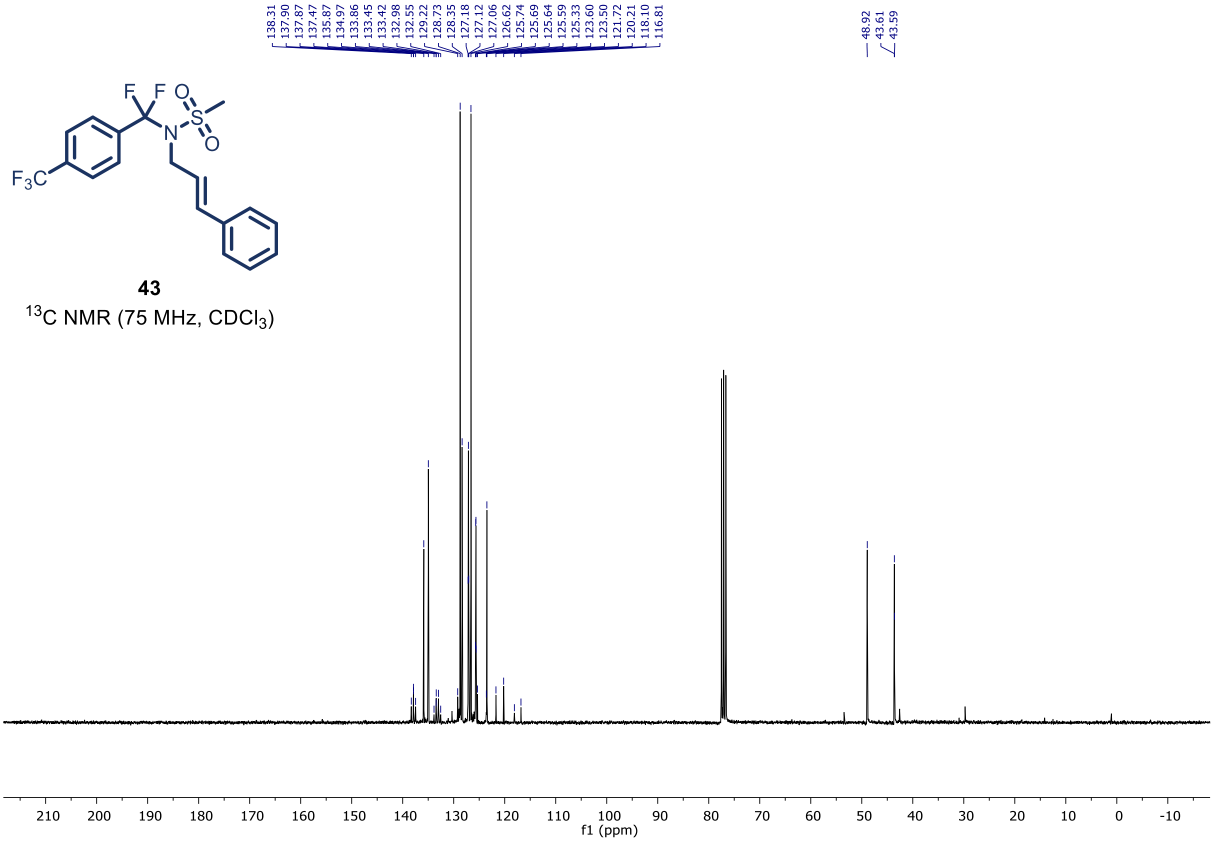

43

^{13}C NMR (75 MHz, $CDCl_3$)

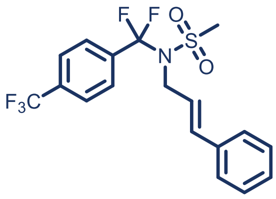

43

^{19}F NMR (282 MHz, $CDCl_3$)

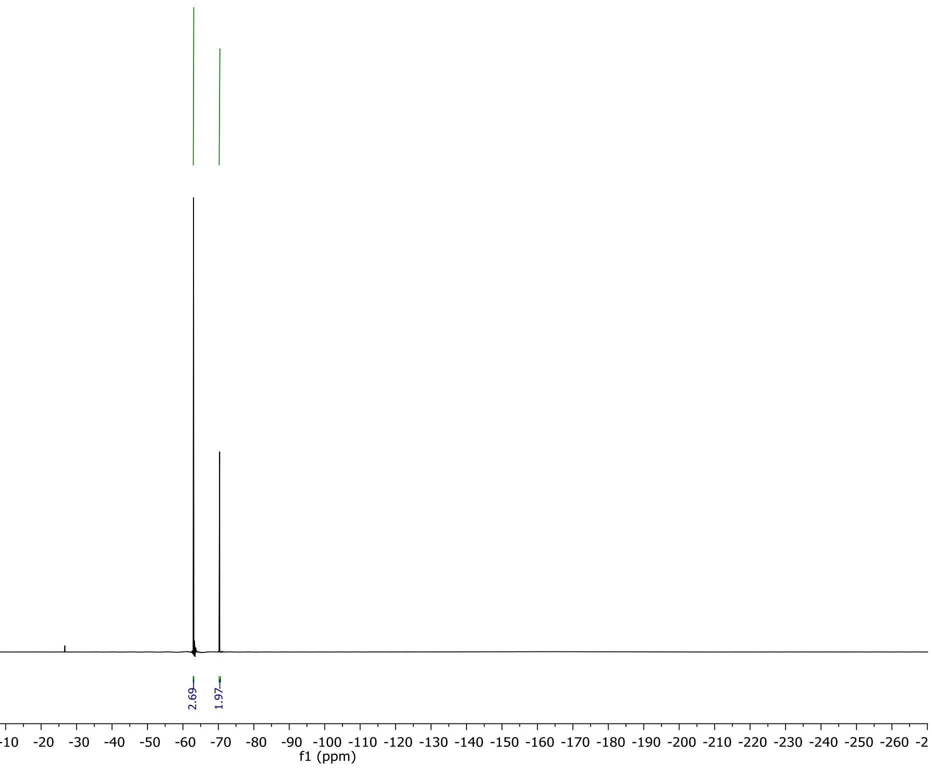

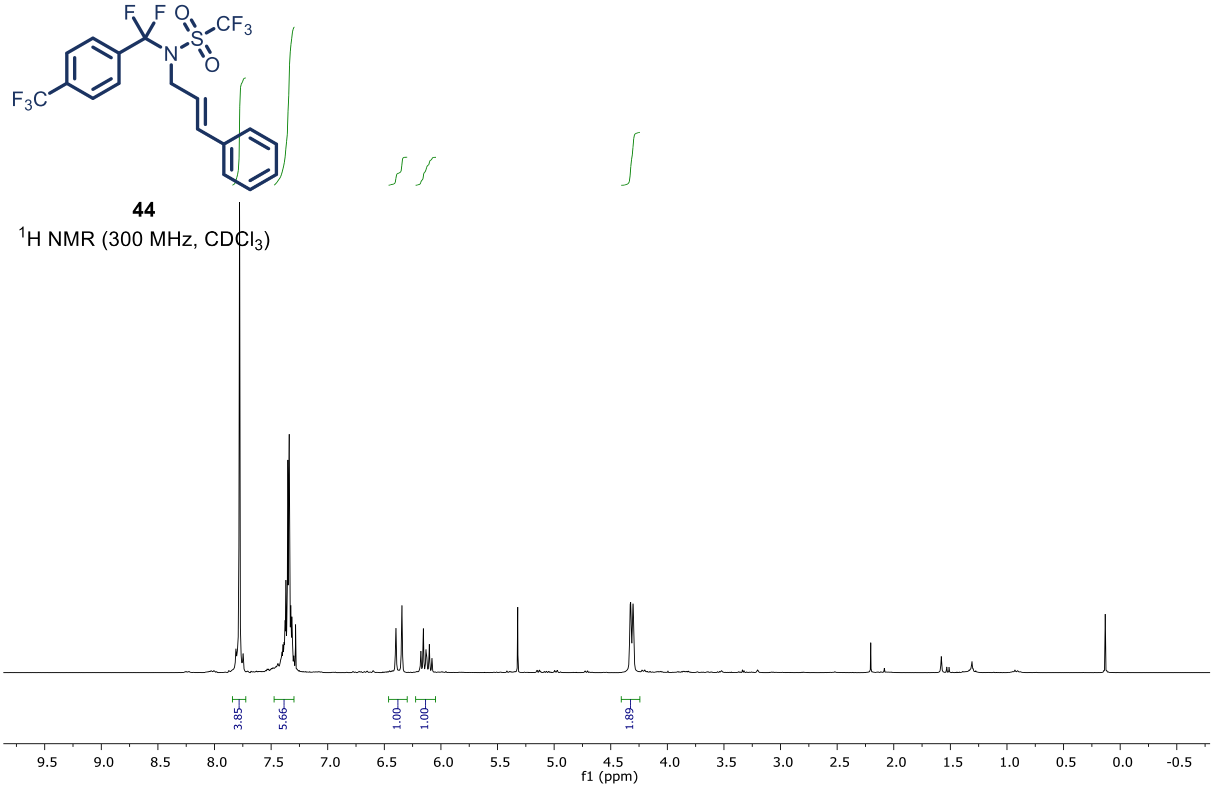

44

^{1}H NMR (300 MHz, $CDCl_3$)

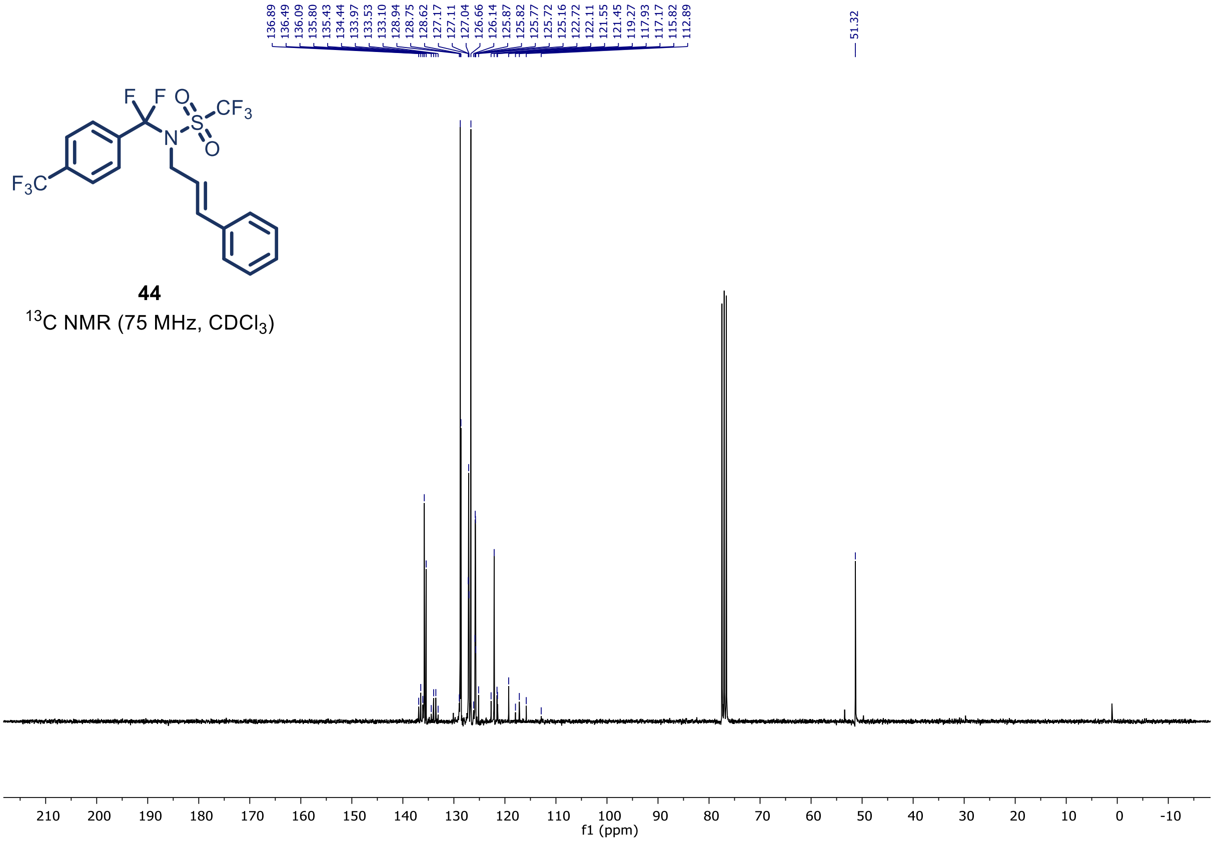

44

^{13}C NMR (75 MHz, $CDCl_3$)

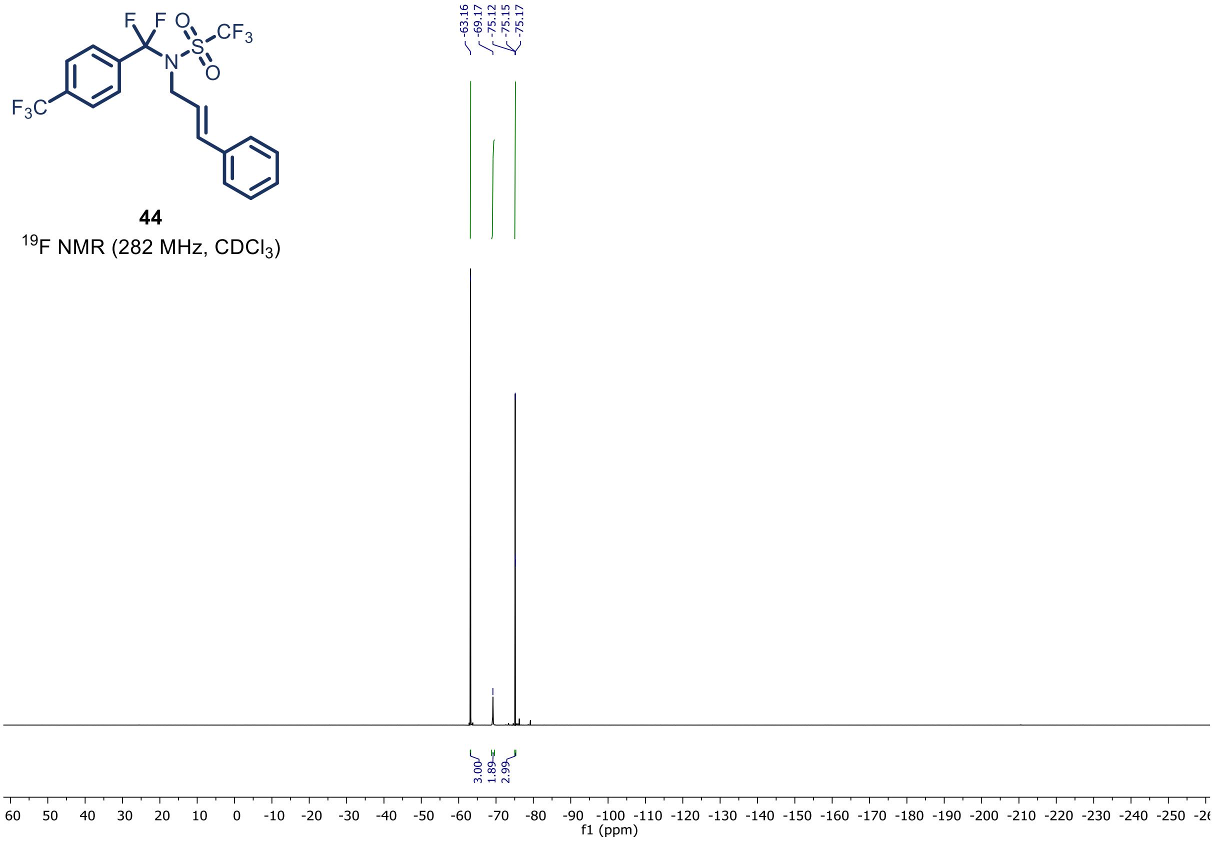

44

^{19}F NMR (282 MHz, $CDCl_3$)

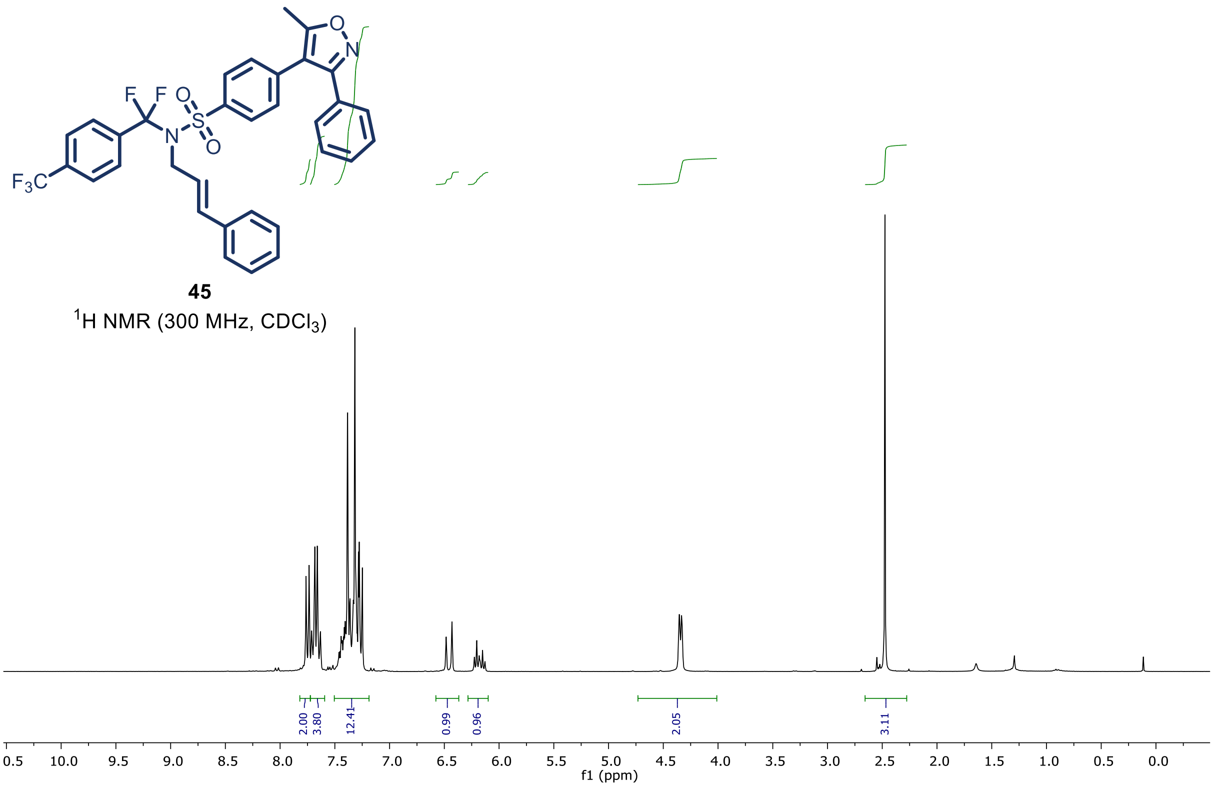
45
¹H NMR (300 MHz, CDCl₃)
2.00
3.80
12.41
0.99
0.96
2.05
3.11
0.5 10.0 9.5 9.0 8.5 8.0 7.5 7.0 6.5 6.0 5.5 5.0 4.5 4.0 3.5 3.0 2.5 2.0 1.5 1.0 0.5 0.0
f1 (ppm)

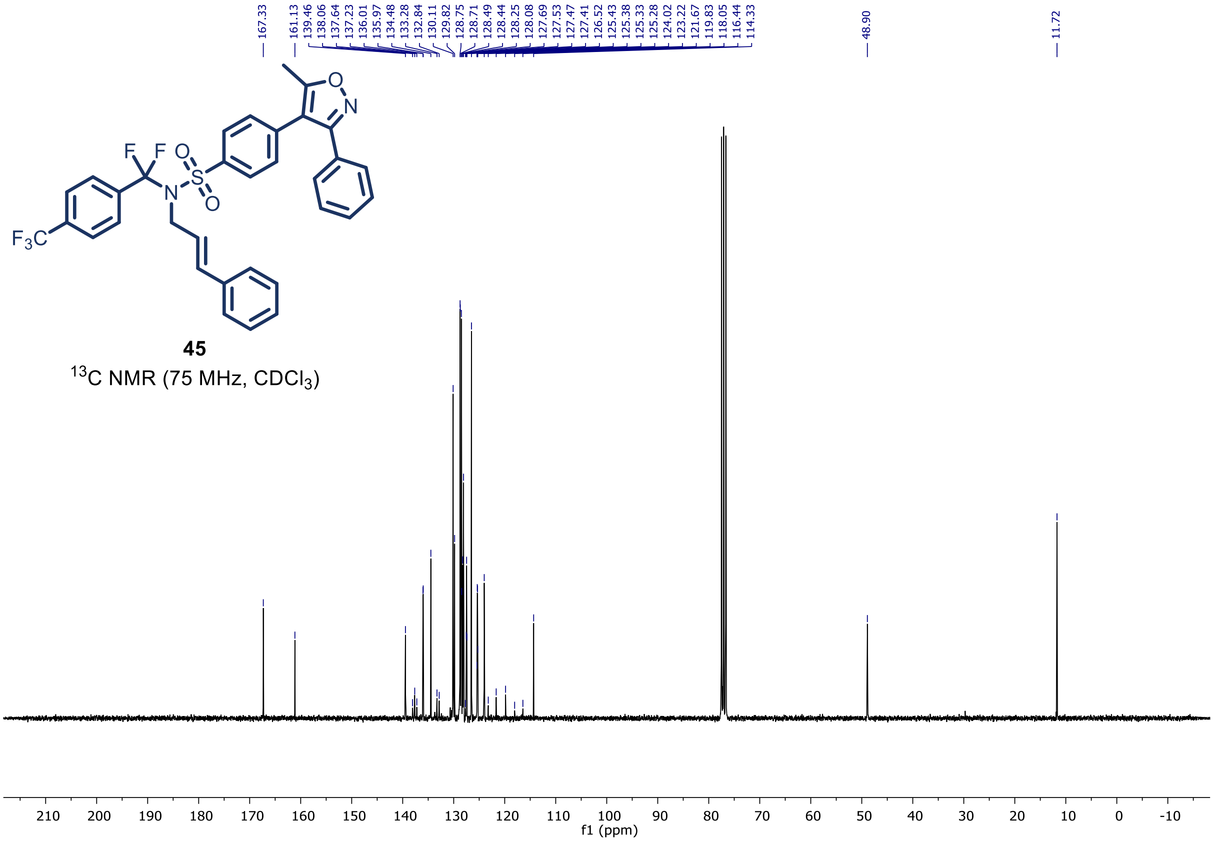
167.33
161.13
139.46
138.06
137.64
137.23
136.01
135.97
134.48
133.28
132.84
130.11
129.82
128.75
128.71
128.49
128.44
128.25
128.08
127.69
127.53
127.47
127.41
126.52
125.43
125.38
125.33
125.28
124.02
123.22
121.67
119.83
118.05
116.44
114.33
48.90
11.72
45
¹³C NMR (75 MHz, CDCl₃)
210 200 190 180 170 160 150 140 130 120 110 100 90 80 70 60 50 40 30 20 10 0 -10
f1 (ppm)

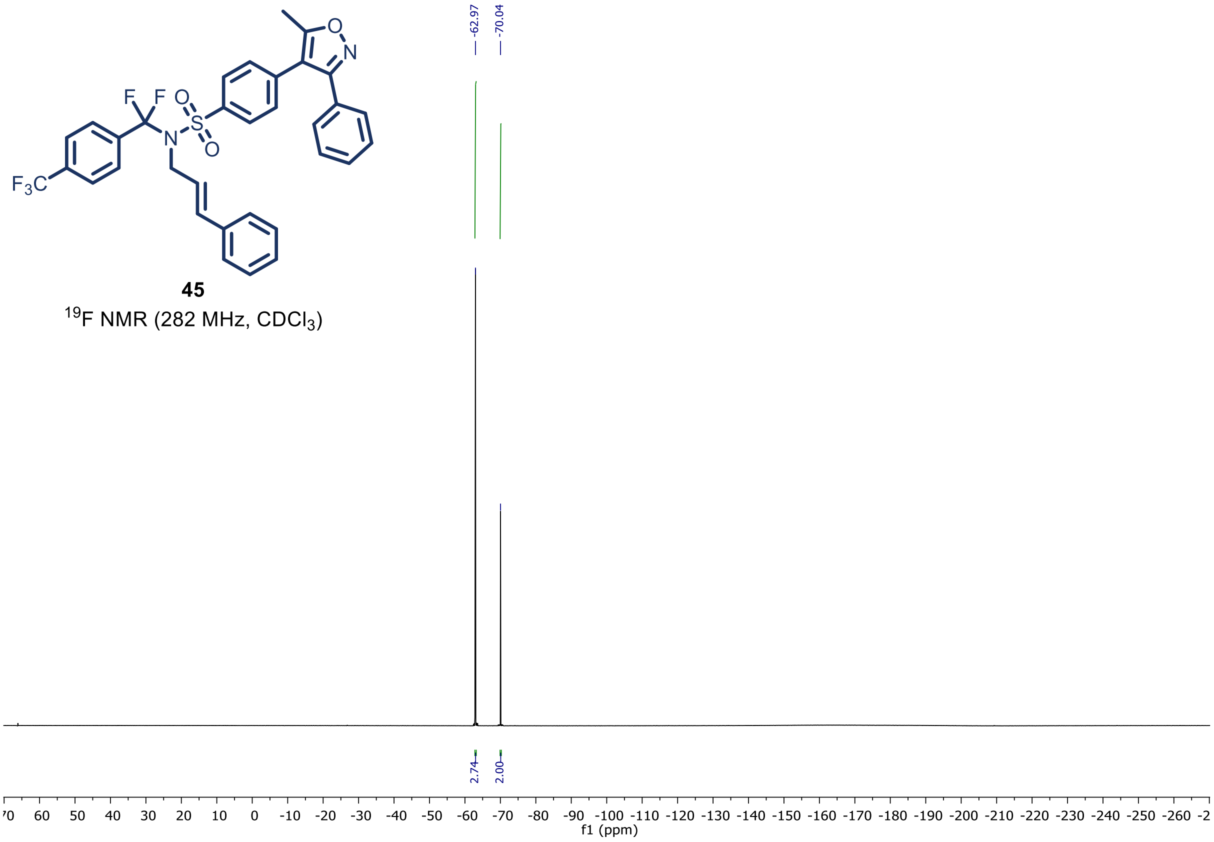

F F
O
N
S
O
O
N
F3C
45
19F NMR (282 MHz, CDCl3)
-62.97
-70.04
2.74
2.00
70 60 50 40 30 20 10 0 -10 -20 -30 -40 -50 -60 -70 -80 -90 -100 -110 -120 -130 -140 -150 -160 -170 -180 -190 -200 -210 -220 -230 -240 -250 -260 -2
f1 (ppm)

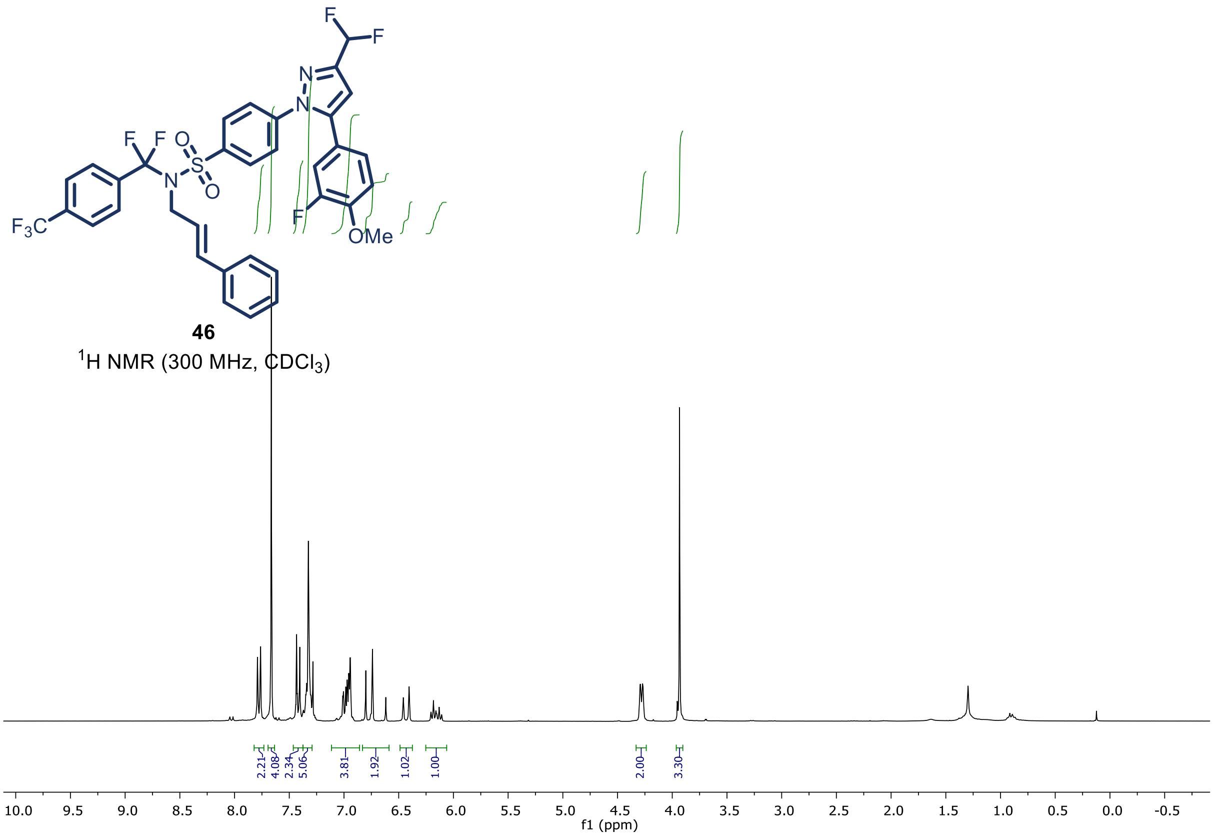

46

^{1}H NMR (300 MHz, $CDCl_3$)

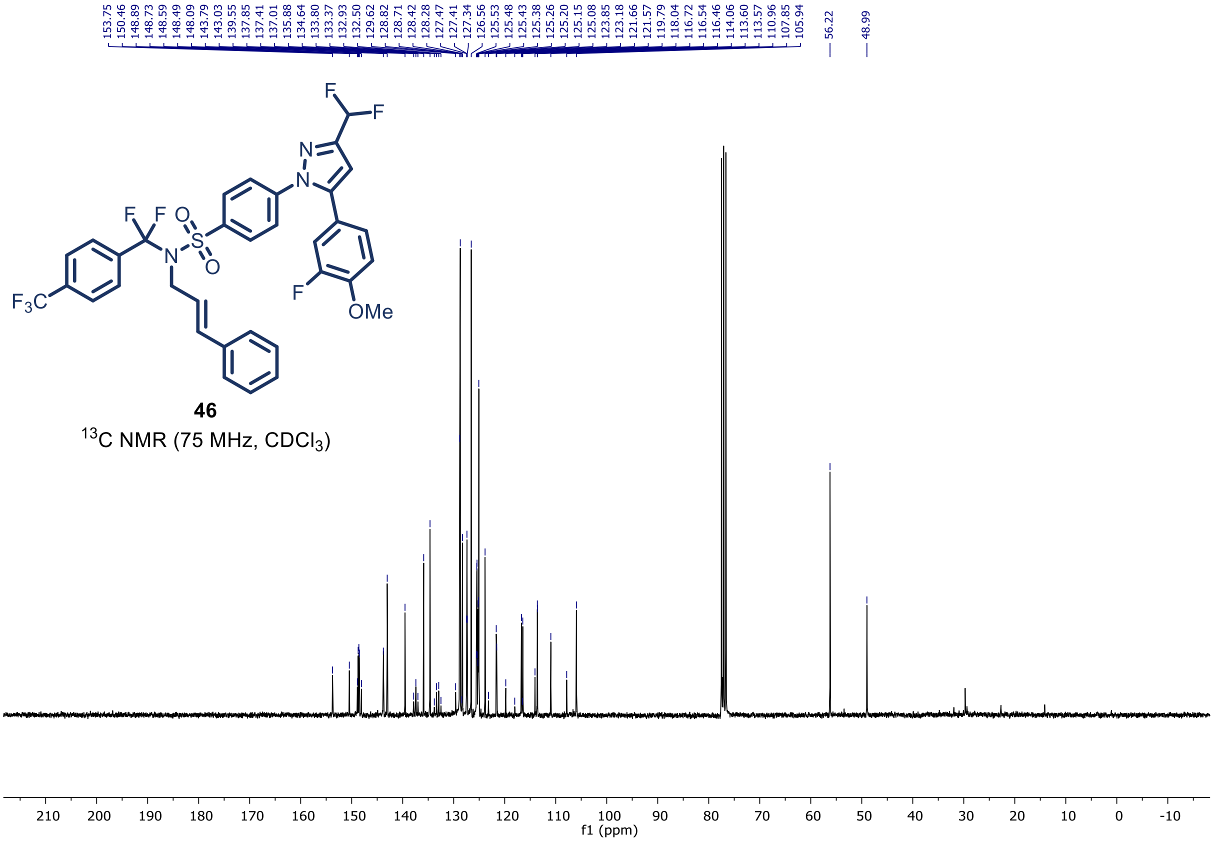

46

^{13}C NMR (75 MHz, $CDCl_3$)

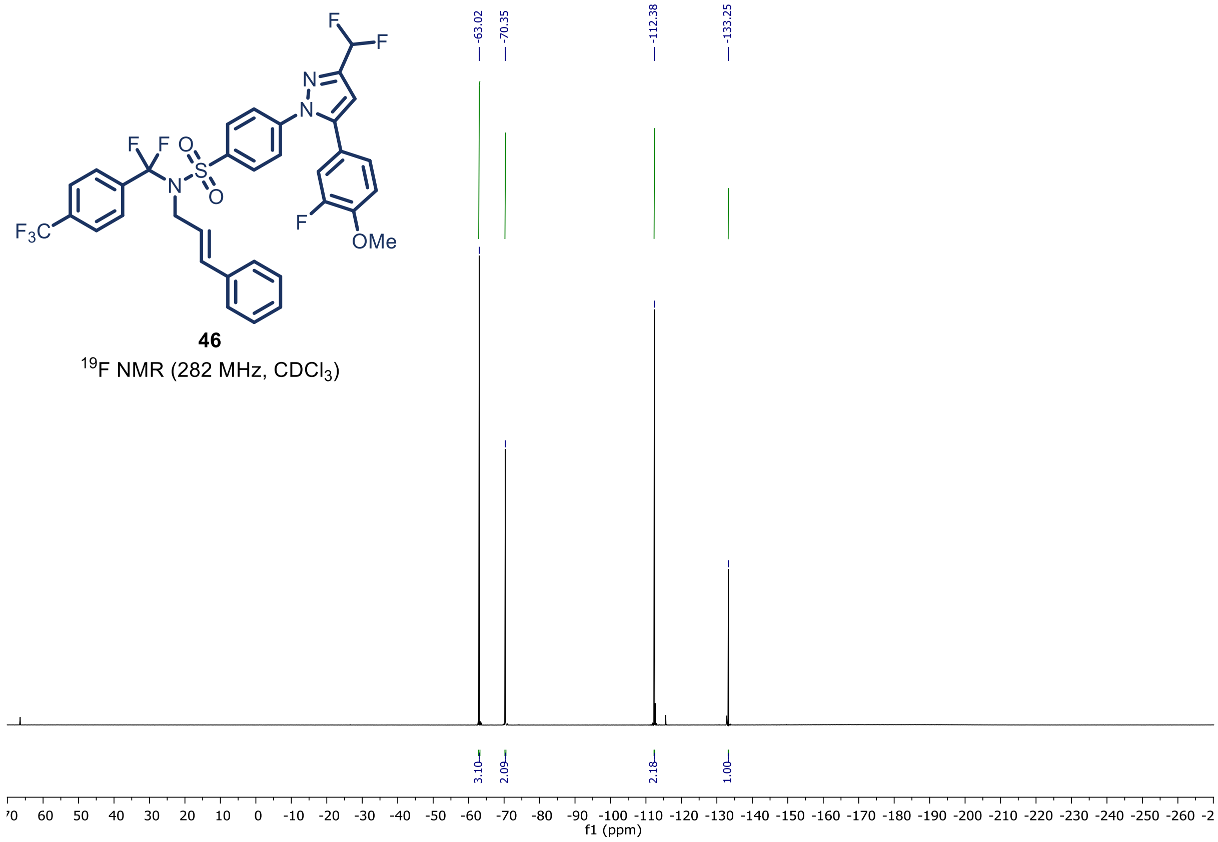

46

^{19}F NMR (282 MHz, $CDCl_3$)

5.6 Applications

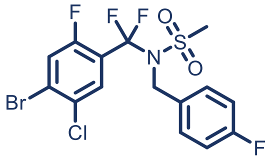

48

^{1}H NMR (300 MHz, $CDCl_3$)

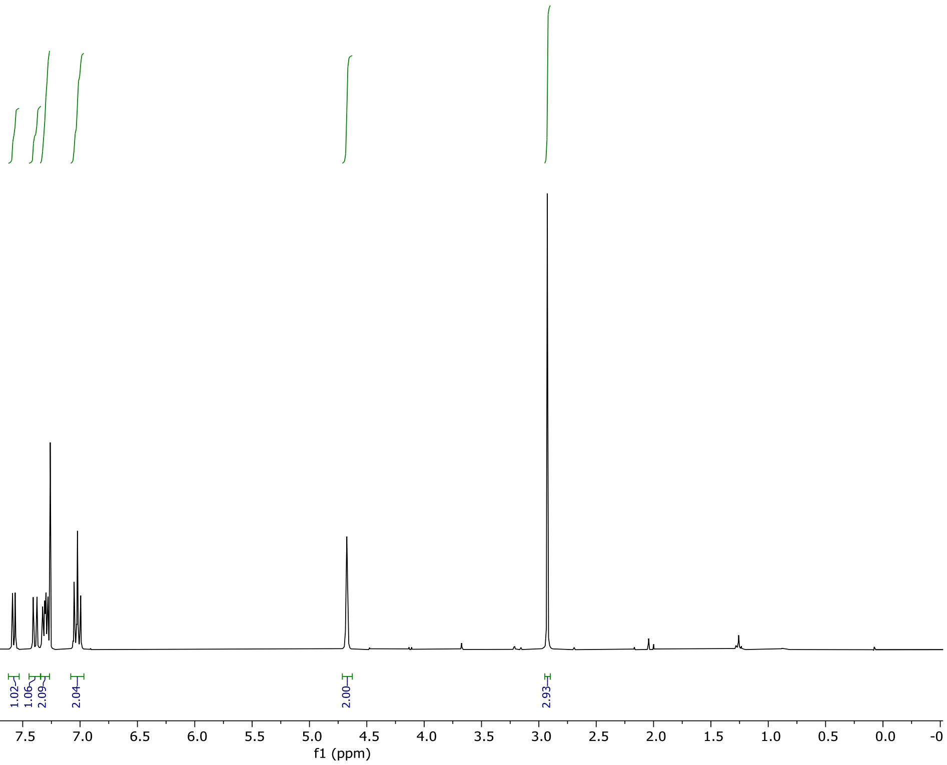

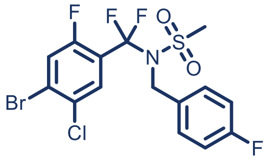

48

^{13}C NMR (75 MHz, $CDCl_3$)

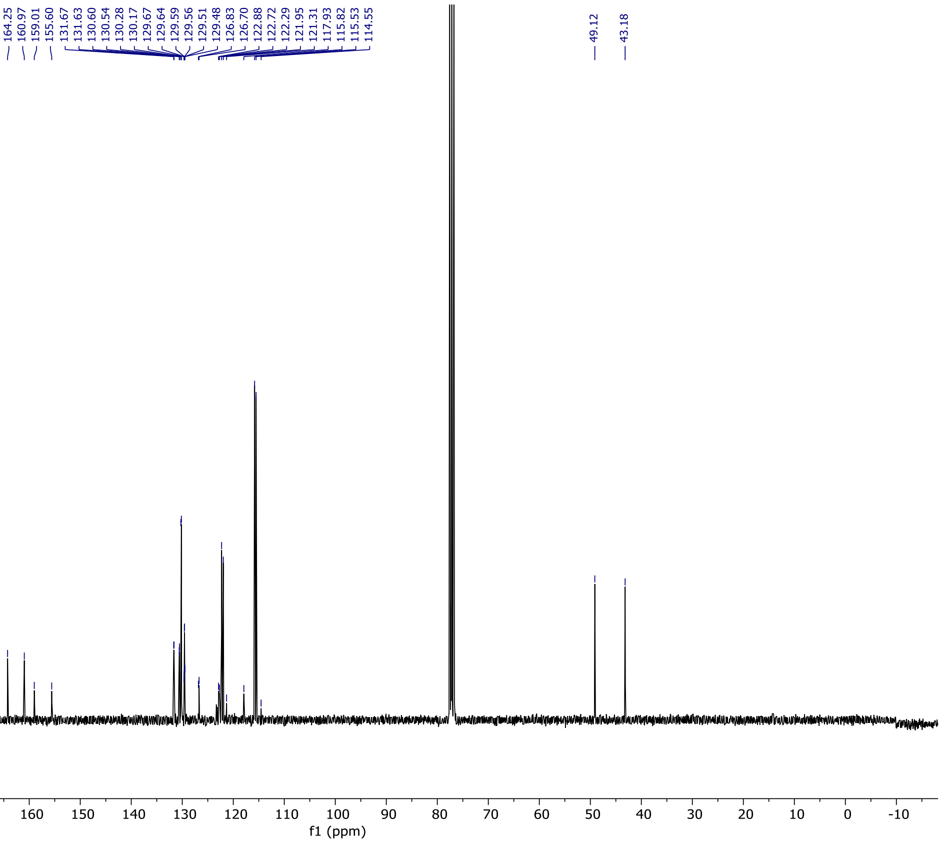

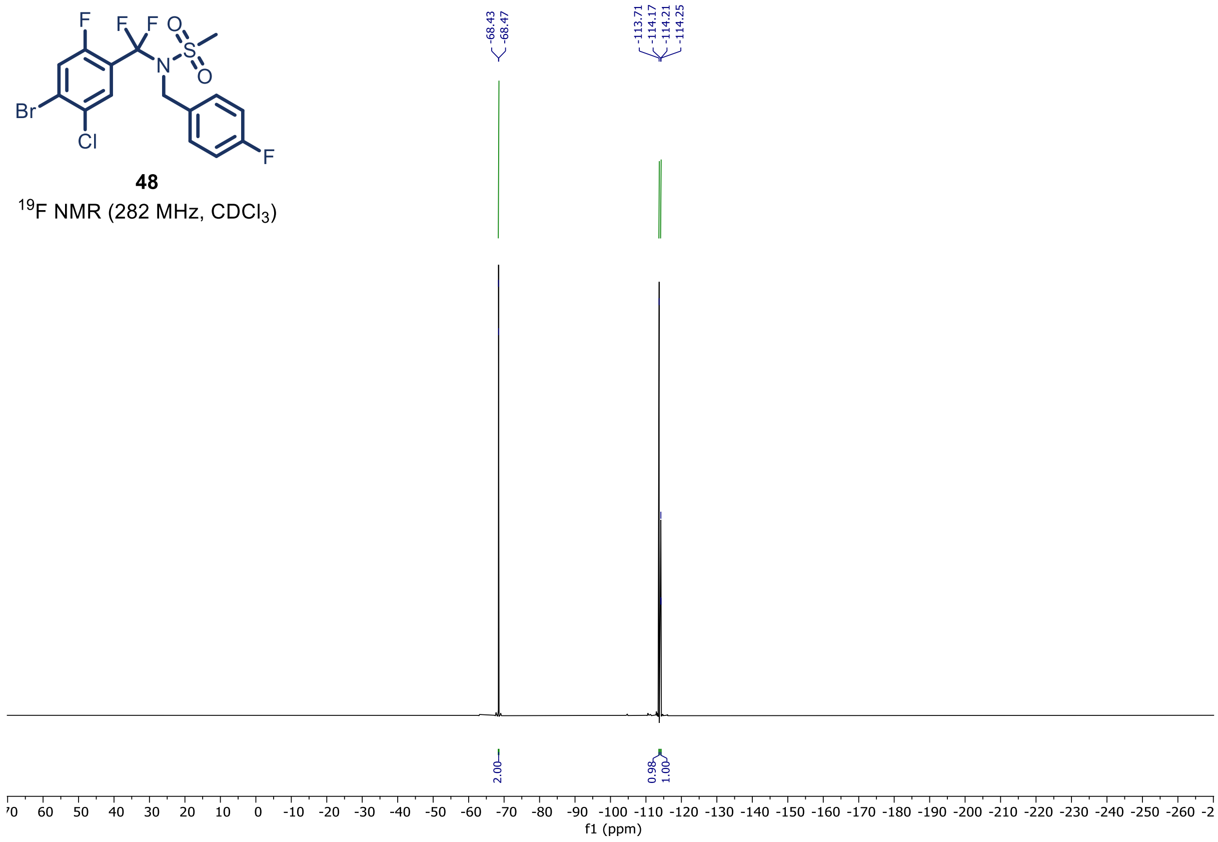

48

^{19}F NMR (282 MHz, $CDCl_3$)

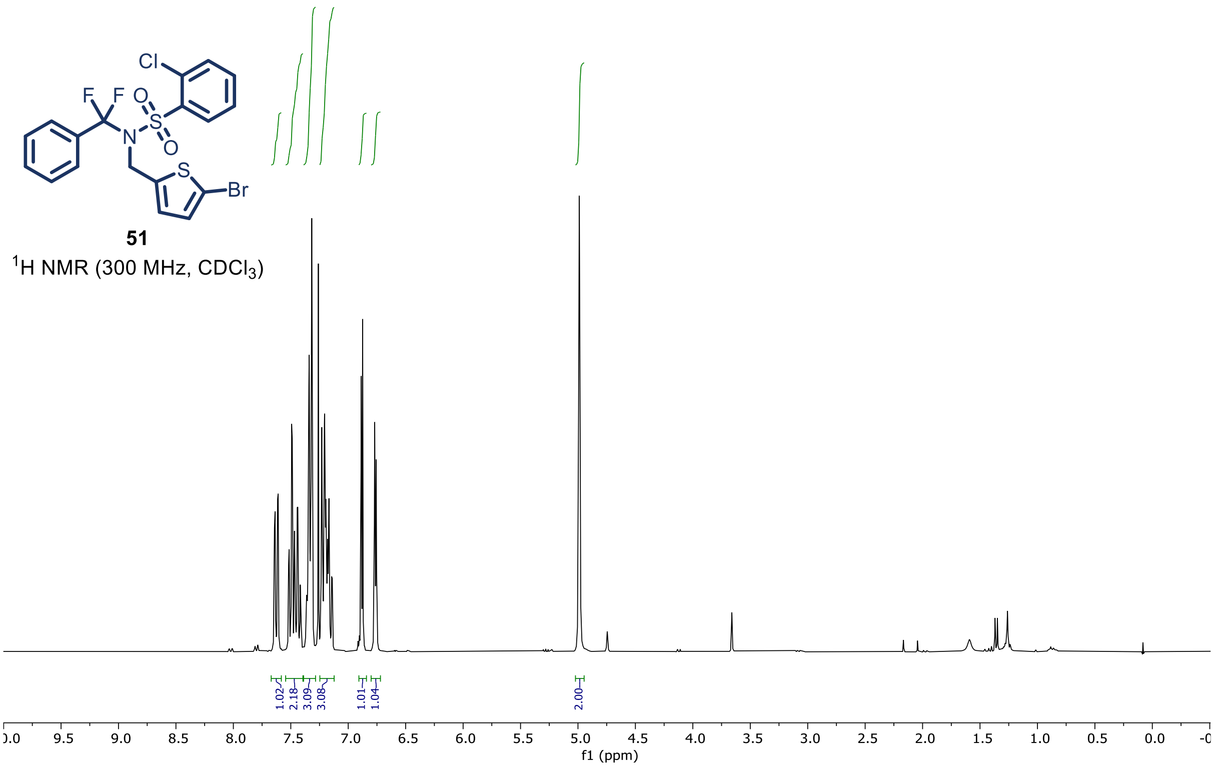
Cl
F F
O
N
S
O
S
Br
51
^{1}H NMR (300 MHz, $CDCl_3$)
1.02
2.18
3.09
3.08
1.01
1.04
2.00
f1 (ppm)

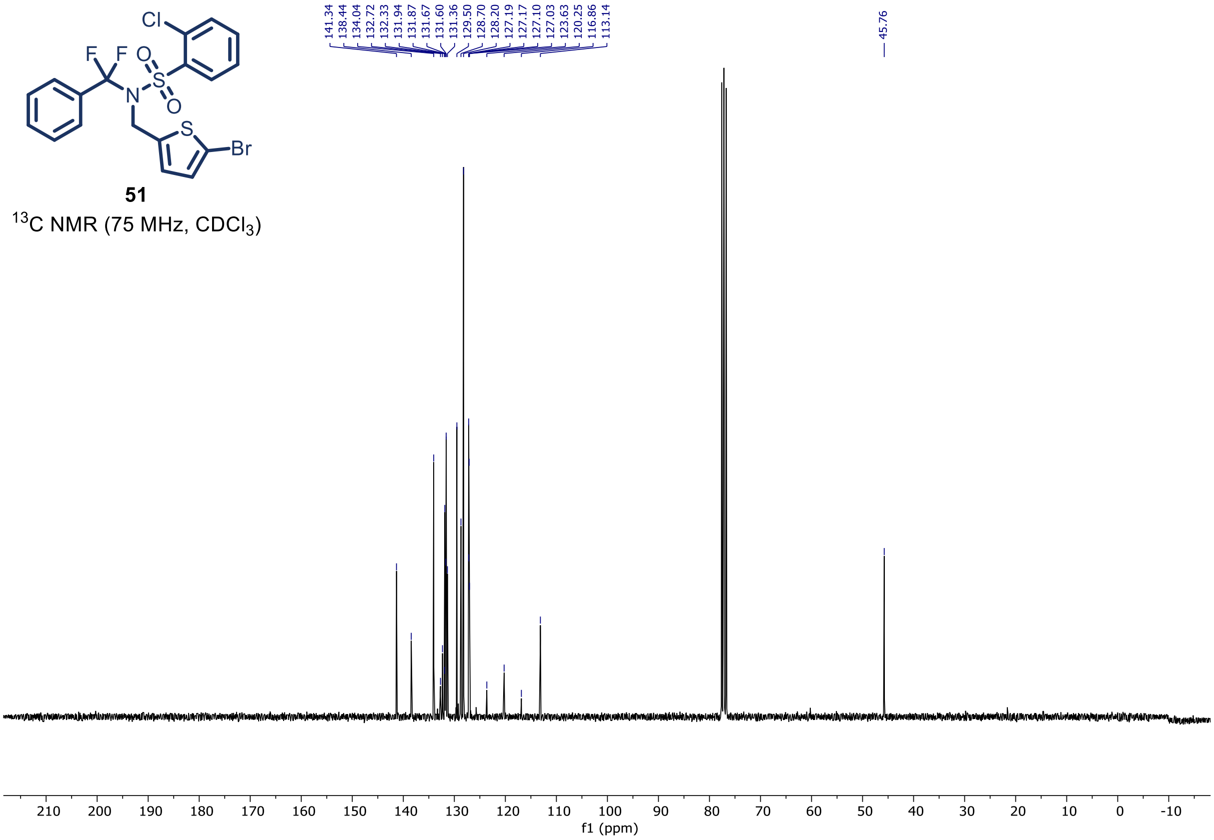
141.34
138.44
134.04
132.72
132.33
131.94
131.87
131.67
131.60
131.36
129.50
128.70
128.20
127.19
127.17
127.10
127.03
123.63
120.25
116.86
113.14
45.76
Cl
F F
O
N
S
O
S
Br
51
^{13}C NMR (75 MHz, $CDCl_3$)
f1 (ppm)

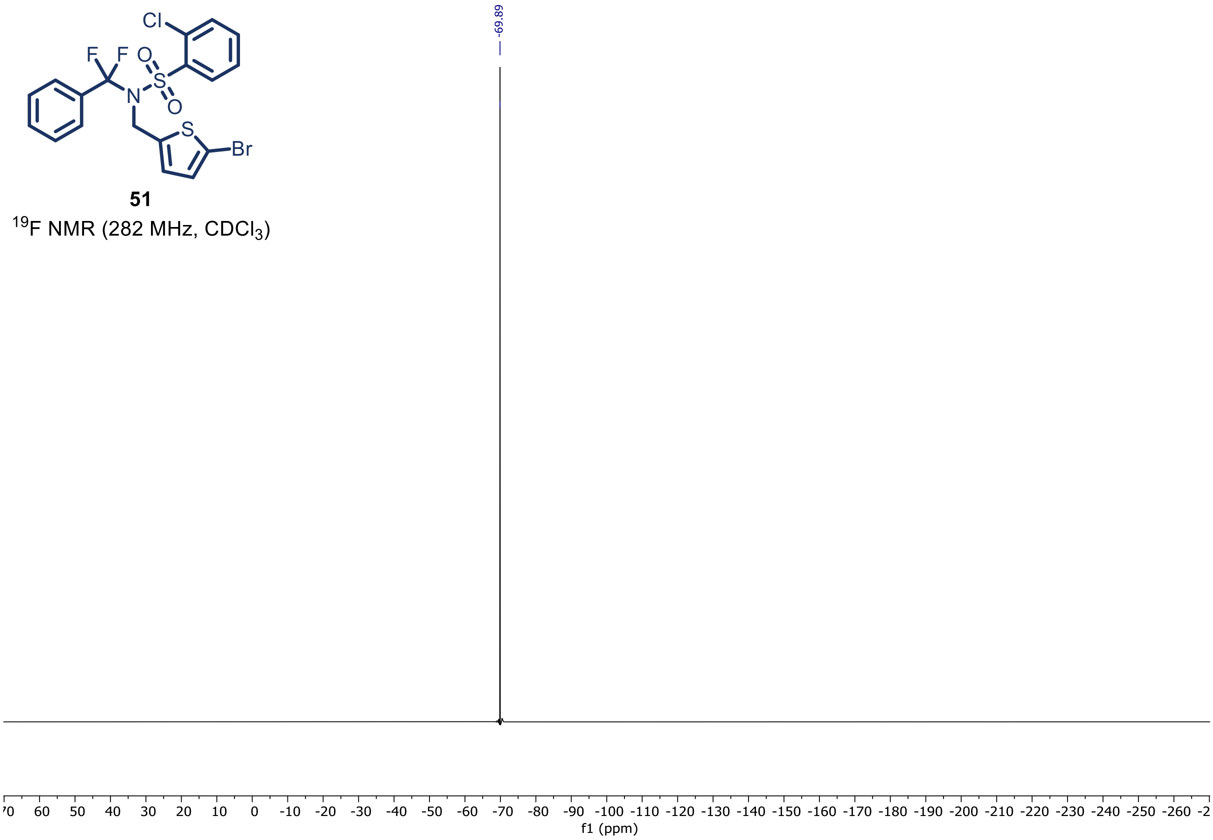

51

^{19}F NMR (282 MHz, $CDCl_3$)

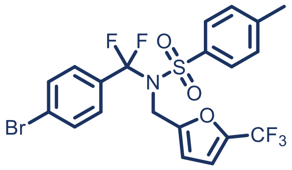

54

^{1}H NMR (300 MHz, $CDCl_3$)

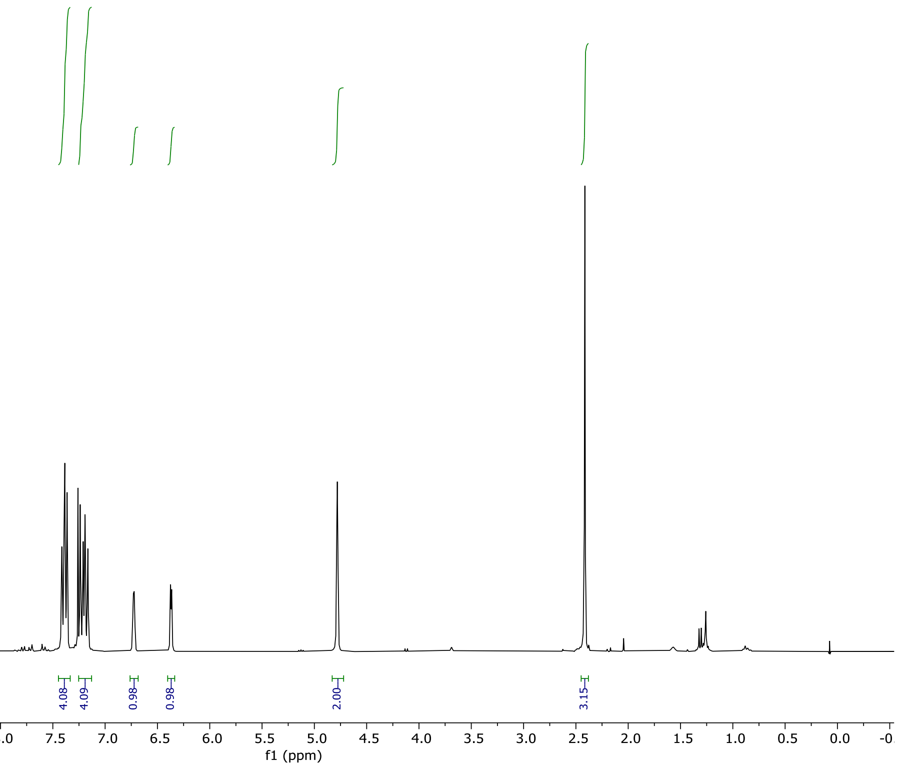

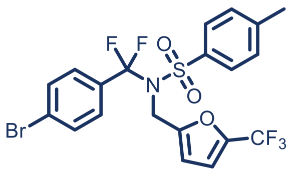

54

^{13}C NMR (75 MHz, $CDCl_3$)

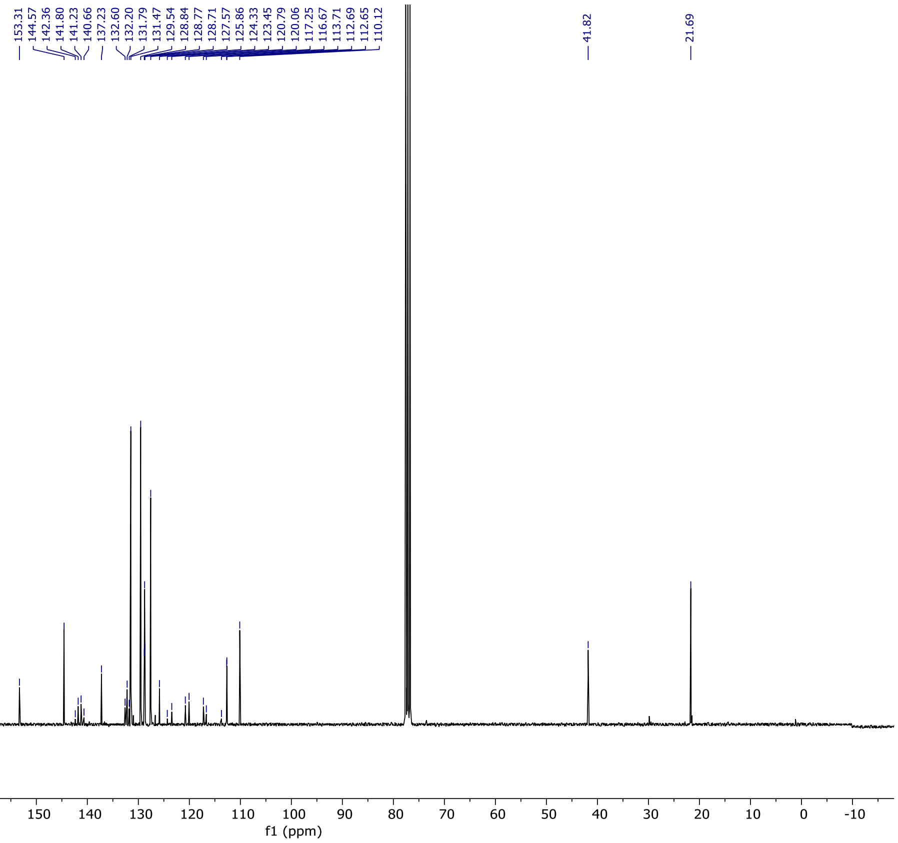

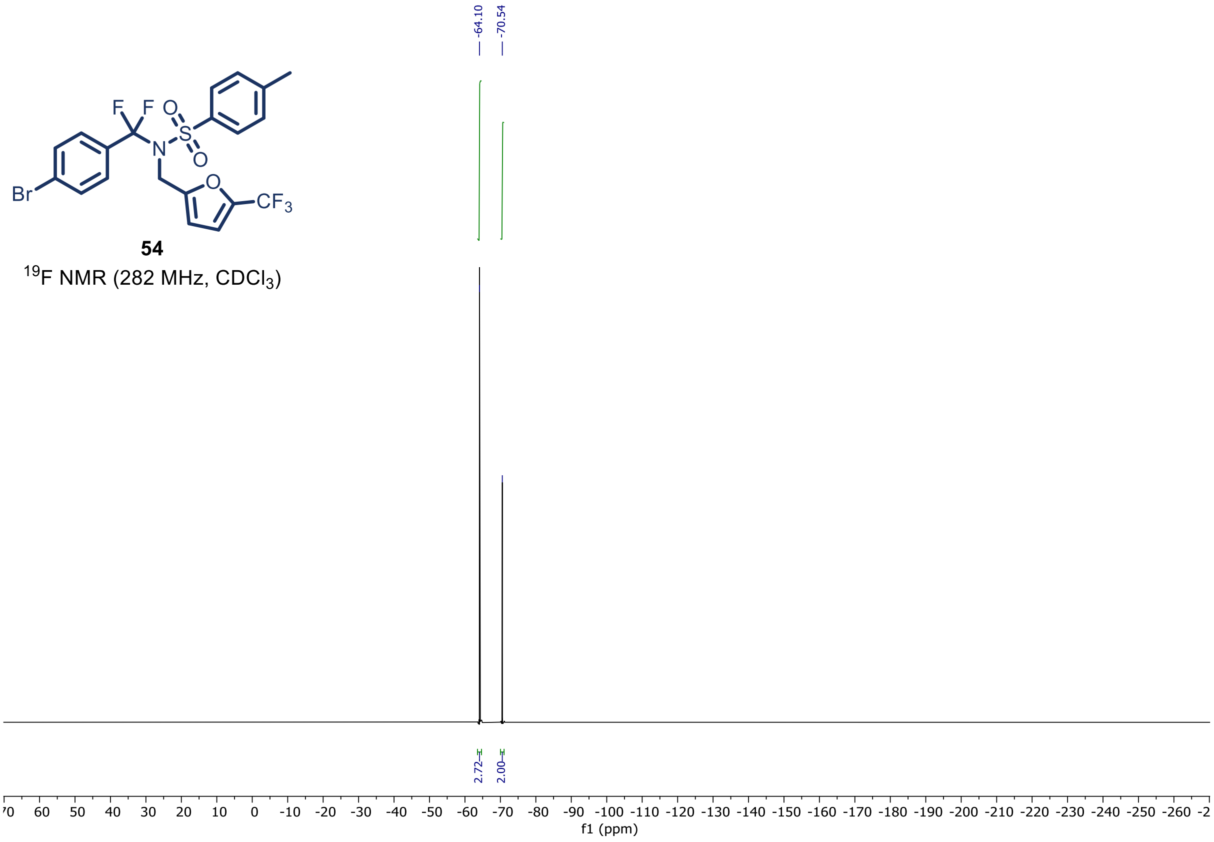

F
F
O
S
O
N
O
Br
CF3
54
19F NMR (282 MHz, CDCl3)
-64.10
-70.54
2.72
2.00
70 60 50 40 30 20 10 0 -10 -20 -30 -40 -50 -60 -70 -80 -90 -100 -110 -120 -130 -140 -150 -160 -170 -180 -190 -200 -210 -220 -230 -240 -250 -260
f1 (ppm)

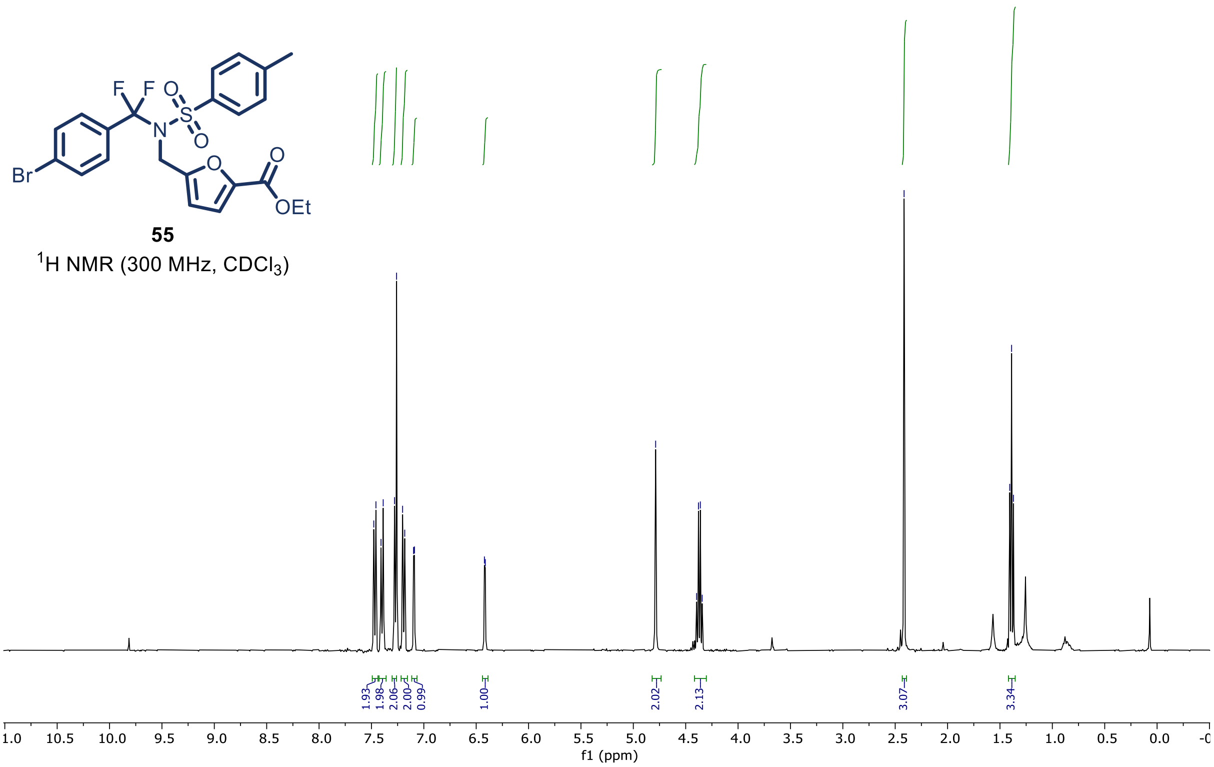

55
^{1}H NMR (300 MHz, CDCl$_3$)
1.93
1.98
2.06
2.00
0.99
1.00
2.02
2.13
3.07
3.34
f1 (ppm)

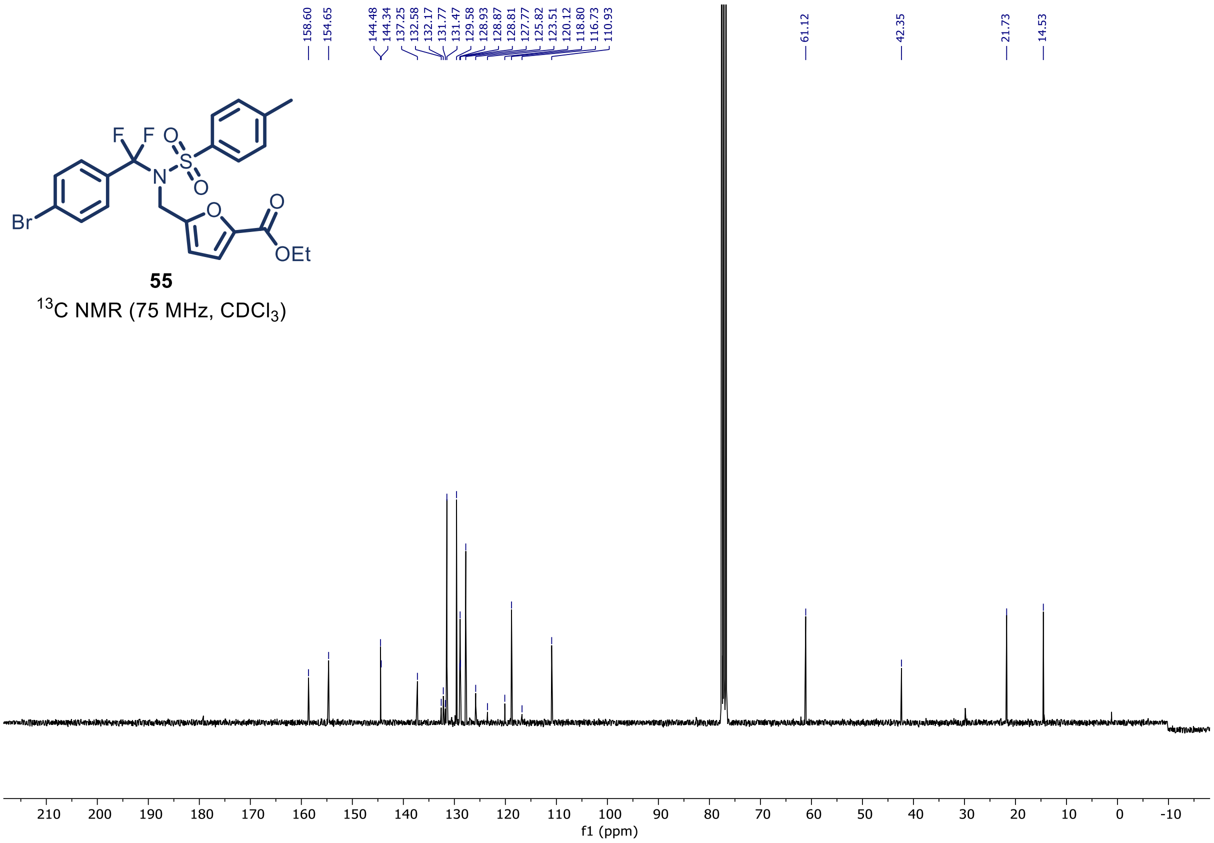

158.60
154.65
144.48
144.34
137.25
132.58
132.17
131.77
131.47
129.58
128.93
128.87
128.81
127.77
125.82
123.51
120.12
118.80
116.73
110.93
61.12
42.35
21.73
14.53
55
^{13}C NMR (75 MHz, CDCl$_3$)
f1 (ppm)

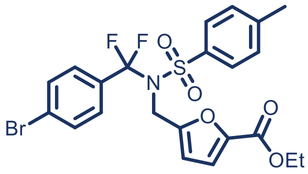

55

^{19}F NMR (282 MHz, $CDCl_3$)

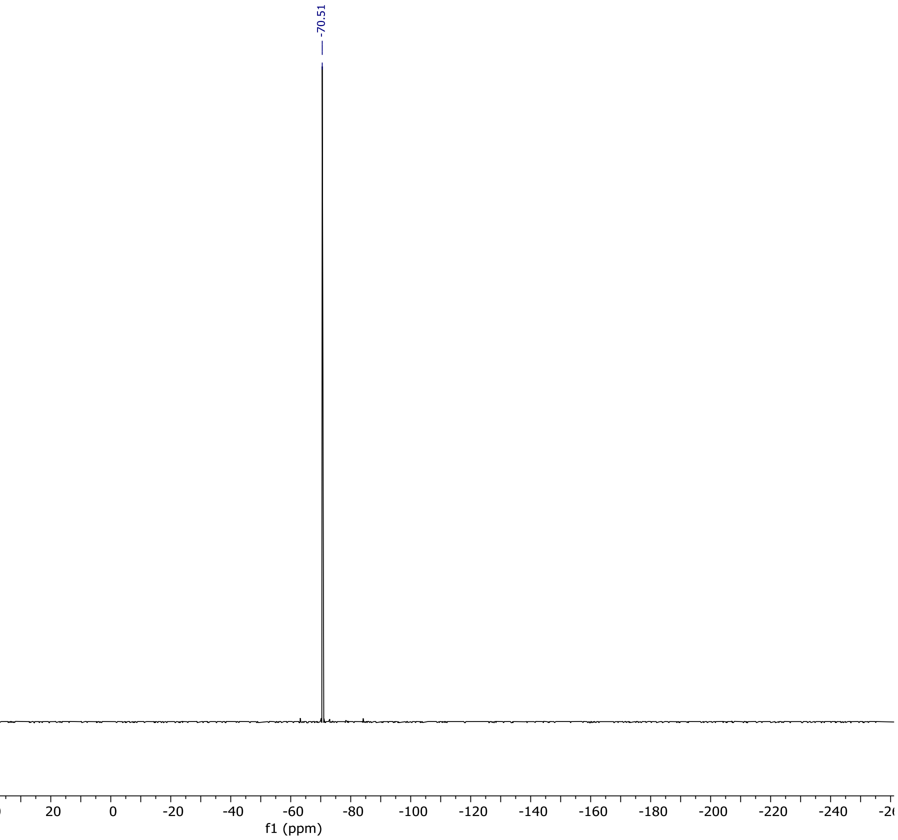

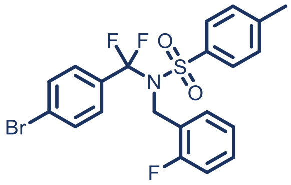

56

^{1}H NMR (300 MHz, $CDCl_3$)

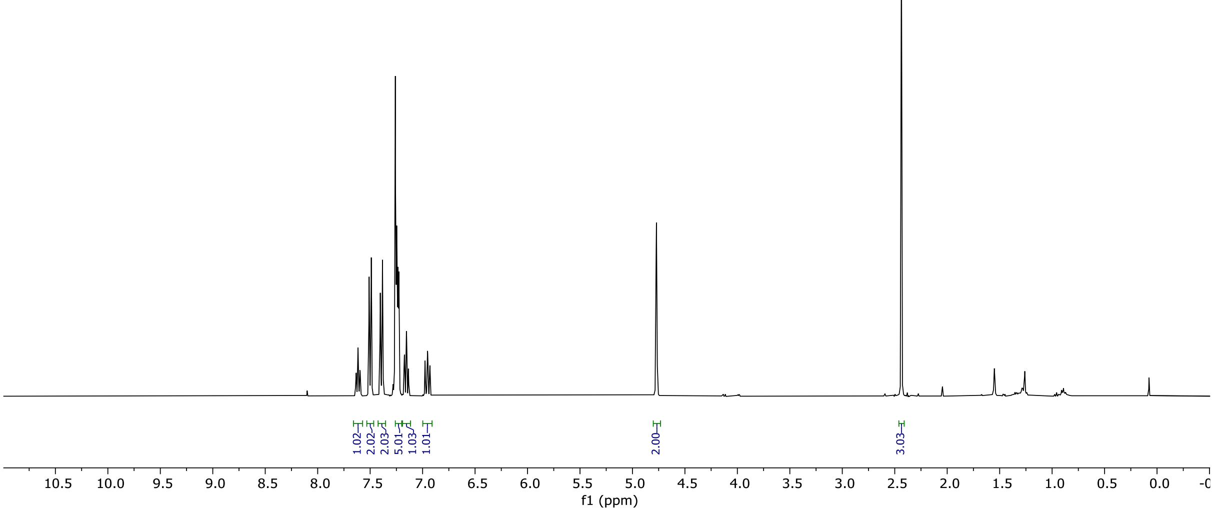

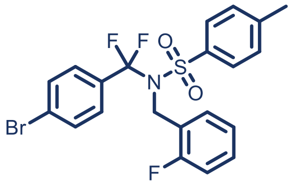

56

^{13}C NMR (75 MHz, $CDCl_3$)

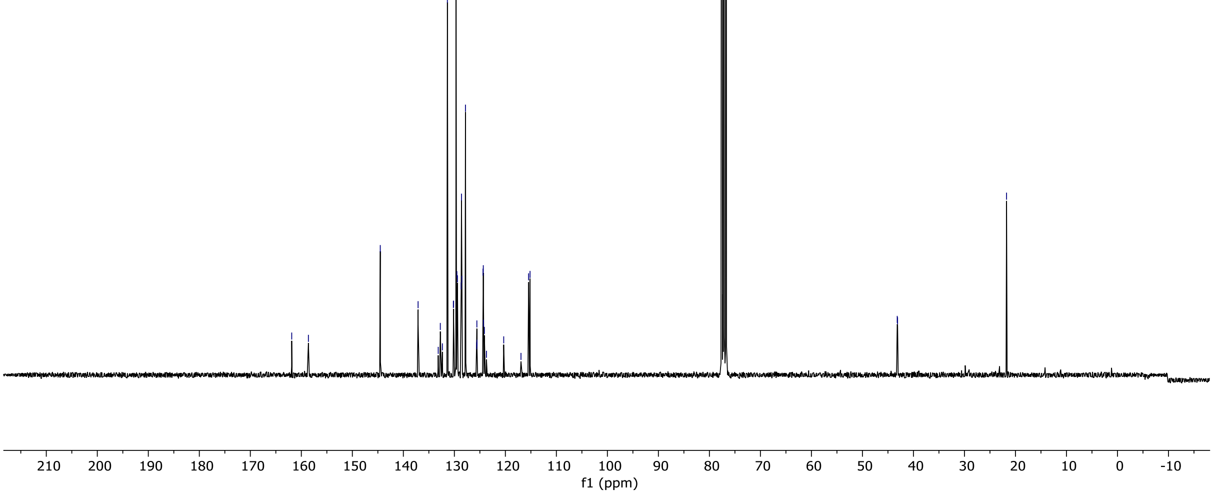

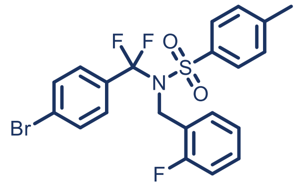

56

^{19}F NMR (282 MHz, $CDCl_3$)

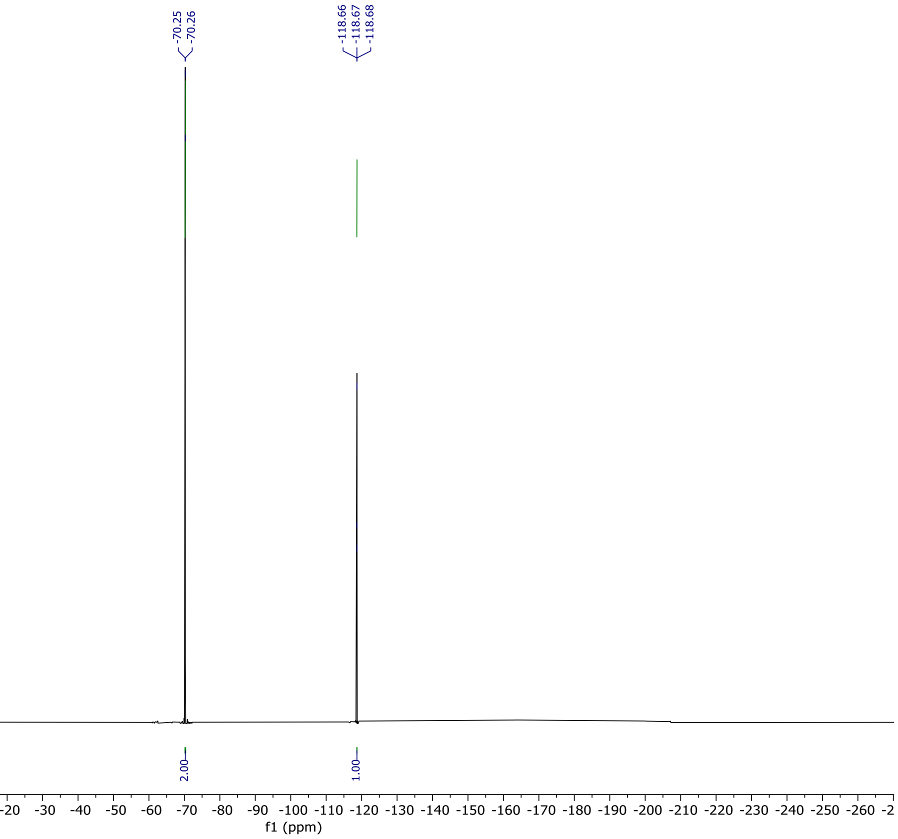

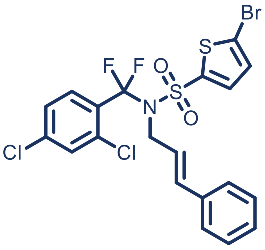

61

^{1}H NMR (300 MHz, $CDCl_3$)

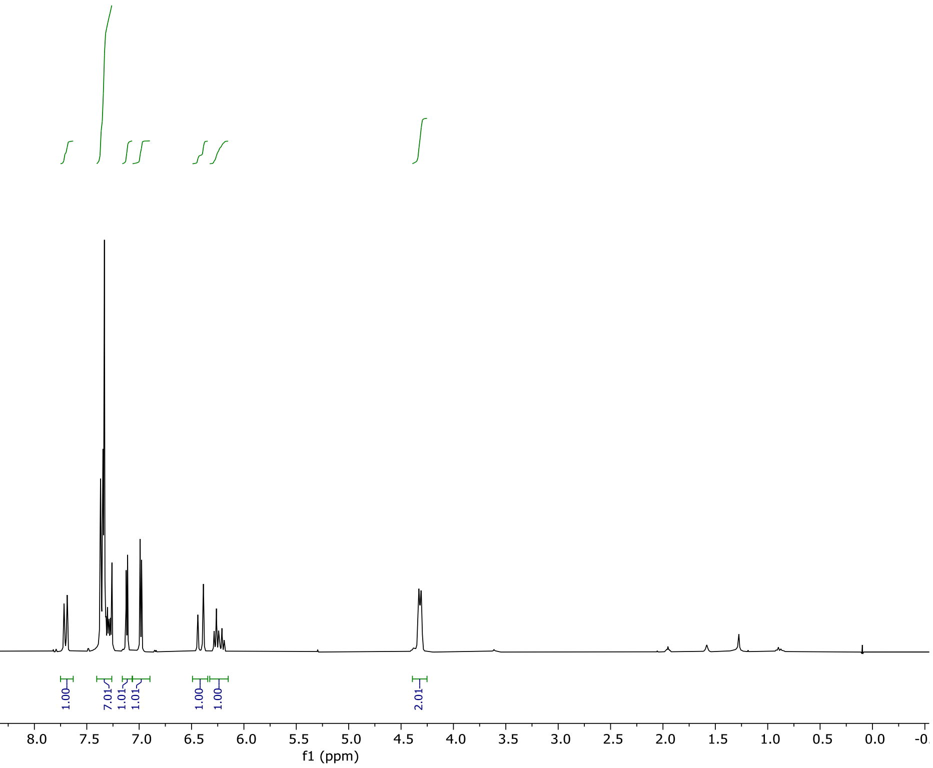

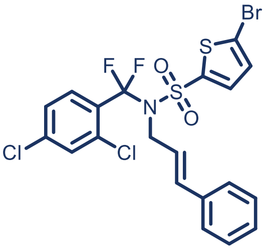

61

^{13}C NMR (75 MHz, $CDCl_3$)

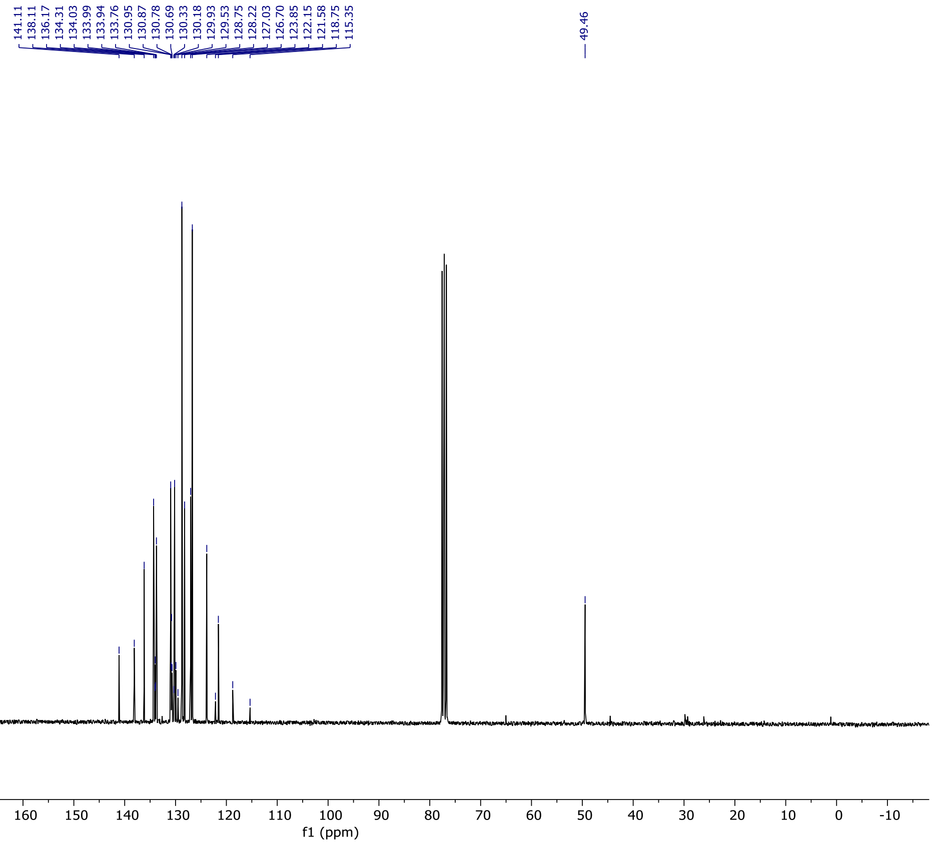

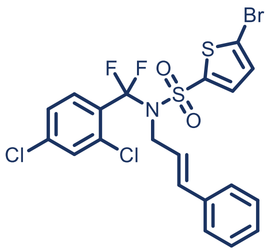

61

^{19}F NMR (282 MHz, $CDCl_3$)

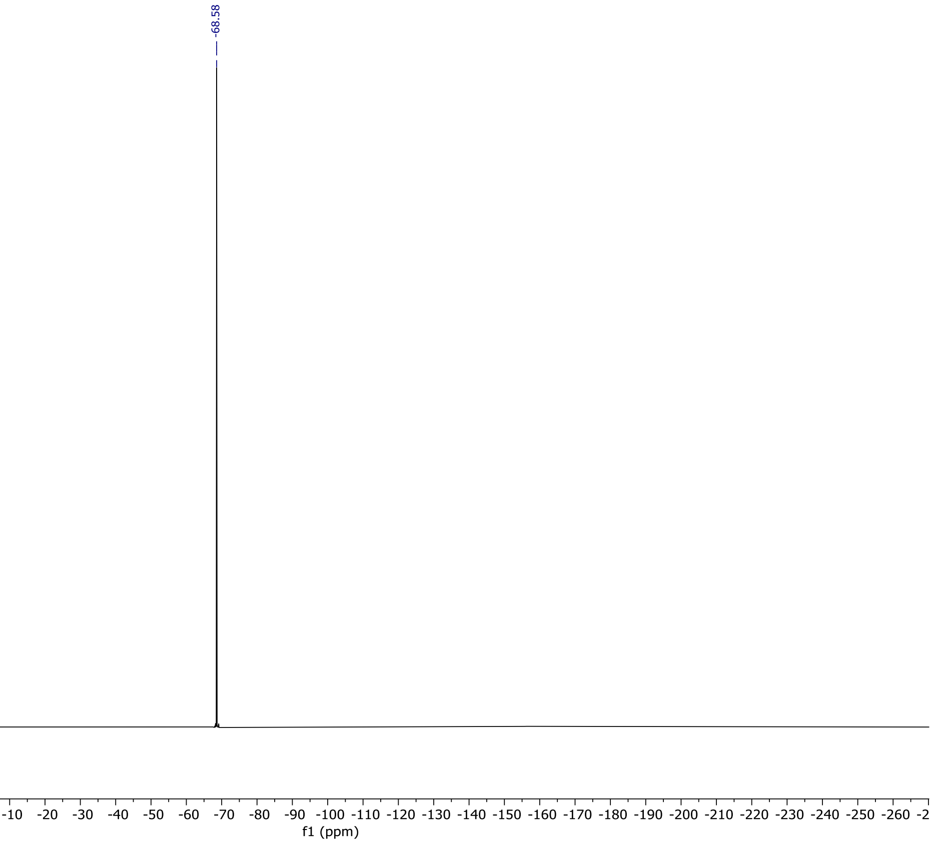

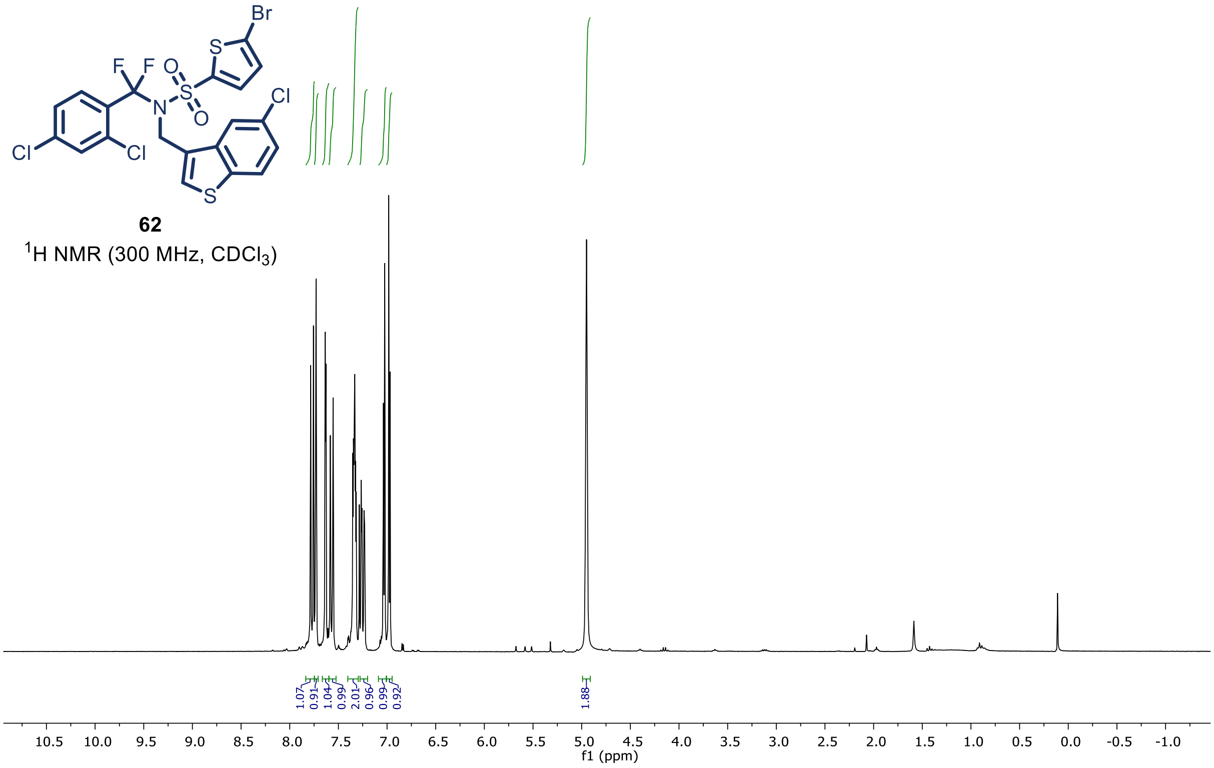

62

^{1}H NMR (300 MHz, $CDCl_3$)

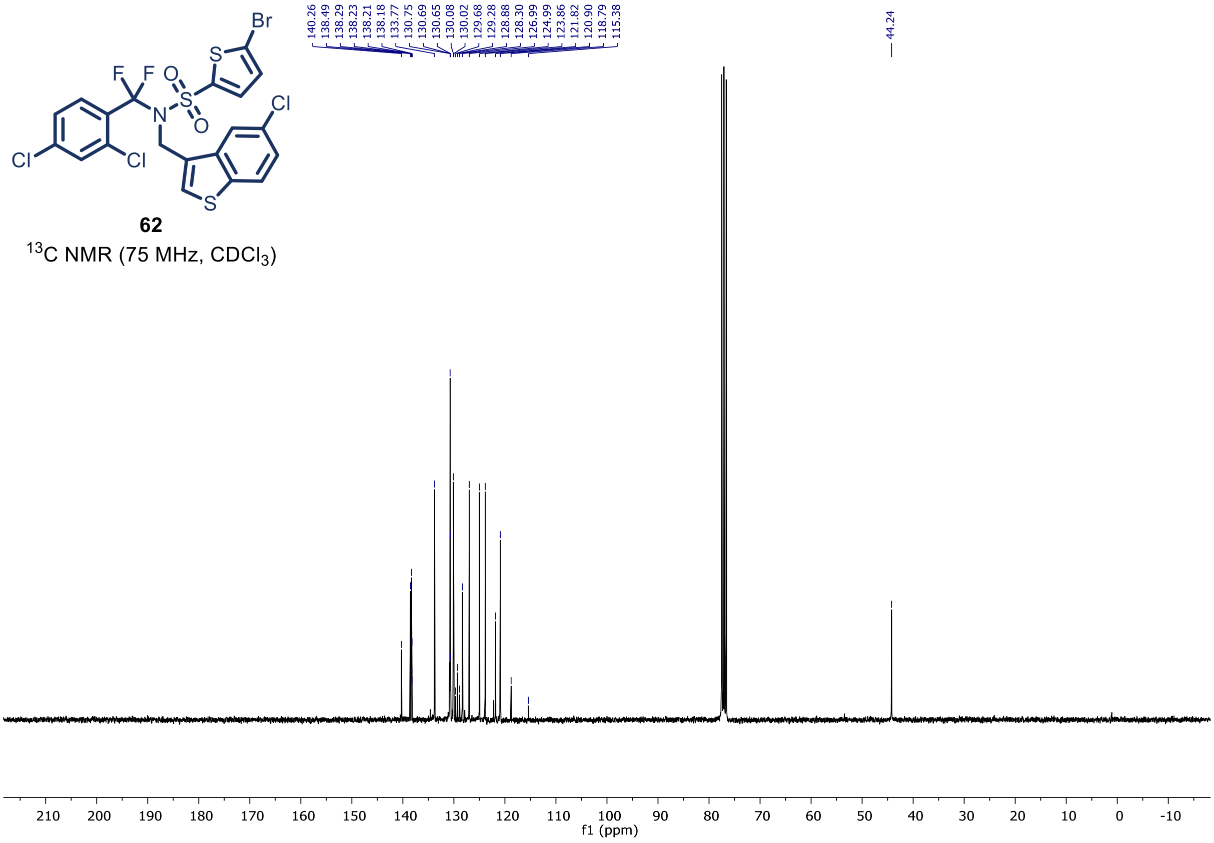

62

^{13}C NMR (75 MHz, $CDCl_3$)

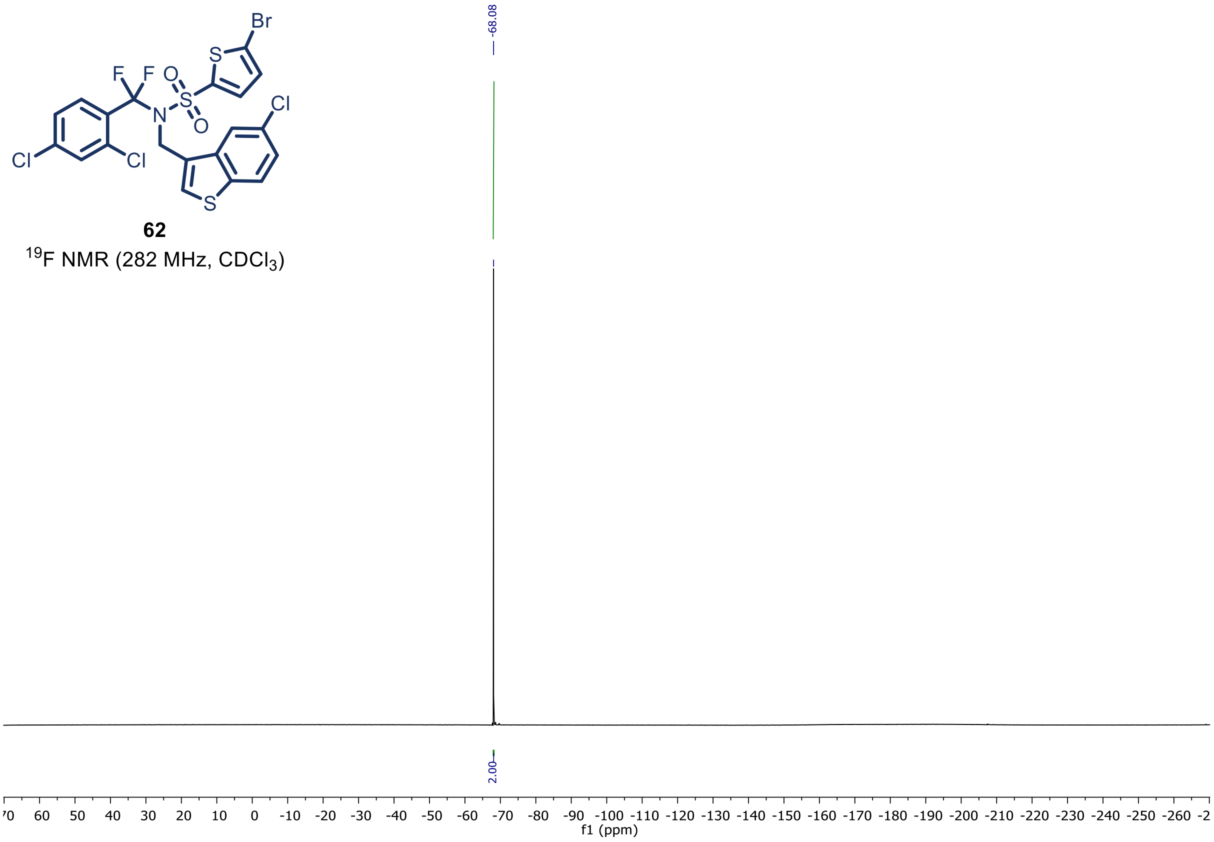

62

^{19}F NMR (282 MHz, $CDCl_3$)

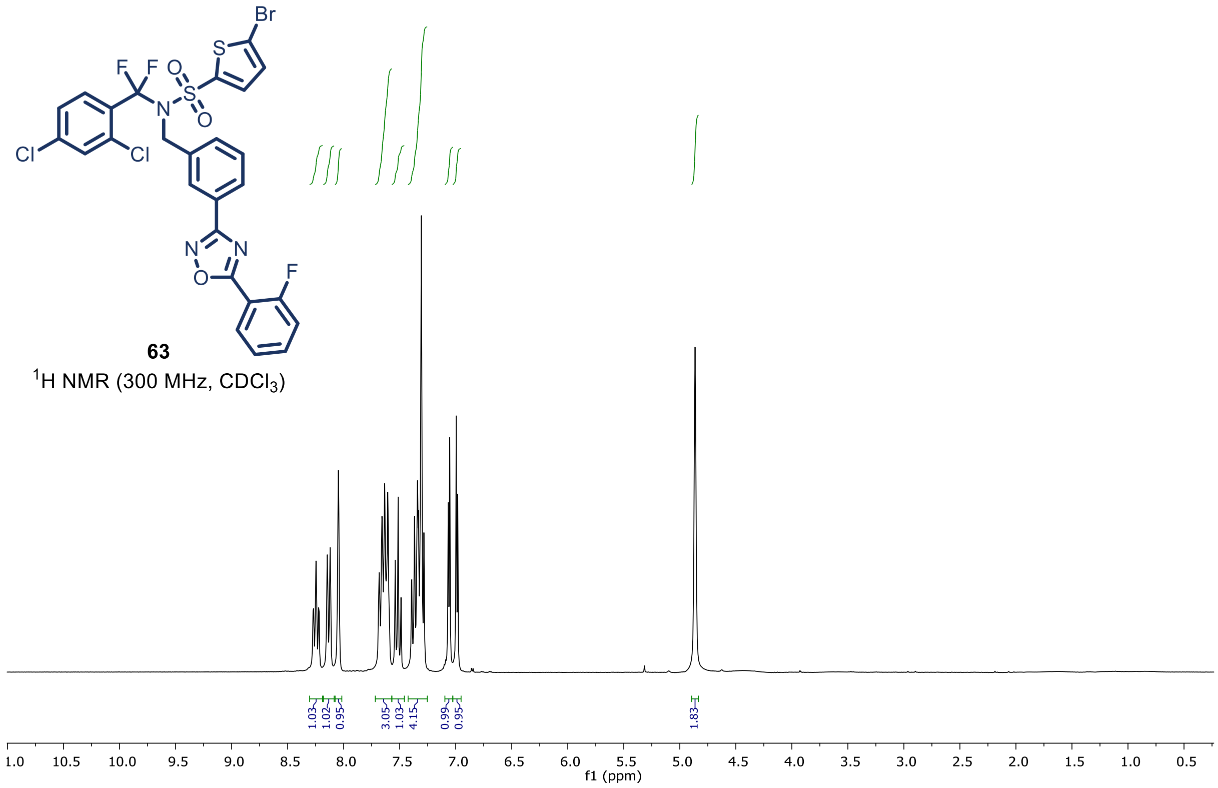
Br
S
F F O
N
S
O
Cl
Cl
N
N
O
F
63
^{1}H NMR (300 MHz, $CDCl_3$)
1.03
1.02
0.95
3.05
1.03
4.15
0.99
0.95
1.83
1.0 10.5 10.0 9.5 9.0 8.5 8.0 7.5 7.0 6.5 6.0 5.5 5.0 4.5 4.0 3.5 3.0 2.5 2.0 1.5 1.0 0.5
f1 (ppm)

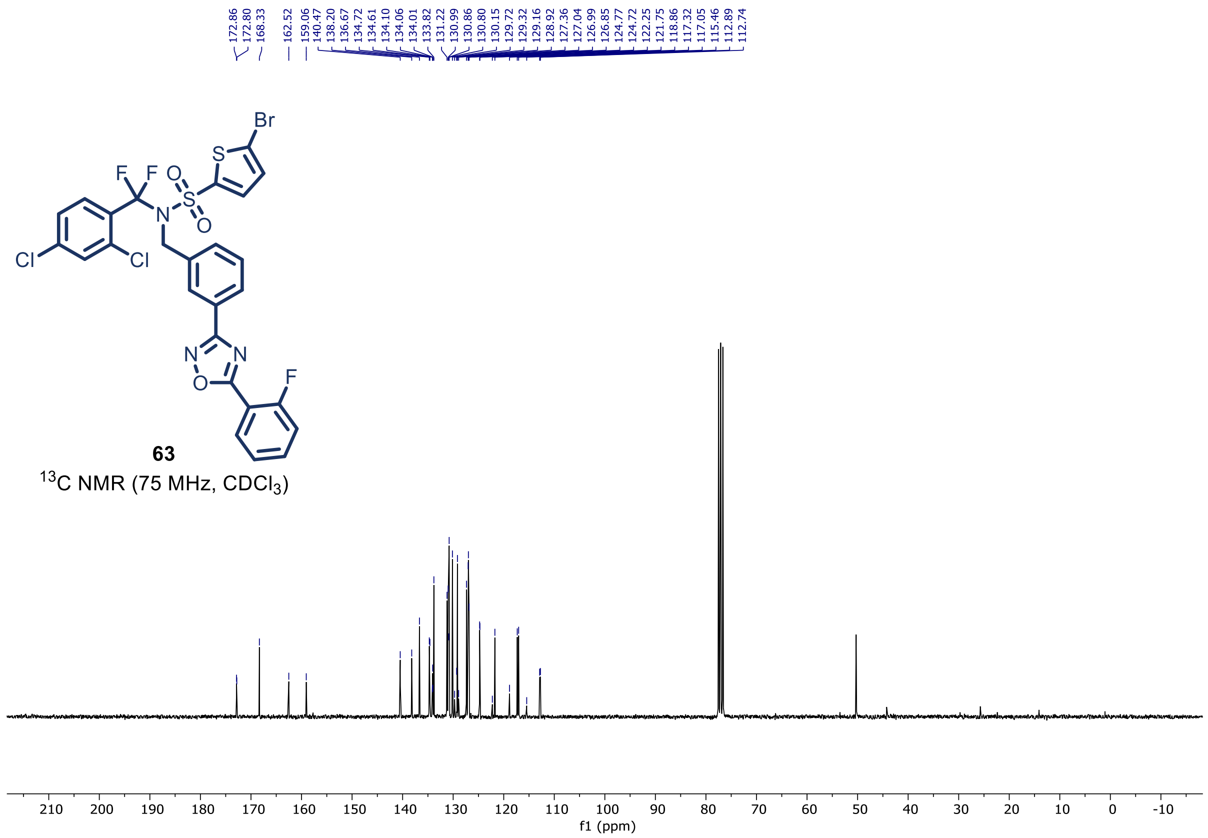
172.86
172.80
168.33
162.52
159.06
140.47
138.20
136.67
134.72
134.61
134.10
134.06
134.01
133.82
131.22
130.99
130.86
130.80
130.15
129.72
129.32
129.16
128.92
127.36
127.04
126.99
126.85
124.77
124.72
122.25
121.75
118.86
117.32
117.05
115.46
112.89
112.74
Br
S
F F O
N
S
O
Cl
Cl
N
N
O
F
63
^{13}C NMR (75 MHz, $CDCl_3$)
210 200 190 180 170 160 150 140 130 120 110 100 90 80 70 60 50 40 30 20 10 0 -10
f1 (ppm)

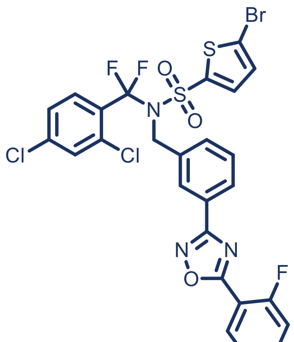

63

^{19}F NMR (282 MHz, $CDCl_3$)

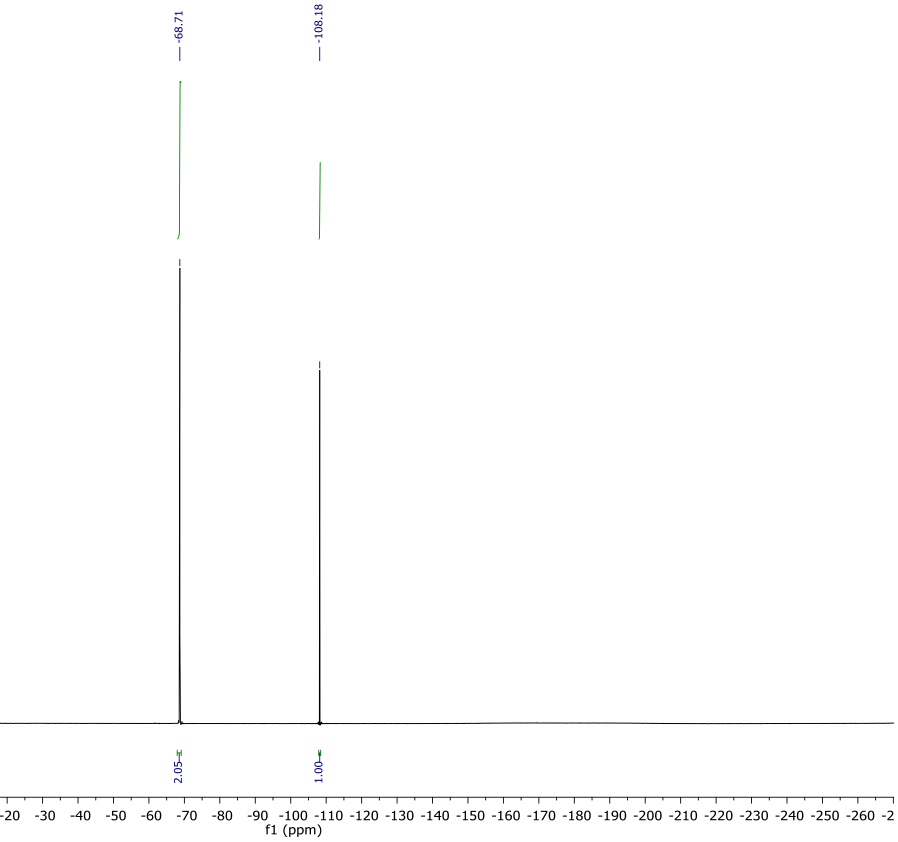

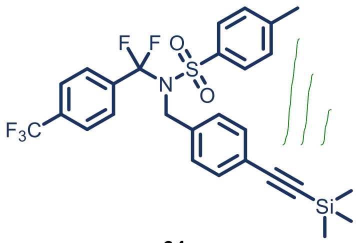

64

^{1}H NMR (300 MHz, $CDCl_3$)

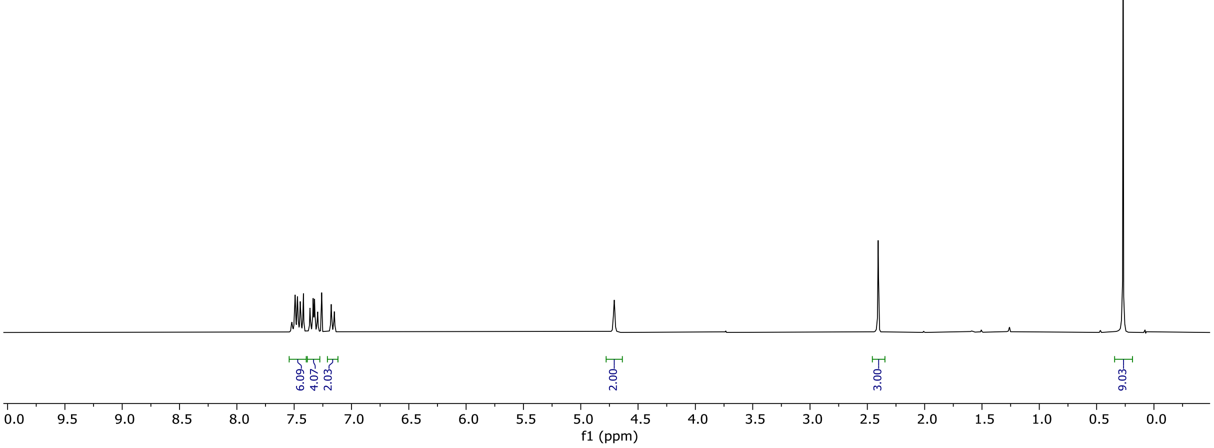

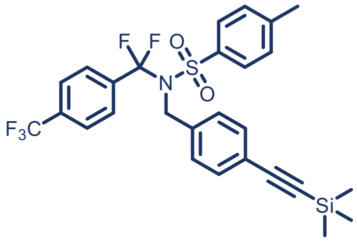

64

^{13}C NMR (75 MHz, $CDCl_3$)

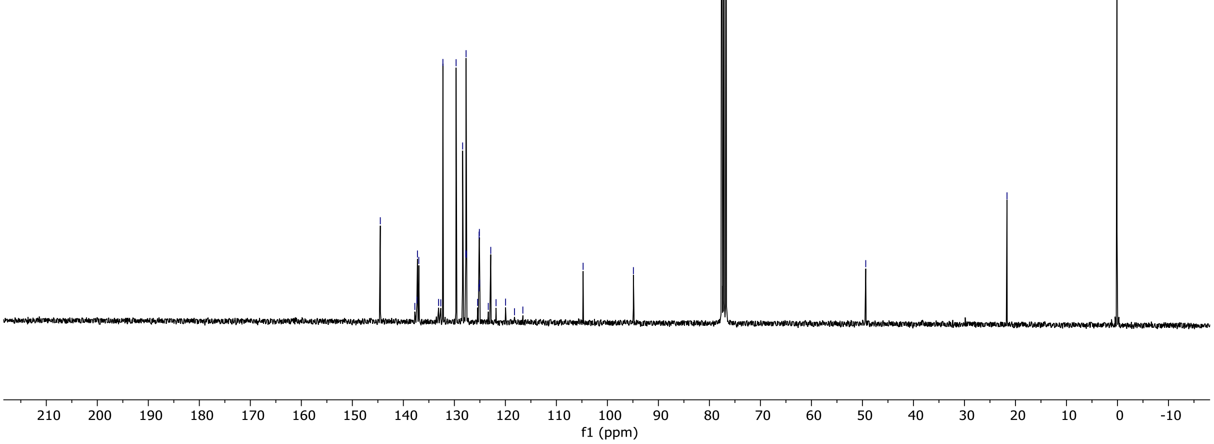

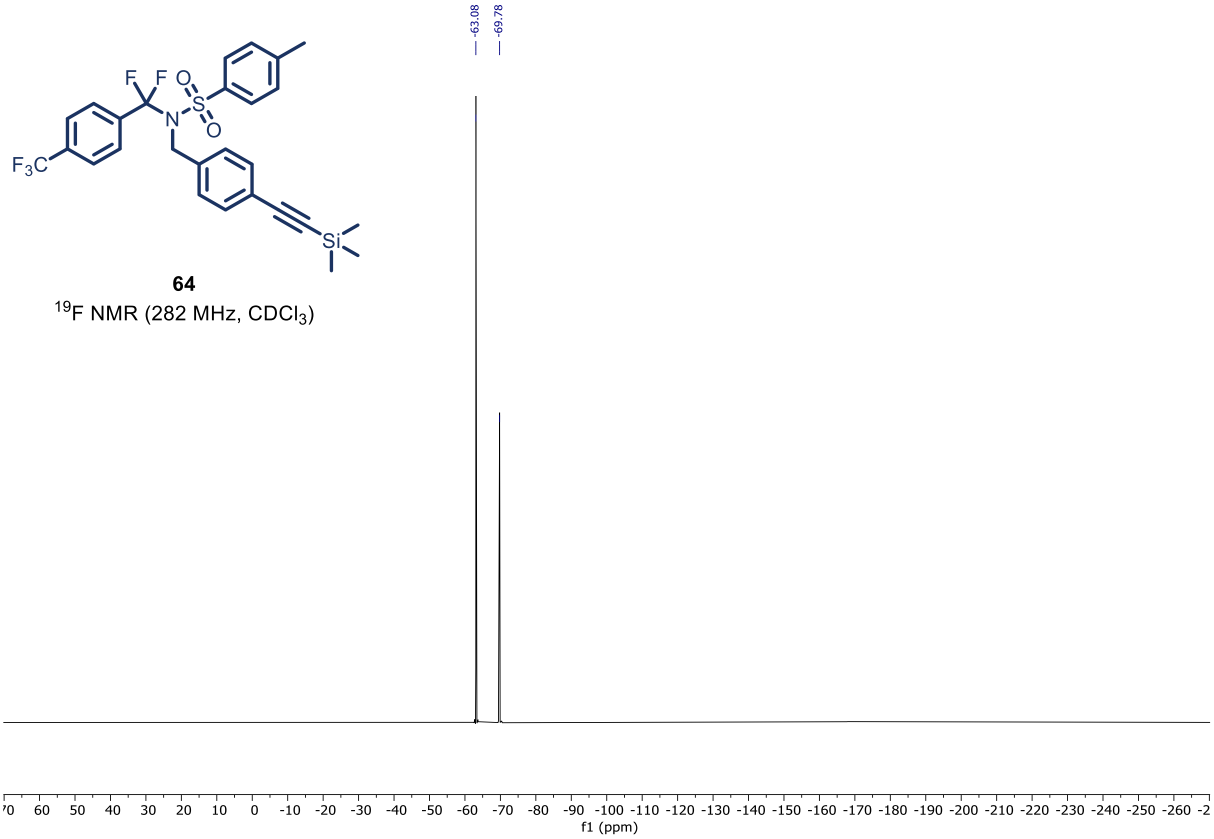

64

^{19}F NMR (282 MHz, $CDCl_3$)

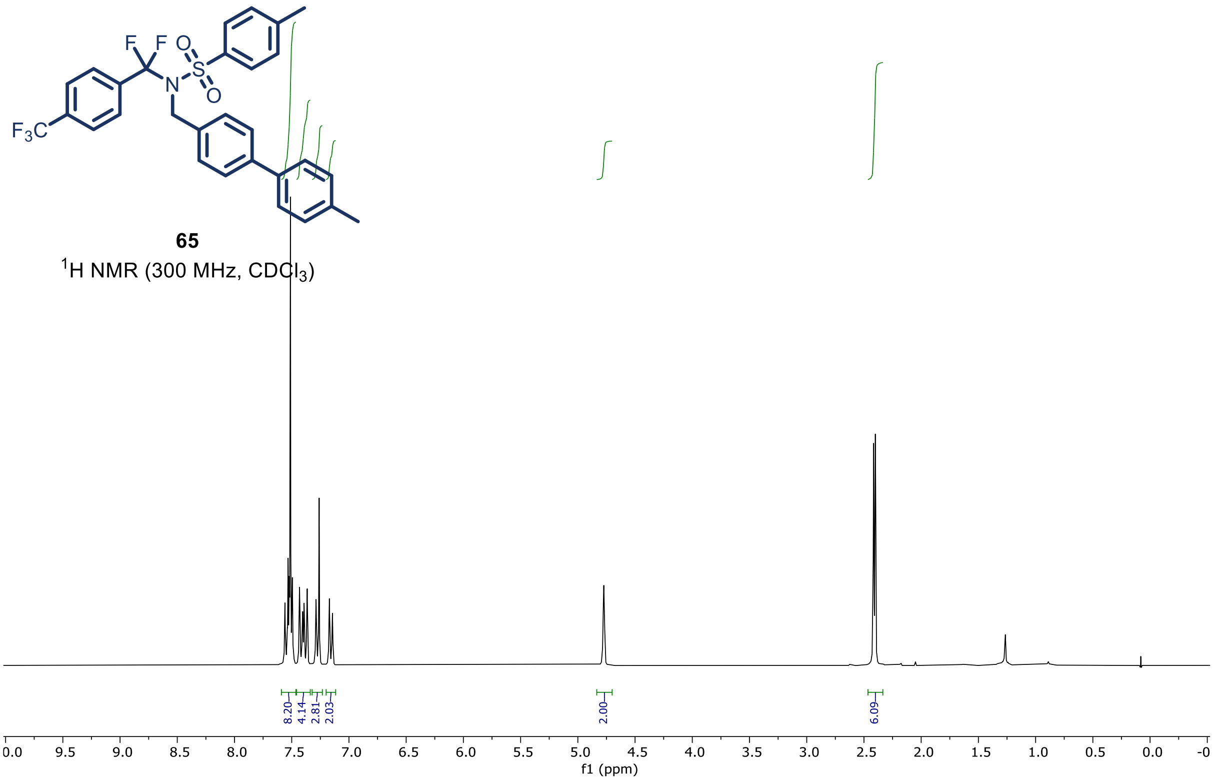

65

^1H NMR (300 MHz, $CDCl_3$)

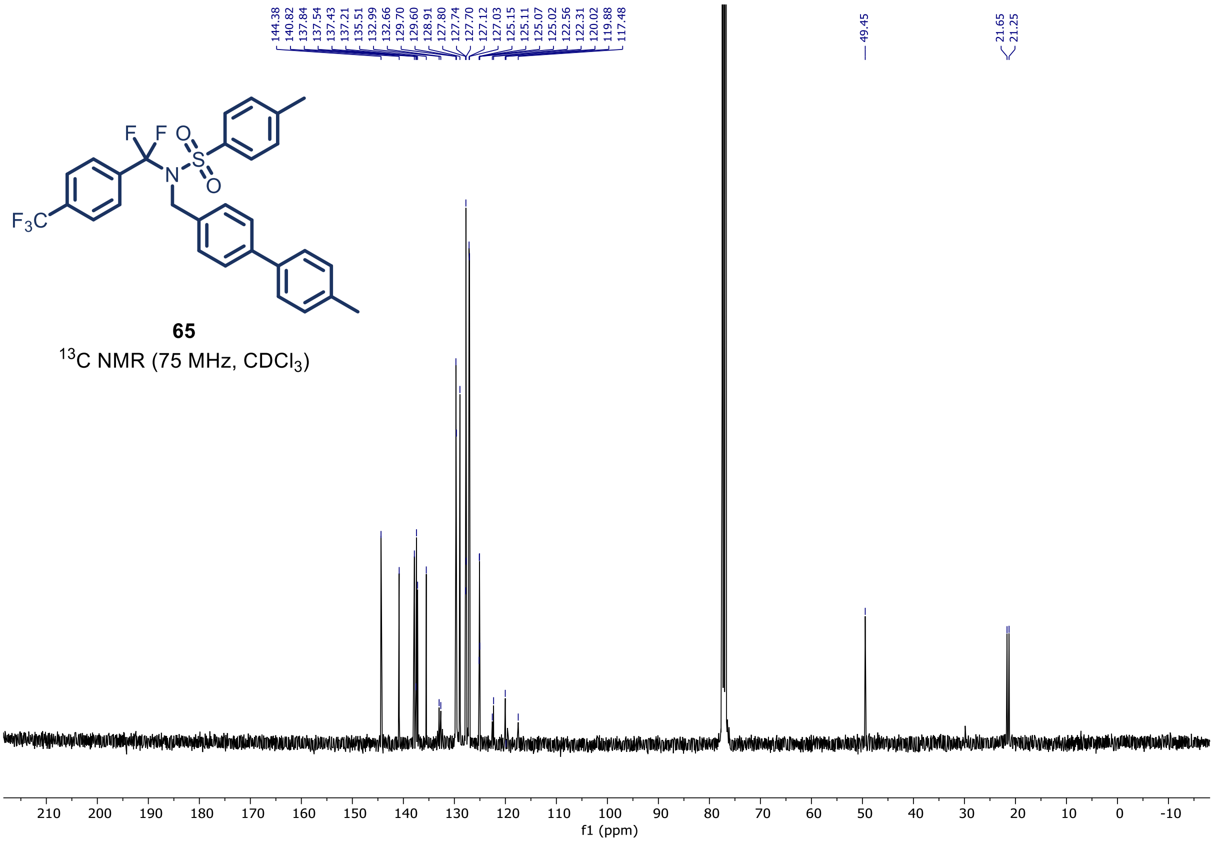

65

^{13}C NMR (75 MHz, $CDCl_3$)

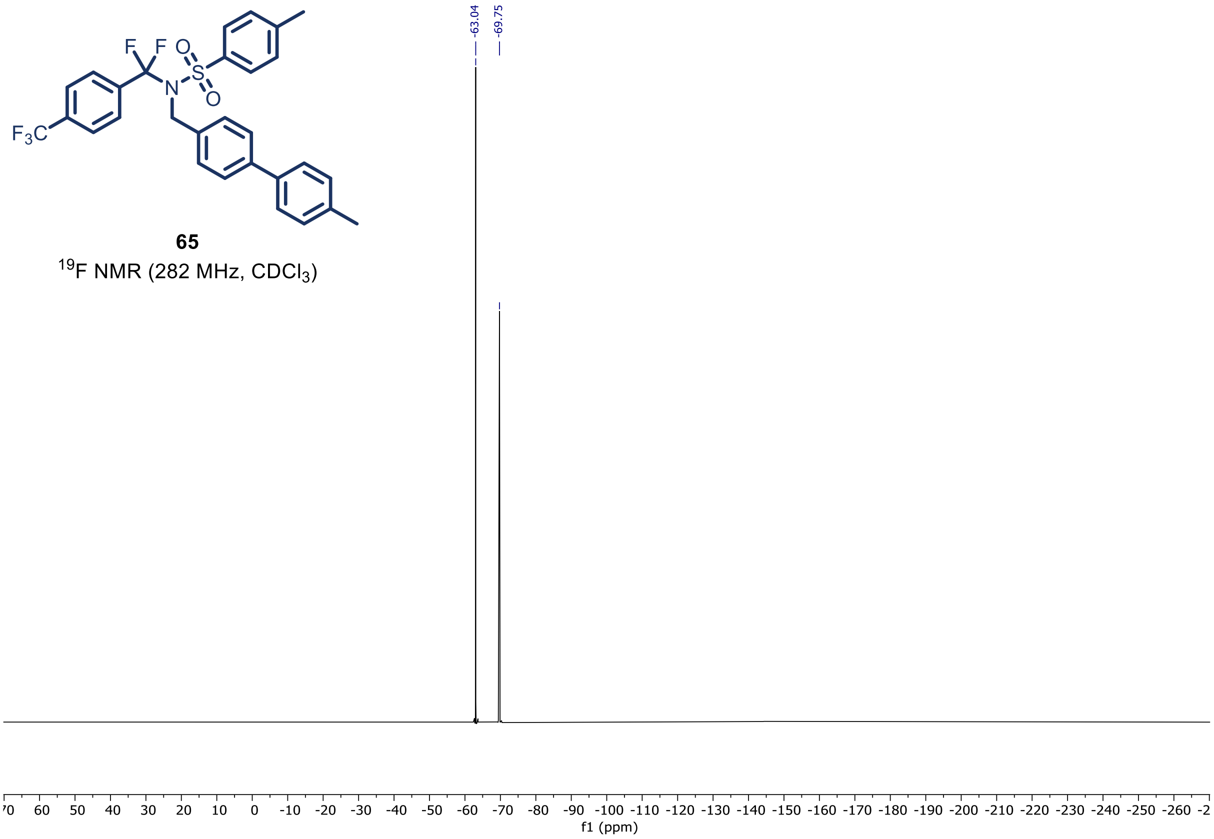

65

^{19}F NMR (282 MHz, $CDCl_3$)

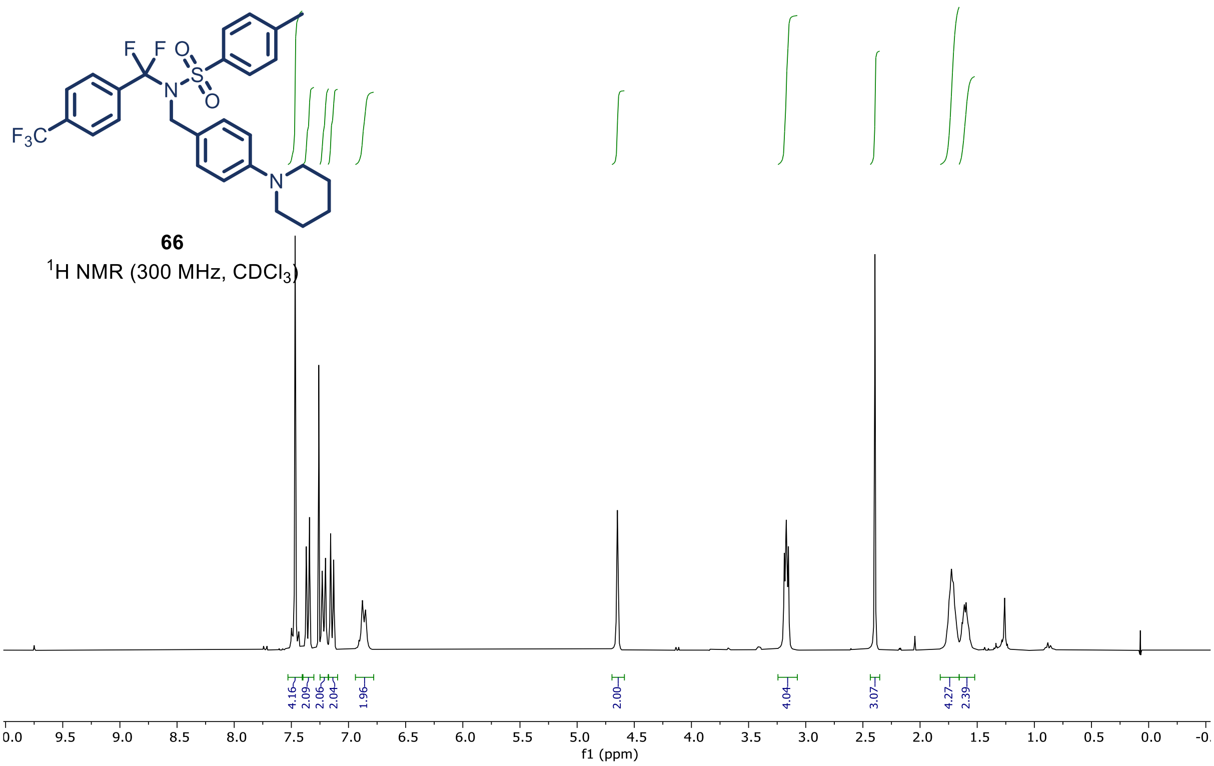

66

^{1}H NMR (300 MHz, $CDCl_3$)

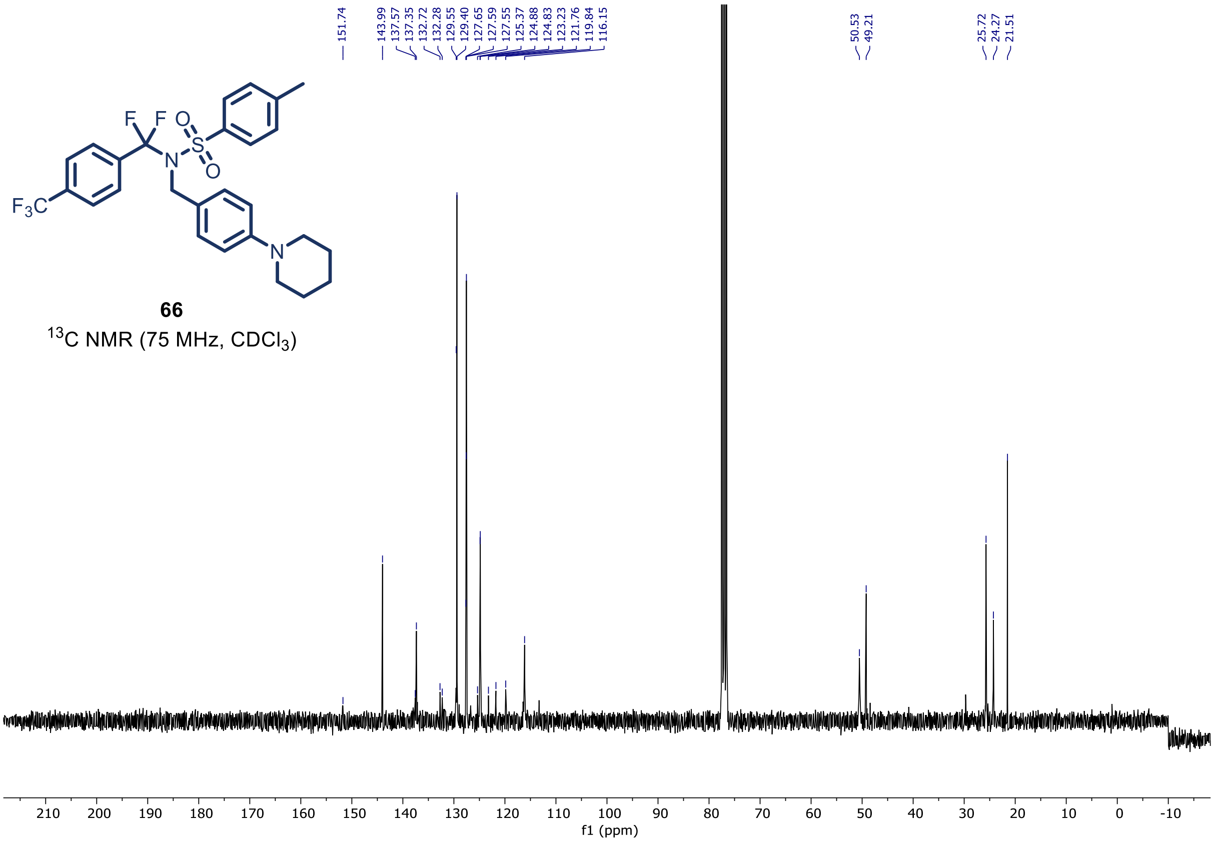

66

^{13}C NMR (75 MHz, $CDCl_3$)

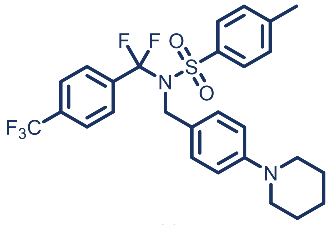

66

^{19}F NMR (282 MHz, $CDCl_3$)

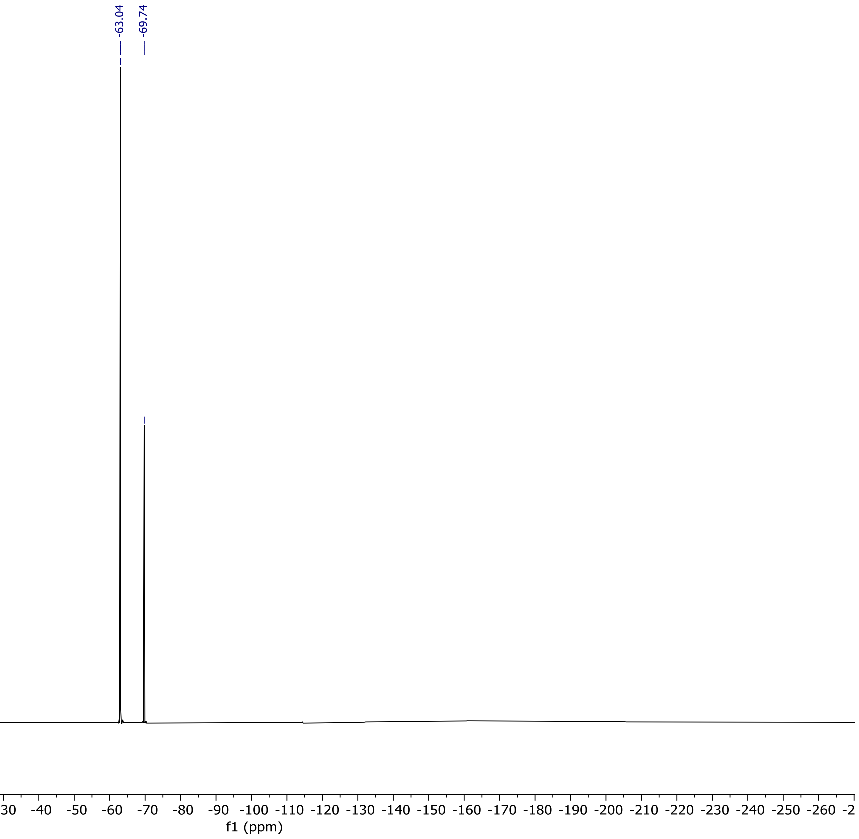

6. References

1. Huang, X., et al. Asymmetric synthesis of difluorinated α-quaternary amino acids (DFAAs) via Cu-catalyzed difluorobenzylation of aldimine esters. *Chin. Chem. Lett.* **2024**, *35* (12), 109665.
2. Pilli, R.; Selvam, K.; Balamurugan, B. S. S.; Jose, V.; Rasappan, R. C(sp3)–C(sp3) Coupling of Cycloalkanes and Alkyl Halides via Dual Photocatalytic Hydrogen Atom Transfer and Nickel Catalysis. *Org. Lett.* **2024**, *26* (15), 2993-2998.
3. Yu, K.; Nie, Q.; Chen, Q.; Liu, W. Manganese-catalyzed cyclopropanation of allylic alcohols with sulfones. *Nat. Commun.* **2024**, *15* (1), 6798.
4. Zhang, X., et al. Bicyclic compound for the treatment of EP2 and EP4 receptor-mediated diseases. WO2022257960A1, 2022.
5. Spennacchio, M., et al. A unified flow strategy for the preparation and use of trifluoromethyl-heteroatom anions. *Science* **2024**, *385*, 991-996.
